# Access to unsaturated bicyclic lactones by overriding conventional C(*sp*^3^)–H site selectivity

**DOI:** 10.1038/s41557-023-01295-x

**Published:** 2023-08-10

**Authors:** Jayabrata Das, Wajid Ali, Animesh Ghosh, Tanay Pal, Astam Mandal, Chitrala Teja, Suparna Dutta, Rajagopal Pothikumar, Haibo Ge, Xinglong Zhang, Debabrata Maiti

**Affiliations:** 1 Department of Chemistry, Indian Institute of Technology Bombay, Mumbai, India; 2 Department of Chemistry and Biochemistry, Texas Tech University, Lubbock, TX USA; 3 Institute of High Performance Computing (IHPC), Agency for Science, Technology and Research (A*STAR), Singapore, Singapore

**Keywords:** Synthetic chemistry methodology, Synthetic chemistry methodology

## Abstract

Transition metal catalysis plays a pivotal role in transforming unreactive C–H bonds. However, regioselective activation of distal aliphatic C–H bonds poses a tremendous challenge, particularly in the absence of directing templates. Activation of a methylene C–H bond in the presence of methyl C–H is underexplored. Here we show activation of a methylene C–H bond in the presence of methyl C–H bonds to form unsaturated bicyclic lactones. The protocol allows the reversal of the general selectivity in aliphatic C–H bond activation. Computational studies suggest that reversible C–H activation is followed by β-hydride elimination to generate the Pd-coordinated cycloalkene that undergoes stereoselective C–O cyclization, and subsequent β-hydride elimination to provide bicyclic unsaturated lactones. The broad generality of this reaction has been highlighted via dehydrogenative lactonization of mid to macro ring containing acids along with the C–H olefination reaction with olefin and allyl alcohol. The method substantially simplifies the synthesis of important bicyclic lactones that are important features of natural products as well as pharmacoactive molecules.

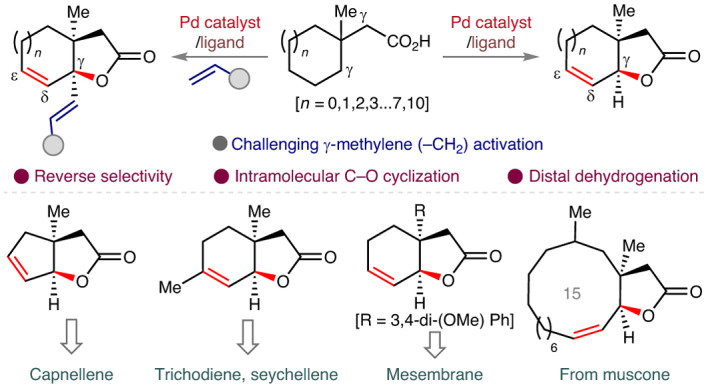

## Abstract (web summary)

Bicyclic lactones are valuable motifs for the synthesis of natural products and bioactive molecules. Now, a palladium-catalysed protocol has been developed to access unsaturated bicyclic lactones in one step from corresponding carboxylic acids. The method demonstrates reverse site selectivity for C(*sp*^3^)–H activation to form diverse bicyclic cores.

## Main

**Fig. 1:**
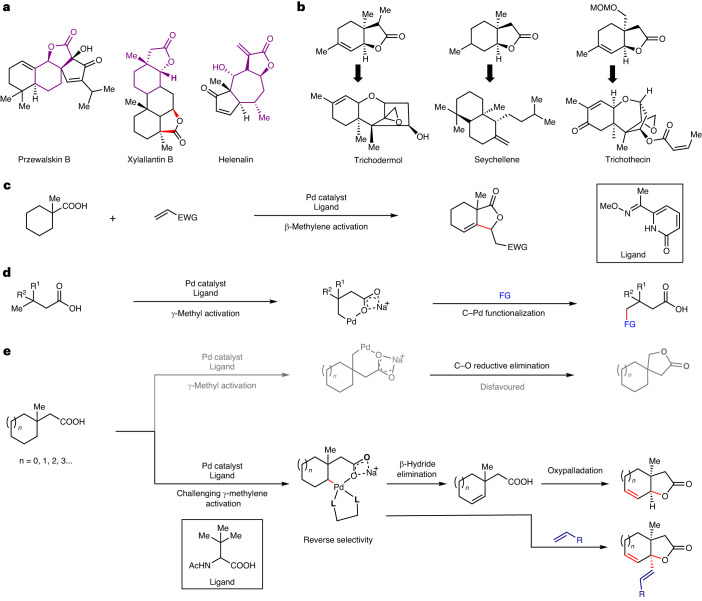
Relevance of bicyclic lactones and state of the art in native carboxylate-assisted γ-C(*sp*^3^)–H activation. **a**, Representative natural products containing bicyclic lactones. **b**, Selected examples of key bicyclic lactone intermediates for synthesis of various natural products. **c**, Existing approach for reverse site selectivity at the proximal β-position of aliphatic carboxylic acid. EWG, electron withdrawing group. Distal γ-methylene activation in the presence of γ-methyl is unexplored. **d**, Conventional approach for γ-functionalization of aliphatic carboxylic acids. FG, functional group. **e**, γ-Methylene activation in preference to facile methyl group activation provides a generic platform to obtain unsaturated bicyclic lactones via an intramolecular and intermolecular way (this work). Bidentate ligand *N*-Ac-^*t*^Leu has been represented as L,L in the intermediate.

Bicyclic lactones comprise a large number of natural products with diverse biological activities (Fig. 1a). Constructing these cyclic architectures has been a topic of great interest among synthetic chemists not just because of their abundance in nature, but also because of their utility as versatile synthons (Fig. 1b)^1,2^. Numerous efforts have been made to prepare bicyclic lactones using different synthetic strategies^3^. Nonetheless, traditional synthetic approaches suffer from atom and step economy due to the complex starting material preparation, multi-step procedures, unwanted side product formations and so on. The past two decades witnessed a notable advance in synthetic organic chemistry, where unactivated C–H bonds were selectively transformed into complex organic molecules. Transition metal catalysis has become a powerful tool to convert unreactive aliphatic C–H bonds into an array of useful functionalities^4–6^. Nonetheless, selective transformation of distal aliphatic C–H bonds still presents an uphill challenge, more so in the absence of an exogenous directing group^7^ that is added externally to the substrate. In this context, aliphatic acids have been identified as the choice of substrates that could be functionalized without utilizing any extra directing group. However, the weak coordination of the carboxylate group has been shown to activate mostly the methyl group at the β or γ positions, limiting the scope and applicability of aliphatic acids^8–11^. In this Article, we sought to develop a simpler and more effective method to access bicyclic lactones that could overcome the existing limitations. Alkyl carboxylic acids have come up as one of the most promising substrates to transform into valuable entities via Pd-catalysed C(*sp*^3^)–H activation. Exploiting the weak coordination of the carboxylate group for challenging activation of C(*sp*^3^)–H bonds offers great advantage as it sidesteps the necessity of using an external directing group. Recent years have witnessed a great progress in this domain where a number of transformations have been achieved at the proximal and distal sites of aliphatic carboxylic acids^12–16^. However, C–H activation was carried out mostly at the terminal methyl group. To push the boundary further, we envisaged if weak coordination of carboxylate could induce challenging methylene activation in the presence of the equally accessible methyl group. In this context, β-methylene C–H activation in the presence of methyl has been explored recently (Fig. 1c)^17,18^. However, such reverse site selectivity is yet to be explored in the more distant sites. Most existing methods report *γ*-C(*sp*^3^)–H functionalization at the terminal methyl sites (Fig. 1d)^19,20^. Reaching out to remote methylene C–H bonds without altering the equally accessible methyl C–H bond would give access to a new range of products starting from readily available aliphatic carboxylic acids. Furthermore, such reversal of conventional selectivity by the ubiquitous carboxylate group represents a notable challenge for the continuous advancement of the field.

## Results and discussion

### Reaction development for unsaturated bicyclic lactone formation

To realize our idea of accessing bicyclic lactones via methylene activation from aliphatic acids, we chose 3-methyl cyclohexane acetic acid as a model substrate to perform the reaction as it has equally accessible γ-methyl and γ-methylene groups. We hypothesized that, although methyl activation would be more facile than methylene in this case, the subsequent C–O reductive elimination from the C(methyl)–H activated intermediate may be unfavourable (Fig. 1e). On the contrary, methylene activation in the cyclic acid substrate could be followed by a facile β-hydride elimination that is conventional from a Pd(II) intermediate. β-Hydride elimination would furnish an alkenoic acid intermediate that is highly likely to undergo oxypalladation to form bicyclic unsaturated lactones (Fig. 1e). Herein, we report the successful attainment of all these ideas via a C–H activation mode that activates the challenging distal methylene group in preference to more facile methyl group activation. Thus, C–H activation, C–O cyclization and dehydrogenation take place in one pot, giving rise to a unique class of chemistry. The combination of C(*sp*^3^)–H activation and oxypalladation presented here generates unsaturated lactones in one step. Computational studies support our hypothesis on the favourability of the formation of the methylene activation product over the methyl activation product, consistent with our experimental results. The addition of an olefin or allyl alcohol in the reaction mixture, interestingly, results in the formation of the unsaturated γ-methylene olefinated bicyclic unsaturated lactone, providing a unique class of products with quaternary carbon via C–H activation. It is worth mentioning that acyclic aliphatic acids do not undergo lactonization under our reaction condition probably due to floppy conformational degrees of freedom that make it harder to form the subsequent intermediates. Notably, iodolactonization is one of the most utilized approaches known to form unsaturated bicyclic lactones that requires two steps from starting alkenoic carboxylic acids^21,22^. Larock has reported formation of unsaturated lactones starting from alkenoic acids^23^. These previous strategies begin with an olefin-containing carboxylic acid substrate. In contrast, our approach starts with completely saturated carboxylic acid substrates and the double bond is constructed in situ, giving rise to bicyclic unsaturated lactones. Unsaturated lactones are of great importance as they can be utilized to synthesize complex natural products or bioactive molecules by manipulating the double bond in the ring. Numerous interesting biological properties of halo lactones, hydroxyl lactones or epoxy lactones are well known in the literature that are prepared from unsaturated bicyclic lactones^24,25^. By harnessing the weak coordination of carboxylate for strenuous methylene C–H activation/oxypalladation, a range of unsaturated bicyclic lactones can become accessible by a highly step and atom efficient approach that is unexplored previously. This palladium-catalysed reaction can greatly simplify the formation of such lactones and enable a generic platform to synthesize complex bioactive molecules in an atom and step economical manner.

We started our studies by treating 3-methyl cyclohexane acetic acid with 10 mol% Pd(OAc)_2_, 20 mol% *N*-Ac-Leu, 2 equiv. Ag_2_CO_3_ and 2 equiv. Na_2_HPO_4_ in 1,1,1,3,3,3-hexafluoro-2-propanol (HFIP) solvent for 24 h. We observed 24% isolated yield of the methylene activated product with no methyl activation product at all under the aforementioned conditions. We reasoned that, if the observed result is ligand controlled, changing the ligand could improve the reactivity. We searched for ligands that may remarkably improve the reactivity for methylene activation. It turned out that changing the side alkyl chain of the α-amino acids led to minor improvement. Among mono-protected α-amino acids such as *N*-Ac-Gly, *N*-Ac-Ala, *N*-Ac-Val, *Ν*-Ac-Ile, *N*-Ac-Nle and so on, *Ν*-Ac-Ile improved the yield at best up to 36%. Changing the ligand from α- to β-amino acids such as *N*-Ac-β-Ala or *Ν*-Ac-β-Phe-Ala did not improve the reactivity. Further, we endeavoured to understand the effect of steric bulk on the backbone of the amino acids. *N*-Ac-Phe-Ala that contains a phenyl group in the side chain improved yield up to 44%, suggesting that a bulky substituent at the α-position of amino acid is beneficial for the reaction. Encouraged by this result, we examined the influence of *N*-Ac-^*t*^Leu on the reaction as it contains a bulky ^*t*^butyl group on the side chain. Gratifyingly, the yield was improved to 66% with *N*-Ac-^*t*^Leu as a ligand in the reaction. Other protecting groups in amino acids such as *N*-Boc, *N*-Cbz and *N*-Bz were incompatible with our protocol and did not form the product even in trace amount. It is worth mentioning that other commonly used ligands in *sp*^*3*^ C−H activation such as pyridone, pyridine, phenanthroline and so on remained fruitless in this reaction (for details, see Supplementary Information Section 3). Subsequently, we tested various combinations of the Pd catalyst, oxidant and alkali metal base to improve the product conversion. It was found that the alkali metal base plays a crucial role in carboxylate-assisted C(*sp*^3^)–H activation reactions as it facilitates C–H activation by binding with carboxylate in a *κ*^2^ coordination mode and thus Pd gets the opportunity to activate the accessible C–H bonds^26^. Among various alkali metal bases such as Na, K, Cs, Li and so on, Na^+^-based bases remained the best ones for this protocol. A detailed optimization revealed that the combination of 10 mol% Pd(OAc)_2_, 20 mol% of *N*-Ac-^*t*^Leu, 2 equiv. of Ag_2_CO_3_ and 2 equiv. Na_3_PO_4_ in HFIP solvent for 24 h at 120 °C rendered maximum conversion (73%) of the lactone product. After finding the optimized reaction condition, we set out to explore the scope of the protocol.

We began by assessing the generality of the reaction with respect to a range of substituted cyclohexane-containing carboxylic acids (Table 1). An array of diverse substituents at the β-position of the acid, starting from shorter methyl to longer octyl chain, were tolerated in the reaction (**2a**–**2g**) giving excellent yields of bicyclic unsaturated lactones. Notably, only a single diastereomer is formed in the cases of shorter chain such as methyl to propyl. However, for longer-chain substituents at the β-site, formation of diastereomers was observed (**2e** and **2g**). Substitution at the α-site of the acid also remained unaffected in the reaction and provided the desired product in good yields (**2h**). Changing the β-substituents from an alkyl to a cycloalkane did not affect the outcome of the methodology and formed desired lactones in good yields and diastereomeric ratio (**2i**–**2j**). Aryl groups with electronically diverse β-substituents were well compatible with the protocol despite the reactive aryl C–H bonds (**2k**–**2n**). We further characterized one of the aryl substituted lactones, which shows syn stereochemistry, by X-ray diffraction (**2k**). Next, we evaluated the effect of substituents on the cyclohexane ring to check if it affects the reaction in any manner. We were delighted to find that substituents at the 2- and 3- positions did not affect the reaction and in fact increased the yield of products to some extent (**2o**–**2r**). Aryl substitutions at the 3-position of cyclohexane furnished bicyclic lactones in excellent yields without affecting the aryl C–H bonds. However, in these cases, we observed formation of regioisomers as the major and minor product. Conformational analysis has been performed to elucidate the outcome of regioisomers (Supplementary Information Section 9.9). Notably, electron donating or withdrawing groups on the aryl ring had no effect on the outcome of the reaction (**2s**–**2x**). We also successfully characterized these lactones by X-ray diffraction which discloses the *syn* stereochemistry of the substituents at the fused site (**2u**, **2w** and **2x**). It is interesting to note that the stereochemistry of the formed bicyclic lactones is *syn* as it is thermodynamically more stable than *anti*^3^. The *syn* stereochemistry of fused lactones is also kinetically more accessible than the *anti*-stereochemistry, as demonstrated by our computational studies (Supplementary Information Section 9.6). We were curious to inspect the effect of 4-substituted cyclohexanes as in these cases, double bond forming in the product would be substituted. We wondered whether in such cases substitutions would particularly affect the reactions. Substitutions at the four positions did affect the reactions as yields decreased in these cases to some extent. Nonetheless, the reaction outcome remained the same irrespective of the substituents like methyl, propyl, ^*t*^butyl, ^*t*^pentyl, trifluoromethyl and phenyl (**2y**–**2af**) (Table 1). After obtaining a broad generality of the reaction with respect to cyclohexane-containing aliphatic acids, we further investigated if this methodology is equally effective to form different ring size bicyclic lactones. [5,5], [7,5] Fused bicyclic lactones are part of many natural products just like [6,6] bicyclic lactones^27–30^. Therefore, we started exploring the compatibility of cyclopentane-containing acids in the reaction. In these cases, [5,5] fused bicyclic unsaturated lactones formed in synthetically useful yields and good diastereoselectivity from their corresponding acids (Table 2). A number of substituents at the β-position such as methyl, ethyl, isopropyl, cyclohexane and so on were tested, and all of these substrates afforded the corresponding lactones in good yields (**3a**–**3d**). Having substitution in the cyclopentane ring itself also did not prevent the reaction from forming fused lactones (**3e**–**3f**). Next, we moved to check the compatibility of the protocol with a larger ring size acid like cycloheptane-containing acids. In these cases, the [7,5] bicyclic unsaturated lactones were formed in good yields irrespective of substituents in the starting materials (**3g**–**3l**). All of these results further spurred us to inspect the effectiveness of the protocol for the lactonization of macro rings such as 12- and 15-membered rings. There are no straightforward routes in the literature to prepare bicyclic lactones such as [12,5], [15,5] fused lactones in an efficient manner. Gratifyingly, employing our method, we successfully obtained such macro bicyclic lactones in synthetically useful yields (**3m**–**3p**) (Table 2), and these further underscore the synthetic versatility of this C–H activation reaction. Muscone, a natural product obtained from deer musk, has been successfully lactonized utilizing our protocol (**3q**).

**Table 1:**
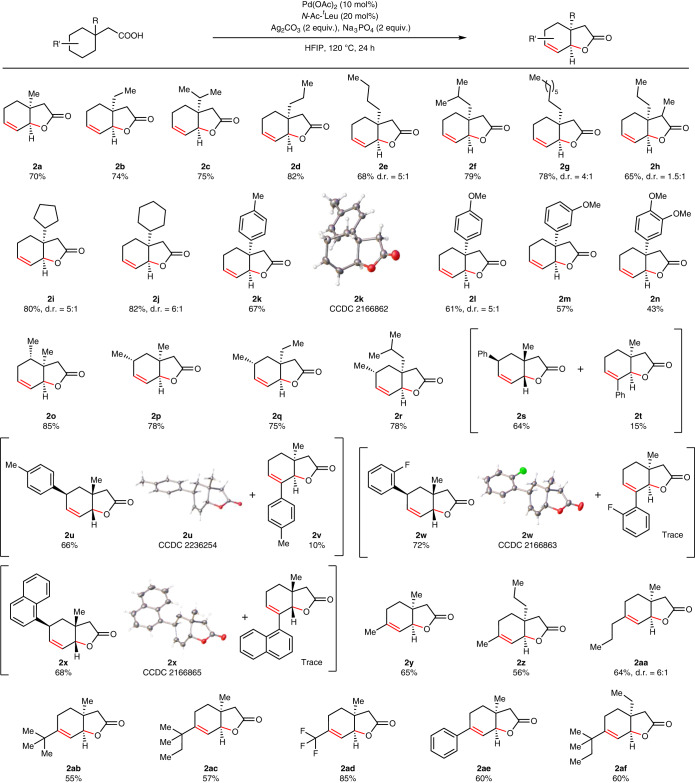
Examples of cyclohexane acetic acids that undergo unsaturated lactone formation

All reactions were performed at 0.2 mmol scale and isolated yields are reported. Standard condition: acid (0.2 mmol), Pd(OAc)_2_ (10 mol%), *N*-Ac-^*t*^Leu (20 mol%), Ag_2_CO_3_ (2 equiv.), Na_3_PO_4_ (2 equiv.), HFIP (1.5 ml), 120 °C, 24 h. All products shown are diastereomers only (not enantiomers).

**Table 2:**
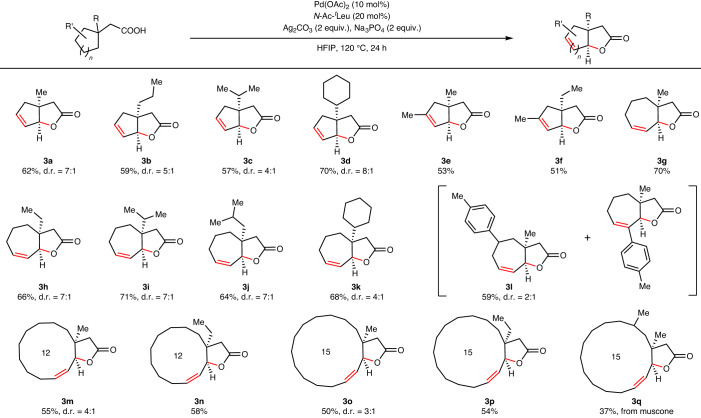
Examples of 5-, 6-, 7-, 12- and 15-membered cycloalkane acetic acids that undergo unsaturated lactone formation

All reactions were conducted at 0.2 mmol scale and isolated yields are reported. Standard condition: acid (0.2 mmol), Pd(OAc)_2_ (10 mol%), *N*-Ac-^*t*^Leu (20 mol%), Ag_2_CO_3_ (2 equiv.), Na_3_PO_4_ (2 equiv.), HFIP (1.5 ml), 120 °C, 24 h. All products shown are diastereomers only (not enantiomers).

The success of this intramolecular reaction further prompted us to explore the reactivity under an intermolecular condition. We reasoned that, if the intramolecular reaction goes via a six-membered C–H activated intermediate, then an external coupling partner should intercept the palladated intermediate to provide a highly functionalized product. If this occurs, our approach would provide access to a quaternary carbon centre in one step from a methylene centre via a tandem process involving C(*sp*^3^)–H activation and allylic C–H activation. A quaternary centre, that is, a molecular substructure consisting of a carbon atom bound to four other carbon(hetero) atoms, is a characteristic feature of numerous natural products and therefore constitutes an important goal in the context of synthetic methodology development^31,32^. The presence of quaternary centres in a molecule can have prominent chemical and biological impact. Our idea was to utilize coupling partners that would not hinder either the lactone formation or dehydrogenation in the product which we have obtained without using any coupling partner. To test our hypothesis, we explored the reactivity of acids with olefins under the similar reaction condition. To our delight, we obtained bicyclic unsaturated lactones with γ-methylene olefinated products. The method presented here offers cyclization–dehydrogenation–olefination in a single-pot reaction and converts a methylene centre to a quaternary carbon centre by forming a C–O and C–C bond simultaneously, highlighting the uniqueness of the C–H activation mode in this protocol. Such multiple bond formation in a single step remains an important goal of synthetic chemists to form elaborated molecules, which our present protocol has achieved. To examine the scope of the olefination reaction, we employed a range of olefins as well as aliphatic acids in the reaction. Various cycloalkane-containing carboxylic acids were also utilized in the reaction. Substituents such as Me, Et, butyl, cyclohexane and so on at the β-position of acid did not hamper the reaction and formed products in good to excellent yields (**4a**–**4e**) (Table 3). Similarly, acids containing substituents at the cyclohexane ring were also compatible with the protocol providing *γ*-olefinated unsaturated bicyclic lactones in useful yields (**4f**–**4h**). In cases of two and three substituted cyclohexanes, we observed some diastereomeric ratios. Similar reactivity has been observed for acrylates containing Me, Et and Bu groups (**4g**–**4j**). Notably, in the case of alkyl substituents at the β-site of the cycloalkane acids (**4b**, **4d**, **4e** and **4h**), where C(methylene)–H can occur in the Et/Pr/Bu/^*i*^Bu groups, the C(methylene)–H activation still occurs within the cyclohexane to furnish the desired products. Acrylates containing simple alkyl groups such as Me, Bu, fluoroalkyl, benzyl and so on provided corresponding products in excellent yields (**4i**–**4l**). Halogen-substituted acrylates also worked well under the reaction condition (**4m**–**4n**). Besides acrylates, we also explored methyl vinyl ketone in the reaction, which afforded the olefinated lactone product in good yield (**4p**). Allyl alcohols are another class of coupling partners that have been utilized extensively over the years in C–H functionalization reactions. Although allyl alcohols resemble olefins such as acrylates or vinyl ketones to some extent, they are less activated compared with the former. Because of the unique reactivity of allyl alcohols, these compounds are capable of undergoing multiple reaction pathways to provide different products^33,34^. Mainly, allyl alcohols have been used either as alkylating or allylating reagents in various C–H activation reactions. We set out to investigate the potential of allyl alcohols as external coupling partners with cycloalkane acids that undergo reaction intramolecularly to form bicyclic lactone in our case. With allyl alcohols, we obtained γ-olefinated bicyclic lactones where –OH in the allyl alcohol undergoes oxidation. A number of differently substituted cyclohexane acetic acids were tested with allyl alcohols to examine the generality of the protocol. Substitutions at various positions of the acid substrate did not affect the outcome or yields of the formed bicyclic lactones (**4q**–**4u**). Allyl fragments such as 3-buten-2-ol, unsubstituted allyl alcohol, 1-penten-3-ol and even long chain containing 1-octen-3-ol were evaluated as coupling partners, and all of them furnished products in excellent yields (**4v**–**4y**). The scope of allyl alcohols was not just limited to primary or secondary allyl alcohols; tertiary allyl alcohol has also been utilized as a suitable coupling partner in this intermolecular reaction where the OH group in the alcohol remains unaltered in the product (**4z**).

**Table 3:**
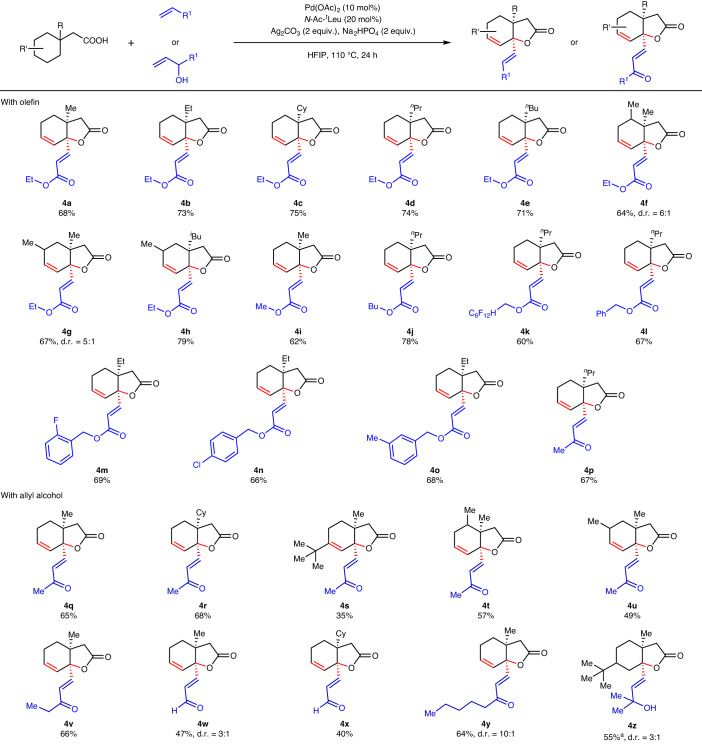
Intermolecular reaction of cyclohexane acetic acids with various olefins and allyl alcohols

All reactions were conducted at 0.2 mmol scale and isolated yields are reported. Reaction condition: acid (0.2 mmol), olefin/allyl alcohol (2 equiv.), Pd(OAc)_2_ (10 mol%), *N*-Ac-^*t*^Leu (20 mol%), Ag_2_CO_3_ (2 equiv.), Na_2_HPO_4_ (2 equiv.), HFIP (2 ml), 110 °C, 24 h. ^a^Reaction carried out at 100 °C.

### Application of the developed method

**Fig. 2:**
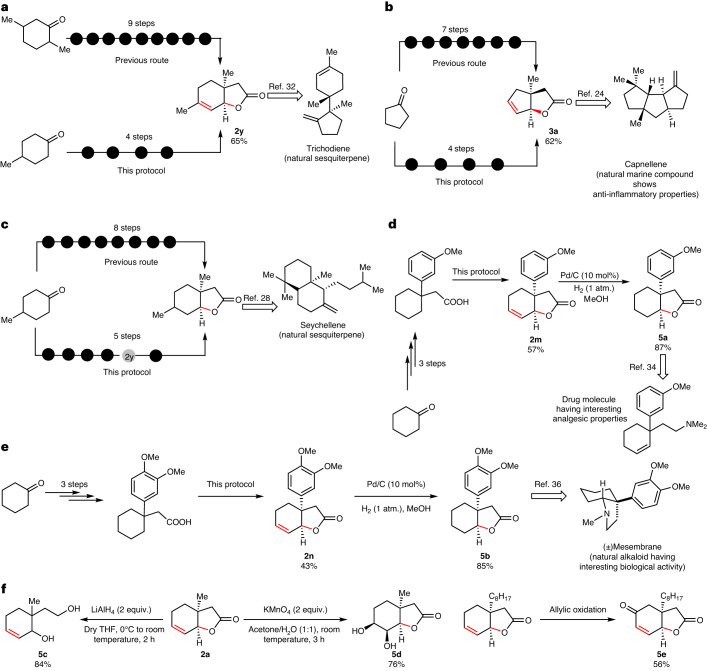
Application of the developed protocol in formal synthesis of natural products and bioactive compounds. **a**, Formal synthesis of trichodiene, a natural sesquiterpene hydrocarbon of the tricothecane family of antibiotics and mycotoxins, through the formation of a bicyclic lactone intermediate in four steps from readily available 4-methyl cyclohexanone. From 4-methyl cyclohexanone the acid substrate was first prepared in three steps followed by cyclization utilizing the developed method. **b**, Formal synthesis of capnellene by forming a bicyclic lactone intermediate in four steps starting from abundant cyclopentanone as a starting material. Capnellene is a marine compound that has anti-inflammatory properties. **c**, Synthesizing a lactone intermediate for the formal synthesis of seychellene, a natural sesquiterpene. **d**, Formal synthesis of 2-(1-(*m*-methoxyphenyl)-2-cyclohexen-1-yl)-*N,N*-dimethylethylamine that showcases analgesic properties. **e**, Formal synthesis of a natural alkaloid, Mesembrane. Mesembrane has diverse biological activities. **f**, One-step transformation of unsaturated bicyclic lactones to different synthons. The unsaturated lactones could be converted to diol **5d**, 1,4-diol **5c** and allylic oxidized product **5e** under mild conditions.

To realize the potential of this developed method, we explored its synthetic utility in constructing important bicyclic lactones that are key intermediates in synthesizing natural products such as terpenoids, and bioactive molecules. Trichodiene, a sesquiterpene hydrocarbon, possesses a wide variety of biological activities. The synthesis of trichodiene goes via a bicyclic intermediate that was previously synthesized in at least nine steps^35^. In contrary, now the same intermediate could be synthesized using this protocol in just four steps, considerably improving the ease of synthesizing such lactones (Fig. 2a). Another interesting natural product is capnellene, a marine compound having anti-inflammatory properties. A [5,5] bicyclic lactone is a key intermediate for its synthesis^27^. Employing the iodolactonization reaction, this bicyclic lactone was previously synthesized in seven steps from cyclopentanone. By comparison, we synthesized the same bicyclic lactone in just four steps from cyclopentanone (Fig. 2b). We identified natural sesquiterpenes seychellene and its derivative isoseychellne that are prepared through a bicyclic lactone intermediate. In the previous route, it was prepared in eight steps from 4-methyl cyclohexanone utilizing an iodolactonization reaction^36^. Our method can deliver the same compound in just five steps starting from the same 4-methyl cyclohexanone (Fig. 2c). 3-Methoxy aryl substituted [6,5] bicyclic lactone, an intermediate for the total synthesis of a drug molecule with analgesic properties, was previously constructed in six steps^37^. We have synthesized the same bicyclic lactone in five steps with 51% yield from readily available cyclohexanone (Fig. 2d). Mesembrane, a natural alkaloid from Amaryllidaceae family, shows impressive diversity of biological activity^38^. We synthesized a crucial intermediate of Mesembrane in five steps starting from cyclohexanone, providing an efficient alternative route to synthesize Mesembrane (Fig. 2e)^39^. Further, the presence of a lactone and double bond in the products allows ample opportunities to manipulate the bicyclic lactones to form valuable molecules. Reduction (**5c**), hydroxylation (**5d**) and oxidation (**5e**) are just some examples that we have shown here (Fig. 2f). Given the convenience and widespread utility of this method, we believe it will find extensive applications in the synthesis of versatile bicyclic lactones en route to the synthesis of complex natural products in a much simplified manner.

**Fig. 3:**
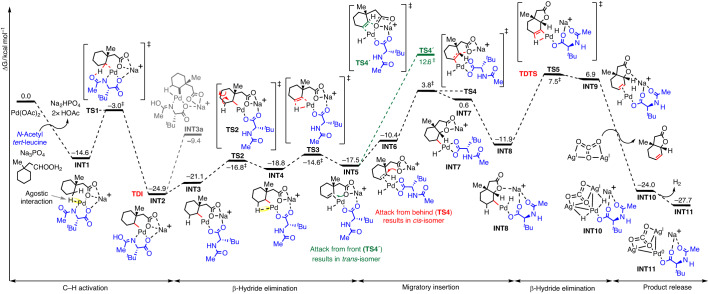
Gibbs free energy profile computed at SMD(HFIP)-MN15/def2-QZVP//MN15/(def2-TZVPPD for Pd, Ag + def2-SVP for all other atoms). All values are quoted in kcal mol^−1^. The catalytic cycle proceeds via C–H activation, then β-H elimination, followed by migratory insertion and a second β-H elimination to yield the final product. TDI, turnover frequency-determining intermediate; TDTS, turnover frequency-determining transition state.

### Mechanistic details

**Fig. 4:**
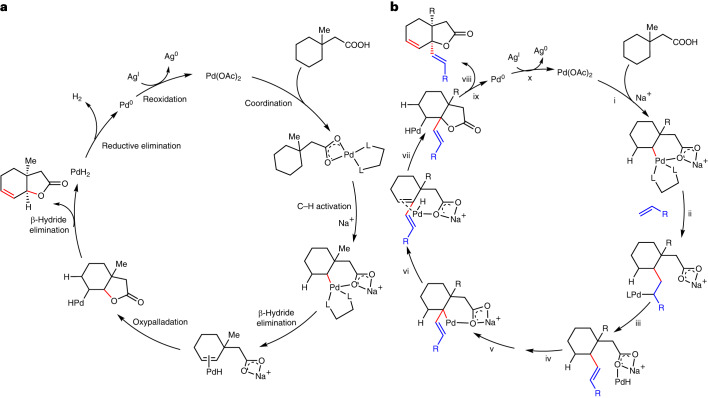
Proposed reaction mechanisms for unsaturated bicyclic lactone formation. **a**, Reaction mechanism for the formation of unsaturated bicyclic lactones without any external coupling partner (intramolecular). L,L represents the bidentate ligand *N*-Ac-^*t*^Leu used in the reaction. **b**, Reaction mechanism for the formation of olefin-containing unsaturated bicyclic lactone in the presence of olefin or allyl alcohol (intermolecular). (i) C–H activation, (ii) olefin 1,2-insertion, (iii) β-hydride elimination, (iv) reductive elimination followed by reoxidation by Ag(I) to form Pd(II), (v) allyl C–H activation, (vi) β-hydride elimination, (vii) oxypalladation, (viii) β-hydride elimination, (ix) reductive elimination, (x) reoxidation.

In order to understand the intricacies of the reaction pathway for bicyclic unsaturated lactone formation, we carried out a computational study of the reaction. Density functional theory (DFT) calculations^40^ (Fig. 3) suggest that the reaction proceeds firstly with the mono-protected amino acid (MPAA)-assisted C(methylene)–H activation (**TS1**) via concerted metalation deprotonation, forming a [5,6]-palladacyclic transition state (TS)^41–45^ conducive for C–H bond cleavage. Similar MPAA-assisted C–H activation has been shown to have a lower barrier than the acetate-assisted (non-MPAA) C–H activation in the Pd-catalysed reactions^46,47^. Although the activation of the methyl group is lower by 3.4 kcal mol^−1^ than the methylene group, the subsequent reductive elimination from the C(methyl)–H activated complex has a very high barrier at 45.3 kcal mol^−1^, making this pathway highly unfavourable (Supplementary Information Section 9.3). As a result, C(methyl)–H activation is predicted to be reversible, consistent with the experimental evidence where the use of deuterated HFIP resulted in considerable deuterium incorporation (59%) at the methyl site (Supplementary Information Section 10). The C(methylene)–H activated complex **INT2** undergoes a rotational transition via **TS2** and positions the β-H closer to the Pd centre for subsequent elimination via **TS3**, thereby forming the cyclohexene C=C bond, **INT5**. The existence of this cyclohexene intermediate was corroborated experimentally when such substrate is subjected to our protocol and formed the lactone product in the same way as using cyclohexane substrate (Supplementary Information Section 12). From **INT2** and **INT3**, potential reductive C–O coupling could occur. However, computational studies indicated that such C–O reductive eliminations have unfavourably high barriers (>45 kcal mol^–1^, **TS3a** and **TS3b**; Supplementary Section 9 and Supplementary Figs. 5 and 6) and are much less kinetically competent than β-hydride elimination step (**TS3**, barrier of 10.3 kcal mol^–1^). Next, migratory insertion of the C=C bond into the Pd–O occurs stereoselectively, either via **TS4** to give the observed *cis*-fused bicyclic lactone or via **TS4**′ to give the *trans*-fused lactone. This is the stereo-determining step of the catalytic reaction where **TS4** has a lower barrier of 8.8 kcal mol^−1^ than **TS4**′ (Supplementary Information Section 9.6). Finally, the oxidatively inserted intermediate **INT8** undergoes another β-H elimination via **TS5** to yield the final product (Supplementary Information Section 9.7). **TS5** has the highest barrier of the whole catalytic cycle and is the turnover frequency-determining transition state (TDTS)^48^. The energetic span of this transformation is 32.4 kcal mol^–1^, translating to a half-life of 50 min, assuming first-order kinetics, at 120 °C reaction temperature, consistent with the experimentally observed reactivity. Finally, product release is assisted with silver carbonate that coordinates to the Pd centre as the lactone product is released (Supplementary Information Section 9.8). A possible loss of H_2_ could give Pd(0) species and subsequent redox reaction can occur to regenerate the Pd(II) catalyst (Fig. 4). The elimination of H_2_ has been experimentally detected using TCD-GC (Supplementary Information Section 11).

## Conclusion

The results presented here signify important advancement in carboxylate-directed distal C–H activation. Remote methylene activation has been achieved by overriding the usual methyl activation in aliphatic acids. We have exhibited both the intramolecular version of the reaction to form unsaturated bicyclic lactones and the intermolecular version with olefins and allyl alcohols to obtain γ-olefinated lactones where a quaternary centre is generated from a methylene one in a single step. Taken together, the outcomes of this protocol could substantially broaden the domain of native functional group directed C–H activation. Considering the prevalence of bicyclic lactones in myriads of natural products, bioactive and pharmacoactive molecules, we expect our protocol to be broadly beneficial in complex molecule synthesis.

## Methods

### Unsaturated bicyclic lactone formation

In an oven-dried screw-capped reaction tube was charged with magnetic stir bar, corresponding acid (0.2 mmol), Pd(OAc)_2_ (10 mol%), *N*-Ac-^t^Leu (20 mol%), Ag_2_CO_3_ (2 equiv.) and Na_3_PO_4_ (2 equiv.) in 1.5 ml (for 0.2 mmol scale) of HFIP were added. The reaction tube was capped and placed in a heating bath at 120 °C with stirring (800 rpm) for 24 h. Upon completion the mixture was diluted with EtOAc and filtered through a celite pad. The filtrate was evaporated under reduced pressure and the crude mixture was purified by column chromatography using silica (100–200 mesh size) and petroleum ether/ethyl acetate as the eluent.

### Unsaturated olefinated bicyclic lactone formation

In an oven-dried screw-capped reaction tube was charged with magnetic stir bar, corresponding acid (0.2 mmol), Pd(OAc)_2_ (10 mol%), *N*-Ac-^t^Leu (20 mol%), Ag_2_CO_3_ (2 equiv.), Na_2_HPO_4_ (2 equiv.) and olefin or allyl alcohol (2 equiv.) in 1.8 ml (for 0.2 mmol scale) of HFIP were added. The reaction tube was capped and placed in a heating bath at 110 °C with stirring (800 rpm) for 24 h. Upon completion the mixture was diluted with EtOAc and filtered through a celite pad. The filtrate was evaporated under reduced pressure and the crude mixture was purified by column chromatography using silica (100–200 mesh size) and petroleum ether/ethyl acetate as the eluent.

## Online content

Any methods, additional references, Nature Portfolio reporting summaries, source data, extended data, supplementary information, acknowledgements, peer review information; details of author contributions and competing interests; and statements of data and code availability are available at 10.1038/s41557-023-01295-x.

### Supplementary information

Supplementary Information Supplementary Tables 1–7, discussion and Figs. 1–15.

Supplementary Data 1 Crystallographic data for compound **2k**; CCDC reference 2166862.

Supplementary Data 2 Crystallographic data for compound **2u**; CCDC reference 2236254.

Supplementary Data 3 Crystallographic data for compound **2w**; CCDC reference 2166863.

Supplementary Data 4 Crystallographic data for compound **2x**; CCDC reference 2166865.

Supplementary Data 5 Atomic coordinates of optimized structures from DFT calculations.

## Data Availability

All data supporting the findings of this study including experimental procedures, computational details and compound characterization are available in Supplementary Information. Atomic coordinates of optimized structures are available as Supplementary Data 5. Crystallographic data for the structures **2k**, **2u**, **2w** and **2x** reported in this article have been deposited at the Cambridge Crystallographic Data Centre under the deposition numbers CCDC 2166862, 2236254, 2166863 and 2166865, respectively. Copies of the data can be obtained free of charge via https://www.ccdc.cam.ac.uk/structures/. DFT optimized structures have been deposited online and are freely available at https://zenodo.org/record/7516355.
