# Supplementary material for: Access to unsaturated bicyclic lactones by overriding conventional C(sp3)–H site selectivity
Source: Nat Chem. 2023 Aug 10;15(11):1626–35. doi: 10.1038/s41557-023-01295-x (PMC10624629; doi:10.1038/s41557-023-01295-x)

nature chemistry

Article https://doi.org/10.1038/s41557-023-01295-x

Access to unsaturated bicyclic lactones by overriding conventional C(sp^3)–H site selectivity

In the format provided by the authors and unedited

List of Contents

1. General Consideration	2
2. Preparation of Starting Materials	3
3. Optimization Details	4-7
4. X-ray Crystallographic Data	8-11
5. Spectral Data of the Starting Material	12-29
6. General Procedures for the Lactonization	30
7. Analysis of the Crude Reaction Mixture	31
8. Spectral Data of the Unsaturated Bicyclic Lactones	32-72
9. DFT Calculation	73-100
10. Deuterium Exchange and Intermediate Competency	100-101
11. H_2 Gas Detection	101-102
12. Plausible Reaction mechanisms	103
13. Control Experiment	104-105
14. NMR Spectra of the Starting Material	106-155
15. NMR Spectra of the Unsaturated Bicyclic Lactones	156-256
16. NMR Spectra of Alkenoic Acid	257

1. General Consideration:

Reagent Information. Unless otherwise stated, all reactions were carried out in screw cap reaction tubes. All the solvents were bought from commercial sources and were used without further purification. Palladium salts and olefins were purchased from Aldrich and TCI-India. Silica gel (100–200 mesh) obtained from SRL Co. was used for column chromatography. Products and starting materials were visualized on TLC plate (Merck, TLC silica gel 60 F254) using UV-light or by staining with $KMnO_4$ solution, followed by heating. A gradient elution using petroleum ether and ethyl acetate was performed, based on Merck aluminium TLC sheets (silica gel 60F_{254}).

Analytical Information. All compounds are characterized by ^{1}H NMR, ^{13}C NMR spectroscopy, and HR-MS. Copies of the ^{1}H NMR, ^{13}C NMR and ^{19}F can be found in the Supporting Information. Unless otherwise stated, all Nuclear Magnetic Resonance spectra were recorded on a Bruker 500 MHz / 400 MHz instrument. All ^{1}H NMR experiments are reported in units, parts per million (ppm), and were measured relative to the signals for residual chloroform (7.26 ppm) in the deuterated solvent, unless otherwise stated. All ^{13}C NMR spectra were reported in ppm relative to deuterochloroform (77.230 ppm), unless otherwise stated, and all were obtained with ^{1}H decoupling. High-resolution mass spectra (HRMS) were recorded on a micro-mass ESI TOF (time of flight) mass spectrometer.

2. Preparation of Cycloalkyl Acetic Acids

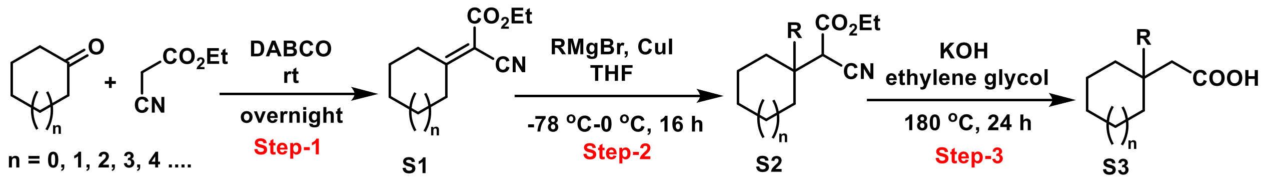

Step 1: Knoevenagel Condensation (S1)

To a round bottom flask with a magnetic stir bar, corresponding ketone (5.0 mmol), ethyl cyanoacetate (6.0 mmol), and 1,4-diazabicyclo[2.2.2]octane (DABCO, 10 mol%) were added. The resulting reaction mixture was stirred for overnight at room temperature. After completion of the reaction the mixture was diluted with EtOAc and washed with water and brine. Combined organic layer was dried over anhydrous Na_2SO_4, filtered, and concentrated in vacuo. The crude product was purified through flash column chromatography using Hexane/EtOAc as the eluent to yield S1 in quantitatively.

Step 2: Grignard Reaction (S2)

To a round bottom flask with a magnetic stir bar was added copper iodide (1.0 equiv.) and THF solvent followed by the dropwise addition of alkyl magnesium bromide in THF or hexane (2.0 equiv.) at -25 °C. The resulting mixture was stirred for 2 h at the same temperature, then corresponding **S1** in THF was added slowly. The reaction mixture was slowly warmed to room temperature and stirred for overnight. The reaction was quenched with sat. NH_4Cl (aq.) and filtered through a pad of celite. The resulting reaction mixture was diluted with EtOAc and washed with water and brine. The combined organic layers were dried over anhydrous Na_2SO_4, filtered, and concentrated in vacuo. The crude product was purified through flash column chromatography using Hexane/EtOAc as the eluent to provide corresponding **S2** in the range of 40-95% yield.

Step 3: Base Mediated Decarboxylative Hydrolysis (S3)

A clean, oven-dried screw cap reaction tube with previously placed magnetic stir–bar was charged with **S2** (2.0 mmol), KOH (10.0 equiv.), followed by addition of ethylene glycol (2 mL). The reaction mixture sealed tightly and was vigorously stirred for 24 h in a preheated oil bath at 180 °C. After stipulated time, the reaction mixture was cooled to room temperature and diluted with water. Diluted reaction mixture was acidified with 2N HCl up to pH 3.0 and then extracted with EtOAc three times. The combined organic layers were dried over anhydrous Na_2SO_4 and concentrated in vacuo. The crude product was purified through flash column chromatography using Hexane/EtOAc as the eluent to provide corresponding acid **S3**.

3. Optimization:

Yield and selectivity were determined by [1]*H NMR analysis of the crude product using internal standard.*

Table S1: Pd-catalyst optimization

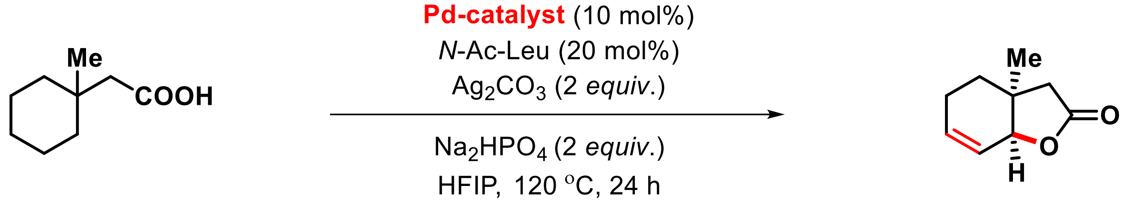

Entry	Catalyst	Yield (%)
**1**	**$Pd(OAc)_2$**	**24**
2	$Pd(PPh_3)_2Cl_2$	trace
3	$Pd(PhCN)_2Cl_2$	8
4	$Pd(allyl)_2Cl_2$	9
5	$Pd(TFA)_2$	17
6	$Pd(OPiv)_2$	20
7	$Pd(COD)_2Cl_2$	16
8	$Pd(PPh_3)_4$	trace
9	$Pd(acac)_2$	21
10	$Pd(CH_3CN)_2Cl_2$	11
11	$[Pd_2(dba)_2]_3$	trace

Table S2: Temperature optimization

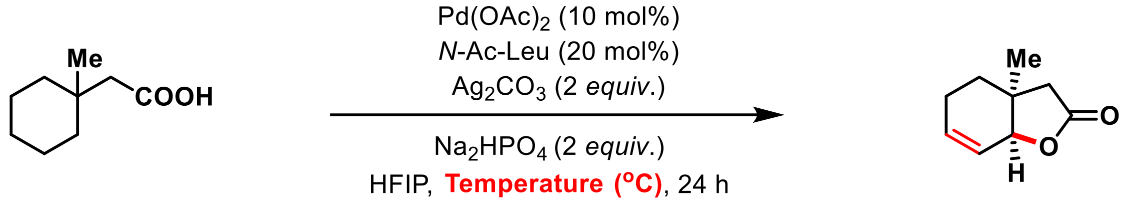

Entry	Temp. (°C)	Yield (%)
1	80	Trace
2	90	<10
3	100	13
4	110	20
**5**	**120**	**24**
6	130	23
7	140	19

Table S3: Ligand optimization

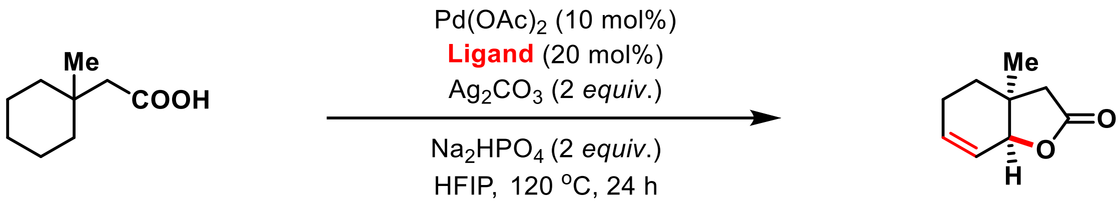

Entry	Ligand	Yield (%)
1	*N*-Ac-Leu	24
2	*N*-Boc-Leu	NR
3	*N*-Ac-Ile	36
4	*N*-Boc-Ile	NR
5	*N*-Ac-Gly	13
6	*N*-Ac-Ala	19
7	*N*-Ac-Val	14
8	*N*-Ac-Nle	17
9	*N*-Ac-*β*-Ala	trace
10	*N*-Ac-*β*-Phe-Ala	trace
11	*N*-Ac-Anthranilic acid	trace
12	*N*-Ac-Ph-Ala	44
**13**	***N*-Ac-^{t}Leu**	**66**
14	*N*-Boc-^{t}Leu	trace
15	*N*-Cbz-^{t}Leu	NR
16	*N*-Bz-^{t}Leu	NR
17	2-Pyridone	NR
18	pyridine	NR
19	phenanthroline	NR
20	8-nitroquinoline	NR
21	4-hydroxy pyridine	NR
22	2-hydroxy-5(trifluoromethyl) pyridine	NR

Table S4: Ligand amount optimization

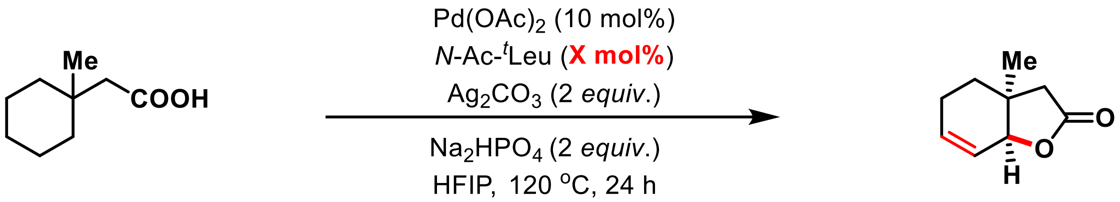

Entry	Amount (mol%)	Yield (%)
1	10	45
2	15	51
**3**	**20**	**66**
4	25	67
5	30	64
6	35	56
7	40	48

Table S5: Oxidant optimization

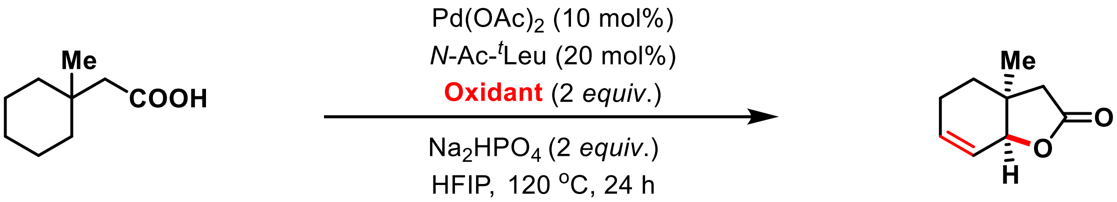

Entry	Oxidant	Yield (%)
1	AgOAc	53
**2**	**Ag_2CO_3**	**66**
3	Ag_2O	42
4	Ag_2SO_4	25
5	AgTFA	15
6	Ag_3PO_4	trace
7	$Cu(OAc)_2$	26

Table S6: Base optimization

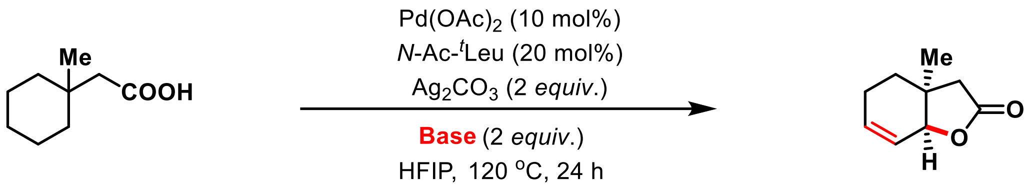

Entry	Base	Yield (%)
1	CsCl	26
2	$NaIO_4$	trace
3	K_2HPO_4	42
4	Na_2CO_3	66
5	K_2CO_3	51
6	$Na_2S_2O_8$	32
**7**	**Na_3PO_4**	**73**
8	Na_3PO_4 (3 eq.)	69
9	Cs_2CO_3	64
10	Na_2HPO_4 (1 eq.)	56
11	Na_2HPO_4 (1.5 eq.)	62
12	Na_2HPO_4 (2 eq.)	66

Table S7: Time optimization

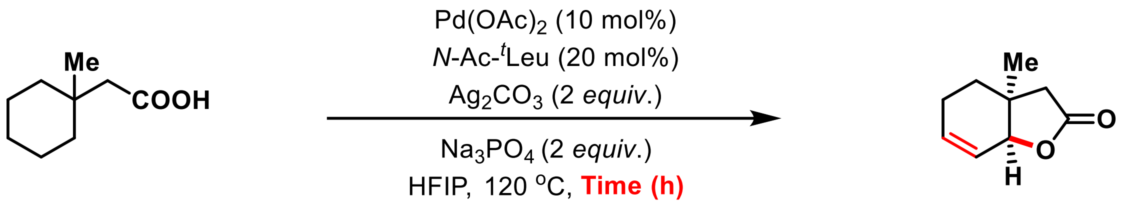

Entry	Time (h)	Yield (%)
1	12	42
2	16	53
**3**	**24**	**73**
4	30	68
5	48	57

4. X-ray Crystallographic Data:

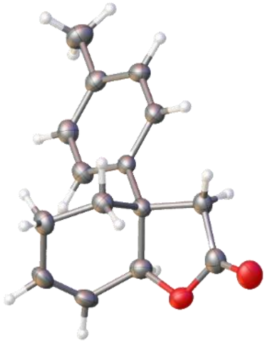

Crystal Data of 2k CCDC 2166862:

Crystal data and structure refinement for $C_{15}H_{16}O_2$	
Identification code	**2k**
Formula	$C_{15}H_{16}O_2$
Formula weight(g/mol)	228.28
Temperature/K	150 K
Crystal system	Monoclinic
Space group	P 1 21/c 1
a/Å	6.2421(4)
b/Å	19.2352(11)
c/Å	9.9231(8)
α/°	90
β/°	100.931(7)
γ/°	90
Volume/Å^3	1169.83(14)
Z	4
P_{calc}g/cm^3	1.296
μ/mm^{-1}	0.085
F(000)	488.0
Crystal size/mm^3	0.115 x 0.098 x 0. 0.092
Radiation	MoKα (λ = 0.71073)
2Θ range for data collection/°	3.1360 to 29.9700
Goodness-of-fit on F^2	1.025
R indices (all data)	R1 = 0.0542, wR2 = 0.1472
Largest diff. peak and hole/ e Å^{-3}	0.662 and -0.271

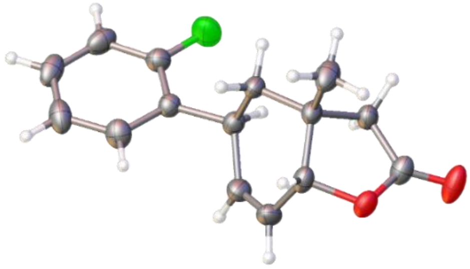

Crystal Data of 2w CCDC 2166863:

Crystal data and structure refinement for $C_{15}H_{16}FO_2$	
Identification code	**2w**
Formula	$C_{15}H_{16}FO_2$
Formula weight(g/mol)	246.27
Temperature/K	150 K
Crystal system	Orthorhombic
Space group	P 21 21 21
a/Å	6.6014(3)
b/Å	9.6883(5)
c/Å	19.9271(11)
α/°	90
β/°	90
γ/°	90
Volume/Å^3	1274.46(11)
Z	4
P_{calc}g/cm^3	1.283
μ/mm^{-1}	0.094
F(000)	520.0
Crystal size/mm^3	0.112 x 0.098 x 0. 0.095
Radiation	MoKα (λ = 0.71073)
2Θ range for data collection/°	3.1360 to 29.9700
Goodness-of-fit on F^2	1.056
R indices (all data)	R1 = 0.0373, wR2 = 0.0863
Largest diff. peak and hole/ e Å^{-3}	0.126 and -0.129

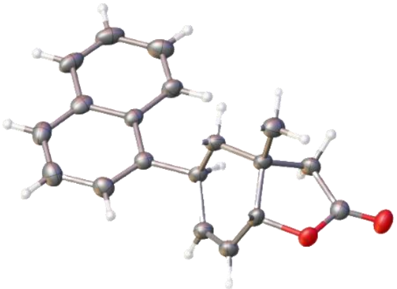

Crystal Data of 2x CCDC 2166865:

Crystal data and structure refinement for $C_{19}H_{18}O_2$	
Identification code	**2x**
Formula	$C_{19}H_{18}O_2$
Formula weight(g/mol)	278.33
Temperature/K	150 K
Crystal system	Monoclinic
Space group	P 1 21 1
a/Å	11.0225(9)
b/Å	6.4677(4)
c/Å	11.3616(10)
α/°	90
β/°	116.111(11)
γ/°	90
Volume/Å^3	727.31(12)
Z	2
P_{calc}g/cm^3	1.271
μ/mm^{-1}	0.081
F(000)	296.0
Crystal size/mm^3	0.112 x 0.102 x 0. 0.096
Radiation	MoKα (λ = 0.71073)
2Θ range for data collection/°	3.1360 to 29.9700
Goodness-of-fit on F^2	0.993
R indices (all data)	R1 = 0.0548, wR2 = 0.1359
Largest diff. peak and hole/ e Å^{-3}	0.172 and -0.272

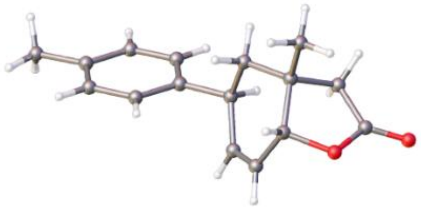

Crystal Data of 2u CCDC: 2236254

Crystal data and structure refinement for $C_{19}H_{18}O_2$	
Identification code	**2u**
Formula	$C_{16}H_{18}O_2$
Formula weight(g/mol)	242.30
Temperature/K	150 K
Crystal system	Monoclinic
Space group	P 1 21 1
a/Å	6.4447(2)
b/Å	99.350(3)
c/Å	10.0261(3)
$\alpha/^o$	90
$\beta/^o$	116.111(11)
$\gamma/^o$	90
Volume/Å^3	1340.83(7)
Z	4
$P_{calc}g/cm^3$	1.200
μ/mm^{-1}	0.078
F(000)	520.0
Crystal size/mm^3	0.11 x 0.098 x 0. 0.095
Radiation	MoKα (λ = 0.71073)
2Θ range for data collection/^{o}	1.937 to 25.0000
Goodness-of-fit on F^2	0.993
R indices (all data)	R1 = 0.144, wR2 = 0.1273
Largest diff. peak and hole/ e Å^{-3}	0.336 and -0.170

5. Spectral Data of the Starting Material:

2-(1-Methylcyclohexyl)acetic acid (1)

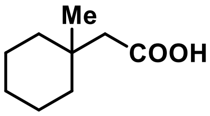

Eluent: ethyl acetate/ petroleum ether (90:10 v/v).

Appearance: colorless gummy

Isolated yield: 81% (254 mg, 1.61 mmol)

^{1}H NMR (400 MHz, $CDCl_3$) δ 2.27 (s, 2H), 1.63 – 1.22 (m, 10H), 1.05 (s, 3H); **^{13}C NMR** (101 MHz, $CDCl_3$) δ 179.38, 45.97, 38.02, 33.52, 26.29, 25.59, 22.18; **HRMS** (ESI): calculated for $C_9H_{17}O_2$ $[M+H]^+$: 157.1229; observed mass 157.1232.

2-(1-Methylcyclopentyl)acetic acid (2)

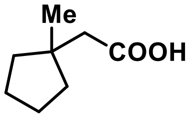

Eluent: ethyl acetate/ petroleum ether (90:10 v/v).

Appearance: colorless gummy.

Isolated yield: 72% (205 mg, 1.44 mmol)

^{1}H NMR (400 MHz, $CDCl_3$) δ 2.34 (s, 2H), 1.66 (qd, *J* = 5.1, 4.6, 1.9 Hz, 4H), 1.61 – 1.52 (m, 2H), 1.46 (dddd, *J* = 12.2, 6.7, 4.6, 3.0 Hz, 2H), 1.09 (s, 3H); **^{13}C NMR** (101 MHz, $CDCl_3$) δ 179.14, 45.98, 41.45, 39.64, 26.18, 24.31; **HRMS (ESI)**: Calculated mass for $C_8H_{15}O_2$ $[M+H]^+$: 143.1072; observed mass: 143.1075.

2-(1,2-Dimethylcyclohexyl)acetic acid (3)

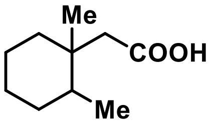

Eluent: ethyl acetate/ petroleum ether (10:90 v/v).

Appearance: colorless semisolid.

Isolated yield: 91% (310 mg, 1.82 mmol)

^{1}H NMR (400 MHz, $CDCl_3$) δ 2.60 – 2.02 (m, 2H), 1.72 (ddtd, *J* = 44.5, 12.6, 3.8, 1.5 Hz, 2H), 1.56 – 0.92 (m, 10H), 0.83 (d, *J* = 6.8 Hz, 3H); **^{13}C NMR** (126 MHz, $CDCl_3$) δ 179.96, 46.90, 42.13, 39.14, 37.72, 37.23, 36.93, 36.70, 36.27, 30.89, 30.67, 27.24, 26.27, 26.13, 22.36,

22.28, 18.04, 16.42, 16.10; **HRMS (ESI)**: Calculated mass for $C_{10}H_{19}O_2$ $[M+H]^+$: 171.1385; observed mass: 171.1390.

2-(1,3-Dimethylcyclohexyl)acetic acid (4)

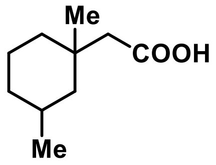

Eluent: ethyl acetate/ petroleum ether (10:90 v/v).

Appearance: colorless solid.

Isolated yield: 75% (205 mg, 1.21 mmol)

^{1}H NMR (400 MHz, $CDCl_3$) δ 2.34 (s, 2H), 1.75 – 1.33 (m, 6H), 1.16 – 0.97 (m, 4H), 0.85 (d, *J* = 6.4 Hz, 3H), 0.82 – 0.70 (m, 2H); **^{13}C NMR** (101 MHz, $CDCl_3$) δ 179.13, 47.26, 41.31, 37.75, 35.04, 34.11, 30.10, 28.07, 23.07, 22.26; **HRMS (ESI)**: Calculated mass for $C_{10}H_{19}O_2$ $[M+H]^+$: 171.1385; observed mass: 171.1387.

2-(1-Ethylcyclohexyl)acetic acid (5)

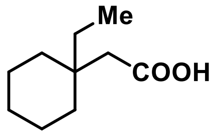

Eluent: ethyl acetate/ petroleum ether (12:88 v/v).

Appearance: colorless semisolid.

Isolated yield: 80% (272 mg, 1.6 mmol)

^{1}H NMR (400 MHz, $CDCl_3$) δ 2.29 (s, 2H), 1.64 – 1.18 (m, 12H), 0.85 (t, *J* = 7.5 Hz, 3H); **^{13}C NMR** (101 MHz, $CDCl_3$) δ 179.24, 41.26, 36.21, 35.58, 30.15, 26.37, 21.85, 7.68; **HRMS (ESI)**: Calculated mass for $C_{10}H_{18}NaO_2$ $[M+Na]^+$: 193.1204; observed mass: 193.1210.

2-(1-Methylcycloheptyl)acetic acid (6)

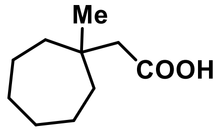

Eluent: ethyl acetate/ petroleum ether (10:90 v/v).

Appearance: white solid.

Isolated yield: 68% (231 mg, 1.36 mmol)

^{1}H NMR (500 MHz, $CDCl_3$) δ 2.23 (s, 2H), 1.87 – 1.34 (m, 12H), 1.04 (s, 3H); **^{13}C NMR** (126 MHz, $CDCl_3$) δ 178.96, 46.99, 40.63, 37.74, 36.68, 30.72, 28.30, 27.83, 23.06, 22.80; **HRMS (ESI)**: Calculated mass for $C_{10}H_{19}O_2$ $[M+H]^+$: 171.1385; observed mass: 171.1378.

2-(1-Ethyl-3-methylcyclohexyl)acetic acid (7)

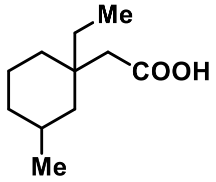

Eluent: ethyl acetate/ petroleum ether (8:92 v/v).

Appearance: colorless gummy.

Isolated yield: 73% (270 mg, 1.46 mmol)

^{1}H NMR (400 MHz, $CDCl_3$) δ 2.38 (s, 2H), 1.83 – 1.49 (m, 5H), 1.49 – 1.29 (m, 3H), 1.03 (td, J = 13.3, 4.1 Hz, 1H), 0.94 – 0.79 (m, 6H), 0.82 – 0.66 (m, 2H); **^{13}C NMR** (101 MHz, CDCl3) δ 179.08, 44.70, 37.92, 36.98, 35.36, 35.25, 34.69, 27.84, 23.21, 22.06, 7.66; **HRMS (ESI)**: Calculated mass for $C_{11}H_{21}O_2$ $[M+H]^+$: 185.1542.; observed mass: 185.1550.

2-(1-Methyl-4-(*tert*-pentyl)cyclohexyl)acetic acid (8)

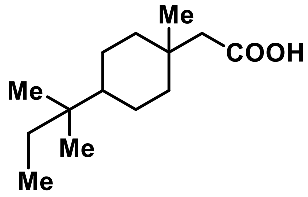

Eluent: ethyl acetate/ petroleum ether (12:88 v/v).

Appearance: white solid.

Isolated yield: 64% (290 mg, 0.64 mmol)

^{1}H NMR (500 MHz, $CDCl_3$) δ 2.31 (s, 2H), 1.85 – 1.67 (m, 2H), 1.64 – 1.45 (m, 2H), 1.25 (q, J = 7.5 Hz, 2H), 1.17 (t, J = 8.8 Hz, 4H), 1.03 (s, 4H), 0.78 (d, J = 6.5 Hz, 9H); **^{13}C NMR** (101 MHz, $CDCl_3$) δ 179.44, 45.28, 40.72, 38.75, 34.88, 33.22, 32.86, 29.76, 24.52, 22.50, 8.31; **HRMS (ESI)**: Calculated mass for $C_{14}H_{27}O_2$ $[M+H]^+$: 227.2011; observed mass: 227.2008.

2-(1-Isopropylcyclohexyl)acetic acid (9)

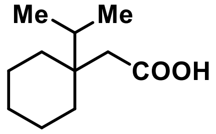

Eluent: ethyl acetate/ petroleum ether (10:90 v/v).

Appearance: colorless gummy.

Isolated yield: 71% (261 mg, 1.42 mmol)

^{1}H NMR (400 MHz, $CDCl_3$) δ 2.34 (s, 2H), 1.90 (hept, J = 6.9 Hz, 1H), 1.62 – 1.27 (m, 10H), 0.87 (d, J = 6.9 Hz, 6H); **^{13}C NMR** (101 MHz, CDCl3) δ 179.92, 38.86, 38.16, 32.54, 32.23, 26.29, 21.74, 16.92, 16.90; **HRMS (ESI)**: Calculated mass for $C_{11}H_{21}O_2$ $[M+H]^+$: 185.1542; observed mass: 185.1550.

2-(4-(*tert*-Butyl)-1-methylcyclohexyl)acetic acid (10)

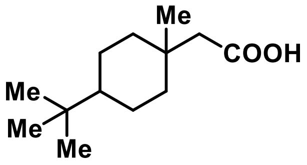

Eluent: ethyl acetate/ petroleum ether (10:90 v/v).

Appearance: white solid.

Isolated yield: 67% (299 mg, 1.34 mmol)

^{1}H NMR (400 MHz, $CDCl_3$) δ 2.32 (s, 2H), 1.78 – 1.68 (m, 2H), 1.64 – 1.51 (m, 2H), 1.22 – 1.11 (m, 4H), 1.04 (s, 3H), 0.96 (m, 1H), 0.85 (s, 9H); **^{13}C NMR** (101 MHz, $CDCl_3$) δ 179.37, 48.14, 40.68, 38.68, 33.14, 32.62, 29.75, 27.78, 22.96; **HRMS (ESI)**: Calculated mass for $C_{13}H_{25}O_2$ $[M+H]^+$: 213.1855; observed mass: 213.1860.

2-(1-Propylcyclohexyl)acetic acid (11)

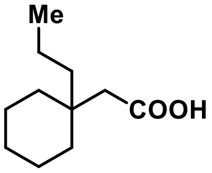

Eluent: ethyl acetate/ petroleum ether (10:90 v/v).

Appearance: colorless gummy.

Isolated yield: 75% (276 mg, 1.5 mmol)

^{1}H NMR (500 MHz, $CDCl_3$) δ 2.30 (s, 2H), 1.62 – 1.32 (m, 12H), 1.32 – 1.21 (m, 2H), 0.90 (t, J = 7.1 Hz, 3H); **^{13}C NMR** (126 MHz, $CDCl_3$) δ 179.28, 41.95, 36.23, 36.02, 26.35, 21.85, 16.42, 15.10; **HRMS (ESI)**: Calculated mass for $C_{11}H_{21}O_2$ $[M+H]^+$: 185.1542; observed mass: 185.1540.

2-(1,4-Dimethylcyclohexyl)acetic acid (12)

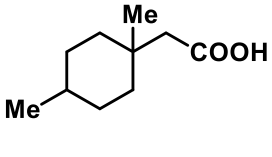

Eluent: ethyl acetate/ petroleum ether (10:90 v/v).

Appearance: whitish solid.

Isolated yield: 63% (215 mg, 1.26 mmol)

^{1}H NMR (400 MHz, $CDCl_3$) δ 2.33 (s, 2H), 1.77 – 1.59 (m, 2H), 1.58 – 1.47 (m, 2H), 1.40 – 1.07 (m, 5H), 1.05 (s, 3H), 0.90 (d, J = 6.5 Hz, 3H); **^{13}C NMR** (101 MHz, $CDCl_3$) δ 179.48, 41.22, 37.89, 33.06, 32.41, 30.80, 29.43, 22.44; **HRMS (ESI)**: Calculated mass for $C_{10}H_{19}O_2$ $[M+H]^+$: 171.1385; observed mass: 171.1379.

2-(1-Ethylcycloheptyl)acetic acid (13)

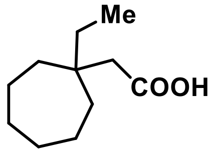

Eluent: ethyl acetate/ petroleum ether (12:88 v/v).

Appearance: colorless gummy.

Isolated yield: 61% (225 mg, 1.22 mmol)

^{1}H NMR (400 MHz, $CDCl_3$) δ 2.23 (s, 2H), 1.49 (dqd, J = 22.3, 7.7, 4.1 Hz, 14H), 0.86 (t, J = 7.5 Hz, 3H); **^{13}C NMR** (101 MHz, $CDCl_3$) δ 179.66, 43.20, 39.44, 38.49, 32.20, 30.90, 23.00, 8.35; **HRMS (ESI)**: Calculated mass for $C_{11}H_{20}NaO_2$ $[M+Na]^+$: 207.1361; observed mass: 207.1365.

2-(1-Propylcyclopentyl)acetic acid (14)

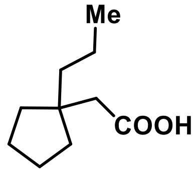

Eluent: ethyl acetate/ petroleum ether (10:90 v/v).

Appearance: colorless gummy.

Isolated yield: 64% (219 mg, 1.28 mmol)

^{1}H NMR (400 MHz, $CDCl_3$) δ 2.33 (s, 2H), 1.70 – 1.53 (m, 5H), 1.48 (tdd, J = 7.0, 4.8, 2.7 Hz, 2H), 1.44 – 1.37 (m, 2H), 1.36 – 1.23 (m, 3H), 0.90 (t, J = 7.0 Hz, 3H); **^{13}C NMR** (101 MHz, $CDCl_3$) δ 179.05, 44.90, 42.58, 41.70, 37.88, 24.56, 18.33, 15.09; **HRMS (ESI)**: Calculated mass for $C_{10}H_{18}NaO_2$ $[M+Na]^+$: 193.1204; observed mass: 193.1210.

2-(1-Methylcyclododecyl)acetic acid (15)

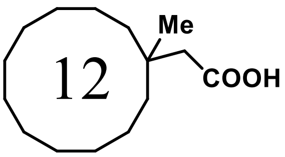

Eluent: ethyl acetate/ petroleum ether (12:88 v/v).

Appearance: colorless semisolid.

Isolated yield: 52% (250 mg, 1.04 mmol)

^{1}H NMR (400 MHz, $CDCl_3$) δ 2.19 (s, 2H), 1.34 (d, *J* = 13.3 Hz, 22H), 1.00 (s, 3H); **^{13}C NMR** (101 MHz, $CDCl_3$) δ 178.88, 45.34, 36.11, 34.51, 26.91, 26.40, 25.46, 22.90, 22.37, 19.35; **HRMS (ESI)**: Calculated mass for $C_{15}H_{29}O_2$ $[M+H]^+$: 241.2168; observed mass: 241.2170.

2-(1-Ethyl-4-(*tert*-pentyl)cyclohexyl)acetic acid (16)

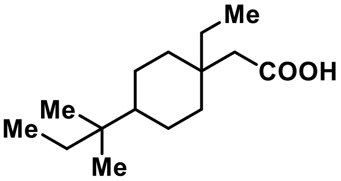

Eluent: ethyl acetate/ petroleum ether (10:90 v/v).

Appearance: white solid.

Isolated yield: 50% (240 mg, 1.0 mmol)

^{1}H NMR (400 MHz, $CDCl_3$) δ 2.35 (s, 2H), 1.75 – 1.64 (m, 2H), 1.58 – 1.50 (m, 2H), 1.38 (q, *J* = 7.5 Hz, 2H), 1.26 (q, *J* = 7.5 Hz, 2H), 1.21 – 1.02 (m, 5H), 0.87 (t, *J* = 7.5 Hz, 3H), 0.79 (d, *J* = 5.3 Hz, 9H); **^{13}C NMR** (101 MHz, $CDCl_3$) δ 178.35, 45.61, 37.15, 36.21, 36.01, 34.91, 34.47, 32.85, 24.51, 22.29, 8.31, 7.80; **HRMS (ESI)**: Calculated mass for $C_{15}H_{28}NaO_2$ $[M+Na]^+$: 263.1987; observed mass: 263.1990.

2-(1-Propylcyclohexyl)propanoic acid (17)

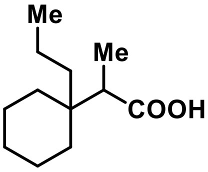

Eluent: ethyl acetate/ petroleum ether (10:90 v/v).

Appearance: colorless gummy.

Isolated yield: 51% (201 mg, 1.02 mmol)

^{1}H NMR (400 MHz, $CDCl_3$) δ 2.62 (q, *J* = 7.1 Hz, 1H), 1.63 – 1.23 (m, 14H), 1.08 (d, *J* = 7.1 Hz, 3H), 0.90 (t, *J* = 7.1 Hz, 3H); **^{13}C NMR** (101 MHz, $CDCl_3$) δ 182.50, 45.15, 37.78, 35.48, 32.12, 31.95, 29.92, 26.25, 21.73, 21.62, 16.29, 15.23, 11.60; **HRMS (ESI)**: Calculated mass for $C_{12}H_{23}O_2$ $[M+H]^+$: 199.1698; observed mass: 199.1700.

2-(1-Butylcyclohexyl)acetic acid (18)

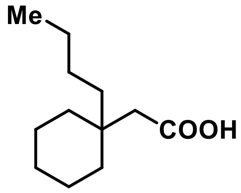

Eluent: ethyl acetate/ petroleum ether (10:90 v/v).

Appearance: colorless gummy.

Isolated yield: 82% (325 mg, 1.64 mmol)

^{1}H NMR (400 MHz, $CDCl_3$) δ 2.30 (s, 2H), 1.51 – 1.33 (m, 10H), 1.31 – 1.16 (m, 6H), 0.90 (t, *J* = 6.9 Hz, 3H); **^{13}C NMR** (101 MHz, $CDCl_3$) δ 179.49, 42.00, 37.53, 36.08, 36.03, 26.35, 25.39, 23.67, 21.86, 14.32; **HRMS (ESI)**: Calculated mass for $C_{12}H_{23}O_2$ $[M+H]^+$: 199.1698; observed mass: 199.1690.

2-(1-Methyl-3-phenylcyclohexyl)acetic acid (19)

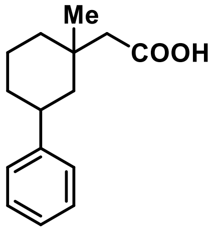

Eluent: ethyl acetate/ petroleum ether (15:85 v/v).

Appearance: white solid.

Isolated yield: 78% (361 mg, 1.56 mmol)

^{1}H NMR (500 MHz, $CDCl_3$) δ 7.29 (t, *J* = 7.5 Hz, 2H), 7.20 (dd, *J* = 17.0, 7.7 Hz, 3H), 2.76 (ddd, *J* = 12.6, 9.1, 3.5 Hz, 1H), 2.54 (d, *J* = 13.1 Hz, 1H), 2.44 (d, *J* = 13.0 Hz, 2H), 1.96 – 1.81 (m, 1H), 1.79 – 1.71 (m, 1H), 1.62 (dd, *J* = 14.0, 3.5 Hz, 1H), 1.49 – 1.18 (m, 4H), 1.12 (s, 3H); **^{13}C NMR** (126 MHz, $CDCl_3$) δ 179.02, 147.26, 128.59, 128.57, 127.08, 127.06, 126.19, 46.10, 46.08, 41.23, 39.61, 39.58, 37.83, 37.81, 34.45, 34.43, 33.58, 33.55, 30.17, 30.14, 22.49, 22.47; **HRMS (ESI)**: Calculated mass for $C_{15}H_{21}O_2$ $[M+H]^+$: 233.1542; observed mass: 233.1545.

2-(1-Methyl-4-phenylcyclohexyl)acetic acid (20)

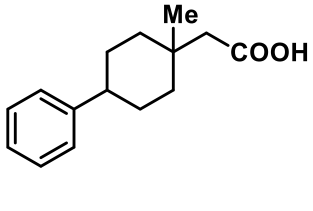

Eluent: ethyl acetate/ petroleum ether (15:85 v/v).

Appearance: white solid.

Isolated yield: 76% (354 mg, 1.52 mmol)

^{1}H NMR (400 MHz, $CDCl_3$) δ 7.30 (t, *J* = 7.5 Hz, 2H), 7.23 (d, *J* = 7.6 Hz, 2H), 7.21 – 7.16 (m, 1H), 2.65 – 2.34 (m, 3H), 1.85 – 1.71 (m, 4H), 1.65 (qd, *J* = 13.2, 3.1 Hz, 2H), 1.38 (td, *J* = 13.3, 3.8 Hz, 2H), 1.13 (s, 3H); **^{13}C NMR** (101 MHz, $CDCl_3$) δ 178.38, 147.31, 128.56, 127.03, 126.19, 44.24, 40.69, 38.39, 32.97, 29.84, 29.77; **HRMS (ESI)**: Calculated mass for $C_{15}H_{21}O_2$ $[M+H]^+$: 233.1542; observed mass: 233.1540.

2-(1-(*p*-Tolyl)cyclohexyl)acetic acid (21)

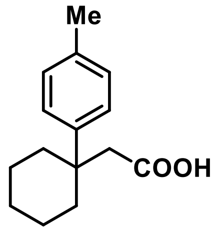

Eluent: ethyl acetate/ petroleum ether (15:85 v/v).

Appearance: colorless liquid.

Isolated yield: 61% (283 mg, 1.22 mmol)

^{1}H NMR (400 MHz, $CDCl_3$) δ 7.24 (d, *J* = 8.1 Hz, 2H), 7.13 (d, *J* = 8.0 Hz, 2H), 2.51 (s, 2H), 2.33 (s, 3H), 2.28 – 2.10 (m, 2H), 1.87 – 1.68 (m, 2H), 1.55 (dt, *J* = 8.7, 4.8 Hz, 2H), 1.52 – 1.35 (m, 4H); **^{13}C NMR** (101 MHz, $CDCl_3$) δ 177.45, 142.09, 135.54, 129.24, 126.68, 48.35, 40.55, 36.14, 26.36, 22.48, 21.13; **HRMS (ESI)**: Calculated mass for $C_{15}H_{21}O_2$ $[M+H]^+$: 233.1542; observed mass: 233.1539.

2-(1-(4-Methoxyphenyl)cyclohexyl)acetic acid (22)

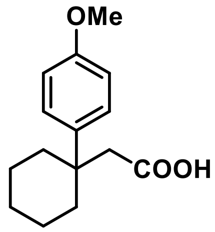

Eluent: ethyl acetate/ petroleum ether (20:80 v/v).

Appearance: white solid.

Isolated yield: 60% (300 mg, 1.2 mmol)

^{1}H NMR (500 MHz, $CDCl_3$) δ 7.26 (dd, *J* = 6.0, 3.9 Hz, 2H), 7.05 – 6.74 (m, 2H), 3.79 (d, *J* = 1.9 Hz, 3H), 2.50 (s, 2H), 2.17 (dd, *J* = 14.0, 6.4 Hz, 2H), 1.76 (t, *J* = 10.8 Hz, 2H), 1.54 (d, *J* = 6.8 Hz, 2H), 1.48 – 1.29 (m, 4H); **^{13}C NMR** (126 MHz, $CDCl_3$) δ 157.77, 127.88, 113.80,

55.33, 40.27, 36.34, 26.39, 22.45; **HRMS (ESI)**: Calculated mass for $C_{15}H_{21}O_3$ $[M+H]^+$: 249.1491; observed mass: 249.1495.

2-(4-Methyl-1-propylcyclohexyl)acetic acid (23)

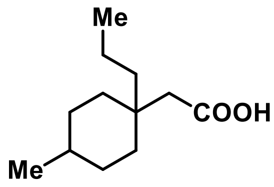

Eluent: ethyl acetate/ petroleum ether (10:90 v/v).

Appearance: colorless gummy.

Isolated yield: 75% (298 mg, 1.5 mmol)

^{1}**H NMR** (400 MHz, $CDCl_3$) δ 2.37 (s, 2H), 1.73 – 1.58 (m, 2H), 1.57 – 1.47 (m, 2H), 1.37 – 1.25 (m, 5H), 1.25 – 1.05 (m, 4H), 0.89 (dd, *J* = 5.7, 2.9 Hz, 6H); ^{13}**C NMR** (101 MHz, $CDCl_3$) δ 179.43, 44.46, 38.12, 35.91, 35.87, 32.77, 30.64, 22.58, 16.48, 15.11; **HRMS (ESI)**: Calculated mass for $C_{12}H_{23}O_2$ $[M+H]^+$: 199.1698; observed mass: 199.1695.

2-(1-Methyl-3-(naphthalen-1-yl)cyclohexyl)acetic acid (24)

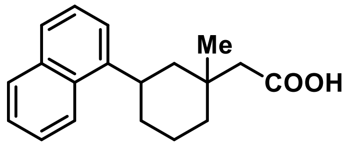

Eluent: ethyl acetate/ petroleum ether (15:85 v/v).

Appearance: white solid.

Isolated yield: 79% (449 mg, 1.58 mmol)

^{1}**H NMR** (400 MHz, $CDCl_3$) δ 8.18 (d, *J* = 8.4 Hz, 1H), 7.84 (dd, *J* = 8.0, 1.5 Hz, 1H), 7.70 (dt, *J* = 7.8, 1.1 Hz, 1H), 7.57 – 7.33 (m, 4H), 3.81 – 3.44 (m, 1H), 2.88 – 2.49 (m, 2H), 2.03 (ddt, *J* = 11.0, 5.3, 2.5 Hz, 2H), 1.80 (dddd, *J* = 23.3, 12.9, 6.9, 2.8 Hz, 3H), 1.59 (td, *J* = 12.3, 4.1 Hz, 1H), 1.43 (dd, *J* = 13.8, 12.5 Hz, 1H), 1.39 – 1.21 (m, 1H), 1.14 (s, 3H); ^{13}**C NMR** (101 MHz, $CDCl_3$) δ 178.71, 143.00, 134.13, 131.58, 129.10, 126.69, 125.94, 125.78, 125.54, 123.37, 122.51, 45.82, 41.32, 38.26, 34.80, 34.11, 33.34, 30.18, 22.86; **HRMS (ESI)**: Calculated mass for $C_{19}H_{23}O_2$ $[M+H]^+$: 283.1698; observed mass: 283.1690.

2-(3-(2-Fluorophenyl)-1-methylcyclohexyl)acetic acid (25)

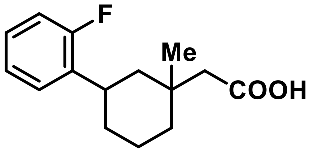

Eluent: ethyl acetate/ petroleum ether (15:85 v/v).

Appearance: white solid.

Isolated yield: 73% (365 mg, 1.46 mmol)

^{1}H NMR (400 MHz, $CDCl_3$) δ 7.22 (td, J = 7.6, 1.9 Hz, 1H), 7.15 (tdd, J = 7.3, 5.1, 1.9 Hz, 1H), 7.07 (td, J = 7.5, 1.4 Hz, 1H), 6.99 (ddd, J = 9.6, 8.1, 1.4 Hz, 1H), 3.22 – 2.98 (m, 1H), 2.61 – 2.40 (m, 2H), 1.94 – 1.56 (m, 5H), 1.47 – 1.32 (m, 2H), 1.29 – 1.15 (m, 1H), 1.12 (s, 3H); **^{13}C NMR** (101 MHz, $CDCl_3$) δ 178.88, 162.12, 159.68, 133.78, 133.63, 127.82, 127.77, 127.49, 127.41, 124.25, 124.22, 115.63, 115.40, 44.56, 41.03, 37.48, 34.35, 32.54, 32.52, 32.42, 30.11, 22.44; **^{19}F NMR** (376 MHz, $CDCl_3$) δ -119.01, -119.03, -119.04, -119.06, -119.07; **HRMS (ESI)**: Calculated mass for $C_{15}H_{20}FO_2$ $[M+H]^+$: 251.1447; observed mass: 251.1450.

2-(1-Isobutylcyclohexyl)acetic acid (26)

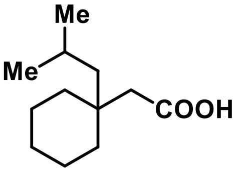

Eluent: ethyl acetate/ petroleum ether (10:90 v/v).

Appearance: colorless gummy.

Isolated yield: 79% (313 mg, 1.58 mmol)

^{1}H NMR (400 MHz, $CDCl_3$) δ 2.36 (s, 2H), 1.80 – 1.64 (m, 1H), 1.57 – 1.37 (m, 9H), 1.36 (d, J = 5.2 Hz, 3H), 0.93 (d, J = 6.7 Hz, 6H); **^{13}C NMR** (101 MHz, $CDCl_3$) δ 179.50, 46.94, 42.13, 36.95, 36.32, 29.92, 26.30, 25.75, 25.69, 23.78, 22.00; **HRMS (ESI)**: Calculated mass for $C_{12}H_{22}NaO_2$ $[M+Na]^+$: 221.1517; observed mass: 221.1520.

2-(1-Cyclopentylcyclohexyl)acetic acid (27)

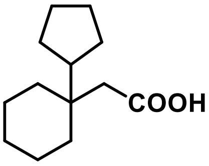

Eluent: ethyl acetate/ petroleum ether (10:90 v/v).

Appearance: colorless gummy.

Isolated yield: 76% (320 mg, 1.52 mmol)

^{1}H NMR (400 MHz, $CDCl_3$) δ 2.38 (s, 2H), 2.23 – 2.00 (m, 1H), 1.75 – 1.43 (m, 12H), 1.41 – 1.19 (m, 6H); **^{13}C NMR** (101 MHz, $CDCl_3$) δ 180.32, 46.73, 39.50, 39.46, 38.36, 32.70, 26.19, 25.93, 21.82; **HRMS (ESI)**: Calculated mass for $C_{13}H_{23}O_2$ $[M+H]^+$: 211.1698; observed mass: 211.1695.

2-(1,3-Dimethylcyclopentyl)acetic acid (28)

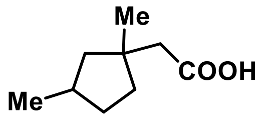

Eluent: ethyl acetate/ petroleum ether (10:90 v/v).

Appearance: colorless liquid.

Isolated yield: 71% (111 mg, 0.71 mmol)

^{1}H NMR (400 MHz, $CDCl_3$) δ 2.33 (dd, J = 17.0, 3.6 Hz, 2H), 2.06 (dq, J = 16.9, 8.2 Hz, 1H), 1.96 – 1.40 (m, 4H), 1.34 – 1.16 (m, 2H), 1.11 (dd, J = 24.4, 3.6 Hz, 3H), 0.98 (dt, J = 6.4, 2.9 Hz, 3H); **^{13}C NMR** (101 MHz, $CDCl_3$) δ 179.19, 48.93, 48.43, 47.42, 46.22, 41.66, 41.53, 40.02, 39.43, 34.05, 33.94, 33.65, 33.32, 28.17, 27.04, 21.31, 21.15; **HRMS (ESI)**: Calculated mass for $C_9H_{17}O_2$ $[M+H]^+$: 157.1229; observed mass: 157.1232.

2-([1,1'-Bi(cyclohexan)]-1-yl)acetic acid (29)

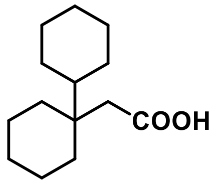

Eluent: ethyl acetate/ petroleum ether (10:90 v/v).

Appearance: white solid.

Isolated yield: 66% (148 mg, 0.66 mmol)

^{1}H NMR (400 MHz, $CDCl_3$) δ 2.34 (s, 2H), 1.87 – 1.72 (m, 4H), 1.65 (dt, J = 13.8, 3.1 Hz, 1H), 1.60 – 1.40 (m, 9H), 1.38 – 1.05 (m, 4H), 1.05 – 0.91 (m, 2H), 0.92 – 0.83 (m, 1H); **^{13}C NMR** (101 MHz, $CDCl_3$) δ 180.23, 43.26, 38.98, 38.84, 32.59, 31.80, 27.53, 26.97, 26.73, 26.30, 22.86, 21.73, 14.31; **HRMS (ESI)**: Calculated mass for $C_{14}H_{25}O_2$ $[M+H]^+$: 225.1855; observed mass: 225.1860.

2-(1-Methyl-4-propylcyclohexyl)acetic acid (30)

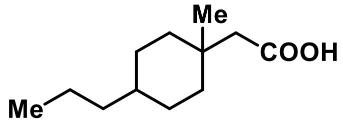

Eluent: ethyl acetate/ petroleum ether (10:90 v/v).

Appearance: colorless oil.

Isolated yield: 82% (162 mg, 0.82 mmol)

^{1}H NMR (400 MHz, $CDCl_3$) δ 2.32 (s, 2H), 1.70 – 1.50 (m, 4H), 1.35 – 1.24 (m, 3H), 1.23 – 1.14 (m, 5H), 1.13 – 1.07 (m, 1H), 1.04 (s, 3H), 0.87 (t, J = 7.2 Hz, 3H); **^{13}C NMR** (101 MHz, $CDCl_3$) δ 179.70, 41.22, 39.42, 37.89, 37.05, 33.45, 29.47, 28.77, 20.24, 14.57; **HRMS (ESI)**: Calculated mass for $C_{12}H_{23}O_2$ $[M+H]^+$: 199.1698; observed mass: 199.1690.

2-(1-Methyl-3-(*p*-tolyl)cyclohexyl)acetic acid (31)

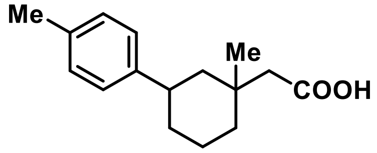

Eluent: ethyl acetate/ petroleum ether (15:85 v/v).

Appearance: white solid.

Isolated yield: 77% (190 mg, 0.77 mmol)

^{1}H NMR (400 MHz, $CDCl_3$) δ 7.11 (s, 4H), 2.73 (tt, J = 12.6, 3.4 Hz, 1H), 2.53 (d, J = 13.2 Hz, 1H), 2.44 (d, J = 13.2 Hz, 1H), 2.33 (s, 3H), 1.96 – 1.79 (m, 2H), 1.78 – 1.71 (m, 1H), 1.67 – 1.54 (m, 1H), 1.46 – 1.17 (m, 4H), 1.12 (s, 3H); **^{13}C NMR** (101 MHz, $CDCl_3$) δ 179.12, 144.30, 135.63, 129.26, 126.92, 46.24, 41.23, 39.16, 37.79, 34.44, 33.70, 30.15, 22.50, 21.17; **HRMS (ESI)**: Calculated mass for $C_{16}H_{23}O_2$ $[M+H]^+$: 247.1698; observed mass: 247.1690.

2-(1-Isopropylcycloheptyl)acetic acid (32)

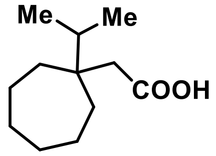

Eluent: ethyl acetate/ petroleum ether (10:90 v/v).

Appearance: colorless gummy.

Isolated yield: 69% (137 mg, 0.69 mmol)

^{1}H NMR (500 MHz, $CDCl_3$) δ 2.24 (s, 2H), 1.84 (p, J = 6.9 Hz, 1H), 1.61 (dd, J = 6.2, 3.7 Hz, 4H), 1.53 – 1.46 (m, 8H), 0.88 (d, J = 6.9 Hz, 6H); **^{13}C NMR** (126 MHz, $CDCl_3$) δ 180.11, 42.06, 41.70, 36.00, 35.32, 30.87, 23.85, 17.70; **HRMS (ESI)**: Calculated mass for $C_{12}H_{22}NaO_2$ $[M+H]^+$: 221.1517; observed mass: 221.1520.

2-(1-Cyclohexylcyclopentyl)acetic acid (33)

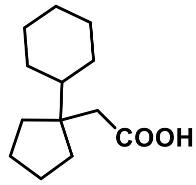

Eluent: ethyl acetate/ petroleum ether (10:90 v/v).

Appearance: colorless gummy.

Isolated yield: 71% (149 mg, 0.71 mmol)

^{1}H NMR (400 MHz, $CDCl_3$) δ 2.31 (s, 2H), 1.75 (dq, *J* = 9.7, 2.8, 2.3 Hz, 4H), 1.69 – 1.51 (m, 9H), 1.40 – 1.28 (m, 1H), 1.27 – 1.07 (m, 3H), 1.00 (qd, *J* = 12.6, 11.8, 3.5 Hz, 2H); **^{13}C NMR** (101 MHz, $CDCl_3$) δ 180.34, 48.31, 46.60, 41.44, 41.38, 41.32, 35.44, 28.52, 27.29, 26.91, 25.48; **HRMS (ESI)**: Calculated mass for $C_{13}H_{23}O_2$ $[M+H]^+$: 211.1698; observed mass: 211.1690.

2-(1-Methylcyclopentadecyl)acetic acid (34)

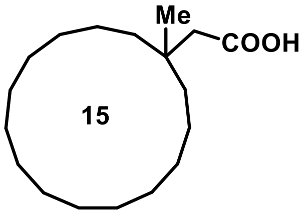

Eluent: ethyl acetate/ petroleum ether (15:85 v/v).

Appearance: white solid.

Isolated yield: 43% (121 mg, 0.43 mmol)

^{1}H NMR (400 MHz, $CDCl_3$) δ 2.22 (s, 2H), 1.60 – 1.21 (m, 28H), 1.02 (s, 3H); **^{13}C NMR** (101 MHz, $CDCl_3$) δ 178.59, 45.50, 38.28, 36.01, 29.92, 28.13, 27.23, 27.13, 26.93, 26.37, 25.88, 21.88; **HRMS (ESI)**: Calculated mass for $C_{18}H_{35}O_2$ $[M+H]^+$: 283.2637; observed mass: 283.2640.

2-(1-Ethyl-3-methylcyclopentyl)acetic acid (35)

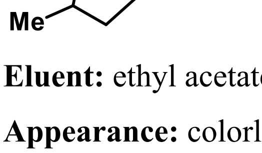

Eluent: ethyl acetate/ petroleum ether (10:90 v/v).

Appearance: colorless liquid.

Isolated yield: 68% (115 mg, 0.68 mmol)

^{1}**H NMR** (500 MHz, $CDCl_3$) δ 2.47 – 2.13 (m, 2H), 2.15 – 1.93 (m, 1H), 1.79 (dtd, *J* = 25.6, 13.1, 7.5 Hz, 2H), 1.69 – 1.44 (m, 4H), 0.98 (d, *J* = 6.7 Hz, 3H), 0.87 (dtd, *J* = 14.7, 7.1, 2.7 Hz, 5H); ^{13}**C NMR** (126 MHz, $CDCl_3$) δ 178.75, 46.59, 46.53, 45.48, 45.02, 43.66, 42.54, 37.52, 37.46, 34.89, 34.05, 33.92, 33.81, 33.12, 32.15, 31.91, 31.81, 29.93, 29.88, 29.59, 27.14, 22.92, 22.88, 22.84, 20.96, 20.95, 14.34, 11.65, 9.49, 9.23; **HRMS (ESI)**: Calculated mass for $C_{10}H_{19}O_2$ $[M+H]^+$: 171.1385; observed mass: 171.1390.

2-(1-Cyclohexylcycloheptyl)acetic acid (36)

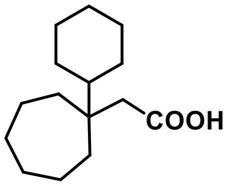

Eluent: ethyl acetate/ petroleum ether (15:85 v/v).
Appearance: white solid.
Isolated yield: 71% (170 mg, 0.71 mmol)
^{1}**H NMR** (400 MHz, $CDCl_3$) δ 2.26 (s, 2H), 1.86 – 1.73 (m, 4H), 1.70 – 1.35 (m, 14H), 1.32 – 1.06 (m, 3H), 0.98 (qd, *J* = 12.4, 11.8, 3.3 Hz, 2H); ^{13}**C NMR** (101 MHz, $CDCl_3$) δ 179.85, 46.28, 42.39, 42.15, 36.27, 31.01, 27.63, 27.50, 26.99, 23.93; **HRMS (ESI)**: Calculated mass for $C_{15}H_{27}O_2$ $[M+H]^+$: 239.2011; observed mass: 239.2015.

2-(1-Octylcyclohexyl)acetic acid (37)

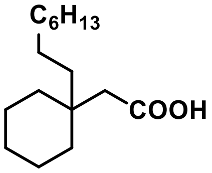

Eluent: ethyl acetate/ petroleum ether (10:90 v/v).
Appearance: colorless gummy.
Isolated yield: 72% (183 mg, 0.72 mmol)
^{1}**H NMR** (400 MHz, $CDCl_3$) δ 9.66 (s, 1H), 2.29 (s, 2H), 1.51 – 1.33 (m, 12H), 1.26 (s, 12H), 0.88 (t, *J* = 6.7 Hz, 3H); ^{13}**C NMR** (101 MHz, $CDCl_3$) δ 179.48, 41.98, 37.84, 36.13, 36.02, 32.14, 30.66, 29.82, 29.56, 26.35, 23.14, 22.91, 21.87, 14.34; **HRMS (ESI)**: Calculated mass for $C_{16}H_{31}O_2$ $[M+H]^+$: 255.2324; observed mass: 255.2330.

2-(1-(3-Methoxyphenyl)cyclohexyl)acetic acid (38)

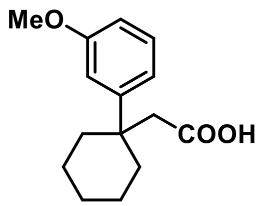

Eluent: ethyl acetate/ petroleum ether (20:80 v/v).

Appearance: white solid.

Isolated yield: 71% (177 mg, 0.71 mmol)

^{1}H NMR (400 MHz, $CDCl_3$) δ 7.29 (d, J = 8.1 Hz, 1H), 6.99 (ddd, J = 7.8, 1.9, 0.9 Hz, 1H), 6.95 (t, J = 2.2 Hz, 1H), 6.78 (ddd, J = 8.2, 2.5, 0.9 Hz, 1H), 3.83 (s, 3H), 2.56 (s, 2H), 2.29 – 2.15 (m, 2H), 1.81 (ddd, J = 12.9, 8.8, 3.5 Hz, 2H), 1.59 (qd, J = 8.8, 4.4 Hz, 2H), 1.47 (ddd, J = 13.5, 9.1, 6.3 Hz, 4H); **^{13}C NMR** (101 MHz, $CDCl_3$) δ 177.01, 159.80, 147.10, 129.40, 119.29, 113.56, 110.77, 55.34, 48.04, 40.94, 36.14, 26.30, 22.51; **HRMS (ESI)**: Calculated mass for $C_{15}H_{20}NaO_3$ $[M+Na]^+$: 271.1310; observed mass: 271.1315.

2-(1-Ethylcyclododecyl)acetic acid (39)

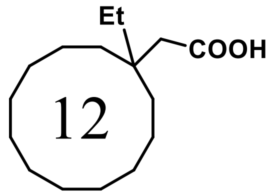

Eluent: ethyl acetate/ petroleum ether (10:90 v/v).

Appearance: colorless gummy.

Isolated yield: 52% (133 mg, 0.52 mmol)

^{1}H NMR (400 MHz, $CDCl_3$) δ 2.19 (s, 2H), 1.41 – 1.16 (m, 24H), 0.86 (t, J = 7.4 Hz, 3H); **^{13}C NMR** (101 MHz, $CDCl_3$) δ 178.42, 40.34, 39.02, 32.63, 29.16, 26.95, 26.39, 22.88, 22.28, 18.81, 8.01; **HRMS (ESI)**: Calculated mass for $C_{16}H_{31}O_2$ $[M+H]^+$: 255.2324; observed mass: 255.2322.

2-(1-Isobutylcycloheptyl)acetic acid (40)

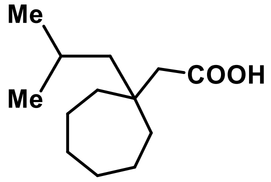

Eluent: ethyl acetate/ petroleum ether (15:85 v/v).

Appearance: colorless gummy.

Isolated yield: 62% (132 mg, 0.62 mmol)

^{1}**H NMR** (400 MHz, $CDCl_3$) δ 2.29 (s, 2H), 1.78 – 1.65 (m, 1H), 1.61 (ddd, *J* = 14.3, 7.5, 2.9 Hz, 2H), 1.56 – 1.39 (m, 10H), 1.36 (d, *J* = 5.2 Hz, 2H), 0.93 (d, *J* = 6.7 Hz, 6H); ^{13}**C NMR** (101 MHz, $CDCl_3$) δ 179.55, 48.82, 44.24, 40.10, 39.13, 30.93, 25.76, 24.03, 22.98; **HRMS (ESI)**: Calculated mass for $C_{13}H_{25}O_2$ $[M+H]^+$: 213.1855; observed mass: 213.1860.

2-(1-Methyl-4-(trifluoromethyl)cyclohexyl)acetic acid (41)

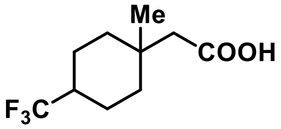

Eluent: ethyl acetate/ petroleum ether (15:85 v/v).

Appearance: white solid.

Isolated yield: 70% (157 mg, 0.59 mmol)

^{1}**H NMR** (400 MHz, $CDCl_3$) δ 2.36 (s, 2H), 1.98 (ddt, *J* = 16.5, 8.5, 4.0 Hz, 1H), 1.85 – 1.70 (m, 4H), 1.60 – 1.38 (m, 2H), 1.21 (td, *J* = 14.6, 13.9, 4.1 Hz, 2H), 1.08 (s, 3H); ^{13}**C NMR** (101 MHz, $CDCl_3$) δ 178.92, 129.36, 126.59, 41.86, 41.59, 40.47, 36.36, 32.74, 29.24, 20.82, 20.80, 20.77, 20.75; ^{19}**F NMR** (376 MHz, $CDCl_3$) δ -73.60; **HRMS (ESI)**: Calculated mass for $C_{10}H_{16}F_3O_2$ $[M+H]^+$: 225.1102; observed mass: 225.1105.

2-(1-(3,4-Dimethoxyphenyl)cyclohexyl)acetic acid (42)

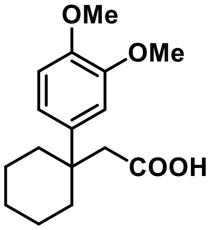

Eluent: ethyl acetate/ petroleum ether (30:70 v/v).

Appearance: white solid.

Isolated yield: 72% (200 mg, 0.72 mmol)

^{1}**H NMR** (500 MHz, $CDCl_3$) δ 6.88 (d, *J* = 8.3 Hz, 2H), 6.80 (d, *J* = 8.2 Hz, 1H), 3.84 (s, 3H), 3.83 (s, 3H), 2.49 (s, 2H), 2.21 – 2.10 (m, 2H), 1.76 (ddd, *J* = 13.0, 9.1, 3.4 Hz, 2H), 1.60 – 1.50 (m, 2H), 1.42 (ddd, *J* = 14.5, 10.6, 7.5 Hz, 4H); ^{13}**C NMR** (126 MHz, $CDCl_3$) δ 177.75, 148.69, 147.21, 137.72, 119.04, 111.02, 110.46, 56.00, 55.83, 48.22, 40.50, 36.28, 26.24, 22.40; **HRMS (ESI)**: Calculated mass for $C_{16}H_{23}O_4$ $[M+H]^+$: 279.1596; observed mass: 279.1600.

2-(1-Methyl-4-(*p*-tolyl)cycloheptyl)acetic acid (43)

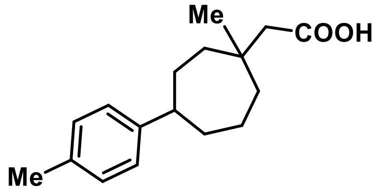

Eluent: ethyl acetate/ petroleum ether (15:85 v/v).

Appearance: white solid.

Isolated yield: 69% (180 mg, 0.69 mmol)

^{1}H NMR (400 MHz, $CDCl_3$) δ 7.14 – 7.02 (m, 4H), 2.66 (ddt, J = 11.3, 8.9, 2.4 Hz, 1H), 2.42 (d, J = 13.2 Hz, 1H), 2.32 (s, 3H), 2.30 – 2.21 (m, 1H), 1.97 – 1.83 (m, 2H), 1.83 – 1.72 (m, 2H), 1.65 (dtdd, J = 18.2, 14.2, 7.1, 4.7 Hz, 4H), 1.54 – 1.42 (m, 2H), 1.09 (s, 3H); **^{13}C NMR** (101 MHz, $CDCl_3$) δ 178.92, 147.19, 135.19, 129.29, 126.46, 49.91, 49.15, 47.98, 46.14, 40.78, 40.27, 40.23, 39.41, 39.30, 36.42, 30.52, 30.39, 29.92, 28.98, 27.14, 22.85, 22.75, 21.16, 14.34; **HRMS (ESI)**: Calculated mass for $C_{17}H_{25}O_2$ $[M+H]^+$: 261.1855; observed mass: 261.1860.

2-(1-Ethylcyclopentadecyl)acetic acid (44)

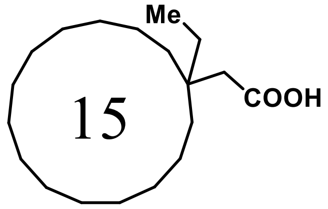

Eluent: ethyl acetate/ petroleum ether (12:88 v/v).

Appearance: white solid.

Isolated yield: 47% (140 mg, 0.47 mmol)

^{1}H NMR (400 MHz, $CDCl_3$) δ 2.21 (s, 2H), 1.49 – 1.15 (m, 30H), 0.85 (t, J = 7.4 Hz, 3H); **^{13}C NMR** (101 MHz, $CDCl_3$) δ 178.93, 40.90, 38.91, 36.09, 29.93, 29.58, 28.16, 27.21, 27.16, 26.90, 26.31, 21.42, 8.02; **HRMS (ESI)**: Calculated mass for $C_{19}H_{37}O_2$ $[M+H]^+$: 297.2794; observed mass: 297.2790.

2-(1-Isobutyl-3-methylcyclohexyl)acetic acid (45)

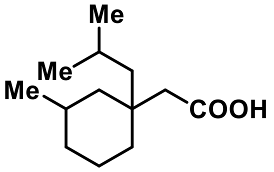

Eluent: ethyl acetate/ petroleum ether (12:88 v/v).

Appearance: colorless liquid.

Isolated yield: 61% (129 mg, 0.61 mmol)

^{1}**H NMR** (400 MHz, $CDCl_3$) δ 2.42 (s, 2H), 1.83 – 1.37 (m, 7H), 1.30 (d, *J* = 5.2 Hz, 2H), 1.12 (td, *J* = 13.3, 4.2 Hz, 1H), 0.93 (d, *J* = 6.6 Hz, 6H), 0.85 (d, *J* = 6.3 Hz, 3H), 0.83 – 0.69 (m, 2H); ^{13}**C NMR** (101 MHz, $CDCl_3$) δ 179.28, 51.11, 45.31, 39.17, 37.81, 35.82, 35.18, 27.88, 25.87, 25.84, 23.55, 23.25, 22.11; **HRMS (ESI)**: Calculated mass for $C_{13}H_{25}O_2$ $[M+H]^+$: 213.1855; observed mass: 213.1860.

2-(1,3-Dimethylcyclopentadecyl)acetic acid (46)

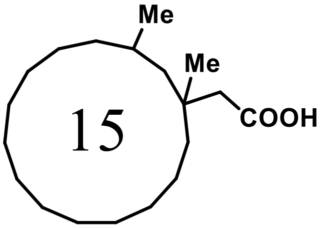

Eluent: ethyl acetate/ petroleum ether (12:88 v/v).

Appearance: colorless liquid.

Isolated yield: 71% (210 mg, 0.71 mmol)

^{1}**H NMR** (400 MHz, $CDCl_3$) δ 2.30 (s, 1H), 2.25 – 2.13 (m, 1H), 1.47 – 1.26 (m, 21H), 1.03 (d, *J* = 14.2 Hz, 3H), 0.88 (ddt, *J* = 13.6, 10.2, 5.1 Hz, 9H); ^{13}**C NMR** (101 MHz, $CDCl_3$) δ 178.87, 178.66, 46.94, 46.10, 44.98, 38.70, 38.29, 37.99, 37.75, 36.85, 36.65, 32.15, 29.93, 29.89, 29.59, 28.13, 27.83, 27.78, 27.71, 27.48, 27.45, 27.24, 27.14, 27.07, 27.04, 26.66, 26.34, 26.24, 26.15, 25.95, 25.89, 25.80, 25.73, 25.58, 25.39, 25.07, 22.92, 22.87, 22.48, 22.31, 22.22, 14.35; **HRMS (ESI)**: Calculated mass for $C_{19}H_{37}O_2$ $[M+H]^+$: 297.2794; observed mass: 297.2791.

2-(1-Isopropylcyclopentyl)acetic acid (47)

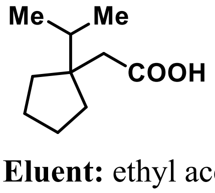

Eluent: ethyl acetate/ petroleum ether (10:90 v/v).

Appearance: colorless liquid.

Isolated yield: 79% (135 mg, 0.79 mmol)

^{1}**H NMR** (400 MHz, $CDCl_3$) δ 2.32 (s, 2H), 1.79 (p, *J* = 6.8 Hz, 1H), 1.66 – 1.52 (m, 8H), 0.89 (d, *J* = 6.8 Hz, 6H); ^{13}**C NMR** (101 MHz, $CDCl_3$) δ 179.87, 48.45, 41.33, 35.29, 35.08, 25.69, 18.45; **HRMS (ESI)**: Calculated mass for $C_{10}H_{19}O_2$ $[M+H]^+$: 171.1385; observed mass: 171.1382.

6. General Procedure for Dehydrogenative Lactonization:

In an oven-dried screw capped reaction tube was charged with magnetic stir-bar, corresponding acid (0.2 mmol), $Pd(OAc)_2$ (10 mol%), *N*-Ac-^{t}Leu (20 mol%), Ag_2CO_3 (2 equiv.), and Na_3PO_4 (2 equiv.) in 1.5 mL of 1,1,1,3,3,3-hexafluoro-2-propanol (HFIP) were added. The reaction tube was capped and placed in a preheated bath at 120 °C with stirring (800 rpm) for 24 h. Upon completion the mixture was diluted with EtOAc and filtered through a celite pad. The filtrate was evaporated under reduced pressure and the crude mixture was purified by column chromatography using silica (100-200 mesh size) and petroleum ether/ ethyl acetate as the eluent.

General Procedure for Intermolecular Dehydrogenative Lactonization with Olefins:

In an oven-dried screw capped reaction tube was charged with magnetic stir-bar, corresponding acid (0.2 mmol), $Pd(OAc)_2$ (10 mol%), *N*-Ac-^{t}Leu (20 mol%), Ag_2CO_3 (2 equiv.), Na_2HPO_4 (2 equiv.) and olefin or allyl alcohol (2 equiv.) in 2 mL of 1,1,1,3,3,3-hexafluoro-2-propanol (HFIP) were added. The reaction tube was capped and placed in a heating bath at 110 °C with stirring (800 rpm) for 24 h. Upon completion the mixture was diluted with EtOAc and filtered through a celite pad. The filtrate was evaporated under reduced pressure and the crude mixture was purified by column chromatography using silica (100-200 mesh size) and petroleum ether/ ethyl acetate as the eluent.

7. Analysis of the Crude Reaction Mixture

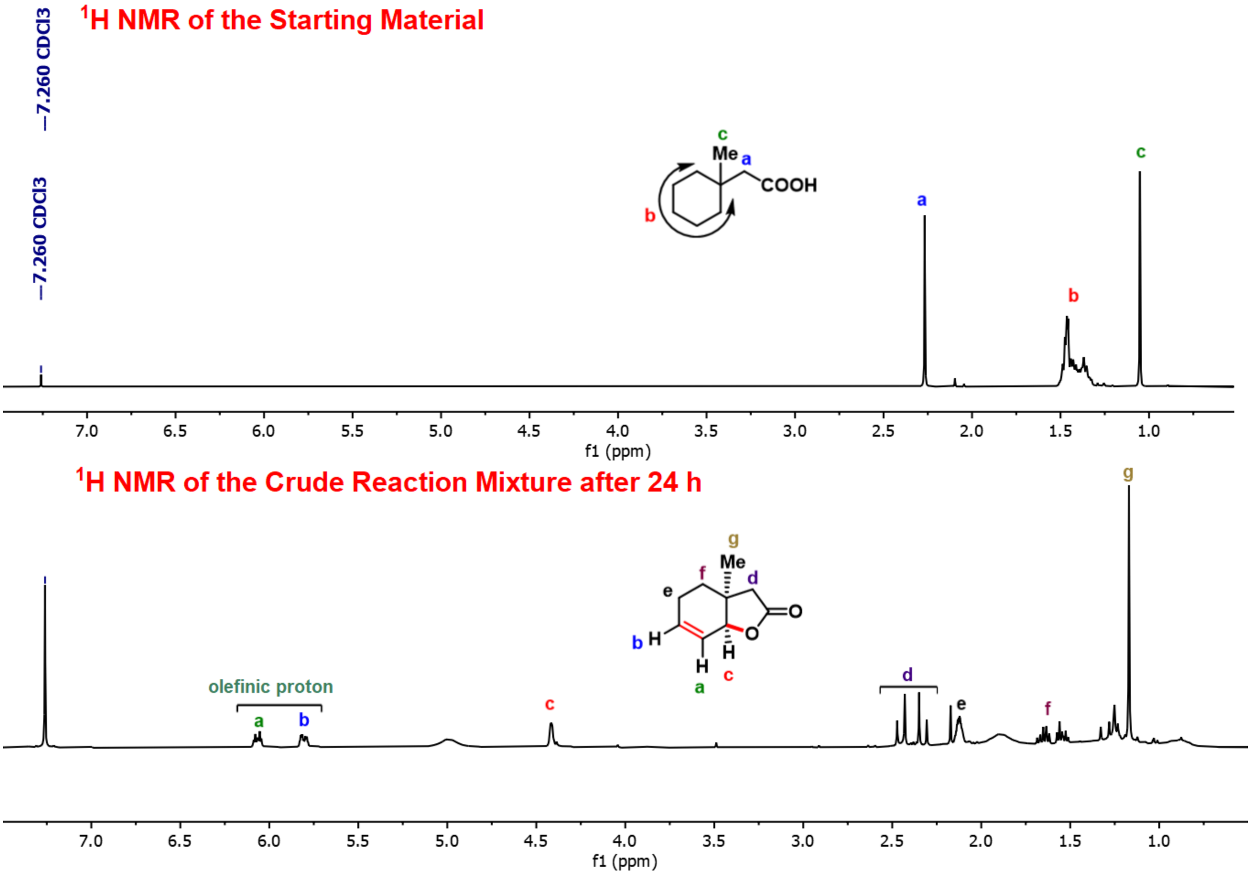

Figure S1: Comparison of ^{1}H NMR of starting material and reaction mixture after 24 h.

8. Spectral Data of the Unsaturated Bicyclic Lactones:

(3a*S*,7a*S*)-3a-Methyl-3a,4,5,7a-tetrahydrobenzofuran-2(3*H*)-one (2a)

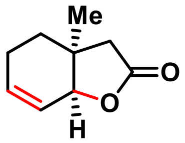

Eluent: ethyl acetate/ petroleum ether (6:94 v/v).

Appearance: colorless liquid.

Isolated yield: 70% (21.5 mg, 0.14 mmol)

^{1}H NMR (400 MHz, $CDCl_3$) δ 6.13 – 5.95 (m, 1H), 5.78 (dq, *J* = 10.2, 1.9 Hz, 1H), 4.44 – 4.28 (m, 1H), 2.51 – 2.37 (m, 1H), 2.34 – 2.24 (m, 1H), 2.17 – 2.03 (m, 2H), 1.69 – 1.58 (m, 1H), 1.52 (dt, *J* = 13.7, 5.6 Hz, 1H), 1.15 (s, 3H); **^{13}C NMR** (101 MHz, $CDCl_3$) δ 176.45, 133.22, 123.03, 81.24, 42.13, 37.26, 29.53, 23.50, 21.85; **HRMS (ESI)**: Calculated mass for $C_9H_{13}O_2$ $[M+H]^+$: 153.0916; observed mass: 153.0920.

(3a*S*,7a*S*)-3a-Ethyl-3a,4,5,7a-tetrahydrobenzofuran-2(3H)-one (2b)

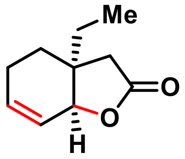

Eluent: ethyl acetate/ petroleum ether (6:94 v/v).

Appearance: colorless gummy.

Isolated yield: 74% (24.5 mg, 0.15 mmol)

^{1}H NMR (400 MHz, $CDCl_3$) δ 6.23 – 5.92 (m, 1H), 5.81 (ddt, *J* = 10.0, 4.1, 2.1 Hz, 1H), 4.42 (dd, *J* = 4.2, 1.6 Hz, 1H), 2.37 (s, 2H), 2.08 (dtt, *J* = 7.3, 3.5, 1.9 Hz, 2H), 1.61 – 1.50 (m, 3H), 1.41 (dq, *J* = 14.7, 7.5 Hz, 1H), 0.93 (t, *J* = 7.5 Hz, 3H); **^{13}C NMR** (101 MHz, $CDCl_3$) δ 176.62, 133.45, 123.22, 80.54, 80.52, 40.89, 39.77, 39.74, 28.70, 26.15, 21.63, 8.93; **HRMS (ESI)**: Calculated mass for $C_{10}H_{15}O_2$ $[M+H]^+$: 167.1072; observed mass: 167.1077.

(3a*S*,7a*S*)-3a-Isopropyl-3a,4,5,7a-tetrahydrobenzofuran-2(3H)-one (2c)

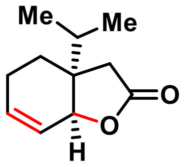

Eluent: ethyl acetate/ petroleum ether (6:96 v/v).

Appearance: colorless liquid.

Isolated yield: 75% (27 mg, 0.15 mmol)

^{1}H NMR (400 MHz, $CDCl_3$) δ 6.05 (dtd, *J* = 10.0, 3.8, 1.0 Hz, 1H), 5.79 (ddt, *J* = 10.1, 4.2, 2.2 Hz, 1H), 4.68 (dq, *J* = 4.1, 1.4 Hz, 1H), 2.41 (d, *J* = 17.4 Hz, 1H), 2.27 (d, *J* = 17.5 Hz, 1H), 2.18 – 2.03 (m, 2H), 1.90 – 1.71 (m, 1H), 1.71 – 1.55 (m, 2H), 0.94 (dd, *J* = 8.5, 6.8 Hz, 6H); **^{13}C NMR** (101 MHz, $CDCl_3$) δ 176.86, 133.16, 124.07, 78.58, 43.48, 35.61, 31.70, 25.80, 21.62, 18.16, 18.13, 17.78, 17.76; **HRMS (ESI)**: Calculated mass for $C_{11}H_{17}O_2$ $[M+H]^+$: 181.1229; observed mass: 181.1235.

(3a*S*,7a*S*)-3a-Propyl-3a,4,5,7a-tetrahydrobenzofuran-2(3H)-one (2d)

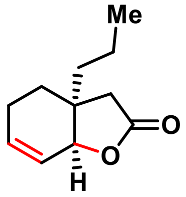

Eluent: ethyl acetate/ petroleum ether (6:94 v/v).

Appearance: colorless liquid.

Isolated yield: 82% (29.5 mg, 0.16 mmol)

^{1}H NMR (400 MHz, $CDCl_3$) δ 6.28 – 5.97 (m, 1H), 5.81 (ddt, *J* = 10.1, 4.2, 2.1 Hz, 1H), 4.40 (dd, *J* = 4.1, 1.4 Hz, 1H), 2.38 (s, 2H), 2.19 – 1.98 (m, 2H), 1.59 – 1.46 (m, 2H), 1.43 – 1.21 (m, 4H), 0.94 (t, *J* = 7.0 Hz, 3H); **^{13}C NMR** (126 MHz, $CDCl_3$) δ 176.65, 133.56, 123.11, 80.67, 40.66, 40.44, 38.42, 26.66, 21.70, 17.99, 14.83; **HRMS (ESI)**: Calculated mass for $C_{11}H_{16}NaO_2$ $[M+Na]^+$: 203.1048; observed mass: 203.1055.

(3a*S*,7a*S*)-3a-Butyl-3a,4,5,7a-tetrahydrobenzofuran-2(3H)-one (2e)

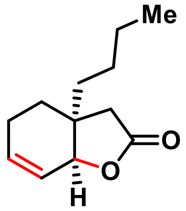

Eluent: ethyl acetate/ petroleum ether (6:94 v/v).

Appearance: colorless liquid.

Isolated yield: 68% (26.5 mg, 0.136 mmol) (dr = 5:1)

^{1}H NMR (400 MHz, $CDCl_3$) δ 6.14 – 6.00 (m, 1H), 5.84 – 5.74 (m, 1H), 4.40 (dd, *J* = 4.0, 1.4 Hz, 1H), 2.38 (s, 2H), 2.08 (ddq, *J* = 7.5, 3.7, 1.8 Hz, 2H), 1.71 – 1.51 (m, 4H), 1.38 – 1.29 (m, 4H), 0.92 (t, *J* = 7.0 Hz, 3H); **^{13}C NMR** (101 MHz, $CDCl_3$) δ 176.63, 133.55, 123.17, 80.72,

40.61, 40.44, 35.84, 29.91, 26.86, 26.69, 23.43, 21.71, 14.18; **HRMS (ESI)**: Calculated mass for $C_{12}H_{19}O_2$ $[M+H]^+$: 195.1385; observed mass: 195.1390.

(3a*R*,7a*S*)-3a-Isobutyl-3a,4,5,7a-tetrahydrobenzofuran-2(3H)-one (2f)

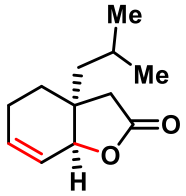

Eluent: ethyl acetate/ petroleum ether (7:93 v/v).

Appearance: colorless gummy.

Isolated yield: 79% (30.5 mg, 0.16 mmol)

^{1}H NMR (400 MHz, $CDCl_3$) δ 6.19 – 6.01 (m, 1H), 5.83 (ddt, *J* = 10.1, 4.3, 2.1 Hz, 1H), 4.29 (d, *J* = 4.4 Hz, 1H), 2.50 (d, *J* = 17.1 Hz, 1H), 2.42 (d, *J* = 17.1 Hz, 1H), 2.17 – 1.99 (m, 2H), 1.79 – 1.64 (m, 2H), 1.62 – 1.48 (m, 2H), 1.11 (ddd, *J* = 14.3, 8.8, 1.1 Hz, 1H), 0.93 (dd, *J* = 11.7, 6.6 Hz, 6H); **^{13}C NMR** (101 MHz, $CDCl_3$) δ 176.66, 134.11, 122.31, 80.83, 80.80, 43.81, 42.89, 40.50, 26.30, 25.16, 25.02, 23.55, 21.91; **HRMS (ESI)**: Calculated mass for $C_{12}H_{19}O_2$ $[M+H]^+$: 195.1385; observed mass: 195.1382.

(3a*S*,7a*S*)-3a-Octyl-3a,4,5,7a-tetrahydrobenzofuran-2(3H)-one (2g)

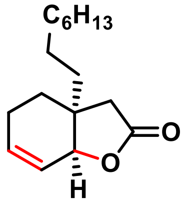

Eluent: ethyl acetate/ petroleum ether (8:92 v/v).

Appearance: colorless gummy.

Isolated yield: 78% (39 mg, 0.156 mmol) (d.r. = 4:1)

^{1}H NMR (500 MHz, $CDCl_3$) δ 6.06 (dt, *J* = 10.1, 3.8 Hz, 1H), 5.85 – 5.76 (m, 1H), 4.42 (dd, *J* = 33.3, 6.0 Hz, 1H), 2.64 – 2.21 (m, 2H), 2.07 (ddd, *J* = 8.1, 4.9, 2.8 Hz, 2H), 1.66 – 1.37 (m, 6H), 1.28 (tdq, *J* = 13.6, 9.7, 5.8, 4.1 Hz, 10H), 0.98 – 0.79 (m, 3H); **^{13}C NMR** (126 MHz, $CDCl_3$) δ 176.60, 136.92, 133.53, 123.60, 123.12, 80.67, 43.96, 40.60, 40.41, 39.86, 36.06, 35.25, 32.45, 32.01, 31.47, 30.85, 30.31, 29.88, 29.63, 29.41, 28.72, 26.63, 25.93, 24.65, 23.27, 22.82, 22.62, 22.52, 21.68, 14.27, 14.22; **HRMS (ESI)**: Calculated mass for $C_{16}H_{27}O_2$ $[M+H]^+$: 251.2011; observed mass: 251.2004.

(3a*S*,7a*S*)-3-Methyl-3a-propyl-3a,4,5,7a-tetrahydrobenzofuran-2(3H)-one (2h)

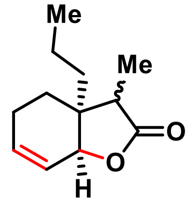

Eluent: ethyl acetate/ petroleum ether (6:94 v/v).

Appearance: colorless gummy.

Isolated yield: 65% (25.3 mg, 0.13 mmol) (d.r. = 1.5:1)

^{1}H NMR (400 MHz, $CDCl_3$) δ 6.11 (ddd, J = 10.2, 5.0, 2.4 Hz, 1H), 5.97 (dtd, J = 10.2, 3.7, 1.1 Hz, 1H), 5.87 (dddd, J = 10.0, 4.5, 2.6, 1.6 Hz, 1H), 5.74 (ddt, J = 10.1, 3.9, 2.2 Hz, 1H), 4.58 (dd, J = 3.3, 1.6 Hz, 1H), 4.25 (d, J = 4.8 Hz, 1H), 2.66 (q, J = 7.2 Hz, 1H), 2.53 (q, J = 7.4 Hz, 1H), 2.09 (dddt, J = 7.2, 5.6, 3.8, 2.0 Hz, 2H), 1.69 (dt, J = 14.2, 7.1 Hz, 1H), 1.53 (dt, J = 14.2, 5.2 Hz, 1H), 1.44 – 1.26 (m, 4H), 1.14 (dd, J = 10.9, 7.3 Hz, 3H), 0.93 (td, J = 6.8, 5.4 Hz, 3H); **^{13}C NMR** (101 MHz, $CDCl_3$) δ 179.85, 134.64, 132.17, 124.16, 121.82, 78.45, 45.18, 42.81, 42.62, 40.58, 35.84, 35.66, 32.13, 29.91, 29.86, 29.57, 26.94, 22.90, 22.87, 21.70, 21.59, 17.80, 17.69, 15.05, 14.95, 14.33, 9.15, 9.07; **HRMS (ESI)**: Calculated mass for $C_{12}H_{19}O_2$ $[M+H]^+$: 195.1385; observed mass: 195.1384.

(3a*S*,7a*S*)-3a-Cyclopentyl-3a,4,5,7a-tetrahydrobenzofuran-2(3H)-one (2i)

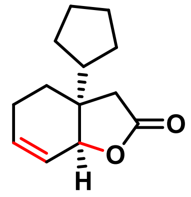

Eluent: ethyl acetate/ petroleum ether (6:94 v/v).

Appearance: colorless gummy.

Isolated yield: 80% (33 mg, 0.16 mmol) (d.r. = 5:1)

^{1}H NMR (500 MHz, $CDCl_3$) δ 6.05 (dt, J = 10.1, 3.8 Hz, 1H), 5.79 (ddt, J = 10.1, 4.2, 2.2 Hz, 1H), 4.59 (d, J = 4.0 Hz, 1H), 2.43 (d, J = 17.4 Hz, 1H), 2.29 (d, J = 17.4 Hz, 1H), 2.11 (ddt, J = 5.8, 3.8, 1.9 Hz, 2H), 1.98 (tt, J = 10.3, 7.7 Hz, 1H), 1.84 – 1.50 (m, 8H), 1.29 (ddd, J = 10.1, 7.9, 3.7 Hz, 1H), 1.16 (ddd, J = 14.2, 6.4, 2.8 Hz, 1H); **^{13}C NMR** (101 MHz, $CDCl_3$) δ 176.85, 133.28, 123.79, 79.40, 79.37, 44.01, 42.84, 36.36, 36.33, 28.26, 27.47, 27.35, 25.63, 25.43, 21.55; **HRMS (ESI)**: Calculated mass for $C_{13}H_{19}O_2$ $[M+H]^+$: 207.1385; observed mass: 207.1392.

(3a*S*,7a*S*)-3a-Cyclohexyl-3a,4,5,7a-tetrahydrobenzofuran-2(3H)-one (2j)

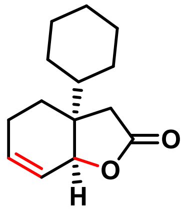

Eluent: ethyl acetate/ petroleum ether (8:92 v/v).

Appearance: colorless gummy.

Isolated yield: 82% (36 mg, 0.16 mmol) (d.r. = 6:1)

^{1}H NMR (400 MHz, $CDCl_3$) δ 6.04 (dt, *J* = 10.0, 3.8 Hz, 1H), 5.78 (ddt, *J* = 10.0, 4.1, 2.1 Hz, 1H), 4.70 (d, *J* = 4.0 Hz, 1H), 2.44 (d, *J* = 17.4 Hz, 1H), 2.27 (d, *J* = 17.4 Hz, 1H), 2.10 (td, *J* = 3.8, 1.9 Hz, 2H), 1.88 – 1.76 (m, 3H), 1.70 – 1.55 (m, 3H), 1.35 (ddd, *J* = 12.0, 9.0, 2.9 Hz, 1H), 1.28 – 1.15 (m, 3H), 1.15 – 0.88 (m, 3H); **^{13}C NMR** (101 MHz, $CDCl_3$) δ 176.95, 133.23, 124.05, 78.28, 43.28, 42.42, 36.44, 28.05, 27.79, 26.93, 26.83, 26.54, 25.83, 21.71; **HRMS (ESI)**: Calculated mass for $C_{14}H_{20}NaO_2$ $[M+Na]^+$:243.1361; observed mass: 243.1355.

(3a*S*,7a*S*)-3a-(*p*-Tolyl)-3a,4,5,7a-tetrahydrobenzofuran-2(3H)-one (2k)

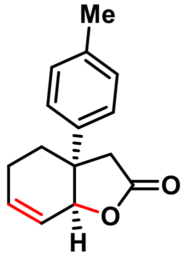

Eluent: ethyl acetate/ petroleum ether (10:90 v/v).

Appearance: white solid.

Isolated yield: 67% (30.5 mg, 0.13 mmol)

^{1}H NMR (400 MHz, $CDCl_3$) δ 7.14 (d, *J* = 8.0 Hz, 2H), 7.03 (d, *J* = 8.2 Hz, 2H), 6.25 (ddd, *J* = 10.7, 5.9, 2.1 Hz, 1H), 6.20 – 6.10 (m, 1H), 4.94 (d, *J* = 4.4 Hz, 1H), 2.99 – 2.78 (m, 2H), 2.33 (s, 3H), 2.14 – 2.00 (m, 1H), 1.93 – 1.80 (m, 1H), 1.79 – 1.65 (m, 2H); **^{13}C NMR** (101 MHz, $CDCl_3$) δ 175.36, 139.69, 137.07, 136.11, 129.70, 129.57, 125.86, 123.00, 78.36, 44.86, 44.38, 31.83, 29.92, 22.12, 21.16; **HRMS (ESI)**: Calculated mass for $C_{15}H_{16}NaO_2$ $[M+Na]^+$: 251.1048; observed mass: 251.1050.

(3a*S*,7a*S*)-3a-(4-Methoxyphenyl)-3a,4,5,7a-tetrahydrobenzofuran-2(3H)-one (2l)

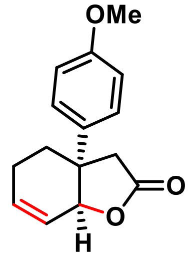

Eluent: ethyl acetate/ petroleum ether (12:88 v/v).

Appearance: white gummy.

Isolated yield: 61% (30 mg, 0.12 mmol) (d.r. = 5:1)

^{1}H NMR (400 MHz, $CDCl_3$) δ 7.06 (d, *J* = 8.8 Hz, 2H), 6.86 (d, *J* = 8.8 Hz, 2H), 6.30 – 6.20 (m, 1H), 6.19 – 6.10 (m, 1H), 4.92 (dd, *J* = 4.3, 1.3 Hz, 1H), 3.80 (s, 3H), 2.87 (d, *J* = 6.2 Hz, 2H), 2.11 – 1.98 (m, 2H), 1.87 – 1.79 (m, 1H), 1.72 (tt, *J* = 2.8, 1.5 Hz, 1H); **^{13}C NMR** (101 MHz, $CDCl_3$) δ 175.34, 158.73, 136.11, 134.73, 128.20, 127.06, 122.97, 114.33, 114.24, 78.44, 55.49, 44.56, 44.46, 31.83, 22.12; **HRMS (ESI)**: Calculated mass for $C_{15}H_{17}O_3$ $[M+H]^+$: 245.1178; observed mass: 245.1180.

(3a*S*,7a*S*)-3a-(3-Methoxyphenyl)-3a,4,5,7a-tetrahydrobenzofuran-2(3H)-one (2m)

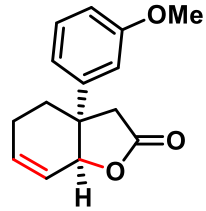

Eluent: ethyl acetate/ petroleum ether (15:85 v/v).

Appearance: white gummy.

Isolated yield: 57% (28 mg, 0.114 mmol)

^{1}H NMR (400 MHz, $CDCl_3$) δ 7.30 – 7.19 (m, 1H), 6.78 (ddd, *J* = 8.2, 2.5, 0.9 Hz, 1H), 6.70 (ddd, *J* = 7.7, 1.9, 0.9 Hz, 1H), 6.65 (t, *J* = 2.2 Hz, 1H), 6.30 – 6.18 (m, 1H), 6.11 (dq, *J* = 9.8, 2.3 Hz, 1H), 4.95 – 4.87 (m, 1H), 3.77 (s, 3H), 2.90 (d, *J* = 17.0 Hz, 1H), 2.82 (d, *J* = 17.0 Hz, 1H), 2.11 – 1.97 (m, 1H), 1.90 – 1.64 (m, 3H); **^{13}C NMR** (101 MHz, $CDCl_3$) δ 175.10, 159.99, 144.32, 136.18, 129.98, 122.96, 118.41, 112.89, 111.76, 78.18, 55.47, 45.19, 44.30, 31.82, 22.17; **HRMS (ESI)**: Calculated mass for $C_{15}H_{16}NaO_3$ $[M+Na]^+$: 267.0997; observed mass: 267.0995.

(3a*S*,7a*S*)-3a-(3,4-Dimethoxyphenyl)-3a,4,5,7a-tetrahydrobenzofuran-2(3H)-one (2n)

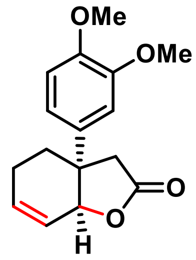

Eluent: ethyl acetate/ petroleum ether (20:80 v/v).

Appearance: white gummy.

Isolated yield: 52% (28.5 mg, 0.104 mmol)

^{1}H NMR (500 MHz, $CDCl_3$) δ 6.81 (d, *J* = 8.1 Hz, 1H), 6.71 – 6.63 (m, 2H), 6.26 (dd, *J* = 10.3, 5.5 Hz, 1H), 6.18 – 6.10 (m, 1H), 4.94 (d, *J* = 4.4 Hz, 1H), 3.87 (s, 3H), 3.85 (s, 3H), 2.92 (d, *J* = 16.8 Hz, 1H), 2.85 (d, *J* = 17.0 Hz, 1H), 2.12 – 2.02 (m, 1H), 1.88 – 1.80 (m, 1H), 1.80 – 1.72 (m, 2H); **^{13}C NMR** (126 MHz, $CDCl_3$) δ 175.22, 149.29, 148.37, 136.10, 135.25, 123.03, 118.24, 111.38, 109.64, 78.44, 56.23, 56.13, 44.85, 44.41, 31.85, 22.22; **HRMS (ESI)**: Calculated mass for $C_{16}H_{19}O_4$ $[M+H]^+$: 275.1283; observed mass: 275.1287.

(3a*R*,7a*S*)-3a,4-Dimethyl-3a,4,5,7a-tetrahydrobenzofuran-2(3H)-one (2o)

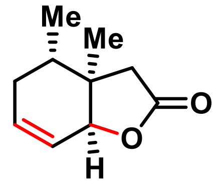

Eluent: ethyl acetate/ petroleum ether (6:94 v/v).

Appearance: colorless liquid.

Isolated yield: 85% (28 mg, 0.17 mmol)

^{1}H NMR (400 MHz, $CDCl_3$) δ 5.91 (ddt, *J* = 10.2, 5.3, 1.6 Hz, 1H), 5.66 (dtd, *J* = 10.2, 2.7, 1.3 Hz, 1H), 4.54 (td, *J* = 2.7, 1.3 Hz, 1H), 2.47 (dd, *J* = 17.0, 0.8 Hz, 1H), 2.25 – 2.06 (m, 1H), 1.98 (d, *J* = 16.9 Hz, 1H), 1.91 – 1.75 (m, 2H), 1.24 (d, *J* = 0.8 Hz, 3H), 0.96 (d, *J* = 6.5 Hz, 3H); **^{13}C NMR** (101 MHz, $CDCl_3$) δ 176.84, 131.05, 125.26, 83.87, 41.74, 34.84, 34.46, 30.41, 25.23, 16.84; **HRMS (ESI)**: Calculated mass for $C_{10}H_{15}O_2$ $[M+H]^+$: 167.1072: observed mass: 167.1075.

(3a*S*,7a*S*)-3a,5-Dimethyl-3a,4,5,7a-tetrahydrobenzofuran-2(3H)-one (2p)

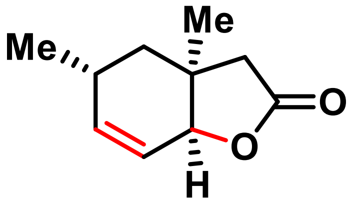

Eluent: ethyl acetate/ petroleum ether (6:94 v/v).

Appearance: colorless gummy.

Isolated yield: 78% (26 mg, 0.16 mmol)

^{1}H NMR (400 MHz, $CDCl_3$) δ 5.82 – 5.73 (m, 1H), 5.63 (dt, *J* = 10.1, 2.7 Hz, 1H), 4.54 (dt, *J* = 2.7, 1.3 Hz, 1H), 2.60 (d, *J* = 17.1 Hz, 1H), 2.36 – 2.24 (m, 1H), 2.02 (d, *J* = 17.0 Hz, 1H), 1.86 (ddd, *J* = 14.2, 5.1, 1.3 Hz, 1H), 1.37 – 1.24 (m, 1H), 1.22 (d, *J* = 0.7 Hz, 3H), 1.04 (d, *J* = 7.0 Hz, 3H); **^{13}C NMR** (126 MHz, $CDCl_3$) δ 176.91, 137.35, 124.31, 82.90, 39.43, 38.28, 38.04, 27.92, 27.25, 21.29; **HRMS (ESI)**: Calculated mass for $C_{10}H_{14}NaO_2$ $[M+Na]^+$: 189.0891; observed mass: 189.0895.

(3a*S*,7a*S*)-3a-Ethyl-5-methyl-3a,4,5,7a-tetrahydrobenzofuran-2(3H)-one (2q)

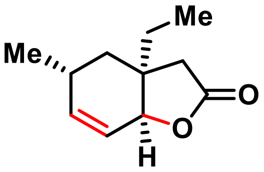

Eluent: ethyl acetate/ petroleum ether (6:94 v/v).

Appearance: colorless liquid.

Isolated yield: 75% (27 mg, 0.15 mmol)

^{1}H NMR (400 MHz, $CDCl_3$) δ 5.78 (dq, *J* = 10.1, 1.5 Hz, 1H), 5.69 – 5.56 (m, 1H), 4.61 (td, *J* = 2.6, 1.1 Hz, 1H), 2.55 (d, *J* = 17.3 Hz, 1H), 2.30 (dddd, *J* = 12.3, 6.9, 4.7, 2.3 Hz, 1H), 2.13 (d, *J* = 17.2 Hz, 1H), 1.83 (ddd, *J* = 14.2, 5.1, 1.3 Hz, 1H), 1.58 – 1.48 (m, 2H), 1.18 (dd, *J* = 14.1, 10.6 Hz, 1H), 1.05 (d, *J* = 7.1 Hz, 3H), 0.94 (t, *J* = 7.5 Hz, 3H); **^{13}C NMR** (101 MHz, $CDCl_3$) δ 177.09, 137.50, 124.55, 81.30, 41.33, 36.47, 35.82, 33.12, 27.01, 21.42, 8.22; **HRMS (ESI)**: Calculated mass for $C_{11}H_{17}O_2$ $[M+H]^+$: 181.1229; observed mass: 181.1235.

(3a*S*,7a*S*)-3a-Isobutyl-5-methyl-3a,4,5,7a-tetrahydrobenzofuran-2(3H)-one (2r)

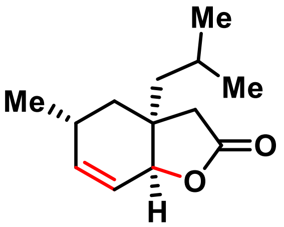

Eluent: ethyl acetate/ petroleum ether (6:94 v/v).

Appearance: colorless gummy.

Isolated yield: 78% (32.5 mg, 0.156 mmol)

^{1}H NMR (400 MHz, $CDCl_3$) δ 5.82 – 5.73 (m, 1H), 5.63 (dt, *J* = 10.1, 2.8 Hz, 1H), 4.59 (td, *J* = 2.7, 1.2 Hz, 1H), 2.55 (d, *J* = 17.2 Hz, 1H), 2.29 (dddt, *J* = 9.7, 7.2, 4.9, 2.4 Hz, 1H), 2.16 (d, *J* = 17.2 Hz, 1H), 1.87 (ddd, *J* = 14.0, 5.1, 1.3 Hz, 1H), 1.79 – 1.68 (m, 1H), 1.43 (d, *J* = 5.7 Hz, 2H), 1.26 (dd, *J* = 14.1, 10.3 Hz, 1H), 1.04 (d, *J* = 7.1 Hz, 3H), 0.96 (d, *J* = 6.6 Hz, 6H); **^{13}C NMR** (101 MHz, $CDCl_3$) δ 177.01, 137.44, 124.31, 81.81, 48.83, 41.83, 36.95, 36.91, 27.07, 25.34, 25.15, 24.51, 21.41; **HRMS (ESI)**: Calculated mass for $C_{13}H_{21}O_2$ $[M+H]^+$: 209.1542; observed mass: 209.1550.

(3a*S*,7a*S*)-3a-Methyl-5-phenyl-3a,4,5,7a-tetrahydrobenzofuran-2(3H)-one (2s)

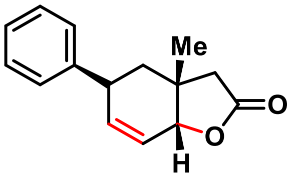

Eluent: ethyl acetate/ petroleum ether (8:92 v/v).

Appearance: colorless gummy.

Isolated yield: 64% (29.5 mg, 0.13 mmol)

^{1}H NMR (400 MHz, $CDCl_3$) δ 7.45 – 7.35 (m, 1H), 7.34 – 7.28 (m, 1H), 7.29 – 7.21 (m, 1H), 7.20 – 7.13 (m, 2H), 6.08 – 5.93 (m, 1H), 5.87 (dt, *J* = 10.2, 2.7 Hz, 1H), 4.68 (q, *J* = 1.4 Hz, 1H), 3.48 (ddd, *J* = 10.8, 5.2, 2.6 Hz, 1H), 2.77 (d, *J* = 17.1 Hz, 1H), 2.28 – 1.96 (m, 2H), 1.63 (d, *J* = 3.3 Hz, 1H), 1.24 (s, 3H); **^{13}C NMR** (101 MHz, $CDCl_3$) δ 176.55, 144.17, 134.58, 129.07, 129.00, 127.96, 127.64, 127.06, 126.07, 82.52, 40.86, 39.21, 38.61, 37.94, 27.68; **HRMS (ESI)**: Calculated mass for $C_{15}H_{17}O_2$ $[M+H]^+$: 229.1229; observed mass: 229.1235.

(3a*S*,7a*R*)-3a-Methyl-7-phenyl-3a,4,5,7a-tetrahydrobenzofuran-2(3H)-one (2t)

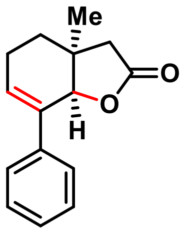

Eluent: ethyl acetate/ petroleum ether (10:90 v/v).

Appearance: colorless gummy.

Isolated yield: 10% (5 mg, 0.02 mmol)

^{1}H NMR (400 MHz, $CDCl_3$) δ 7.54 – 7.45 (m, 2H), 7.34 (t, *J* = 7.4 Hz, 2H), 7.30 – 7.26 (m, 1H), 6.45 (dd, *J* = 4.8, 3.4 Hz, 1H), 4.78 (d, *J* = 1.2 Hz, 1H), 2.60 – 2.46 (m, 2H), 2.39 – 2.28

(m, 2H), 1.72 (ddd, J = 13.4, 9.4, 6.5 Hz, 1H), 1.56 (dd, J = 9.2, 4.7 Hz, 1H), 1.21 (s, 3H); **^{13}C NMR** (101 MHz, $CDCl_3$) δ 176.20, 139.54, 132.93, 130.39, 128.70, 128.47, 127.74, 126.06, 82.28, 43.92, 38.14, 28.99, 22.95, 21.71; **HRMS (ESI)**: Calculated mass for $C_{15}H_{17}O_2$ $[M+H]^+$: 229.1229; observed mass: 229.1231.

(3a*S*,7a*S*)-3a-Methyl-5-(*p*-tolyl)-3a,4,5,7a-tetrahydrobenzofuran-2(3H)-one (2u)

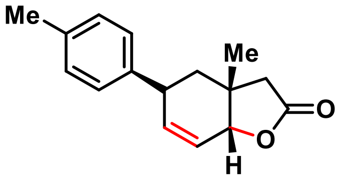

Eluent: ethyl acetate/ petroleum ether (6:94 v/v).

Appearance: colorless gummy.

Isolated yield: 66% (32 mg, 0.13 mmol)

^{1}H NMR (400 MHz, $CDCl_3$) δ 7.15 (d, J = 7.9 Hz, 2H), 7.06 (d, J = 8.1 Hz, 2H), 6.02 – 5.94 (m, 1H), 5.85 (dt, J = 10.2, 2.8 Hz, 1H), 4.67 (td, J = 2.6, 1.3 Hz, 1H), 3.44 (ddd, J = 10.8, 5.2, 2.6 Hz, 1H), 2.77 (d, J = 17.0 Hz, 1H), 2.34 (s, 3H), 2.12 (d, J = 17.0 Hz, 1H), 2.07 (ddd, J = 14.3, 5.3, 1.3 Hz, 1H), 1.61 (dd, J = 14.4, 11.0 Hz, 1H), 1.23 (s, 3H); **^{13}C NMR** (101 MHz, $CDCl_3$) δ 176.60, 141.15, 136.66, 134.84, 129.65, 127.49, 125.86, 82.55, 40.91, 38.76, 38.61, 37.90, 27.66, 21.19; **HRMS (ESI)**: Calculated mass for $C_{16}H_{19}O_2$ $[M+H]^+$: 243.1385.; observed mass: 243.1387.

(3a*S*,7a*R*)-3a-Methyl-7-(*p*-tolyl)-3a,4,5,7a-tetrahydrobenzofuran-2(3H)-one (2v)

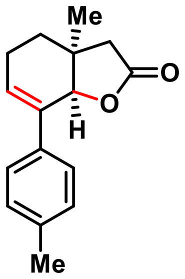

Eluent: ethyl acetate/ petroleum ether (8:92 v/v).

Appearance: colorless gummy.

Isolated yield: 10% (5 mg, 0.02 mmol)

^{1}H NMR (400 MHz, $CDCl_3$) δ 7.38 (d, J = 8.3 Hz, 2H), 7.15 (d, J = 8.1 Hz, 2H), 6.41 (dd, J = 4.7, 3.4 Hz, 1H), 4.77 (s, 1H), 2.49 (d, J = 11.2 Hz, 2H), 2.33 (d, J = 7.9 Hz, 5H), 1.70 (ddd, J = 13.4, 9.3, 6.7 Hz, 1H), 1.59 – 1.50 (m, 1H), 1.20 (s, 3H); **^{13}C NMR** (101 MHz, $CDCl_3$) δ 176.26, 137.48, 136.68, 132.66, 129.49, 129.36, 125.89, 82.36, 43.93, 38.10, 29.00, 22.89,

21.69, 21.27; **HRMS (ESI)**: Calculated mass for $C_{16}H_{19}O_2$ $[M+H]^+$: 243.1385.; observed mass: 243.1390.

(3a*S*,7a*S*)-5-(2-Fluorophenyl)-3a-methyl-3a,4,5,7a-tetrahydrobenzofuran-2(3H)-one (2w)

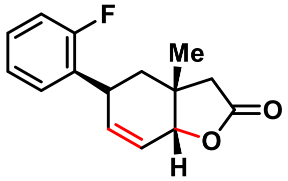

Eluent: ethyl acetate/ petroleum ether (10:90 v/v).

Appearance: white solid.

Isolated yield: 72% (35.5 mg, 0.14 mmol)

^{1}H NMR (400 MHz, $CDCl_3$) δ 7.25 – 7.19 (m, 1H), 7.17 – 7.09 (m, 2H), 7.04 (ddd, *J* = 10.4, 8.0, 1.1 Hz, 1H), 5.98 – 5.92 (m, 1H), 5.90 (dt, *J* = 10.2, 2.6 Hz, 1H), 4.67 (td, *J* = 2.6, 1.1 Hz, 1H), 3.84 (ddt, *J* = 10.1, 5.2, 2.4 Hz, 1H), 2.79 (d, *J* = 17.0 Hz, 1H), 2.21 – 2.06 (m, 2H), 1.64 – 1.56 (m, 1H), 1.24 (s, 3H); **^{13}C NMR** (101 MHz, $CDCl_3$) δ 176.42, 161.97, 159.53, 133.48, 130.81, 130.67, 128.97, 128.92, 128.68, 128.60, 126.56, 124.77, 124.73, 115.86, 115.64, 82.35, 38.79, 38.54, 37.88, 32.21, 32.18, 27.64; **^{19}F NMR** (376 MHz, $CDCl_3$) δ -119.841; **HRMS (ESI)**: Calculated mass for $C_{15}H_{16}FO_2$ $[M+H]^+$: 247.1134; observed mass: 247.1140.

(3a*S*,7a*S*)-3a-Methyl-5-(naphthalen-1-yl)-3a,4,5,7a-tetrahydrobenzofuran-2(3H)-one (2x)

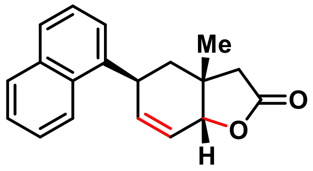

Eluent: ethyl acetate/ petroleum ether (10:90 v/v).

Appearance: colorless gummy.

Isolated yield: 68% (38 mg, 0.14 mmol)

^{1}H NMR (400 MHz, $CDCl_3$) δ 8.04 (dt, *J* = 8.1, 0.9 Hz, 1H), 7.94 – 7.86 (m, 1H), 7.81 – 7.74 (m, 1H), 7.60 – 7.47 (m, 2H), 7.46 (dd, *J* = 8.2, 7.2 Hz, 1H), 7.31 (dd, *J* = 7.2, 1.2 Hz, 1H), 6.26 – 6.12 (m, 1H), 5.99 (dt, *J* = 10.2, 2.8 Hz, 1H), 4.76 (td, *J* = 2.6, 1.3 Hz, 1H), 4.32 (ddd, *J* = 10.6, 5.1, 2.5 Hz, 1H), 2.94 (d, *J* = 16.9 Hz, 1H), 2.31 (ddd, *J* = 14.4, 5.3, 1.4 Hz, 1H), 2.24 (d, *J* = 16.9 Hz, 1H), 1.76 (dd, *J* = 14.5, 10.9 Hz, 1H), 1.24 (s, 3H); **^{13}C NMR** (101 MHz, $CDCl_3$) δ 176.49, 140.00, 135.02, 134.21, 131.22, 129.42, 127.68, 126.47, 126.26, 125.92,

124.86, 122.77, 82.72, 39.83, 38.75, 38.19, 27.60; **HRMS (ESI)**: Calculated mass for $C_{19}H_{19}O_2$ $[M+H]^+$: 279.1385; observed mass: 279.1390.

(3a*S*,7a*S*)-3a,6-Dimethyl-3a,4,5,7a-tetrahydrobenzofuran-2(3H)-one (2y)

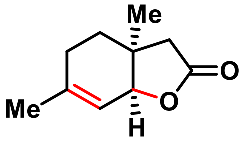

Eluent: ethyl acetate/ petroleum ether (8:92 v/v).

Appearance: colorless gummy.

Isolated yield: 65% (21.5 mg, 0.13 mmol)

^{1}H NMR (400 MHz, $CDCl_3$) δ 5.54 (dq, *J* = 4.5, 1.6 Hz, 1H), 4.67 – 4.14 (m, 1H), 2.42 (d, *J* = 17.1 Hz, 1H), 2.33 (d, *J* = 17.0 Hz, 1H), 2.16 – 1.90 (m, 2H), 1.76 (t, *J* = 1.2 Hz, 3H), 1.73 – 1.61 (m, 1H), 1.56 – 1.48 (m, 1H), 1.14 (s, 3H); **^{13}C NMR** (101 MHz, $CDCl_3$) δ 176.53, 141.83, 117.61, 82.47, 42.58, 36.75, 30.11, 26.95, 23.72, 23.18; **HRMS (ESI)**: Calculated mass for $C_{10}H_{15}O_2$ $[M+H]^+$: 167.1072; observed mass: 167.1075.

(3a*S*,7a*S*)-6-Methyl-3a-propyl-3a,4,5,7a-tetrahydrobenzofuran-2(3H)-one (2z)

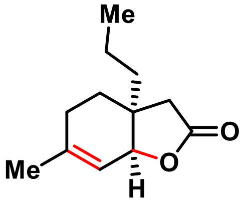

Eluent: ethyl acetate/ petroleum ether (8:92 v/v).

Appearance: colorless gummy.

Isolated yield: 56% (22 mg, 0.112 mmol)

^{1}H NMR (400 MHz, $CDCl_3$) δ 5.63 – 5.48 (m, 1H), 4.61 – 4.26 (m, 1H), 2.48 – 2.27 (m, 2H), 1.98 (tdd, *J* = 5.1, 1.8, 0.9 Hz, 2H), 1.76 (t, *J* = 1.2 Hz, 3H), 1.69 – 1.61 (m, 1H), 1.51 – 1.44 (m, 1H), 1.43 – 1.32 (m, 1H), 1.32 – 1.17 (m, 3H), 0.94 (t, *J* = 7.1 Hz, 3H); **^{13}C NMR** (126 MHz, $CDCl_3$) δ 176.76, 142.22, 117.56, 81.91, 40.80, 40.13, 38.01, 27.09, 26.73, 23.75, 18.08, 14.84; **HRMS (ESI)**: Calculated mass for $C_{12}H_{18}NaO_2$ $[M+Na]^+$: 217.1204; observed mass: 217.1208.

(3a*S*,7a*S*)-3a-Methyl-6-propyl-3a,4,5,7a-tetrahydrobenzofuran-2(3H)-one (2aa)

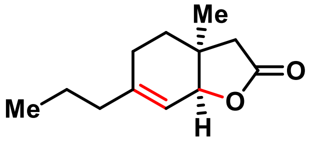

Eluent: ethyl acetate/ petroleum ether (7:93 v/v).

Appearance: colorless gummy.

Isolated yield: 64% (25 mg, 0.13 mmol) (d.r. = 6:1)

^{1}H NMR (500 MHz, $CDCl_3$) δ 5.54 (dt, *J* = 4.4, 1.6 Hz, 1H), 4.43 (d, *J* = 4.1 Hz, 1H), 2.42 (d, *J* = 17.1 Hz, 1H), 2.34 (d, *J* = 17.1 Hz, 1H), 2.02 (t, *J* = 7.6 Hz, 4H), 1.71 – 1.62 (m, 1H), 1.46 (qd, *J* = 7.4, 2.3 Hz, 1H), 1.25 (d, *J* = 2.3 Hz, 2H), 1.14 (s, 3H), 0.89 (t, *J* = 7.4 Hz, 3H); **^{13}C NMR** (126 MHz, $CDCl_3$) δ 176.61, 145.51, 117.05, 82.51, 42.61, 39.64, 36.97, 30.13, 29.91, 25.27, 23.15, 20.67, 13.91; **HRMS (ESI)**: Calculated mass for $C_{12}H_{19}O_2$ $[M+H]^+$: 195.1385; observed mass: 195.1387.

(3a*S*,7a*S*)-6-(*tert*-Butyl)-3a-methyl-3a,4,5,7a-tetrahydrobenzofuran-2(3H)-one (2ab)

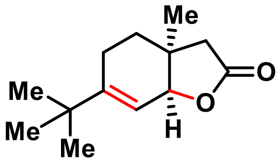

Eluent: ethyl acetate/ petroleum ether (8:92 v/v).

Appearance: colorless gummy.

Isolated yield: 55% (23 mg, 0.11 mmol)

^{1}H NMR (400 MHz, $CDCl_3$) δ 5.61 (d, *J* = 4.2 Hz, 1H), 4.46 (d, *J* = 4.2 Hz, 1H), 2.43 (d, *J* = 17.1 Hz, 1H), 2.34 (d, *J* = 17.1 Hz, 1H), 2.21 – 1.98 (m, 2H), 1.73 – 1.60 (m, 1H), 1.56 – 1.48 (m, 1H), 1.14 (s, 3H), 1.06 (s, 9H); **^{13}C NMR** (126 MHz, $CDCl_3$) δ 176.59, 153.09, 114.22, 82.95, 42.53, 36.67, 35.90, 30.73, 28.91, 23.25, 21.34; **HRMS (ESI)**: Calculated mass for $C_{13}H_{21}O_2$ $[M+H]^+$: 209.1542; observed mass: 209.1540.

(3a*S*,7a*S*)-3a-Methyl-6-(*tert*-pentyl)-3a,4,5,7a-tetrahydrobenzofuran-2(3H)-one (2ac)

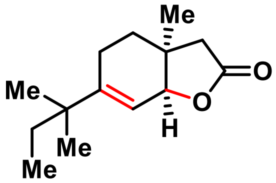

Eluent: ethyl acetate/ petroleum ether (8:92 v/v).

Appearance: colorless gummy.

Isolated yield: 57% (25.5 mg, 0.114 mmol)

^{1}H NMR (500 MHz, $CDCl_3$) δ 5.57 (dt, J = 4.2, 1.6 Hz, 1H), 4.47 (d, J = 4.2 Hz, 1H), 2.42 (d, J = 17.1 Hz, 1H), 2.35 (d, J = 17.1 Hz, 1H), 2.17 – 2.05 (m, 1H), 1.99 (dddt, J = 17.7, 8.6, 5.2, 1.6 Hz, 1H), 1.62 (td, J = 8.8, 4.4 Hz, 1H), 1.53 (dt, J = 13.5, 5.3 Hz, 1H), 1.38 (qd, J = 7.5, 2.1 Hz, 2H), 1.14 (s, 3H), 1.02 (s, 6H), 0.68 (t, J = 7.5 Hz, 3H); **^{13}C NMR** (126 MHz, $CDCl_3$) δ 176.63, 151.19, 116.17, 82.91, 42.61, 39.15, 36.67, 33.31, 30.64, 26.81, 26.60, 23.17, 21.16, 9.21; **HRMS (ESI)**: Calculated mass for $C_{14}H_{23}O_2$ $[M+H]^+$: 223.1698; observed mass: 223.1695.

(3a*S*,7a*S*)-3a-Ethyl-6-(*tert*-pentyl)-3a,4,5,7a-tetrahydrobenzofuran-2(3H)-one (2ad)

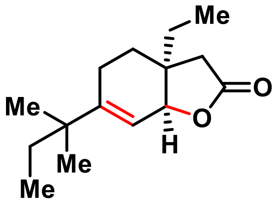

Eluent: ethyl acetate/ petroleum ether (8:92 v/v).

Appearance: colorless gummy.

Isolated yield: 60% (28.5 mg, 0.12 mmol)

^{1}H NMR (400 MHz, $CDCl_3$) δ 5.57 (d, J = 4.4 Hz, 1H), 4.47 (d, J = 4.3 Hz, 1H), 2.37 (d, J = 5.0 Hz, 2H), 2.14 – 1.99 (m, 1H), 1.99 – 1.88 (m, 1H), 1.65 (dd, J = 13.7, 5.1 Hz, 1H), 1.58 – 1.46 (m, 2H), 1.45 – 1.31 (m, 3H), 1.01 (d, J = 1.4 Hz, 6H), 0.92 (t, J = 7.5 Hz, 3H), 0.68 (t, J = 7.5 Hz, 3H); **^{13}C NMR** (101 MHz, $CDCl_3$) δ 176.72, 151.40, 116.29, 82.24, 40.29, 40.19, 39.16, 33.32, 28.39, 27.17, 26.79, 26.59, 20.88, 9.23, 9.04; **HRMS (ESI)**: Calculated mass for $C_{15}H_{25}O_2$ $[M+H]^+$: 237.1855; observed mass: 237.1860.

(3a*S*,7a*S*)-3a-Methyl-6-(trifluoromethyl)-3a,4,5,7a-tetrahydrobenzofuran-2(3H)-one (2ae)

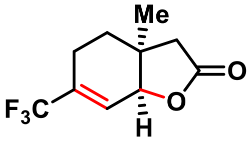

Eluent: ethyl acetate/ petroleum ether (10:90 v/v).

Appearance: colorless gummy.

Isolated yield: 85% (37.5 mg, 0.17 mmol)

^{1}H NMR (400 MHz, $CDCl_3$) δ 6.43 – 6.34 (m, 1H), 4.56 – 4.42 (m, 1H), 2.48 (d, J = 17.3 Hz, 1H), 2.39 (dd, J = 17.3, 0.8 Hz, 1H), 2.33 – 2.22 (m, 2H), 1.80 – 1.65 (m, 2H), 1.21 (s, 3H);

^{13}C NMR (101 MHz, $CDCl_3$) δ 175.15, 133.91, 133.60, 133.29, 125.14, 125.09, 125.03, 124.98, 124.47, 121.76, 79.46, 41.38, 37.02, 29.15, 23.46, 19.21; **^{19}F NMR** (376 MHz, $CDCl_3$) δ -70.12; **HRMS (ESI)**: Calculated mass for $C_{10}H_{12}F_3O_2$ $[M+H]^+$: 221.0789; observed mass: 221.0795.

(3a*S*,7a*S*)-3a-Methyl-6-phenyl-3a,4,5,7a-tetrahydrobenzofuran-2(3H)-one (2af)

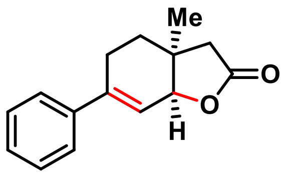

Eluent: ethyl acetate/ petroleum ether (10:90 v/v).

Appearance: colorless gummy.

Isolated yield: 60% (28 mg, 0.12 mmol)

^{1}H NMR (400 MHz, $CDCl_3$) δ 7.44 – 7.40 (m, 2H), 7.38 – 7.28 (m, 3H), 6.15 (dd, J = 4.2, 1.8 Hz, 1H), 5.06 – 4.50 (m, 1H), 2.57 – 2.51 (m, 2H), 2.50 (s, 1H), 2.41 (d, J = 17.1 Hz, 1H), 1.83 (ddd, J = 13.8, 7.6, 6.2 Hz, 1H), 1.72 (dt, J = 13.7, 5.4 Hz, 1H), 1.24 (s, 3H); **^{13}C NMR** (101 MHz, $CDCl_3$) δ 176.28, 142.93, 140.34, 128.70, 128.44, 125.77, 119.22, 82.30, 42.35, 36.89, 30.33, 24.39, 23.32; **HRMS (ESI)**: Calculated mass for $C_{15}H_{17}O_2$ $[M+H]^+$: 229.1229; observed mass: 229.1219.

(3a*S*,6a*S*)-3a-Methyl-3,3a,4,6a-tetrahydro-2H-cyclopenta[b]furan-2-one (3a)

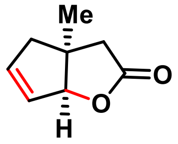

Eluent: ethyl acetate/ petroleum ether (6:94 v/v).

Appearance: colorless liquid.

Isolated yield: 62% (17 mg, 0.123 mmol) (d.r. = 7:1)

^{1}H NMR (400 MHz, $CDCl_3$) δ 6.04 (dt, J = 5.2, 2.4 Hz, 1H), 5.88 – 5.76 (m, 1H), 5.03 (dt, J = 2.1, 1.1 Hz, 1H), 2.66 – 2.30 (m, 4H), 1.35 (s, 3H); **^{13}C NMR** (101 MHz, $CDCl_3$) δ 176.83, 136.92, 136.64, 129.12, 128.00, 95.40, 88.78, 46.58, 44.40, 43.38, 40.53, 38.51, 32.14, 29.91, 25.61, 23.39; **HRMS (ESI)**: Calculated mass for $C_8H_{11}O_2$ $[M+H]^+$: 139.0759; observed mass: 139.0765.

(3a*S*,6a*S*)-3a-Propyl-3,3a,4,6a-tetrahydro-2H-cyclopenta[b]furan-2-one (3b)

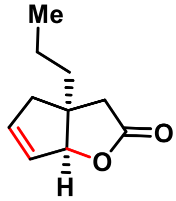

Eluent: ethyl acetate / petroleum ether (6:94 v/v)

Appearance: colorless liquid.

Isolated yield: 59% (19.5 mg, 0.117 mmol) (d.r. = 5:1)

^{1}H NMR (400 MHz, $CDCl_3$) δ 6.03 (dt, *J* = 5.2, 2.4 Hz, 1H), 5.82 (dq, *J* = 6.1, 2.1 Hz, 1H), 5.11 (d, *J* = 2.1 Hz, 1H), 2.63 – 2.53 (m, 1H), 2.51 – 2.40 (m, 3H), 1.59 – 1.51 (m, 2H), 1.40 – 1.29 (m, 2H), 0.96 (t, *J* = 7.3 Hz, 3H); **^{13}C NMR** (101 MHz, $CDCl_3$) δ 177.04, 136.55, 135.14, 132.52, 129.29, 128.94, 94.14, 87.87, 48.42, 44.56, 41.94, 41.33, 41.10, 40.23, 39.34, 39.12, 18.53, 14.74; **HRMS (ESI)**: Calculated mass for $C_{10}H_{14}NaO_2$ $[M+Na]^+$: 189.0891; observed mass: 189.0895.

(3a*S*,6a*S*)-3a-Isopropyl-3,3a,4,6a-tetrahydro-2H-cyclopenta[b]furan-2-one (3c)

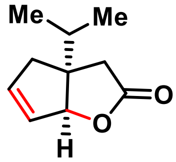

Eluent: ethyl acetate/ petroleum ether (6:94 v/v).

Appearance: colorless gummy.

Isolated yield: 57% (19 mg, 0.114 mmol) (d.r. = 4:1)

^{1}H NMR (400 MHz, $CDCl_3$) δ 6.02 (dtd, *J* = 5.8, 2.3, 0.9 Hz, 1H), 5.82 (dq, *J* = 6.1, 2.0 Hz, 1H), 5.21 (dq, *J* = 2.6, 1.3 Hz, 1H), 2.62 (d, *J* = 18.3 Hz, 1H), 2.47 (dq, *J* = 17.5, 2.1 Hz, 1H), 2.40 – 2.30 (m, 2H), 1.84 (p, *J* = 6.8 Hz, 1H), 0.93 (dd, *J* = 17.2, 6.8 Hz, 6H); **^{13}C NMR** (101 MHz, $CDCl_3$) δ 177.24, 136.22, 132.50, 130.25, 129.65, 93.09, 86.98, 52.49, 43.14, 39.84, 38.69, 38.56, 35.43, 34.89, 19.04, 18.05, 17.87; **HRMS (ESI)**: Calculated mass for $C_{10}H_{15}O_2$ $[M+H]^+$: 167.1072; observed mass: 167.1064.

(3a*S*,6a*S*)-3a-Cyclohexyl-3,3a,4,6a-tetrahydro-2H-cyclopenta[b]furan-2-one (3d)

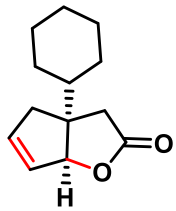

Eluent: ethyl acetate/ petroleum ether (6:94 v/v).

Appearance: colorless gummy.

Isolated yield: 70% (29 mg, 0.14 mmol) (d.r. = 8:1)

^{1}H NMR (400 MHz, $CDCl_3$) δ 6.01 (dt, *J* = 5.7, 2.5 Hz, 1H), 5.81 (dq, *J* = 6.0, 2.0 Hz, 1H), 5.24 (q, *J* = 2.3 Hz, 1H), 2.63 (d, *J* = 18.1 Hz, 1H), 2.47 (dq, *J* = 17.2, 2.1 Hz, 1H), 2.37 – 2.28 (m, 2H), 1.79 – 1.57 (m, 5H), 1.45 (tt, *J* = 12.0, 3.1 Hz, 1H), 1.28 – 1.09 (m, 3H), 1.07 – 0.97 (m, 2H); **^{13}C NMR** (101 MHz, $CDCl_3$) δ 177.26, 136.05, 133.14, 129.86, 129.57, 92.85, 86.98, 52.35, 45.88, 45.41, 43.17, 39.88, 38.94, 38.58, 29.14, 28.29, 28.21, 28.06, 26.63, 26.51, 26.48, 26.46, 26.36, 26.26; **HRMS (ESI)**: Calculated mass for $C_{13}H_{19}O_2$ $[M+H]^+$: 207.1385; observed mass: 207.1380.

(3a*S*,6a*S*)-3a,5-Dimethyl-3,3a,4,6a-tetrahydro-2H-cyclopenta[b]furan-2-one (3e)

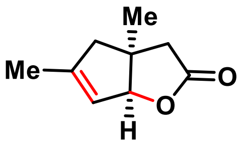

Eluent: ethyl acetate/ petroleum ether (6:94 v/v).

Appearance: colorless gummy.

Isolated yield: 53% (16 mg, 0.105 mmol)

^{1}H NMR (400 MHz, $CDCl_3$) δ 6.04 (s, 1H), 4.99 (d, *J* = 2.0 Hz, 1H), 4.53 – 4.34 (m, 2H), 4.10 (p, *J* = 5.9 Hz, 1H), 2.59 (d, *J* = 18.1 Hz, 1H), 2.55 – 2.41 (m, 3H), 1.38 (s, 3H); **^{13}C NMR** (101 MHz, $CDCl_3$) δ 176.48, 136.90, 135.33, 93.36, 70.78, 45.92, 44.73, 43.31, 29.91, 25.74; **HRMS (ESI)**: Calculated mass for $C_9H_{13}O_2$ $[M+H]^+$: 153.0916; observed mass: 153.0920.

(3a*S*,6a*S*)-3a-Ethyl-5-methyl-3,3a,4,6a-tetrahydro-2H-cyclopenta[b]furan-2-one (3f)

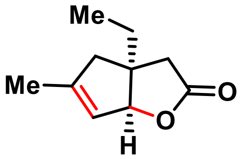

Eluent: ethyl acetate/ petroleum ether (7:93 v/v).

Appearance: colorless gummy.

Isolated yield: 51% (17 mg, 0.10 mmol)

^{1}H NMR (400 MHz, $CDCl_3$) δ 6.03 (s, 1H), 5.07 (s, 1H), 4.50 – 4.31 (m, 2H), 4.10 (p, *J* = 5.9 Hz, 1H), 2.61 (d, *J* = 18.2 Hz, 1H), 2.57 – 2.36 (m, 3H), 1.67 (q, *J* = 7.4 Hz, 2H), 0.97 (t, *J* = 7.4 Hz, 3H); **^{13}C NMR** (101 MHz, $CDCl_3$) δ 176.61, 137.07, 134.95, 91.77, 70.75, 49.16, 43.54, 40.84, 31.34, 9.29; **HRMS (ESI)**: Calculated mass for $C_{10}H_{15}O_2$ $[M+H]^+$: 167.1072; observed mass: 167.1075.

(3a*S*,8a*S*)-3a-Methyl-3,3a,4,5,6,8a-hexahydro-2H-cyclohepta[b]furan-2-one (3g)

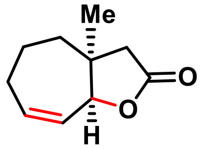

Eluent: ethyl acetate/ petroleum ether (7:93 v/v).

Appearance: colorless gummy.

Isolated yield: 70% (23.2 mg, 0.139 mmol)

^{1}H NMR (400 MHz, $CDCl_3$) δ 5.71 (dtd, *J* = 11.6, 5.3, 2.2 Hz, 1H), 5.57 (ddt, *J* = 11.7, 3.4, 1.6 Hz, 1H), 4.85 (dq, *J* = 4.0, 2.1 Hz, 1H), 2.43 (d, *J* = 17.2 Hz, 1H), 2.29 (d, *J* = 17.1 Hz, 1H), 2.21 (dtt, *J* = 6.8, 5.0, 1.8 Hz, 2H), 1.76 – 1.55 (m, 4H), 1.22 (s, 3H); **^{13}C NMR** (101 MHz, $CDCl_3$) δ 176.53, 130.80, 127.29, 127.25, 86.34, 86.28, 43.88, 43.79, 43.76, 43.73, 42.52, 35.73, 28.94, 25.89, 25.83, 25.69, 19.06; **HRMS (ESI)**: Calculated mass for $C_{10}H_{15}O_2$ $[M+H]^+$: 167.1072; observed mass: 167.1080.

(3a*S*,8a*S*)-3a-Ethyl-3,3a,4,5,6,8a-hexahydro-2H-cyclohepta[b]furan-2-one (3h)

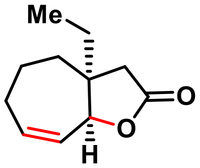

Eluent: ethyl acetate/ petroleum ether (8:92 v/v).

Appearance: colorless gummy.

Isolated yield: 66% (24 mg, 0.133 mmol) (d.r. = 7:1)

^{1}H NMR (400 MHz, $CDCl_3$) δ 5.75 (dtd, *J* = 11.3, 5.6, 2.0 Hz, 1H), 5.62 (ddd, *J* = 11.5, 4.1, 1.6 Hz, 1H), 4.84 (dd, *J* = 3.9, 1.9 Hz, 1H), 2.44 (d, *J* = 17.6 Hz, 1H), 2.29 (d, *J* = 17.5 Hz, 1H), 2.19 (d, *J* = 14.2 Hz, 2H), 1.85 – 1.69 (m, 1H), 1.69 – 1.49 (m, 5H), 0.92 (t, *J* = 7.5 Hz, 3H); **^{13}C NMR** (101 MHz, $CDCl_3$) δ 176.84, 131.11, 127.71, 86.04, 45.42, 41.32, 32.84, 30.55, 28.63, 18.90, 8.67; **HRMS (ESI)**: Calculated mass for $C_{11}H_{16}NaO_2$ $[M+Na]^+$: 203.1048; observed mass: 203.1050.

(3a*S*,8a*S*)-3a-Isopropyl-3,3a,4,5,6,8a-hexahydro-2H-cyclohepta[b]furan-2-one (3i)

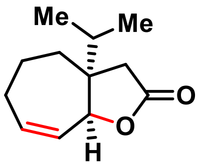

Eluent: ethyl acetate/ petroleum ether (10:90 v/v).

Appearance: colorless gummy.

Isolated yield: 71% (27.5 mg, 0.14 mmol) (d.r. = 8:1)

^{1}H NMR (400 MHz, $CDCl_3$) δ 5.80 (dtd, *J* = 11.2, 5.7, 1.3 Hz, 1H), 5.72 (ddd, *J* = 11.1, 4.8, 1.8 Hz, 1H), 4.94 – 4.80 (m, 1H), 2.51 (d, *J* = 18.0 Hz, 1H), 2.37 – 2.25 (m, 1H), 2.20 (d, *J* = 17.9 Hz, 1H), 2.16 – 2.01 (m, 1H), 1.90 – 1.80 (m, 1H), 1.79 – 1.70 (m, 1H), 1.57 – 1.45 (m, 3H), 0.92 (dt, *J* = 6.8, 4.9 Hz, 6H); **^{13}C NMR** (101 MHz, $CDCl_3$) δ 177.10, 131.92, 127.86, 85.56, 85.52, 47.85, 40.05, 34.69, 28.21, 18.37, 18.01, 17.70; **HRMS (ESI)**: Calculated mass for $C_{12}H_{19}O_2$ $[M+H]^+$: 195.1385; observed mass: 195.1392.

(3a*R*,8a*S*)-3a-Isobutyl-3,3a,4,5,6,8a-hexahydro-2H-cyclohepta[b]furan-2-one (3j)

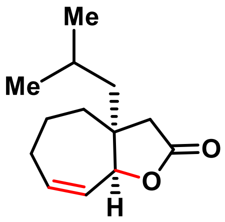

Eluent: ethyl acetate/ petroleum ether (8:92 v/v).

Appearance: colorless gummy.

Isolated yield: 64% (26.5 mg, 0.127 mmol) (d.r. = 7:1)

^{1}H NMR (400 MHz, $CDCl_3$) δ 5.83 (dddd, *J* = 11.8, 6.1, 4.9, 1.3 Hz, 1H), 5.62 (ddt, *J* = 11.7, 5.1, 1.7 Hz, 1H), 4.69 (dd, *J* = 5.1, 1.3 Hz, 1H), 2.54 (d, *J* = 17.3 Hz, 1H), 2.36 (d, *J* = 17.4 Hz, 1H), 2.24 (ddt, *J* = 8.0, 4.5, 1.5 Hz, 2H), 1.82 – 1.67 (m, 2H), 1.67 – 1.57 (m, 4H), 1.30 (dd, *J* = 14.2, 7.3 Hz, 1H), 0.95 (t, *J* = 6.8 Hz, 6H); **^{13}C NMR** (101 MHz, $CDCl_3$) δ 176.95, 133.84, 125.53, 86.34, 46.65, 46.19, 43.16, 33.71, 29.54, 25.29, 24.70, 24.38, 19.48; **HRMS (ESI)**: Calculated mass for $C_{13}H_{21}O_2$ $[M+H]^+$: 209.1542.; observed mass: 209.1548.

(3a*S*,8a*S*)-3a-Cyclohexyl-3,3a,4,5,6,8a-hexahydro-2H-cyclohepta[b]furan-2-one (3k)

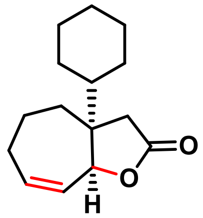

Eluent: ethyl acetate/ petroleum ether (8:92 v/v).

Appearance: colorless gummy.

Isolated yield: 68% (32 mg, 0.136 mmol) (d.r. = 4:1)

^{1}H NMR (400 MHz, $CDCl_3$) δ 6.14 – 5.60 (m, 2H), 4.84 (d, *J* = 4.9 Hz, 1H), 2.84 – 2.44 (m, 1H), 2.31 (dtd, *J* = 15.5, 6.2, 2.7 Hz, 1H), 2.20 (d, *J* = 17.9 Hz, 1H), 2.07 (ddt, *J* = 16.9, 7.6, 5.3 Hz, 1H), 1.89 – 1.36 (m, 10H), 1.31 – 0.91 (m, 5H); **^{13}C NMR** (101 MHz, $CDCl_3$) δ 177.20,

135.51, 131.95, 129.76, 127.76, 126.69, 125.66, 86.36, 85.47, 52.65, 47.94, 46.69, 45.57, 40.94, 39.66, 32.08, 31.39, 28.27, 28.04, 27.84, 27.69, 27.59, 26.93, 26.80, 26.77, 26.74, 26.54, 26.51, 18.35; **HRMS (ESI)**: Calculated mass for $C_{15}H_{22}NaO_2$ [M+H]$^+$: 257.1517; observed mass: 257.1520.

(3a*S*,8a*S*)-3a-Methyl-6-(*p*-tolyl)-3,3a,4,5,6,8a-hexahydro-2H-cyclohepta[b]furan-2-one (3l)

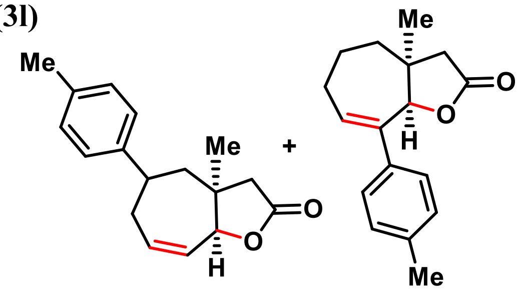

Eluent: ethyl acetate/ petroleum ether (8:92 v/v).

Appearance: colorless gummy.

Isolated yield: 59% (30.2 mg, 0.117 mmol) (r.r. = 2:1)

^{1}H NMR (400 MHz, $CDCl_3$) δ 7.23 – 7.02 (m, 12H), 6.05 (dd, *J* = 7.6, 5.1 Hz, 1H), 5.95 (dt, *J* = 12.3, 5.0 Hz, 2H), 5.72 (dddd, *J* = 12.2, 5.5, 2.4, 1.5 Hz, 2H), 5.04 (s, 1H), 4.73 (d, *J* = 5.5 Hz, 2H), 3.04 (tdd, *J* = 10.8, 4.6, 1.6 Hz, 2H), 2.71 (d, *J* = 17.4 Hz, 2H), 2.66 – 2.56 (m, 2H), 2.54 – 2.39 (m, 3H), 2.36 – 2.24 (m, 14H), 2.10 (dd, *J* = 14.5, 10.6 Hz, 2H), 1.95 – 1.85 (m, 3H), 1.71 (dd, *J* = 4.0, 1.7 Hz, 3H), 1.21 (d, *J* = 1.2 Hz, 9H); **^{13}C NMR** (101 MHz, $CDCl_3$) δ 176.69, 176.66, 144.58, 139.88, 137.01, 136.82, 136.18, 134.60, 132.46, 129.55, 129.49, 129.17, 127.26, 126.60, 124.20, 89.06, 85.98, 46.02, 44.46, 42.90, 42.07, 41.68, 39.09, 38.01, 36.89, 28.90, 27.29, 25.41, 21.24, 21.15, 19.37; **HRMS (ESI)**: Calculated mass for $C_{17}H_{21}O_2$ [M+H]$^+$: 257.1542; observed mass: 257.1550.

(3a*S*,13a*S*,*Z*)-3a-Methyl-3a,4,5,6,7,8,9,10,11,13a-decahydrocyclododeca[b]furan-2(3H)-one (3m)

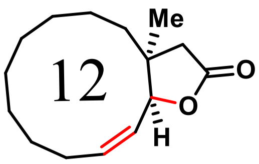

Eluent: ethyl acetate/ petroleum ether (10:90 v/v).

Appearance: colorless gummy.

Isolated yield: 55% (26 mg, 0.11 mmol) (d.r. = 4:1)

^{1}H NMR (400 MHz, $CDCl_3$) δ 5.93 (ddd, *J* = 14.9, 8.4, 6.1 Hz, 1H), 5.53 – 5.40 (m, 1H), 4.53 (d, *J* = 8.5 Hz, 1H), 2.45 (d, *J* = 17.1 Hz, 1H), 2.28 (d, *J* = 19.5 Hz, 1H), 2.23 – 2.07 (m, 2H),

1.55 – 1.23 (m, 14H), 1.19 (s, 3H); **^{13}C NMR** (101 MHz, $CDCl_3$) δ 176.48, 138.02, 137.89, 124.28, 122.98, 90.17, 80.40, 43.10, 42.50, 41.05, 34.99, 32.88, 32.84, 31.36, 27.90, 26.82, 26.56, 26.50, 26.34, 26.28, 25.43, 25.39, 25.23, 25.05, 24.65, 24.37, 22.64, 22.50, 22.14, 22.04, 21.77, 20.05, 19.94, 19.56; **HRMS (ESI)**: Calculated mass for $C_{15}H_{24}NaO_2$ $[M+Na]^+$: 259.1674; observed mass: 259.1678.

(3a*S*,13a*S*,*Z*)-3a-Ethyl-3a,4,5,6,7,8,9,10,11,13a-decahydrocyclododeca[b]furan-2(3H)-one (3n)

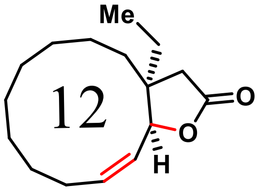

Eluent: ethyl acetate/ petroleum ether (10:90 v/v).

Appearance: colorless gummy.

Isolated yield: 58% (29 mg, 0.116 mmol)

^{1}H NMR (400 MHz, $CDCl_3$) δ 5.72 – 5.61 (m, 1H), 5.54 – 5.44 (m, 1H), 4.25 (dd, J = 9.9, 2.4 Hz, 1H), 2.65 – 2.54 (m, 2H), 2.44 – 2.31 (m, 2H), 2.27 – 2.17 (m, 2H), 1.93 – 1.79 (m, 2H), 1.69 (ddd, J = 13.3, 6.7, 4.9 Hz, 2H), 1.52 – 1.42 (m, 2H), 1.38 (dd, J = 14.4, 6.9 Hz, 2H), 0.94 (ddd, J = 11.3, 6.6, 2.7 Hz, 9H); **^{13}C NMR** (101 MHz, $CDCl_3$) δ 176.60, 137.34, 124.40, 90.17, 45.92, 39.71, 32.81, 31.86, 29.53, 27.67, 26.73, 26.68, 25.38, 24.69, 19.53, 8.51; **HRMS (ESI)**: Calculated mass for $C_{16}H_{27}O_2$ $[M+H]^+$: 251.2011; observed mass: 251.2015.

(3a*R*,16a*S*,*E*)-3a-Methyl-3,3a,4,5,6,7,8,9,10,11,12,13,14,16a-tetradecahydro-2H-cyclopentadeca[b]furan-2-one (3o)

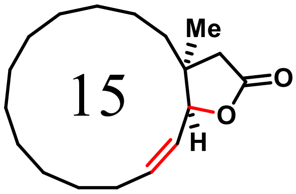

Eluent: ethyl acetate/ petroleum ether (8:92 v/v).

Appearance: colorless gummy.

Isolated yield: 50% (27.8 mg, 0.1 mmol) (d.r. = 3:1)

^{1}H NMR (400 MHz, $CDCl_3$) δ 5.84 (ddd, J = 14.8, 10.1, 4.2 Hz, 1H), 5.58 – 5.40 (m, 1H), 4.48 (d, J = 9.1 Hz, 1H), 2.44 (dd, J = 16.9, 5.0 Hz, 1H), 2.33 – 2.25 (m, 1H), 2.21 – 1.97 (m, 2H), 1.52 – 1.21 (m, 20H), 1.04 (s, 3H); **^{13}C NMR** (101 MHz, $CDCl_3$) δ 176.36, 140.00, 124.97, 90.05, 44.55, 43.67, 38.49, 31.98, 28.74, 27.80, 27.60, 27.38, 26.70, 26.59, 26.51, 25.72, 22.89,

19.43; **HRMS (ESI)**: Calculated mass for $C_{18}H_{31}O_2$ $[M+H]^+$: 279.2324; observed mass: 279.2328.

(3a*S*,16a*S*,*E*)-3a-Ethyl-3,3a,4,5,6,7,8,9,10,11,12,13,14,16a-tetradecahydro-2H-cyclopentadeca[b]furan-2-one (3p)

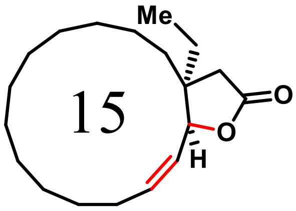

Eluent: ethyl acetate/ petroleum ether (8:92 v/v).

Appearance: colorless gummy.

Isolated yield: 54% (31.5 mg, 0.107 mmol)

^{1}H NMR (400 MHz, $CDCl_3$) δ 5.88 (ddd, *J* = 14.7, 10.1, 4.1 Hz, 1H), 5.60 (ddd, *J* = 15.2, 9.3, 1.9 Hz, 1H), 4.58 (d, *J* = 9.4 Hz, 1H), 2.49 (d, *J* = 17.1 Hz, 1H), 2.35 – 2.22 (m, 2H), 2.14 – 2.02 (m, 1H), 1.51 – 1.16 (m, 22H), 0.91 (t, *J* = 7.5 Hz, 3H); **^{13}C NMR** (101 MHz, $CDCl_3$) δ 176.34, 140.78, 125.01, 91.22, 46.39, 41.68, 41.06, 37.21, 32.97, 31.98, 28.39, 28.31, 28.02, 27.74, 27.18, 27.14, 26.98, 26.87, 26.67, 26.50, 26.21, 26.10, 26.06, 25.72, 23.70, 22.92, 21.92, 8.34; **HRMS (ESI)**: Calculated mass for $C_{19}H_{33}O_2$ $[M+H]^+$: 293.2481; observed mass: 293.2490.

(3a*S*,16a*S*,*E*)-3a,5-Dimethyl-3,3a,4,5,6,7,8,9,10,11,12,13,14,16a-tetradecahydro-2H-cyclopentadeca[b]furan-2-one (3q)

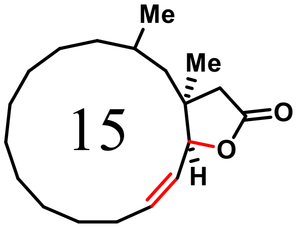

Eluent: ethyl acetate/ petroleum ether (9:91 v/v).

Appearance: colorless gummy.

Isolated yield: 37% (21.5 mg, 0.074 mmol)

^{1}H NMR (400 MHz, $CDCl_3$) δ 5.96 – 5.62 (m, 1H), 5.58 – 5.35 (m, 1H), 4.76 – 4.25 (m, 1H), 2.60 – 2.41 (m, 1H), 2.34 – 2.00 (m, 3H), 1.46 – 1.20 (m, 16H), 1.18 – 1.05 (m, 3H), 1.02 – 0.85 (m, 6H); **^{13}C NMR** (126 MHz, $CDCl_3$) δ 176.79, 138.97, 137.39, 125.11, 124.62, 124.46, 91.81, 90.60, 88.04, 45.02, 43.87, 43.62, 43.47, 42.53, 41.25, 37.54, 36.98, 35.49, 32.26, 31.65, 29.91, 29.26, 28.68, 27.88, 27.78, 27.67, 27.57, 27.50, 27.28, 26.90, 26.84, 26.79, 26.72, 26.61, 26.44, 26.32, 26.23, 26.20, 26.10, 25.99, 25.87, 25.44, 25.06, 24.01, 23.57, 22.45, 22.27, 21.14,

18.52; **HRMS (ESI)**: Calculated mass for $C_{19}H_{33}O_2$ $[M+H]^+$: 293.2481; observed mass: 293.2484.

Ethyl (*E*)-3-((3a*S*,7a*R*)-3a-methyl-2-oxo-3,3a,4,5-tetrahydrobenzofuran-7a(2H)-yl)acrylate (4a)

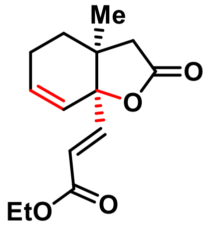

Eluent: ethyl acetate/ petroleum ether (10:90 v/v).

Appearance: colorless liquid.

Isolated yield: 68% (34 mg, 0.136 mmol)

^{1}H NMR (400 MHz, $CDCl_3$) δ 6.88 (d, *J* = 15.7 Hz, 1H), 6.10 (d, *J* = 15.7 Hz, 1H), 6.07 – 5.95 (m, 1H), 5.48 (dt, *J* = 10.1, 2.2 Hz, 1H), 4.21 (q, *J* = 7.1 Hz, 2H), 2.64 (d, *J* = 16.9 Hz, 1H), 2.26 (s, 1H), 2.22 – 2.13 (m, 2H), 1.80 – 1.60 (m, 2H), 1.30 (t, *J* = 7.1 Hz, 3H), 1.07 (s, 3H); **^{13}C NMR** (126 MHz, $CDCl_3$) δ 175.22, 166.02, 144.48, 131.49, 126.19, 122.47, 86.21, 61.00, 41.31, 40.01, 37.99, 29.09, 26.28, 23.71, 22.16, 21.74, 14.39; **HRMS (ESI)**: Calculated mass for $C_{14}H_{19}O_4$ $[M+H]^+$: 251.1283; observed mass: 251.1285.

Ethyl (*E*)-3-((3a*S*,7a*R*)-3a-ethyl-2-oxo-3,3a,4,5-tetrahydrobenzofuran-7a(2H)-yl)acrylate (4b)

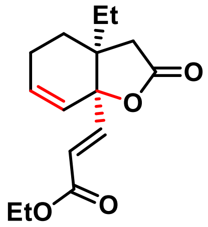

Eluent: ethyl acetate/ petroleum ether (10:90 v/v).

Appearance: colorless liquid.

Isolated yield: 73% (38.5 mg, 0.146 mmol)

^{1}H NMR (400 MHz, $CDCl_3$) δ 6.89 (d, *J* = 15.7 Hz, 1H), 6.10 (d, *J* = 15.8 Hz, 1H), 6.04 (dt, *J* = 10.1, 3.7 Hz, 1H), 5.47 (dt, *J* = 10.0, 2.2 Hz, 1H), 4.21 (q, *J* = 7.1 Hz, 2H), 2.50 (dd, *J* = 17.1, 0.9 Hz, 1H), 2.35 (d, *J* = 17.1 Hz, 1H), 2.16 (dddd, *J* = 8.2, 5.5, 2.7, 1.5 Hz, 2H), 1.77 (dt, *J* = 14.3, 5.2 Hz, 1H), 1.68 – 1.61 (m, 1H), 1.52 – 1.38 (m, 2H), 1.30 (t, *J* = 7.1 Hz, 3H), 0.89 (t, *J* = 7.5 Hz, 3H); **^{13}C NMR** (101 MHz, $CDCl_3$) δ 175.25, 166.02, 144.65, 131.59, 126.39, 122.29,

86.57, 60.98, 44.76, 36.83, 27.93, 25.64, 21.59, 14.41, 8.64; **HRMS (ESI)**: Calculated mass for $C_{15}H_{21}O_4$ $[M+H]^+$: 265.1440; observed mass: 265.1447.

Ethyl (*E*)-3-((3a*S*,7a*R*)-3a-cyclohexyl-2-oxo-3,3a,4,5-tetrahydrobenzofuran-7a(2H)-yl)acrylate (4c)

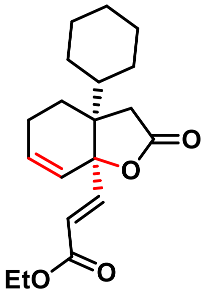

Eluent: ethyl acetate/ petroleum ether (12:88 v/v).

Appearance: colorless gummy.

Isolated yield: 75% (48 mg, 0.15 mmol)

^{1}H NMR (400 MHz, $CDCl_3$) δ 7.03 (d, J = 15.8 Hz, 1H), 6.14 (d, J = 15.7 Hz, 1H), 5.95 (ddd, J = 10.0, 4.5, 2.8 Hz, 1H), 5.37 (ddd, J = 10.0, 2.6, 1.7 Hz, 1H), 4.22 (q, J = 7.2 Hz, 2H), 2.45 (d, J = 17.3 Hz, 1H), 2.33 (d, J = 17.3 Hz, 1H), 2.28 – 2.08 (m, 2H), 1.95 (ddd, J = 14.6, 5.9, 3.3 Hz, 1H), 1.83 – 1.68 (m, 3H), 1.64 – 1.50 (m, 4H), 1.39 (td, J = 8.8, 4.3 Hz, 1H), 1.30 (t, J = 7.2 Hz, 3H), 1.17 – 0.91 (m, 4H); **^{13}C NMR** (101 MHz, $CDCl_3$) δ 175.79, 166.14, 143.97, 129.96, 127.92, 121.80, 87.24, 60.95, 47.11, 44.46, 34.63, 29.90, 28.67, 27.08, 26.84, 26.57, 26.42, 25.11, 22.03, 14.41; **HRMS (ESI)**: Calculated mass for $C_{19}H_{27}O_4$ $[M+H]^+$: 319.1909; observed mass: 319.1914.

Ethyl (*E*)-3-((3a*S*,7a*R*)-2-oxo-3a-propyl-3,3a,4,5-tetrahydrobenzofuran-7a(2H)-yl)acrylate (4d)

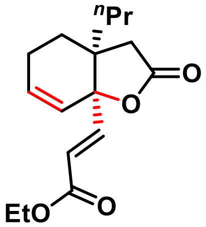

Eluent: ethyl acetate/ petroleum ether (10:90 v/v).

Appearance: colorless gummy.

Isolated yield: 74% (41 mg, 0.148 mmol)

^{1}H NMR (400 MHz, $CDCl_3$) δ 6.89 (d, J = 15.7 Hz, 1H), 6.09 (d, J = 15.7 Hz, 1H), 6.03 (dt, J = 10.0, 3.7 Hz, 1H), 5.46 (dt, J = 10.0, 2.1 Hz, 1H), 4.21 (q, J = 7.1 Hz, 2H), 2.50 (d, J = 17.0 Hz, 1H), 2.36 (d, J = 17.1 Hz, 1H), 2.15 (td, J = 5.4, 2.1 Hz, 2H), 1.75 (dt, J = 14.3, 5.3 Hz,

1H), 1.69 – 1.58 (m, 1H), 1.38 – 1.20 (m, 7H), 0.91 (t, *J* = 6.6 Hz, 3H); **^{13}C NMR** (101 MHz, $CDCl_3$) δ 175.37, 166.04, 144.71, 131.73, 126.17, 122.20, 86.51, 60.98, 44.56, 37.53, 37.47, 26.21, 21.60, 17.67, 14.84, 14.40; **HRMS (ESI)**: Calculated mass for $C_{16}H_{23}O_4$ $[M+H]^+$: 279.1526; observed mass: 279.1530.

Ethyl (*E*)-3-((3a*S*,7a*R*)-3a-butyl-2-oxo-3,3a,4,5-tetrahydrobenzofuran-7a(2H)-yl)acrylate (4e)

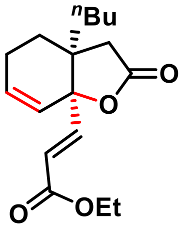

Eluent: ethyl acetate/ petroleum ether (10:90 v/v).

Appearance: colorless gummy.

Isolated yield: 71% (41.5 mg, 0.142 mmol)

^{1}H NMR (400 MHz, $CDCl_3$) δ 6.89 (d, *J* = 15.7 Hz, 1H), 6.08 (d, *J* = 15.7 Hz, 1H), 6.03 (dt, *J* = 10.0, 3.7 Hz, 1H), 5.46 (dt, *J* = 10.1, 2.2 Hz, 1H), 4.21 (q, *J* = 7.1 Hz, 2H), 2.49 (d, *J* = 17.1 Hz, 1H), 2.35 (d, *J* = 17.1 Hz, 1H), 2.15 (tt, *J* = 6.0, 2.1 Hz, 2H), 1.75 (dt, *J* = 14.3, 5.3 Hz, 1H), 1.62 (ddd, *J* = 14.2, 7.8, 6.3 Hz, 1H), 1.46 – 1.19 (m, 9H), 0.88 (t, *J* = 7.1 Hz, 3H); **^{13}C NMR** (101 MHz, $CDCl_3$) δ 175.37, 166.01, 144.72, 131.72, 126.15, 122.20, 86.52, 60.96, 44.47, 37.47, 26.54, 26.20, 23.41, 21.58, 14.43, 14.33, 14.18; **HRMS (ESI)**: Calculated mass for $C_{17}H_{25}O_4$ $[M+H]^+$: 293.1753; observed mass: 293.1760.

Ethyl (*E*)-3-((3a*R*,7a*R*)-3a,4-dimethyl-2-oxo-3,3a,4,5-tetrahydrobenzofuran-7a(2H)-yl)acrylate (4f)

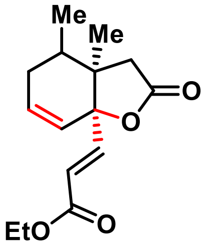

Eluent: ethyl acetate/ petroleum ether (10:90 v/v).

Appearance: colorless liquid.

Isolated yield: 64% (34 mg, 0.128 mmol) (d.r. = 6:1)

^{1}H NMR (500 MHz, $CDCl_3$) δ 6.87 (d, *J* = 15.7 Hz, 1H), 6.10 (d, *J* = 15.8 Hz, 1H), 5.94 (ddd, *J* = 10.2, 5.1, 1.8 Hz, 1H), 5.51 – 5.38 (m, 1H), 4.20 (q, *J* = 7.2 Hz, 2H), 2.61 (d, *J* = 16.7 Hz, 1H), 2.27 – 2.17 (m, 1H), 2.08 (d, *J* = 16.8 Hz, 1H), 1.96 – 1.80 (m, 2H), 1.29 (t, *J* = 7.1 Hz,

3H), 1.07 (s, 3H), 0.96 (d, *J* = 6.4 Hz, 3H); **^{13}C NMR** (126 MHz, $CDCl_3$) δ 175.20, 166.06, 144.09, 141.72, 130.34, 126.87, 122.98, 122.38, 87.55, 60.95, 45.50, 36.47, 32.47, 30.74, 21.91, 16.93, 14.40; **HRMS (ESI)**: Calculated mass for $C_{15}H_{21}O_4$ $[M+H]^+$: 265.1440; observed mass: 265.1445.

Ethyl (*E*)-3-((3a*S*,7a*R*)-3a,5-dimethyl-2-oxo-3,3a,4,5-tetrahydrobenzofuran-7a(2H)-yl)acrylate (4g)

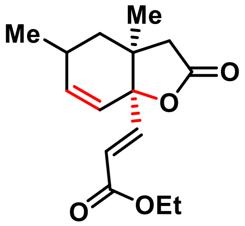

Eluent: ethyl acetate/ petroleum ether (10:90 v/v).

Appearance: colorless gummy.

Isolated yield: 67% (35.5 mg, 0.134 mmol) (d.r. = 5:1)

^{1}H NMR (400 MHz, $CDCl_3$) δ 6.85 (d, *J* = 15.7 Hz, 1H), 6.07 (d, *J* = 15.7 Hz, 1H), 5.82 (dt, *J* = 10.2, 1.7 Hz, 1H), 5.40 (dd, *J* = 10.0, 2.7 Hz, 1H), 4.20 (q, *J* = 7.1 Hz, 2H), 2.75 (d, *J* = 16.9 Hz, 1H), 2.36 (ddd, *J* = 10.3, 5.3, 2.6 Hz, 1H), 2.11 (d, *J* = 16.9 Hz, 1H), 1.82 (ddd, *J* = 14.6, 5.5, 1.3 Hz, 1H), 1.36 – 1.25 (m, 4H), 1.07 (d, *J* = 7.0 Hz, 3H), 1.04 – 1.01 (m, 3H); **^{13}C NMR** (101 MHz, $CDCl_3$) δ 175.15, 166.01, 143.97, 136.75, 125.77, 122.51, 86.70, 60.97, 42.09, 39.63, 37.66, 27.49, 24.77, 21.07, 14.38; **HRMS (ESI)**: Calculated mass for $C_{15}H_{21}O_4$ $[M+H]^+$: 265.1440; observed mass: 265.1442.

Ethyl (*E*)-3-((3a*S*,7a*R*)-3a-isobutyl-5-methyl-2-oxo-3,3a,4,5-tetrahydrobenzofuran-7a(2H)-yl)acrylate (4h)

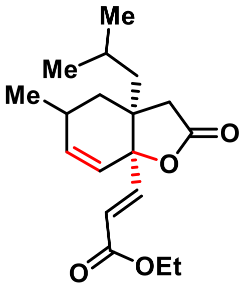

Eluent: ethyl acetate/ petroleum ether (10:90 v/v).

Appearance: colorless gummy.

Isolated yield: 79% (48.5 mg, 0.158 mmol)

^{1}H NMR (400 MHz, $CDCl_3$) δ 6.84 (d, *J* = 15.7 Hz, 1H), 6.07 (d, *J* = 15.7 Hz, 1H), 5.81 (dt, *J* = 10.0, 1.6 Hz, 1H), 5.35 (dd, *J* = 10.0, 2.6 Hz, 1H), 4.22 (q, *J* = 7.1 Hz, 2H), 2.62 (dd, *J* =

17.1, 1.9 Hz, 1H), 2.39 (d, J = 17.1 Hz, 1H), 2.32 (dtd, J = 9.9, 6.1, 5.2, 2.5 Hz, 1H), 2.02 (ddd, J = 14.5, 5.4, 1.3 Hz, 1H), 1.66 – 1.52 (m, 1H), 1.40 – 1.32 (m, 1H), 1.30 (d, J = 7.2 Hz, 3H), 1.20 – 1.11 (m, 2H), 1.08 (d, J = 7.0 Hz, 3H), 0.94 (dd, J = 6.7, 4.8 Hz, 6H); **^{13}C NMR** (101 MHz, $CDCl_3$) δ 175.24, 166.12, 143.95, 136.40, 126.03, 122.26, 87.34, 60.98, 46.15, 44.28, 36.11, 35.05, 27.36, 25.74, 25.54, 24.48, 21.22, 14.41; **HRMS (ESI)**: Calculated mass for $C_{18}H_{26}NaO_4$ $[M+Na]^+$: 329.1729; observed mass: 329.1725.

Methyl (*E*)-3-((3a*S*,7a*R*)-3a-methyl-2-oxo-3,3a,4,5-tetrahydrobenzofuran-7a(2H)-yl)acrylate (4i)

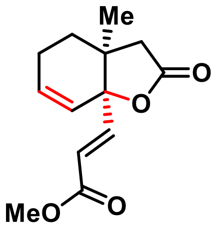

Eluent: ethyl acetate/ petroleum ether (10:90 v/v).

Appearance: colorless gummy.

Isolated yield: 62% (29.5 mg, 0.124 mmol)

^{1}H NMR (400 MHz, $CDCl_3$) δ 6.88 (d, J = 15.8 Hz, 1H), 6.11 (d, J = 15.8 Hz, 1H), 6.06 – 5.99 (m, 1H), 5.47 (dt, J = 10.1, 2.2 Hz, 1H), 3.75 (s, 3H), 2.64 (d, J = 16.9 Hz, 1H), 2.22 – 2.13 (m, 3H), 1.87 – 1.55 (m, 2H), 1.06 (s, 3H); **^{13}C NMR** (126 MHz, $CDCl_3$) δ 175.17, 166.46, 144.74, 131.53, 126.13, 121.93, 86.17, 52.07, 41.28, 39.96, 37.98, 33.44, 29.06, 26.27, 23.73, 22.15, 21.73; **HRMS (ESI)**: Calculated mass for $C_{13}H_{16}NaO_4$ $[M+Na]^+$: 259.0946; observed mass: 259.0950.

Butyl (*E*)-3-((3a*S*,7a*R*)-2-oxo-3a-propyl-3,3a,4,5-tetrahydrobenzofuran-7a(2H)-yl)acrylate (4j)

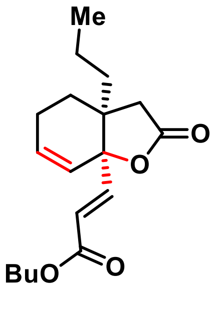

Eluent: ethyl acetate/ petroleum ether (10:90 v/v).

Appearance: colorless gummy.

Isolated yield: 78% (48 mg, 0.156 mmol)

^{1}H NMR (400 MHz, $CDCl_3$) δ 6.89 (d, J = 15.7 Hz, 1H), 6.10 (d, J = 15.7 Hz, 1H), 6.04 (dt, J = 10.0, 3.7 Hz, 1H), 5.47 (dt, J = 10.0, 2.2 Hz, 1H), 4.16 (t, J = 6.7 Hz, 2H), 2.50 (d, J = 17.1

Hz, 1H), 2.36 (d, J = 17.0 Hz, 1H), 2.15 (ddt, J = 5.6, 3.8, 2.0 Hz, 2H), 1.82 – 1.71 (m, 1H), 1.69 – 1.59 (m, 3H), 1.48 – 1.31 (m, 5H), 1.33 – 1.18 (m, 1H), 1.00 – 0.85 (m, 6H); **^{13}C NMR** (101 MHz, $CDCl_3$) δ 175.40, 166.15, 144.71, 131.75, 126.18, 122.22, 86.52, 64.91, 44.57, 37.56, 37.50, 30.82, 26.24, 21.61, 19.36, 17.69, 14.84, 13.92; **HRMS (ESI)**: Calculated mass for $C_{18}H_{27}O_4$ $[M+H]^+$: 307.1909; observed mass: 307.1912.

7-(l2-Fluoraneyl)-7,7,7,7,7,7,7,7,7,7,7-undecafluoro-7l15-hepta-2,4,6-triyn-1-yl (*E*)-3-((3a*S*,7a*R*)-2-oxo-3a-propyl-3,3a,4,5-tetrahydrobenzofuran-7a(2H)-yl)acrylate (4k)

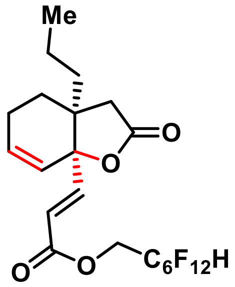

Eluent: ethyl acetate/ petroleum ether (12:88 v/v).

Appearance: colorless gummy.

Isolated yield: 60% (68 mg, 0.12 mmol)

^{1}H NMR (400 MHz, $CDCl_3$) δ 7.00 (d, J = 15.7 Hz, 1H), 6.17 (d, J = 15.7 Hz, 1H), 6.07 (ddd, J = 10.1, 4.5, 2.7 Hz, 2H), 5.45 (dt, J = 10.0, 2.2 Hz, 1H), 4.67 (td, J = 13.4, 2.1 Hz, 2H), 2.52 (d, J = 17.1 Hz, 1H), 2.36 (d, J = 17.1 Hz, 1H), 2.17 (ddd, J = 9.0, 4.0, 1.9 Hz, 2H), 1.78 (dt, J = 14.4, 5.2 Hz, 1H), 1.68 – 1.56 (m, 1H), 1.42 – 1.14 (m, 4H), 0.93 – 0.83 (m, 3H); **^{13}C NMR** (101 MHz, $CDCl_3$) δ 175.05, 164.17, 147.59, 132.15, 125.75, 119.98, 110.30, 108.08, 107.77, 105.23, 86.34, 60.25, 59.98, 59.71, 44.61, 37.75, 37.28, 26.23, 21.58, 17.60, 14.69; **^{19}F NMR** (376 MHz, $CDCl_3$) δ -119.37, -119.42, -119.46, -122.10, -122.14, -122.19, -122.23, -122.28, -123.32, -123.37, -123.42, -123.52, -129.45, -129.45, -129.51, -137.07, -137.07; **HRMS (ESI)**: Calculated mass for $C_{21}H_{21}F_{12}O_4$ $[M+H]^+$: 565.1248; observed mass: 565.1250.

Benzyl (*E*)-3-((3a*S*,7a*R*)-2-oxo-3a-propyl-3,3a,4,5-tetrahydrobenzofuran-7a(2H)-yl)acrylate (4l)

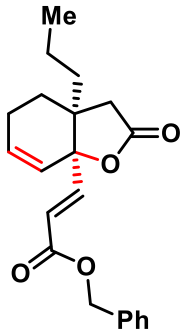

Eluent: ethyl acetate/ petroleum ether (15:85 v/v).

Appearance: colorless gummy.

Isolated yield: 67% (45.5 mg, 0.134 mmol)

^{1}H NMR (400 MHz, $CDCl_3$) δ 7.51 – 7.30 (m, 5H), 6.95 (d, *J* = 15.7 Hz, 1H), 6.15 (d, *J* = 15.7 Hz, 1H), 6.03 (dt, *J* = 10.0, 3.7 Hz, 1H), 5.56 – 5.41 (m, 1H), 5.21 (s, 2H), 2.50 (d, *J* = 17.0 Hz, 1H), 2.36 (d, *J* = 17.0 Hz, 1H), 2.15 (ddd, *J* = 5.8, 4.5, 2.3 Hz, 2H), 1.75 (dt, *J* = 14.3, 5.3 Hz, 1H), 1.62 (ddd, *J* = 14.2, 7.8, 6.3 Hz, 1H), 1.43 – 1.29 (m, 2H), 1.27 – 1.15 (m, 2H), 1.03 – 0.86 (m, 3H); **^{13}C NMR** (101 MHz, $CDCl_3$) δ 175.30, 165.81, 145.40, 135.87, 131.80, 128.81, 128.55, 128.45, 126.10, 121.90, 86.49, 66.78, 44.59, 37.51, 37.49, 26.20, 21.59, 17.67, 14.84; **HRMS (ESI)**: Calculated mass for $C_{21}H_{25}O_4$ $[M+H]^+$: 341.1753; observed mass: 341.1760.

2-Fluorobenzyl (*E*)-3-((3a*S*,7a*R*)-3a-ethyl-2-oxo-3,3a,4,5-tetrahydrobenzofuran-7a(2H)-yl)acrylate (4m)

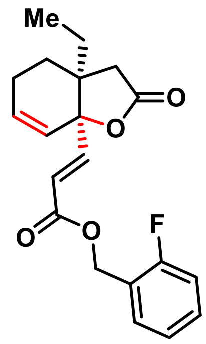

Eluent: ethyl acetate/ petroleum ether (15:85 v/v).

Appearance: colorless gummy.

Isolated yield: 69% (47.5 mg, 0.138 mmol)

^{1}H NMR (400 MHz, $CDCl_3$) δ 7.40 (td, *J* = 7.5, 1.9 Hz, 1H), 7.33 (tdd, *J* = 7.5, 5.3, 1.8 Hz, 1H), 7.15 (td, *J* = 7.5, 1.2 Hz, 1H), 7.08 (ddd, *J* = 9.7, 8.3, 1.2 Hz, 1H), 6.94 (d, *J* = 15.7 Hz, 1H), 6.14 (d, *J* = 15.7 Hz, 1H), 6.03 (dt, *J* = 10.0, 3.7 Hz, 1H), 5.46 (dt, *J* = 10.0, 2.1 Hz, 1H), 5.26 (d, *J* = 1.2 Hz, 2H), 2.50 (dd, *J* = 17.1, 0.8 Hz, 1H), 2.34 (d, *J* = 17.1 Hz, 1H), 2.16 (tt, *J* = 5.6, 2.1 Hz, 2H), 1.77 (dt, *J* = 14.4, 5.2 Hz, 1H), 1.66 – 1.54 (m, 1H), 1.54 – 1.34 (m, 2H), 0.88 (t, *J* = 7.5 Hz, 3H); **^{13}C NMR** (101 MHz, $CDCl_3$) δ 175.22, 165.67, 162.17, 159.97, 145.53, 131.71, 130.87, 130.83, 130.59, 130.51, 126.19, 124.42, 124.38, 123.12, 122.98, 121.70, 115.83, 115.62, 86.55, 60.72, 60.67, 44.77, 36.73, 27.91, 25.55, 21.54, 8.62; **^{19}F NMR** (376 MHz, $CDCl_3$) δ -117.90; **HRMS (ESI)**: Calculated mass for $C_{20}H_{21}FNaO_4$ $[M+Na]^+$: 367.1322; observed mass: 367.1325.

4-Chlorobenzyl (*E*)-3-((3a*S*,7a*R*)-3a-ethyl-2-oxo-3,3a,4,5-tetrahydrobenzofuran-7a(2H)-yl)acrylate (4n)

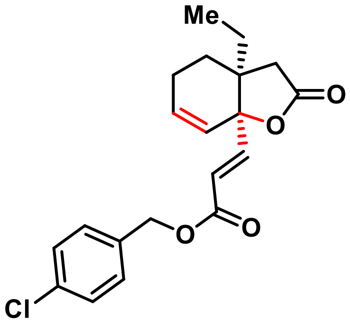

Eluent: ethyl acetate/ petroleum ether (15:85 v/v).

Appearance: colorless gummy.

Isolated yield: 66% (48 mg, 0.132 mmol)

^{1}H NMR (400 MHz, $CDCl_3$) δ 7.39 – 7.28 (m, 4H), 6.93 (d, *J* = 15.7 Hz, 1H), 6.14 (d, *J* = 15.7 Hz, 1H), 6.04 (d, *J* = 10.0 Hz, 1H), 5.46 (d, *J* = 10.0 Hz, 1H), 5.15 (s, 2H), 2.50 (dd, *J* = 17.1, 0.9 Hz, 1H), 2.34 (d, *J* = 17.1 Hz, 1H), 2.16 (ddd, *J* = 7.7, 4.2, 2.1 Hz, 2H), 1.77 (dt, *J* = 14.4, 5.1 Hz, 1H), 1.61 – 1.53 (m, 1H), 1.53 – 1.33 (m, 2H), 0.88 (t, *J* = 7.4 Hz, 3H); **^{13}C NMR** (101 MHz, $CDCl_3$) δ 175.12, 165.68, 145.58, 134.50, 134.44, 131.73, 129.87, 129.02, 128.89, 126.24, 121.69, 86.52, 65.94, 44.77, 36.76, 27.96, 25.62, 21.57, 8.62; **HRMS (ESI)**: Calculated mass for $C_{20}H_{22}ClO_4$ $[M+H]^+$: 361.1207; observed mass: 361.1210.

3-Methylbenzyl (*E*)-3-((3a*S*,7a*R*)-3a-ethyl-2-oxo-3,3a,4,5-tetrahydrobenzofuran-7a(2H)-yl)acrylate (4o)

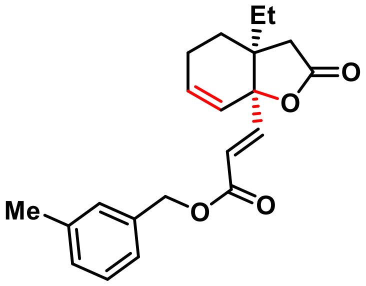

Eluent: ethyl acetate/ petroleum ether (9:91 v/v).

Appearance: colorless gummy.

Isolated yield: 68% (46.2 mg, 0.136 mmol)

^{1}H NMR (500 MHz, $CDCl_3$) δ 7.37 – 7.21 (m, 1H), 7.22 – 7.10 (m, 3H), 6.94 (d, *J* = 15.7 Hz, 1H), 6.15 (d, *J* = 15.7 Hz, 1H), 6.03 (dt, *J* = 10.0, 3.7 Hz, 1H), 5.46 (dt, *J* = 10.1, 2.2 Hz, 1H), 5.16 (s, 2H), 2.50 (d, *J* = 17.1 Hz, 1H), 2.37 (s, 3H), 2.34 (d, *J* = 10.8 Hz, 1H), 2.16 (ddt, *J* = 8.5, 6.0, 2.7 Hz, 2H), 1.76 (dt, *J* = 14.4, 5.2 Hz, 1H), 1.62 – 1.55 (m, 1H), 1.44 (dp, *J* = 29.2, 7.1 Hz, 2H), 0.88 (t, *J* = 7.5 Hz, 3H); **^{13}C NMR** (126 MHz, $CDCl_3$) δ 175.25, 165.83, 145.26,

138.54, 135.77, 131.67, 129.31, 129.23, 128.72, 126.25, 125.55, 121.99, 86.58, 66.85, 44.78, 36.77, 27.91, 25.57, 21.55, 8.63; **HRMS (ESI)**: Calculated mass for $C_{21}H_{25}O_4$ $[M+H]^+$: 341.1753; observed mass: 341.1760.

(3a*S*,7a*R*)-7a-((*E*)-3-Oxobut-1-en-1-yl)-3a-propyl-3a,4,5,7a-tetrahydrobenzofuran-2(3H)-one (4p)

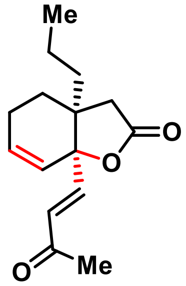

Eluent: ethyl acetate/ petroleum ether (20:80 v/v).

Appearance: colorless gummy.

Isolated yield: 67% (33.5 mg, 0.135 mmol)

^{1}H NMR (400 MHz, $CDCl_3$) δ 6.74 (d, *J* = 15.9 Hz, 1H), 6.37 (d, *J* = 15.9 Hz, 1H), 6.04 (dt, *J* = 10.0, 3.7 Hz, 1H), 5.45 (dt, *J* = 10.0, 2.2 Hz, 1H), 2.52 (d, *J* = 17.1 Hz, 1H), 2.36 (d, *J* = 17.1 Hz, 1H), 2.29 (s, 3H), 2.16 (ddd, *J* = 8.5, 4.3, 2.3 Hz, 2H), 1.77 (dt, *J* = 14.4, 5.2 Hz, 1H), 1.68 – 1.55 (m, 1H), 1.38 – 1.30 (m, 4H), 0.96 – 0.87 (m, 3H); **^{13}C NMR** (101 MHz, $CDCl_3$) δ 197.46, 175.33, 142.96, 131.74, 129.80, 126.17, 86.56, 44.67, 37.70, 37.36, 28.87, 26.18, 21.59, 17.63, 14.85; **HRMS (ESI)**: Calculated mass for $C_{15}H_{21}O_3$ $[M+H]^+$: 249.1491; observed mass: 249.1495.

(3a*S*,7a*R*)-3a-Methyl-7a-((*E*)-3-oxobut-1-en-1-yl)-3a,4,5,7a-tetrahydrobenzofuran-2(3H)-one (4q)

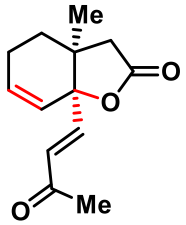

Eluent: ethyl acetate/ petroleum ether (15:85 v/v).

Appearance: colorless gummy.

Isolated yield: 65% (28.5 mg, 0.130 mmol)

^{1}H NMR (400 MHz, $CDCl_3$) δ 6.72 (d, *J* = 15.9 Hz, 1H), 6.36 (d, *J* = 15.9 Hz, 1H), 6.04 (dt, *J* = 10.0, 3.7 Hz, 1H), 5.61 – 5.40 (m, 1H), 2.67 (d, *J* = 16.9 Hz, 1H), 2.29 (s, 3H), 2.23 – 2.11 (m, 3H), 1.78 – 1.64 (m, 2H), 1.07 (s, 3H); **^{13}C NMR** (101 MHz, $CDCl_3$) δ 197.53, 175.11,

142.82, 131.56, 130.21, 126.16, 86.24, 41.43, 39.91, 29.01, 28.65, 23.82, 21.74; **HRMS (ESI)**: Calculated mass for $C_{13}H_{17}O_3$ $[M+H]^+$: 221.1178; observed mass: 221.1185.

(3a*S*,7a*R*)-3a-Cyclohexyl-7a-((*E*)-3-oxobut-1-en-1-yl)-3a,4,5,7a-tetrahydrobenzofuran-2(3H)-one (4r)

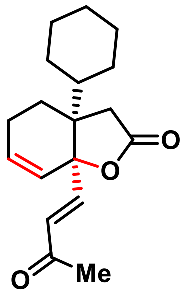

Eluent: ethyl acetate/ petroleum ether (10:90 v/v).

Appearance: colorless gummy.

Isolated yield: 68% (39.5 mg, 0.136 mmol)

^{1}H NMR (400 MHz, $CDCl_3$) δ 6.88 (d, *J* = 15.8 Hz, 1H), 6.42 (d, *J* = 15.9 Hz, 1H), 5.96 (ddd, *J* = 10.0, 4.6, 2.8 Hz, 1H), 5.36 (dt, *J* = 10.1, 2.1 Hz, 1H), 2.47 (d, *J* = 17.4 Hz, 1H), 2.34 (d, *J* = 17.4 Hz, 1H), 2.30 (s, 3H), 2.25 – 2.09 (m, 2H), 1.98 (ddd, *J* = 14.5, 5.8, 3.1 Hz, 1H), 1.79 (d, *J* = 11.0 Hz, 2H), 1.56 – 1.31 (m, 5H), 1.14 – 0.80 (m, 5H); **^{13}C NMR** (101 MHz, $CDCl_3$) δ 197.50, 175.78, 142.30, 130.01, 129.46, 127.84, 87.31, 53.64, 47.23, 44.58, 34.37, 28.80, 28.72, 27.01, 26.83, 26.61, 26.39, 25.12, 21.95; **HRMS (ESI)**: Calculated mass for $C_{18}H_{25}O_3$ $[M+H]^+$: 289.1804; observed mass: 289.1810.

(3a*S*,7a*S*)-6-(*tert*-Butyl)-3a-methyl-7a-((*E*)-3-oxobut-1-en-1-yl)-3a,4,5,7a-tetrahydrobenzofuran-2(3H)-one (4s)

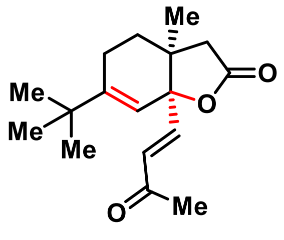

Eluent: ethyl acetate/ petroleum ether (15:85 v/v).

Appearance: colorless liquid.

Isolated yield: 35% (19.5 mg, 0.07 mmol)

^{1}H NMR (400 MHz, $CDCl_3$) δ 6.62 (d, *J* = 15.9 Hz, 1H), 6.32 (d, *J* = 15.9 Hz, 1H), 5.43 (s, 1H), 2.29 (s, 3H), 2.28 – 2.10 (m, 3H), 1.98 (dd, *J* = 15.2, 2.4 Hz, 1H), 1.78 (dd, *J* = 15.2, 4.5 Hz, 2H), 1.18 (s, 9H), 1.02 (s, 3H); **^{13}C NMR** (101 MHz, $CDCl_3$) δ 197.65, 175.60, 150.27,

142.25, 129.83, 121.69, 87.14, 63.70, 41.61, 39.33, 39.05, 35.83, 30.44, 28.79, 24.94; **HRMS (ESI)**: Calculated mass for $C_{17}H_{25}O_3$ $[M+H]^+$: 277.1804; observed mass: 277.1810.

(3a*R*,7a*R*)-3a,4-Dimethyl-7a-((*E*)-3-oxobut-1-en-1-yl)-3a,4,5,7a-tetrahydrobenzofuran-2(3H)-one (4t)

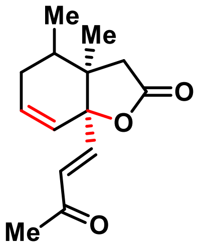

Eluent: ethyl acetate/ petroleum ether (8:92 v/v).

Appearance: colorless gummy.

Isolated yield: 57% (26.5 mg, 0.114 mmol)

^{1}H NMR (500 MHz, $CDCl_3$) δ 6.72 (d, *J* = 15.9 Hz, 1H), 6.37 (d, *J* = 15.9 Hz, 1H), 5.96 (ddd, *J* = 10.1, 5.1, 1.9 Hz, 1H), 5.43 (dt, *J* = 10.2, 2.2 Hz, 1H), 2.62 (d, *J* = 16.8 Hz, 1H), 2.28 (s, 3H), 2.26 – 2.19 (m, 1H), 2.10 (d, *J* = 16.8 Hz, 1H), 1.97 – 1.83 (m, 2H), 1.07 (s, 3H), 0.96 (d, *J* = 6.1 Hz, 3H); **^{13}C NMR** (126 MHz, $CDCl_3$) δ 197.63, 175.14, 142.52, 130.52, 130.21, 126.79, 87.56, 45.64, 36.48, 32.52, 30.76, 28.59, 21.92, 16.92; **HRMS (ESI)**: Calculated mass for $C_{14}H_{19}O_3$ $[M+H]^+$: 235.1334; observed mass: 235.1339.

(3a*S*,7a*R*)-3a,5-Dimethyl-7a-((*E*)-3-oxobut-1-en-1-yl)-3a,4,5,7a-tetrahydrobenzofuran-2(3H)-one (4u)

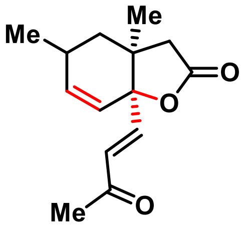

Eluent: ethyl acetate/ petroleum ether (10:90 v/v).

Appearance: colorless gummy.

Isolated yield: 49% (23 mg, 0.098 mmol)

^{1}H NMR (400 MHz, $CDCl_3$) δ 6.71 (d, *J* = 15.9 Hz, 1H), 6.35 (d, *J* = 15.9 Hz, 1H), 5.91 – 5.80 (m, 1H), 5.41 (dd, *J* = 10.1, 2.7 Hz, 1H), 2.77 (d, *J* = 16.9 Hz, 1H), 2.43 – 2.33 (m, 1H), 2.30 (s, 3H), 2.13 (d, *J* = 16.8 Hz, 1H), 1.84 (ddd, *J* = 14.5, 5.5, 1.3 Hz, 1H), 1.36 – 1.26 (m, 1H), 1.09 (d, *J* = 7.0 Hz, 3H), 1.04 (s, 3H); **^{13}C NMR** (126 MHz, $CDCl_3$) δ 197.63, 175.11, 142.44,

136.94, 130.35, 125.73, 86.75, 42.24, 39.68, 37.72, 29.92, 28.61, 27.54, 24.80, 21.09; **HRMS (ESI)**: Calculated mass for $C_{14}H_{19}O_3$ $[M+H]^+$: 235.1334; observed mass: 235.1330.

(3a*S*,7a*R*)-3a-Methyl-7a-((*E*)-3-oxopent-1-en-1-yl)-3a,4,5,7a-tetrahydrobenzofuran-2(3H)-one (4v)

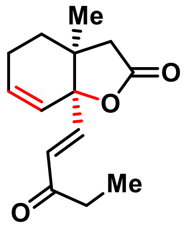

Eluent: ethyl acetate/ petroleum ether (16:84 v/v).

Appearance: colorless gummy.

Isolated yield: 66% (31 mg, 0.132 mmol)

^{1}H NMR (500 MHz, $CDCl_3$) δ 6.75 (d, *J* = 15.8 Hz, 1H), 6.40 (d, *J* = 15.8 Hz, 1H), 6.03 (dt, *J* = 10.1, 3.7 Hz, 1H), 5.47 (dt, *J* = 10.0, 2.3 Hz, 1H), 2.66 (d, *J* = 16.9 Hz, 1H), 2.59 (q, *J* = 7.3 Hz, 2H), 2.19 (dt, *J* = 14.2, 5.6 Hz, 3H), 1.80 – 1.58 (m, 2H), 1.12 (d, *J* = 7.3 Hz, 3H), 1.06 (s, 3H); **^{13}C NMR** (126 MHz, $CDCl_3$) δ 200.18, 175.23, 141.63, 131.44, 129.00, 126.26, 86.37, 41.36, 39.98, 37.99, 35.24, 29.07, 23.80, 22.16, 21.75, 8.04; **HRMS (ESI)**: Calculated mass for $C_{14}H_{19}O_3$ $[M+H]^+$: 235.1334; observed mass: 235.1340.

(*E*)-3-((3a*S*,7a*R*)-3a-Methyl-2-oxo-3,3a,4,5-tetrahydrobenzofuran-7a(2H)-yl)acrylaldehyde (4w)

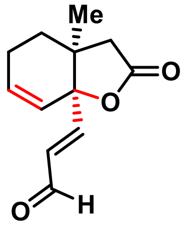

Eluent: ethyl acetate/ petroleum ether (15:85 v/v).

Appearance: colorless gummy.

Isolated yield: 47% (19.5 mg, 0.094 mmol) (d.r. = 3:1)

^{1}H NMR (400 MHz, $CDCl_3$) δ 9.62 (dd, *J* = 7.6, 1.0 Hz, 1H), 6.75 (dd, *J* = 15.8, 1.0 Hz, 1H), 6.35 (ddd, *J* = 15.8, 7.5, 1.2 Hz, 1H), 6.08 (dt, *J* = 10.1, 3.7 Hz, 1H), 5.52 (ddd, *J* = 10.1, 2.8, 1.6 Hz, 1H), 2.69 (d, *J* = 16.8 Hz, 1H), 2.28 – 2.13 (m, 3H), 1.82 – 1.73 (m, 2H), 1.10 (s, 3H); **^{13}C NMR** (101 MHz, $CDCl_3$) δ 192.45, 174.71, 152.40, 132.72, 131.94, 131.19, 126.58, 125.83, 86.07, 77.43, 41.57, 40.92, 40.81, 39.83, 38.99, 31.79, 29.91, 29.71, 29.03, 27.09,

23.89, 22.69, 21.80, 21.75, 14.30; **HRMS (ESI)**: Calculated mass for $C_{12}H_{15}O_3$ $[M+H]^+$: 207.1021; observed mass: 207.1025.

(*E*)-3-((3a*S*,7a*R*)-3a-Cyclohexyl-2-oxo-3,3a,4,5-tetrahydrobenzofuran-7a(2H)-yl)acrylaldehyde (4x)

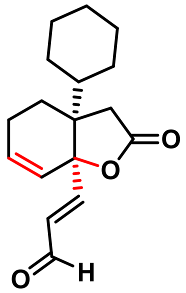

Eluent: ethyl acetate/ petroleum ether (15:85 v/v).

Appearance: colorless gummy.

Isolated yield: 40% (22 mg, 0.08 mmol)

^{1}H NMR (400 MHz, $CDCl_3$) δ 9.63 (d, *J* = 7.6 Hz, 1H), 6.90 (d, *J* = 15.8 Hz, 1H), 6.41 (dd, *J* = 15.8, 7.6 Hz, 1H), 6.05 – 5.94 (m, 1H), 5.47 – 5.33 (m, 1H), 2.49 (d, *J* = 17.4 Hz, 1H), 2.41 – 2.27 (m, 3H), 2.03 (ddd, *J* = 15.2, 5.8, 3.2 Hz, 2H), 1.81 (d, *J* = 11.4 Hz, 4H), 1.15 – 0.94 (m, 7H); **^{13}C NMR** (126 MHz, $CDCl_3$) δ 192.58, 151.98, 132.06, 130.36, 127.45, 87.19, 47.40, 44.65, 34.10, 32.15, 29.92, 29.88, 29.58, 28.70, 26.93, 26.81, 26.54, 26.32, 25.18, 22.91, 21.91, 14.34; **HRMS (ESI)**: Calculated mass for $C_{17}H_{22}NaO_3$ $[M+Na]^+$: 297.1467; observed mass: 297.1470.

(3a*S*,7a*R*)-3a-Methyl-7a-((*E*)-3-oxooct-1-en-1-yl)-3a,4,5,7a-tetrahydrobenzofuran-2(3H)-one (4y)

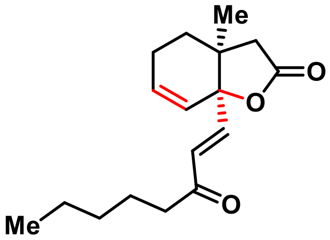

Eluent: ethyl acetate/ petroleum ether (15:85 v/v).

Appearance: colorless gummy.

Isolated yield: 64% (35.5 mg, 0.128 mmol) (d.r. = 10:1)

^{1}H NMR (400 MHz, $CDCl_3$) δ 6.73 (d, *J* = 15.8 Hz, 1H), 6.39 (d, *J* = 15.8 Hz, 1H), 6.03 (dt, *J* = 10.1, 3.7 Hz, 1H), 5.47 (dt, *J* = 10.1, 2.2 Hz, 1H), 2.66 (d, *J* = 16.9 Hz, 1H), 2.61 – 2.50 (m,

2H), 2.24 – 2.08 (m, 3H), 1.76 – 1.64 (m, 2H), 1.30 (ddd, *J* = 7.3, 5.5, 3.5 Hz, 6H), 1.06 (s, 3H), 0.99 – 0.83 (m, 3H); **^{13}C NMR** (101 MHz, $CDCl_3$) δ 199.95, 175.19, 141.72, 131.42, 129.29, 126.30, 86.38, 42.03, 41.37, 40.00, 31.60, 29.10, 23.88, 23.80, 22.65, 21.77, 14.12; **HRMS (ESI)**: Calculated mass for $C_{17}H_{25}O_3$ $[M+H]^+$: 277.1804; observed mass: 277.1807.

(3a*S*,7a*S*)-6-(*tert*-Butyl)-7a-((*E*)-3-hydroxy-3-methylbut-1-en-1-yl)-3a-methyl-3a,4,5,7a-tetrahydrobenzofuran-2(3H)-one (4z)

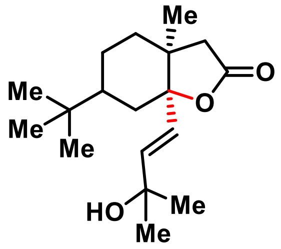

Eluent: ethyl acetate/ petroleum ether (15:85 v/v).

Appearance: colorless gummy.

Isolated yield: 55% (32.2 mg, 0.110 mmol) (d.r. = 3:1)

^{1}H NMR (400 MHz, $CDCl_3$) δ 6.02 (d, *J* = 15.6 Hz, 1H), 5.83 (d, *J* = 15.7 Hz, 1H), 2.84 (d, *J* = 17.1 Hz, 1H), 2.13 – 1.91 (m, 2H), 1.77 – 1.58 (m, 6H), 1.33 (dd, *J* = 8.1, 1.9 Hz, 6H), 0.94 (s, 3H), 0.86 (s, 9H); **^{13}C NMR** (101 MHz, $CDCl_3$) δ 176.18, 142.87, 138.22, 122.73, 120.51, 89.18, 71.09, 47.97, 44.54, 41.36, 40.49, 39.17, 38.02, 35.61, 33.40, 32.58, 32.45, 30.40, 30.29, 30.04, 27.63, 27.61, 27.50, 25.83, 24.26, 23.38, 22.60; **HRMS (ESI)**: Calculated mass for $C_{18}H_{31}O_3$ $[M+H]^+$: 295.2273; observed mass: 295.2276.

Procedure for the Hydrogenation of Unsaturated Bicyclic Lactones:

In an oven-dried round bottom flask was charged with magnetic stir-bar, corresponding unsaturated bicyclic lactone (1.0 equiv.), Pd/C (10 mol%), in MeOH and then H_2 balloon was purged through the reaction. The reaction mixture was stirred for 2-3 h at room temperature. Upon completion the mixture was diluted with EtOAc and filtered through a celite pad. The filtrate was evaporated under reduced pressure and dried under high vacuum yielding saturated lactones in almost quantitative yield. The resulting saturated lactone was characterized by NMR and HRMS analysis.

Procedure for the Reduction of Unsaturated Bicyclic Lactone 2a with $LiAlH_4$:

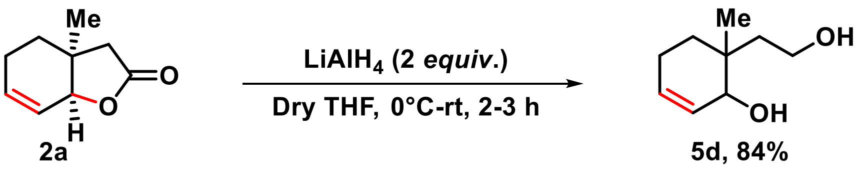

In an oven-dried screw capped reaction tube was charged with magnetic stir-bar, unsaturated bicyclic lactone **2a** (1.0 equiv.) and then dry THF (2 mL) was added. Then $LiAlH_4$ (2.0 equiv.) was added to it under ice cold condition and the reaction was stirred for 2 h at room temperature. Upon completion the reaction was quenched with NH_4Cl solution and then was diluted with EtOAc and filtered through a celite pad. The filtrate was evaporated under reduced pressure and the crude mixture was purified by column chromatography using silica (100-200 mesh size) and petroleum ether/ ethyl acetate as the eluent to yield diol product in 84% (**5c**).

Procedure for the Dihydroxylation of Unsaturated Bicyclic Lactone 2a with $KMnO_4$:

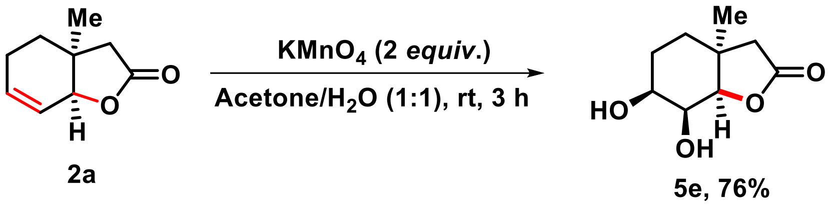

In an oven-dried screw capped reaction tube was charged with magnetic stir-bar, unsaturated bicyclic lactone **2a** (1.0 equiv.), $KMnO_4$ (2.0 equiv.) then acetone and water (2 mL, 1:1) was added to it and the reaction was stirred for 3 h at room temperature. Upon completion the solvent was removed under reduce pressure and diluted with EtOAc. The organic layer was washed with brine solution two times and dried on anhydrous sodium sulfate. The solvent was evaporated under reduced pressure and the crude mixture was purified by column

chromatography using silica (100-200 mesh size) and petroleum ether/ ethyl acetate as the eluent to yield dihydroxylated product in 76% (**5d**).

Procedure for the Allylic Oxidation of Unsaturated Bicyclic Lactone:

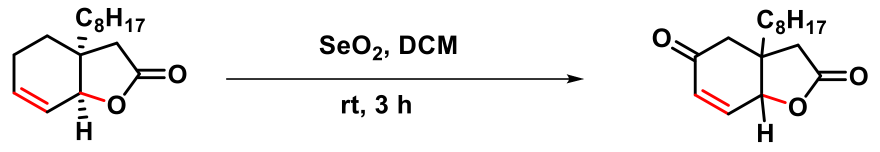

5f, 56%

In an oven-dried screw capped reaction tube was charged with magnetic stir-bar, unsaturated bicyclic lactone **2g** (1.0 equiv.), SeO_2 (1.2 equiv.) then dichloromethane (2 mL) was added to it and the reaction was stirred for 3 h at room temperature. Upon completion the solvent was removed under reduce pressure and diluted with EtOAc. The organic layer was washed with brine solution two times and dried on anhydrous sodium sulfate. The solvent was evaporated under reduced pressure and the crude mixture was purified by column chromatography using silica (100-200 mesh size) and petroleum ether/ ethyl acetate as the eluent to yield allylic oxidation product in 56% (**5e**).

(3a*S*,7a*S*)-3a-(3-Methoxyphenyl)hexahydrobenzofuran-2(3H)-one (5a)

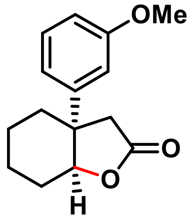

Eluent: ethyl acetate/ petroleum ether (13:87 v/v).
Appearance: colorless gummy.
Isolated yield: 87% (10.7 mg, 0.0435 mmol) (d.r. = 1:1)
^{1}H NMR (500 MHz, $CDCl_3$) δ 7.26 (ddd, *J* = 23.5, 15.9, 8.0 Hz, 2H), 7.03 – 6.67 (m, 6H), 4.89 (d, *J* = 3.4 Hz, 1H), 3.82 (s, 3H), 3.78 (s, 3H), 3.46 (s, 1H), 2.86 – 2.60 (m, 2H), 2.50 (s, 2H), 2.31 – 2.01 (m, 4H), 1.95 – 1.65 (m, 4H), 1.68 – 1.50 (m, 4H), 1.42 (dq, *J* = 20.7, 12.3, 10.6 Hz, 4H); **^{13}C NMR** (126 MHz, $CDCl_3$) δ 175.90, 160.00, 159.77, 144.06, 129.98, 129.36, 119.33, 118.92, 113.69, 113.64, 111.38, 110.66, 81.92, 55.48, 55.33, 47.11, 45.75, 40.93, 36.15, 34.57, 26.44, 26.32, 22.53, 21.03, 19.82; **HRMS (ESI)**: Calculated mass for $C_{15}H_{19}O_3$ $[M+H]^+$: 247.1334; observed mass: 247.1340.

(3a*S*,7a*S*)-3a-(3,4-Dimethoxyphenyl)hexahydrobenzofuran-2(3H)-one (5b)

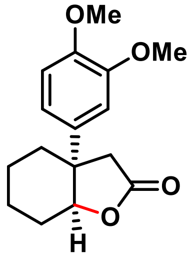

Eluent: ethyl acetate/ petroleum ether (18:82 v/v).

Appearance: colorless gummy.

Isolated yield: 85% (11.7 mg, 0.0425 mmol) (d.r. = 3:1)

^{1}H NMR (500 MHz, $CDCl_3$) δ 6.91 (d, *J* = 10.4 Hz, 1H), 6.87 – 6.70 (m, 2H), 4.94 – 4.84 (m, 1H), 3.95 – 3.80 (m, 6H), 3.63 (s, 1H), 2.72 (dd, *J* = 20.0, 9.8 Hz, 1H), 2.53 (s, 1H), 2.24 – 2.13 (m, 2H), 2.12 – 1.74 (m, 2H), 1.65 – 1.53 (m, 2H), 1.48 – 1.40 (m, 2H); **^{13}C NMR** (126 MHz, $CDCl_3$) δ 175.64, 148.84, 147.35, 119.19, 118.83, 111.34, 111.11, 110.60, 82.15, 56.29, 56.16, 55.95, 47.32, 40.55, 36.42, 34.46, 32.15, 29.58, 26.34, 22.91, 22.51; **HRMS (ESI)**: Calculated mass for $C_{16}H_{21}O_4$ $[M+H]^+$: 277.1440; observed mass: 277.1446.

(1*S*,6*S*)-6-(2-Hydroxyethyl)-6-methylcyclohex-2-en-1-ol (5c)

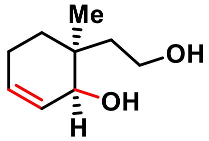

Eluent: ethyl acetate/ petroleum ether (35:65 v/v).

Appearance: colorless gummy.

Isolated yield: 84% (26.3 mg, 0.168 mmol)

^{1}H NMR (400 MHz, $CDCl_3$) δ 5.79 (dtd, *J* = 10.0, 3.5, 1.1 Hz, 1H), 5.74 – 5.65 (m, 1H), 3.93 – 3.76 (m, 2H), 3.71 (ddd, *J* = 10.8, 5.2, 4.3 Hz, 1H), 3.01 (s, 2H), 2.09 – 1.99 (m, 2H), 1.88 (ddd, *J* = 14.3, 9.6, 4.4 Hz, 1H), 1.57 (dt, *J* = 13.4, 7.3 Hz, 1H), 1.42 – 1.26 (m, 2H), 0.91 (s, 3H); **^{13}C NMR** (101 MHz, $CDCl_3$) δ 129.74, 129.71, 129.66, 128.45, 128.41, 128.36, 71.62, 71.59, 71.51, 71.47, 58.77, 39.95, 39.85, 36.10, 31.63, 29.88, 23.02, 22.98, 21.96, 21.82, 21.67; **HRMS (ESI)**: Calculated mass for $C_9H_{17}O_2$ $[M+H]^+$: 157.1229; observed mass: 157.1235.

(3a*S*,6*S*,7*S*,7a*R*)-6,7-Dihydroxy-3a-methylhexahydrobenzofuran-2(3H)-one (5d)

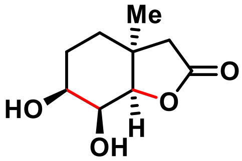

Eluent: ethyl acetate/ petroleum ether (35:65 v/v).

Appearance: white solid.

Isolated yield: 76% (28.3 mg, 0.152 mmol)

^{1}H NMR (400 MHz, $CDCl_3$) δ 4.24 (d, *J* = 5.6 Hz, 1H), 3.96 (dt, *J* = 8.0, 3.1 Hz, 1H), 3.85 (dd, *J* = 5.6, 3.0 Hz, 1H), 2.42 (d, *J* = 17.0 Hz, 1H), 2.24 (d, *J* = 17.0 Hz, 1H), 2.19 – 2.04 (m, 2H), 2.02 – 1.81 (m, 1H), 1.81 – 1.70 (m, 1H), 1.57 (tdd, *J* = 13.5, 7.5, 3.8 Hz, 2H), 1.26 (s, 3H); **^{13}C NMR** (101 MHz, $CDCl_3$) δ 176.45, 87.73, 71.60, 68.74, 42.21, 38.90, 29.43, 25.39, 24.77; **HRMS (ESI)**: Calculated mass for $C_9H_{14}NaO_4$ $[M+Na]^+$: 209.0790; observed mass: 209.0795.

(3a*S*,7a*S*)-3a-Octyl-3a,7a-dihydrobenzofuran-2,5(3H,4H)-dione (5e)

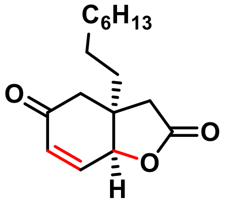

Eluent: ethyl acetate / petroleum ether (15:85 v/v)

Appearance: colorless gummy.

Isolated yield: 56% (15 mg, 0.056 mmol)

^{1}H NMR (500 MHz, $CDCl_3$) δ 6.89 (ddd, *J* = 9.2, 5.7, 2.4 Hz, 1H), 5.96 (dq, *J* = 10.1, 1.2 Hz, 1H), 5.72 (p, *J* = 6.1 Hz, 1H), 2.94 (d, *J* = 16.3 Hz, 1H), 2.63 (s, 1H), 2.46 (dddd, *J* = 19.0, 10.8, 5.3, 2.7 Hz, 1H), 2.40 – 2.33 (m, 1H), 2.24 (ddd, *J* = 13.7, 11.0, 5.4 Hz, 1H), 2.07 – 1.89 (m, 1H), 1.65 – 1.52 (m, 2H), 1.37 – 1.20 (m, 10H), 0.87 (t, *J* = 7.0 Hz, 3H); **^{13}C NMR** (126 MHz, $CDCl_3$) δ 201.04, 168.82, 148.99, 128.24, 66.69, 66.42, 66.14, 47.25, 38.39, 34.00, 32.02, 30.71, 30.19, 29.92, 29.57, 29.41, 24.05, 23.05, 22.83, 14.28; **HRMS (ESI)**: Calculated mass for $C_{16}H_{25}O_3$ $[M+H]^+$: 265.1804; observed mass: 265.1810.

Procedure for the formation of alkenoic acid intermediate:

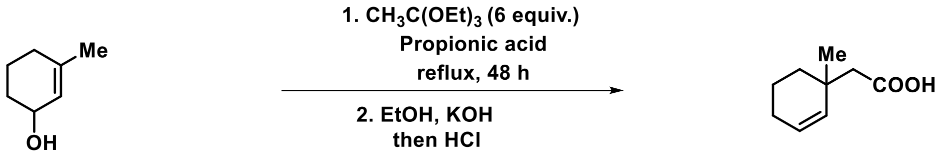

To a solution of 3-methylcyclohex-2-en-1-ol (2 mmol) in triethyl orthoacetate (6 *equiv.*) was added 0.5 mL propionic acid. The reaction mixture was heated to reflux and stirred for 2 days, then cooled to room temperature. Then it was diluted by 30 mL ethanol, and KOH (4 *equiv.*) was added. The mixture was stirred at room temperature for 8 hours. The solvent was removed under reduced pressure and 30 mL water was added. Then it was extracted with EtOAc (20 mL × 3). The organic layer was discarded and the aqueous layer was acidified with 12 M HCl and extracted with EtOAc (20 mL × 3). The combined organic layers were dried with anhydrous MgSO4, concentrated under reduced pressure and purified by column chromatography on silica gel (petroleum ether/EtOH = 10 : 1) to give the alkenoic acid.

2-(1-methylcyclohex-2-en-1-yl)acetic acid (5f)

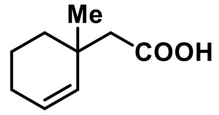

Eluent: ethyl acetate / petroleum ether (10:90 v/v)

Appearance: yellow

Isolated yield: 81% (25 mg)

^{1}H NMR (400 MHz, $CDCl_3$) δ 5.68 – 5.61 (m, 1H), 5.57 – 5.48 (m, 1H), 2.32 (d, *J* = 2.5 Hz, 2H), 1.96 (dd, *J* = 3.7, 1.8 Hz, 2H), 1.73 – 1.59 (m, 3H), 1.55 – 1.47 (m, 1H), 1.13 (s, 3H); **^{13}C NMR** (101 MHz, $CDCl_3$) δ 178.34, 134.94, 126.71, 46.63, 35.37, 34.21, 27.42, 25.10, 19.17.; **HRMS (ESI)**: Calculated mass for $C_9H_{15}O_2$ $[M+H]^+$: 155.1072; observed mass: 155.1073.

9. DFT Calculation:

Computational Methods.

Density functional theory (DFT) calculations were performed with *Gaussian 16* rev. B.01.[1] Geometry optimizations were initially performed using the global-hybrid meta-NGA (nonseparable gradient approximation) MN15 functional[2] with the def2-SVP[3,4] Karlsruhe-family basis set and the optimized structures further refined with a mix of larger basis set consisting of triple-ζ valence def2-TZVPPD (where 'D' indicates diffuse basis functions) for Pd[5,6] atom and def2-SVP[3,4] for all other atoms (BS1). Minima and transition structures on the potential energy surface (PES) were confirmed using harmonic frequency analysis at the same level of theory, showing respectively zero and one imaginary frequency. Where appropriate for cases where visual inspection of TS imaginary frequency is not obvious, intrinsic reaction coordinate (IRC) analyses[7,8] were performed to confirm that the found TSs connect to the right reactants and products.

Single point (SP) corrections were performed using MN15 functional and def2-QZVP[3] basis set for all atoms. The SMD implicit continuum solvation model[9] was used to account for the effect of hexafluoroisopropanol (HFIP) solvent on the computed Gibbs energy profile. Since HIFP solvent is not available in the list of default/pre-defined solvents in the *Gaussian 16* software, it is herein parametrised using a set of *seven* parameters.[9] These include 1) the static dielectric constant of the solvent at 25ºC (*Eps* = 16.7);[10–12] 2) dynamic (optical) dielectric constant – the square of the refractive index value of 1.275 at 20ºC was used[13] (*EpsInf = 1.625625*); 3) hydrogen bond acidity (*HBondAcidity = 0.77*)[14] and 4) hydrogen bond basicity (*HBondBasicity = 0.10*)[14], which are Abraham's *A* and *B* values respectively; 5) the surface tension of the solvent at interface (*SurfaceTensionAtInterface = 23.23*)[15] – this value is obtained from the conversion of the surface tension of HFIP at 16.14 mN/m at 25ºC[16] to cal mol^{-1} $Å^{-2}$ used in the SMD model by the conversion factor of 1 dyne/cm = 1 mN/m = 1.43932 cal mol^{-1} $Å^{-2}$ as outlined in the Truhlar's Minnesota Solvent Descriptor Database[17]; 6) carbon aromaticity – the fraction of aromatic carbons (*CarbonAromaticity = 0.00*) and 7) electronegative halogenicity – the fraction of halogens (*Electronegative Halogenicity = 0.60*). These parameters were specified using the keyword "SCRF = (SMD, Solvent= Generic, Read)" in *Gaussian 16*.

Gibbs energies were evaluated at the reaction temperature of 393.15 K (120ºC), using a quasi-RRHO treatment of vibrational entropies.[18,19] Vibrational entropies of frequencies below 100

cm^{-1} were obtained according to a free rotor description, using a smooth damping function to interpolate between the two limiting descriptions. The free energies were further corrected using standard concentration of 1 mol/L, which were used in solvation calculations. Unless otherwise stated, the final SMD (dichloroethane)-MN15/def2-QZVP//MN15/BS1 Gibbs energies are used for discussion throughout. *All Gibbs energy values in the text and figures are quoted in kcal mol^{-1}.* All molecular structures and molecular orbitals were visualized using *PyMOL* software.[20]

9.1. Model reaction

For computational modelling, we have chosen the following reaction (Scheme S1) for mechanistic studies.

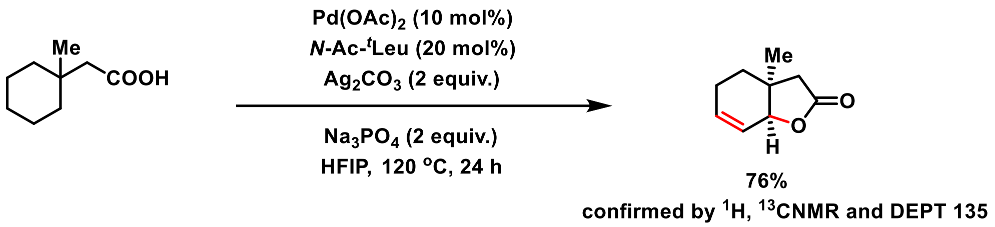

Scheme S1. Model reaction used for computational mechanistic studies.

9.2. Actual substrate for the reaction

The acid substrate in the reaction will get deprotonated by sodium phosphate, so that the actual substrate involved in the transformation will be its corresponding sodium salt. We calculated the thermodynamics for this reaction and found that the formation of sodium salt of the acid is indeed favoured, by 19.1 kcal mol^{-1} (Scheme S2).

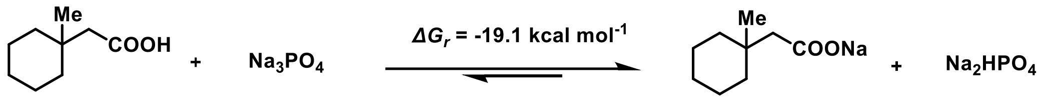

Scheme S2. Thermodynamics for the deprotonation of the acid substrate.

9.3. C–H activation transition states (TSs) – methylene vs methyl activation

The Gibbs energy profiles for the C–H activation of different H atoms are shown in Figure S2. In these transition states for the concerted metalation deprotonation (CMD) using the mono-protected amino acid (MPAA), N-acetyl *tert*-leucine, as an internal base, C–H activation can occur at either methylene carbon or methyl carbon. MPAA has been shown to lower the C–H

activation barrier over acetate ligands innate in $Pd(OAc)_2$ by forming favourable [5,6]-palladacyclic ring[21–26] conducive for C–H bond cleavage.

For the C–H activation at the methylene site, two different, prochiral H-atoms can be deprotonated, giving activated Pd–C bond either *cis* or *trans* to the methyl group. The pathway **INT1 → TS1 → INT2** *via* **TS1** forms **INT2** with Pd–C bond *cis* to the methyl group, whereas the pathway **INT1' → TS1' → INT2'** via **TS1'** forms **INT2'** with Pd–C bond *trans* to the methyl group. The pathway **INT1'' → TS1'' → INT2''** carries out C–H activation of the methyl C–H bond via **TS1''** (Figure S2).

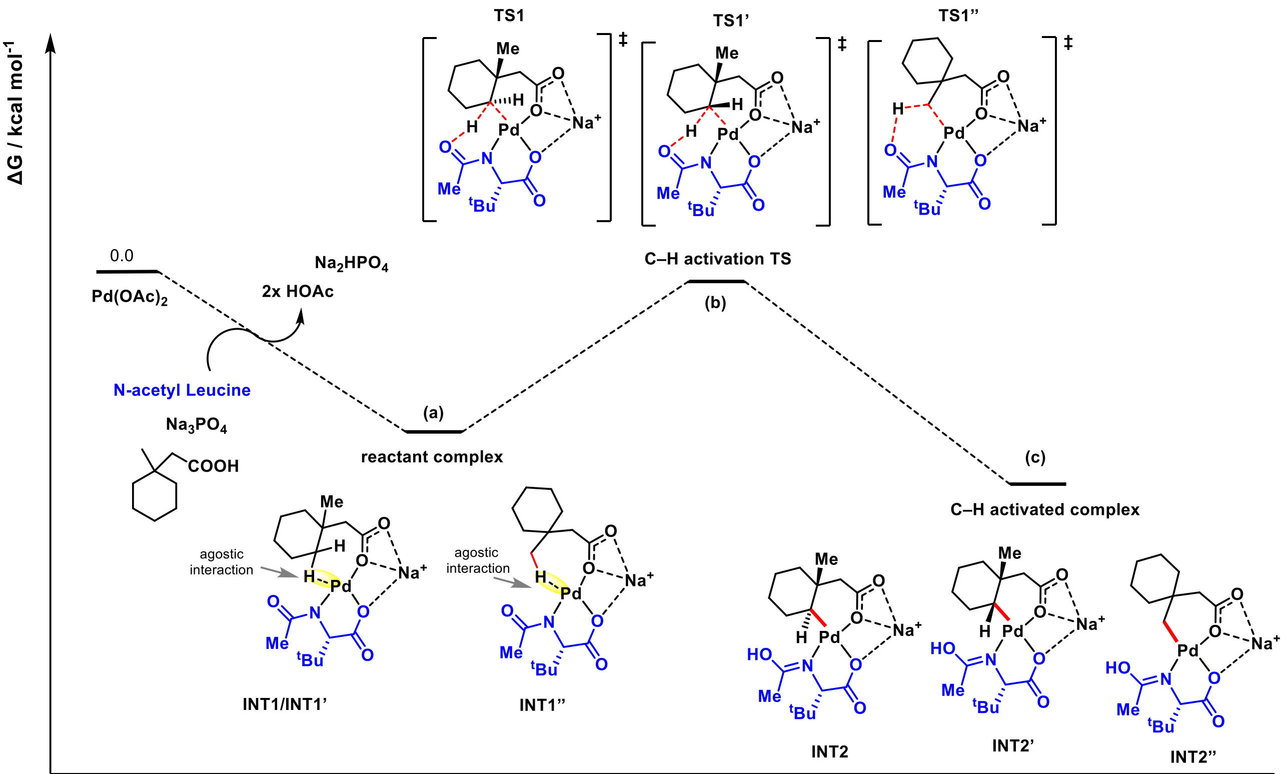

Pathways	(a)	(b)	(c)
**INT1 → TS1 → INT2**	-14.6	-3.0	-24.9
**INT1' → TS1' → INT2'**	-15.2	0.4	-23.5
**INT1'' → TS1'' → INT2''**	-15.8	-6.4	-25.4

Figure S1. Gibbs energy profiles for the C–H activation step at different sites. Values are quoted in kcal mol^{-1}.

The DFT optimised structures for these TSs and their reactant and product states are shown in Figure S3. Conformational flexibility in how the acetate coordinates to the Pd-centre to form differently ring-puckered orientations (TSs with different conformations) have been considered. For methylene activation, **TS1** (at -3.0 kcal mol^{-1}) has a lower barrier than **TS1'**

(at 0.4 kcal mol^{-1}), by 3.4 kcal mol^{-1}. The activation of C(methyl)–H bond via **TS1''** has the lowest barrier, at -6.4 kcal mol^{-1}, which is lower than the C(methylene)–H activation, **TS1** by 3.4 kcal mol^{-1}. This suggests that the C(methyl)–H activation is kinetically favoured by about 78 times than C(methylene)–H at the reaction temperature of 120ºC. However, the subsequent reductive elimination of **INT2''** occurs via **TS2''** at 19.9 kcal mol^{-1} (*vide infra*), giving a barrier of 45.3 kcal mol^{-1} from the activated complex **INT2''**. Thus, the reductive elimination step could not occur at the reaction condition and that the C(methyl)–H activation leads to catalytic off-cycle. It is likely that **INT2''** reverts back to the reactant complex, via **TS1''**, with a backwards barrier from **INT2'' to INT1''** of 21.1 kcal mol^{-1} than going forward with a barrier of 45.3 kcal mol^{-1} to undergo reductive elimination.

**TS1**	**TS1-c2**
$\Delta G^{\ddagger}$ = -3.0 kcal mol^{-1}	-2.9 kcal mol^{-1}
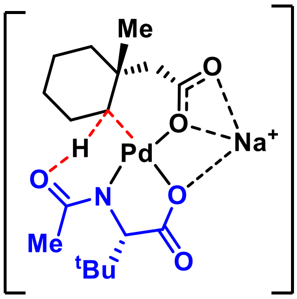	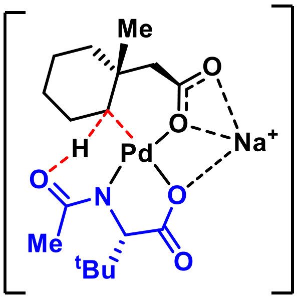
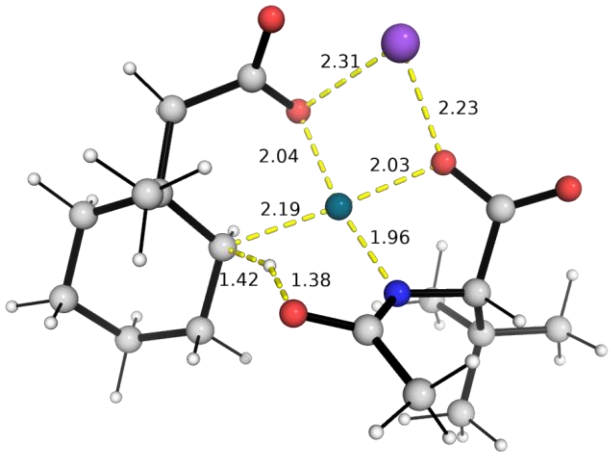	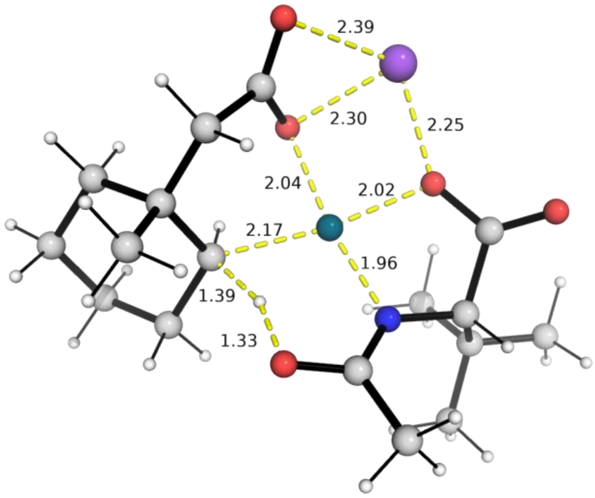
**INT2**	**INT2-c2**
$\Delta G^{\ddagger}$ = -24.9 kcal mol^{-1}	

<table>
<tr><td>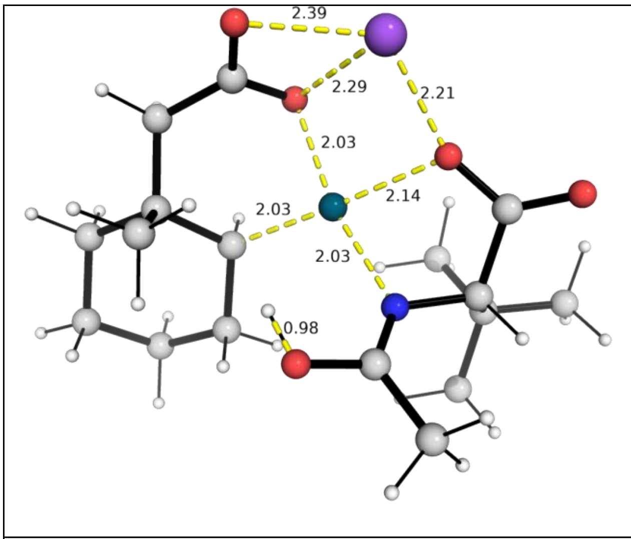
</td><td>Structure optimises to INT2</td></tr>
<tr><td>TS1’</td><td>TS1’-c2</td></tr>
<tr><td>$\Delta G^{\ddagger}$ = 0.4 kcal mol^{-1}</td><td>2.2 kcal mol^{-1}</td></tr>
<tr><td>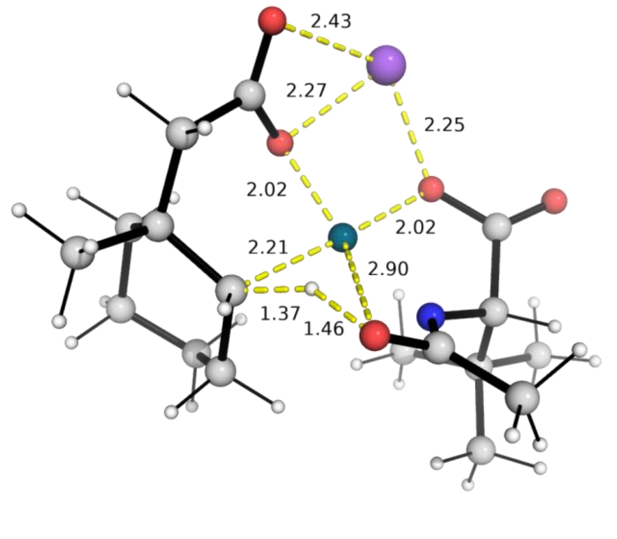
</td><td>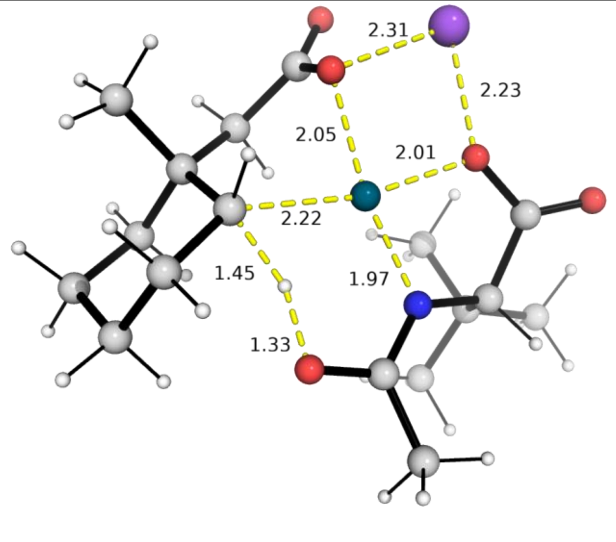
</td></tr>
<tr><td>INT2’</td><td>INT2’-c2</td></tr>
<tr><td>$\Delta G^{\ddagger}$ = -23.5 kcal mol^{-1}</td><td>-17.5 kcal mol^{-1}</td></tr>
</table>

<table>
<tr><td>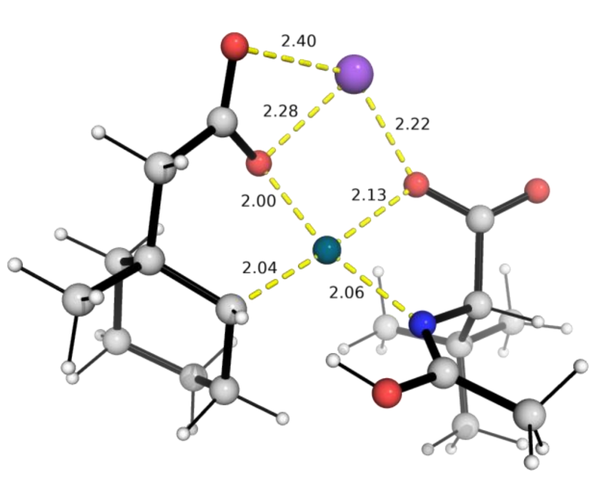
</td><td>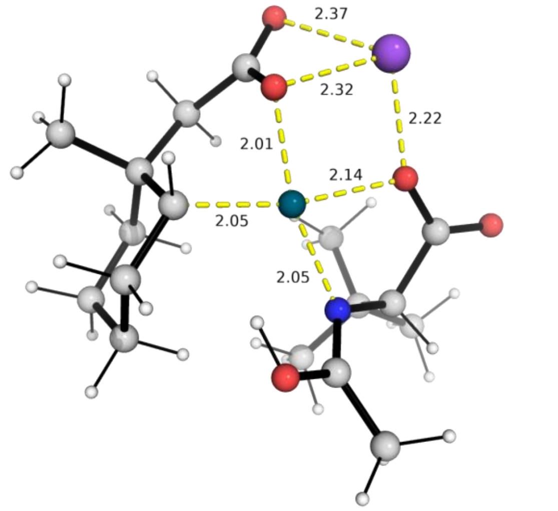
</td></tr>
<tr><td>TS1’’</td><td>TS1’’-c2</td></tr>
<tr><td>ΔG‡ = -6.4 kcal mol^{-1}</td><td>-4.3 kcal mol^{-1}</td></tr>
<tr><td>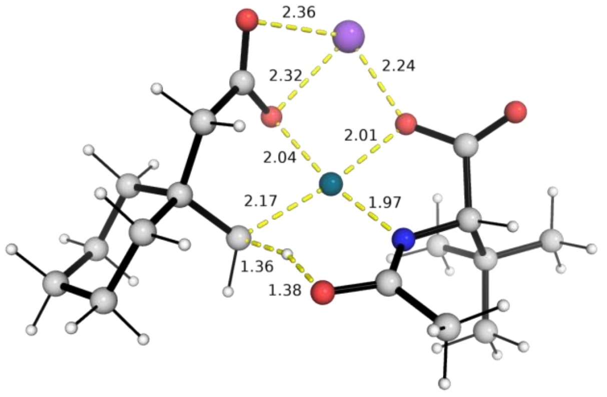
</td><td>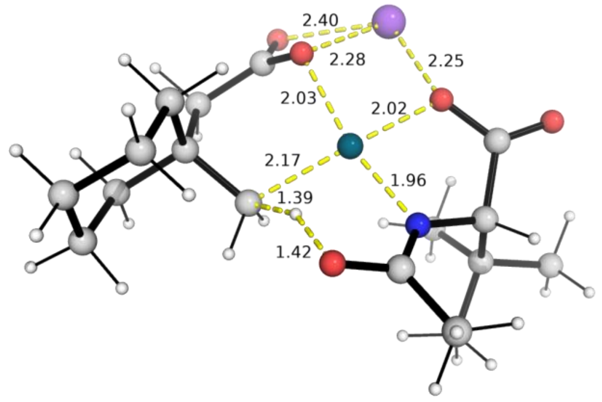
</td></tr>
<tr><td>INT2’’</td><td>INT2’’-c2</td></tr>
<tr><td>ΔG‡ = -25.4 kcal mol^{-1}</td><td>-22.9 kcal mol^{-1}</td></tr>
<tr><td>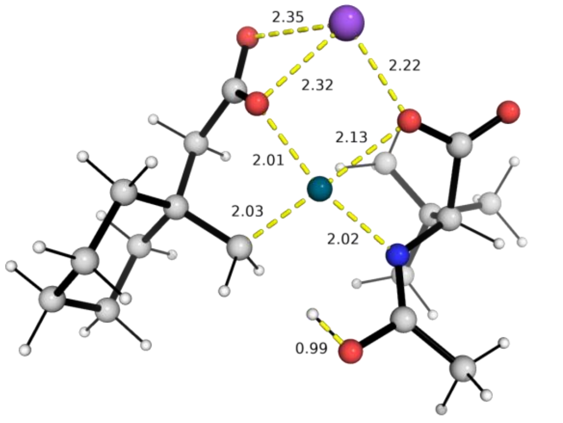
</td><td>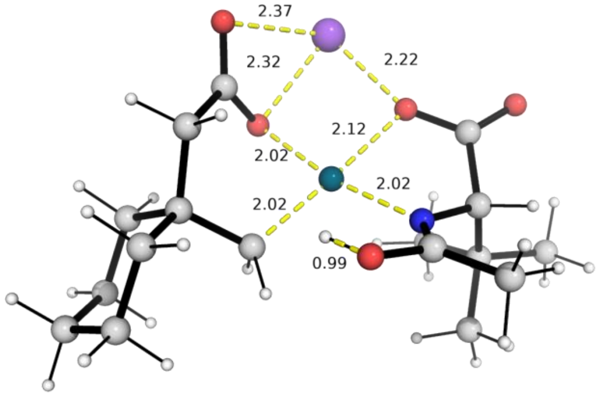
</td></tr>
</table>

Figure S2. DFT optimised transition state structures for the C–H activation of substrate at different sites. Activation barriers are taken relative to the sum of starting materials.

9.4. Reductive C–O bond coupling in C–H activated complexes

For the C(methyl)–H activation pathway **INT1'' → TS1'' → INT2''**, no β-H is available on the quaternary carbon in the C–H activated intermediate **INT2''** for elimination. We considered the alternative C–O bond formation following reductive elimination in **INT2''** to give the spirocyclic lactone product (Scheme S3a)). For the C(methylene)–H activation pathway **INT1 → TS1 → INT2**, in addition to β-H elimination that was considered, we also considered the alternative pathway of C–O reductive coupling to give the bicyclic lactone side product (Scheme S3b)).

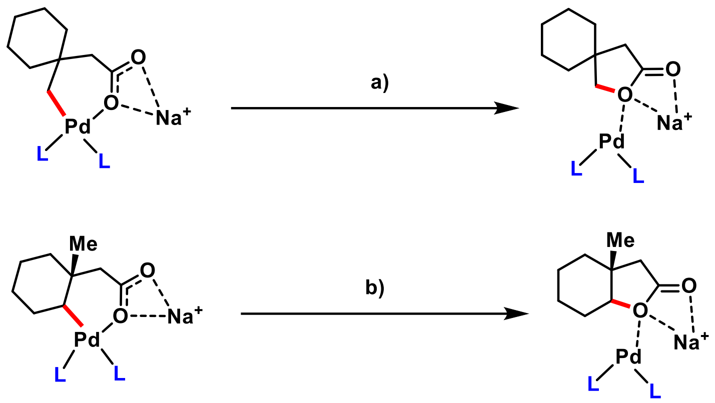

Scheme S3. Reductive C–O bond formation in a) C(methyl)–H activated complex and b) C(methylene)–H activated complex.

The optimized DFT TS structures with different ligands and conformations are shown in Figure S4 and the Gibbs energy profile for the reaction pathway following from C(methyl)–H activation is shown in Figure S5. We see that the reductive elimination with MPAA ligand coordinating in its imidic acid form (**TS2''-c2**, at 28.0 kcal mol^{-1}) has a much higher barrier than with MPAA coordinating via bidentate acetate moiety (**TS2''**, at 19.9 kcal mol^{-1}). We further note that the replacement of MPAA in **TS2''** by acetate ligand gives the C–O reductive coupling transition structure **TS2''-ac** at 19.4 kcal mol^{-1}, which is very similar to **TS2''**. This is likely because both MPAA and acetate ligands, in **TS2''** and **TS2''-ac** respectively, coordinate in a bidentate fashion (Figure S4), where both species have two Pd–O interactions; the Pd–O interactions are similar in both cases and are dominant over possible non-covalent interactions (NCIs) in the side chains of the MPAA ligand in **TS2''**.

From the Gibbs energy profile in Figure S5, we see that the activation barriers for the reductive C–O coupling is 44.8 kcal mol^{-1} (from **INT2''** to **TS2''-ac**) and 45.3 kcal mol^{-1} (from **INT2''** to **TS2''**), which are thermodynamically inaccessible at the reaction temperature of 120ºC. On the other hand, the reaction from **INT2''** back to **INT1''** though **TS1''** has a barrier of 19.0

kcal mol^{-1}, which is much lower than the forward reaction from **INT2’’** to **INT4’’**. Thus, the C(methyl)–H activation step is reversible.

**TS2’’**	**TS2’’-c2**
$\Delta G^{\ddagger}$ = 19.9 kcal mol^{-1}	28.0 kcal mol^{-1}
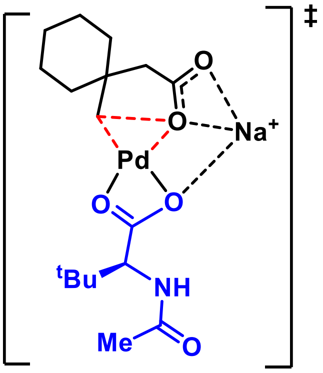	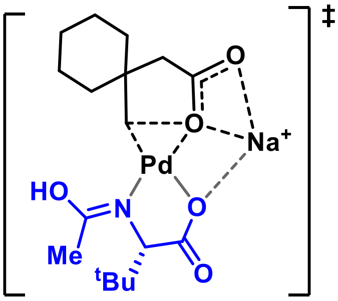
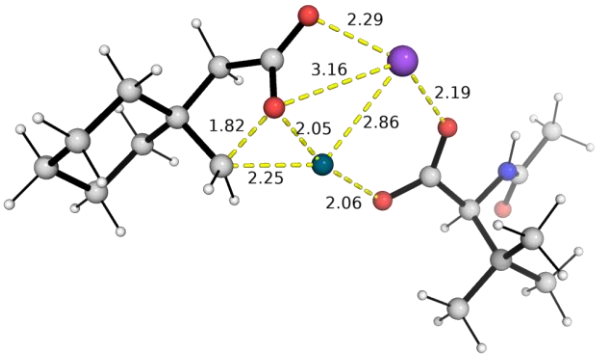	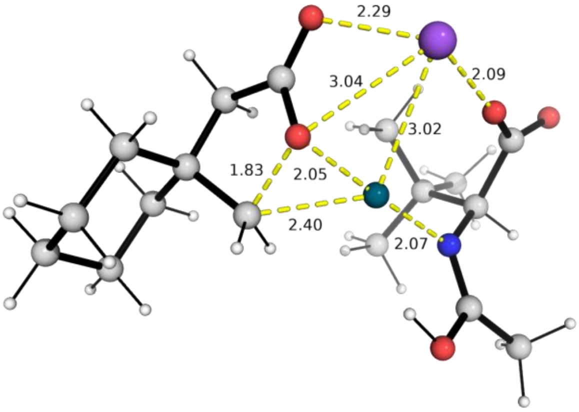
**TS2’’-ac**	
$\Delta G^{\ddagger}$ = 19.4 kcal mol^{-1}	
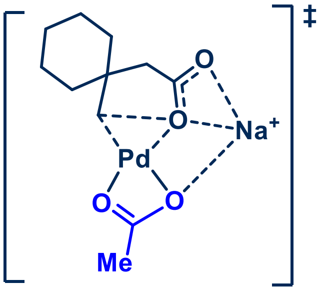	

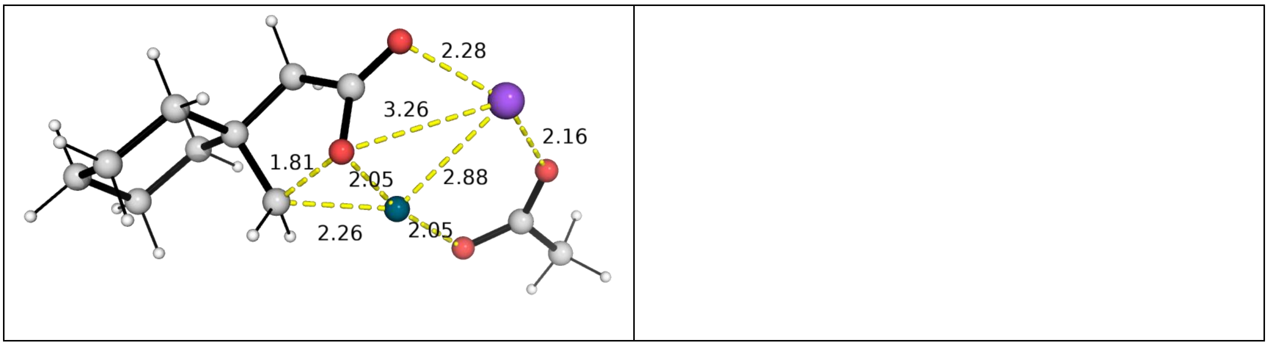

Figure S4. DFT optimised transition state structures for the reductive elimination to form C–O bond from the C(methyl)–H activated intermediate. Activation barriers are taken relative to the sum of starting materials.

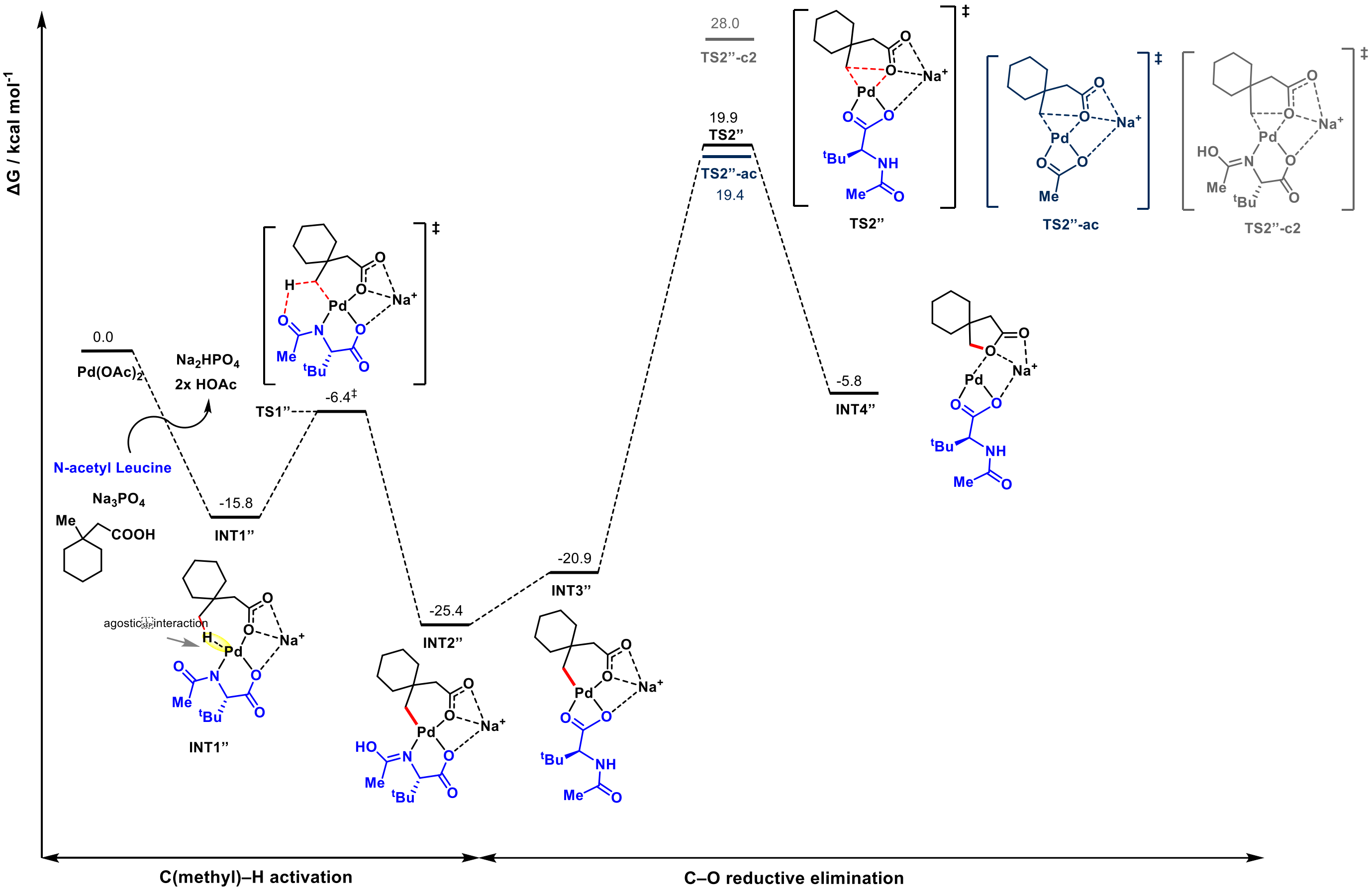

Figure S5. Gibbs energy profile for the reaction pathway following from C(methyl)–H activation.

To consider the C–O reductive coupling in the C(methylene)–H activated complex **INT2**, to give the bicyclic lactone side product (Scheme S3b)), we separately performed relaxed PES scans along the prospective C–O bond starting from optimised structure **INT2** and **INT3**. Using the highest energy structures on these PESs as initial guess, we successfully located the TSs for the C–O reductive elimination. The DFT optimised TS structures are shown in Figure S6 and the Gibbs energy profile for the reaction pathways following from C(methylene)–H

activation, comparing β-H elimination vs C–O reductive elimination, is shown in Figure S7. We note that the barriers for the C–O reductive elimination are similar to those identified C(methyl)–H activation pathway (**TS2'', TS2''-c2**, and **TS2''-ac**, Figure S4). In addition, these barriers (**TS3a** and **TS3b**) are much higher than the barrier for β-H elimination (**TS3**), by more than 35 kcal mol^{-1}, suggesting C–O reductive elimination is much energetically less favourable than β-H elimination.

TS3a	TS3b
$\Delta G^{\ddagger}$ = 20.9 kcal mol^{-1}	28.6 kcal mol^{-1}
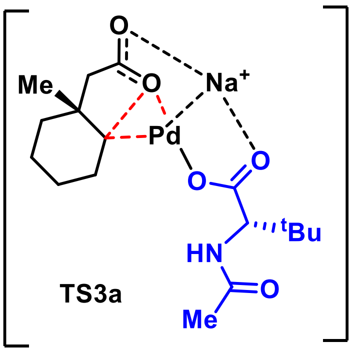	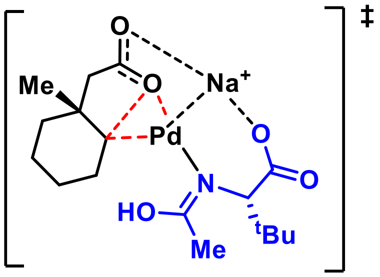 TS3b
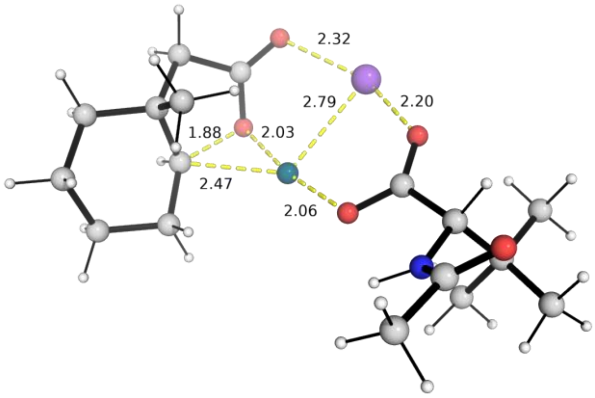	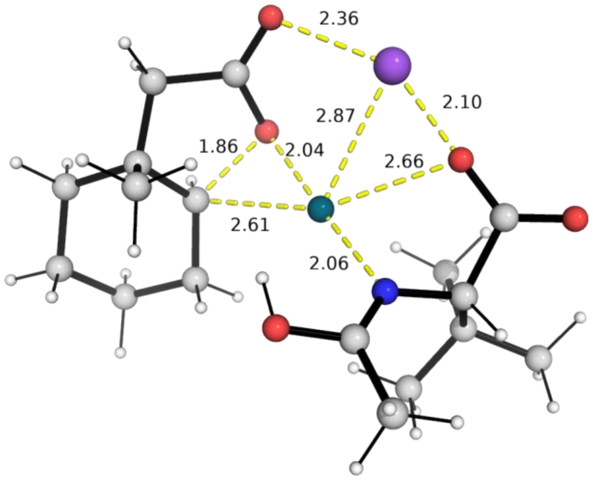

Figure S6. DFT optimised transition state structures for the reductive elimination to form C–O bond from the C(methylene)–H activated intermediate **INT2** and **INT3**. Activation barriers are taken relative to the sum of starting materials.

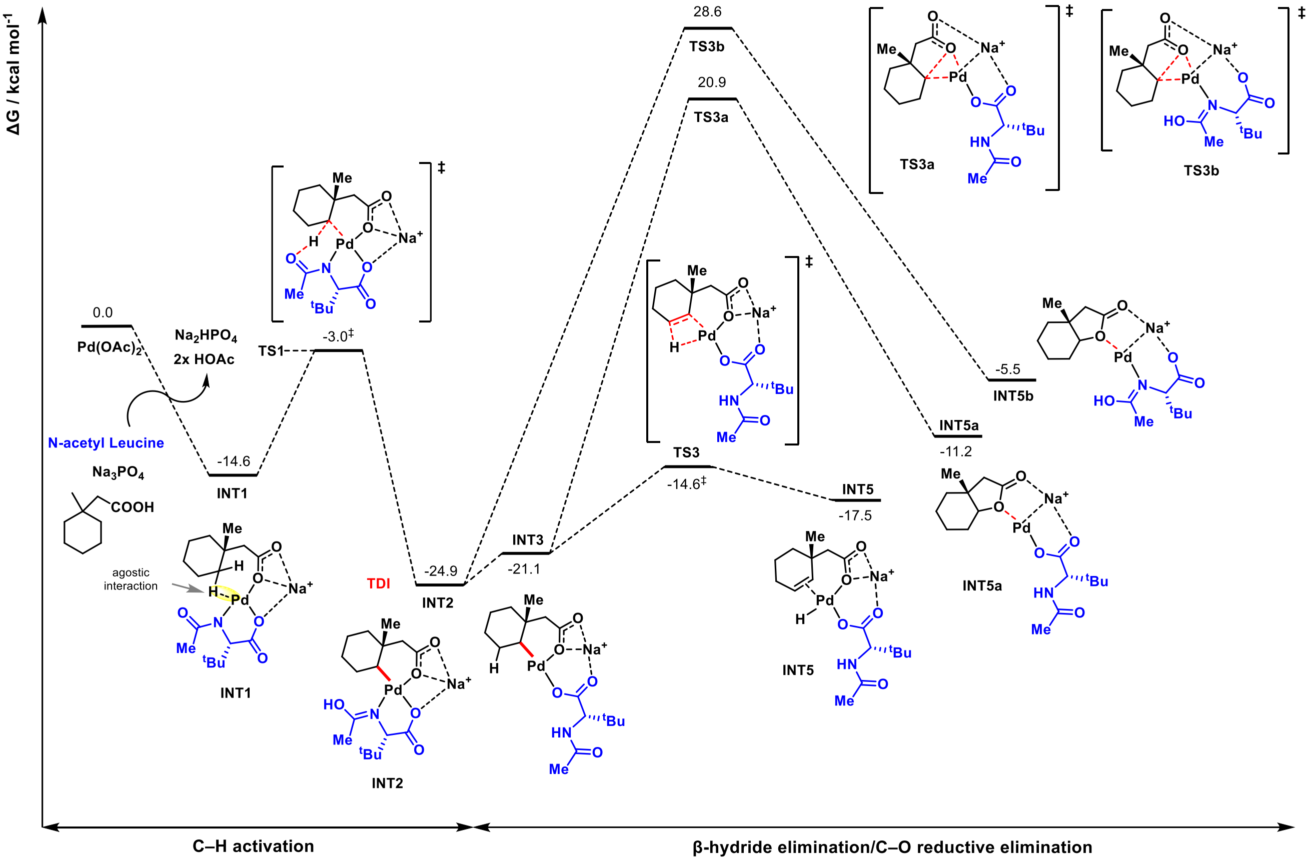

Figure S7. Gibbs energy profile for the reaction pathways (β-H elimination vs C–O reductive elimination) following from C(methylene)–H activation.

9.5. Rotational transition state for coordinating β-H to vacant Pd-site for elimination

From the C(methylene)–H activated **INT2**, a stable intermediate resulting from both **TS1** and **TS1-c2** (Figure S3), we found that the structure undergoes a rotation along the H(1)–C–C–Pd dihedral angle (**TS2**) to position one of the adjacent methylene H atoms to coordinate to the Pd-centre via agostic interaction. The DFT optimised structure **TS2** and the resulting intermediate **INT4** are shown in Figure S8.

TS2	INT4
$\Delta G^{\ddagger}$ = -16.8 kcal mol^{-1}	-18.8 kcal mol^{-1}
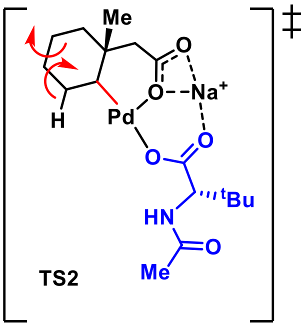 TS2	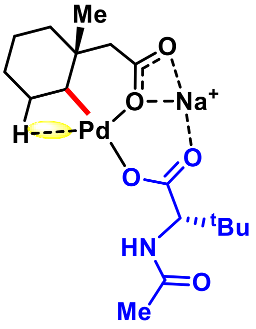

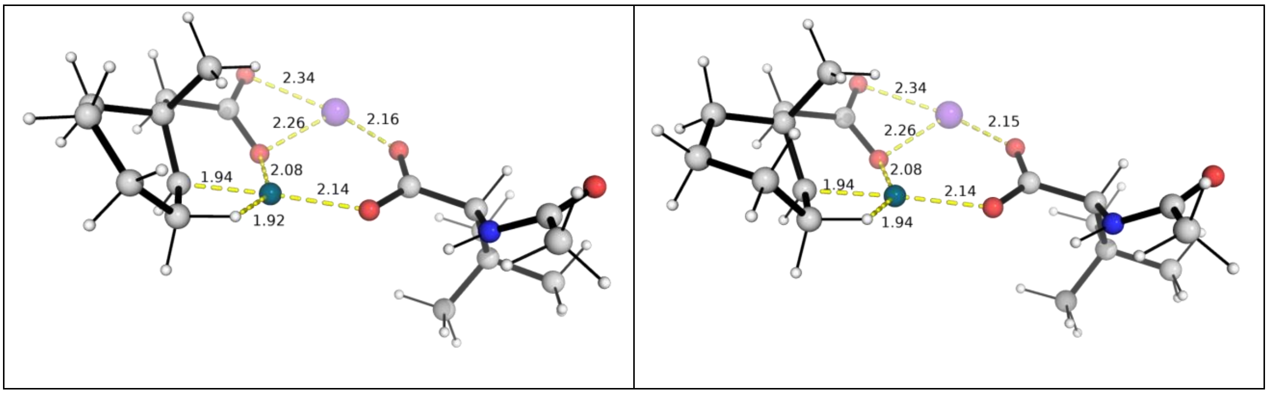

Figure S8. DFT optimised structures for the dihedral angle rotation in intermediate **INT2** to give **INT4** with CH–Pd agostic interaction. Activation barriers are taken relative to the sum of starting materials.

We note that, however, there is no such a rotational barrier to position the other H atom to coordinate to the Pd centre, as shown by the relaxed PES scan along the H(2)–C–C–Pd dihedral angle which shows no maximum point as the dihedral angle sweeps from negative value to positive value (Figure S9). This indicates that may not be a rotational barrier to bring the H(2) atom to coordinate to Pd-centre to give CH–Pd agostic interaction.

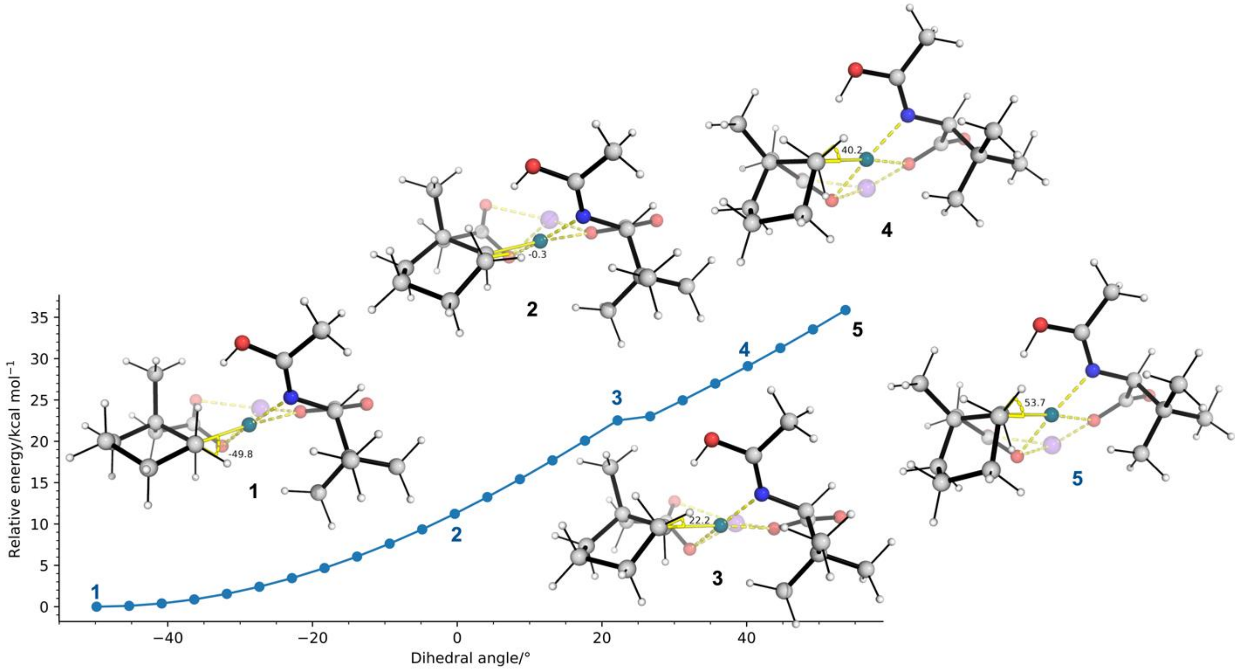

Figure S9. Relaxed PES scan in the gas-phase along H(2)–C–C–Pd dihedral angle. Gas-phase energies are used without further corrections.

9.6. Stereo determining migratory insertion TSs

Figure S10 shows the DFT optimised TS structures for the migratory insertion of the cyclohexene olefin C=C double bond into the Pd–O bond. This step is stereodetermining as the formation of new C–O bond resulting from the attack of O-atom from either side of the C=C

bond generates different stereochemistry at the fused carbons. The reaction pathway proceeding via transition state **TS4**, at 3.8 kcal mol^{-1}, gives the product with observed stereochemistry at the fused ring (*cis*-isomer). On the other hand, the reaction pathway proceeding via transition state **TS4’**, at 12.6 kcal mol^{-1}, would give the *trans*-isomer. The barrier difference of 8.8 kcal mol^{-1} suggests that **TS4** will be favoured kinetically by around 78,000 times, indicating that the *cis*-isomer will be formed predominantly, consistent with experimental observation of stereochemistry of the lactone product at the fused rings.

**TS4**	**TS4’**
$\Delta G^{\ddagger}$ = 3.8 kcal mol^{-1}	12.6 kcal mol^{-1}
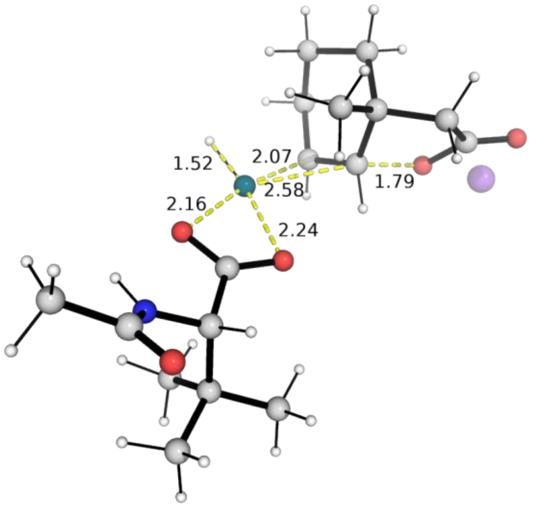	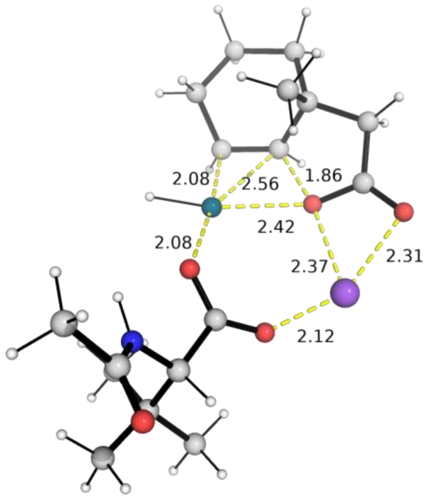

Figure S10. DFT optimised transition state structures for the stereodetermining migratory insertion step. Activation barriers are taken relative to the sum of starting materials.

9.7. β-H elimination TSs

β-H elimination occurs firstly after the C–H activation step to give the cyclohexene and secondly after the migratory insertion of cyclohexene C=C bond into Pd–O bond to regenerate the cyclohexene C=C bond, as the lactone ring closes. The DFT optimised TS structures for these steps are given in Figure S11.

**TS3**	**TS5**
$\Delta G^{\ddagger}$ = -14.6 kcal mol^{-1}	7.5 kcal mol^{-1}

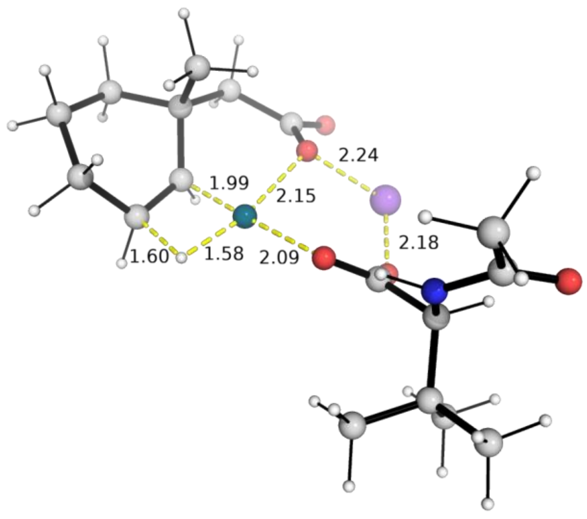	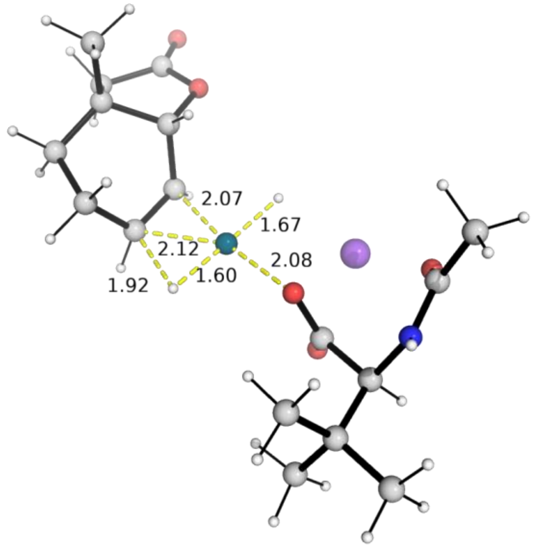
**TS5-c2**	**TS5-c3**
$\Delta G^{\ddagger}$ = 12.6 kcal mol^{-1}	22.2 kcal mol^{-1}
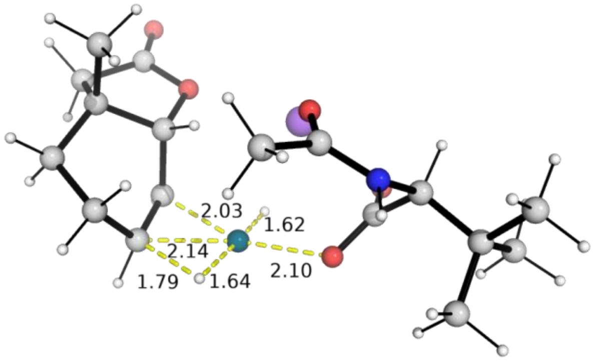	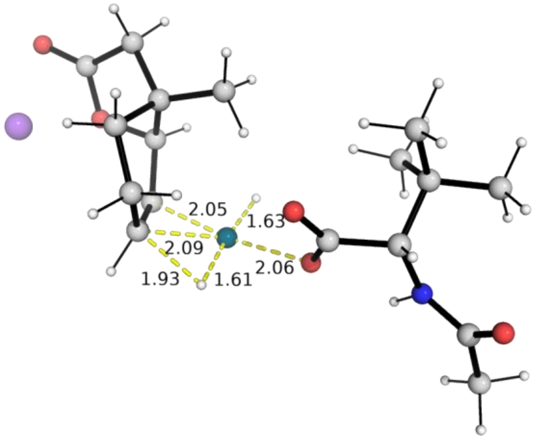

Figure S11. DFT optimised transition state structures for the β-H elimination step. Activation barriers are taken relative to the sum of starting materials. Conformers are denoted c2, c3 etc.

9.8. Product release

The formation of palladium-bound lactone product, **INT9**, is endergonic and uphill by 6.9 kcal mol^{-1} and is thus thermodynamically disfavoured. We investigated the release of the lactone product from the catalyst centre (Figure S12).

The release of the lactone product from **INT9** in the absence of another species gives **INT10'**, with Pd centre having a vacant site (Figure S12a). This process is uphill by 16.7 kcal mol^{-1} and is unfavourable. When one HFIP solvent molecule is used to displace the product from **INT9** (Figure 12b), the resulting Pd-species formed, **INT11'**, is uphill by 14.1 kcal mol^{-1}. This is still thermodynamically unfavourable. When the silver carbonate salt is used to displace the lactone

product (Figure S12c), the resulting Pd–Ag species is thermodynamically downhill ad is thus favourable.

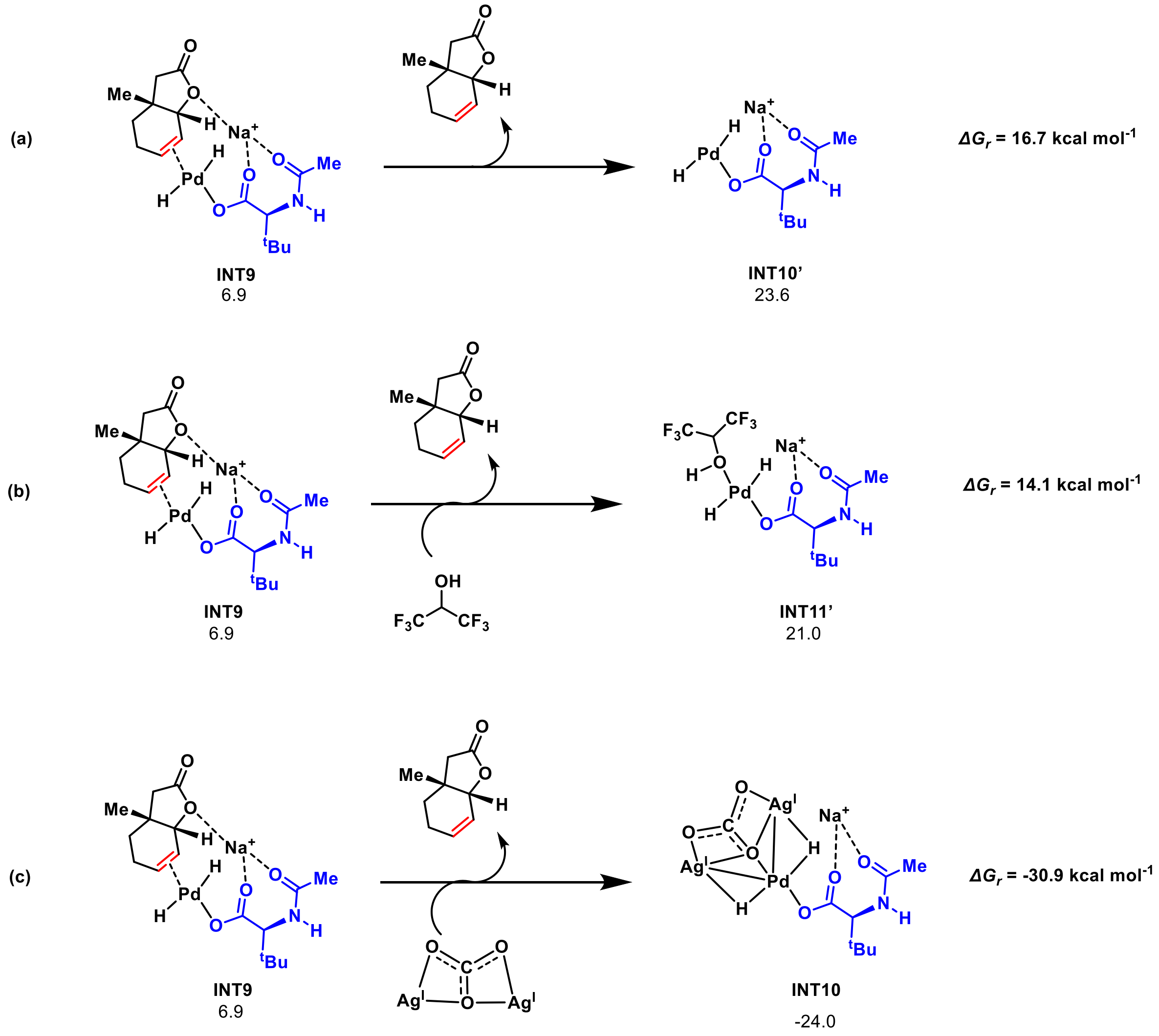

Figure S12. Thermodynamics for the release of bicyclic lactone product from Pd-catalyst.

9.9. Regioselectivity studies for 3-substituted substrate

3-Phenyl substituted substrate, where Ph and Me groups are syn to each other (relative stereochemistry), was used as a representative to study the regioselectivity outcomes for 3-substituted substrates using DFT. Herein, only a single enantiomer was used for DFT calculations as minor images of all structures are isoenergetic (the syn relation between Me and Aryl has been established through NOESY experiment). As the reaction outcomes depend on the turnover frequency-determining intermediate (TDI) and transition state (TDTS)[27], we study the energetics for these two states for the competing pathways. Figure S13 shows the associated Gibbs energy profile. Different conformations where the Me and Ph groups can be either axial or equatorial, were considered for both the TDI and the TDTS (lowest energy

conformers **INT2** and **TS5** from the study of unsubstituted substrate were used as guess structures) and the DFT-optimised structures were shown in Figure S14. For the competing pathways for the formation of regioisomeric products shown in Figure S13, the overall TDI for the reaction is **INT2Ph-equatorial,** as the C–H activation step leading to the activated complex **INT2-regio-equatorial** is reversible, such that **INT2-regio-equatorial** will revert back to the starting materials and form **INT2Ph-equatorial**, which is more thermodynamically stable (competing pathways with shared states need to take the lowest/most stable state into account)[27]. As such, the selectivity outcomes for the formation of the major vs minor product depends on the difference in the activation barriers for the TDTSs. **TS5Ph-equatorial** (at 4.3 kcal mol^{-1}) leading to the major product has a barrier that is 3.9 kcal mol^{-1} lower than **TS5Ph-regio** (at 8.2 kcal mol^{-1}) leading to the minor product. This predicts the right major product which was experimentally observed.

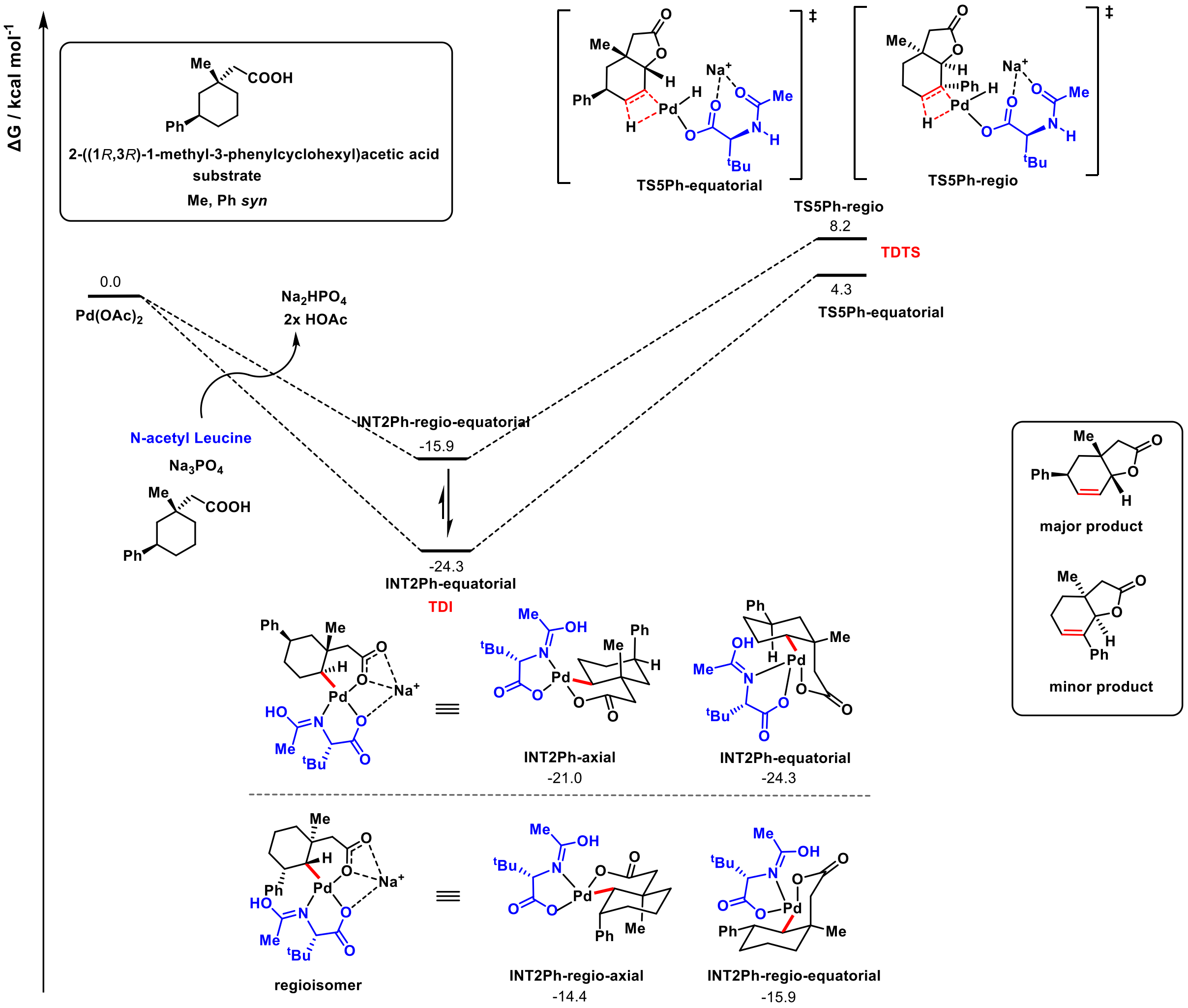

Figure S13. Gibbs energy profile for the turnover-frequency determining intermediate (TDI) and transition state (TDTS) for the functionalisation of 3-substituted substrate.

<table>
<tr><th>INT2Ph-equatorial</th><th>INT2Ph-axial</th></tr>
<tr><td>ΔG = -24.3 kcal mol^{-1}</td><td>-21.0 kcal mol^{-1}</td></tr>
<tr><td>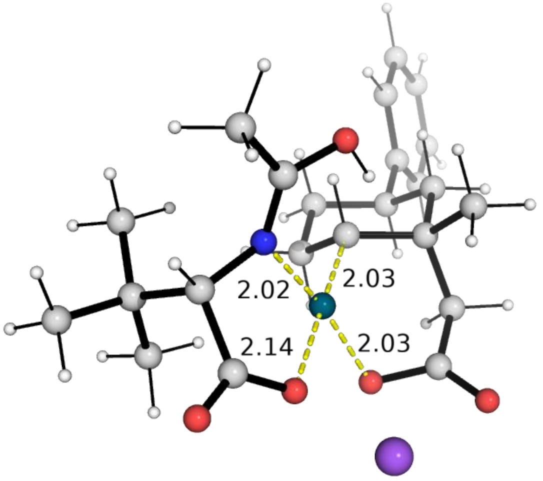
</td><td>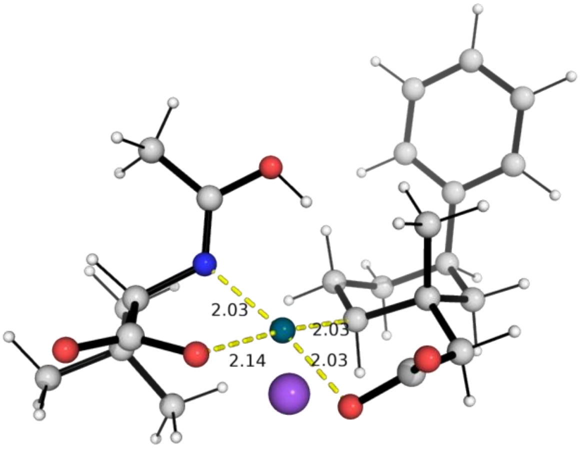
</td></tr>
<tr><td>INT2Ph-regio-equatorial</td><td>INT2Ph-regio-axial</td></tr>
<tr><td>ΔG = -15.9 kcal mol^{-1}</td><td>-14.4 kcal mol^{-1}</td></tr>
<tr><td>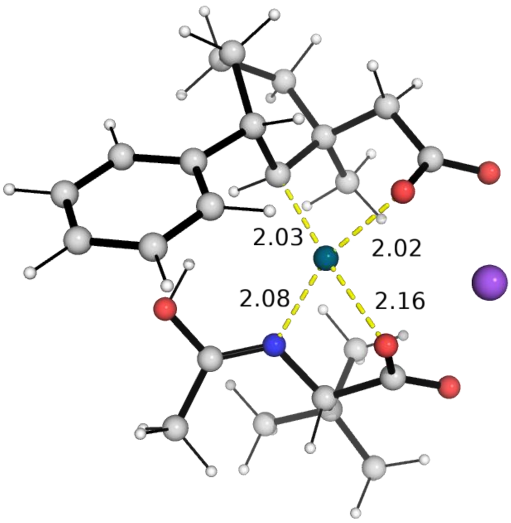
</td><td>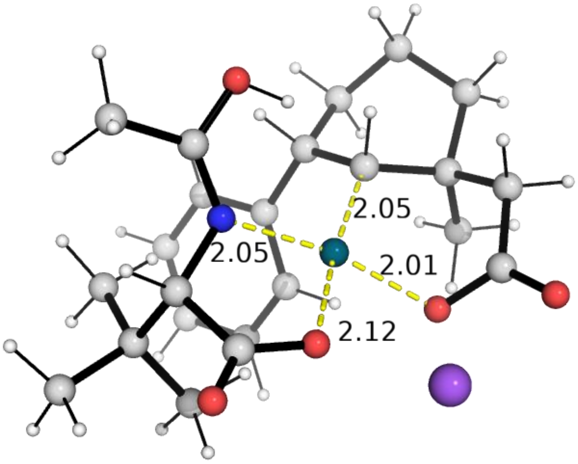
</td></tr>
<tr><td>TS5Ph-equatorial</td><td>TS5Ph-axial</td></tr>
<tr><td>ΔG‡ = 4.3 kcal mol^{-1}</td><td>12.3 kcal mol^{-1}</td></tr>
</table>

<table>
<tr><td>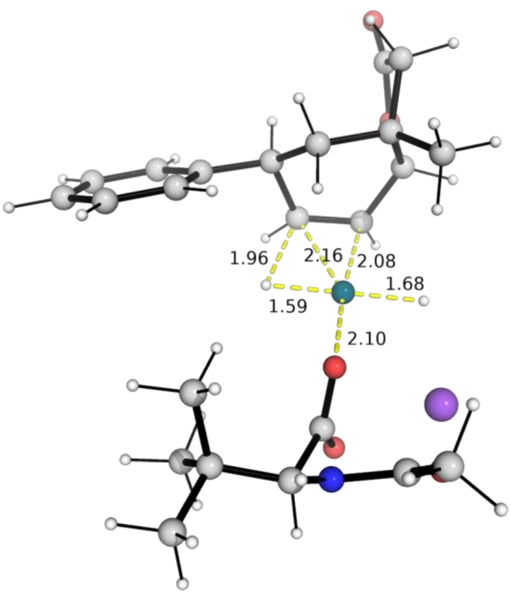</td><td>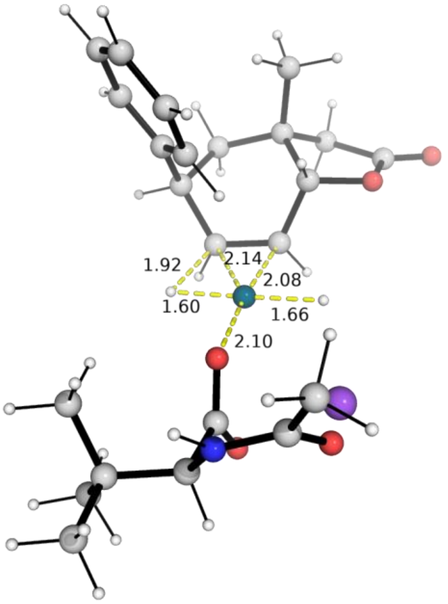</td></tr>
<tr><td>TS5Ph-equatorial-c2</td><td>TS5Ph-axial-c2</td></tr>
<tr><td>$\Delta G^{\ddagger}$ = 25.9 kcal mol^{-1}</td><td>22.3 kcal mol^{-1}</td></tr>
<tr><td>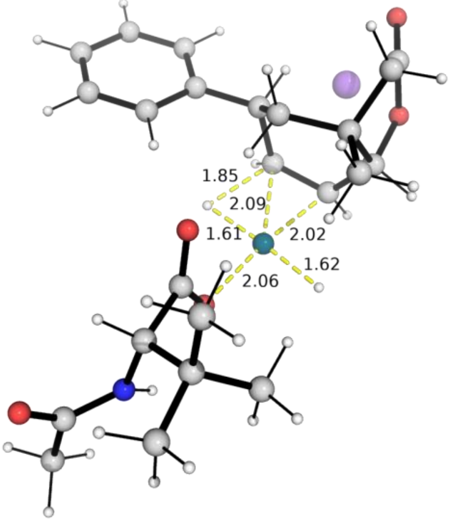</td><td>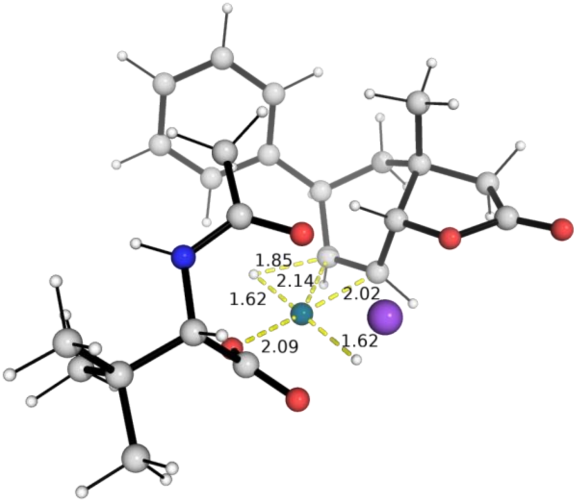</td></tr>
<tr><td>TS5Ph-regio</td><td></td></tr>
<tr><td>$\Delta G^{\ddagger}$ = 8.2 kcal mol^{-1}</td><td></td></tr>
</table>

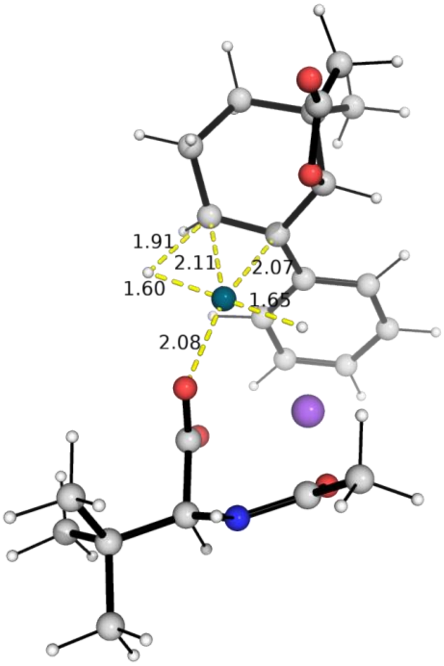

Figure S14. DFT optimised turnover frequency-determining intermediate (TDI) and transition state (TDTS) structures for the 3-substituted substrate. Gibbs energy values are taken relative to the sum of starting materials. Different transition state conformers are included and are denoted by c2, c3 etc, in increasing energy.

9.10. Optimized structures and absolute energies, zero-point energies

Geometries of all optimized structures (in *.xyz* format with their associated energy in Hartrees) are included in a separate folder named *final_xyz* with an associated readme.txt file. All these data have been deposited and uploaded to zenodo.org (https://zenodo.org/record/7516355; DOI: 10.5281/zenodo.7516355).

Absolute values (in Hartrees) for SCF energy, zero-point vibrational energy (ZPE), enthalpy and quasi-harmonic Gibbs free energy (at 120ºC/393.15 K) for optimized structures are given below. Single point corrections in SMD hexafluoroisopropanol using MN15/def2-QZVP level of theory are also included.

Structure	E/au	ZPE/au	H/au	T.S/au	qh-G/au	SP SMD MN15/def2-QZVP
**HOAc**	-228.644533	0.062197	-228.57411	0.041723	-228.61541	-229.06457600

**acetate**	-228.059294	0.048219	-228.00312	0.04228	-228.044179	-228.592055
**PdOAc2_monomer**	-583.809931	0.104326	-583.68809	0.068364	-583.753096	-584.634037
**Na_substrate_2_salt**	-663.638026	0.230412	-663.38552	0.076065	-663.458441	-664.677056
**substrate_2**	-502.040305	0.241876	-501.77836	0.069148	-501.845831	-502.967425
**Na2HPO4**	-966.579407	0.029498	-966.53614	0.057707	-966.592495	-967.610931
**Na3PO4**	-1128.161176	0.019146	-1128.1266	0.061821	-1128.187368	-1129.292047
**Nacetyltertleucine**	-593.162022	0.230084	-592.90756	0.080011	-592.984379	-594.257468
**hfip**	-788.500116	0.064458	-788.42028	0.061798	-788.480223	-789.866397
**Ag2CO3**	-556.727692	0.017256	-556.6994	0.055206	-556.753788	-557.239514
**Pd_leucine_2hfip**	-2296.722852	0.341101	-2296.3255	0.155423	-2296.46888	-2300.5114
**lactone_prd**	-499.613569	0.196067	-499.40007	0.062779	-499.461986	-500.529069
**lactone_prd-c2**	-499.620948	0.196417	-499.40723	0.062024	-499.468845	-500.536485
**INT1’**	-1383.335648	0.43882	-1382.8495	0.129897	-1382.972713	-1385.432754

**TS1’**	-1383.323155	0.43538	-1382.8415	0.126011	-1382.962016	-1385.413749
**INT2’**	-1383.364381	0.441078	-1382.8764	0.127674	-1382.998363	-1385.456831
**INT1’-c2**	-1383.342748	0.439502	-1382.856	0.131174	-1382.979685	-1385.440655
**TS1’-c2**	-1383.320921	0.436012	-1382.839	0.125391	-1382.958815	-1385.411889
**INT2’-c2**	-1383.354526	0.441179	-1382.8665	0.126831	-1382.988131	-1385.447502
**INT1’’**	-1383.343151	0.439278	-1382.8567	0.13052	-1382.979811	-1385.441807
**TS1’’-c2**	-1383.329042	0.435238	-1382.8477	0.127481	-1382.968455	-1385.420691
**INT2’’**	-1383.365984	0.440823	-1382.8783	0.129708	-1383.000675	-1385.459128
**TS2’’**	-1383.279908	0.438468	-1382.7939	0.136725	-1382.91985	-1385.381637
**TS2’’-c2**	-1383.264164	0.43842	-1382.7786	0.132998	-1382.902705	-1385.370122
**INT1’’-c2**	-1383.342957	0.439803	-1382.8562	0.130316	-1382.978913	-1385.440542
**TS1’’**	-1383.333053	0.43579	-1382.8514	0.126471	-1382.971542	-1385.425058
**INT2’’-c2**	-1383.361396	0.44101	-1382.8736	0.129636	-1382.995893	-1385.455347

**INT1**	-1383.343325	0.439916	-1382.8563	0.129438	-1382.979185	-1385.440646
**TS1**	-1383.327851	0.435623	-1382.846	0.126501	-1382.966669	-1385.41921
**TS1-c2**	-1383.322726	0.436062	-1382.8408	0.125248	-1382.960493	-1385.414883
**INT2**	-1383.366115	0.441226	-1382.8782	0.126819	-1382.999607	-1385.4595
**INT2-c2**	-1383.366115	0.441228	-1382.8782	0.126815	-1382.999606	-1385.459521
**INT3a**	-1383.320589	0.438974	-1382.8341	0.130558	-1382.957934	-1385.431006
**INT3**	-1383.344464	0.43888	-1382.8569	0.139031	-1382.985178	-1385.446155
**TS2**	-1383.338477	0.437974	-1382.853	0.135594	-1382.97858	-1385.439945
**INT4**	-1383.340493	0.437925	-1382.8539	0.138773	-1382.982176	-1385.441536
**TS3**	-1383.332195	0.43513	-1382.849	0.136164	-1382.975425	-1385.433272
**INT5**	-1383.338328	0.436855	-1382.8529	0.137179	-1382.980274	-1385.439261
**INT5-c2**	-1383.336579	0.437138	-1382.851	0.136232	-1382.977823	-1385.437325
**INT6**	-1383.30662	0.436092	-1382.8216	0.140235	-1382.950205	-1385.42623

**TS4**	-1383.282682	0.435416	-1382.7995	0.136458	-1382.925685	-1385.40426
**TS4-c2**	-1383.278811	0.434536	-1382.796	0.136282	-1382.922753	-1385.381705
**TS4’**	-1383.288948	0.435196	-1382.8054	0.138342	-1382.932889	-1385.389267
**INT7**	-1383.249681	0.436333	-1382.7656	0.134317	-1382.890737	-1385.411514
**INT7-c2**	-1383.286997	0.436392	-1382.8027	0.136092	-1382.92865	-1385.409455
**TS5**	-1383.290487	0.432471	-1382.8106	0.13477	-1382.935867	-1385.396015
**TS5-c2**	-1383.290714	0.432382	-1382.8113	0.129501	-1382.93409	-1385.389864
**TS5-c3**	-1383.231182	0.431733	-1382.7522	0.1333	-1382.876281	-1385.372786
**TS5-c4**	-1383.248007	0.431787	-1382.7689	0.1314	-1382.892672	-1385.348645
**INT8**	-1383.337455	0.437343	-1382.8523	0.133859	-1382.977575	-1385.432126
**INT8-c2**	-1383.329525	0.436405	-1382.8447	0.134037	-1382.970905	-1385.426338
**INT8-c3**	-1383.315704	0.436002	-1382.8315	0.137518	-1382.95882	-1385.422172
**INT9**	-1383.290498	0.432857	-1382.8092	0.137526	-1382.936634	-1385.39617

**INT9-c2**	-1383.293609	0.433447	-1382.8125	0.131029	-1382.936539	-1385.393111
**INT10**	-1440.48886	0.25374	-1440.1936	0.123322	-1440.309722	-1442.151878
**INT10’**	-883.602395	0.23426	-883.33769	0.096314	-883.429468	-884.804197
**INT11**	-1439.307803	0.239055	-1439.0279	0.124309	-1439.143945	-1440.970405
**INT11’**	-1672.150065	0.300931	-1671.8028	0.13505	-1671.927464	-1674.704568
**INT11’-c2**	-1672.138611	0.30038	-1671.7916	0.134421	-1671.916467	-1674.687228
**TS2’’-ac**	-1018.755777	0.270425	-1018.4539	0.100356	-1018.547966	-1020.185855
**INT3’’**	-1383.349947	0.439982	-1382.8622	0.135325	-1382.987826	-1385.448716
**TS2’’**	-1383.279908	0.438468	-1382.7939	0.136725	-1382.91985	-1385.381637
**INT4’’**	-1383.325912	0.441611	-1382.8366	0.137826	-1382.963234	-1385.425169
**TS3a**	-1383.276909	0.438712	-1382.7903	0.137402	-1382.91709	-1385.379846
**INT5a**	-1383.2926	0.441924	-1382.8026	0.139008	-1382.93027	-1385.433396
**TS3b**	-1383.262465	0.438685	-1382.7766	0.131414	-1382.900103	-1385.370109

**INT5b**	-1383.316429	0.441099	-1382.8278	0.134185	-1382.953082	-1385.425482

9.11. References:

Full reference Gaussian 16:

Gaussian 16, Revision B.01, Frisch, M. J.; Trucks, G. W.; Schlegel, H. B.; Scuseria, G. E.; Robb, M. A.; Cheeseman, J. R.; Scalmani, G.; Barone, V.; Mennucci, B.; Petersson, G. A.; Nakatsuji, H.; Caricato, M.; Li, X.; Hratchian, H. P.; Izmaylov, A. F.; Bloino, J.; Zheng, G.; Sonnenberg, J. L.; Hada, M.; Ehara, M.; Toyota, K.; Fukuda, R.; Hasegawa, J.; Ishida, M.; Nakajima, T.; Honda, Y.; Kitao, O.; Nakai, H.; Vreven, T.; Montgomery Jr., J. A.; Peralta, J. E.; Ogliaro, F.; Bearpark, M.; Heyd, J. J.; Brothers, E.; Kudin, K. N.; Staroverov, V. N.; Kobayashi, R.; Normand, J.; Raghavachari, K.; Rendell, A.; Burant, J. C.; Iyengar, S. S.; Tomasi, J.; Cossi, M.; Rega, N.; Millam, J. M.; Klene, M.; Knox, J. E.; Cross, J. B.; Bakken, V.; Adamo, C.; Jaramillo, J.; Gomperts, R.; Stratmann, R. E.; Yazyev, O.; Austin, A. J.; Cammi, R.; Pomelli, C.; Ochterski, J. W.; Martin, R. L.; Morokuma, K.; Zakrzewski, V. G.; Voth, G. A.; Salvador, P.; Dannenberg, J. J.; Dapprich, S.; Daniels, A. D.; Farkas, Ö.; Foresman, J. B.; Ortiz, J. V; Cioslowski, J.; Fox, D. J. Gaussian, Inc., Wallingford CT, 2016.

(1) Frisch, M. J. .; Trucks, G. W. .; Schlegel, H. B. .; Scuseria, G. E. .; Robb, M. A. .; Cheeseman, J. R. .; Scalmani, G. .; Barone, V. .; Petersson, G. A. .; Nakatsuji, H. .; et al. Gaussian 16, Revision B.01. 2016.

(2) Yu, H. S.; He, X.; Li, S. L.; Truhlar, D. G. MN15: A Kohn–Sham Global-Hybrid Exchange–Correlation Density Functional with Broad Accuracy for Multi-Reference and Single-Reference Systems and Noncovalent Interactions. *Chem. Sci.* **2016**, *7* (8), 5032–5051.

(3) Weigend, F.; Ahlrichs, R. Balanced Basis Sets of Split Valence, Triple Zeta Valence and Quadruple Zeta Valence Quality for H to Rn: Design and Assessment of Accuracy. *Phys. Chem. Chem. Phys.* **2005**, *7* (18), 3297–3305.

(4) Weigend, F. Accurate Coulomb-Fitting Basis Sets for H to Rn. *Phys. Chem. Chem. Phys.* **2006**, *8* (9), 1057–1065.

(5) Rappoport, D.; Furche, F. Property-Optimized Gaussian Basis Sets for Molecular

Response Calculations. *J. Chem. Phys.* **2010**, *133* (13), 134105.

(6) Andrae, D.; Häußermann, U.; Dolg, M.; Stoll, H.; Preuß, H. Energy-Adjustedab Initio Pseudopotentials for the Second and Third Row Transition Elements. *Theor. Chim. Acta* **1990**, *77* (2), 123–141.

(7) Fukui, K. Formulation of the Reaction Coordinate. *J. Phys. Chem.* **2005**, *74* (23), 4161–4163.

(8) Fukui, K. The Path of Chemical Reactions - The IRC Approach. *Acc. Chem. Res.* **1981**, *14* (12), 363–368.

(9) Marenich, A. V.; Cramer, C. J.; Truhlar, D. G. Universal Solvation Model Based on Solute Electron Density and on a Continuum Model of the Solvent Defined by the Bulk Dielectric Constant and Atomic Surface Tensions. *J. Phys. Chem. B* **2009**, *113* (18), 6378–6396.

(10) Eberson, L.; Hartshorn, M. P.; Persson, O.; Radner, F. Making Radical Cations Live Longer. *Chem. Commun.* **1996**, No. 18, 2105–2112.

(11) Gu, X.; Song, X.; Shao, C.; Zeng, P.; Lu, X.; Shen, X.; Yang, Q. Electrospinning of Poly(Butylene-Carbonate): Effect of Solvents on the Properties of the Nanofibers Film. *Int. J. Electrochem. Sci.* **2014**, *9* (12), 8045–8056.

(12) Carraro, M.; Gardan, M.; Sartorel, A.; Maccato, C.; Bonchio, M. Hydrogen Peroxide Activation by Fluorophilic Polyoxotungstates for Fast and Selective Oxygen Transfer Catalysis. *Dalt. Trans.* **2016**, *45* (37), 14544–14548.

(13) Sigma-Aldrich. 1,1,1,3,3,3-Hexafluoro-2-propanol https://www.sigmaaldrich.com/catalog/product/aldrich/105228 (accessed Jun 6, 2017).

(14) Abraham, M. H.; Andonian-Haftvan, J.; Whiting, G. S.; Leo, A.; Taft, R. S. Hydrogen Bonding. Part 34. The Factors That Influence the Solubility of Gases and Vapours in Water at 298 K, and a New Method for Its Determination. *J. Chem. Soc. Perkin Trans. 2* **1994**, No. 8, 1777–1791.

(15) Richmond, E.; Yi, J.; Vuković, V. D.; Sajadi, F.; Rowley, C. N.; Moran, J. Ring-Opening Hydroarylation of Monosubstituted Cyclopropanes Enabled by Hexafluoroisopropanol. *Chem. Sci.* **2018**, *9* (30), 6411–6416.

(16) Berkessel, A.; Adrio, J. A. Dramatic Acceleration of Olefin Epoxidation in Fluorinated

Alcohols: Activation of Hydrogen Peroxide by Multiple H-Bond Networks. *J. Am. Chem. Soc.* **2006**, *128* (41), 13412–13420.

(17) Winget, P.; Dolney, D. M.; Giesen, D. J.; Cramer, C. J.; Truhlar, D. G. Minnesota solvent descriptor database https://comp.chem.umn.edu/solvation/mnsddb.pdf (accessed Mar 15, 2022).

(18) Grimme, S. Supramolecular Binding Thermodynamics by Dispersion-Corrected Density Functional Theory. *Chem.: Eur. J.* **2012**, *18* (32), 9955–9964.

(19) Funes-Ardoiz, I.; Paton, R. S. GoodVibes v1.0.1 http://doi.org/10.5281/zenodo.56091.

(20) Schrödinger, L. *The PyMOL Molecular Graphics Development Component, Version 1.8*; 2015.

(21) Chen, G.; Gong, W.; Zhuang, Z.; Andrä, M. S.; Chen, Y. Q.; Hong, X.; Yang, Y. F.; Liu, T.; Houk, K. N.; Yu, J. Q. Ligand-Accelerated Enantioselective Methylene C(Sp^3)-H Bond Activation. *Science* **2016**, *353* (6303), 1023–1027.

(22) Dutta, U.; Modak, A.; Bhaskararao, B.; Bera, M.; Bag, S.; Mondal, A.; Lupton, D. W.; Sunoj, R. B.; Maiti, D. Catalytic Arene Meta-C–H Functionalization Exploiting a Quinoline-Based Template. *ACS Catal.* **2017**, *7* (5), 3162–3168.

(23) Cheng, G. J.; Yang, Y. F.; Liu, P.; Chen, P.; Sun, T. Y.; Li, G.; Zhang, X.; Houk, K. N.; Yu, J. Q.; Wu, Y. D. Role of N-Acyl Amino Acid Ligands in Pd(II)-Catalyzed Remote C–H Activation of Tethered Arenes. *J. Am. Chem. Soc.* **2014**, *136* (3), 894–897.

(24) Yang, Y. F.; Hong, X.; Yu, J. Q.; Houk, K. N. Experimental-Computational Synergy for Selective Pd(II)-Catalyzed C–H Activation of Aryl and Alkyl Groups. *Acc. Chem. Res.* **2017**, *50* (11), 2853–2860.

(25) Achar, T. K. T. K.; Zhang, X.; Mondal, R.; Shanavas, M. S. S.; Maiti, S.; Maity, S.; Pal, N.; Paton, R. S. R. S.; Maiti, D. Palladium-Catalyzed Directed *Meta*-Selective C–H Allylation of Arenes: Unactivated Internal Olefins as Allyl Surrogates. *Angew. Chem. Int. Ed.* **2019**, *58* (30), 10353–10360.

(26) Porey, S.; Zhang, X.; Bhowmick, S.; Kumar Singh, V.; Guin, S.; Paton, R. S. R. S.; Maiti, D. Alkyne Linchpin Strategy for Drug:Pharmacophore Conjugation: Experimental and Computational Realization of a *Meta*-Selective Inverse Sonogashira

Coupling. *J. Am. Chem. Soc.* **2020**, *142* (8), 3762–3774.
(27) Kozuch, S.; Shaik, S. How to Conceptualize Catalytic Cycles? The Energetic Span Model. *Acc. Chem. Res.* **2011**, *44* (2), 101–110.

10. Deuterium Exchange Experiment:

In an oven-dried screw capped reaction tube was charged with magnetic stir-bar, corresponding acid (0.1 mmol), $Pd(OAc)_2$ (10 mol%), *N*-Ac-^{t}Leu (20 mol%), Ag_2CO_3 (2 equiv.), and Na_3PO_4 (2 equiv.) in 1 mL of deuterated version of 1,1,1,3,3,3-hexafluoro-2-propanol (d^2-HFIP) were added. The reaction tube was capped and placed in a preheated bath at 120 °C with stirring (800 rpm) for 24 h. Upon completion the mixture was diluted with EtOAc and filtered through a celite pad. The filtrate was evaporated under reduced pressure and NMR of the crude mixture was taken. *γ*-Methyl group was found to be 59% deuterated and *γ*-methylene 45% deuterated.

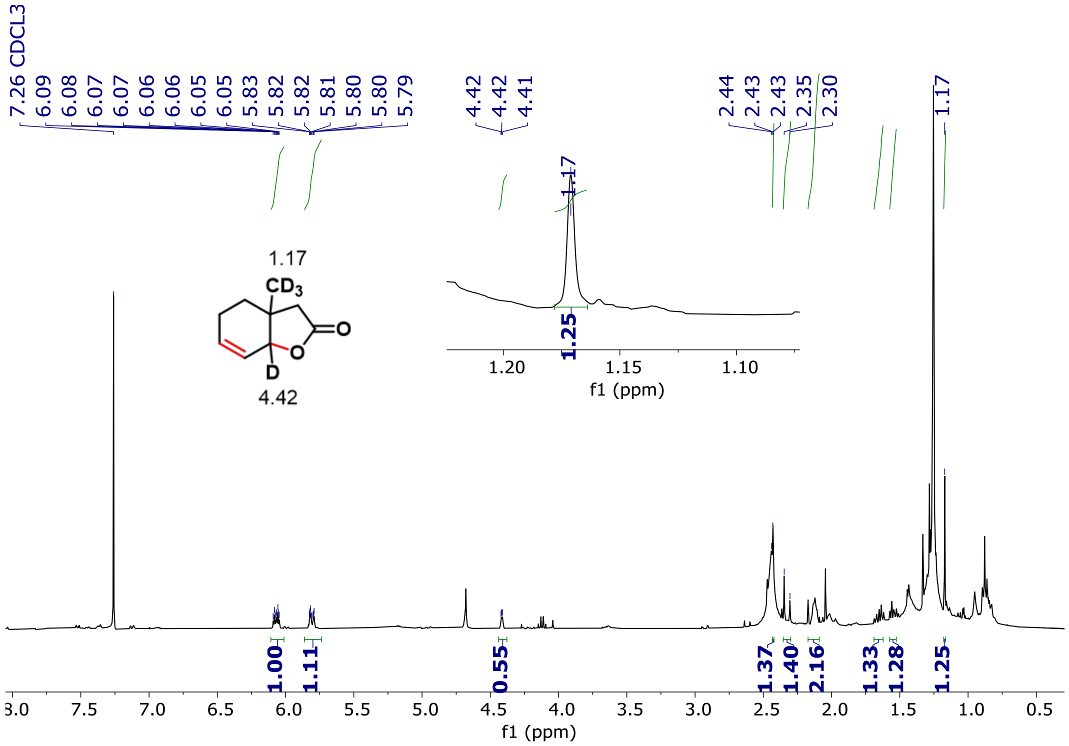

Figure S15. ^{1}H NMR spectra of product performed in d^2-HFIP.

Chemical competence of the alkenoic acid 5f.

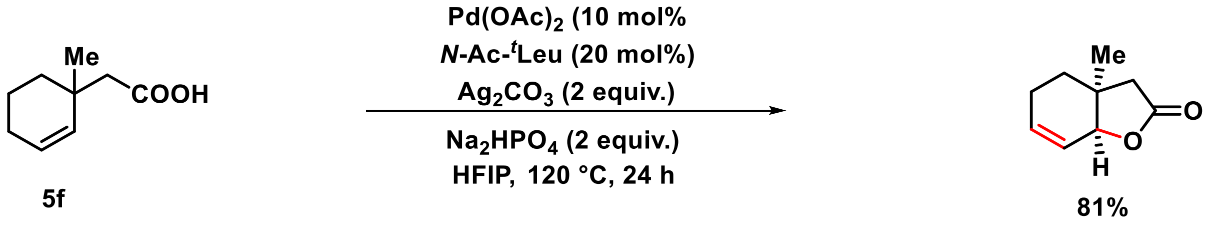

In an oven-dried screw capped reaction tube was charged with magnetic stir-bar, corresponding alkenoic acid (0.2 mmol), $Pd(OAc)_2$ (10 mol%), *N*-Ac-^{t}Leu (20 mol%), Ag_2CO_3 (2 equiv.), and Na_3PO_4 (2 equiv.) in 1.5 mL of 1,1,1,3,3,3-hexafluoro-2-propanol (HFIP) were added. The reaction tube was capped and placed in a preheated bath at 120 °C with stirring (800 rpm) for 24 h. Upon completion the mixture was diluted with EtOAc and filtered through a celite pad. The filtrate was evaporated under reduced pressure and the crude mixture was purified by column chromatography using silica (100-200 mesh size) and petroleum ether/ ethyl acetate as the eluent.

11. Headspace Analysis:

Following the general procedure, after 24 h, the gas phase over the reaction mixture was analysed by headspace GC analysis using an Nucon 5700 gas chromatography. Argon used as carrier gas (pressure 15 psi) and the sample was analysed by a temperature conductivity detector at 40 °C (injected temperature 40 °C, detector temperature 40 °C, oven temperature 40 °C, current 120 mA). For comparison a blank sample of the carrier gas was conducted.

Sample Name: DM-JD-CY-BLANK
Detector: FID
System: GC
Run Date: 14-05-2022
Analysis Type: Percent Method
Time: 06:54:36
Chan No: Chan 1

Peak Width	Peak Threshold	Area Reject	Height Reject
16	60	500	60

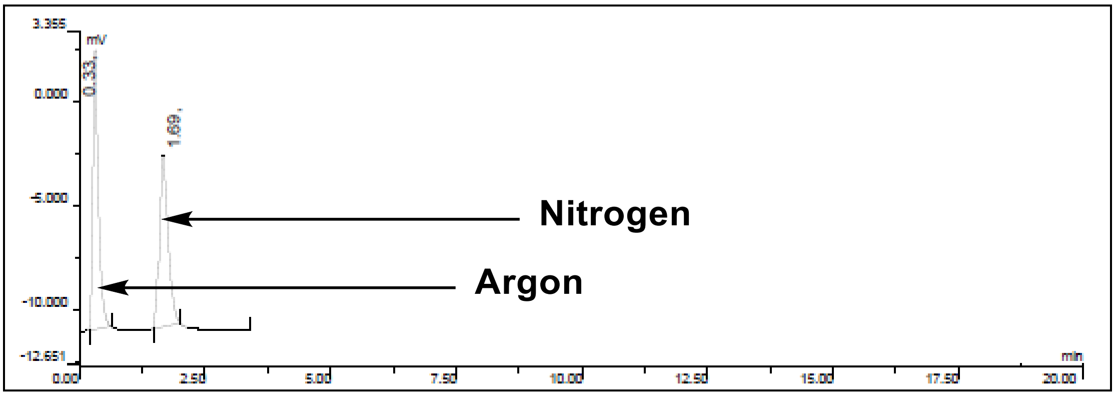

AREA%, HEIGHT %

Sr. No.	Component Name	Ret. Time.	Area µ Volt Sec	Area %	Height µ Volt	Height %	Type	ID Type
1		0.33	102837.7143	51.18	13360	62.03	BB	U
2		1.69	98083.4286	48.82	8177	37.97	BB	U
			200921.1429	100.00	21537	100.00		

SUMMARY

Total Peaks : 2
Multiplication Factor : 1.0000
Sample Amount : 100.0000
Dilution Factor : 1.0000
Syssuit Standards : IP
ISTD Amount : 0.0

Figure S16. Headspace analysis of the blank.

Sample Name: DM-JD-CY-REACTION
Detector: FID
System: GC
Run Date: 14-05-2022

Analysis Type: Percent Method
Time: 04:55:25
Chan No: Chan 1

Peak Width	Peak Threshold	Area Reject	Height Reject
16	60	500	60

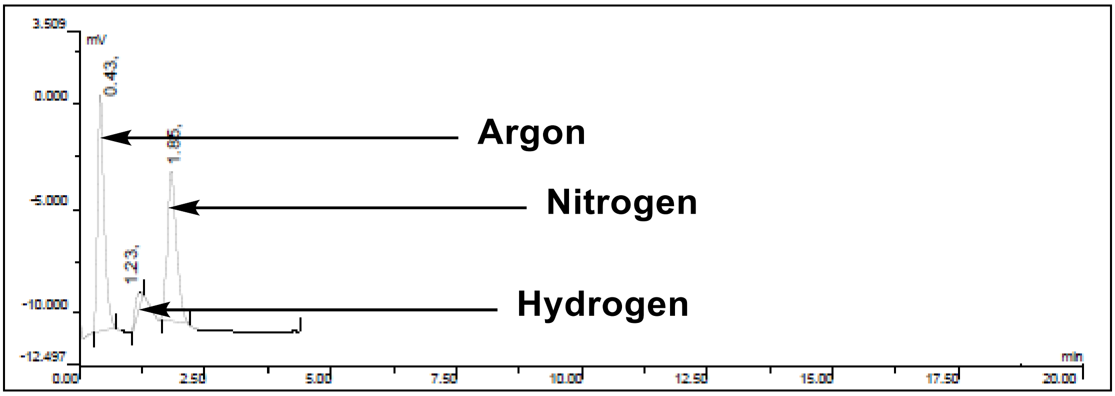

AREA%, HEIGHT %

Sr. No.	Component Name	Ret. Time.	Area µ Volt Sec	Area %	Height µ Volt	Height %	Type	ID Type
1		0.43	101479.6000	51.13	11302	59.81	BB	U
2		1.23	5409.2571	2.73	422	2.24	BB	U
3		1.85	91572.7429	46.14	7171	37.95	BB	U
			**198461.6000**	**100.00**	**18897**	**100.00**		

SUMMARY

Total Peaks : 3
Multiplication Factor : 1.0000
Sample Amount : 100.0000
Dilution Factor : 1.0000
Syssuit Standards : IP
ISTD Amount : 0.0

Figure S17. Headspace analysis of the reaction mixture.

12.1. Proposed catalytic cycle for the unsaturated bicyclic lactone formation

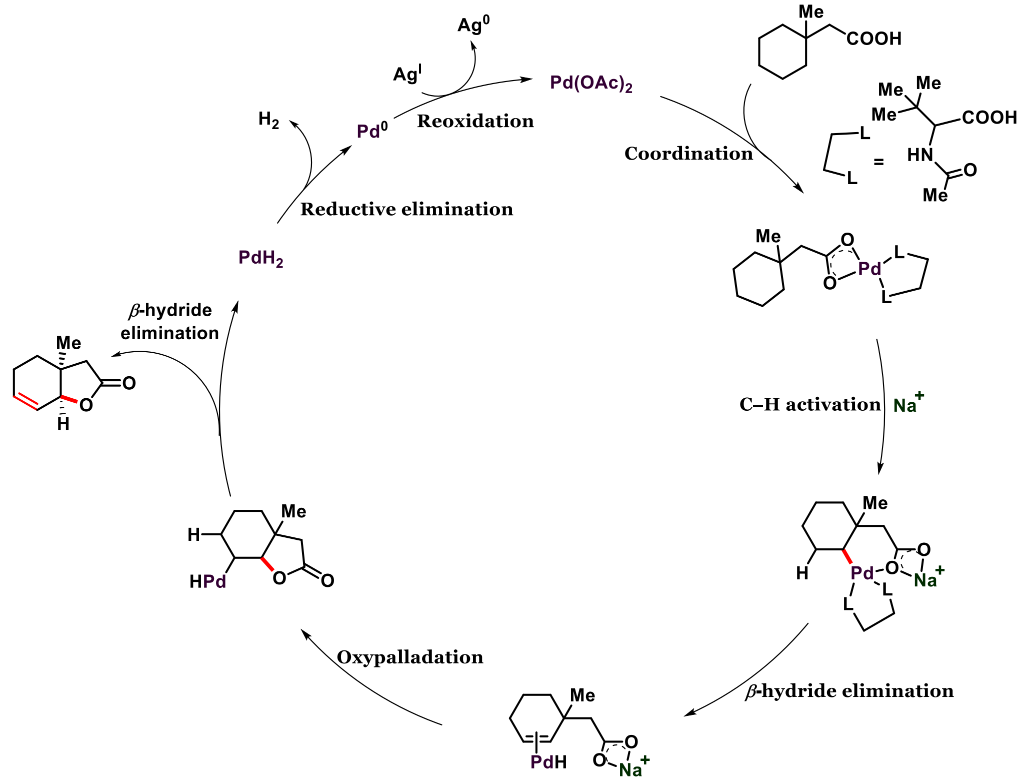

12.2. Proposed catalytic cycle for the unsaturated olefin containing bicyclic lactone formation

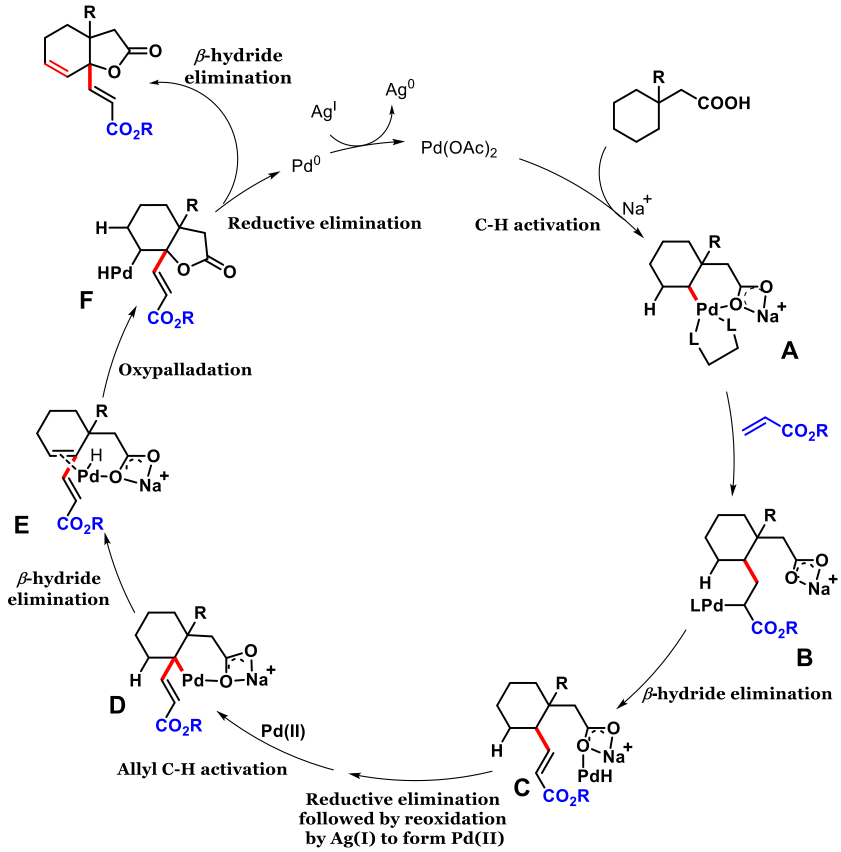

Control experiment:

In an oven-dried screw capped reaction tube was charged with magnetic stir-bar, corresponding alkenoic acid (**5f**, 0.2 mmol), $Pd(OAc)_2$ (10 mol%), *N*-Ac-^{t}Leu (20 mol%), Ag_2CO_3 (2 equiv.), and Na_3PO_4 (2 equiv.) in 1.5 mL of 1,1,1,3,3,3-hexafluoro-2-propanol (HFIP) were added. The reaction tube was capped and placed in a preheated bath at 110 °C with stirring (800 rpm) for 24 h. Upon completion the mixture was diluted with EtOAc and filtered through a celite pad. The filtrate was evaporated under reduced pressure and the crude mixture was purified by column chromatography using silica (100-200 mesh size) and petroleum ether/ ethyl acetate as the eluent.

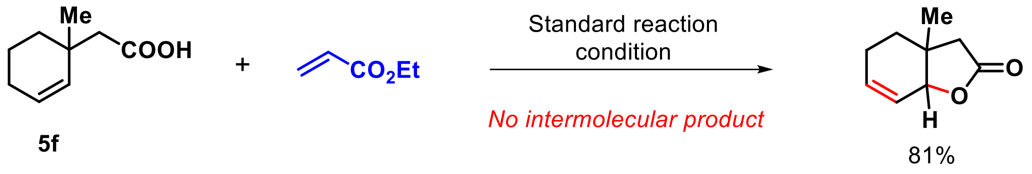

This control experiment implies that in presence of an olefin, the activated intermediate **A** undergo olefin insertion not *β*-hydride elimination as shown in abovementioned mechanism. Had *β*-hydride elimination occurs before olefin insertion; above experiment should have provided the intermolecular product and not intramolecular product. Thus, alkenoic acid **5f** most likely is not an intermediate for the intermolecular product formation mechanism and allyl activation is likely occurring in this case.

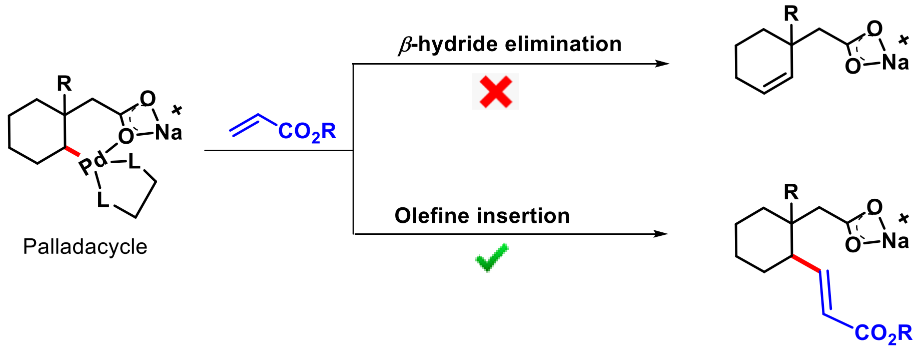

A similar report involving allyl activation of acid has been recently reported in literature.[27]

Mass spectral analysis of intermediate

A Clean, oven-dried screw cap reaction tube was charged with a magnetic stir-bar, 2-(1-methylcyclohexyl)acetic acid (15 mg, 0.1 mmol) $Pd(OAc)_2$ (1.2 mg, 10 mol%), *N*-Ac-Gly-OH (2.3 mg, 20 mol%)), Ag_2CO_3 (55 mg, 2 equiv.), Na_2HPO_4 (28 mg, 2 equiv.) and ethyl acrylate (32 µL, 2 equiv.) Then 1 mL of 1,1,1,3,3,3-hexafluoro-2-propanol (HFIP) was added. The reaction tube was screwed by a cap fitted with a rubber septum. The reaction mixture was

stirred vigorously on a preheated oil bath at 110 ˚C for 8 h. The crude reaction mixture was subjected to mass analysis.

ESI-MS of the reaction mixture

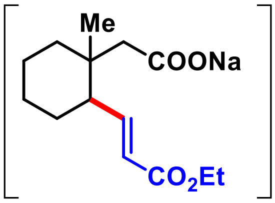

Calculated Mass = 277.1410
Observed Mass = 277.1414

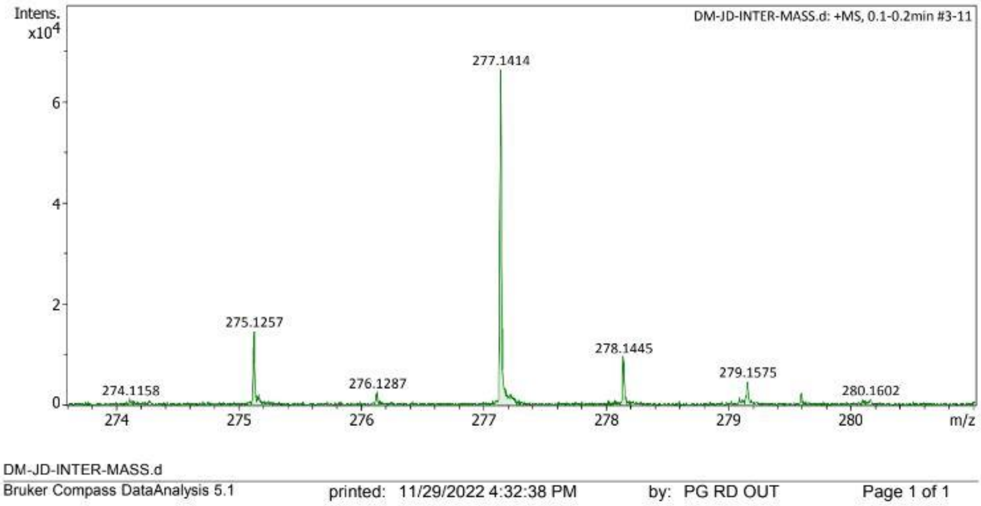

Figure S18. ESI-MS spectra of the reaction mixture showing γ-olefinated intermediate mass.

Reference:

27. Das, J.; Pal, T.; Ali, W.; Sahoo, S. K.; Maiti. D. Pd-Catalyzed Dual-*γ*-1,1-C(sp^3)−H Activation of Free Aliphatic Acids with Allyl−O Moieties. *ACS. Catal*. **2022**, *12*, 11169-11176.

14. NMR Spectra of the Starting Material:

2-(1-Methylcyclohexyl)acetic acid (1)

^1H NMR (400 MHz, $CDCl_3$)

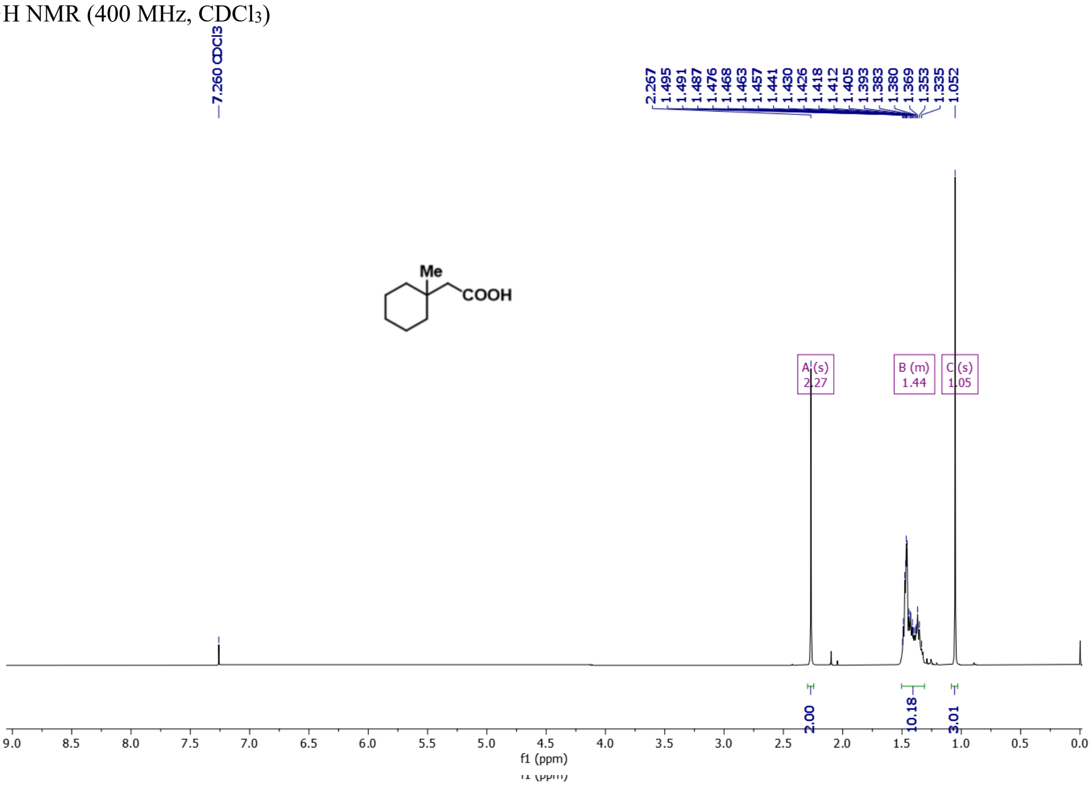

^{13}C NMR (101 MHz, $CDCl_3$)

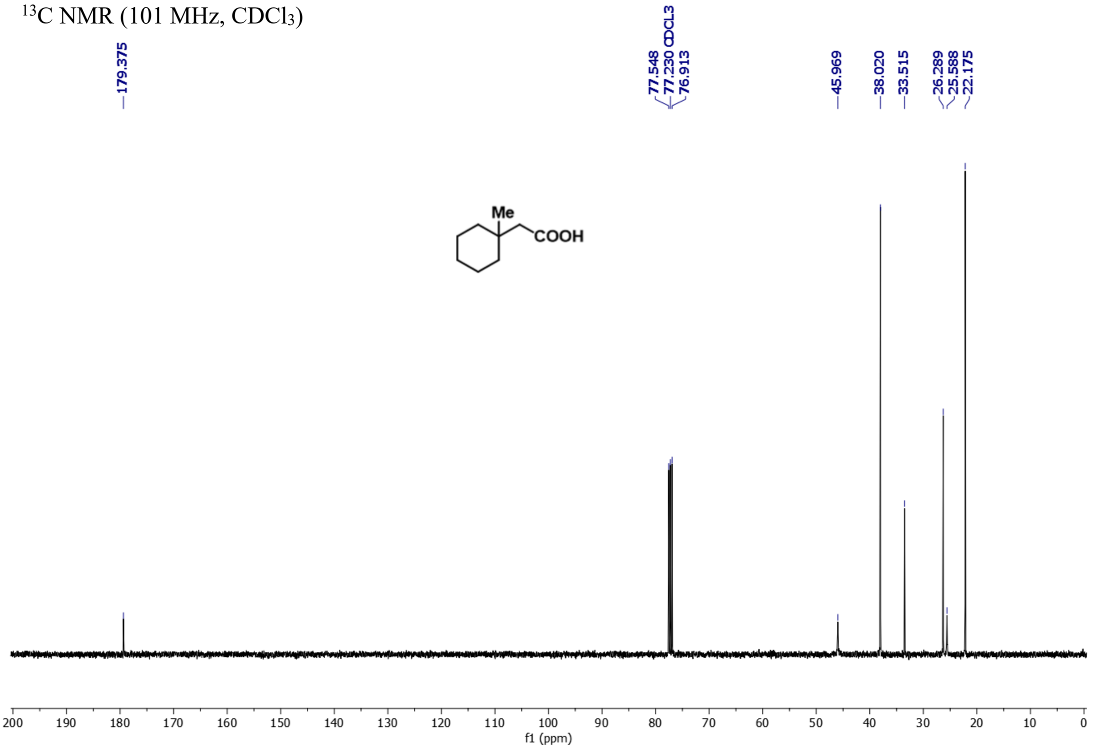

2-(1-Methylcyclopentyl)acetic acid (2)

^{1}H NMR (400 MHz, $CDCl_3$)

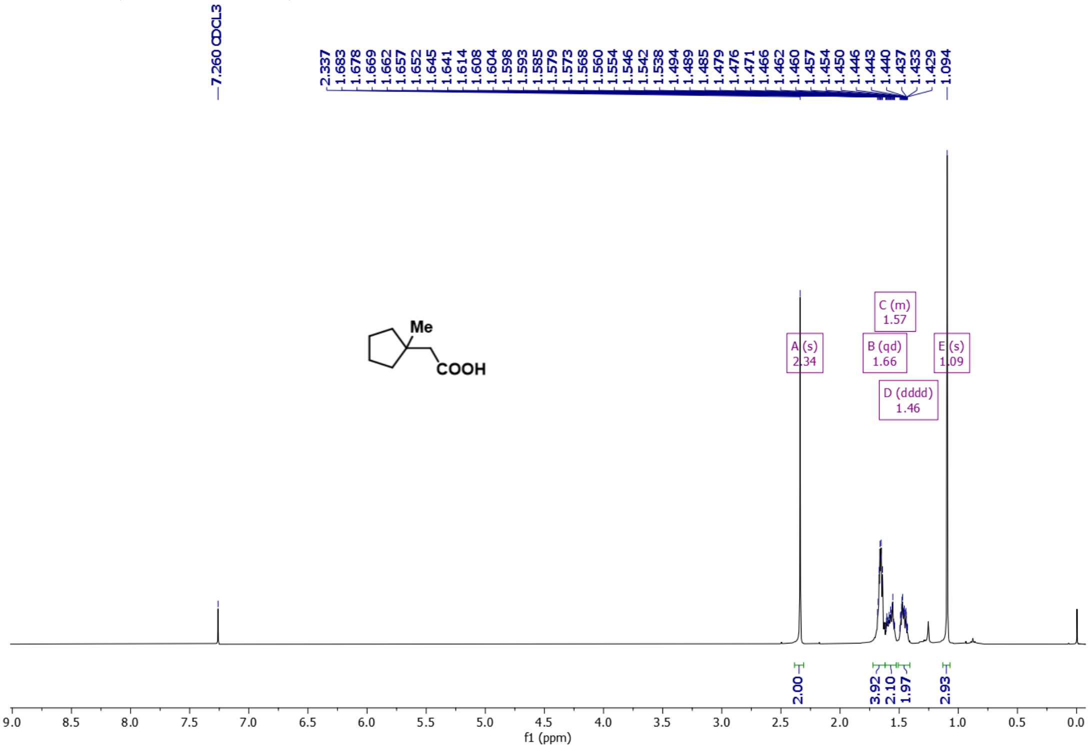

^{13}C NMR (101 MHz, $CDCl_3$)

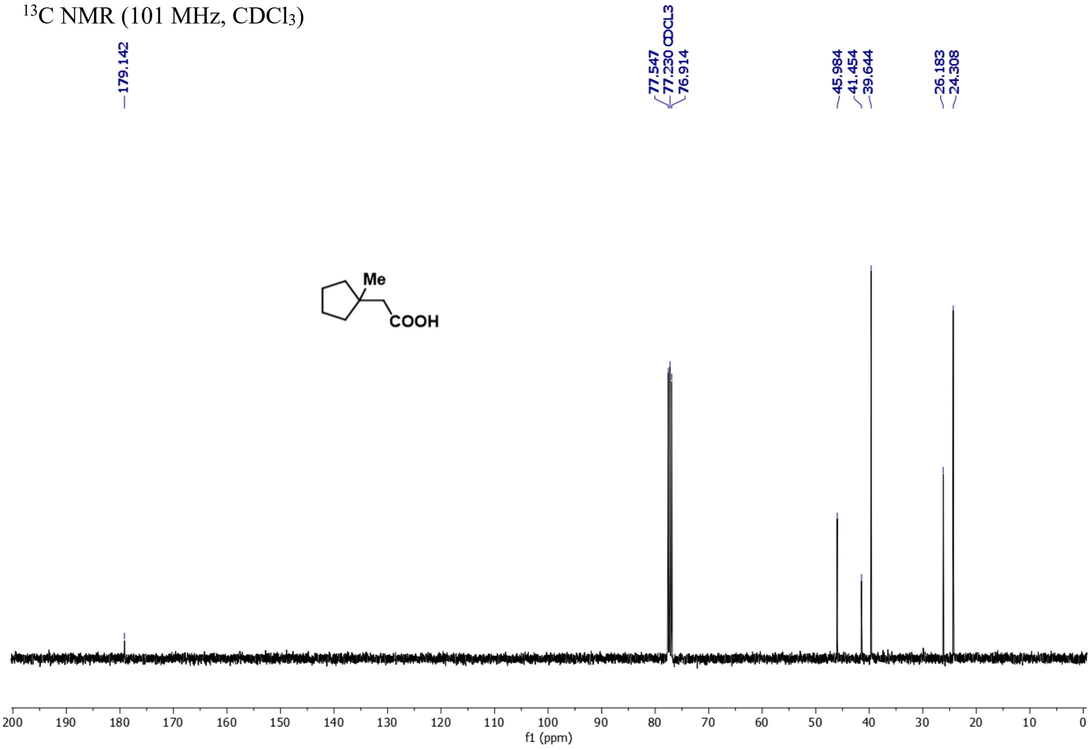

2-(1,2-Dimethylcyclohexyl)acetic acid (3)

^{1}H NMR (400 MHz, $CDCl_3$)

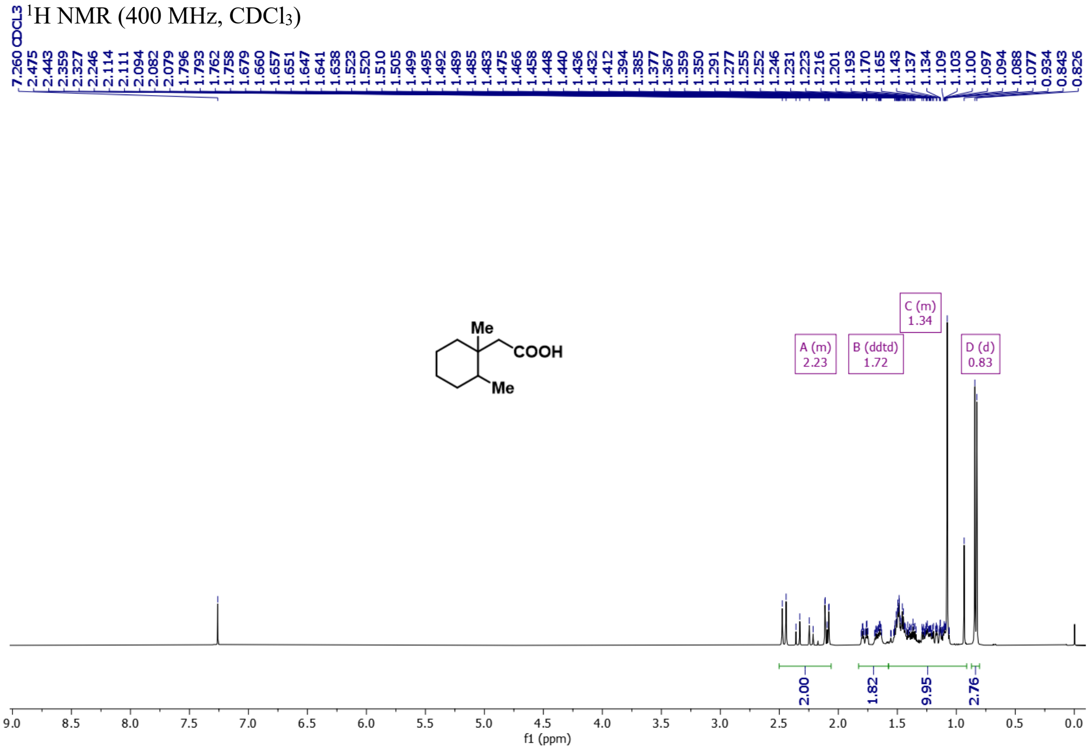

^{13}C NMR (126 MHz, $CDCl_3$)

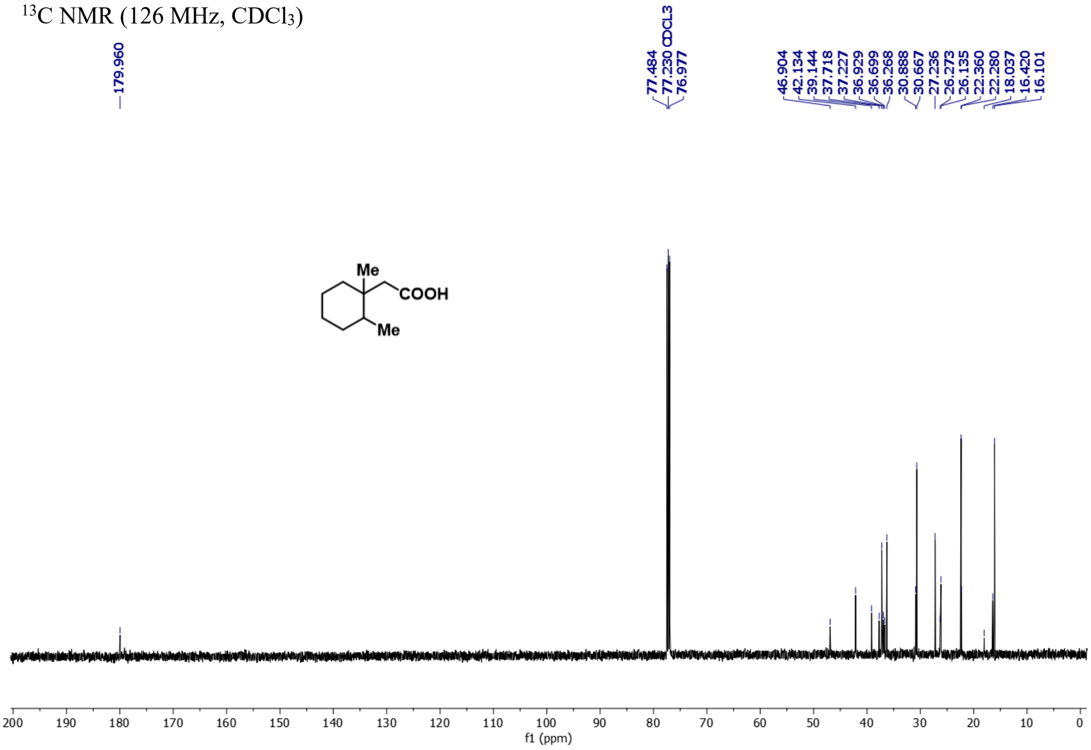

2-(1,3-Dimethylcyclohexyl)acetic acid (4)

^{1}H NMR (400 MHz, $CDCl_3$)

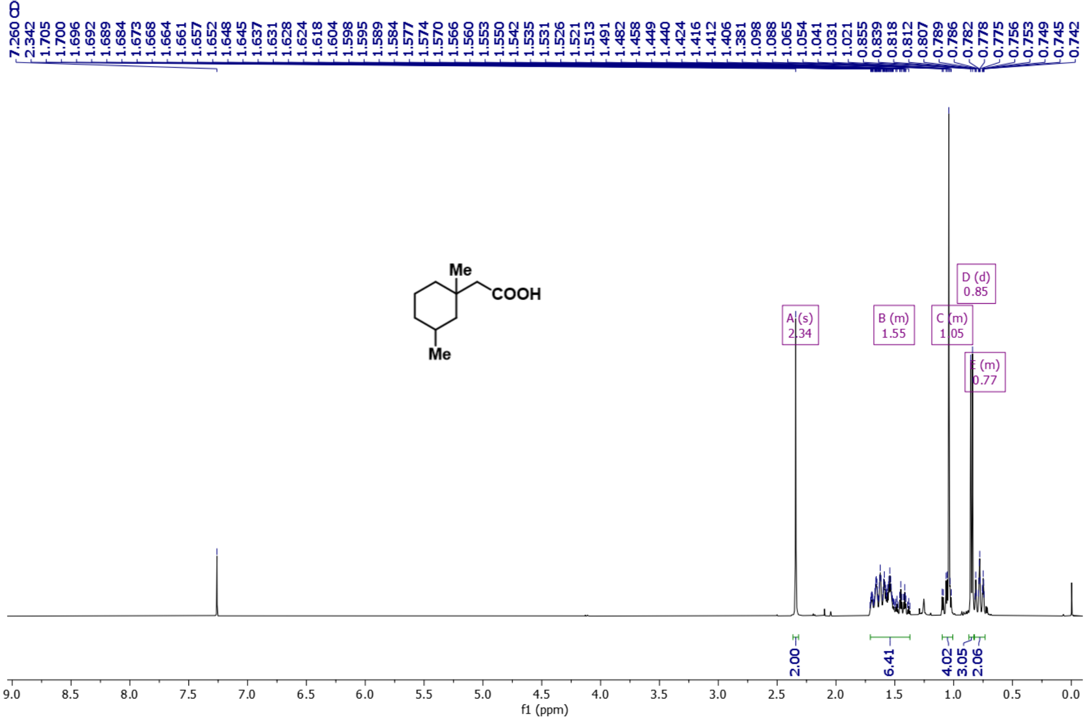

^{13}C NMR (101 MHz, $CDCl_3$)

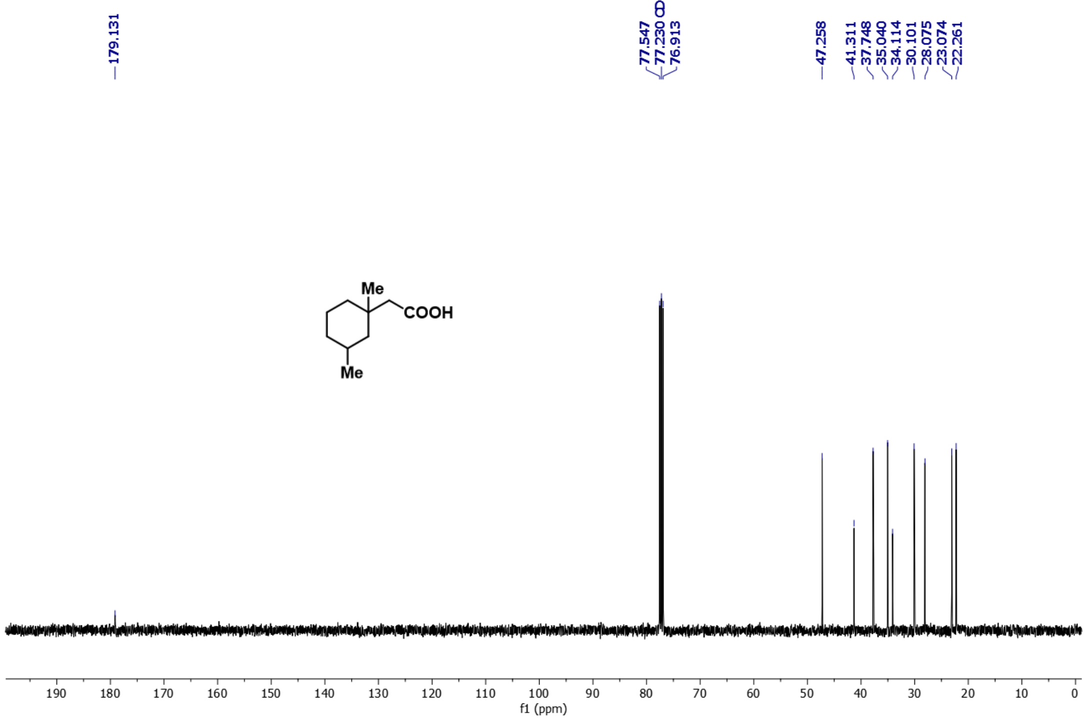

2-(1-Ethylcyclohexyl)acetic acid (5)

^{1}H NMR (400 MHz, $CDCl_3$)

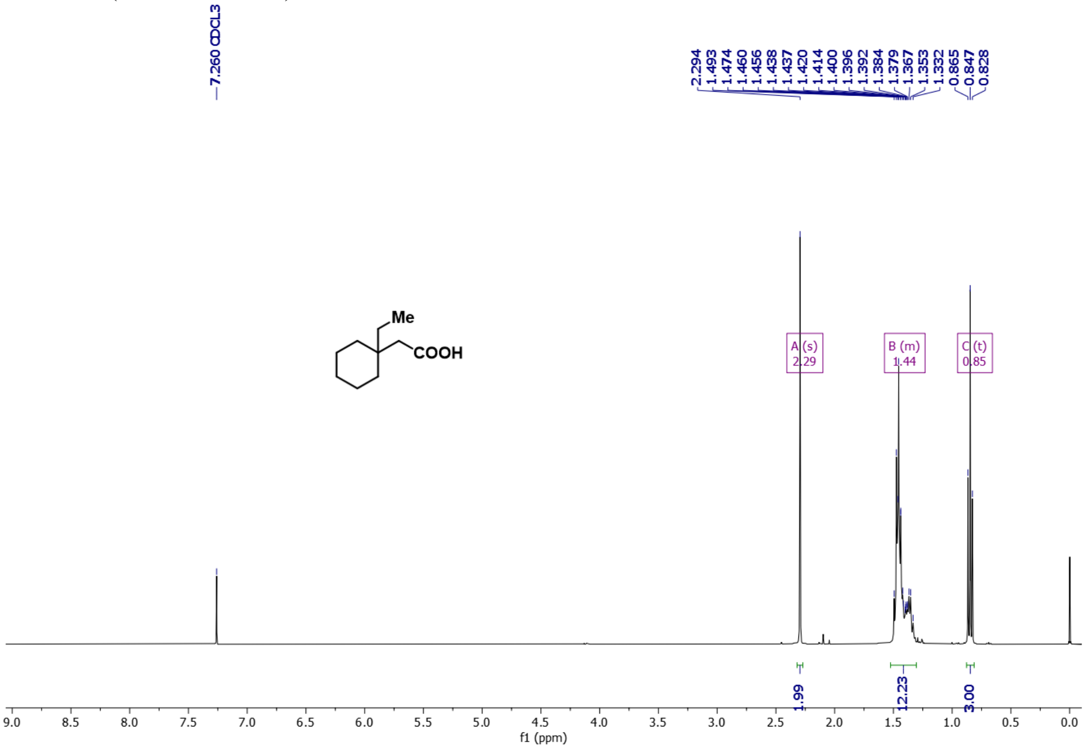

^{13}C NMR (101 MHz, $CDCl_3$)

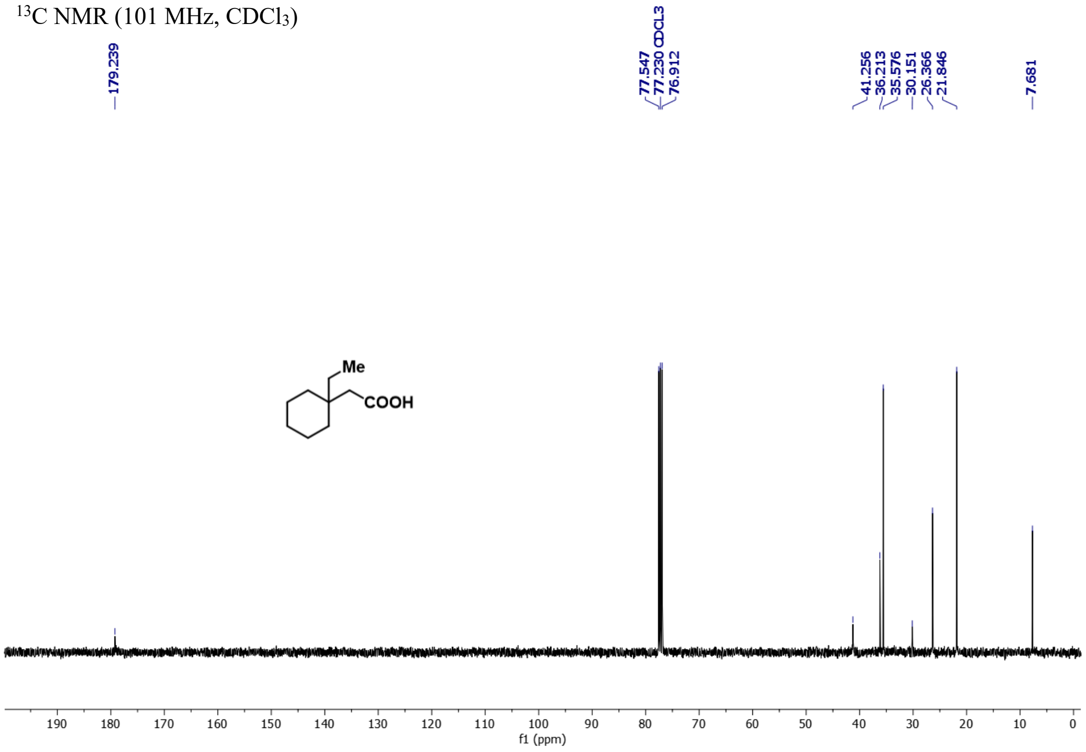

2-(1-Methylcycloheptyl)acetic acid (6)

^{1}H NMR (500 MHz, $CDCl_3$)

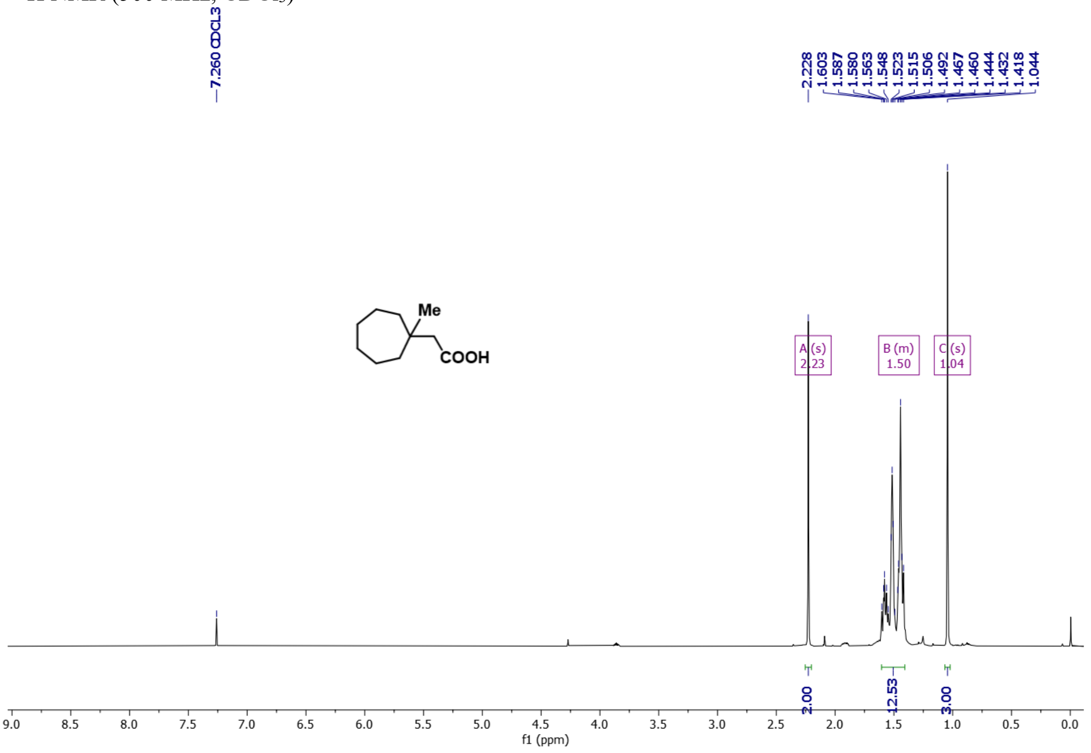

^{13}C NMR (126 MHz, $CDCl_3$)

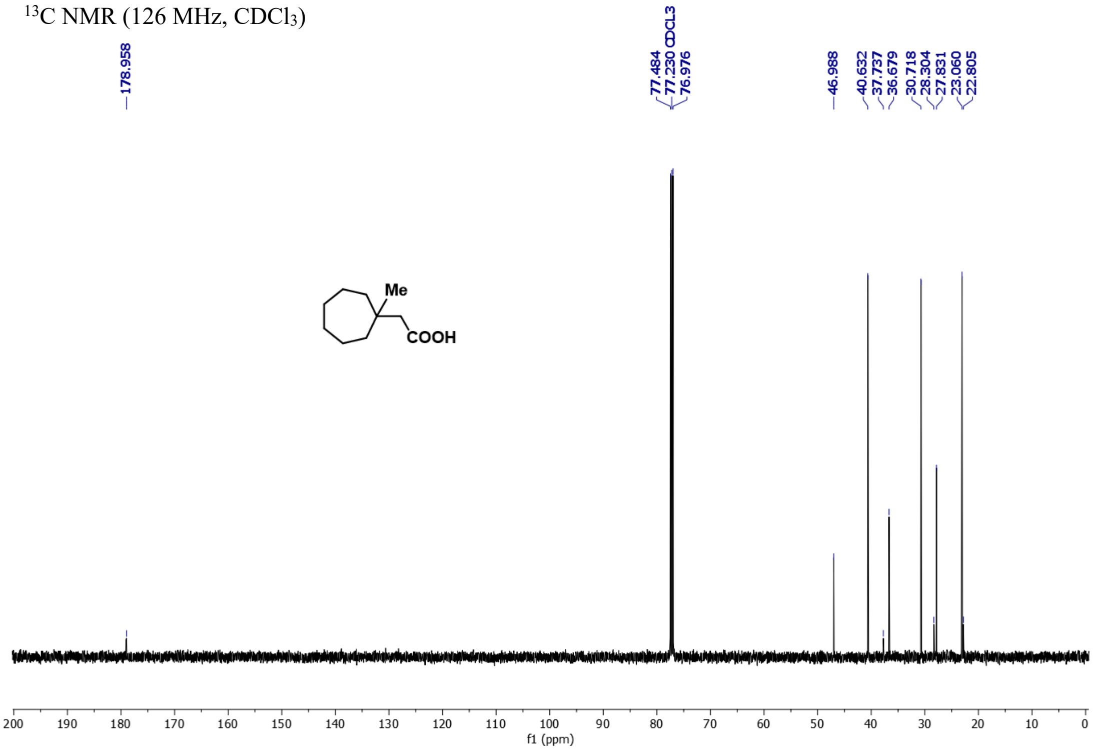

2-(1-Ethyl-3-methylcyclohexyl)acetic acid (7)

^1H NMR (400 MHz, $CDCl_3$)

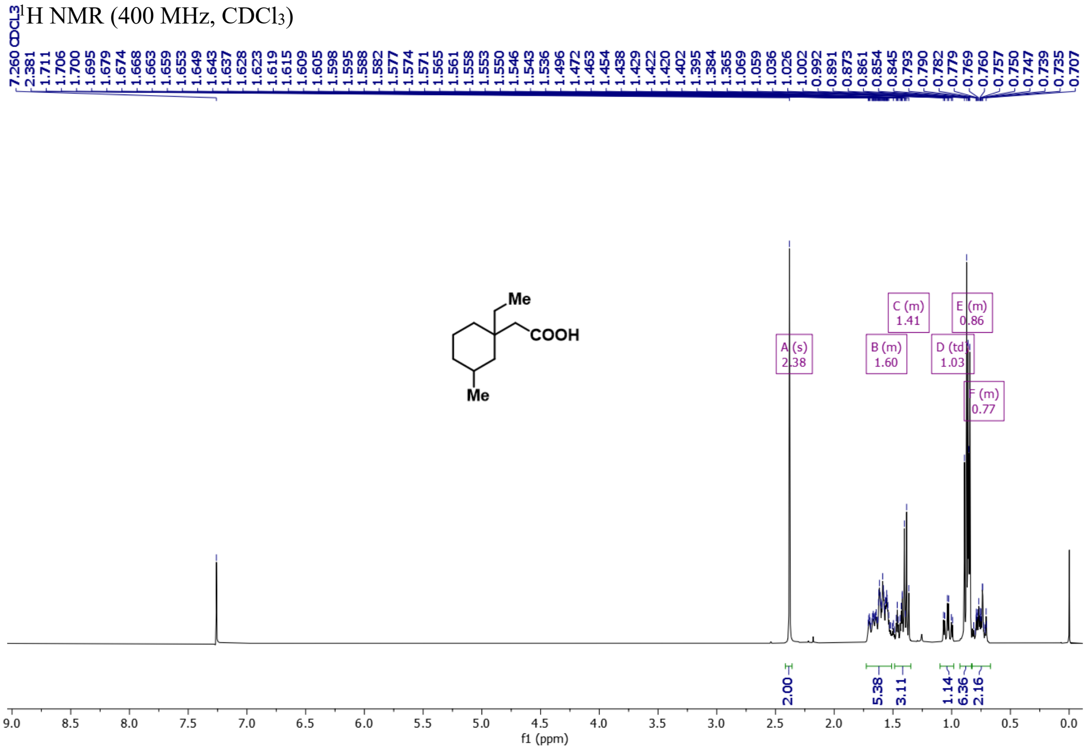

^{13}C NMR (101 MHz, $CDCl_3$)

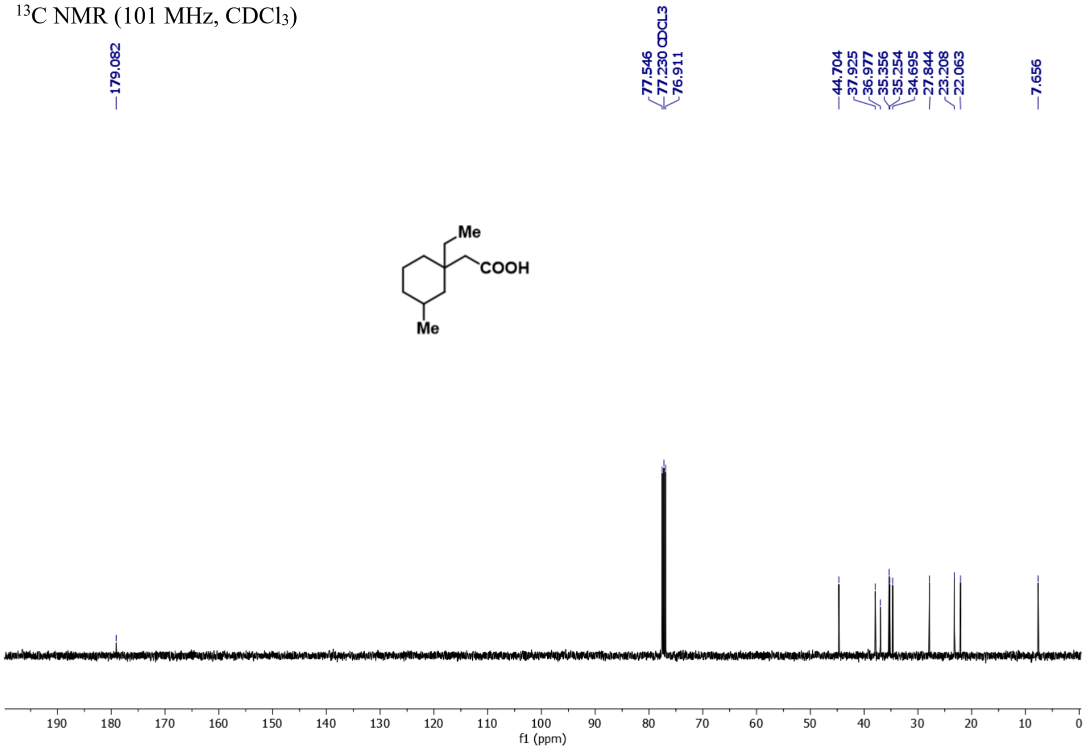

2-(1-Methyl-4-(*tert*-pentyl)cyclohexyl)acetic acid (8)

^{1}H NMR (500 MHz, $CDCl_3$)

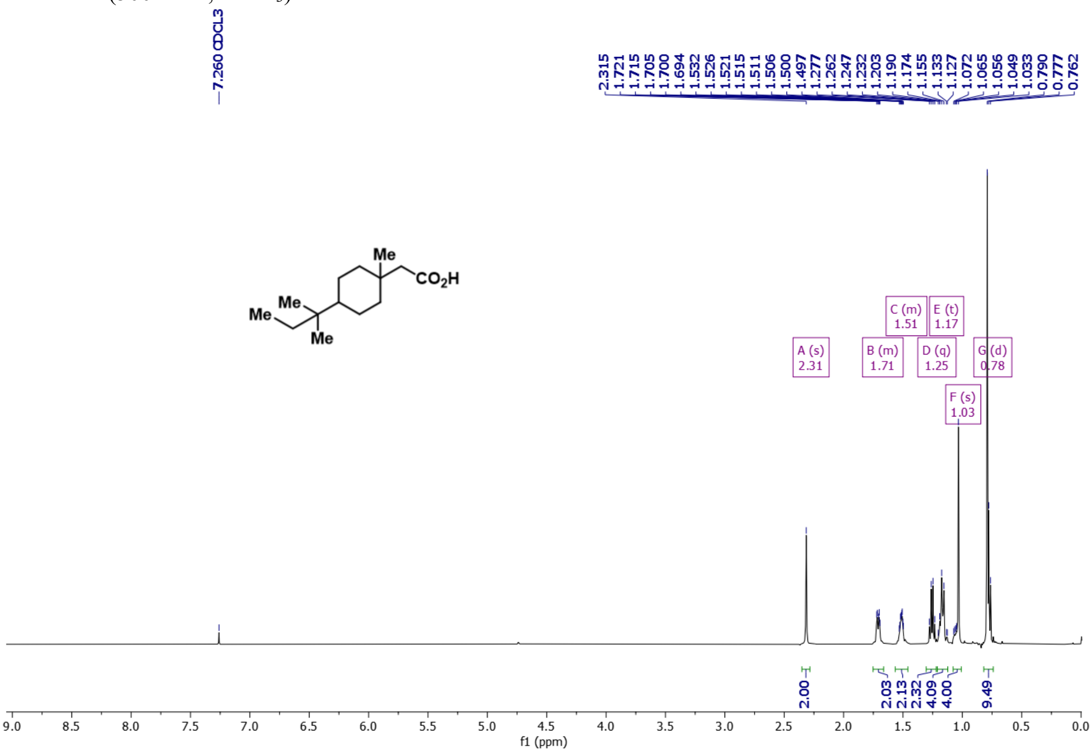

^{13}C NMR (101 MHz, $CDCl_3$)

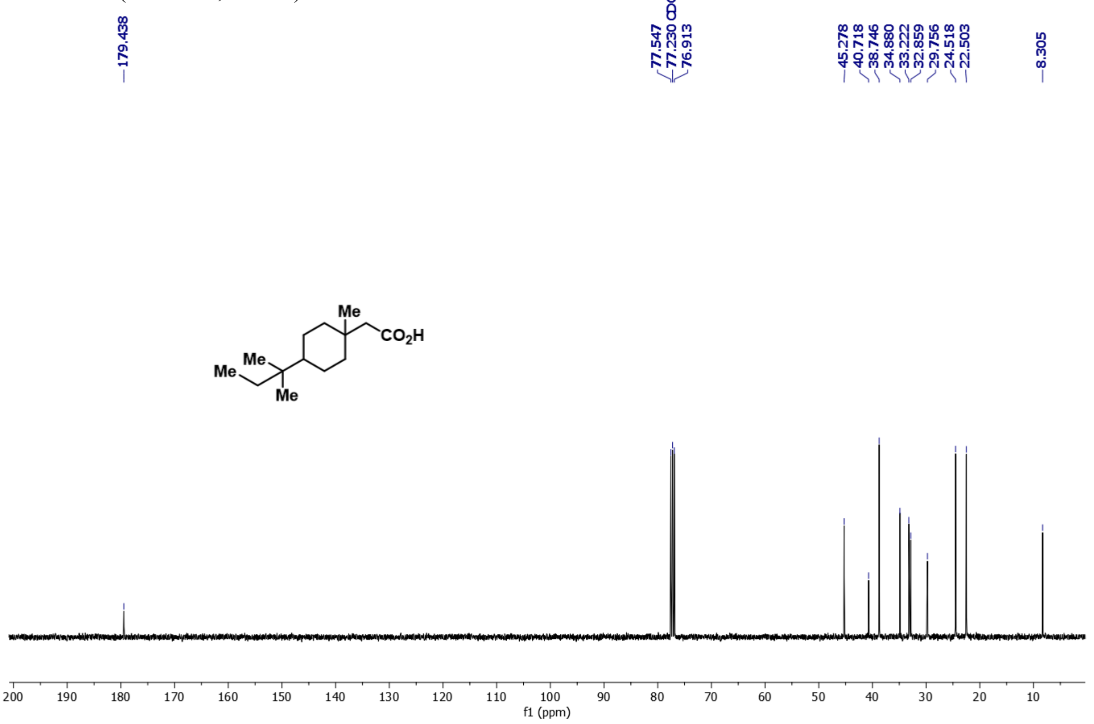

2-(1-Isopropylcyclohexyl)acetic acid (9)

^{1}H NMR (400 MHz, $CDCl_3$)

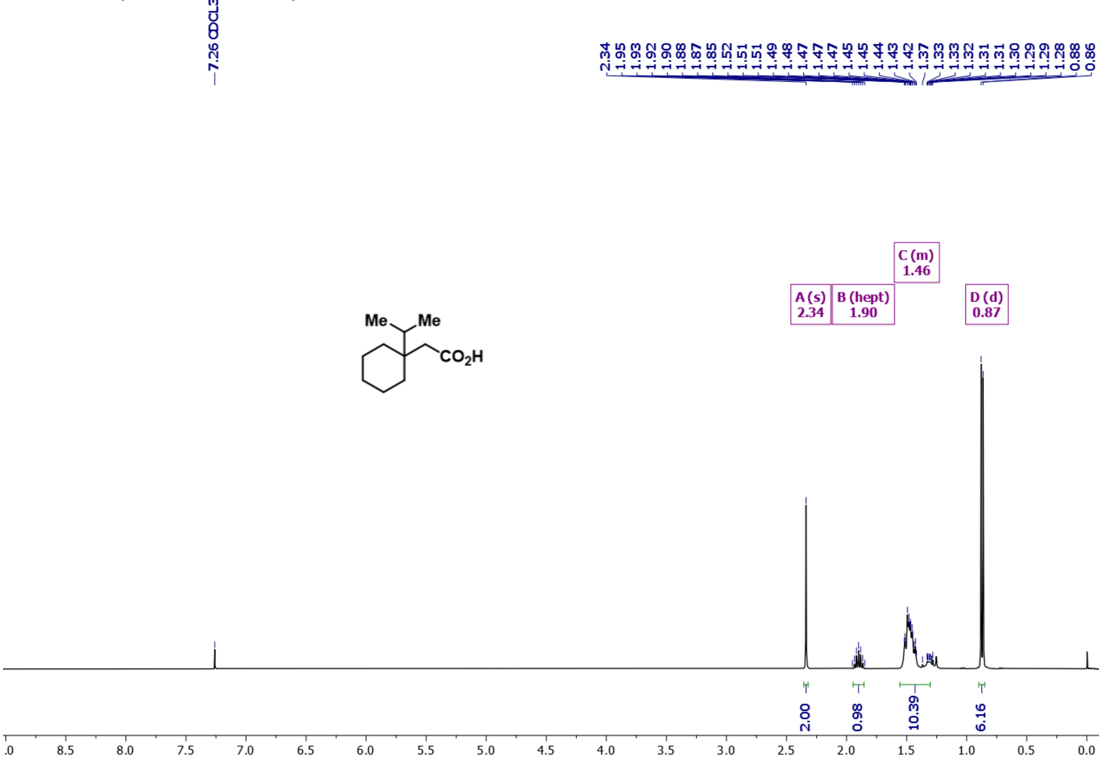

^{13}C NMR (101 MHz, $CDCl_3$)

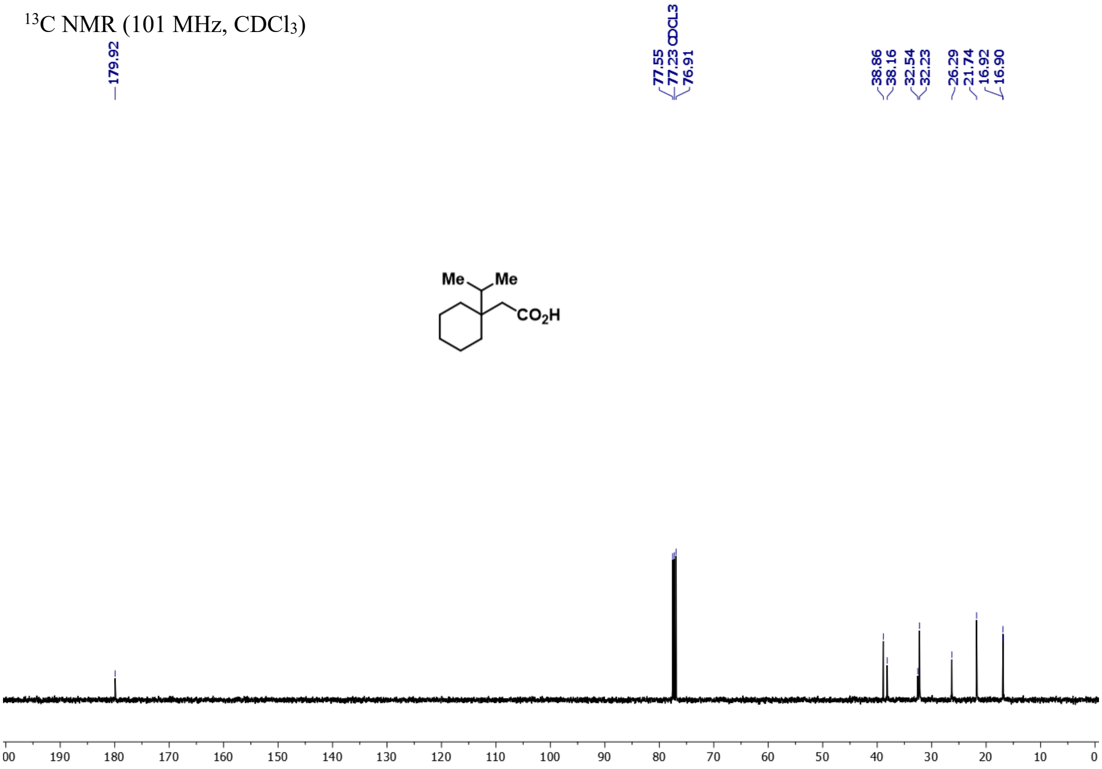

2-(4-(*tert*-Butyl)-1-methylcyclohexyl)acetic acid (10)

^{1}H NMR (400 MHz, $CDCl_3$)

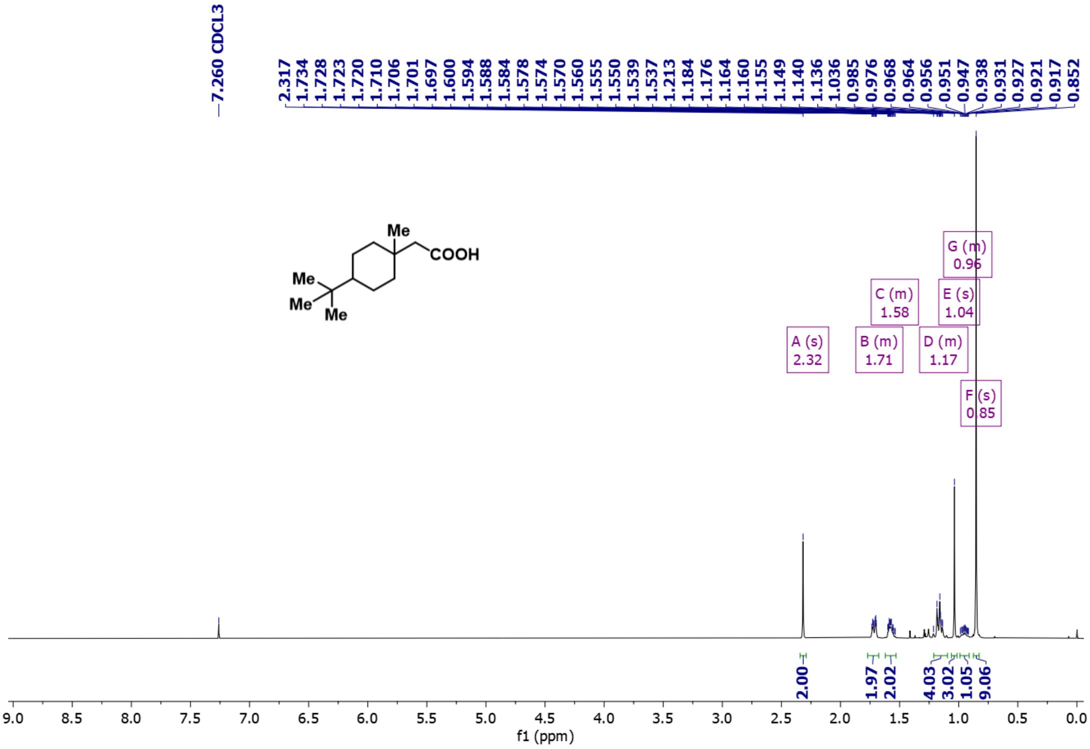

^{13}C NMR (101 MHz, $CDCl_3$)

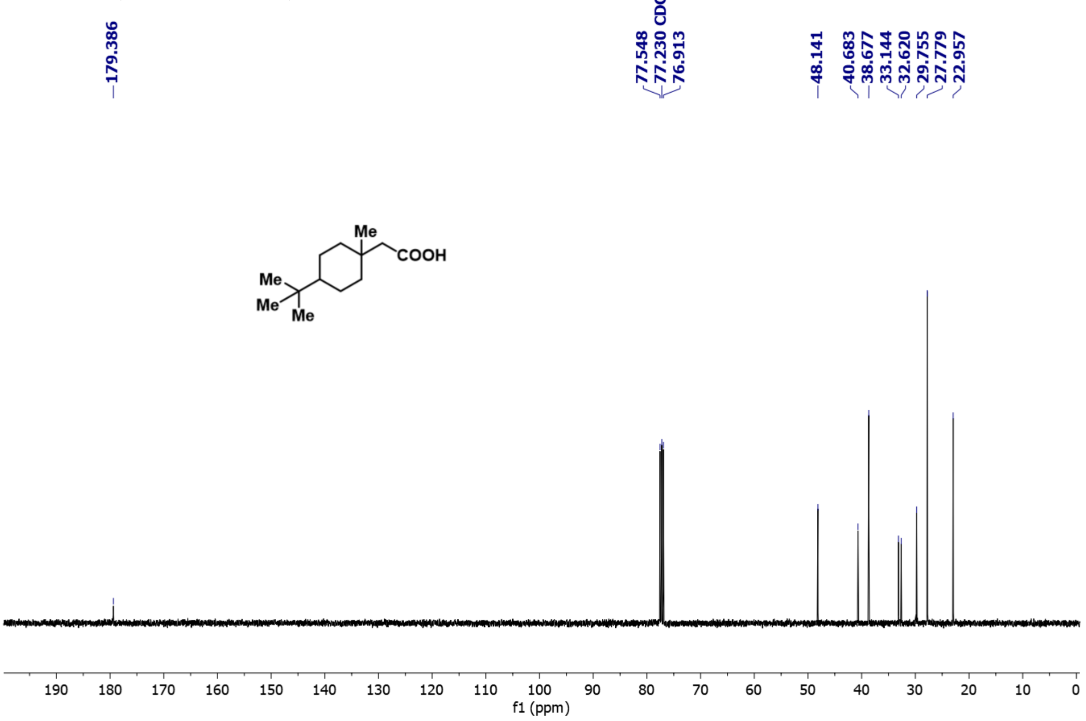

2-(1-Propylcyclohexyl)acetic acid (11)

^{1}H NMR (500 MHz, $CDCl_3$)

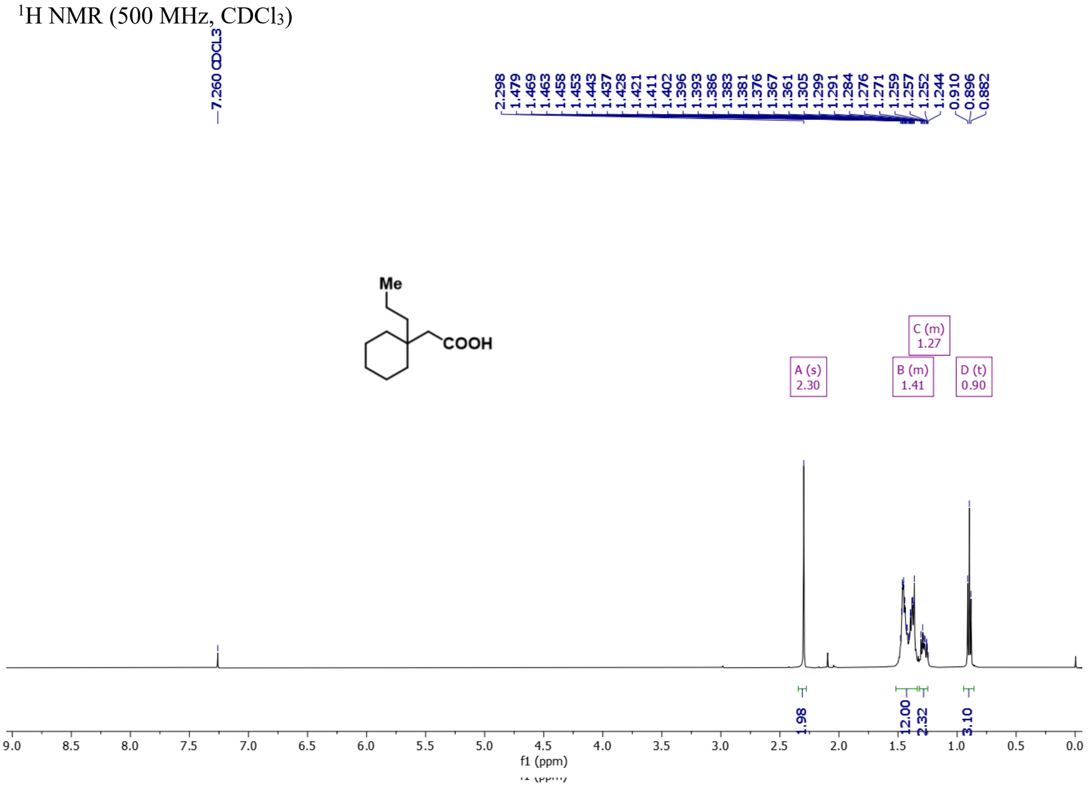

^{13}C NMR (126 MHz, $CDCl_3$)

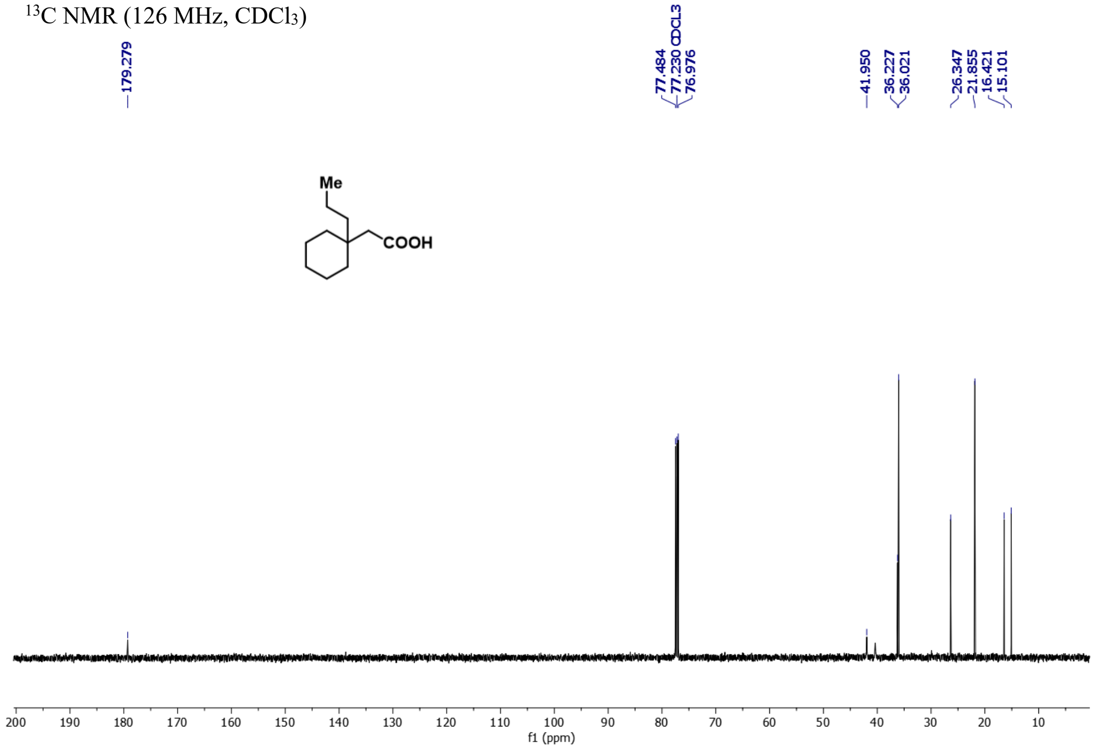

2-(1,4-Dimethylcyclohexyl)acetic acid (12)

^{1}H NMR (400 MHz, CDCl$_3$)

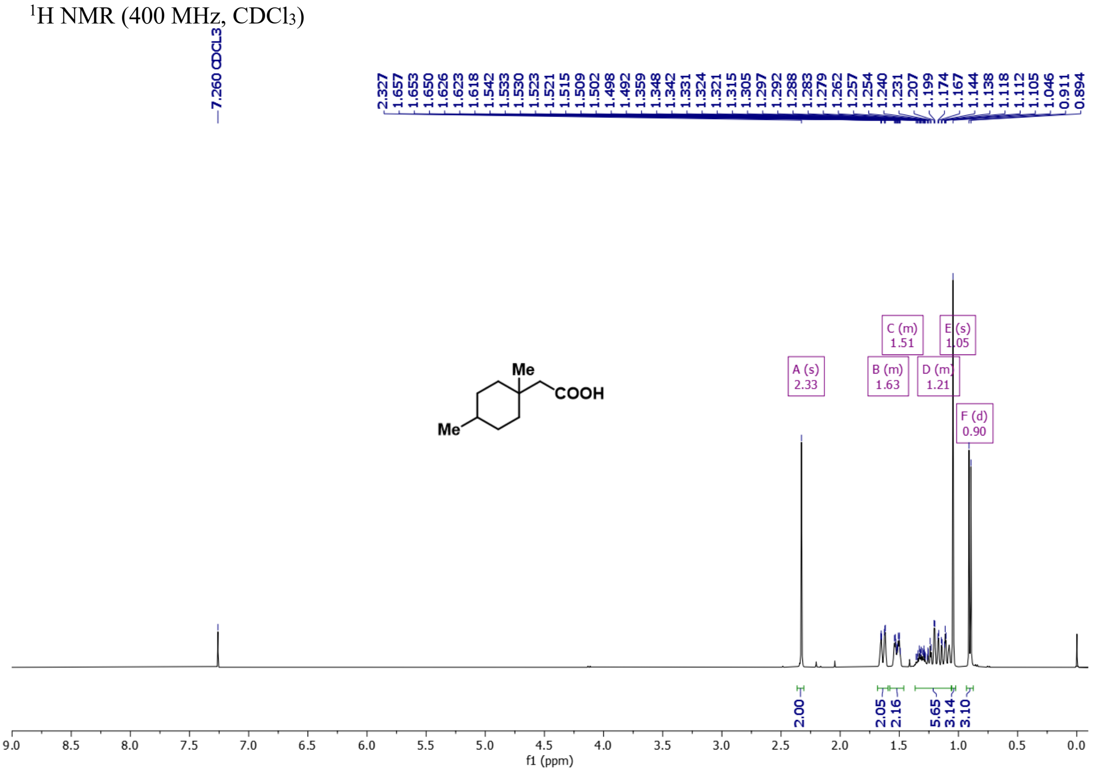

^{13}C NMR (101 MHz, CDCl$_3$)

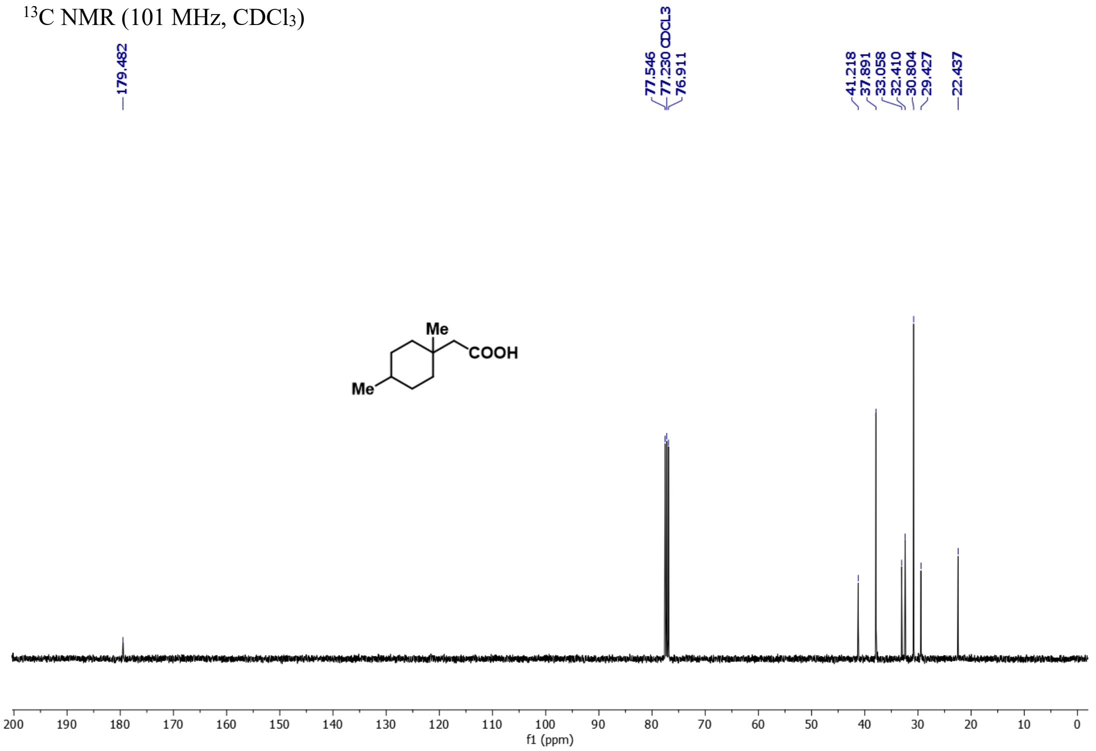

2-(1-Ethylcycloheptyl)acetic acid (13)

^{1}H NMR (400 MHz, $CDCl_3$)

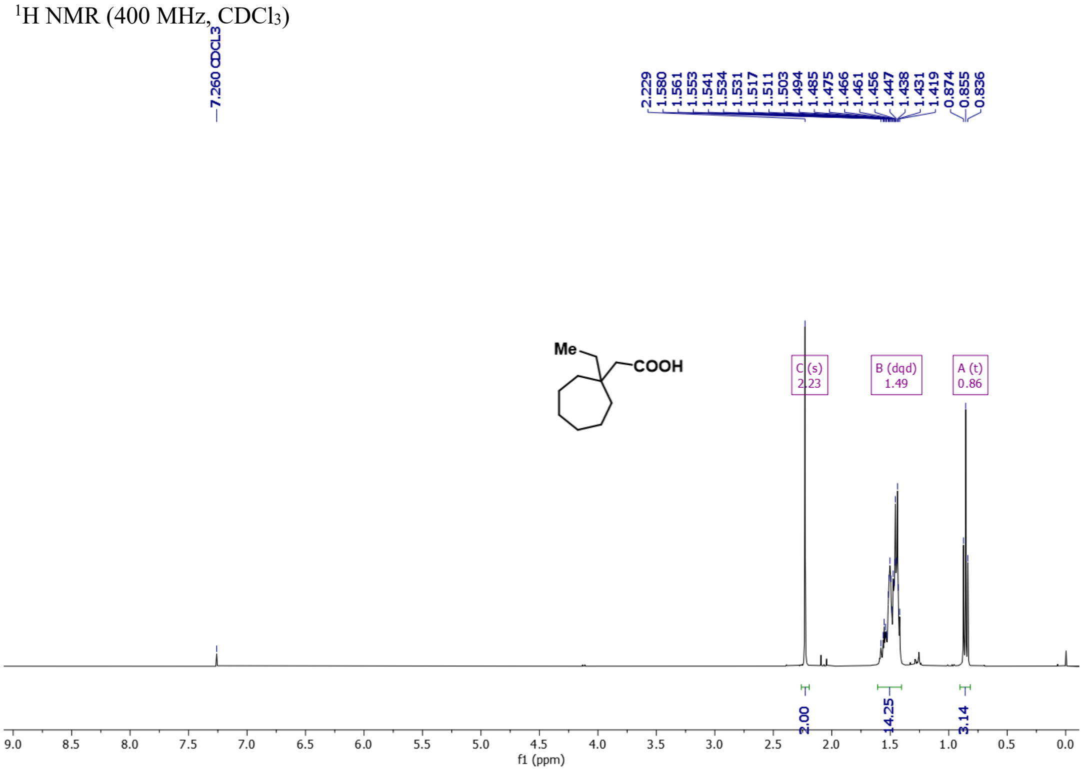

^{13}C NMR (101 MHz, $CDCl_3$)

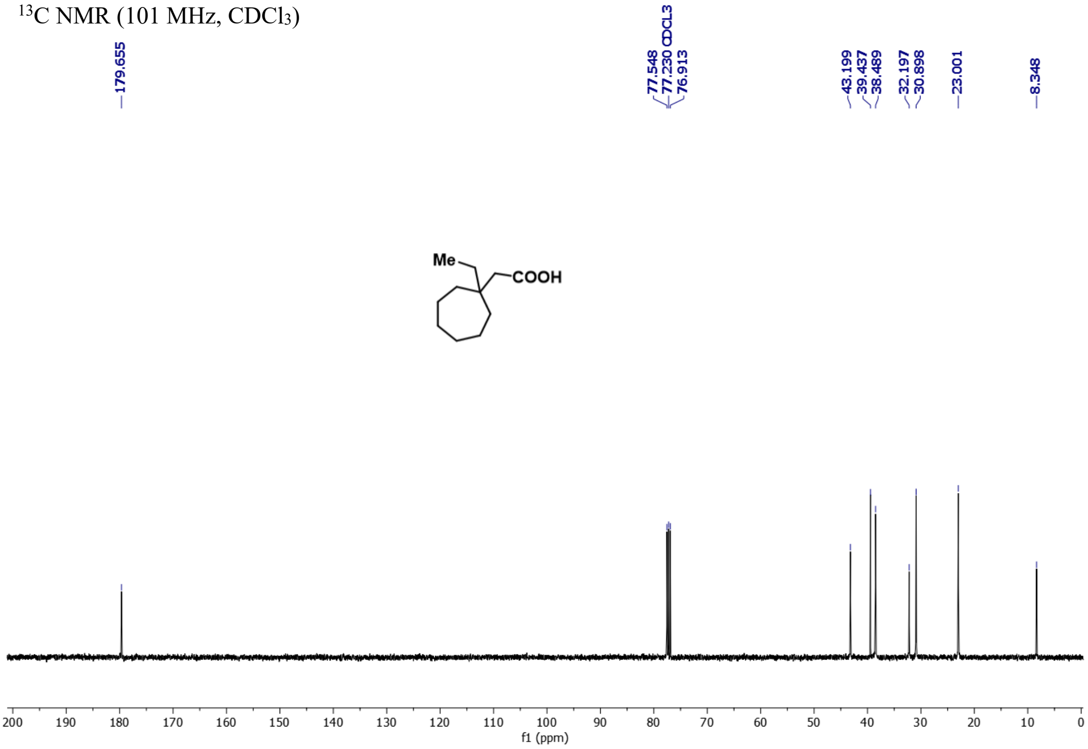

2-(1-Propylcyclopentyl)acetic acid (14)

^{1}H NMR (400 MHz, $CDCl_3$)

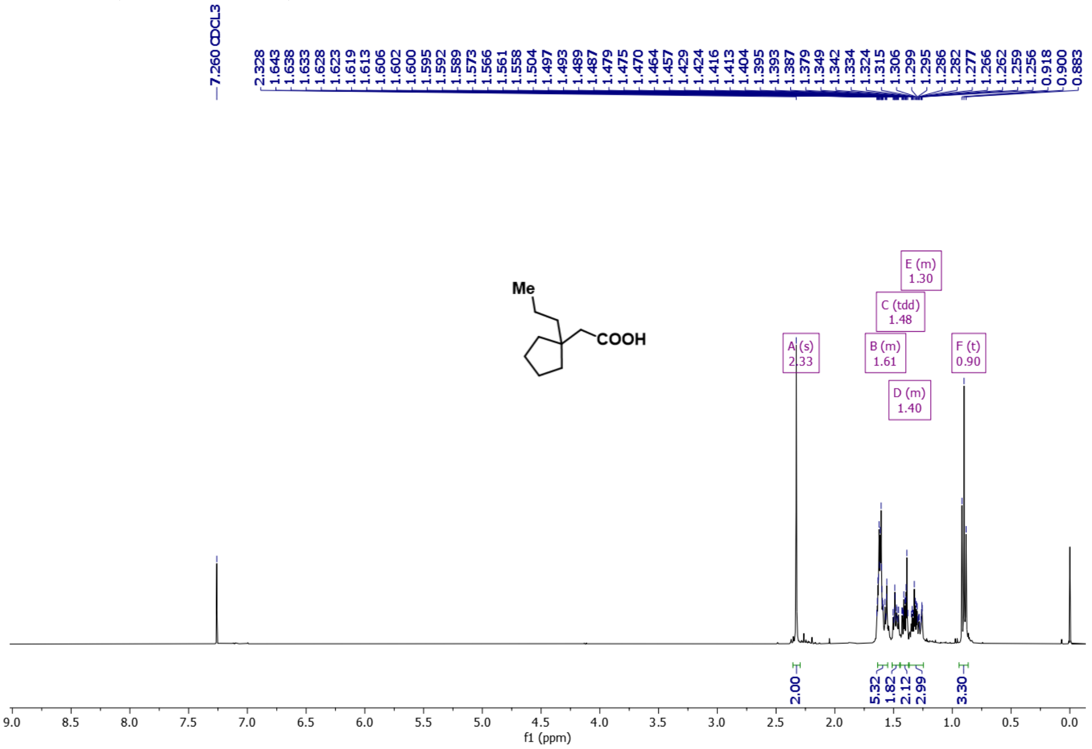

^{13}C NMR (101 MHz, $CDCl_3$)

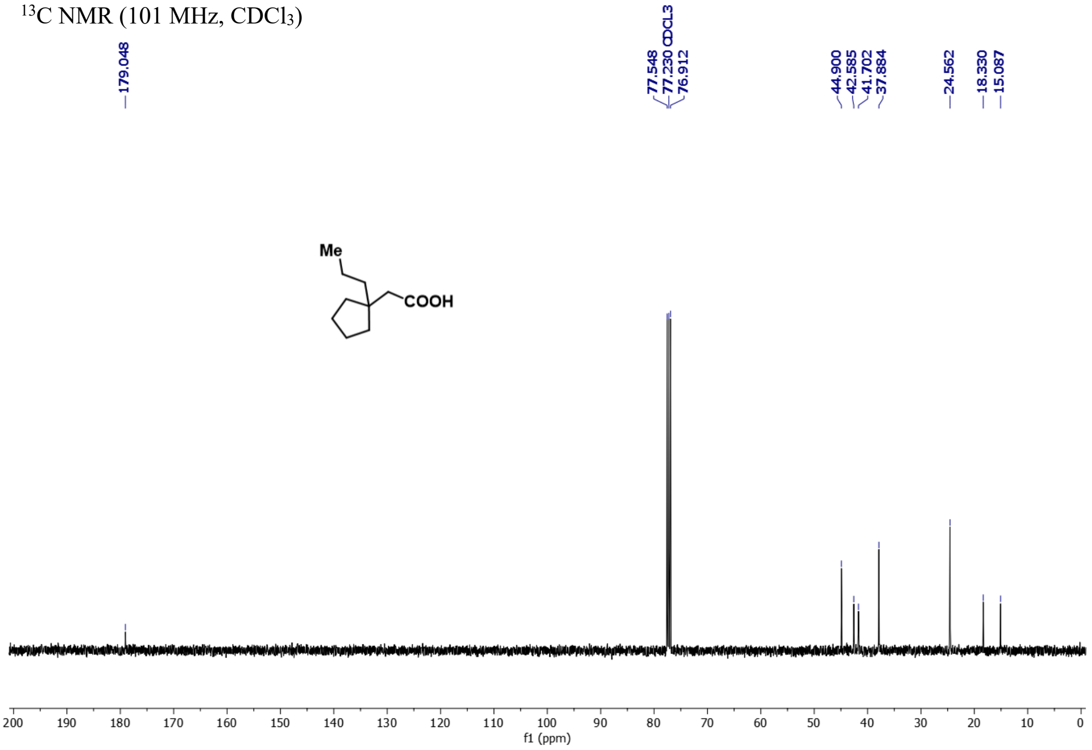

2-(1-Methylcyclododecyl)acetic acid (15)

^{1}H NMR (400 MHz, $CDCl_3$)

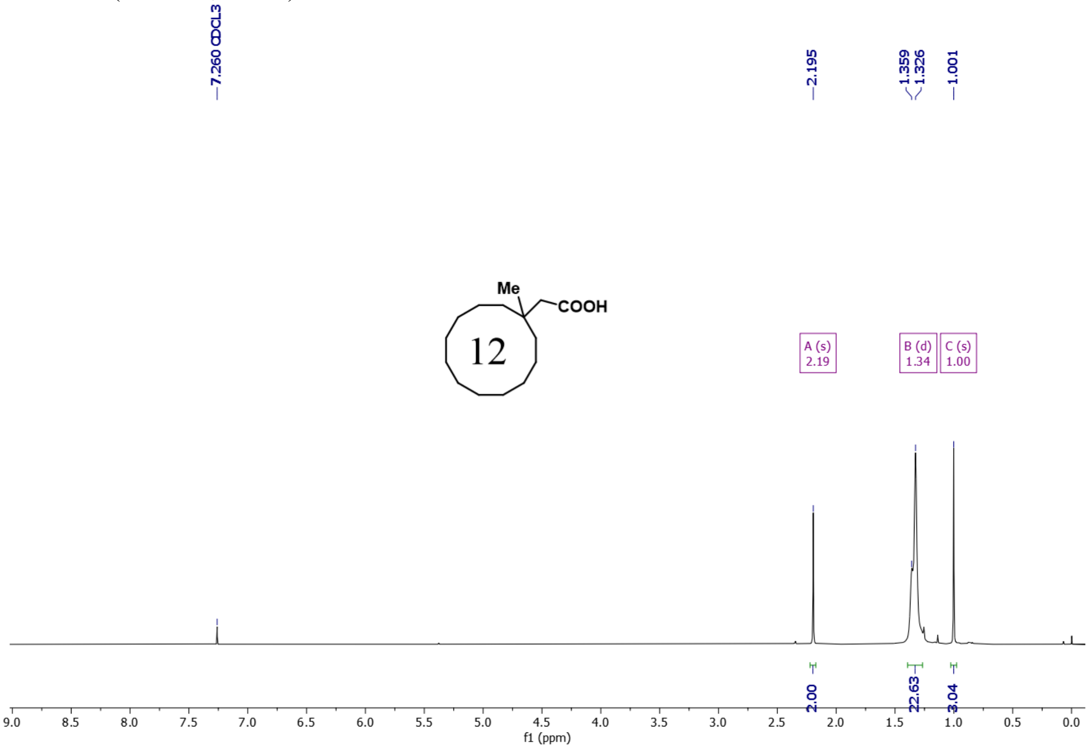

^{13}C NMR (101 MHz, $CDCl_3$)

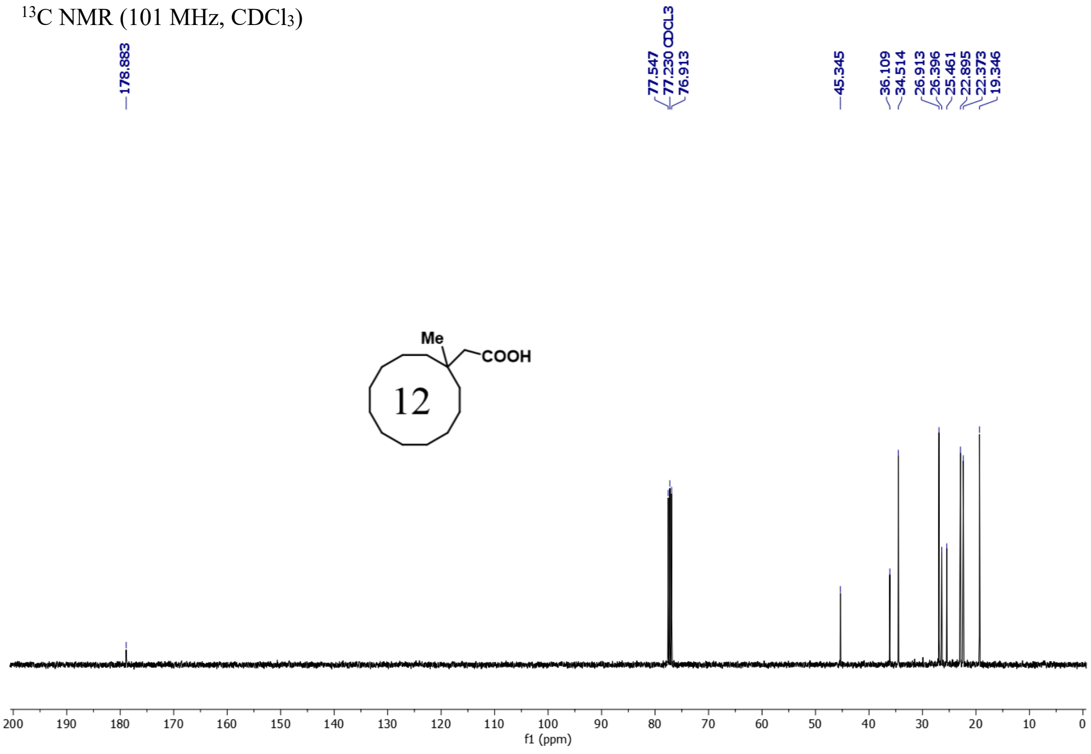

2-(1-Ethyl-4-(*tert*-pentyl)cyclohexyl)acetic acid (16)

^{1}H NMR (400 MHz, $CDCl_3$)

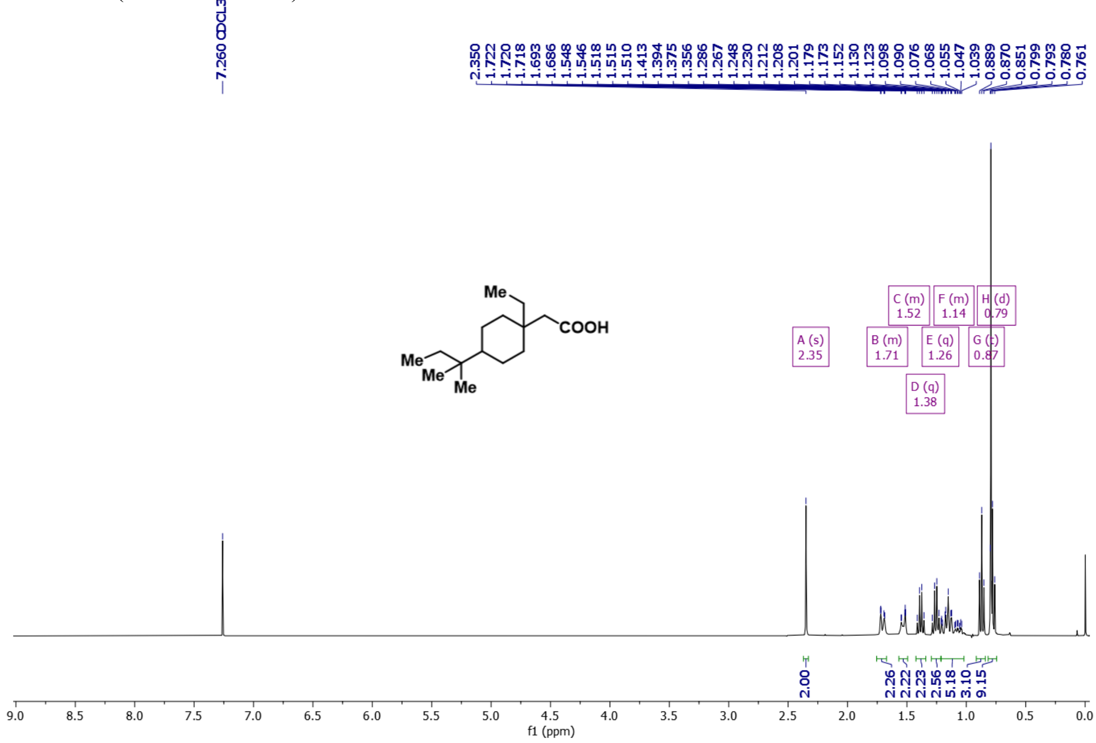

^{13}C NMR (101 MHz, $CDCl_3$)

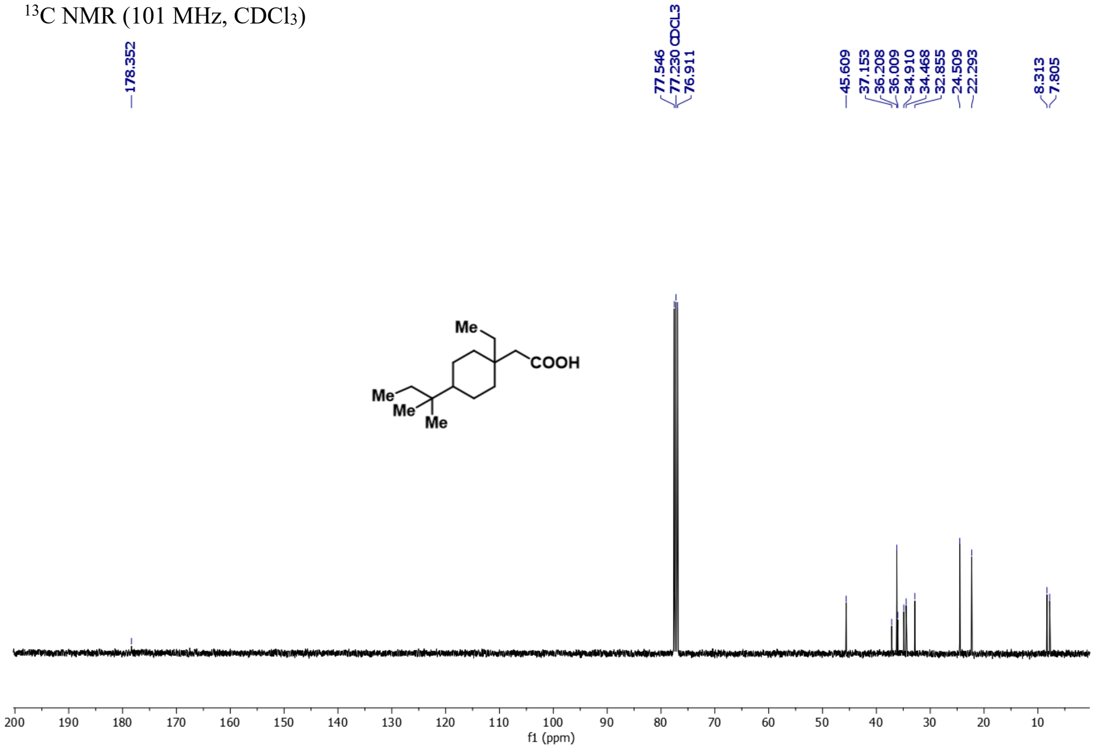

2-(1-Propylcyclohexyl)propanoic acid (17)

^{1}H NMR (400 MHz, $CDCl_3$)

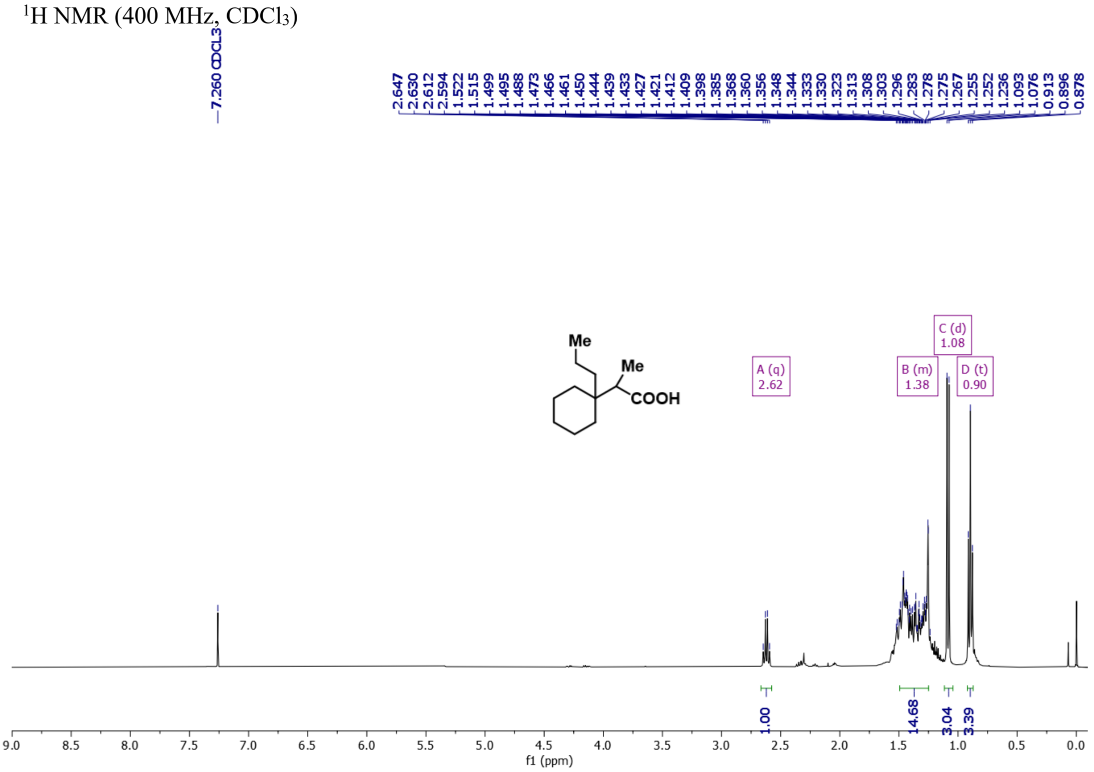

^{13}C NMR (101 MHz, $CDCl_3$)

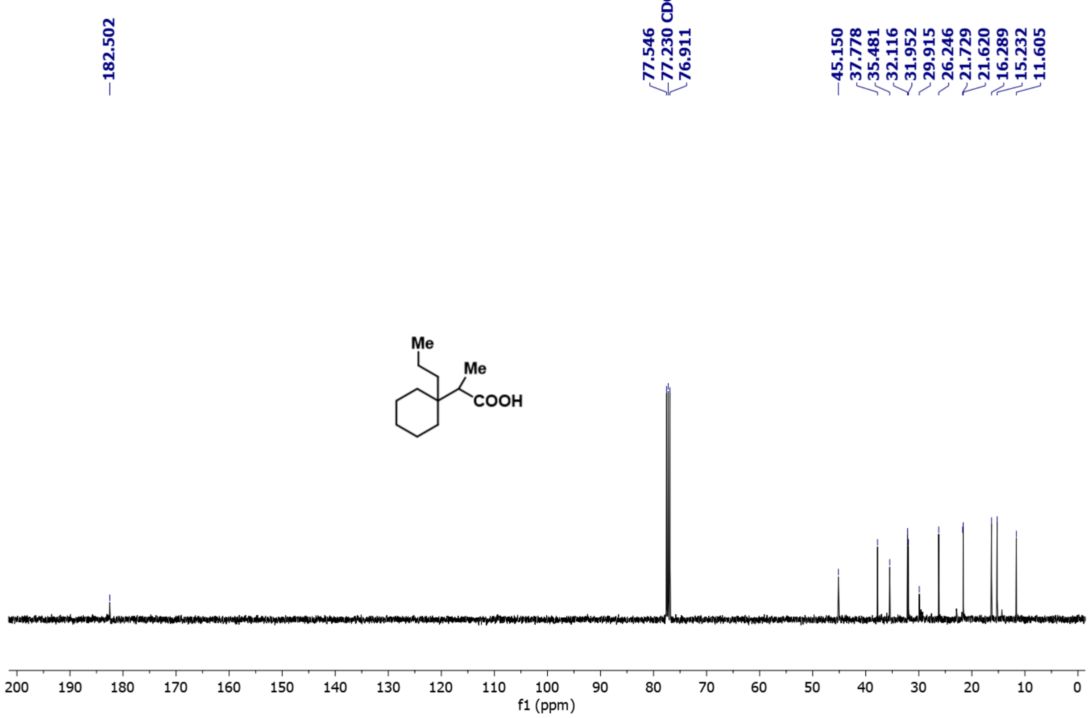

2-(1-Butylcyclohexyl)acetic acid (18)

^{1}H NMR (400 MHz, $CDCl_3$)

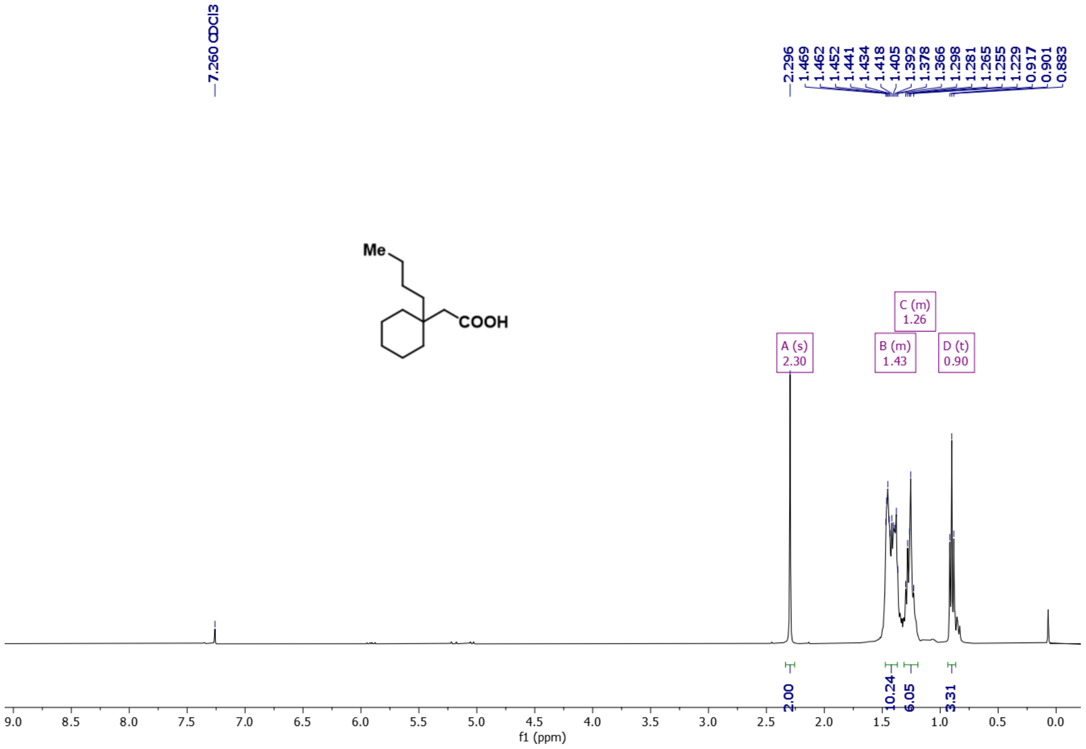

^{13}C NMR (101 MHz, $CDCl_3$)

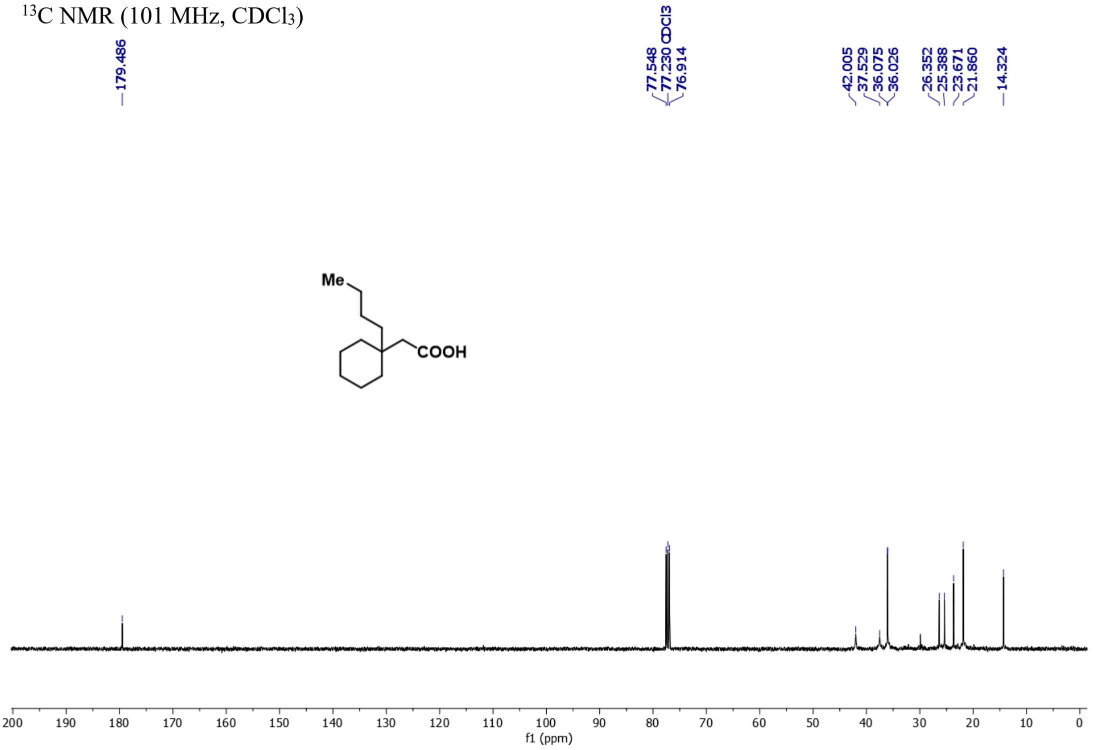

2-(1-Methyl-3-phenylcyclohexyl)acetic acid (19)

^{1}H NMR (500 MHz, $CDCl_3$)

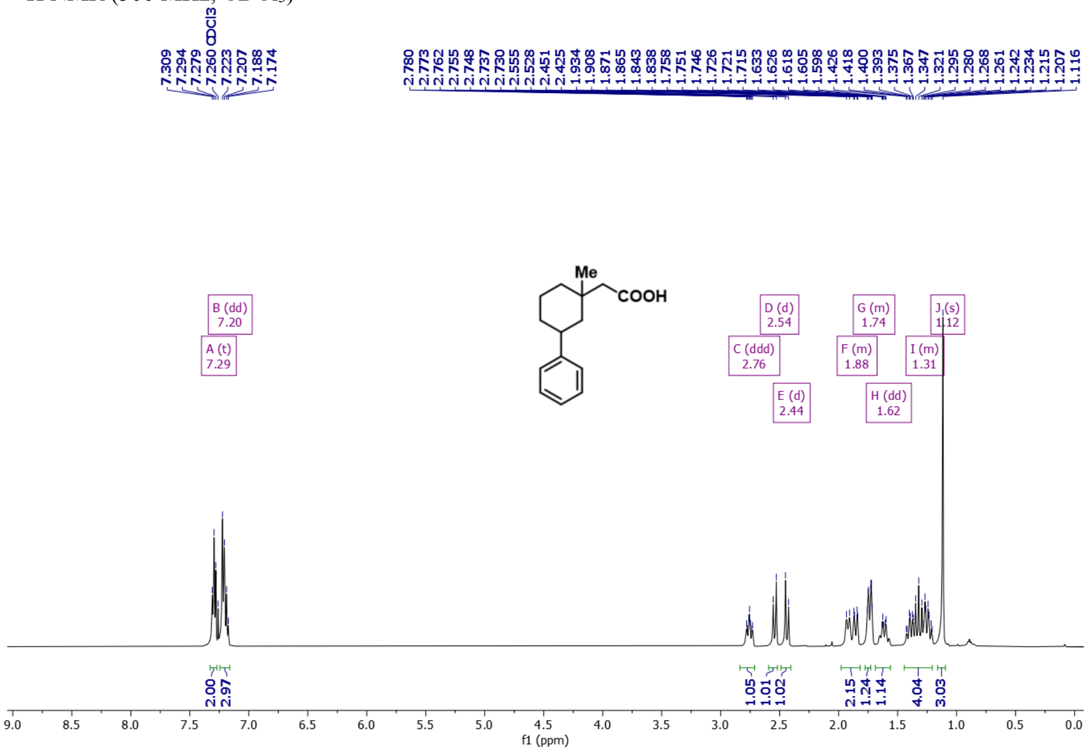

^{13}C NMR (126 MHz, $CDCl_3$)

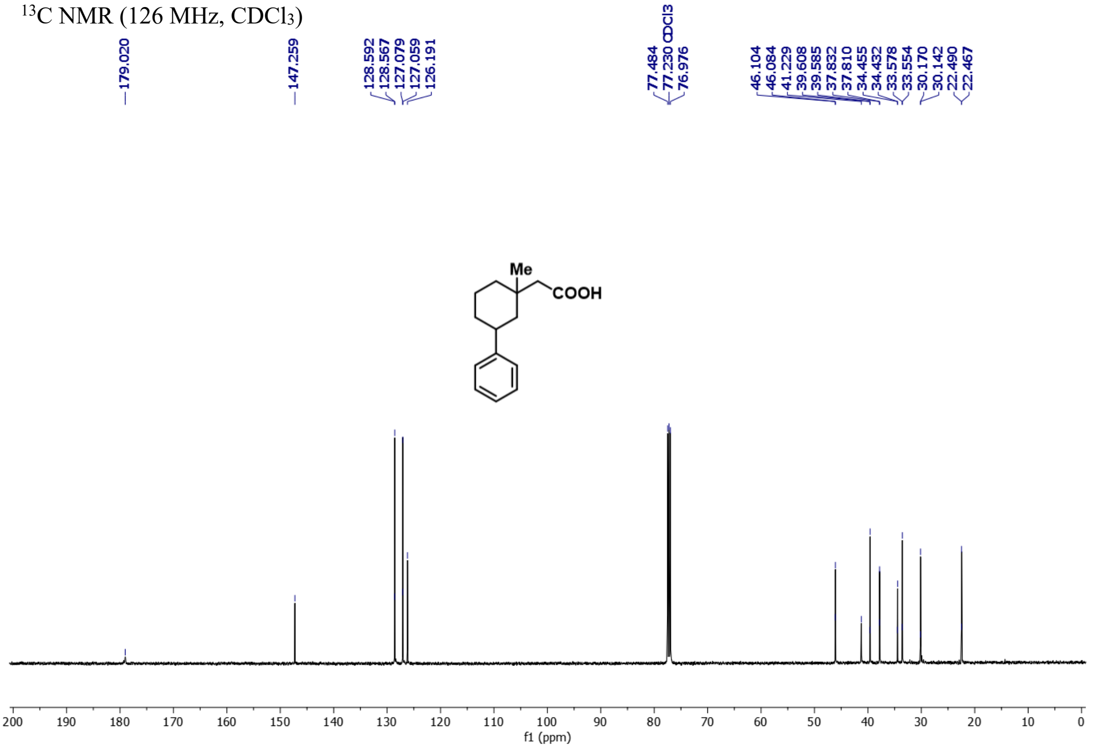

2-(1-Methyl-4-phenylcyclohexyl)acetic acid (20)

^{1}H NMR (400 MHz, $CDCl_3$)

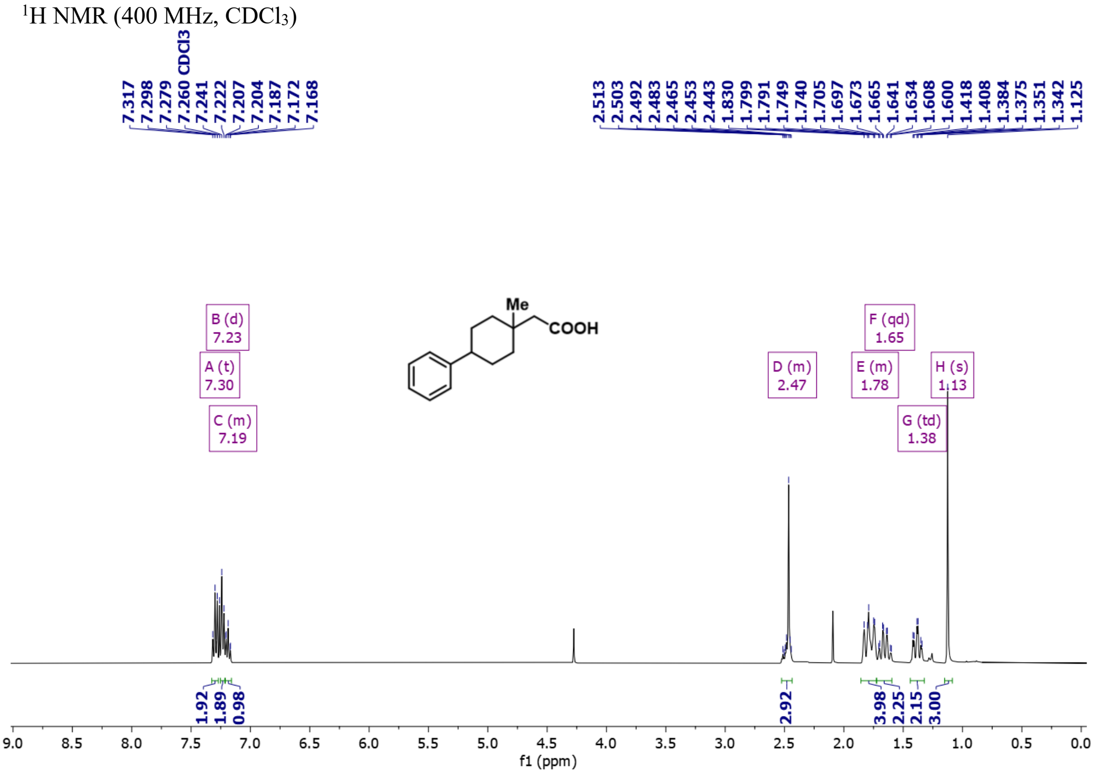

^{13}C NMR (101 MHz, $CDCl_3$)

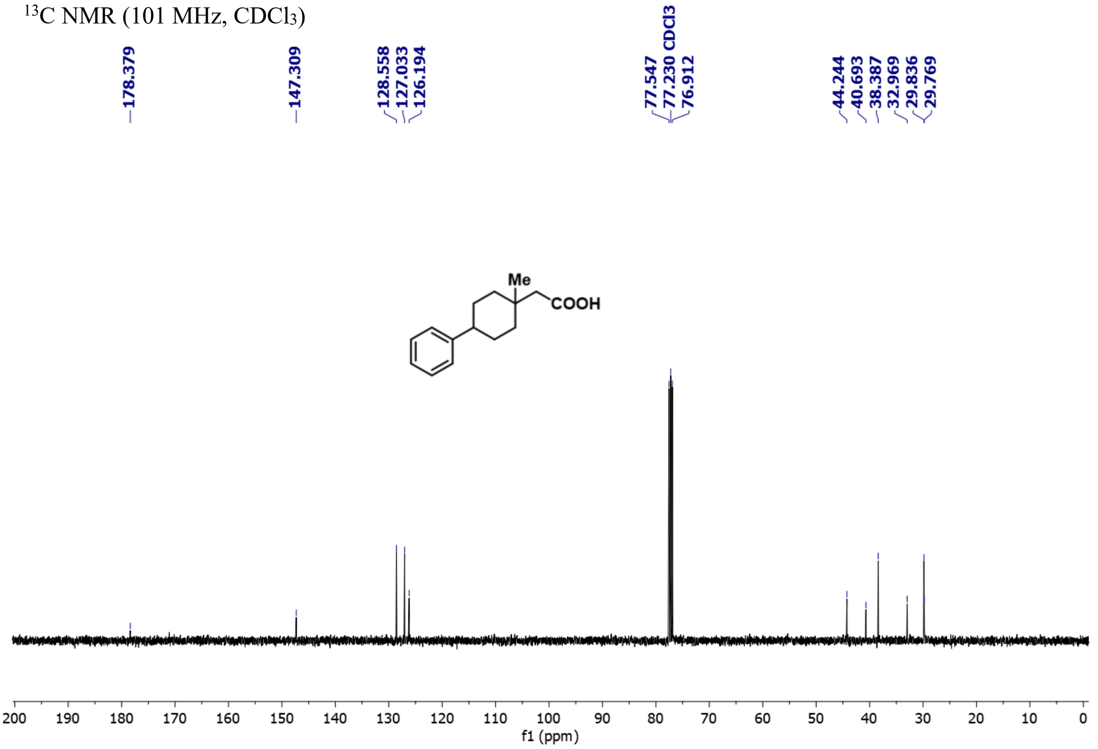

2-(1-(*p*-Tolyl)cyclohexyl)acetic acid (21)

^{1}H NMR (400 MHz, $CDCl_3$)

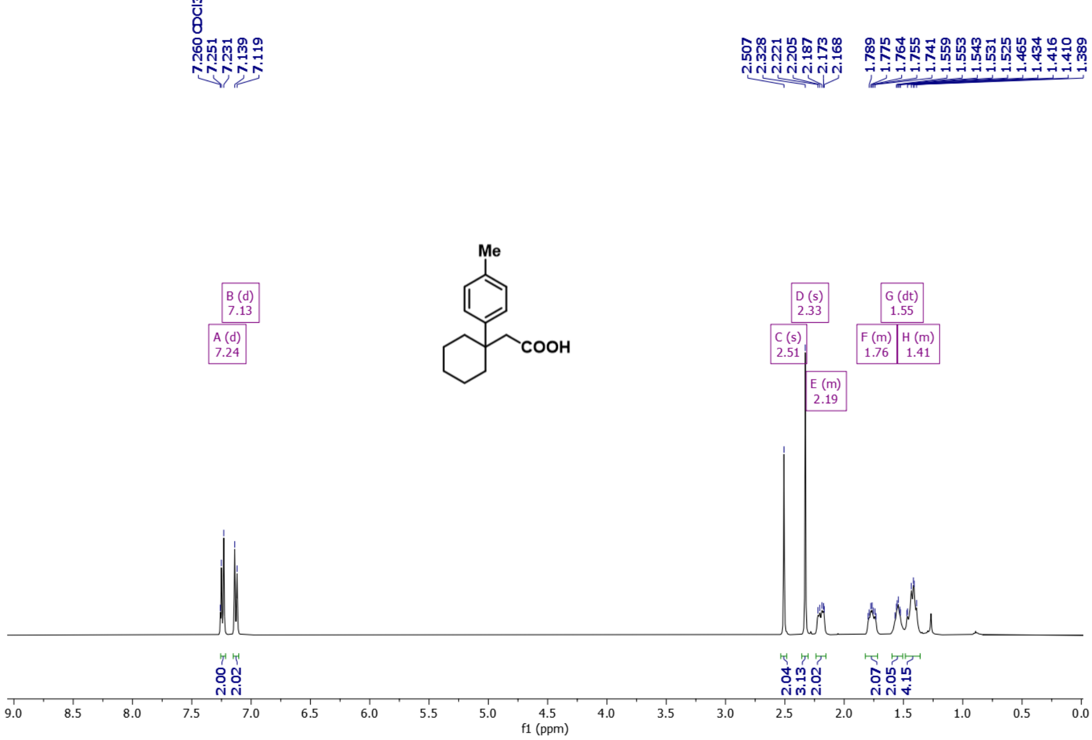

^{13}C NMR (101 MHz, $CDCl_3$)

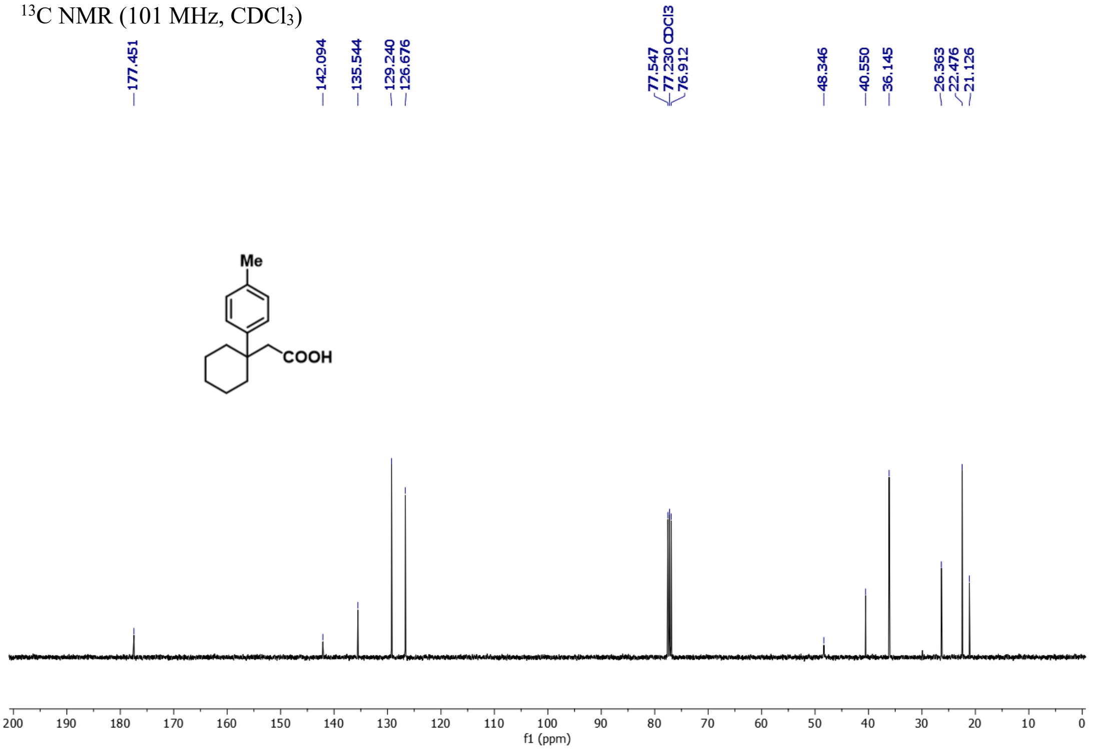

2-(1-(4-Methoxyphenyl)cyclohexyl)acetic acid (22)

^{1}H NMR (500 MHz, $CDCl_3$)

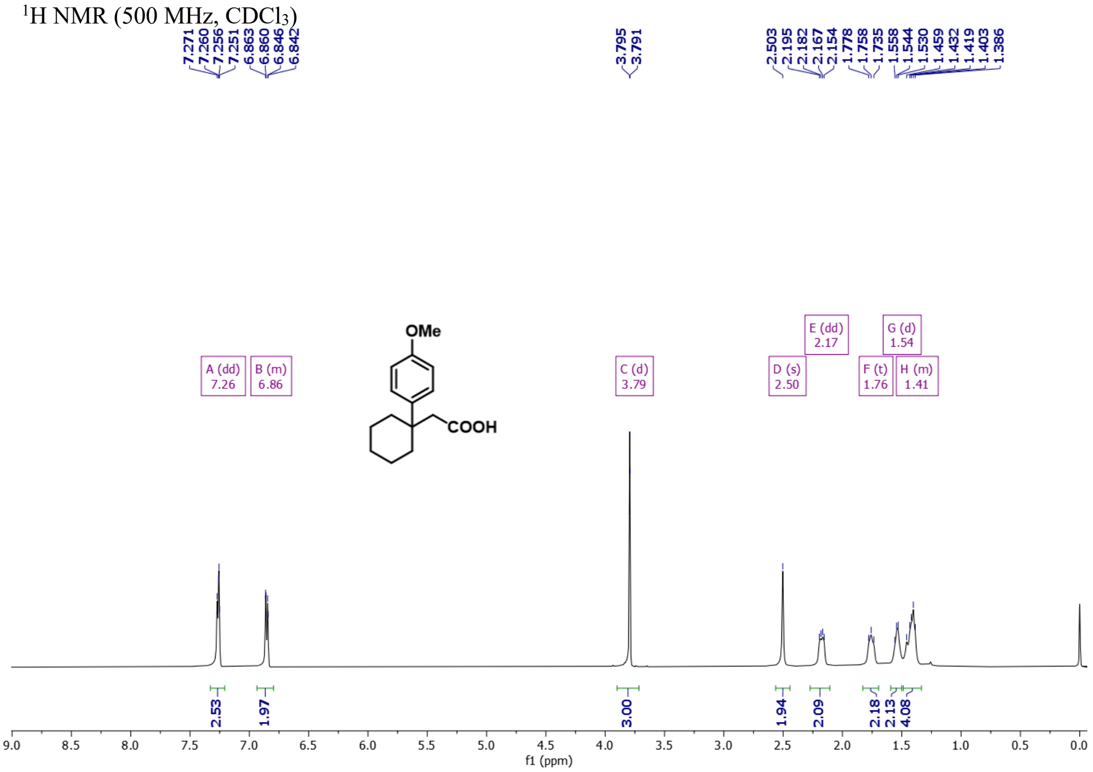

^{13}C NMR (126 MHz, $CDCl_3$)

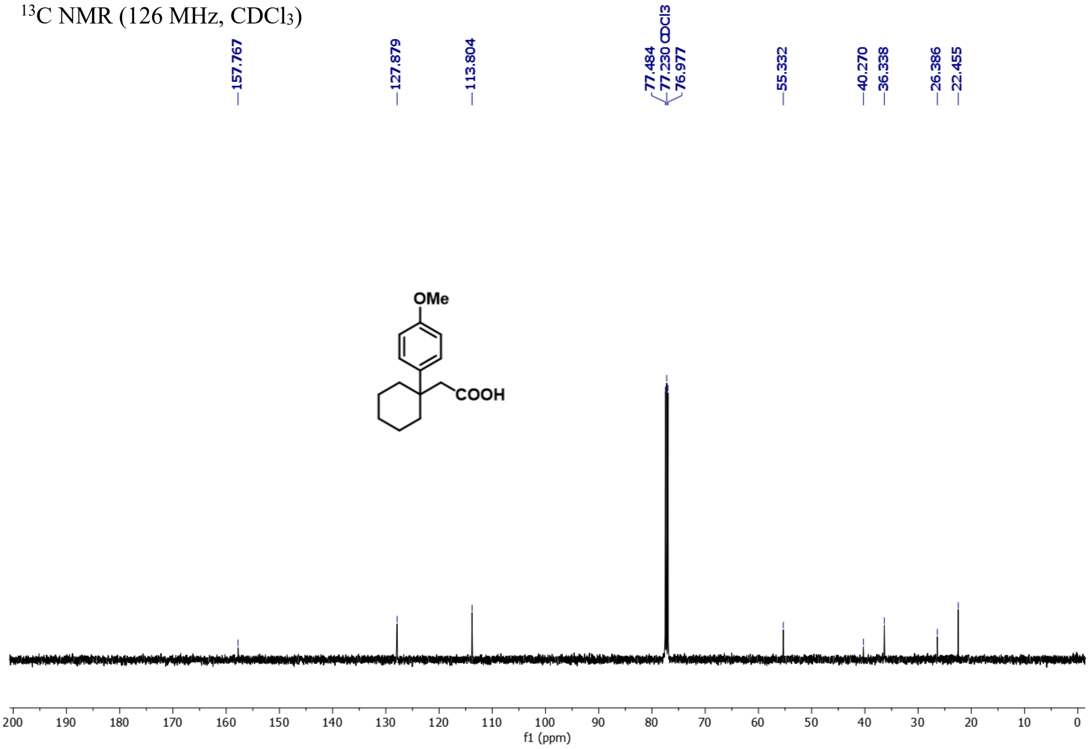

2-(4-Methyl-1-propylcyclohexyl)acetic acid (23)

^{1}H NMR (400 MHz, $CDCl_3$)

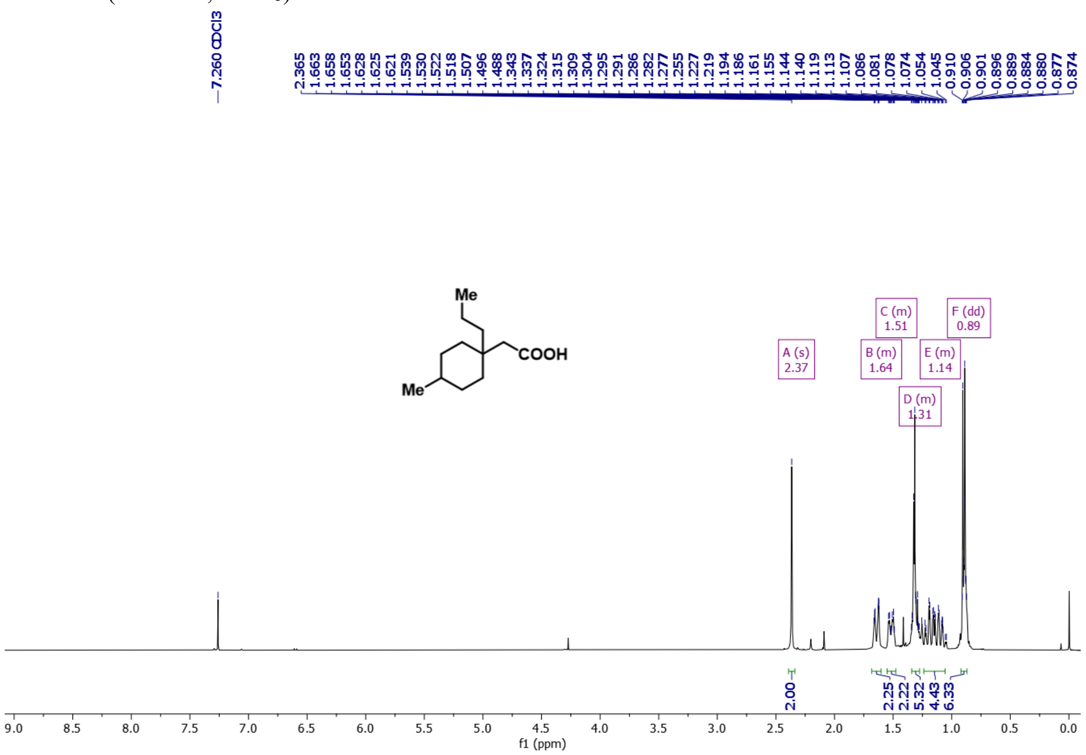

^{13}C NMR (101 MHz, $CDCl_3$)

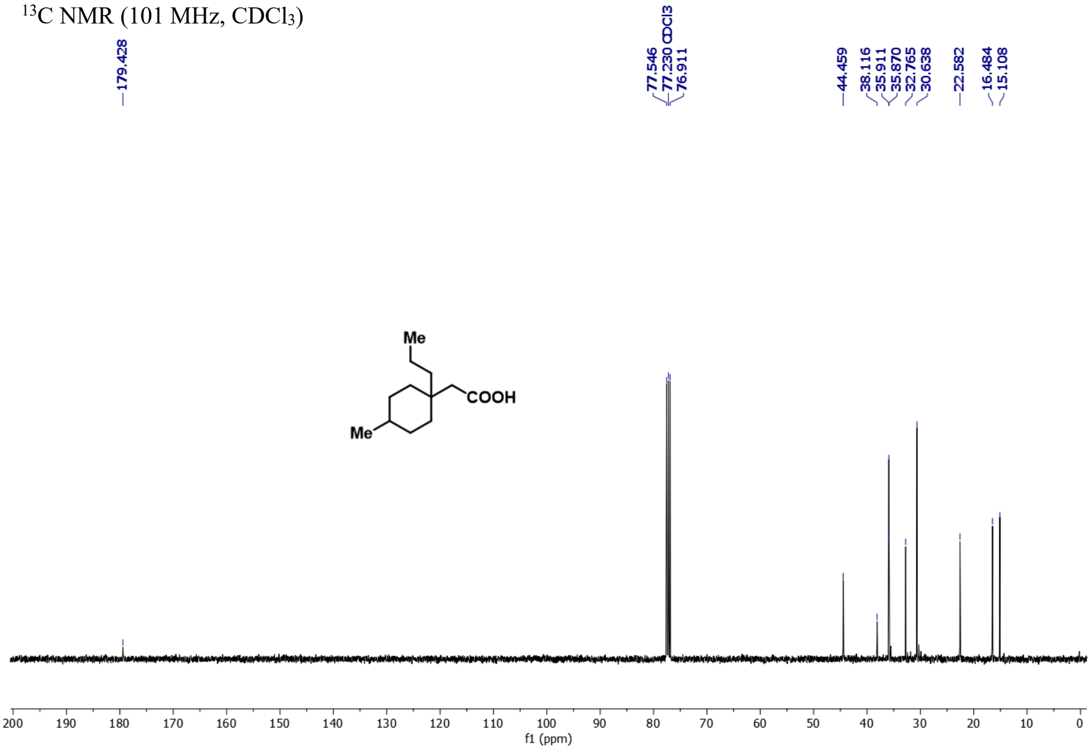

2-(1-Methyl-3-(naphthalen-1-yl)cyclohexyl)acetic acid (24)

^{1}H NMR (400 MHz, $CDCl_3$)

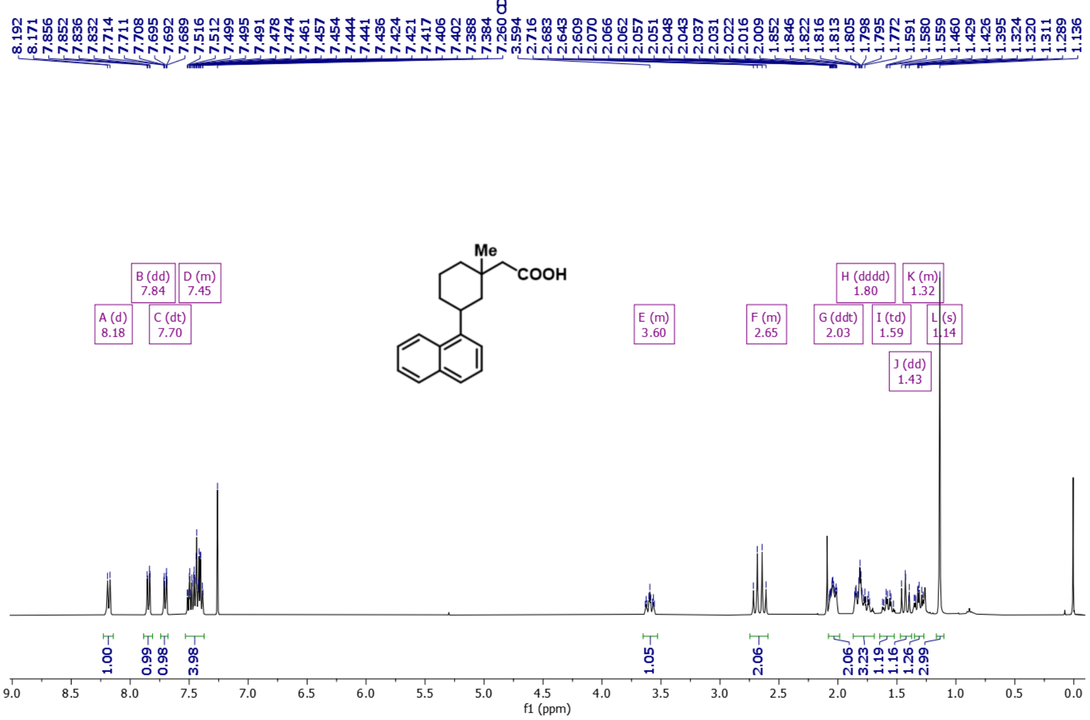

^{13}C NMR (101 MHz, $CDCl_3$)

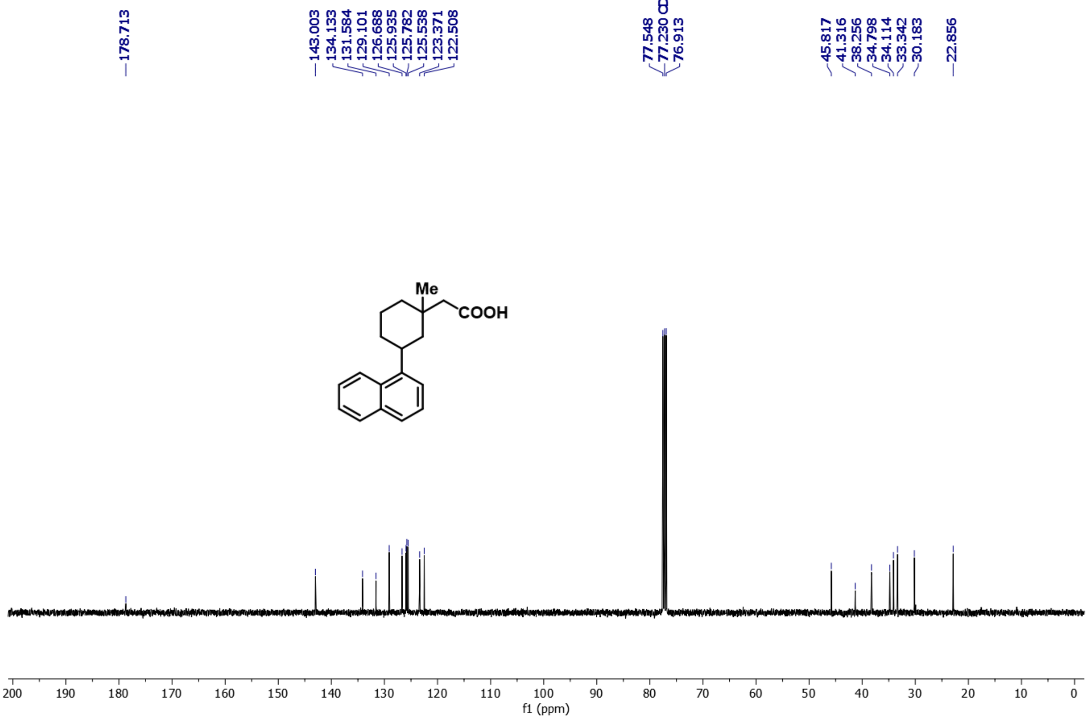

2-(3-(2-Fluorophenyl)-1-methylcyclohexyl)acetic acid (25)

^{1}H NMR (400 MHz, $CDCl_3$)

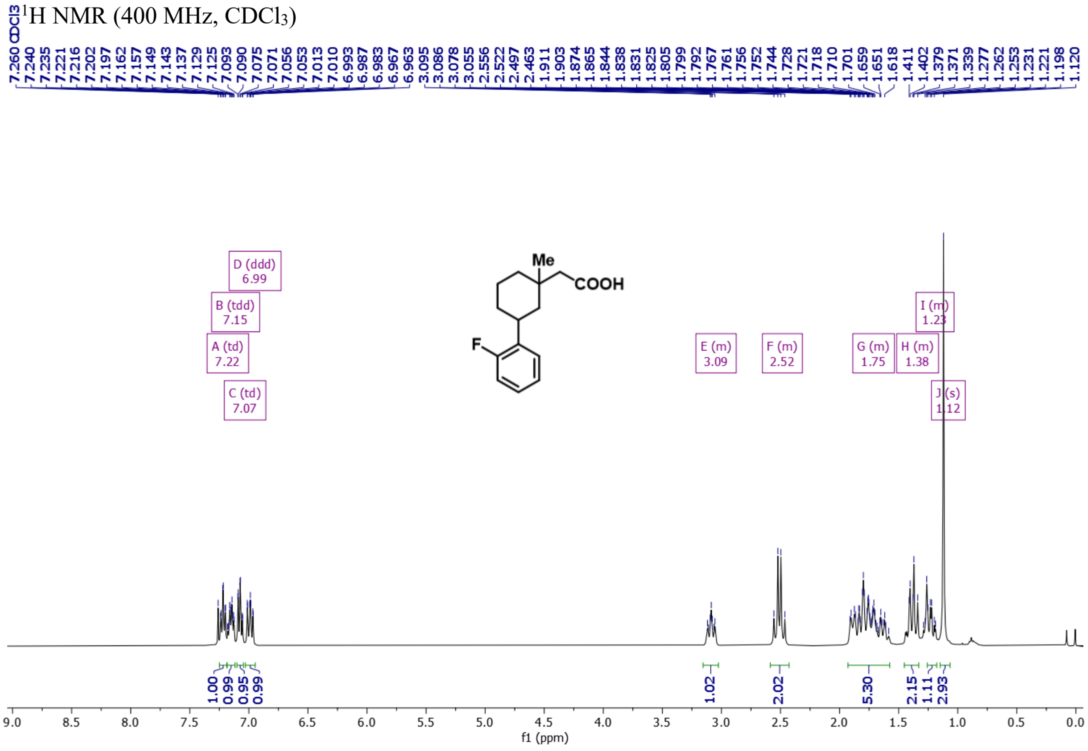

^{13}C NMR (101 MHz, $CDCl_3$)

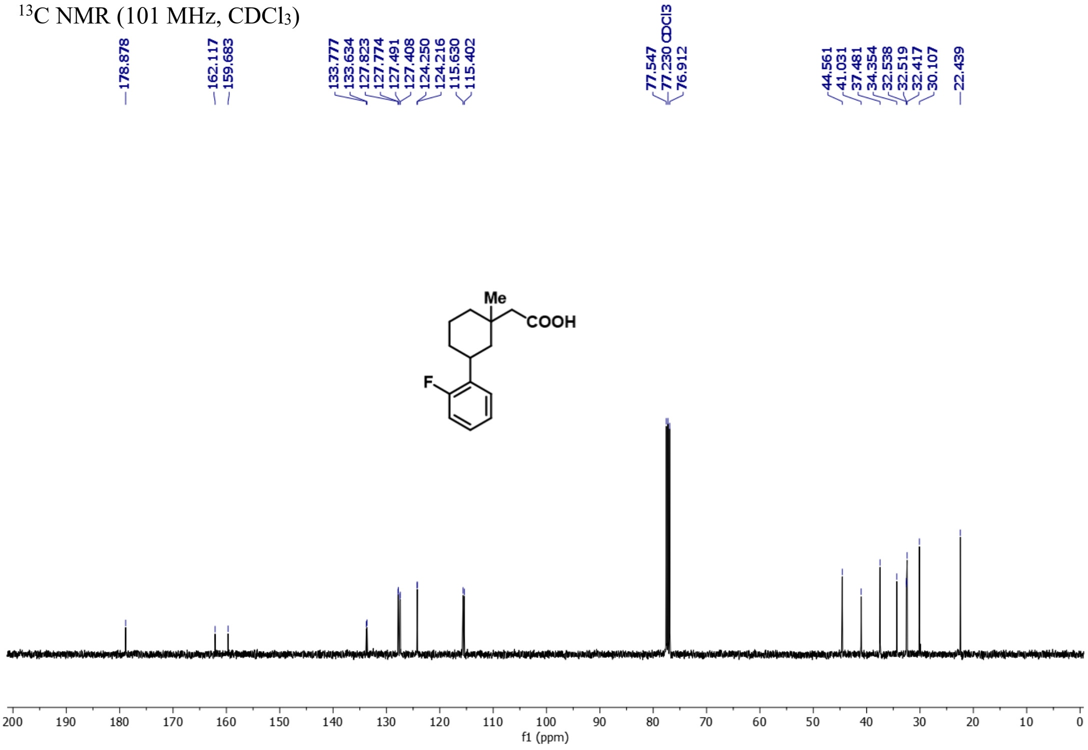

^{19}F NMR (376 MHz, $CDCl_3$)

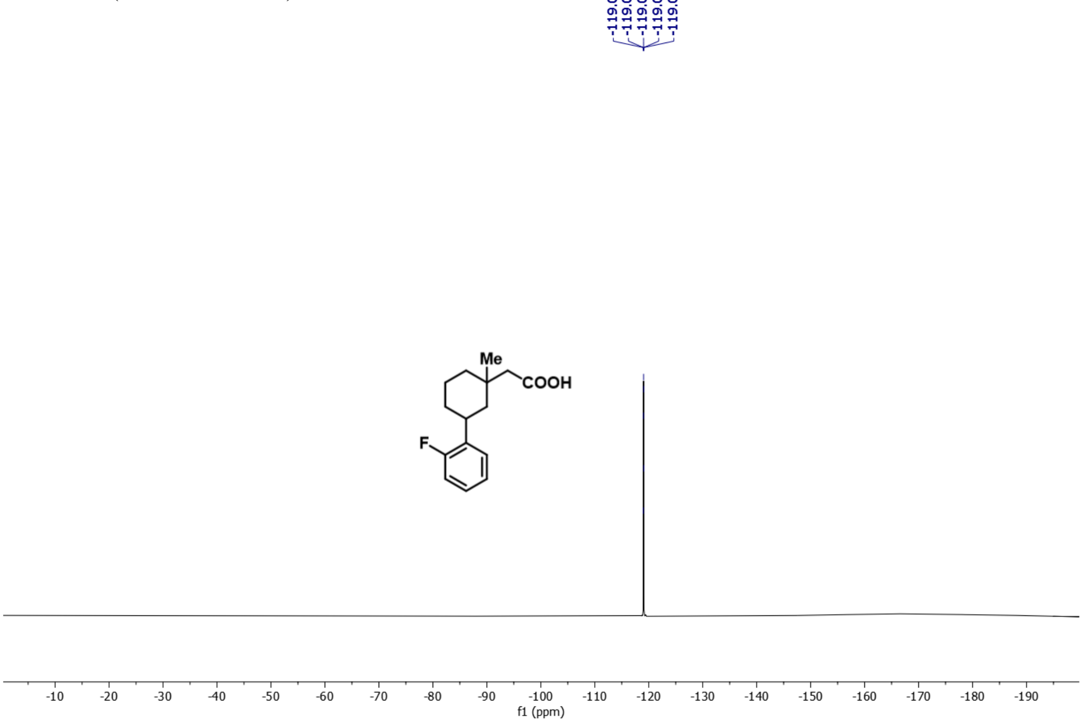

NOESY data to establish the stereochemistry of 3-aryl-1-(methylcyclohexyl)acetic acids. 2-(3-(2-fluorophenyl)-1-methylcyclohexyl)acetic acid has taken here as an representative example.

There could be total four conformations based on syn and anti-relationship between Me and 3-substituted Aryl group as shown below. Conformations where Aryl ring is in axial position would be less stable due to 1,3-diaxial interaction (A and C). Thus B and D are more stable conformations for syn and anti-diastereomers. Our aim here to identify the relative stereochemistry (syn or anti) though NOESY.

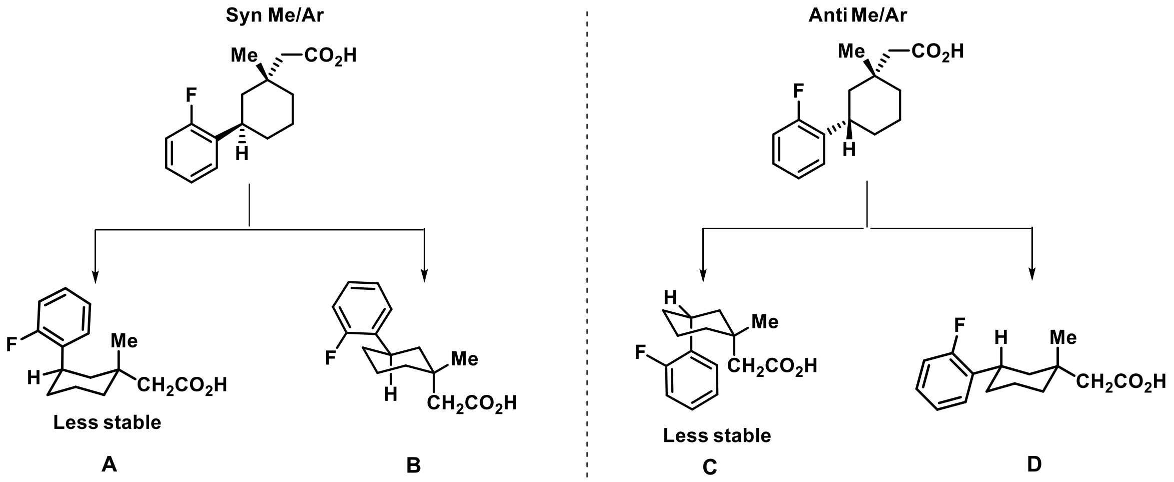

Following is the NOESY data for 2-(3-(2-fluorophenyl)-1-methylcyclohexyl)acetic acid.

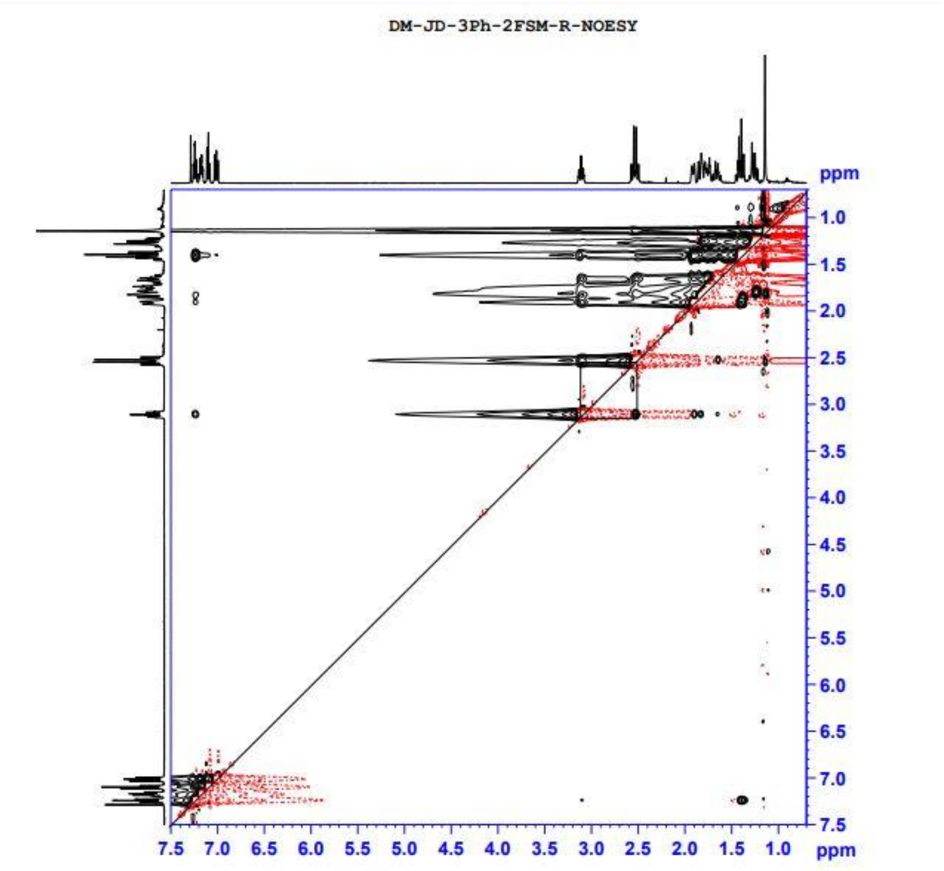

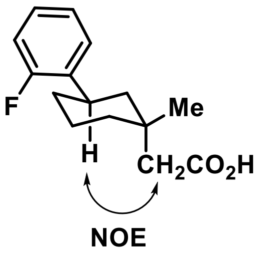

B

Established via NOESY experiment

Thus NOE interaction between H and methylene group as shown in the abovementioned structure indicates the syn Me/Aryl relationship.

2-(1-Isobutylcyclohexyl)acetic acid (26)

^{1}H NMR (400 MHz, $CDCl_3$)

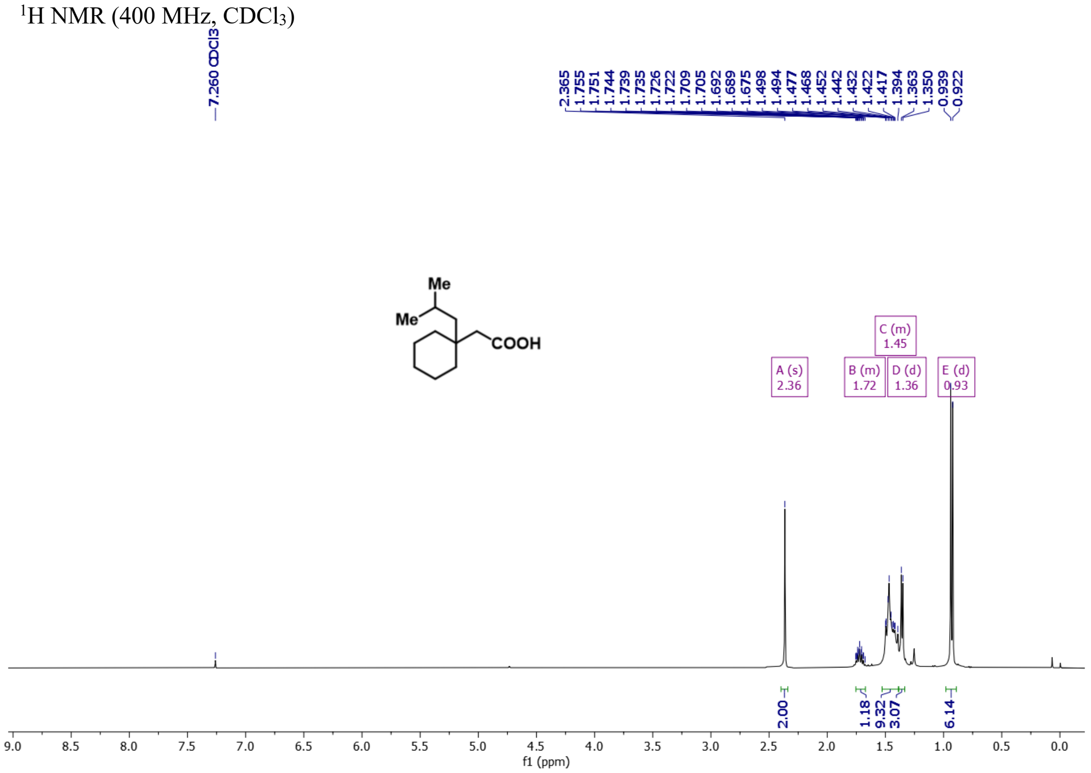

^{13}C NMR (101 MHz, $CDCl_3$)

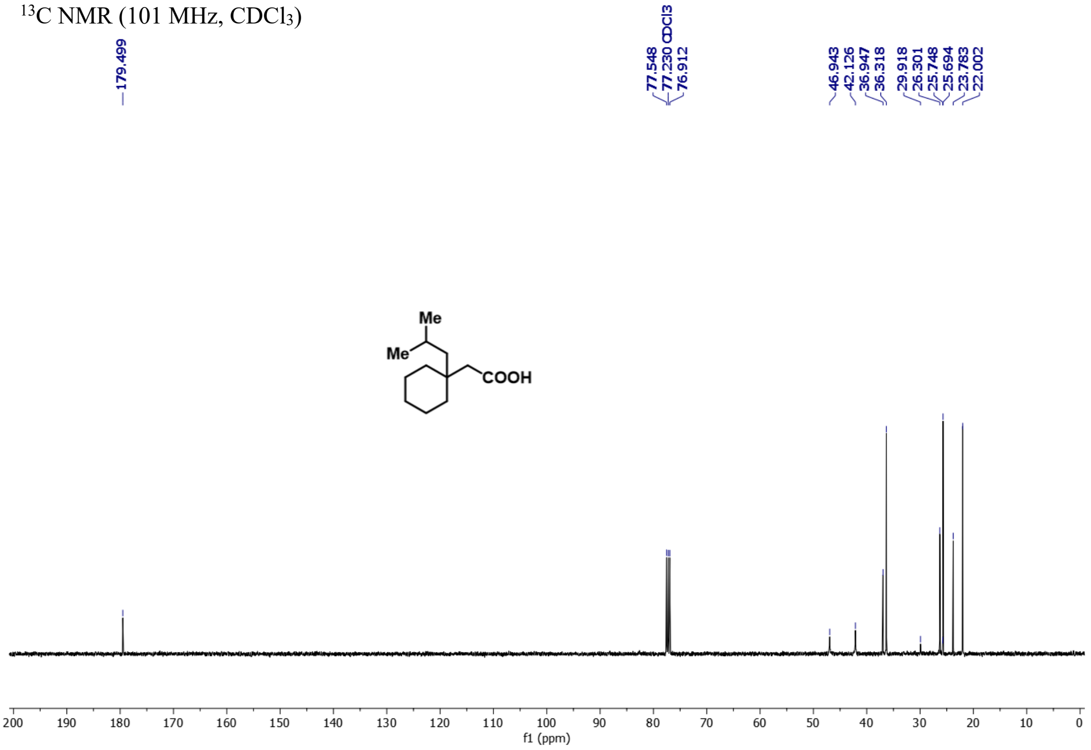

2-(1-Cyclopentylcyclohexyl)acetic acid (27)

^{1}H NMR (400 MHz, $CDCl_3$)

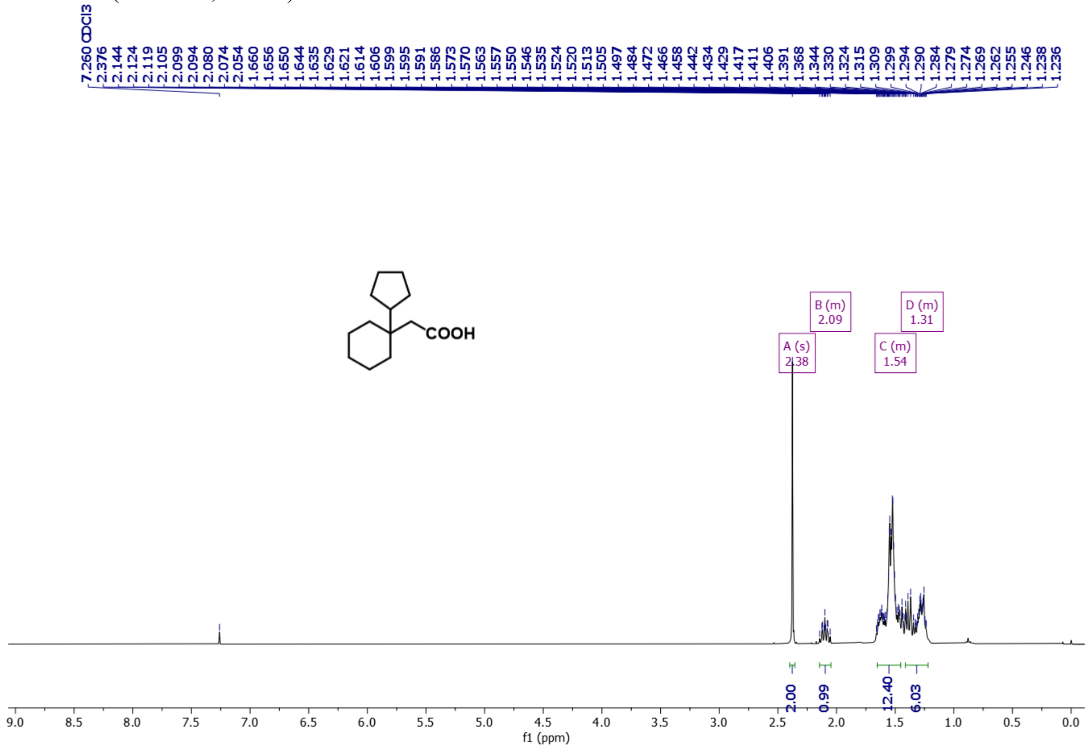

^{13}C NMR (101 MHz, $CDCl_3$)

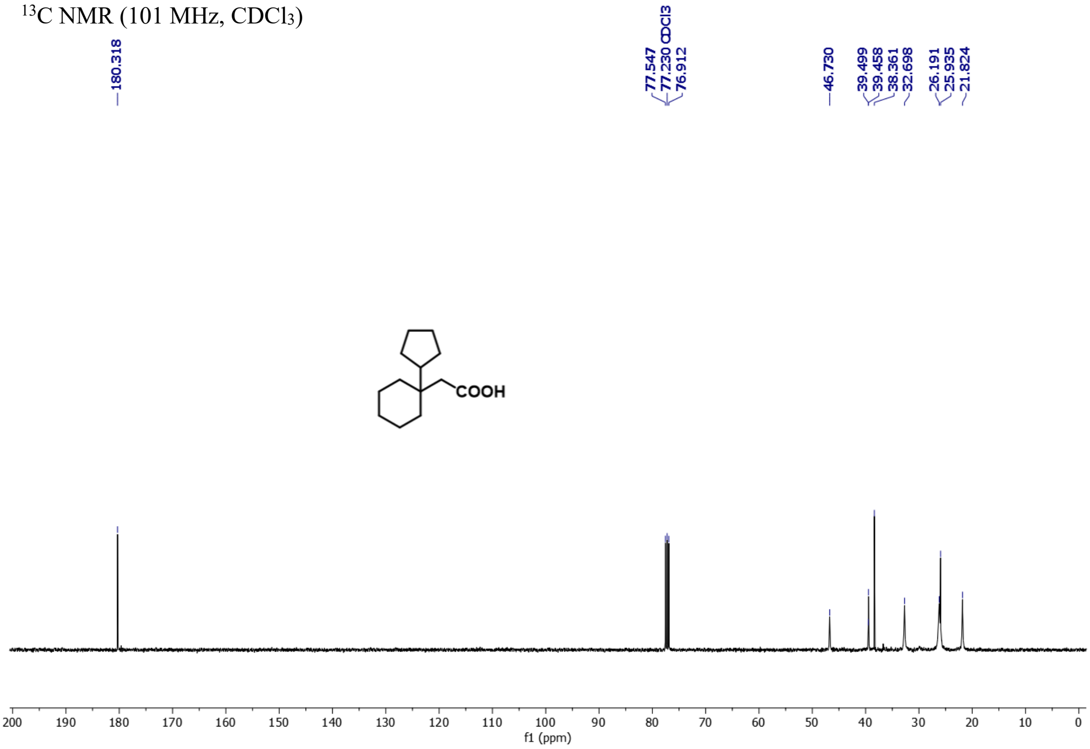

2-(1,3-Dimethylcyclopentyl)acetic acid (28)

^{1}H NMR (400 MHz, $CDCl_3$)

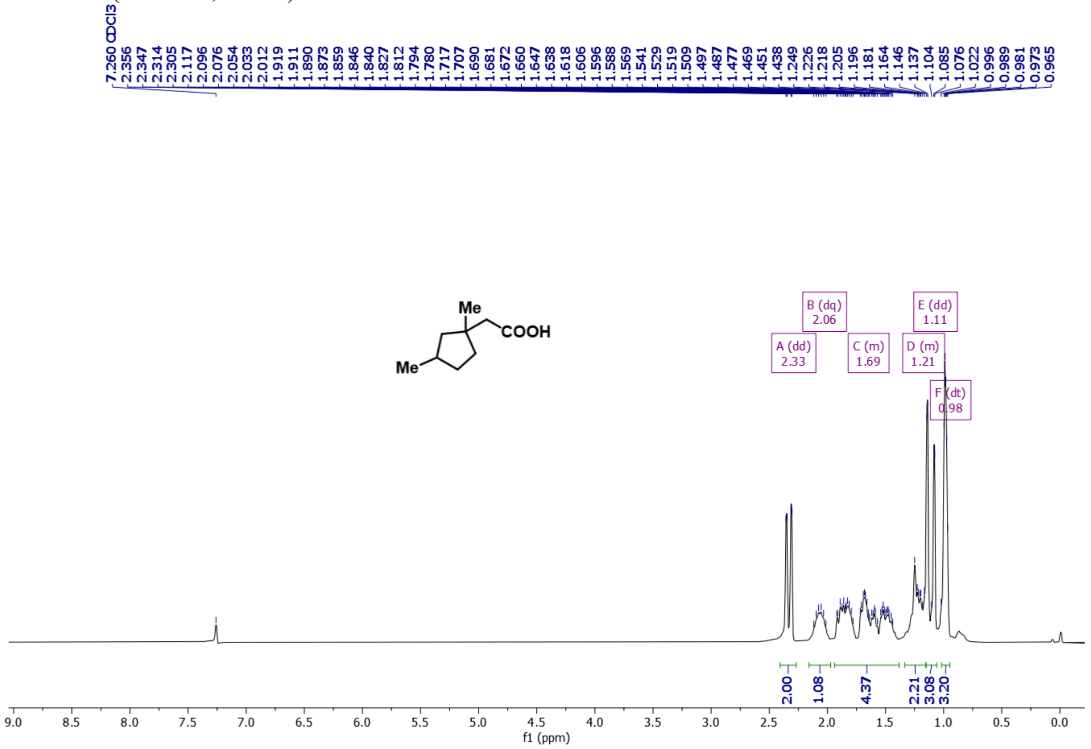

^{13}C NMR (101 MHz, $CDCl_3$)

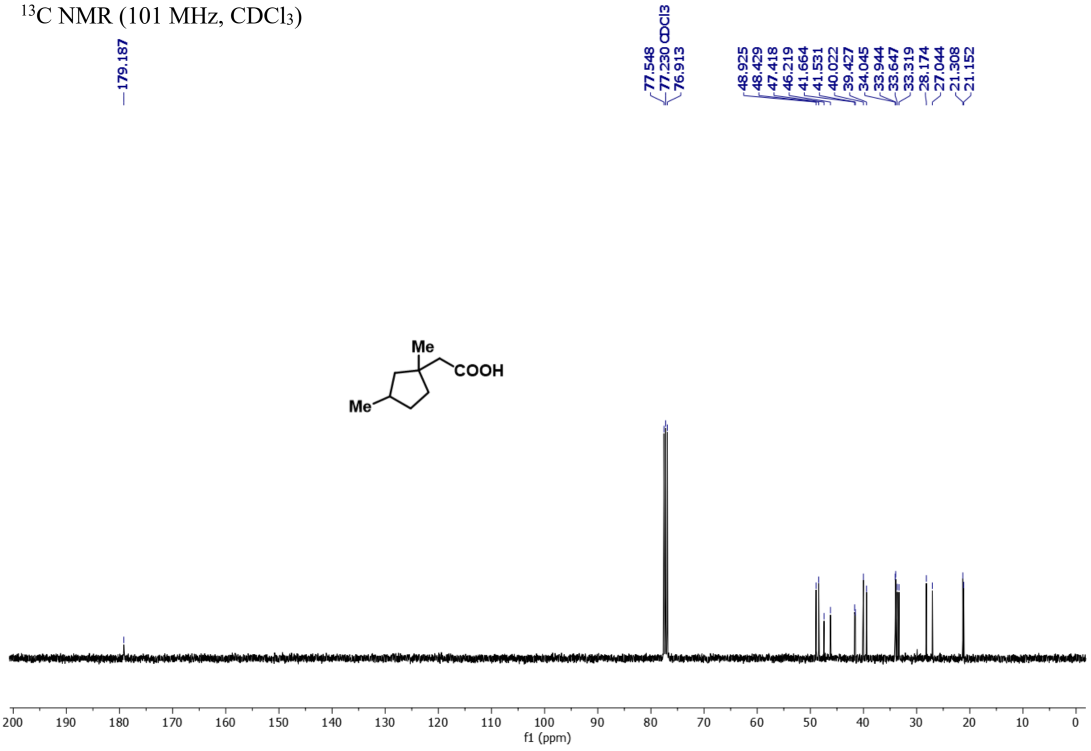

2-([1,1'-Bi(cyclohexan)]-1-yl)acetic acid (29)

^{1}H NMR (400 MHz, $CDCl_3$)

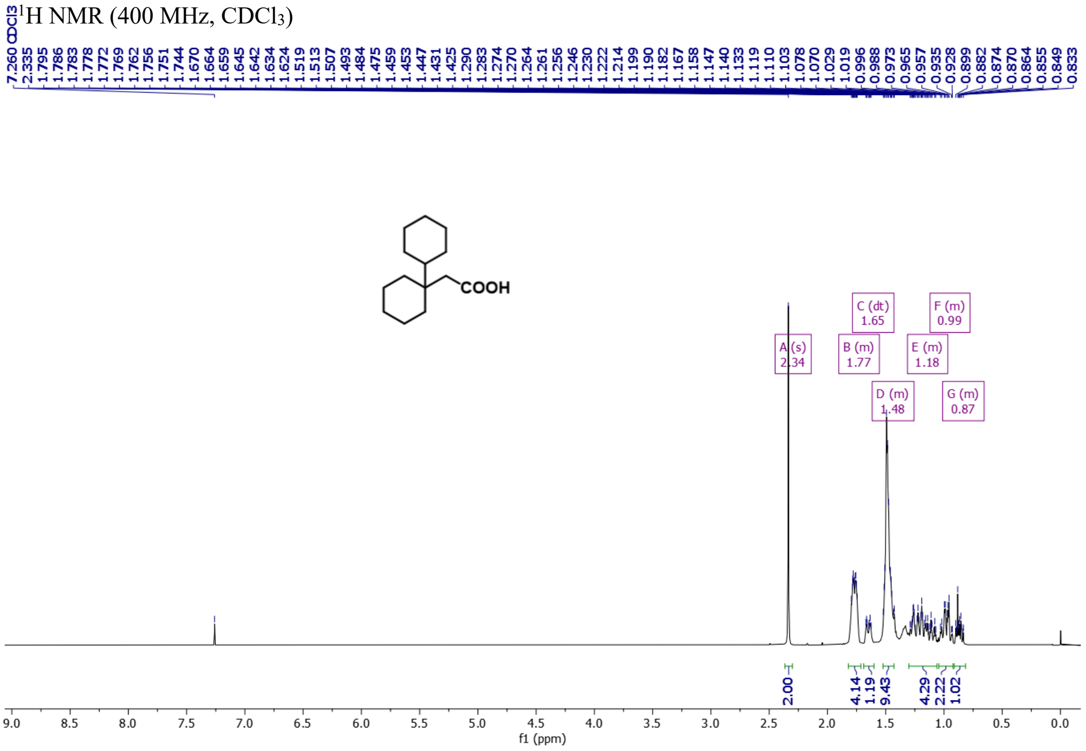

^{13}C NMR (101 MHz, $CDCl_3$)

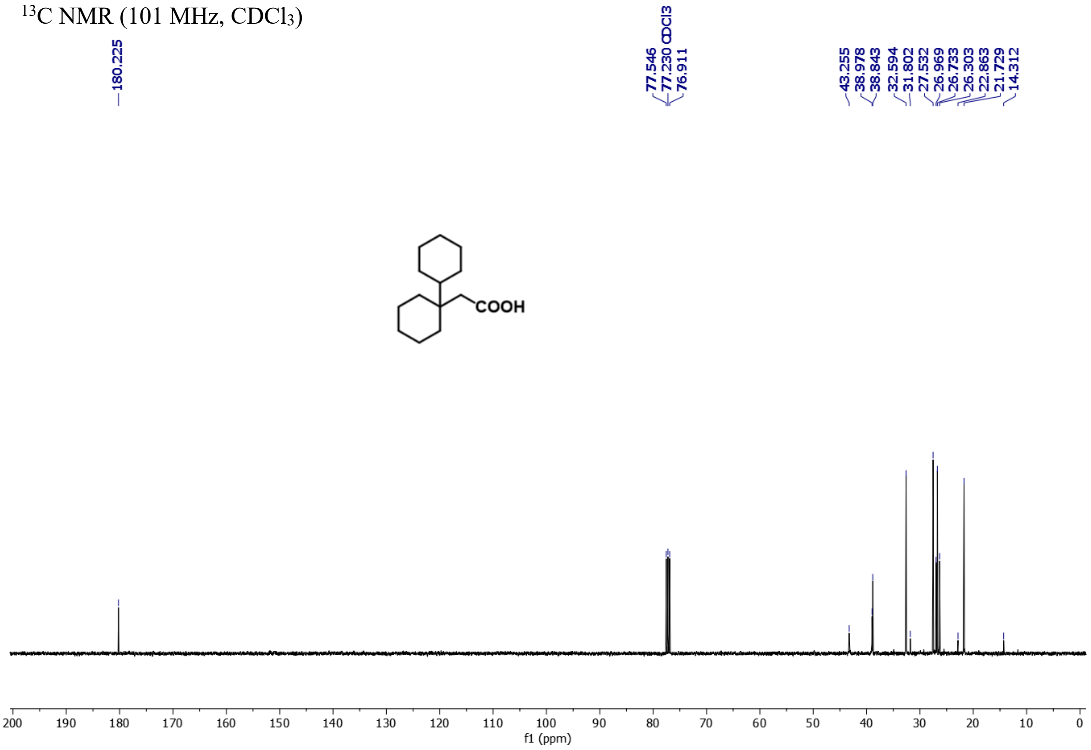

2-(1-Methyl-4-propylcyclohexyl)acetic acid (30)

^{1}H NMR (400 MHz, $CDCl_3$)

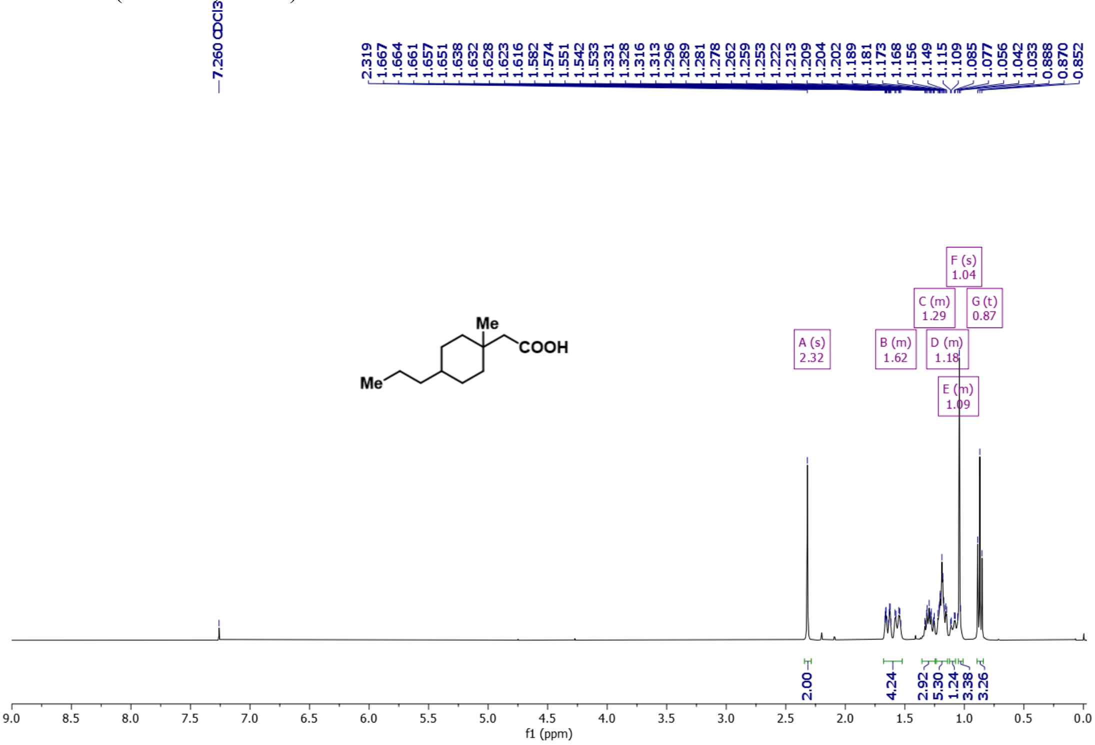

^{13}C NMR (101 MHz, $CDCl_3$)

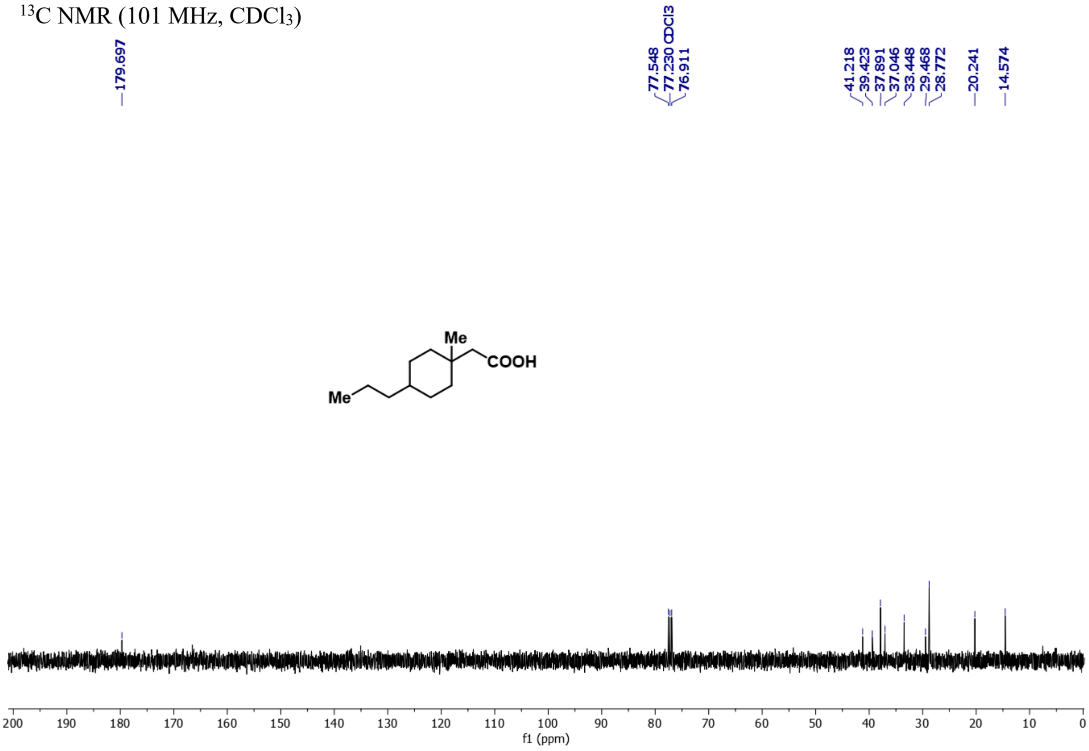

2-(1-Methyl-3-(*p*-tolyl)cyclohexyl)acetic acid (31)

^{1}H NMR (400 MHz, $CDCl_3$)

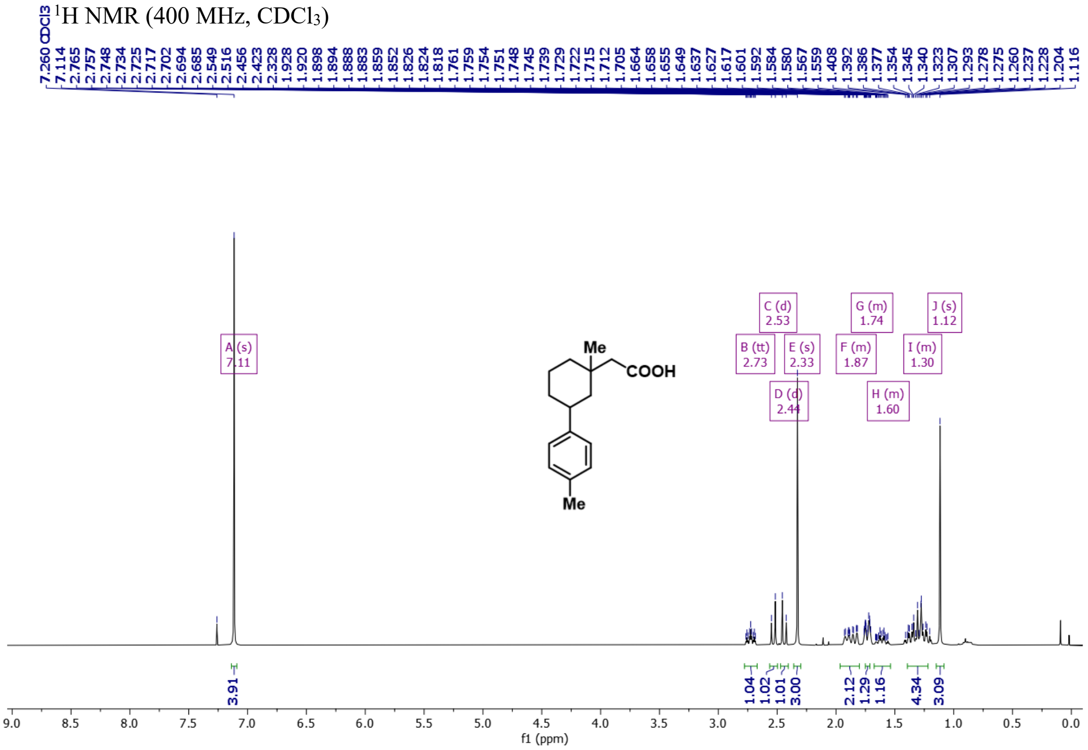

^{13}C NMR (101 MHz, $CDCl_3$)

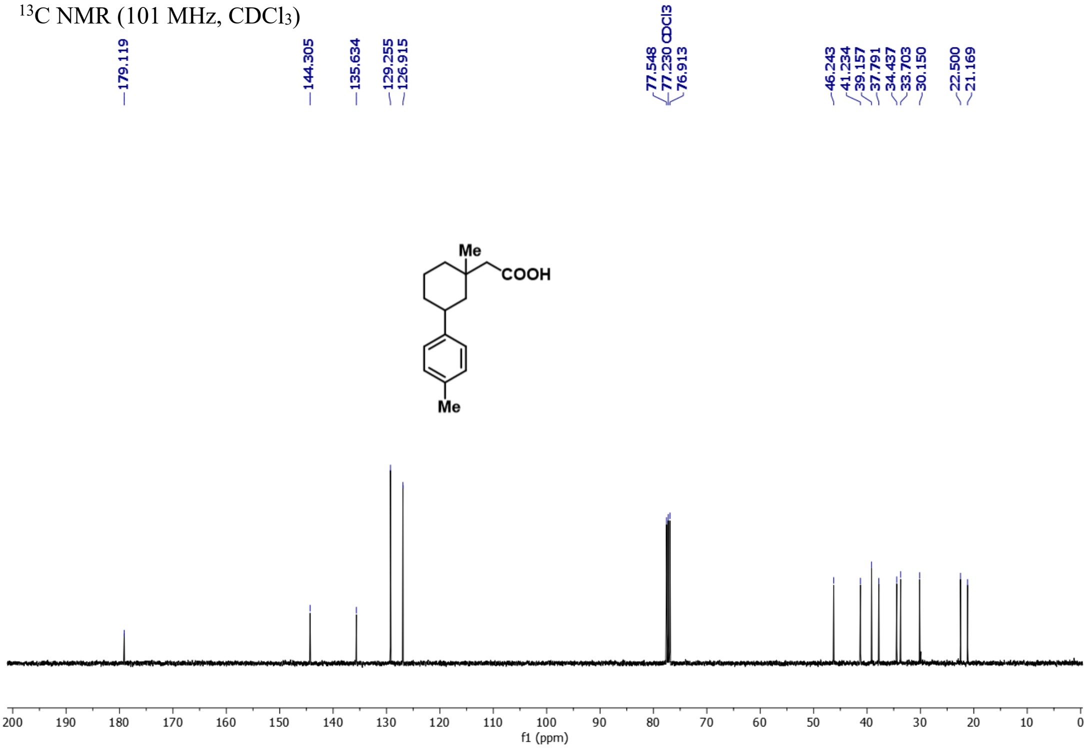

2-(1-Isopropylcycloheptyl)acetic acid (32)

^{1}H NMR (500 MHz, $CDCl_3$)

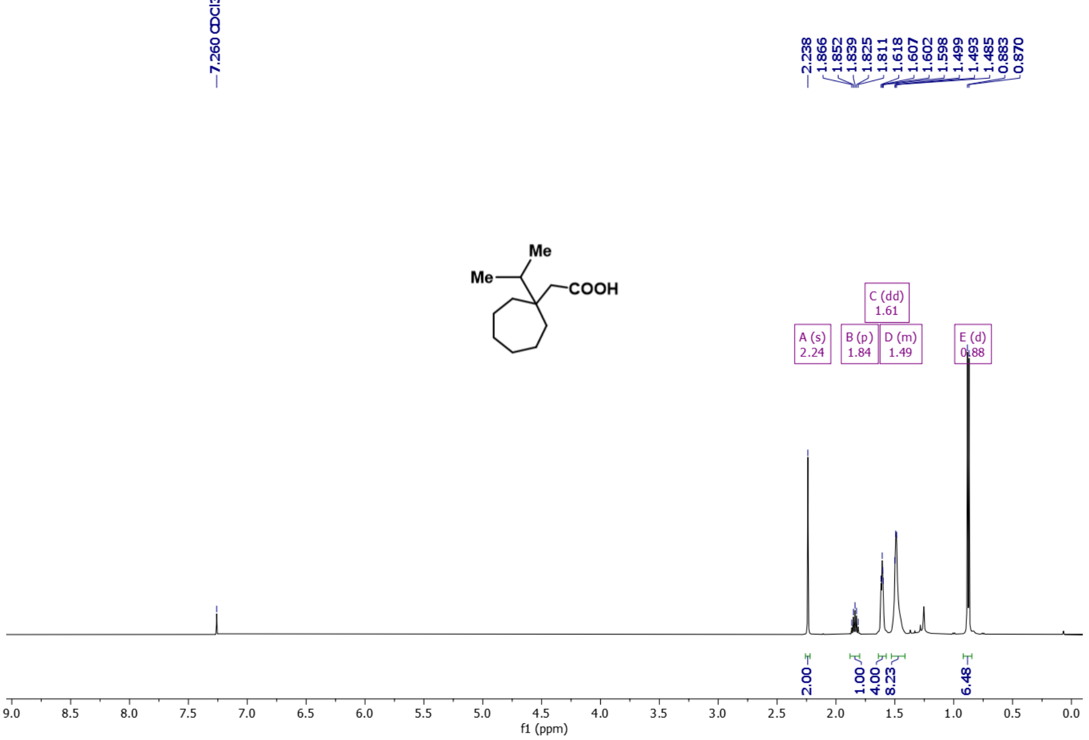

^{13}C NMR (126 MHz, $CDCl_3$)

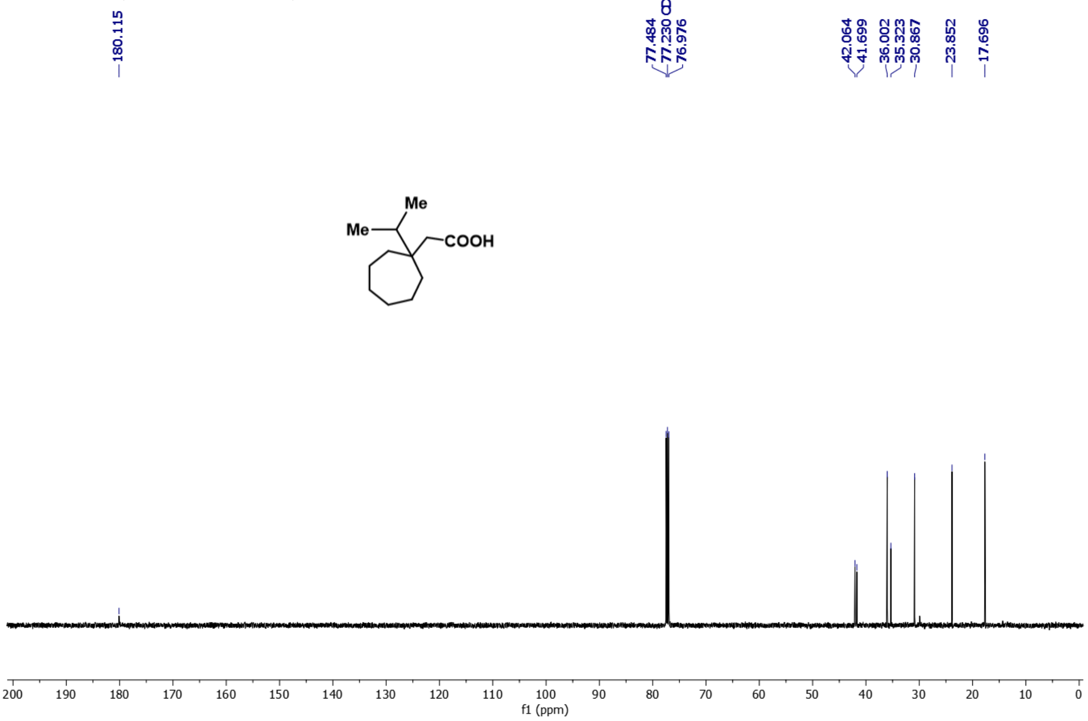

2-(1-Cyclohexylcyclopentyl)acetic acid (33)

^{1}H NMR (400 MHz, $CDCl_3$)

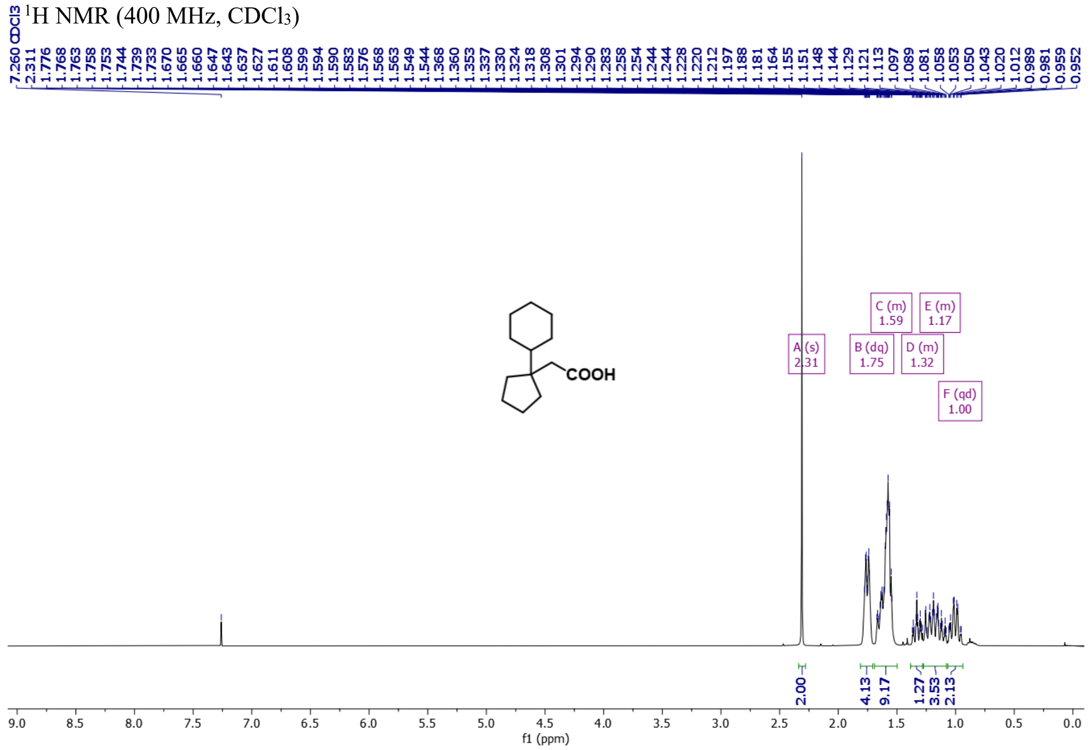

^{13}C NMR (101 MHz, $CDCl_3$)

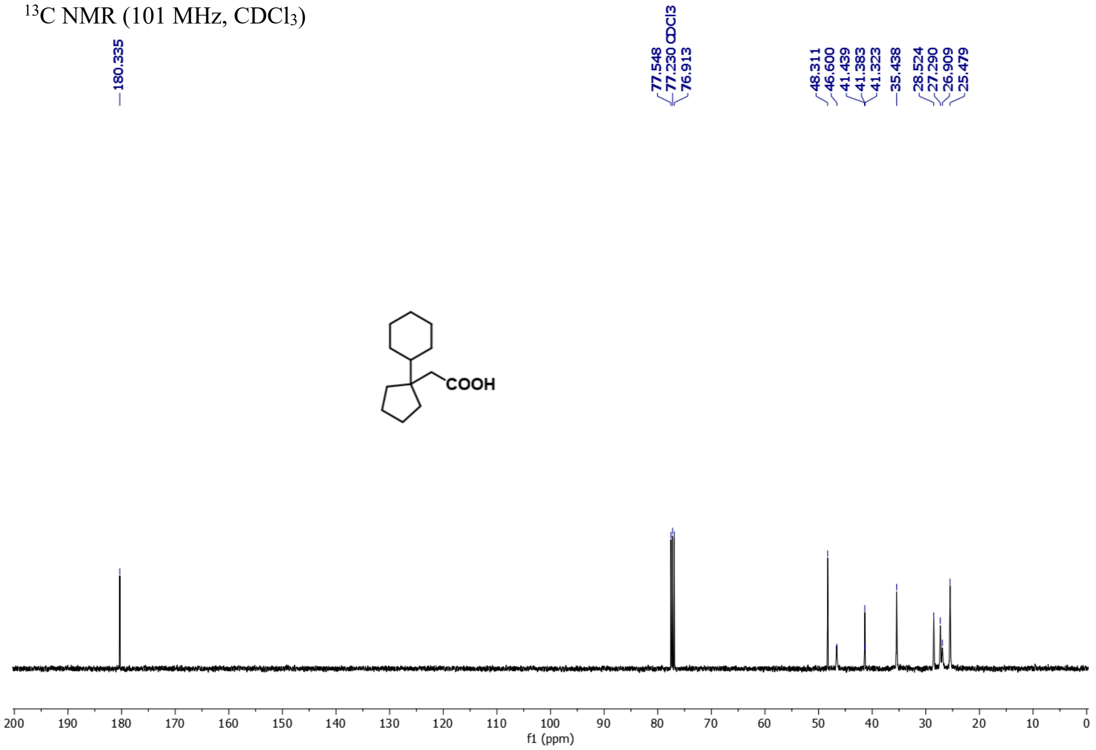

2-(1-Methylcyclopentadecyl)acetic acid (34)

^{1}H NMR (400 MHz, $CDCl_3$)

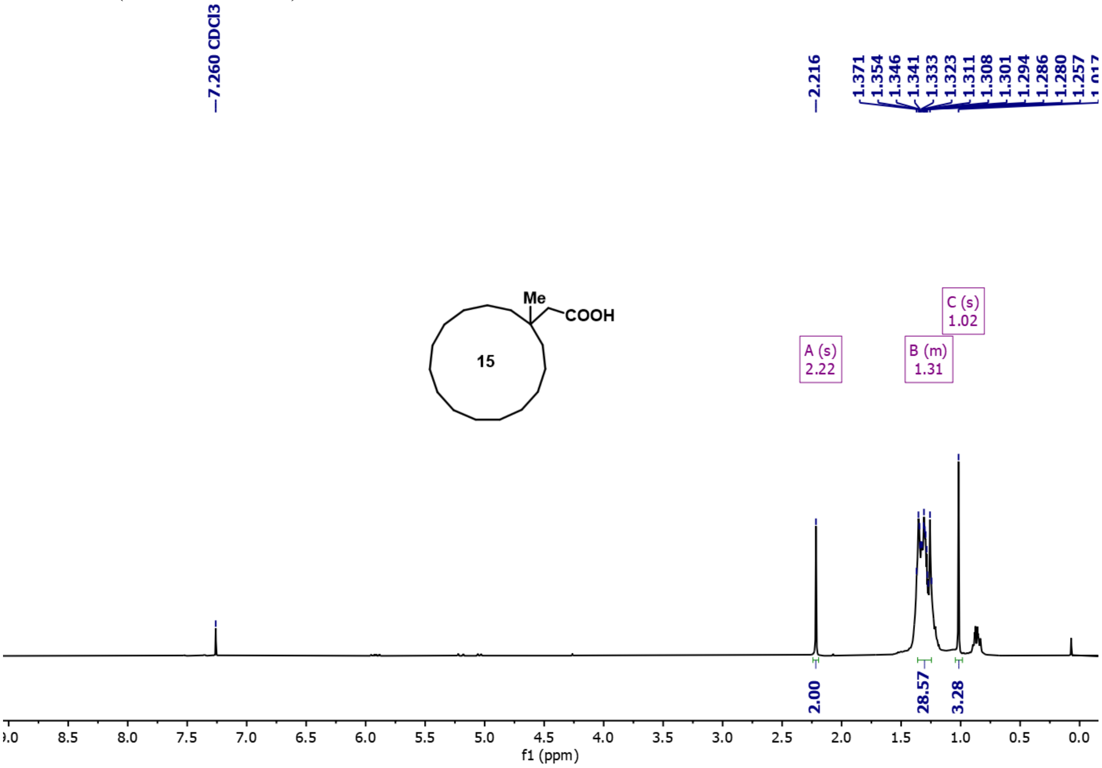

^{13}C NMR (101 MHz, $CDCl_3$)

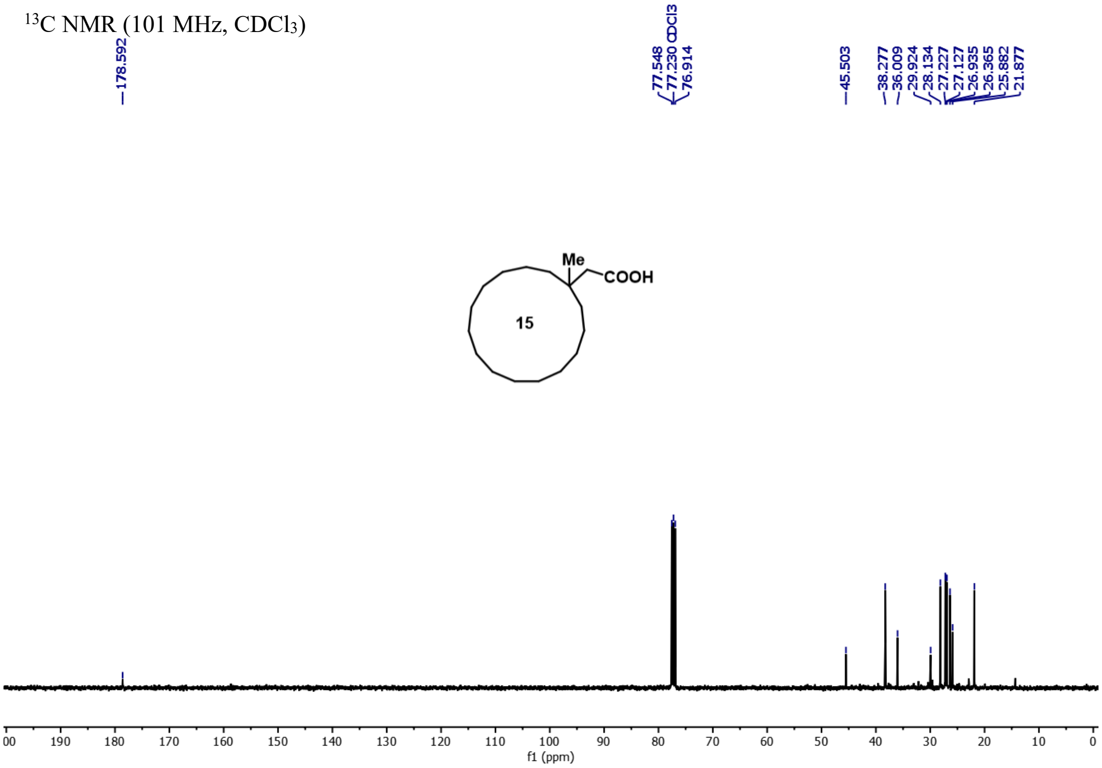

2-(1-Ethyl-3-methylcyclopentyl)acetic acid (35)

^{1}H NMR (500 MHz, $CDCl_3$)

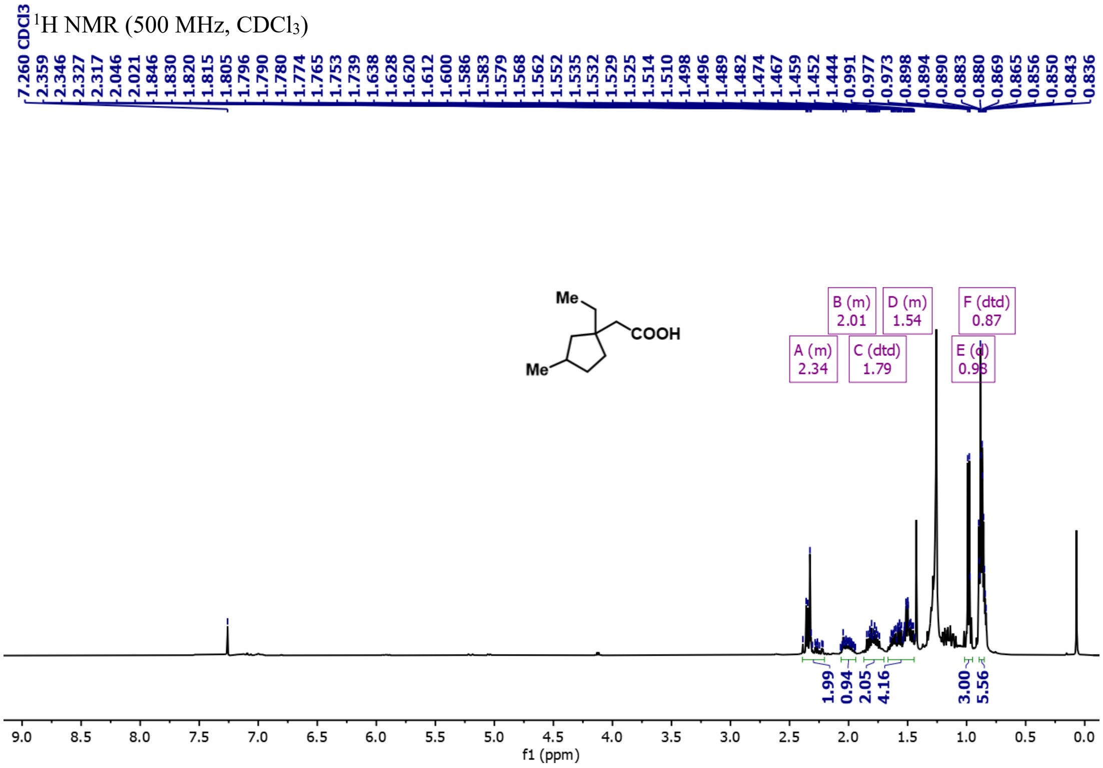

^{13}C NMR (126 MHz, $CDCl_3$)

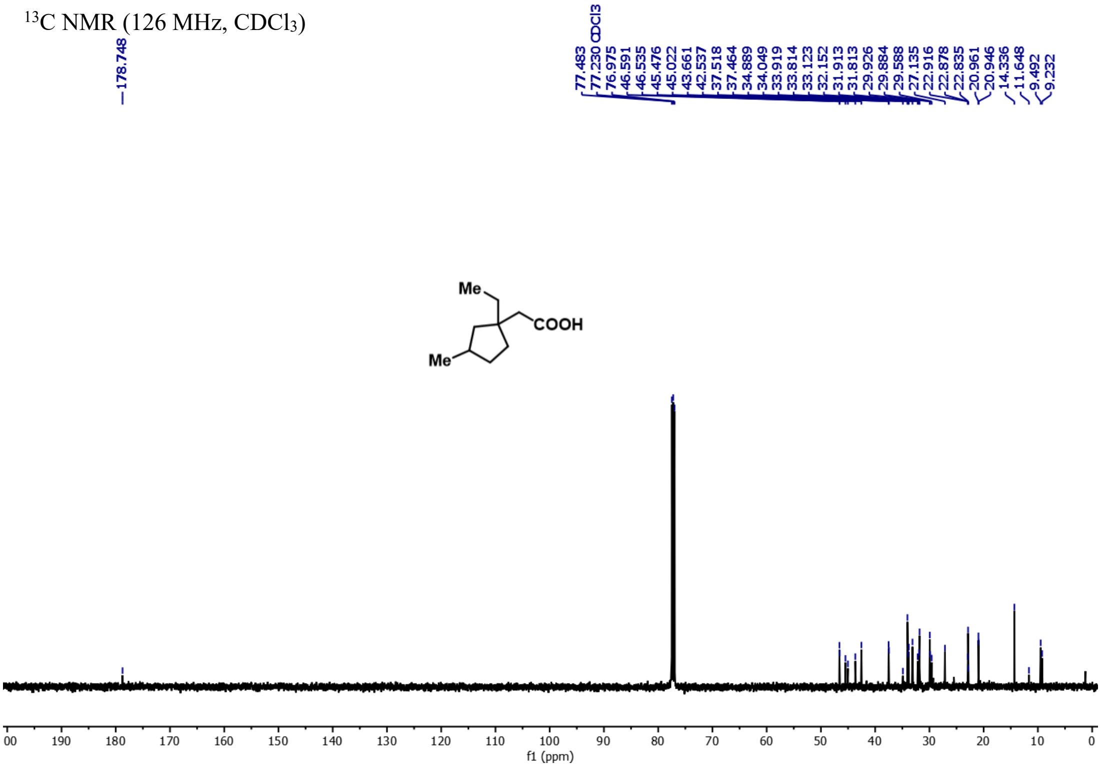

2-(1-Cyclohexylcycloheptyl)acetic acid (36)

^{1}H NMR (400 MHz, $CDCl_3$)

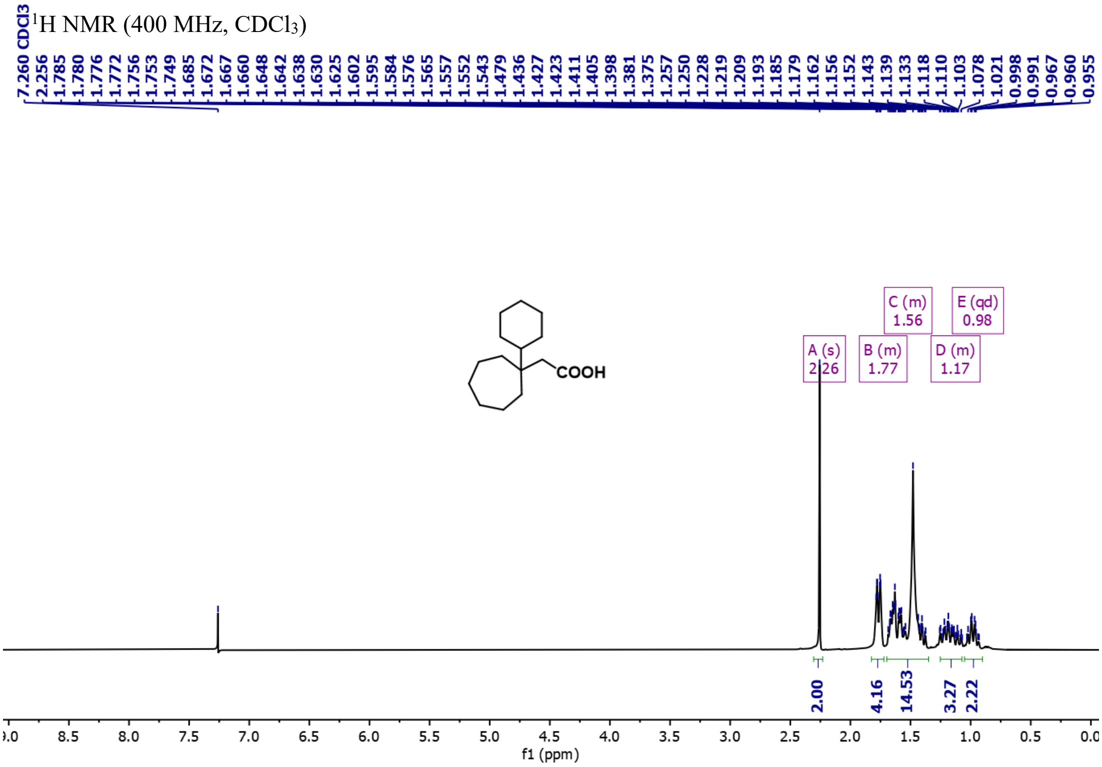

^{13}C NMR (101 MHz, $CDCl_3$)

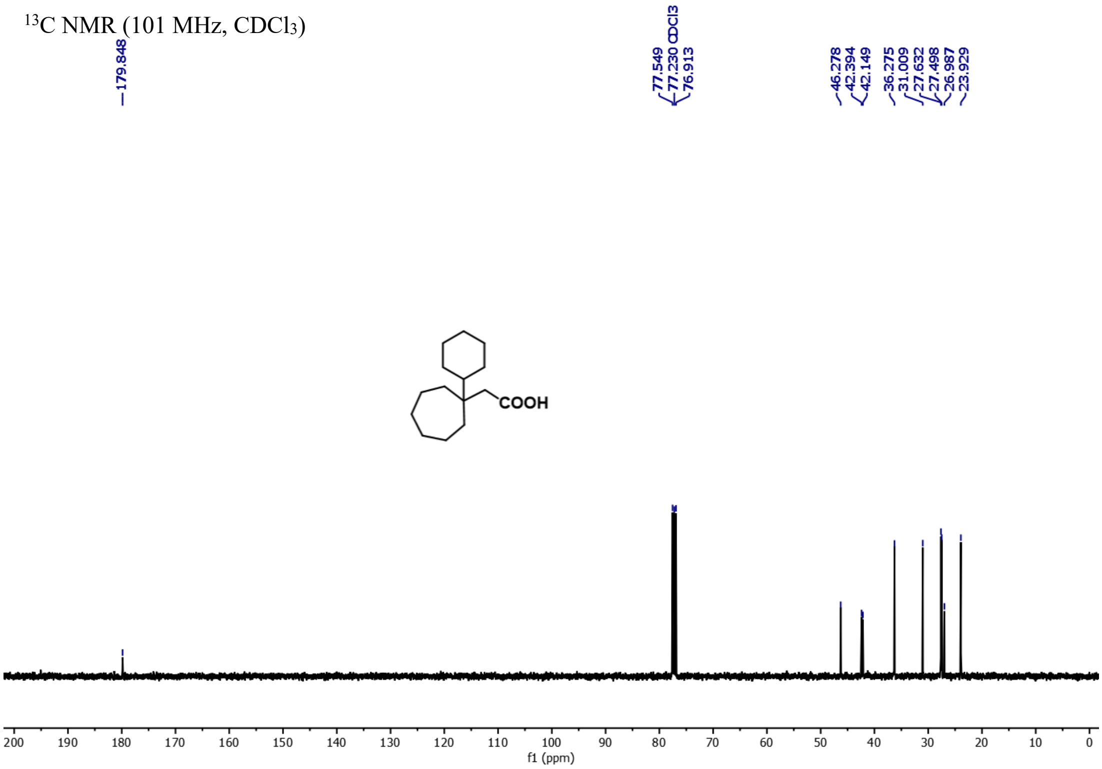

2-(1-Octylcyclohexyl)acetic acid (37)

^1H NMR (400 MHz, $CDCl_3$)

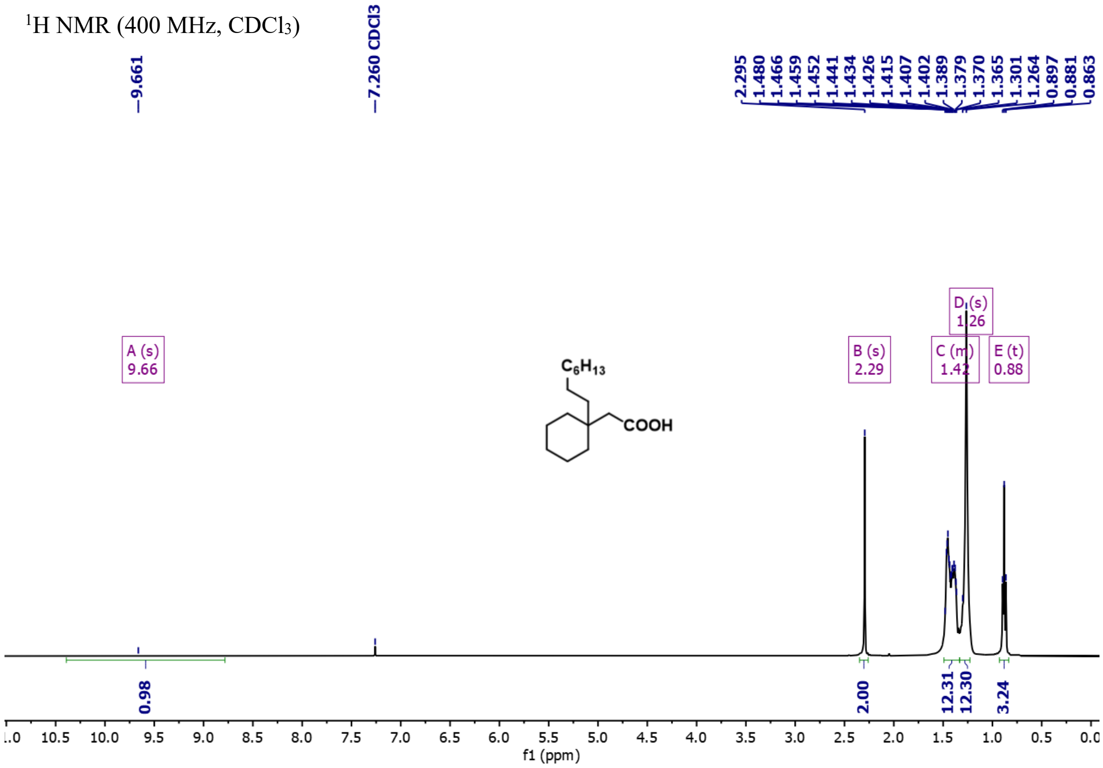

^{13}C NMR (101 MHz, $CDCl_3$)

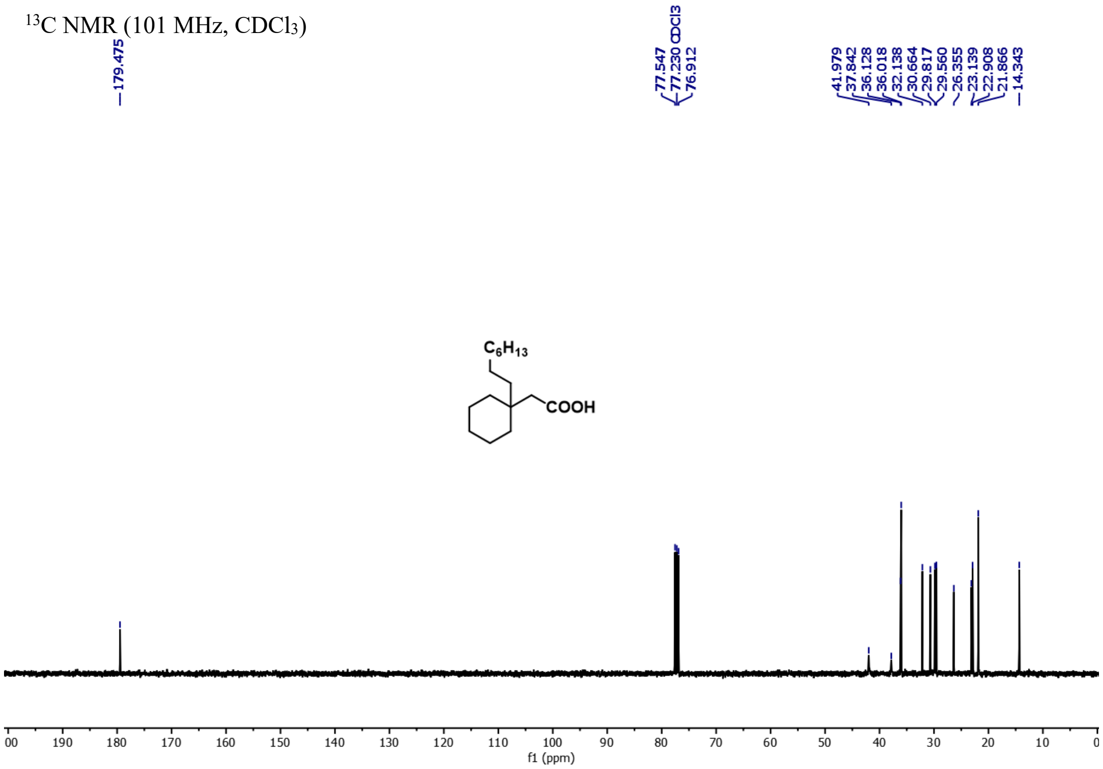

2-(1-(3-Methoxyphenyl)cyclohexyl)acetic acid (38)

^{1}H NMR (400 MHz, $CDCl_3$)

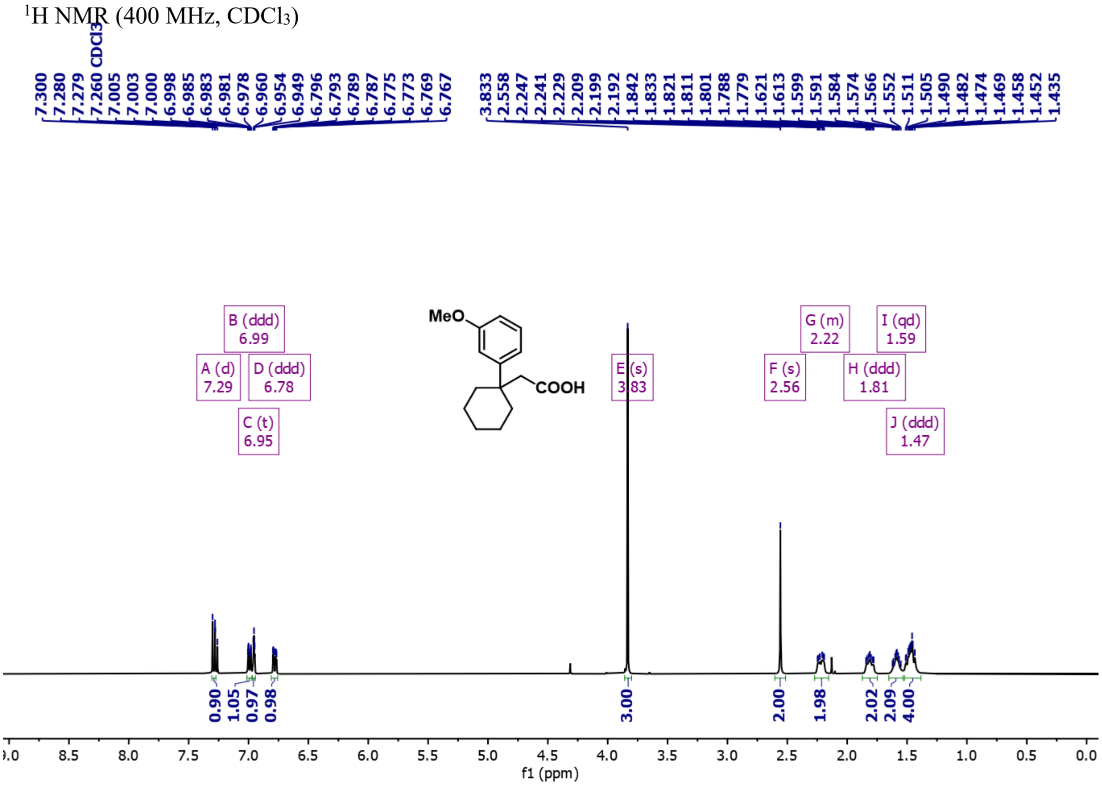

^{13}C NMR (101 MHz, $CDCl_3$)

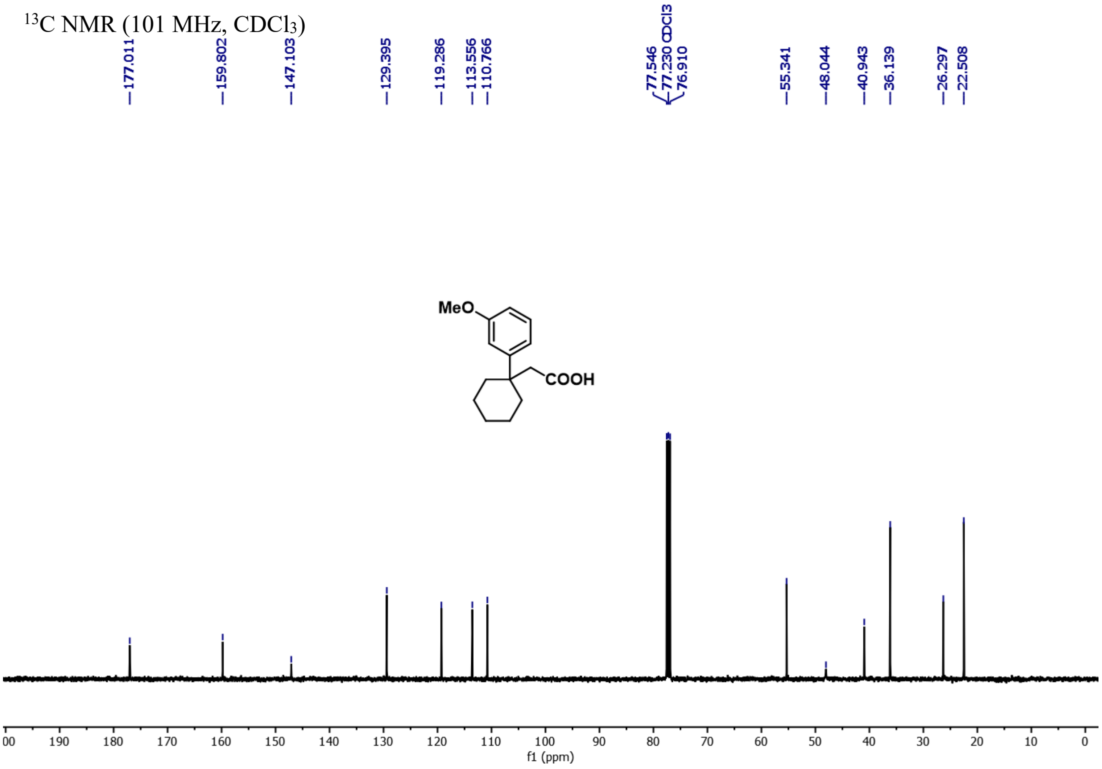

2-(1-Ethylcyclododecyl)acetic acid (39)

^{1}H NMR (400 MHz, $CDCl_3$)

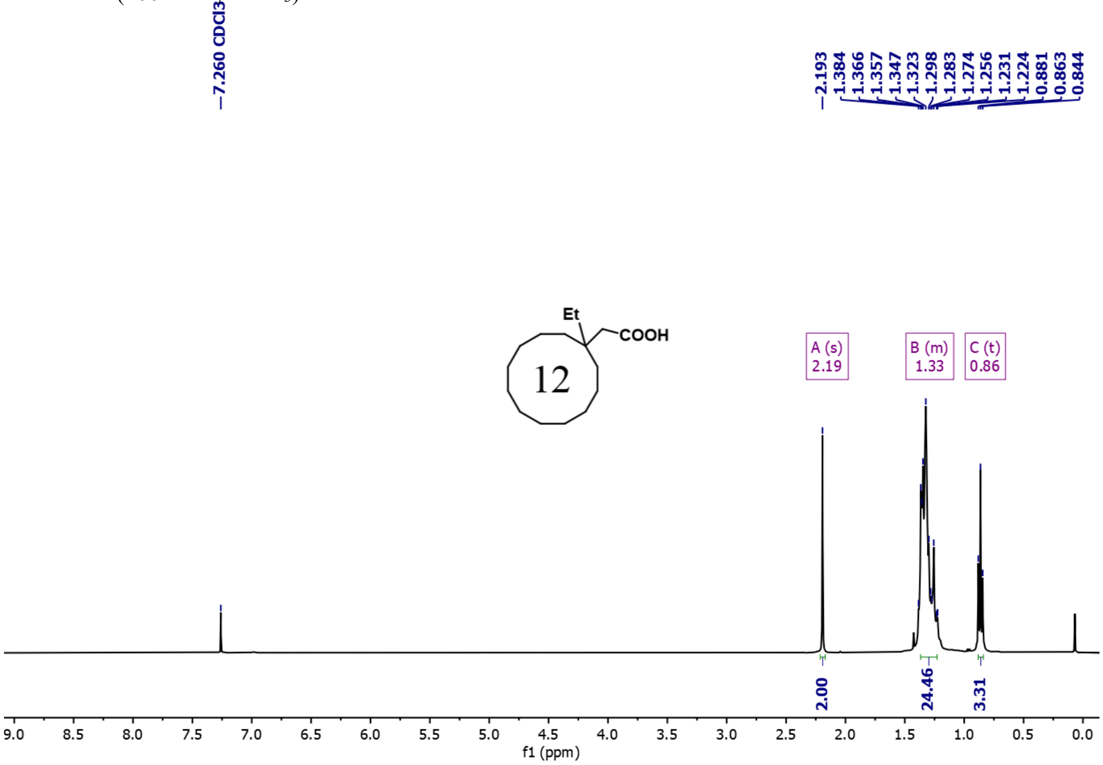

^{13}C NMR (101 MHz, $CDCl_3$)

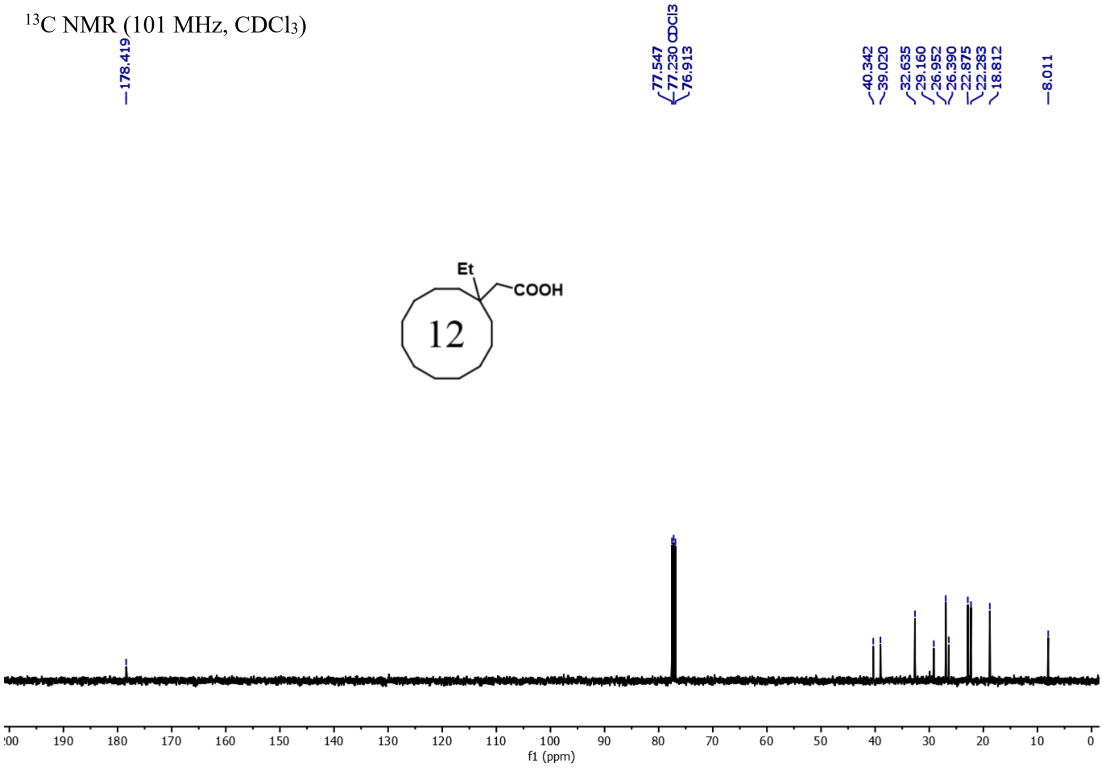

2-(1-Isobutylcycloheptyl)acetic acid (40)

^{1}H NMR (400 MHz, $CDCl_3$)

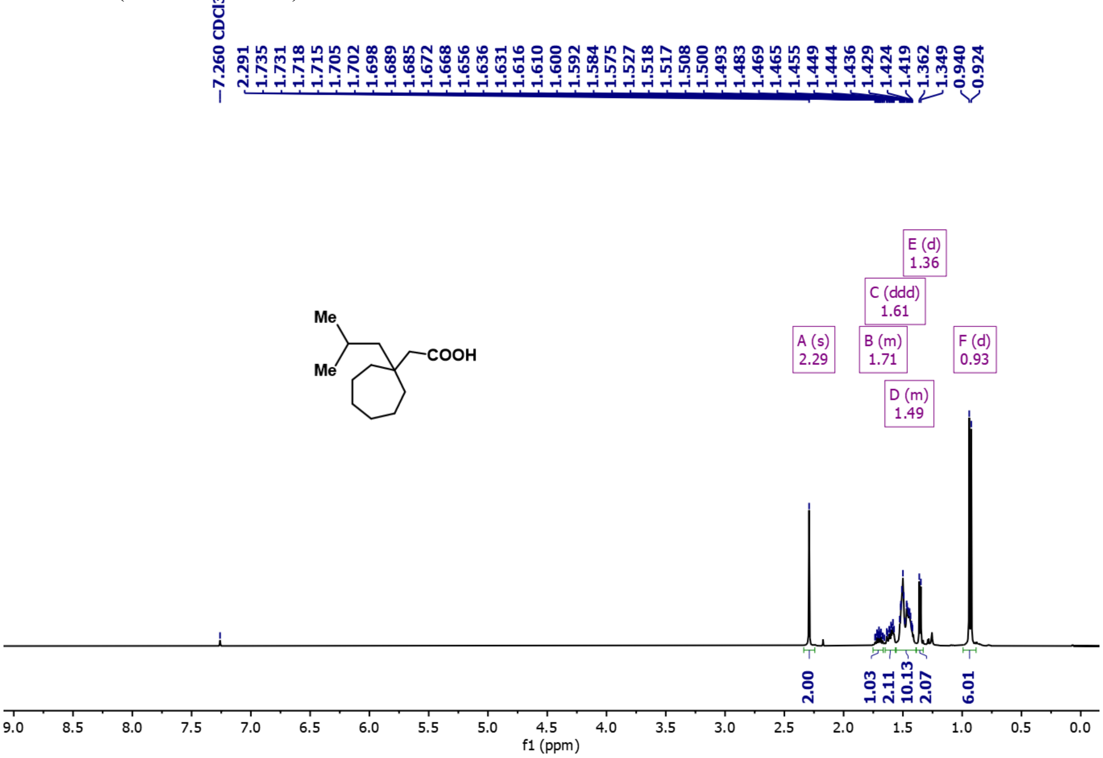

^{13}C NMR (101 MHz, $CDCl_3$)

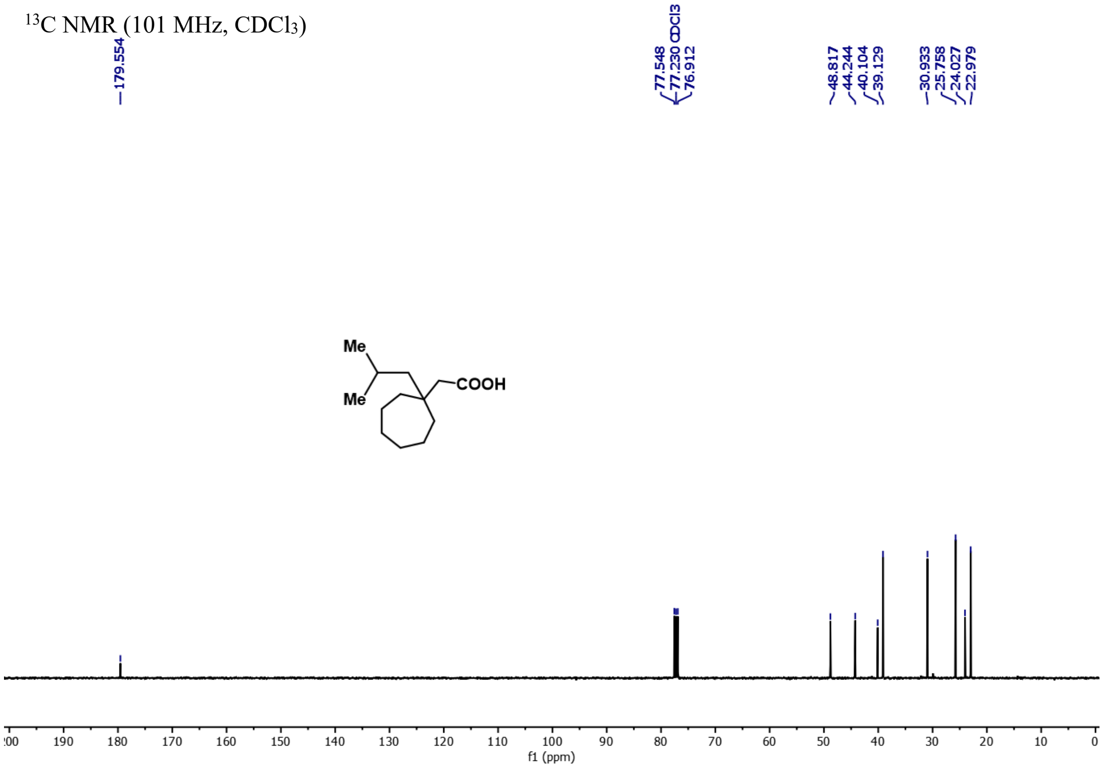

2-(1-Methyl-4-(trifluoromethyl)cyclohexyl)acetic acid (41)

^{1}H NMR (400 MHz, $CDCl_3$)

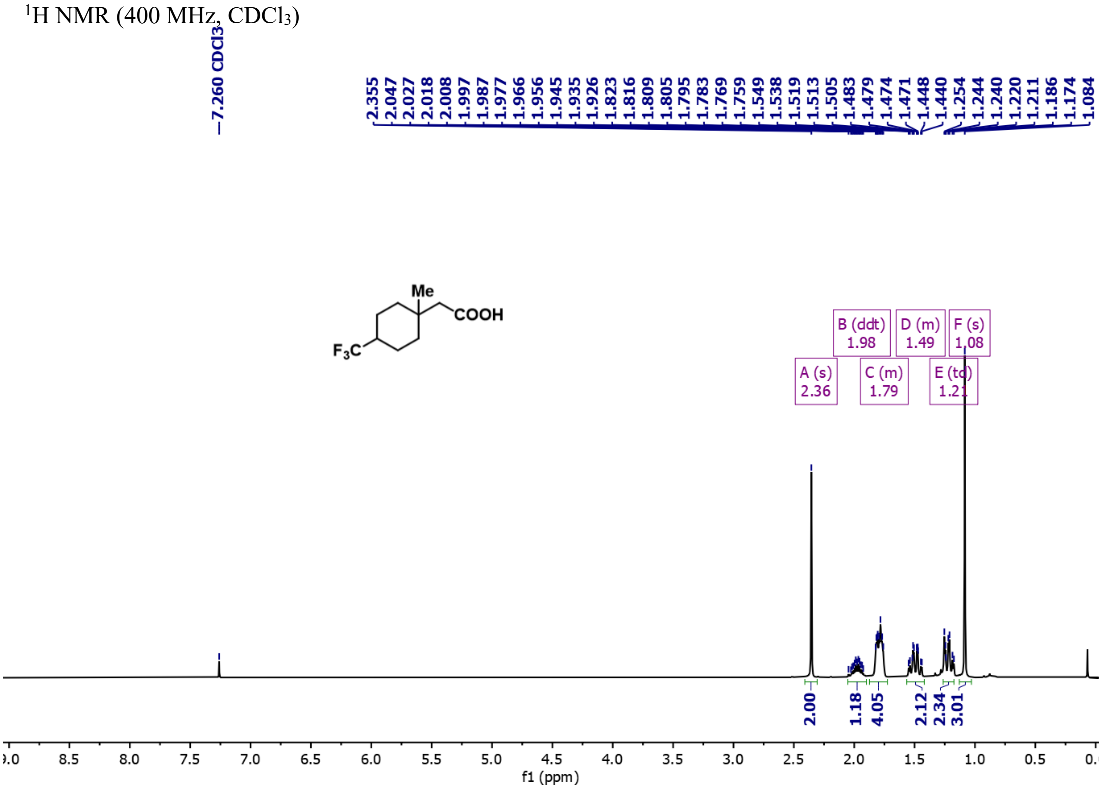

^{13}C NMR (101 MHz, $CDCl_3$)

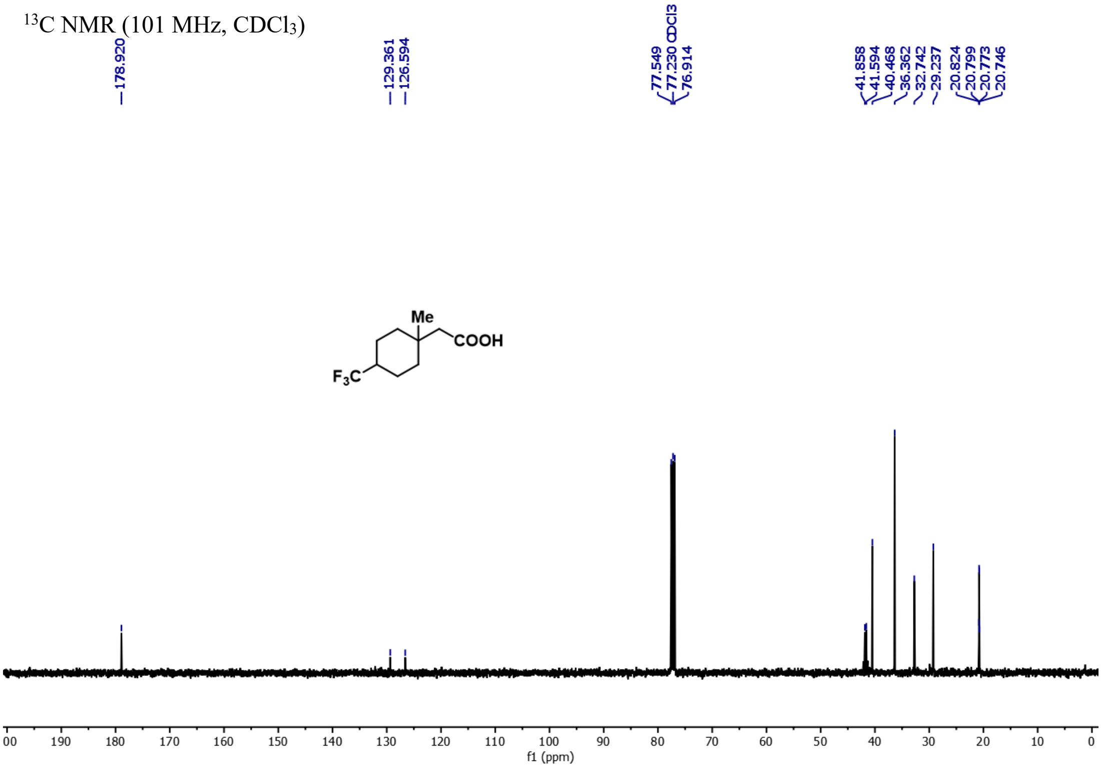

^{19}F NMR 376 MHz, $CDCl_3$)

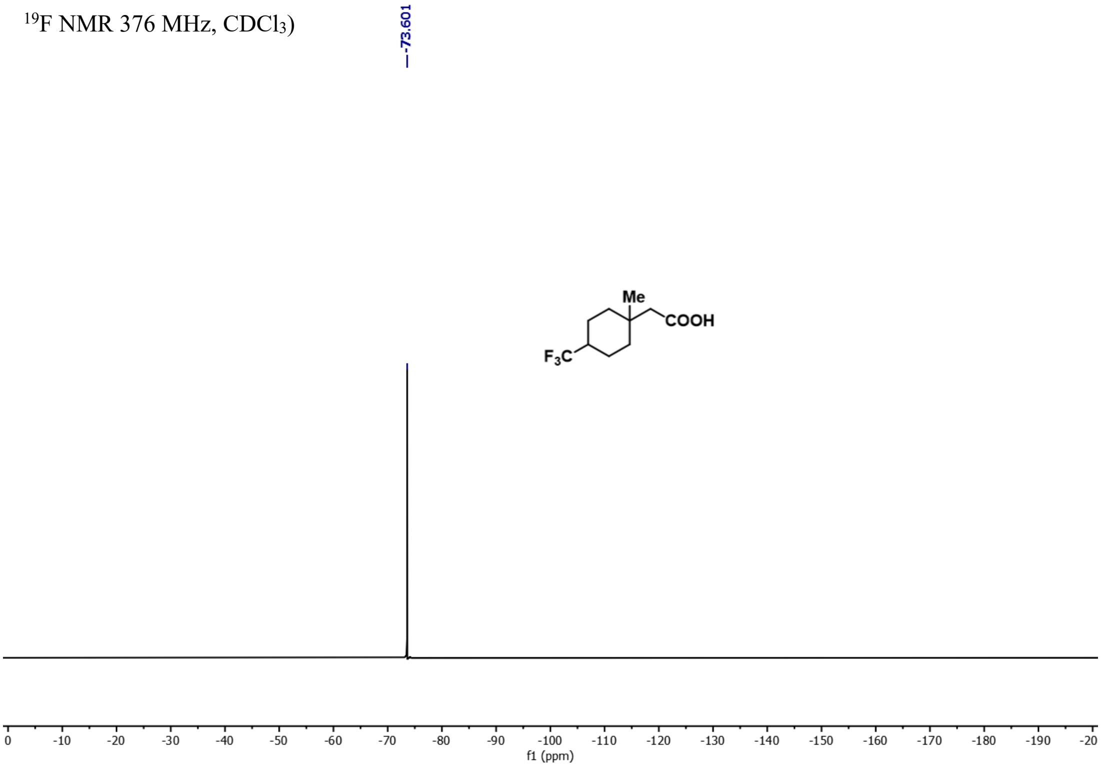

2-(1-(3,4-Dimethoxyphenyl)cyclohexyl)acetic acid (42)

^{1}H NMR (500 MHz, $CDCl_3$)

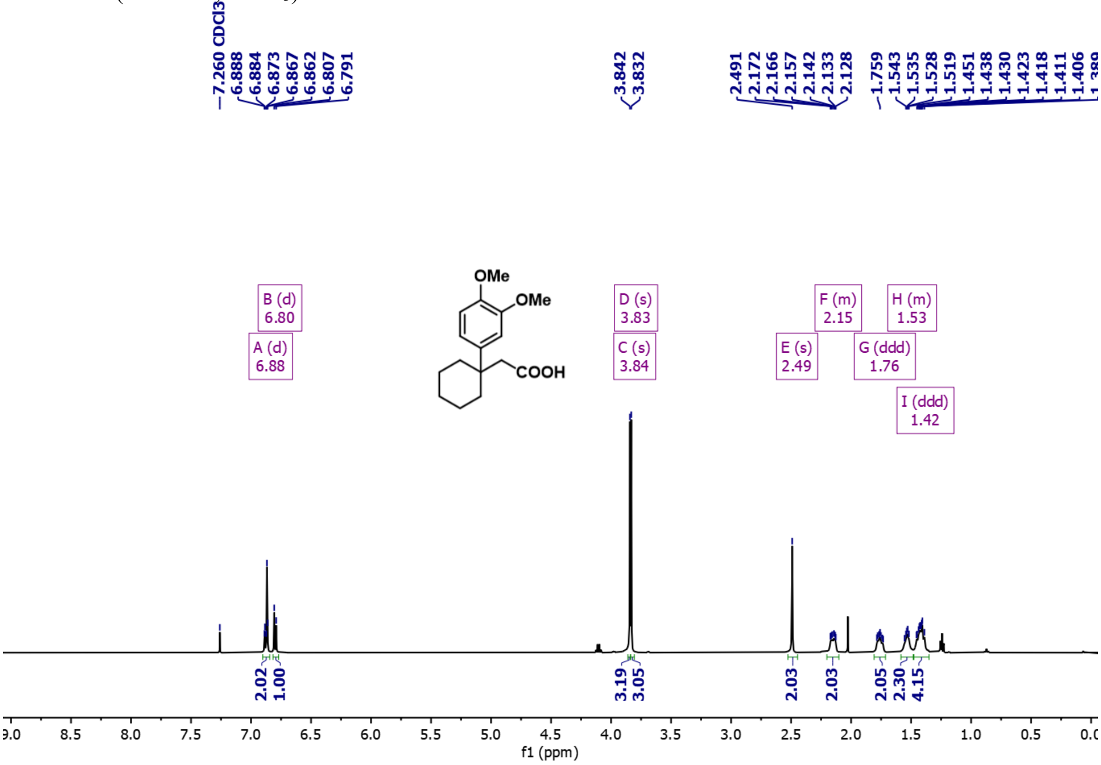

^{13}C NMR (126 MHz, $CDCl_3$)

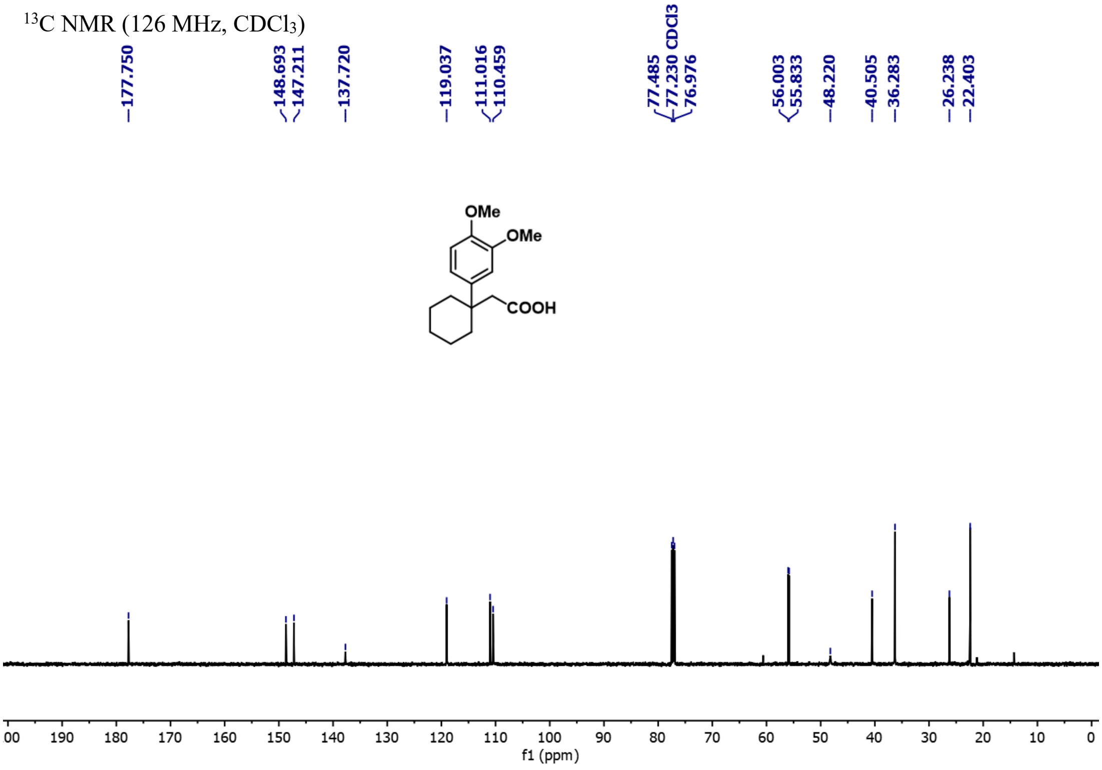

2-(1-Methyl-4-(*p*-tolyl)cycloheptyl)acetic acid (43)

^{1}H NMR (400 MHz, $CDCl_3$)

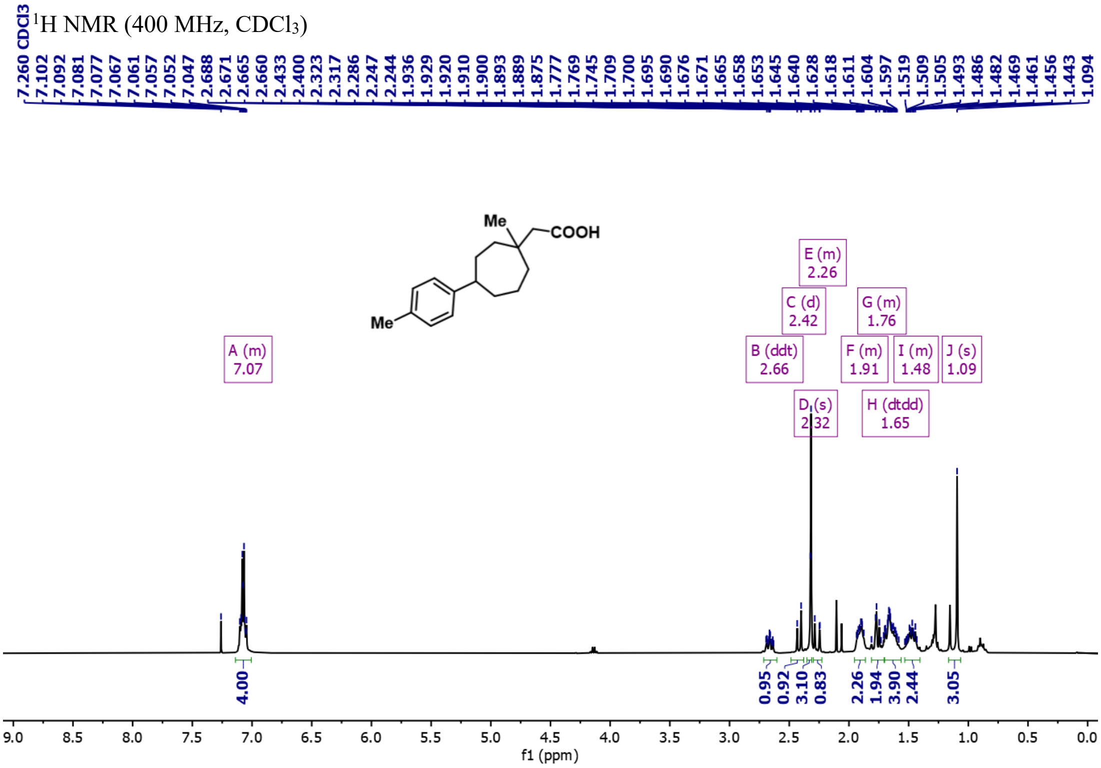

^{13}C NMR (101 MHz, $CDCl_3$)

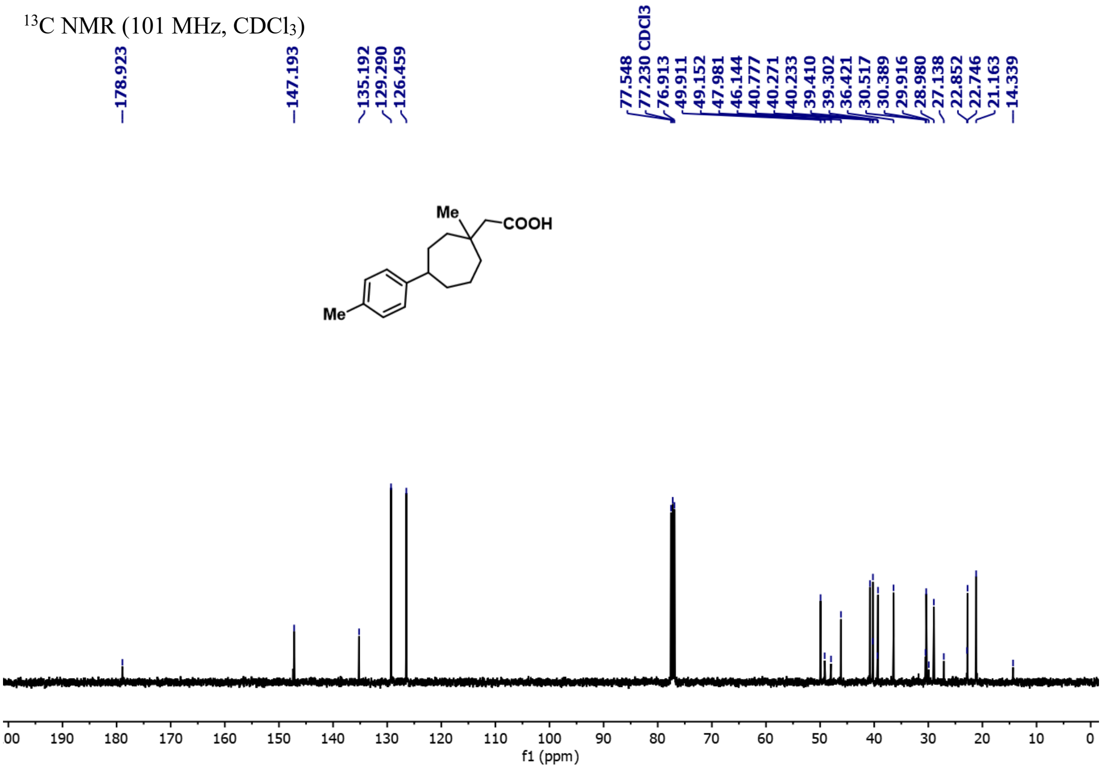

2-(1-Ethylcyclopentadecyl)acetic acid (44)

^{1}H NMR (400 MHz, $CDCl_3$)

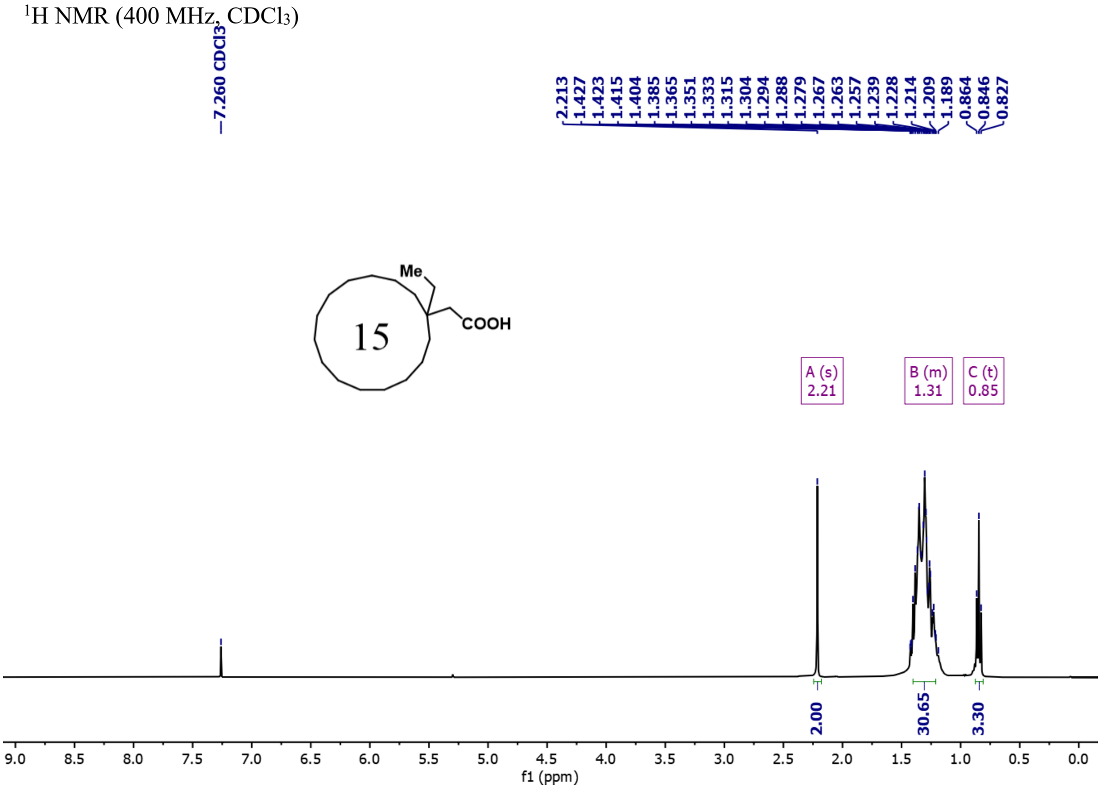

^{13}C NMR (101 MHz, $CDCl_3$)

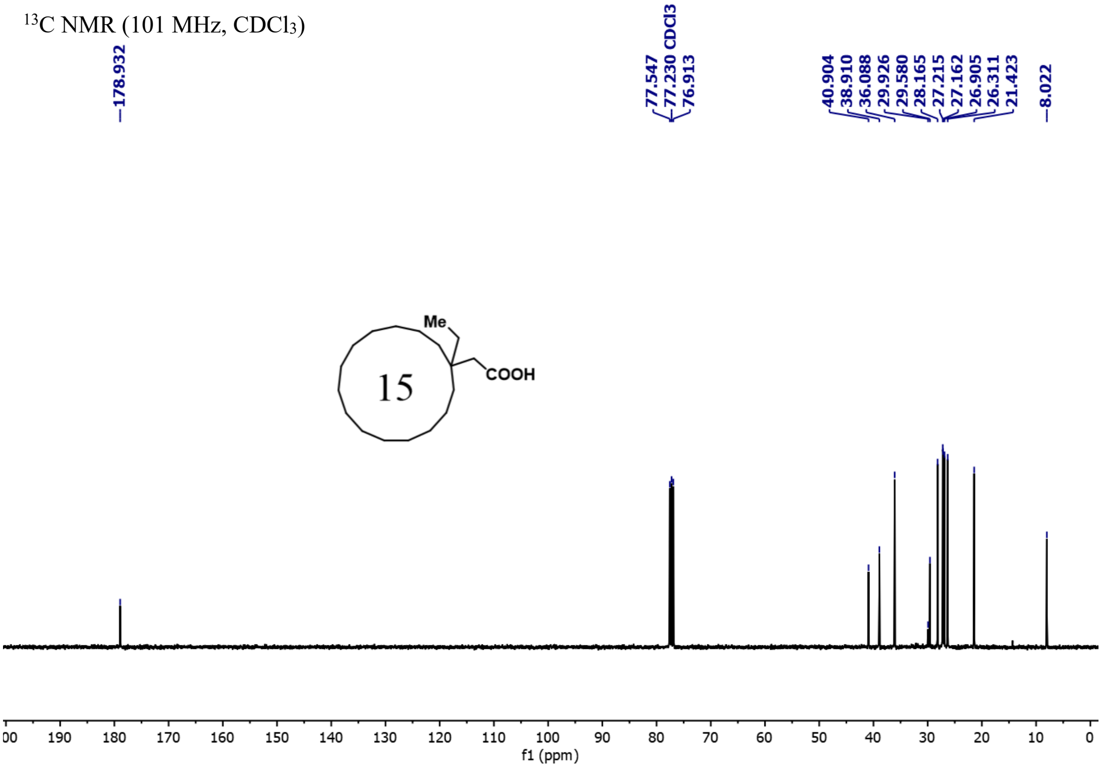

2-(1-Isobutyl-3-methylcyclohexyl)acetic acid (45)

^{1}H NMR (400 MHz, $CDCl_3$)

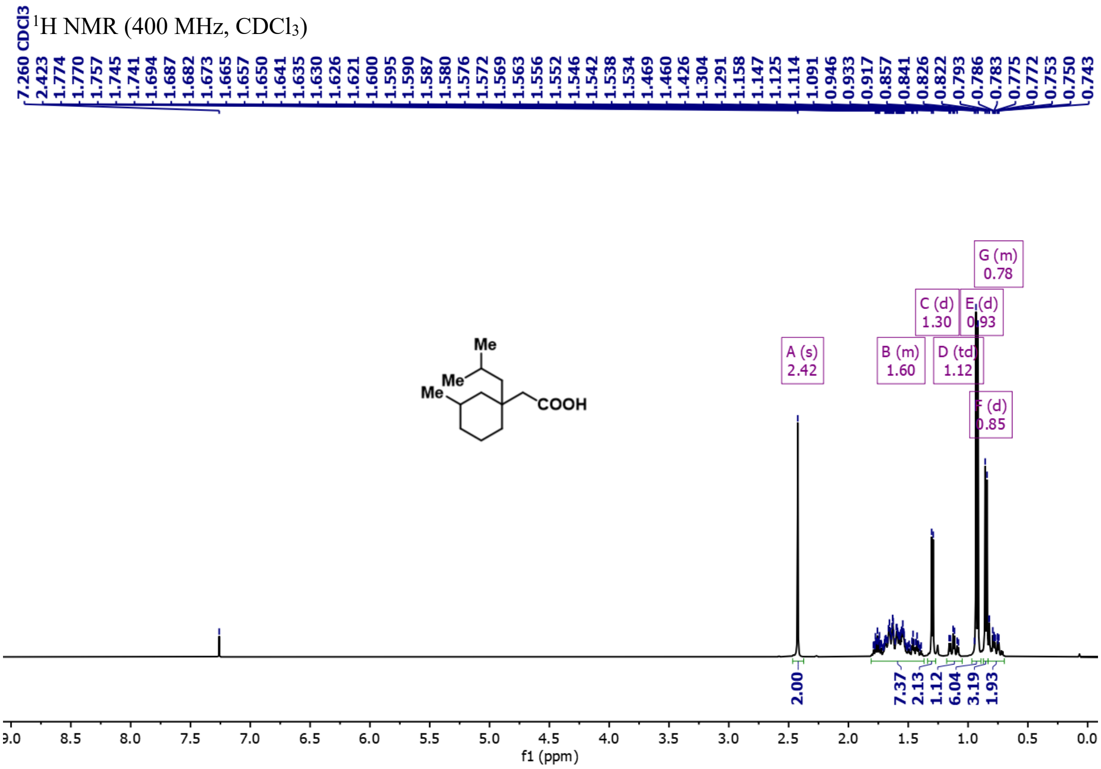

^{13}C NMR (101 MHz, $CDCl_3$)

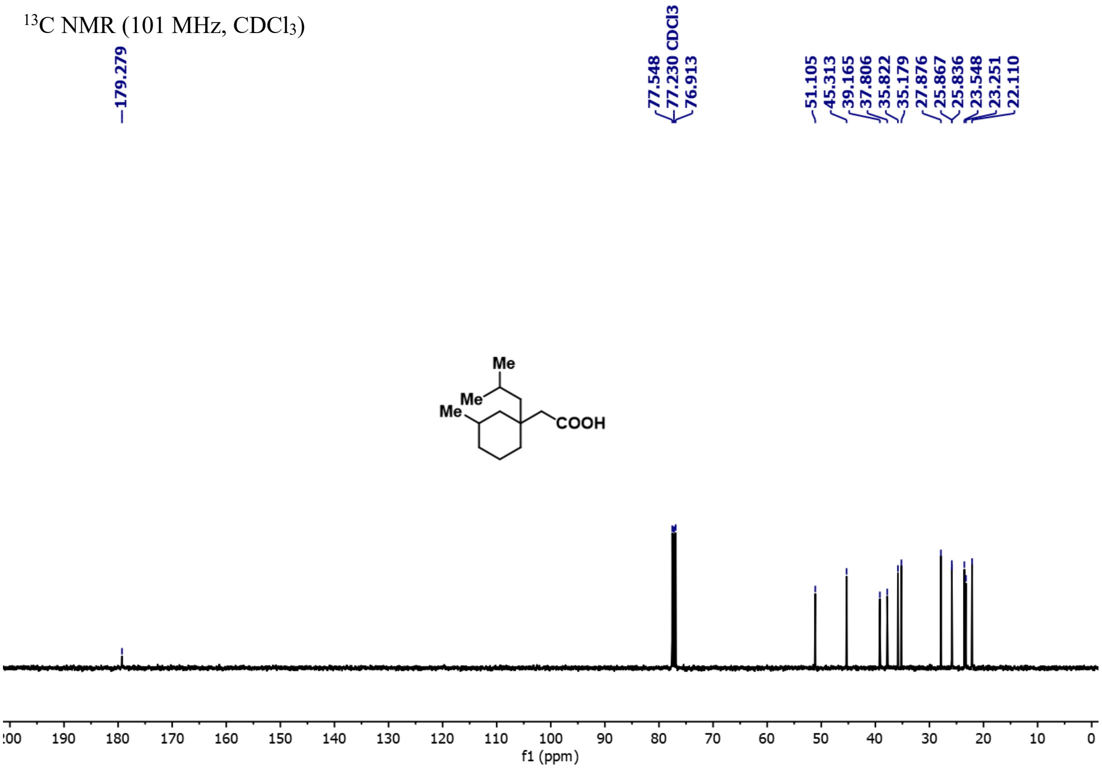

2-(1,3-Dimethylcyclopentadecyl)acetic acid (46)

^{1}H NMR (400 MHz, $CDCl_3$)

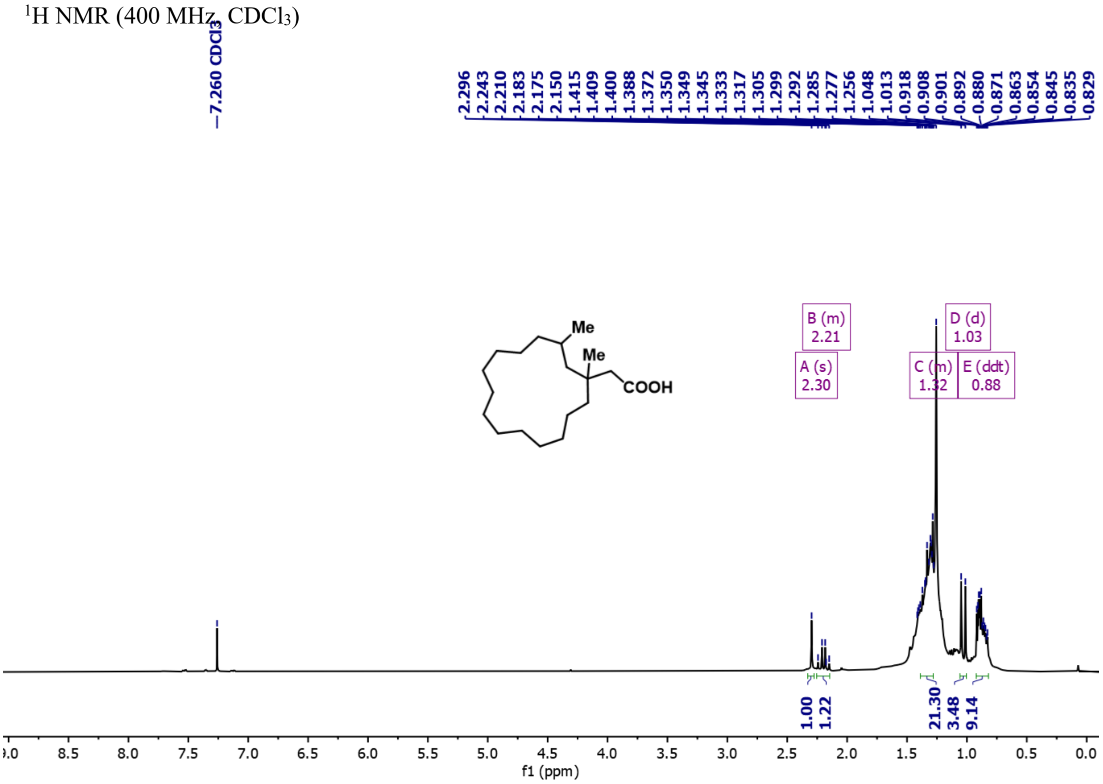

^{13}C NMR (101 MHz, $CDCl_3$)

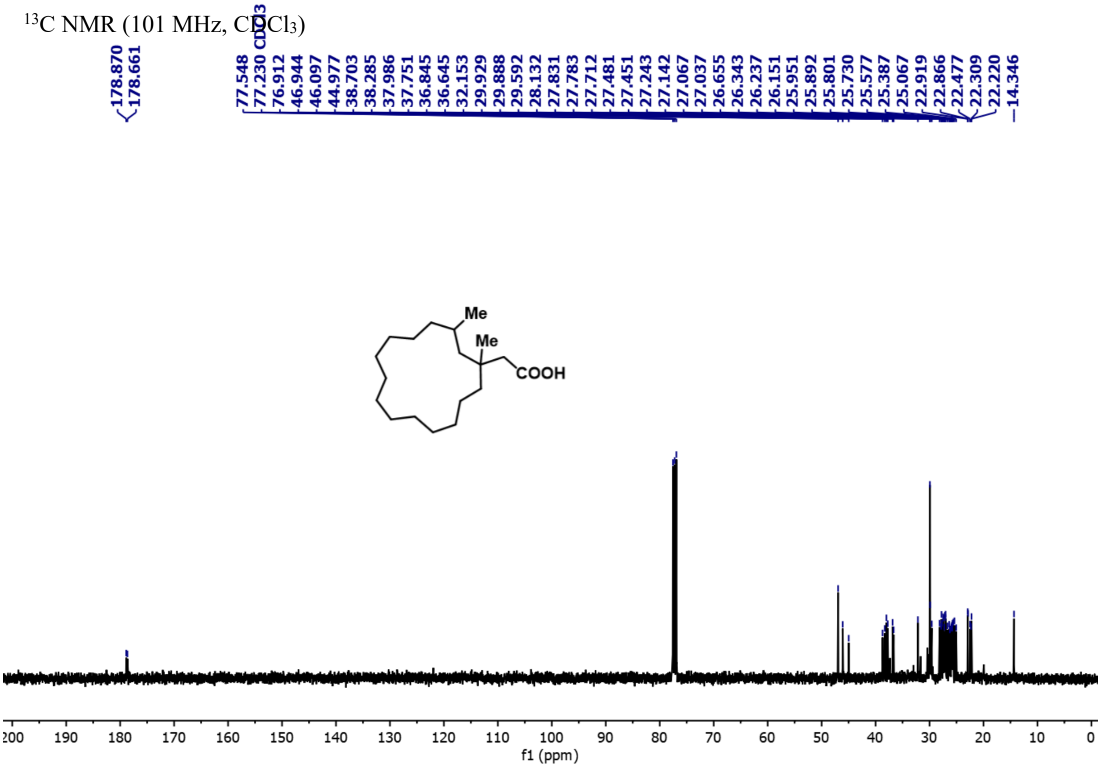

2-(1-Isopropylcyclopentyl)acetic acid (47)

^{1}H NMR (400 MHz, $CDCl_3$)

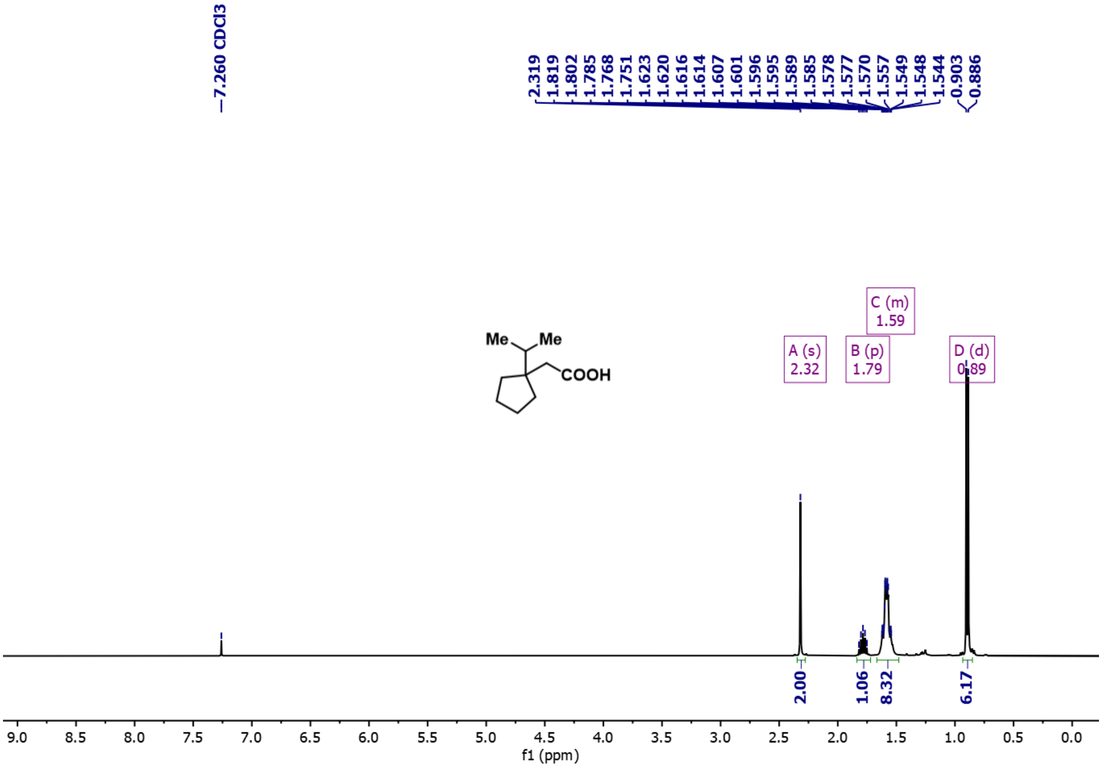

^{13}C NMR (101 MHz, $CDCl_3$)

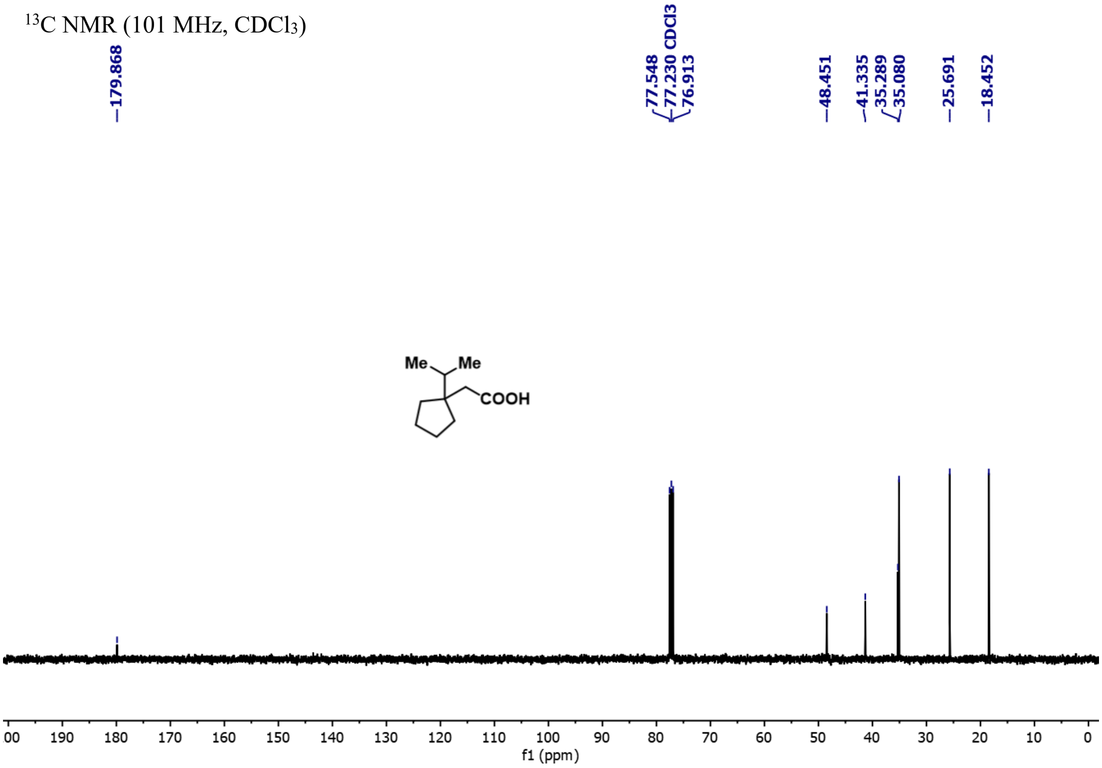

15. NMR Spectra of the Unsaturated Bicyclic Lactones:

Compound 2a

(3a*S*,7a*S*)-3a-Methyl-3a,4,5,7a-tetrahydrobenzofuran-2(3*H*)-one

^{1}H NMR (400 MHz, $CDCl_3$)

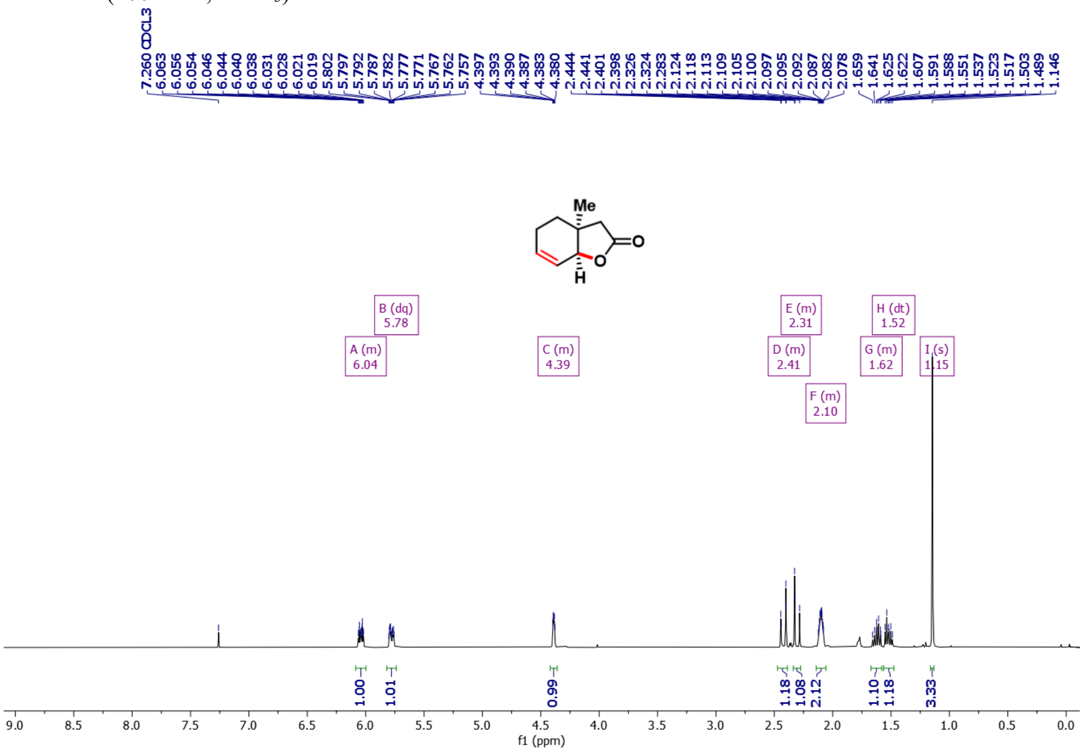

^{13}C NMR (101 MHz, $CDCl_3$)

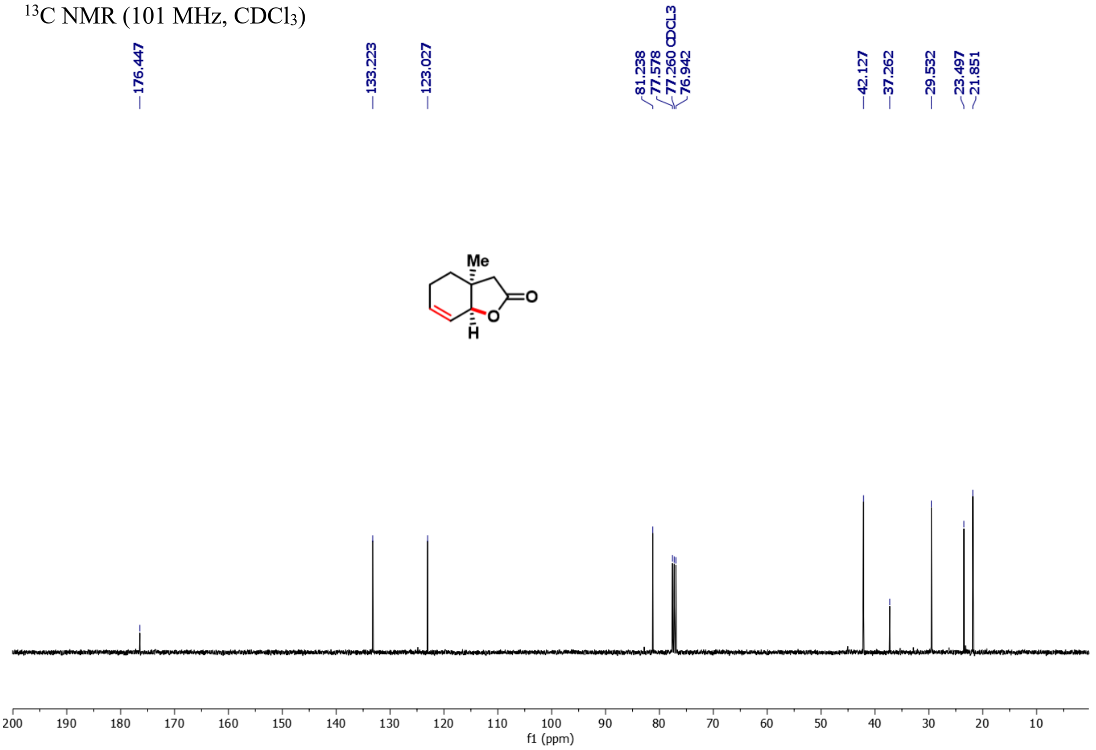

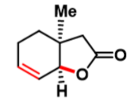
Me
O
O
H

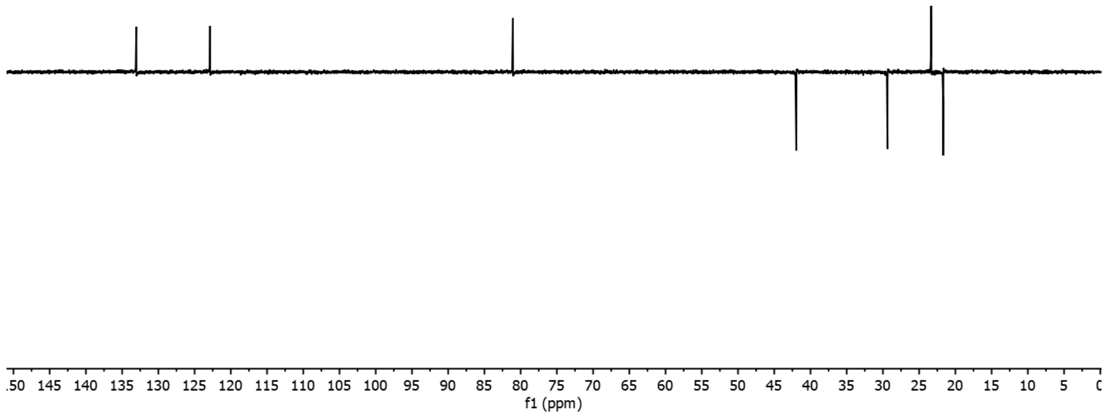
.50 145 140 135 130 125 120 115 110 105 100 95 90 85 80 75 70 65 60 55 50 45 40 35 30 25 20 15 10 5 0
f1 (ppm)

Compound 2b

(3a*S*,7a*S*)-3a-Ethyl-3a,4,5,7a-tetrahydrobenzofuran-2(3H)-one

^{1}H NMR (400 MHz, $CDCl_3$)

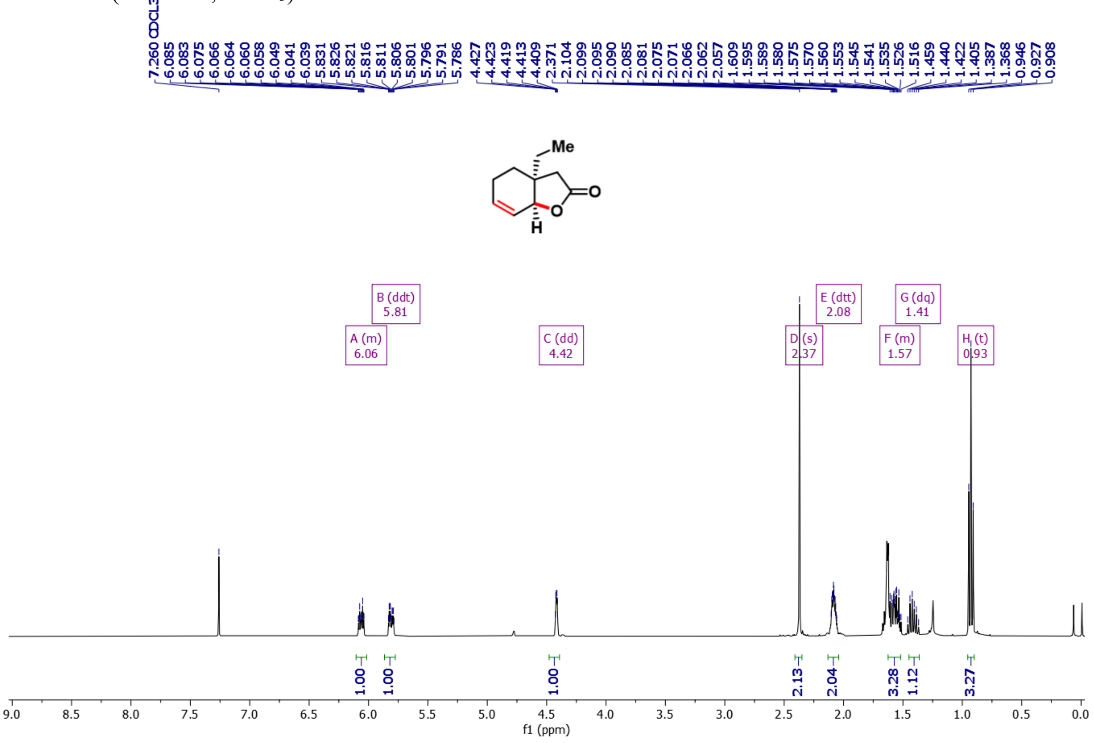

^{13}C NMR (101 MHz, $CDCl_3$)

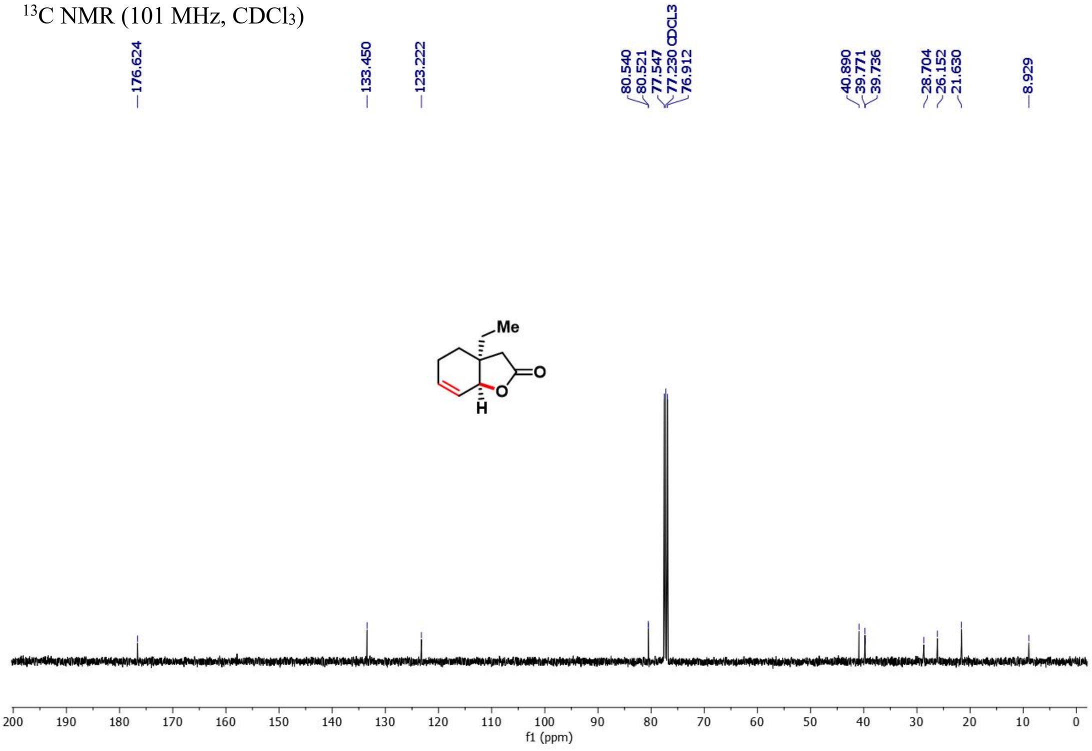

Compound 2c

(3a*S*,7a*S*)-3a-Isopropyl-3a,4,5,7a-tetrahydrobenzofuran-2(3H)-one

^{1}H NMR (400 MHz, $CDCl_3$)

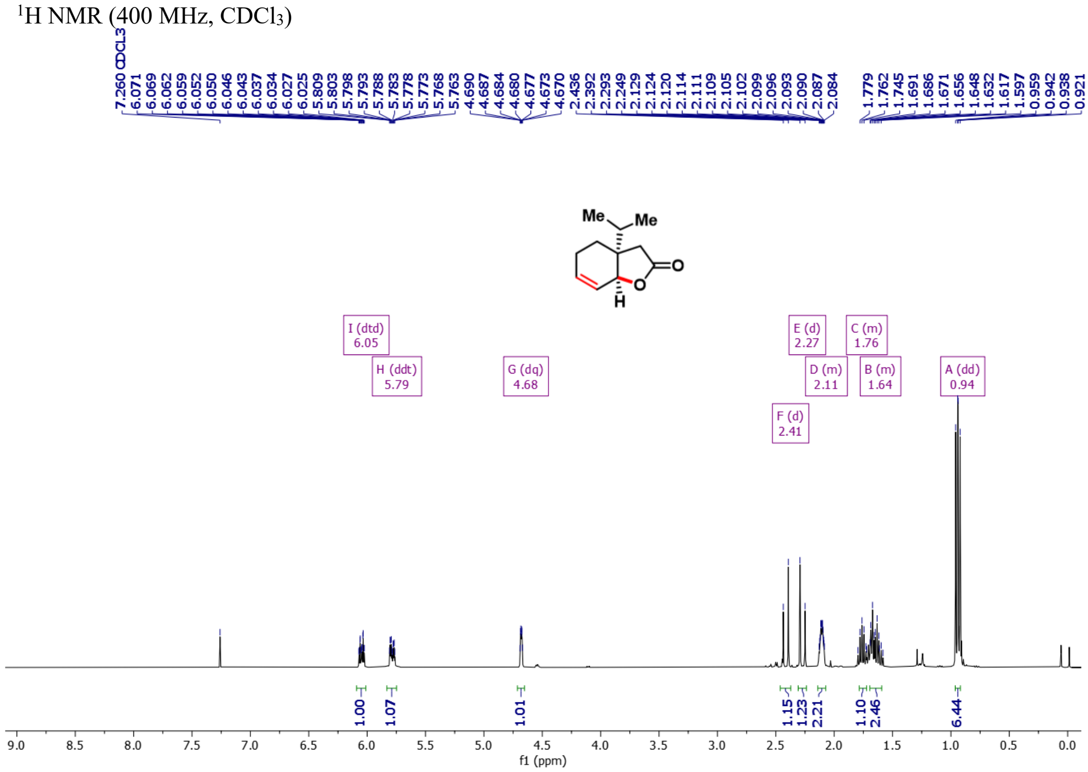

^{13}C NMR (101 MHz, $CDCl_3$)

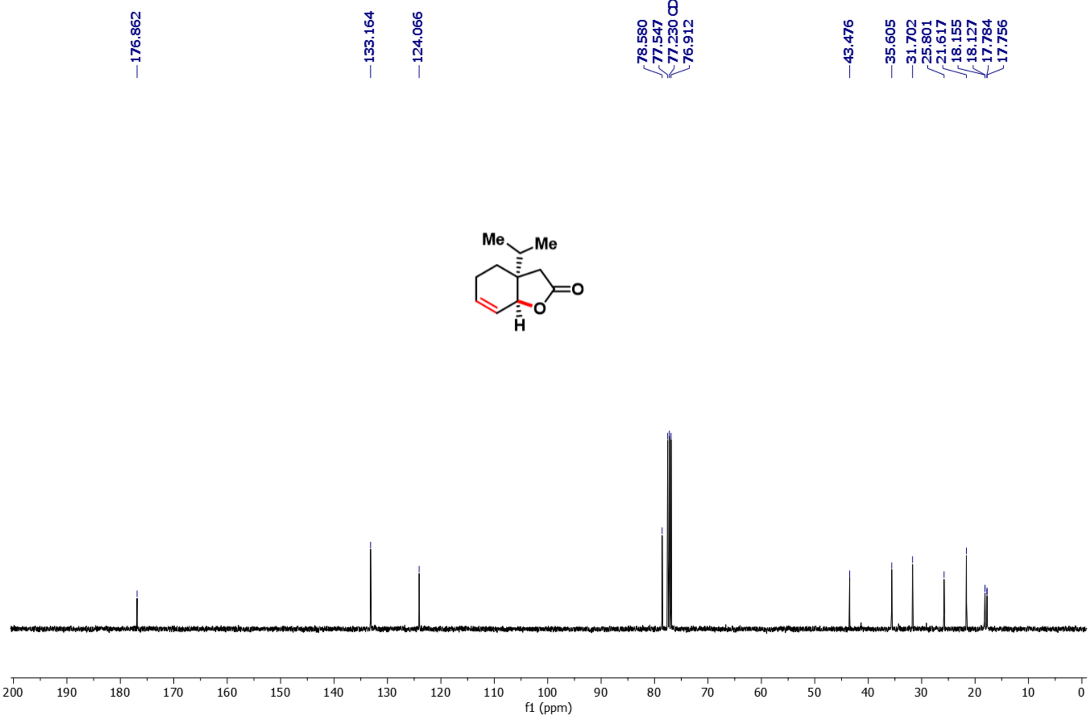

Compound 2d

(3a*S*,7a*S*)-3a-Propyl-3a,4,5,7a-tetrahydrobenzofuran-2(3H)-one

^{1}H NMR (400 MHz, $CDCl_3$)

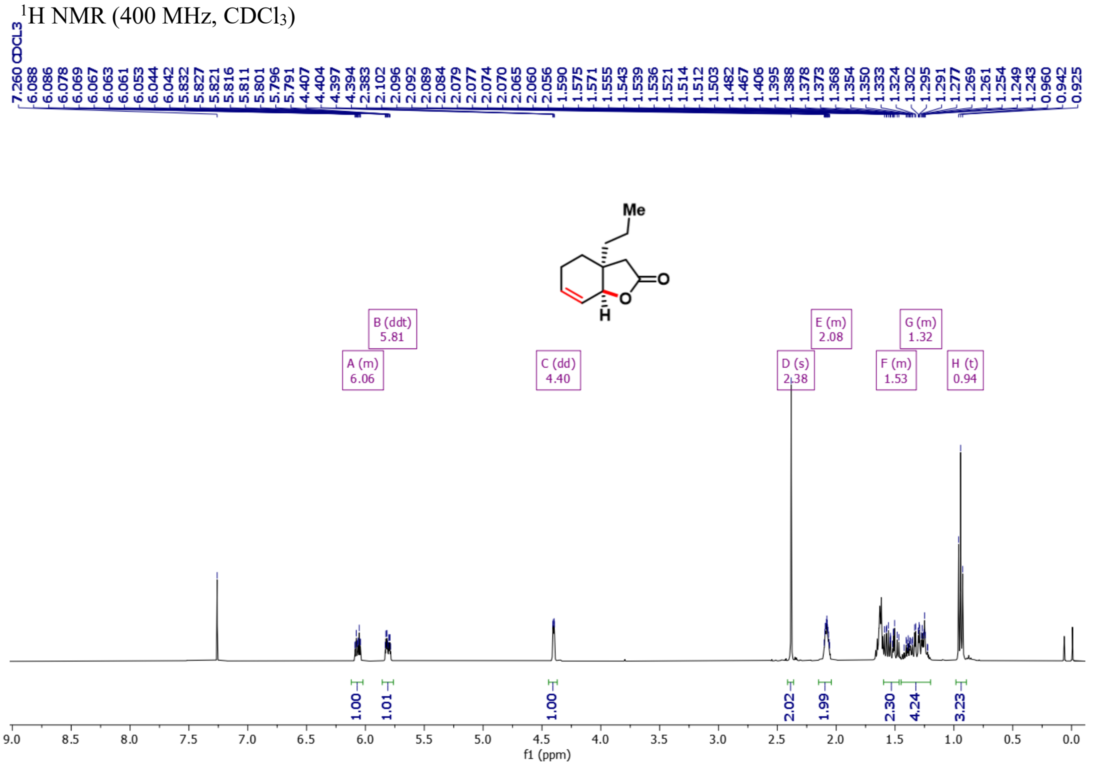

^{13}C NMR (126 MHz, $CDCl_3$)

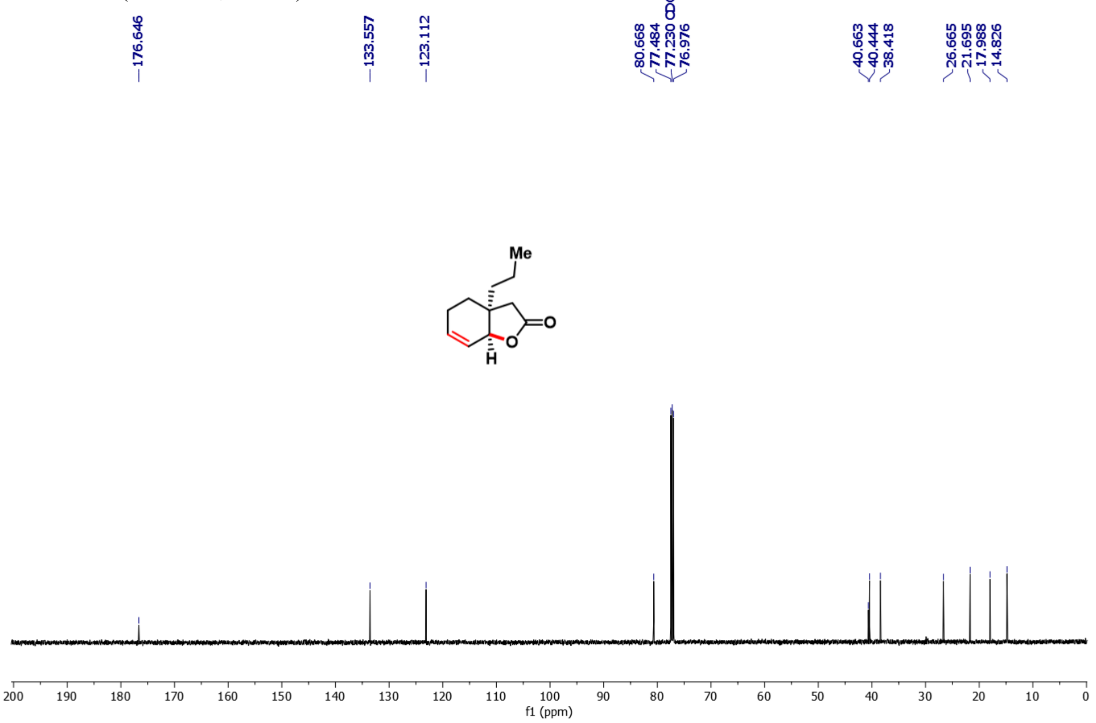

Compound 2e

(3a*S*,7a*S*)-3a-Butyl-3a,4,5,7a-tetrahydrobenzofuran-2(3H)-one

^{1}H NMR (400 MHz, $CDCl_3$)

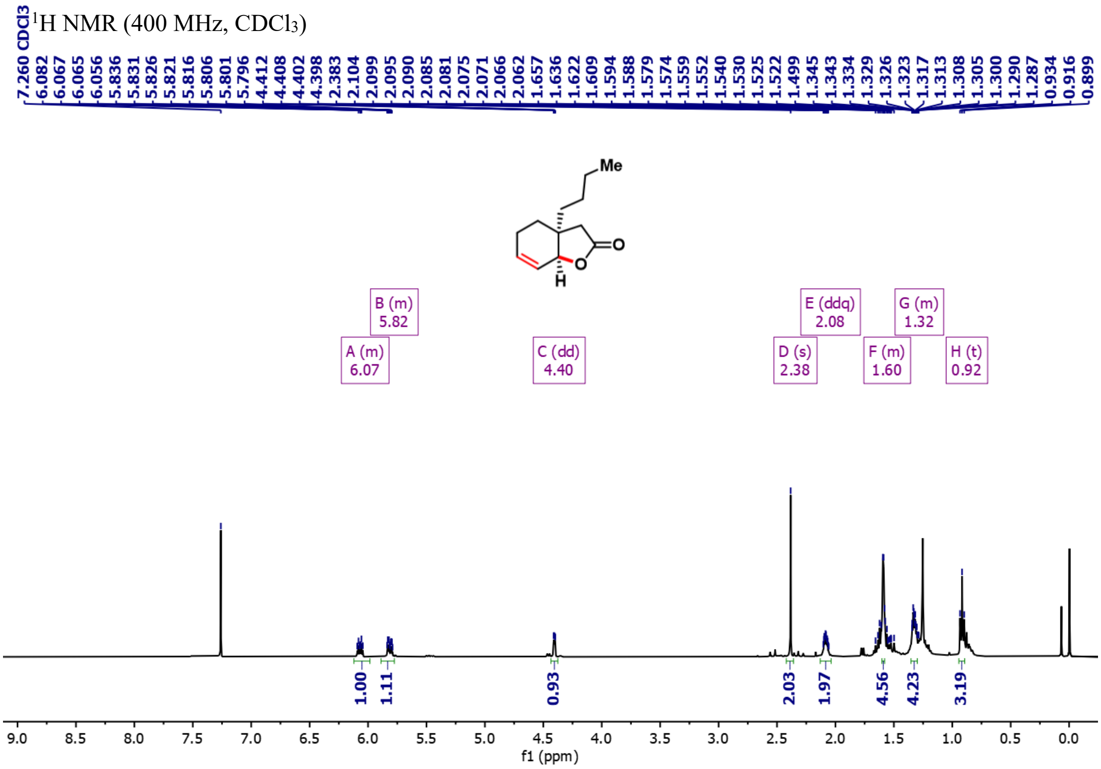

^{13}C NMR (101 MHz, $CDCl_3$)

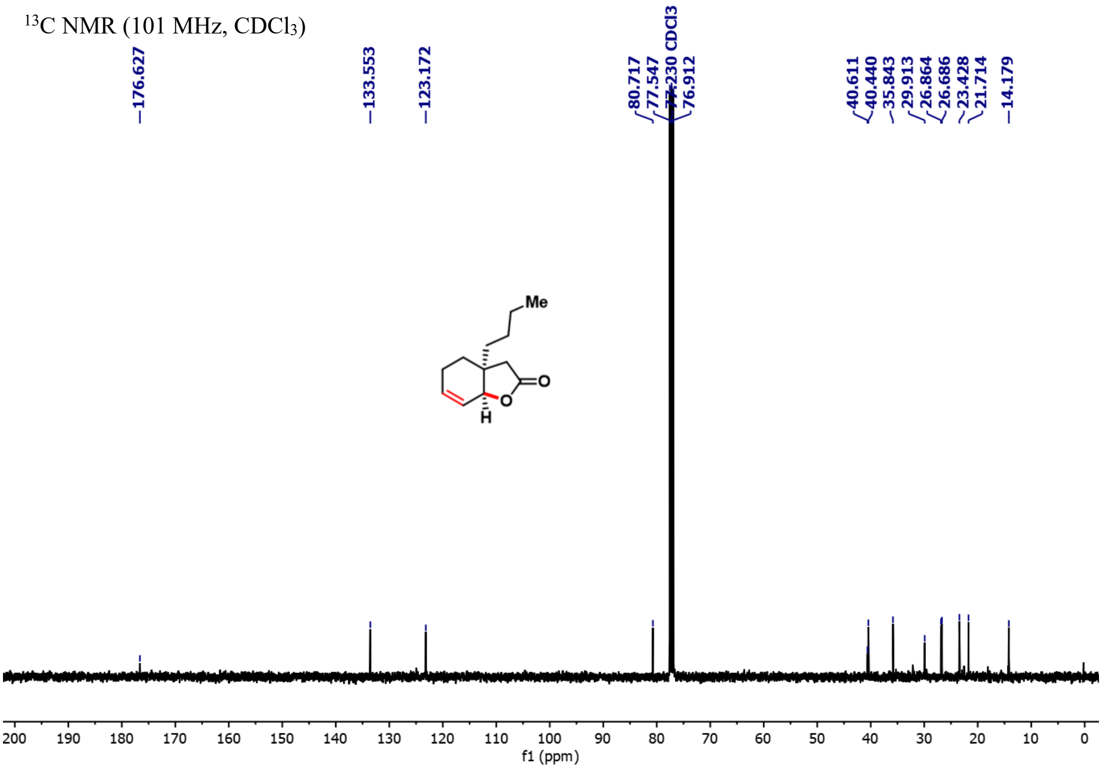

Compound 2f

(3a*R*,7a*S*)-3a-Isobutyl-3a,4,5,7a-tetrahydrobenzofuran-2(3H)-one

^{1}H NMR (400 MHz, $CDCl_3$)

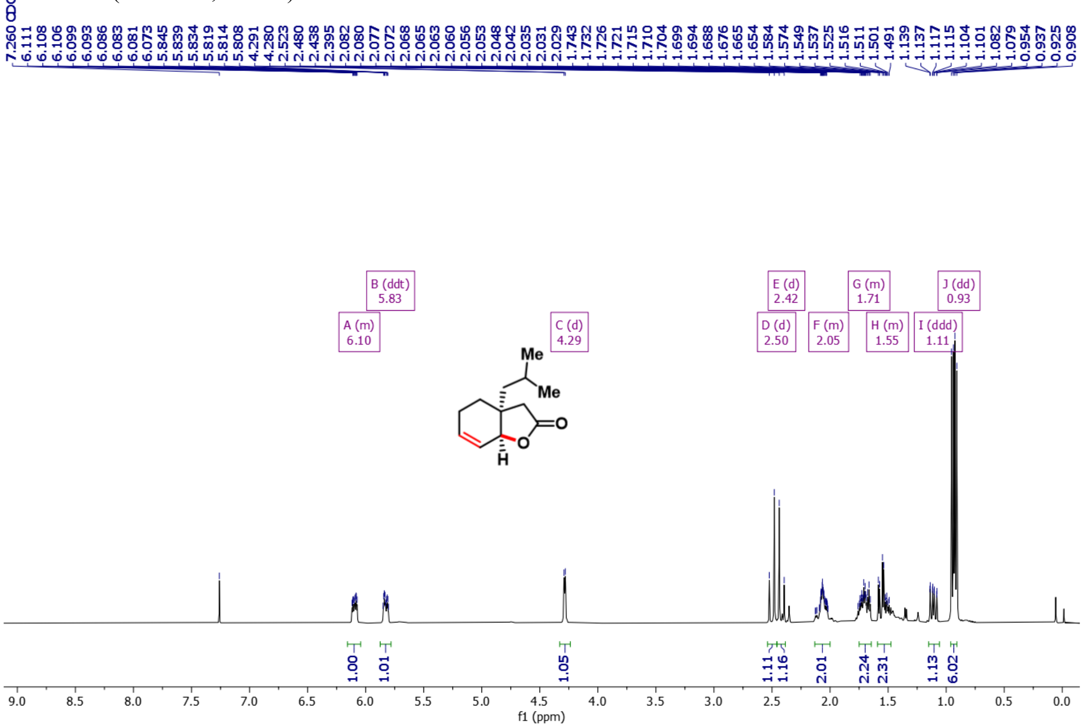

^{13}C NMR (101 MHz, $CDCl_3$)

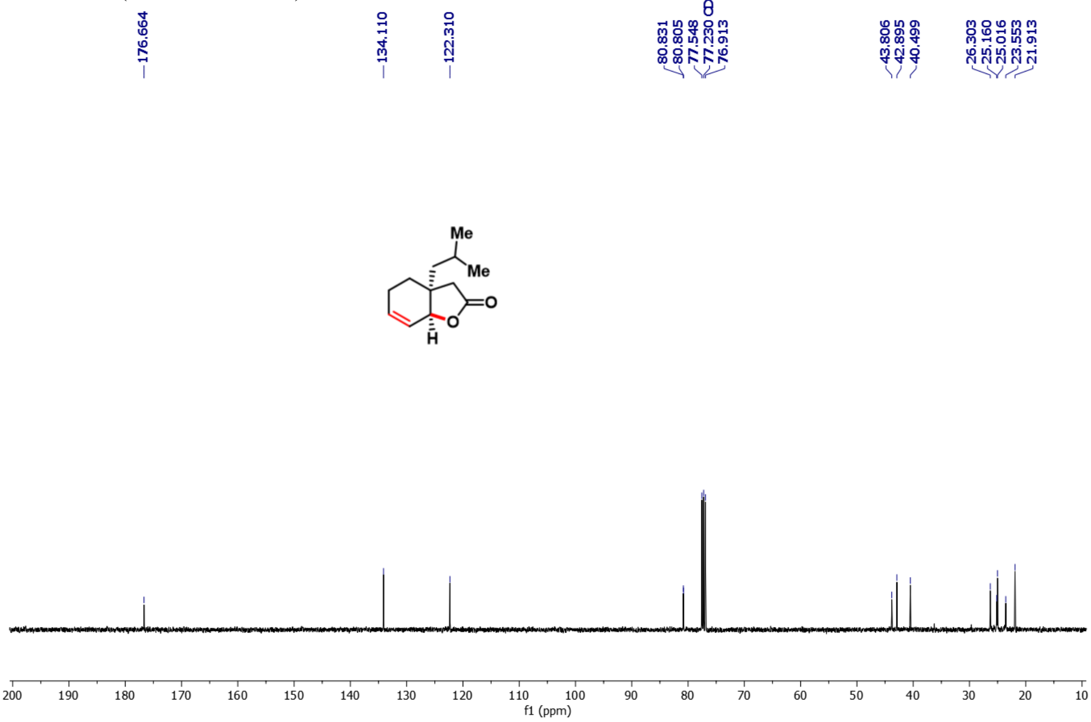

Compound 2g

(3a*S*,7a*S*)-3a-Octyl-3a,4,5,7a-tetrahydrobenzofuran-2(3H)-one

^{1}H NMR (500 MHz, $CDCl_3$)

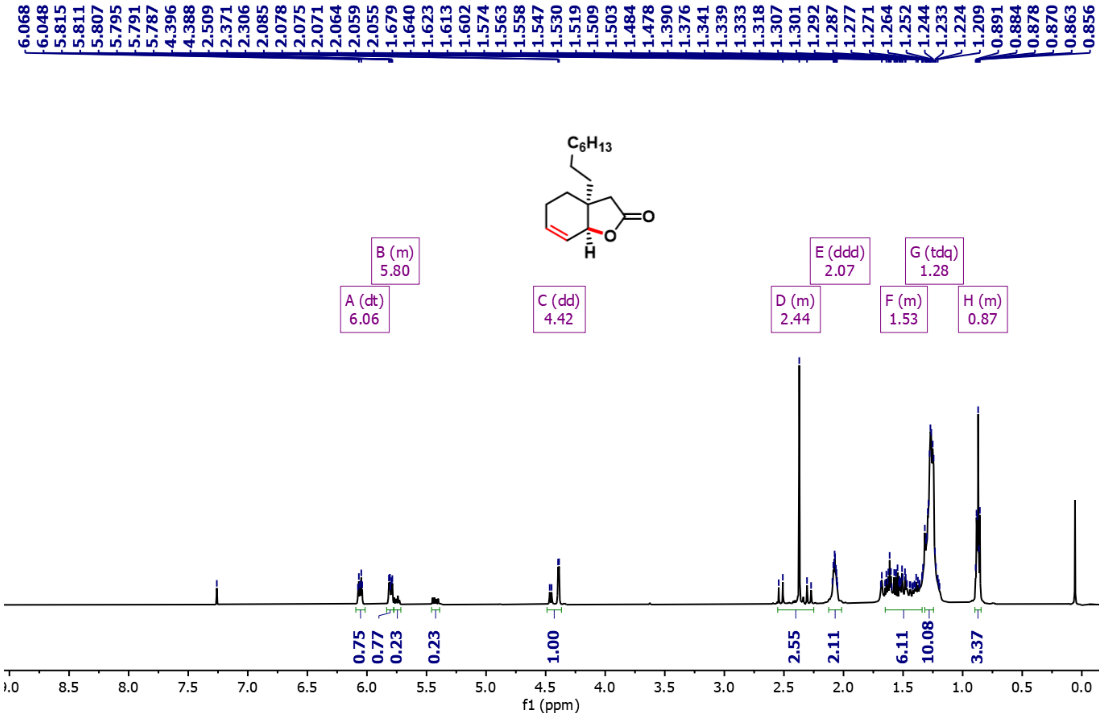

^{13}C NMR (126 MHz, $CDCl_3$)

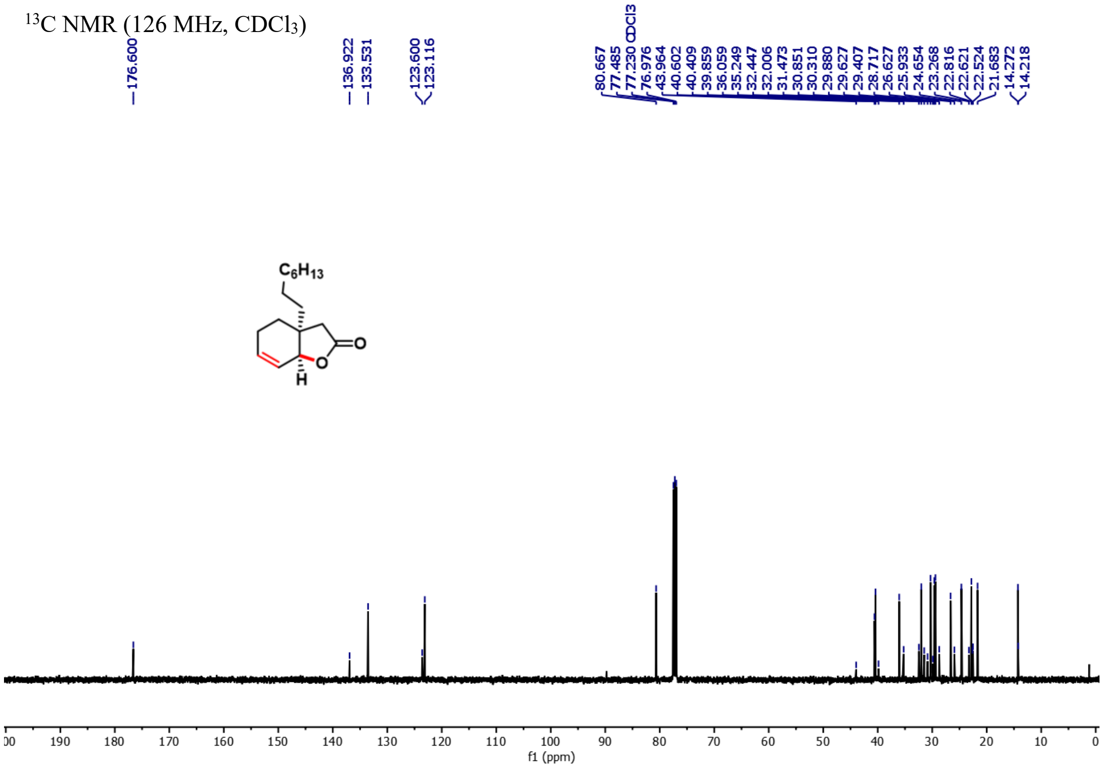

Compound 2h

(3a*S*,7a*S*)-3-Methyl-3a-propyl-3a,4,5,7a-tetrahydrobenzofuran-2(3H)-one

^{1}H NMR (400 MHz, $CDCl_3$)

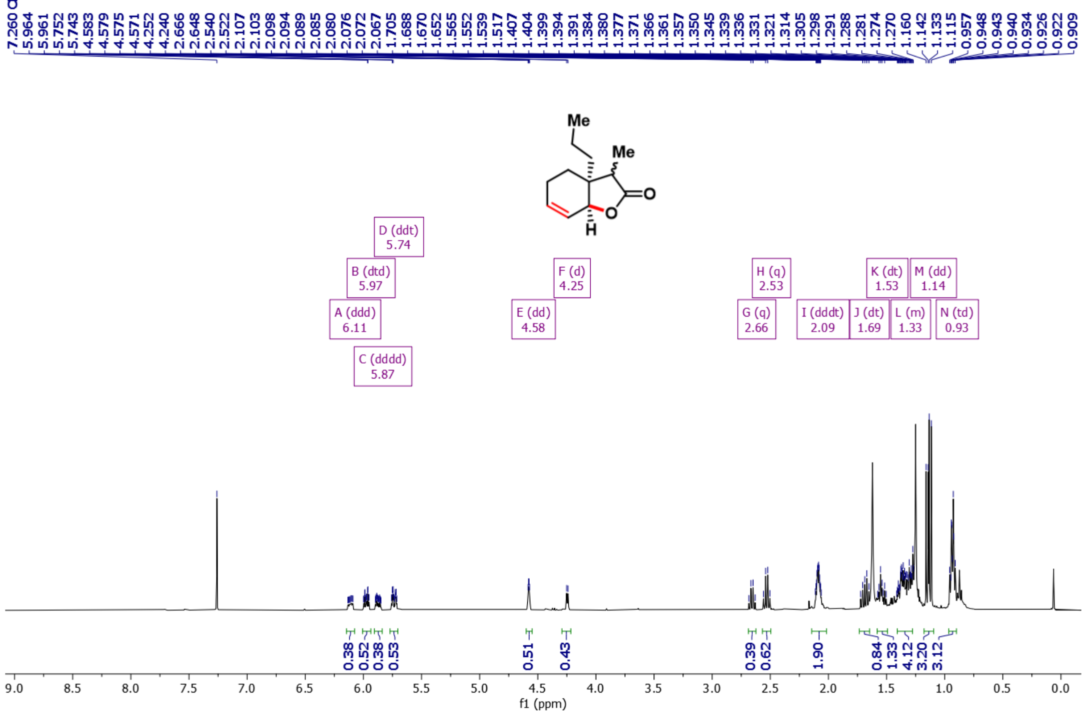

^{13}C NMR (101 MHz, $CDCl_3$)

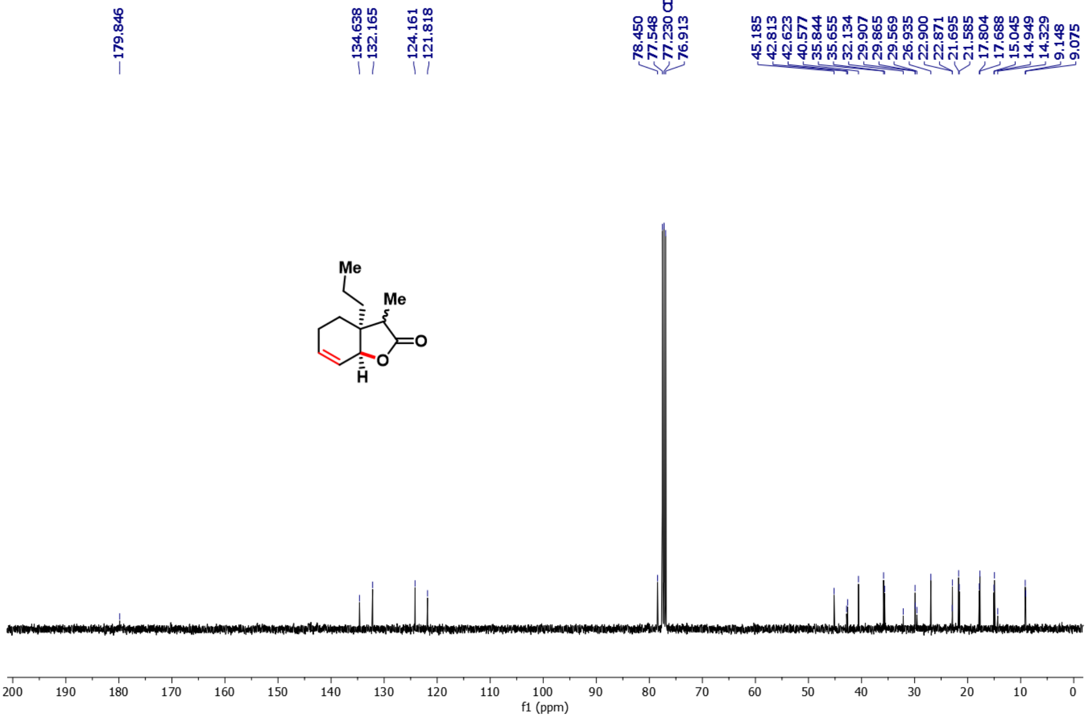

Compound 2i

(3a*S*,7a*S*)-3a-Cyclopentyl-3a,4,5,7a-tetrahydrobenzofuran-2(3H)-one

^{1}H NMR (500 MHz, $CDCl_3$)

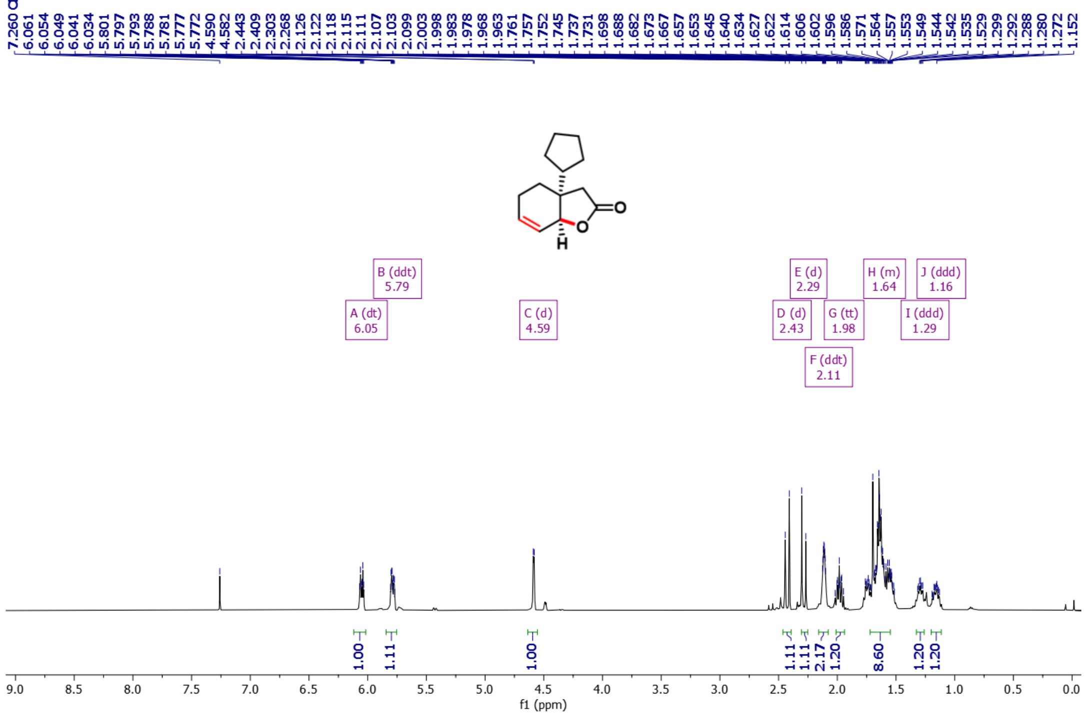

^{13}C NMR (101 MHz, $CDCl_3$)

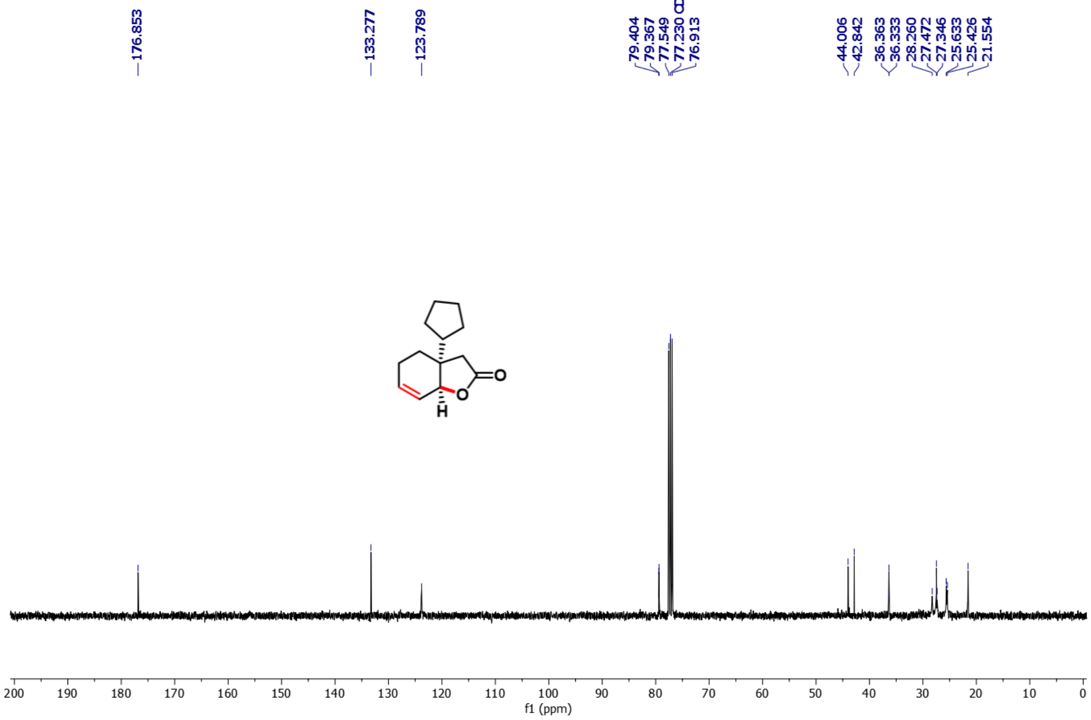

Compound 2j

(3a*S*,7a*S*)-3a-Cyclohexyl-3a,4,5,7a-tetrahydrobenzofuran-2(3H)-one

^{1}H NMR (400 MHz, $CDCl_3$)

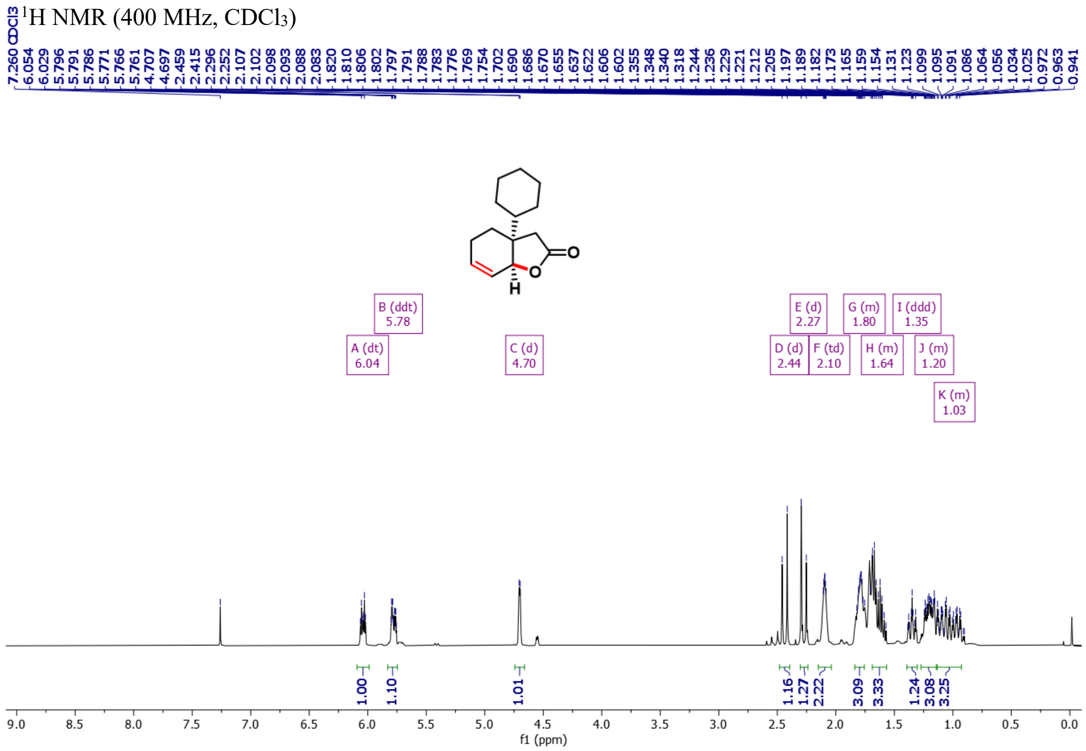

^{13}C NMR (101 MHz, $CDCl_3$)

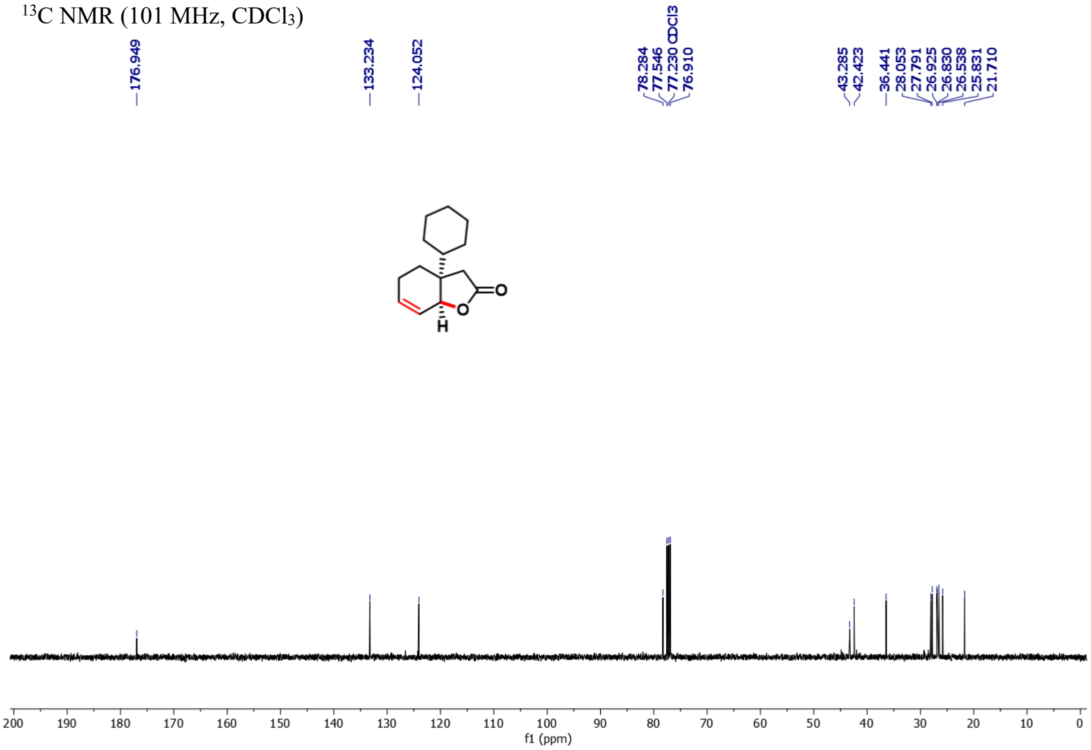

Compound 2k

(3a*S*,7a*S*)-3a-(*p*-Tolyl)-3a,4,5,7a-tetrahydrobenzofuran-2(3H)-one

^{1}H NMR (400 MHz, $CDCl_3$)

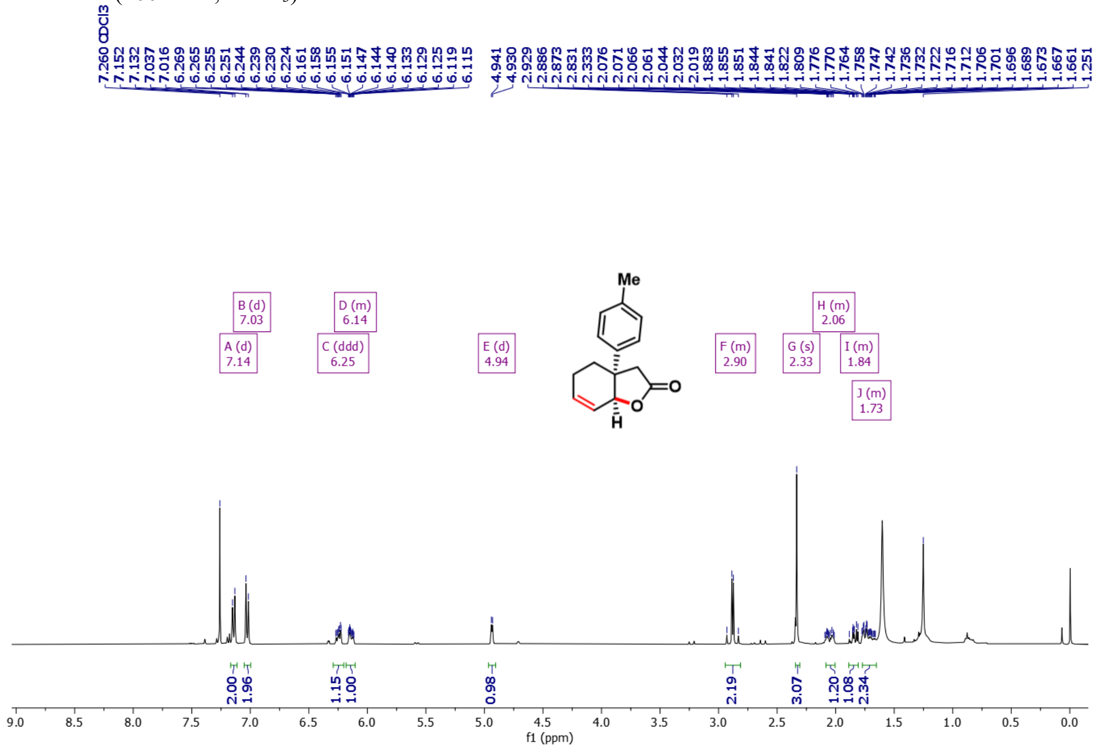

^{13}C NMR (101 MHz, $CDCl_3$)

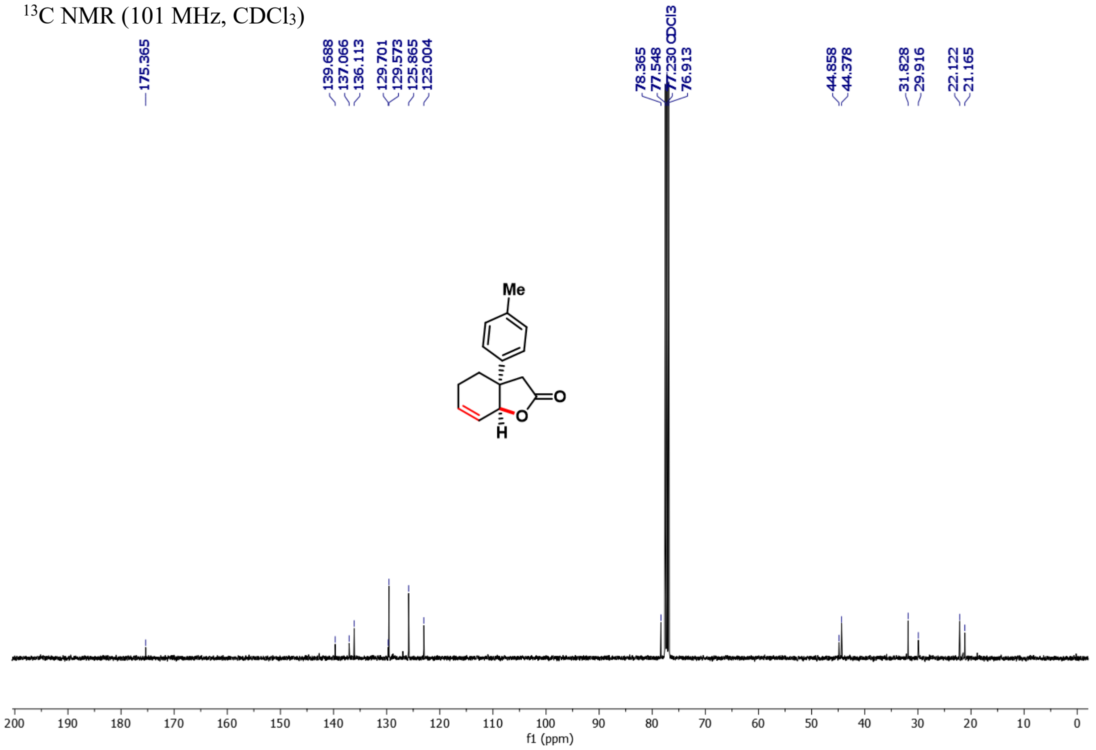

Compound 2l

(3a*S*,7a*S*)-3a-(4-Methoxyphenyl)-3a,4,5,7a-tetrahydrobenzofuran-2(3H)-one

^{1}H NMR (400 MHz, CDCl$_3$)

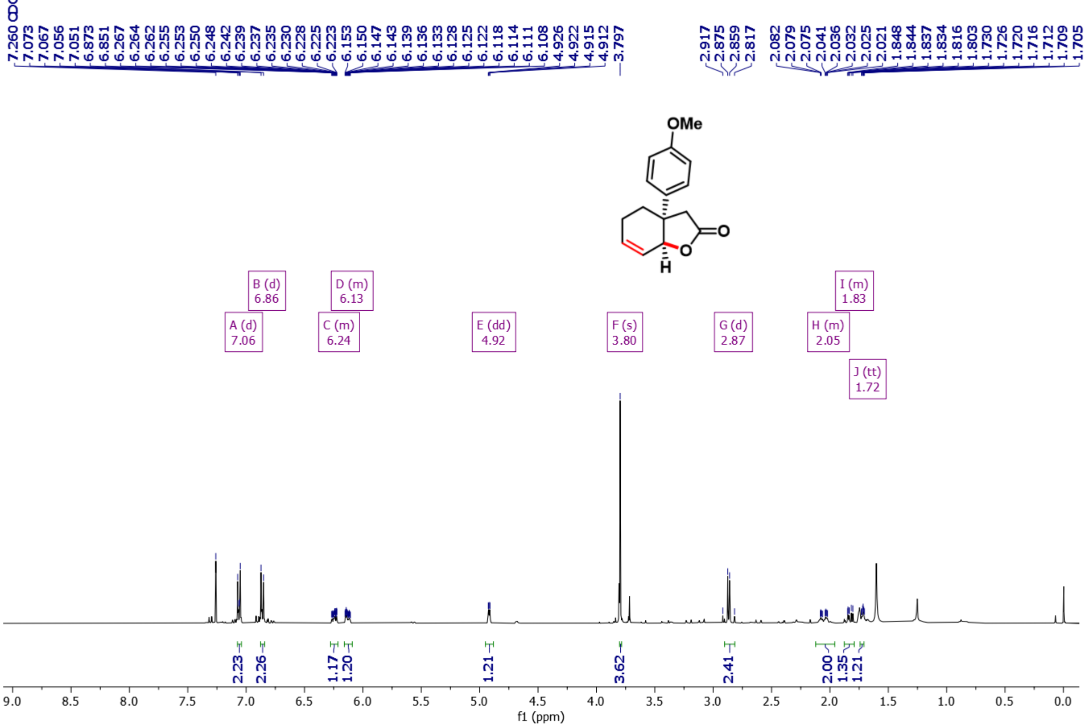

^{13}C NMR (101 MHz, CDCl$_3$)

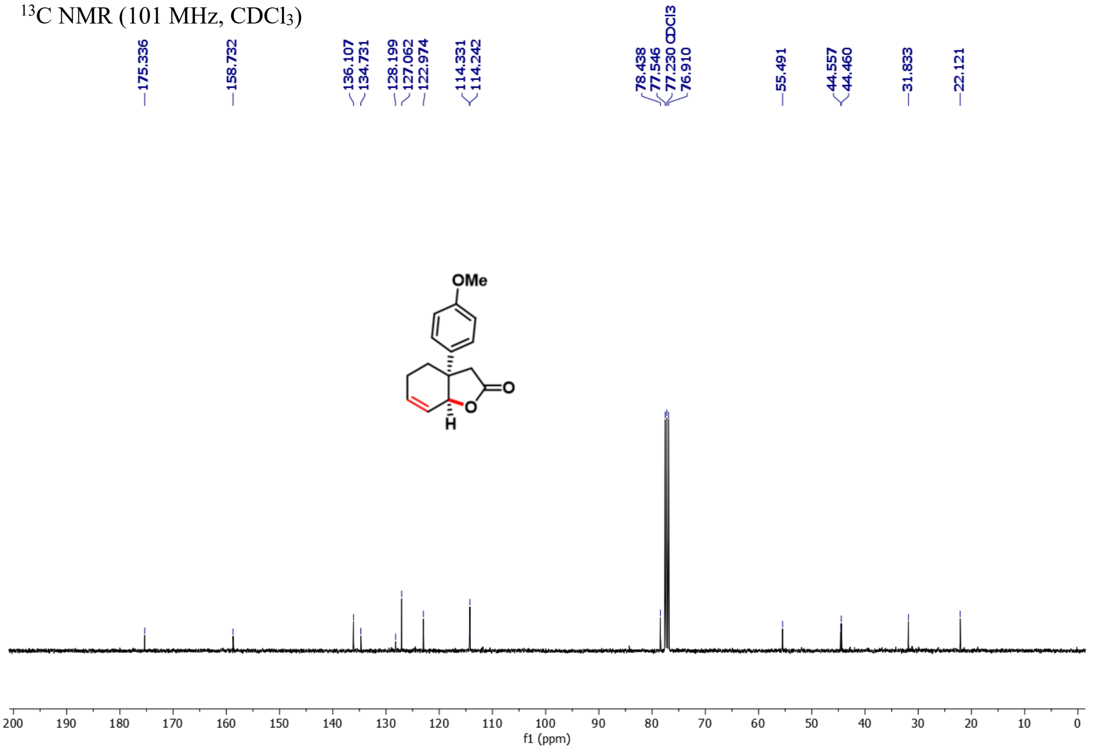

Compound 2m

(3a*S*,7a*S*)-3a-(3-Methoxyphenyl)-3a,4,5,7a-tetrahydrobenzofuran-2(3H)-one

^{1}H NMR (400 MHz, $CDCl_3$)

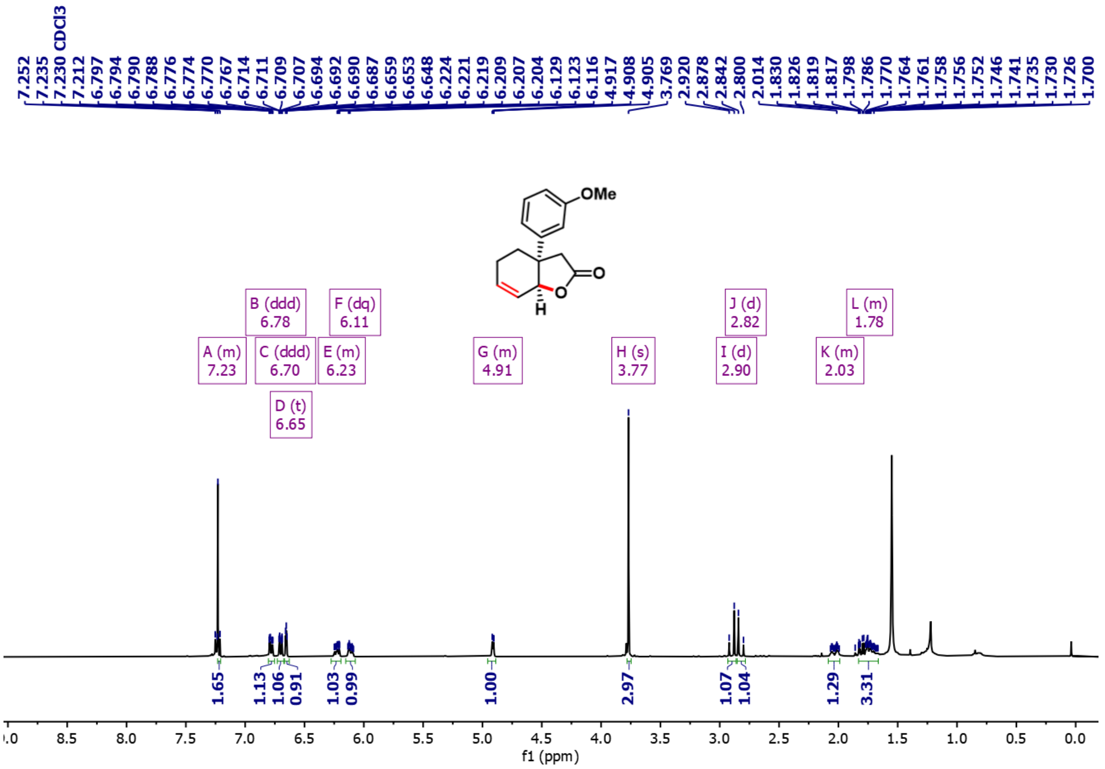

^{13}C NMR (101 MHz, $CDCl_3$)

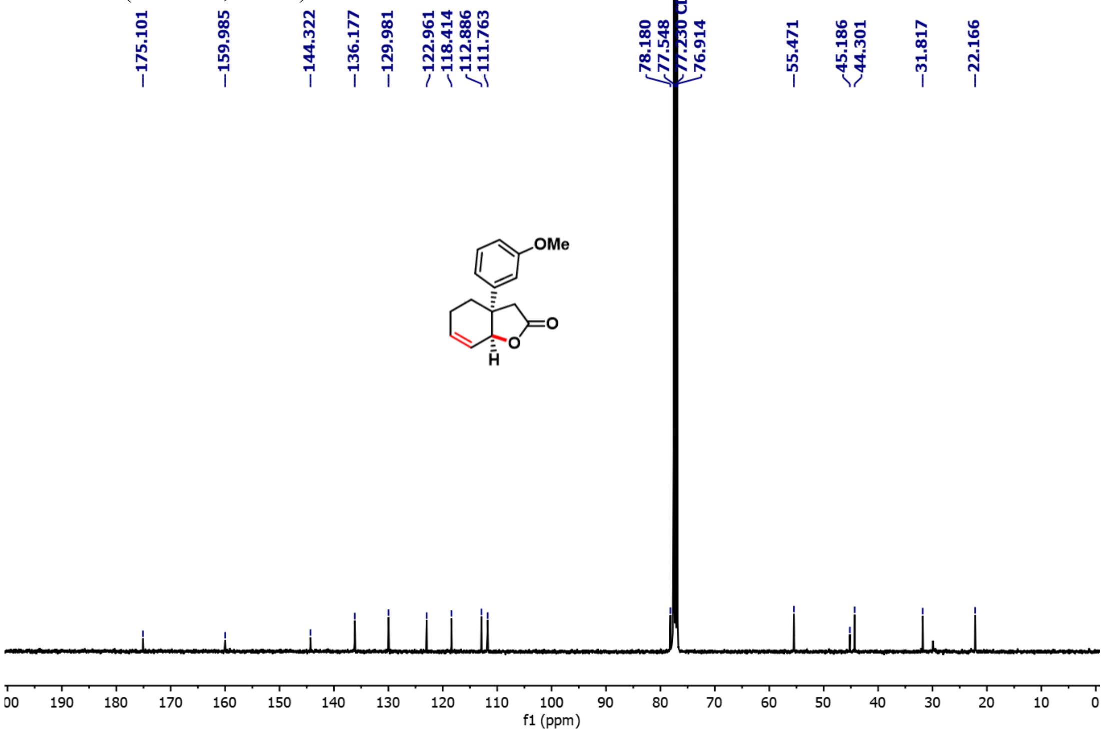

Compound 2n

(3a*S*,7a*S*)-3a-(3,4-Dimethoxyphenyl)-3a,4,5,7a-tetrahydrobenzofuran-2(3H)-one

^{1}H NMR (500 MHz, CDCl$_3$)

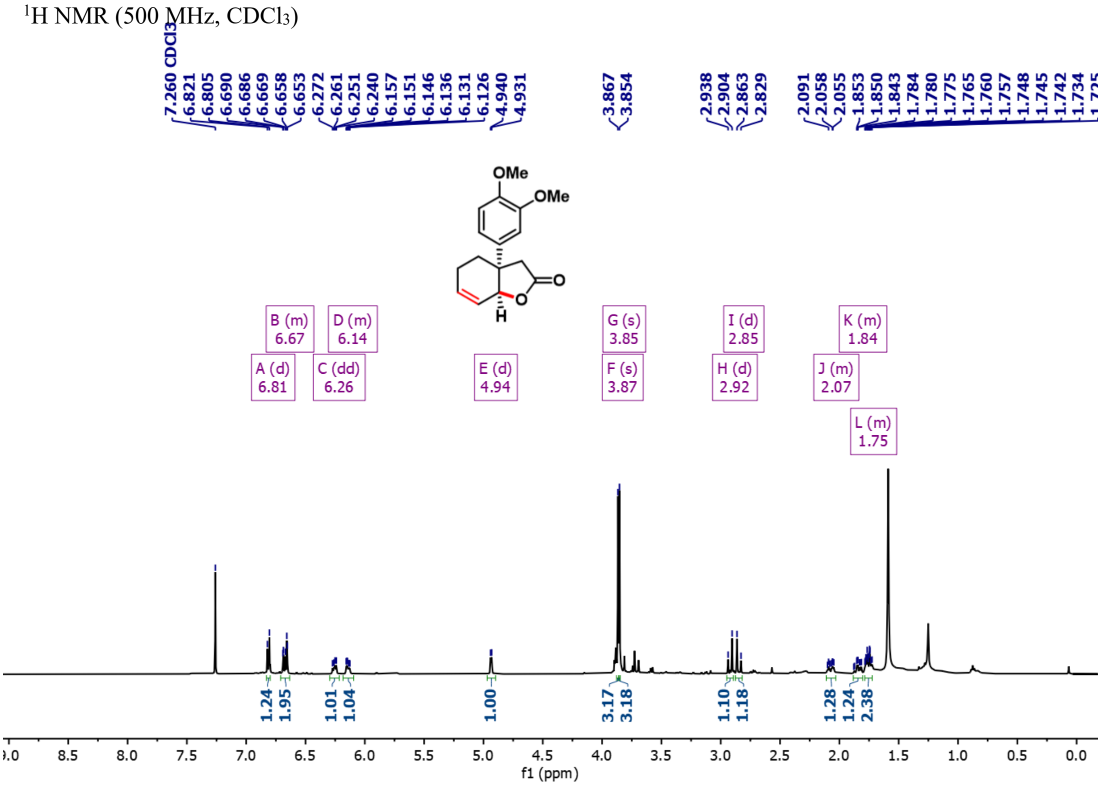

^{13}C NMR (126 MHz, CDCl$_3$)

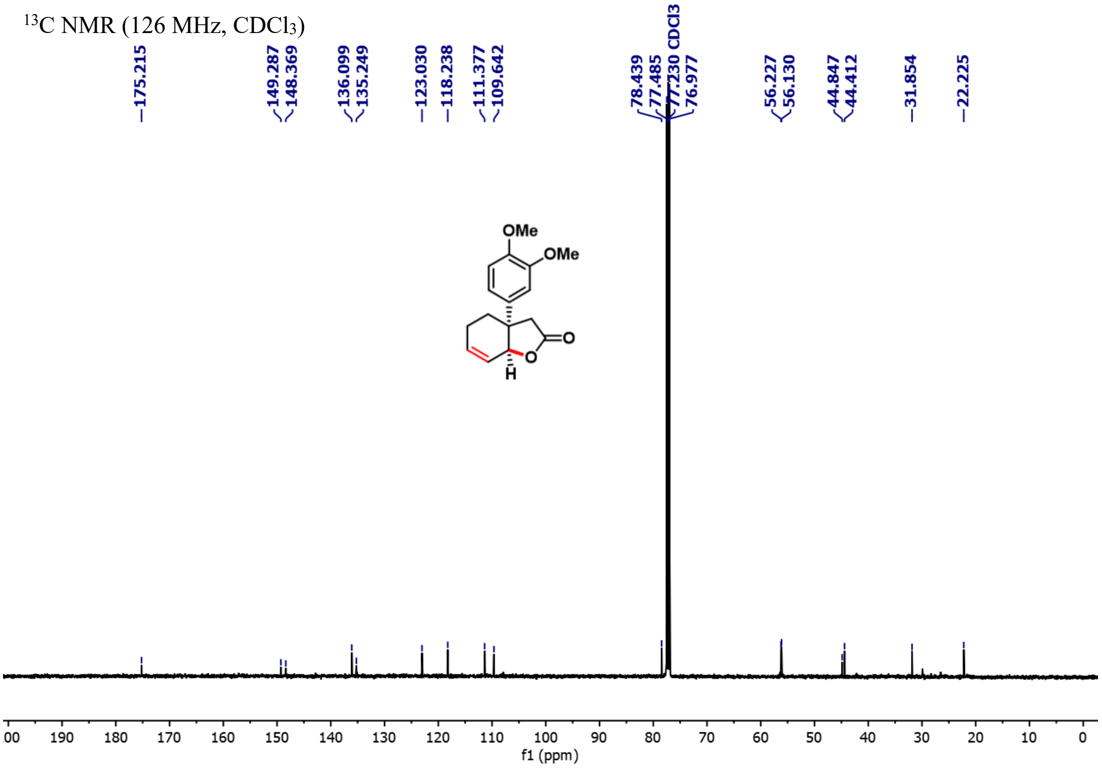

Compound 2o

(3a*R*,7a*S*)-3a,4-Dimethyl-3a,4,5,7a-tetrahydrobenzofuran-2(3H)-one

^{1}H NMR (400 MHz, $CDCl_3$)

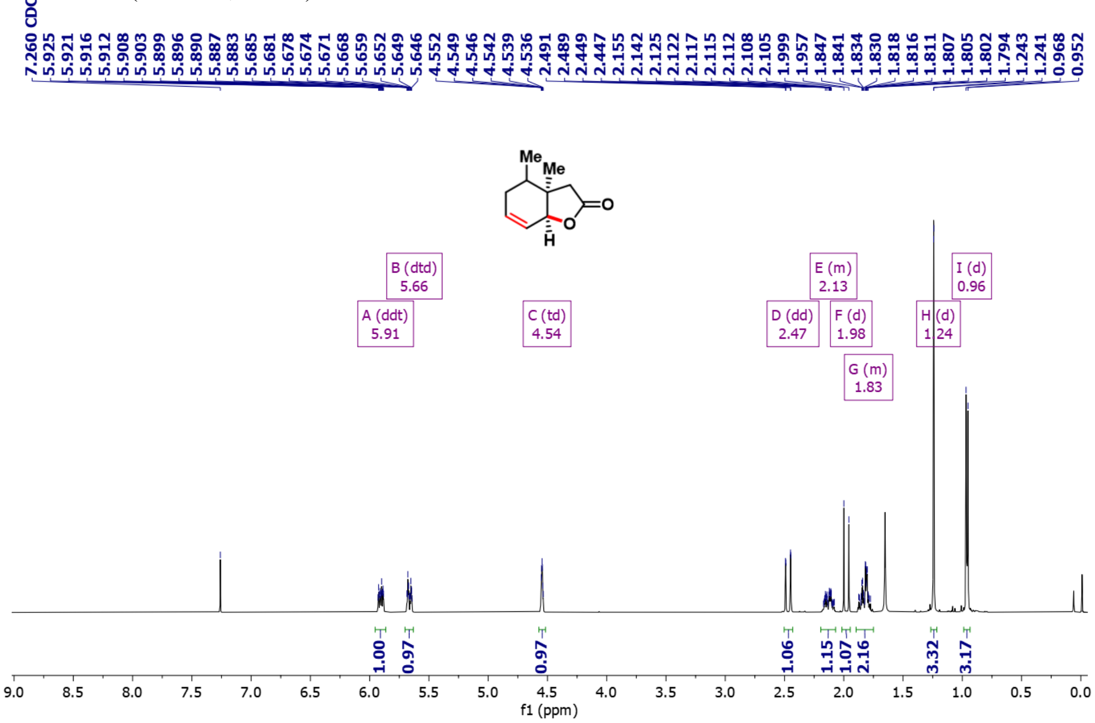

^{13}C NMR (101 MHz, $CDCl_3$)

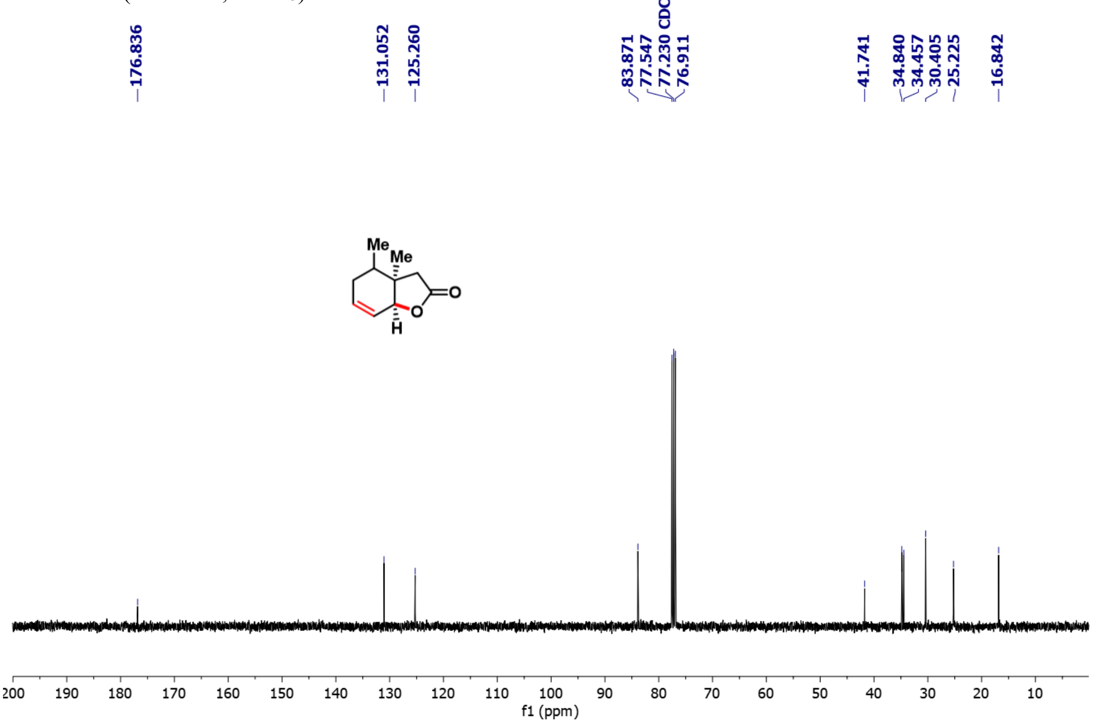

NOE Experiment:

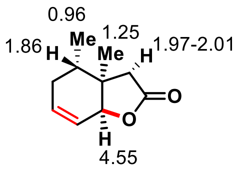

Me (1.25) $\xrightarrow{\text{Strong NOE}}$ Me (0.96)

Me (1.25) $\xrightarrow{\text{Strong NOE}}$ H (2.01)

Me (1.25) $\xrightarrow{\text{Strong NOE}}$ H (4.55)

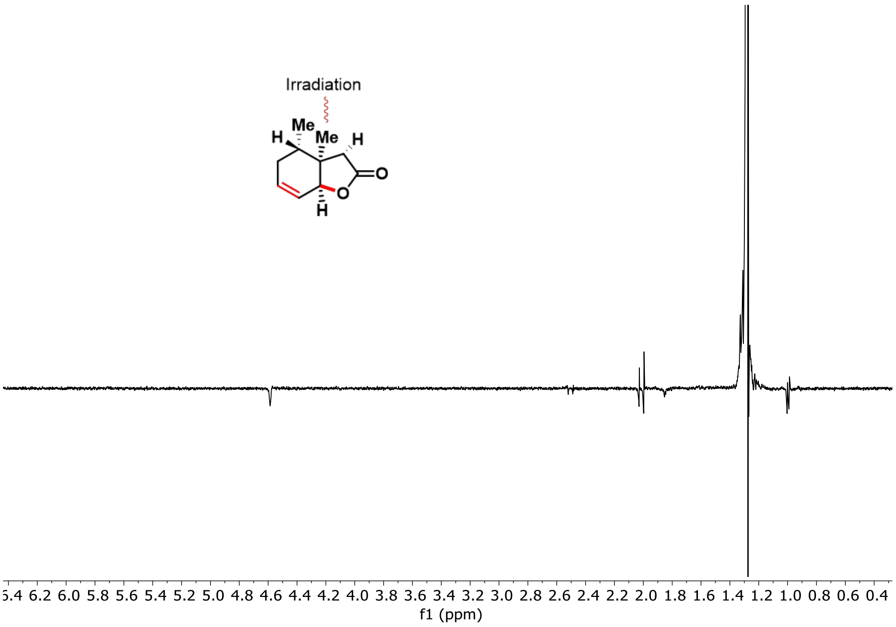

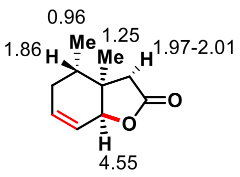

Me (0.96) —Strong NOE→ Me (1.25)

Me (0.96) —NOE→ H (1.86)

Me (0.96) —NOE→ H (2.01)

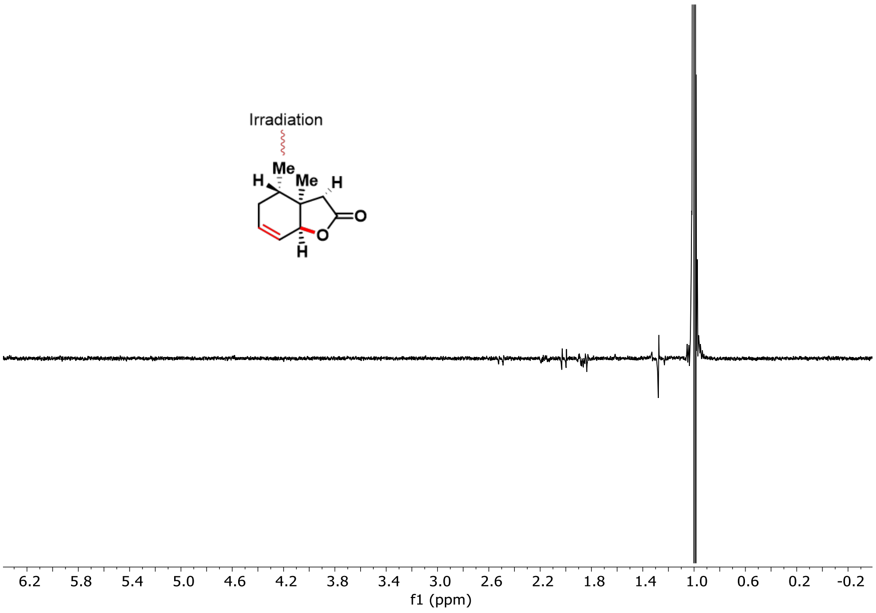

Compound 2p

(3a*S*,7a*S*)-3a,5-Dimethyl-3a,4,5,7a-tetrahydrobenzofuran-2(3H)-one

^{1}H NMR (400 MHz, $CDCl_3$)

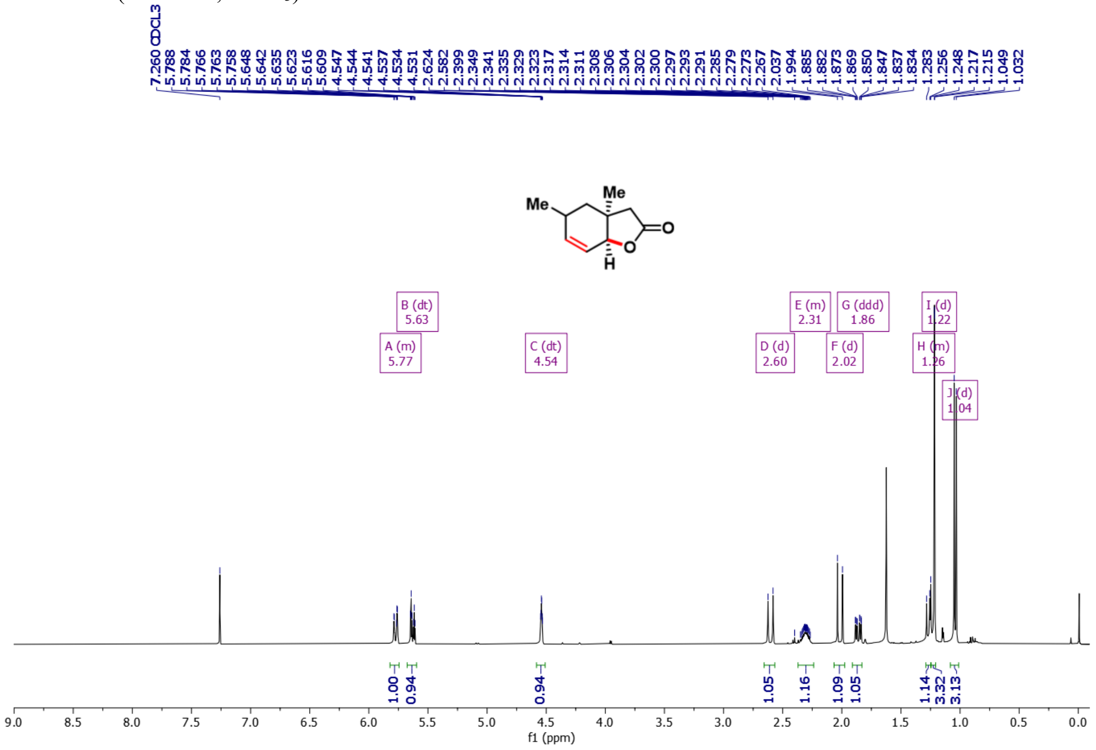

^{13}C NMR (126 MHz, $CDCl_3$)

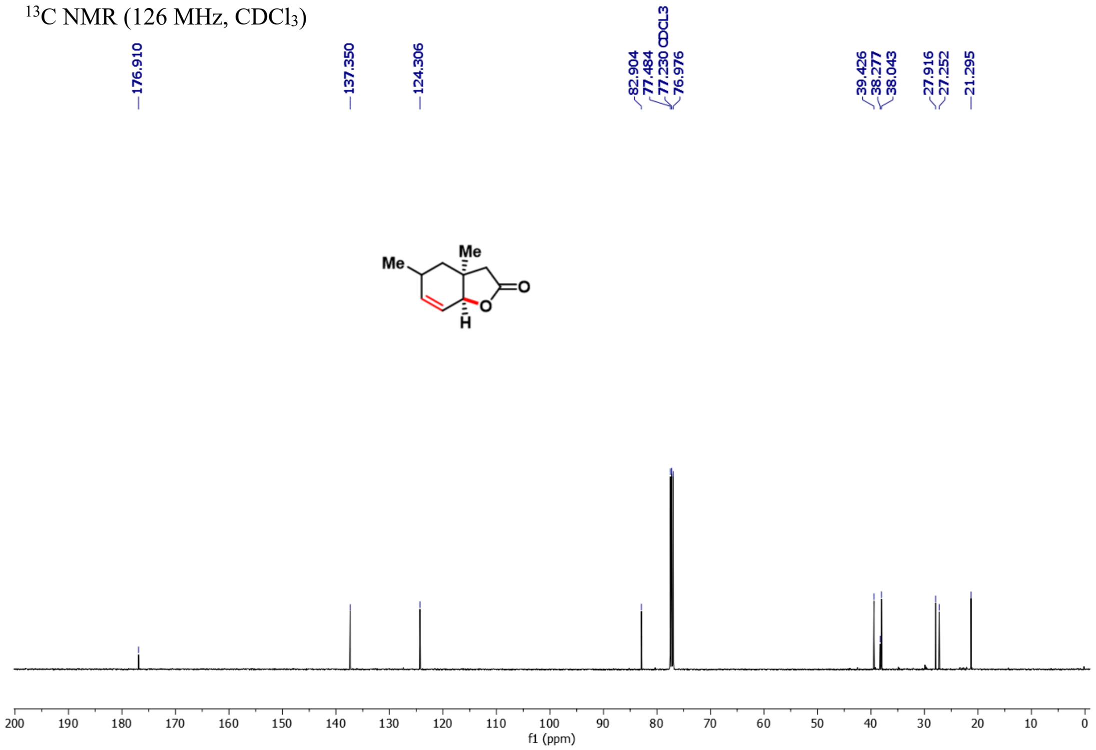

Compound 2q

(3a*S*,7a*S*)-3a-Ethyl-5-methyl-3a,4,5,7a-tetrahydrobenzofuran-2(3H)-one

^{1}H NMR (400 MHz, $CDCl_3$)

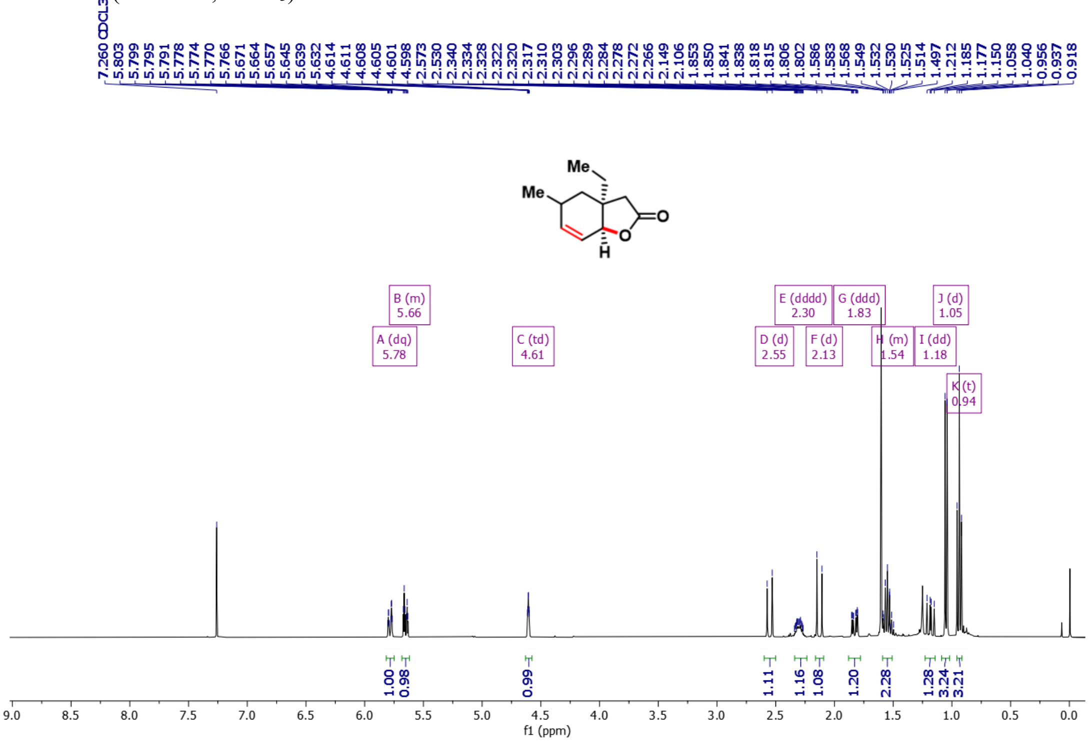

^{13}C NMR (101 MHz, $CDCl_3$)

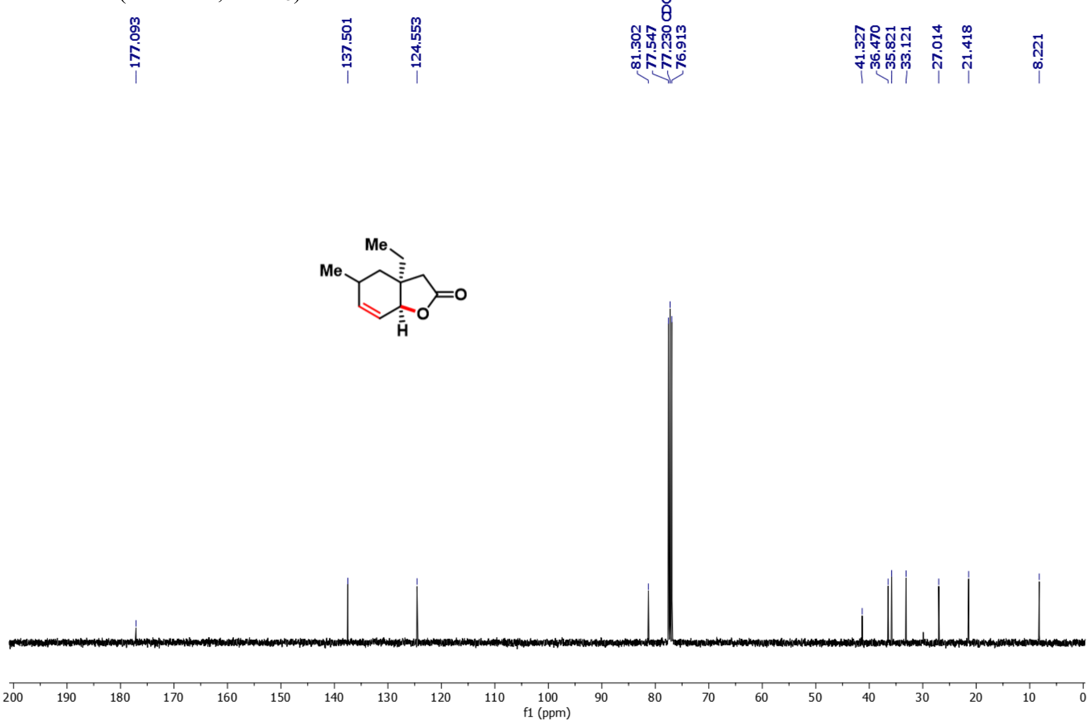

Compound 2r

(3a*S*,7a*S*)-3a-Isobutyl-5-methyl-3a,4,5,7a-tetrahydrobenzofuran-2(3H)-one

^{1}H NMR (400 MHz, $CDCl_3$)

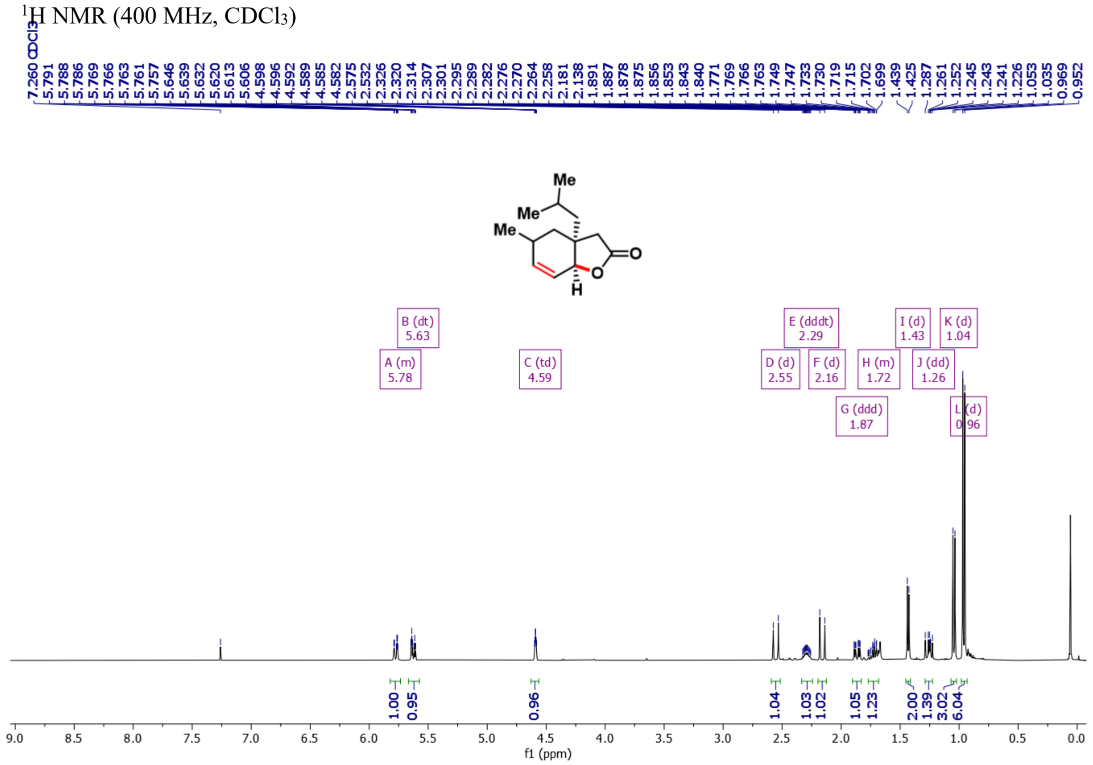

^{13}C NMR (101 MHz, $CDCl_3$)

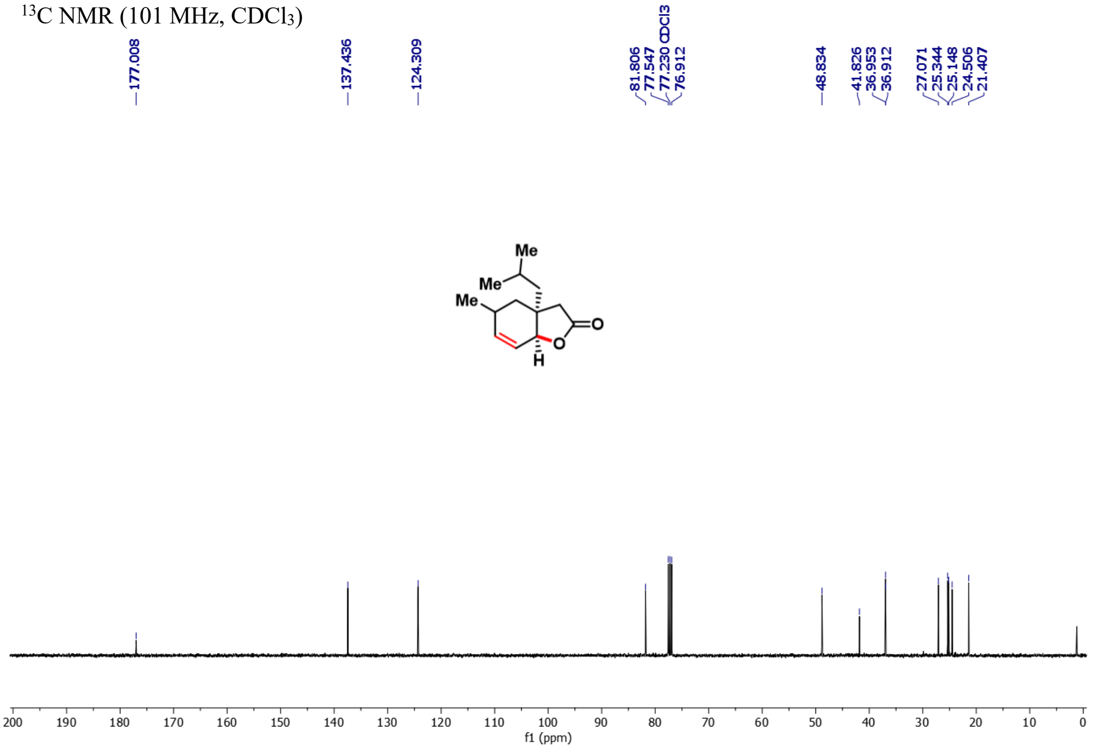

Compound 2s

(3a*S*,7a*S*)-3a-Methyl-5-phenyl-3a,4,5,7a-tetrahydrobenzofuran-2(3H)-one

^{1}H NMR (400 MHz, $CDCl_3$)

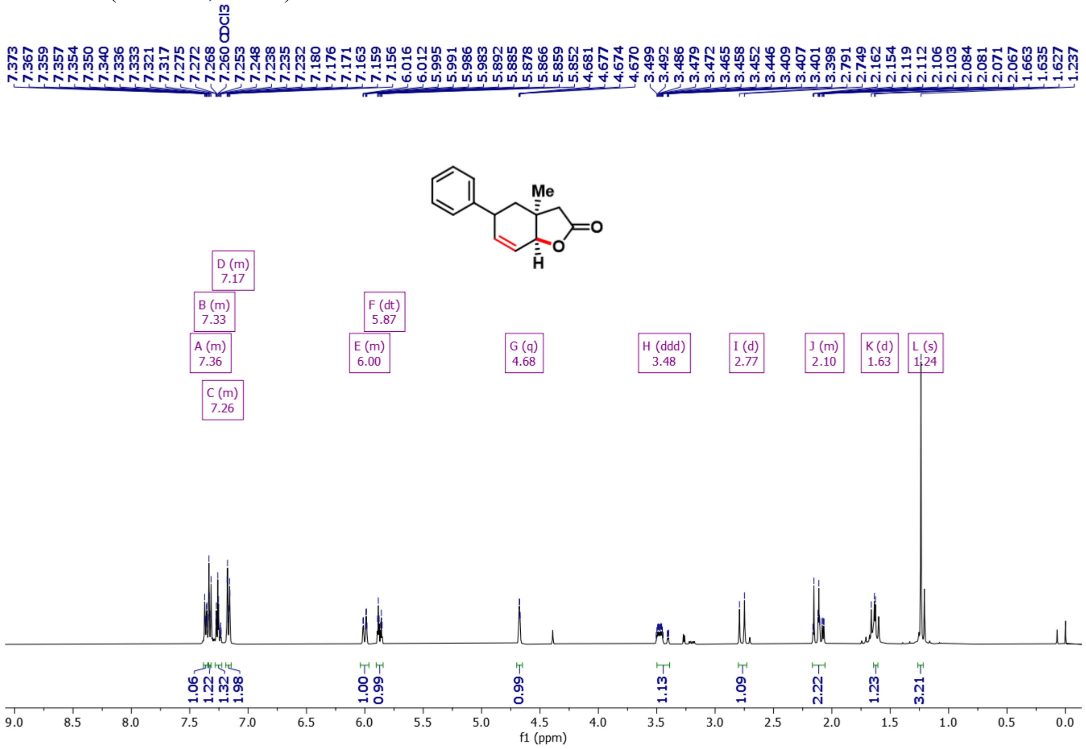

^{13}C NMR (101 MHz, $CDCl_3$)

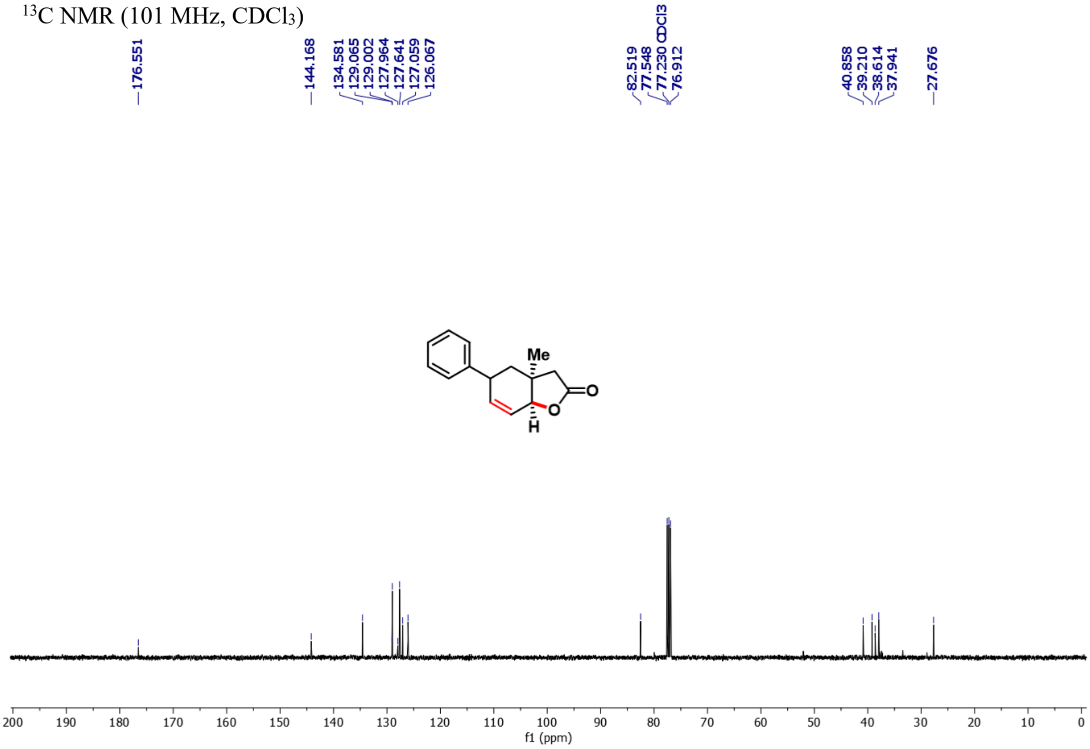

Compound 2t

(3a*S*,7a*R*)-3a-Methyl-7-phenyl-3a,4,5,7a-tetrahydrobenzofuran-2(3H)-one

^{1}H NMR (400 MHz, $CDCl_3$)

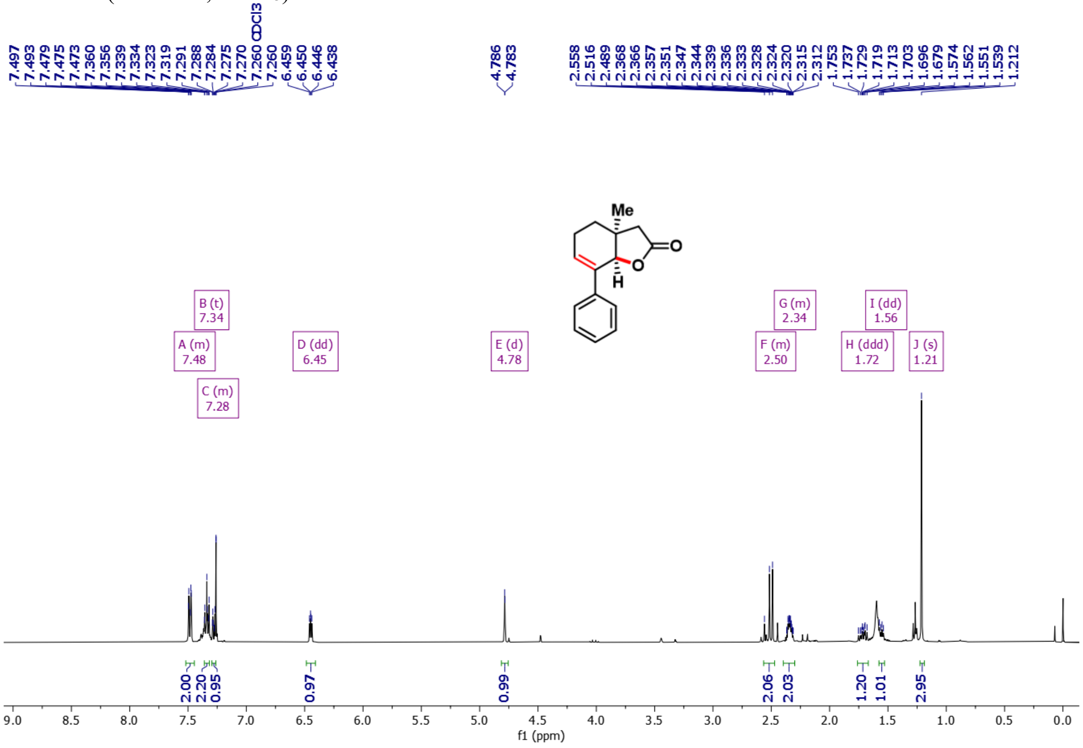

^{13}C NMR (101 MHz, $CDCl_3$)

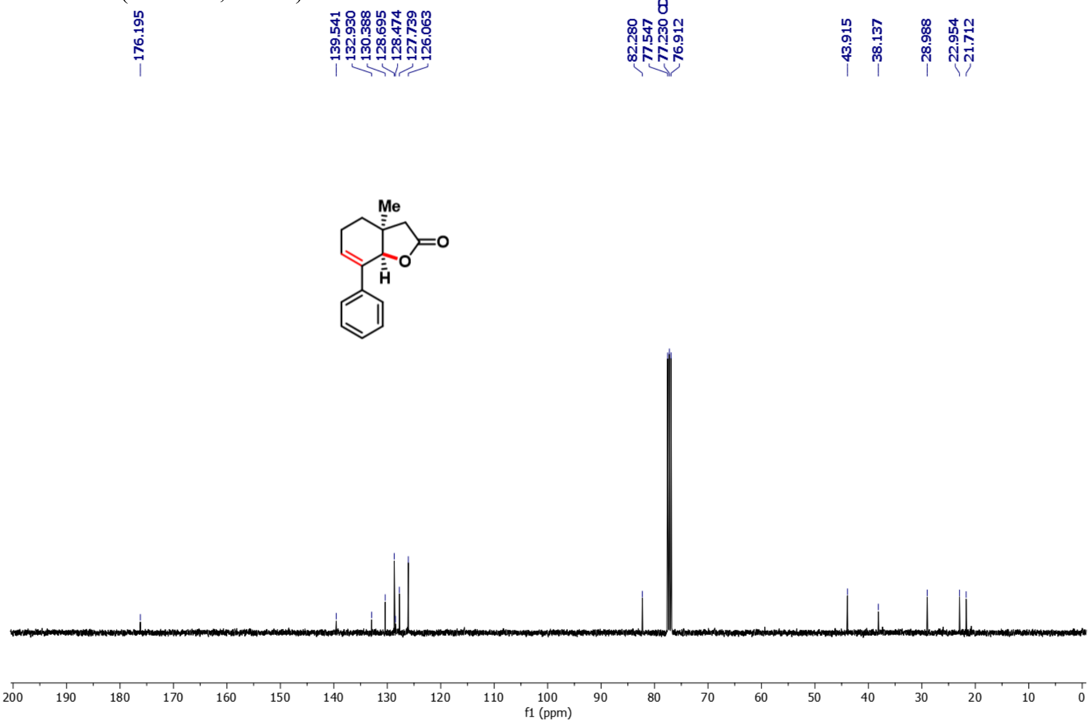

Compound 2u

(3a*S*,7a*S*)-3a-Methyl-5-(*p*-tolyl)-3a,4,5,7a-tetrahydrobenzofuran-2(3H)-one

^{1}H NMR (400 MHz, $CDCl_3$)

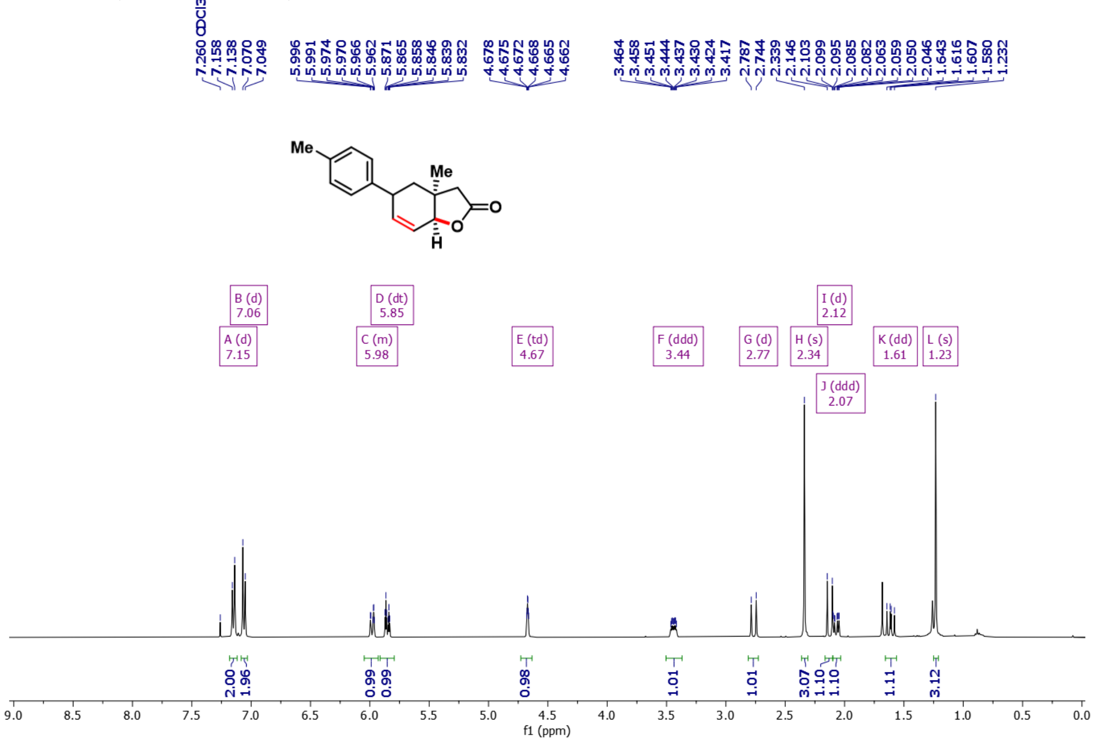

^{13}C NMR (101 MHz, $CDCl_3$)

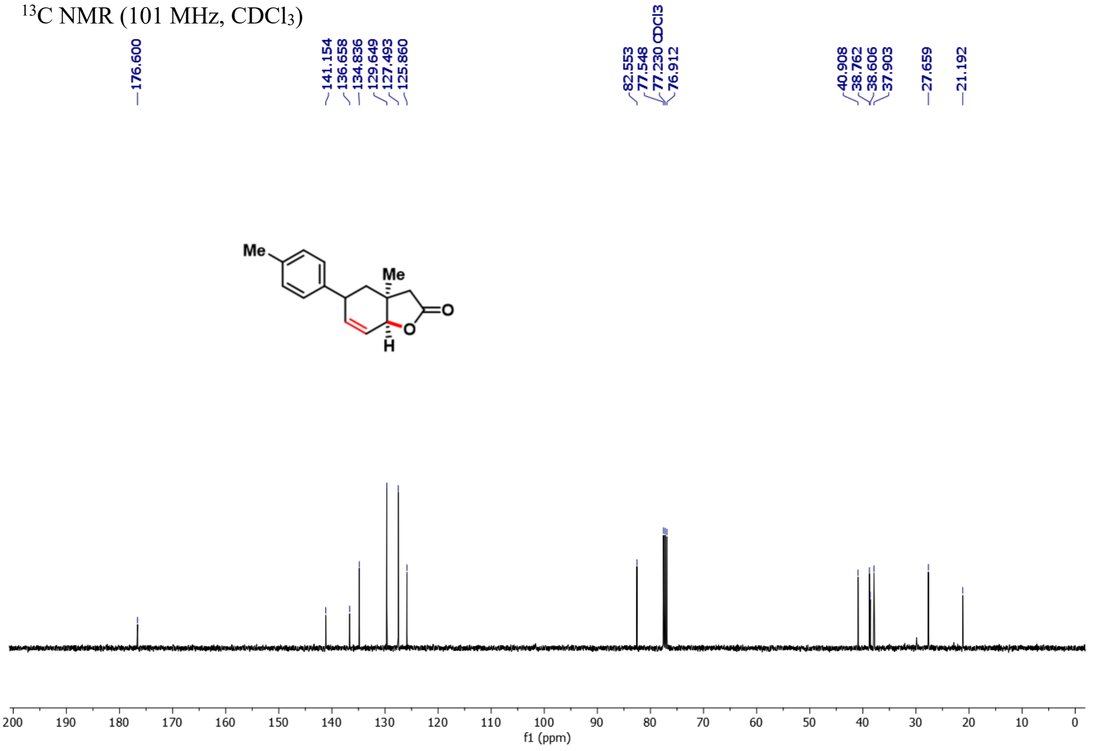

NOE experiment:

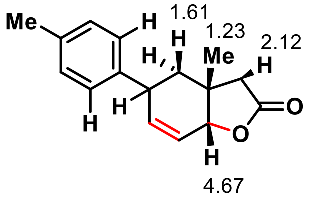

H (1.23) —Strong NOE→ H (1.61)

H (1.23) —Strong NOE→ H (2.12)

H (1.23) —Strong NOE→ H (4.67)

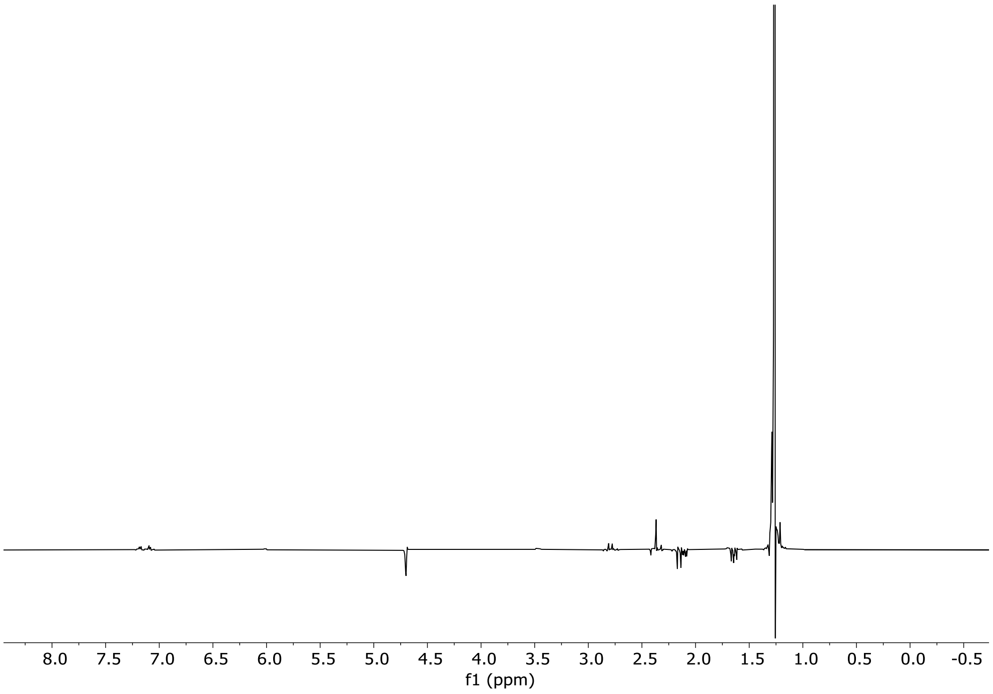

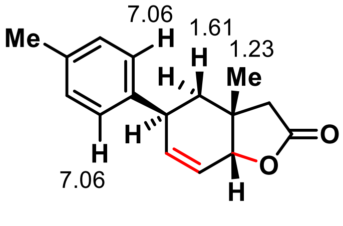

H (1.61) —Strong NOE→ H (7.06)

H (1.61) —Strong NOE→ H (1.23)

H (1.61) —Strong NOE→ H (2.12)

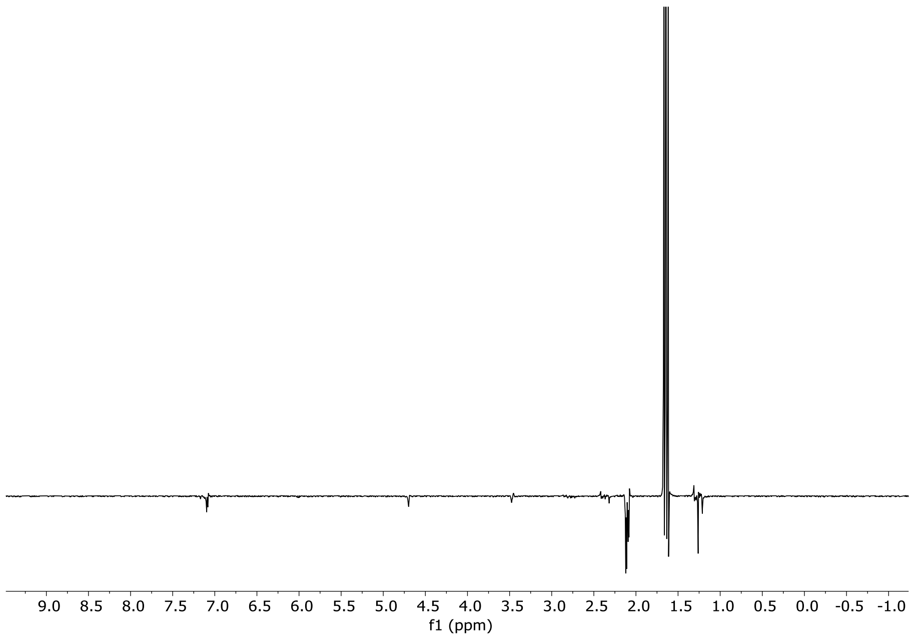

NOESY Experiment:

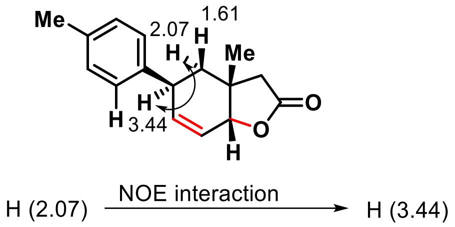

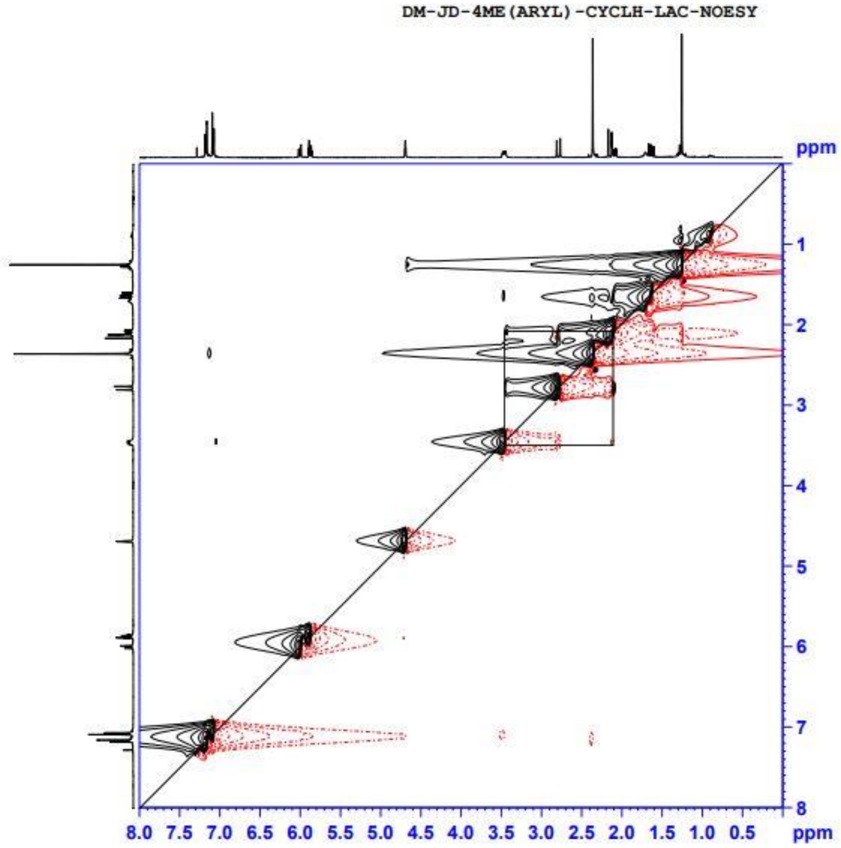

Compound 2v

(3a*S*,7a*R*)-3a-Methyl-7-(*p*-tolyl)-3a,4,5,7a-tetrahydrobenzofuran-2(3H)-one

^{1}H NMR (400 MHz, $CDCl_3$)

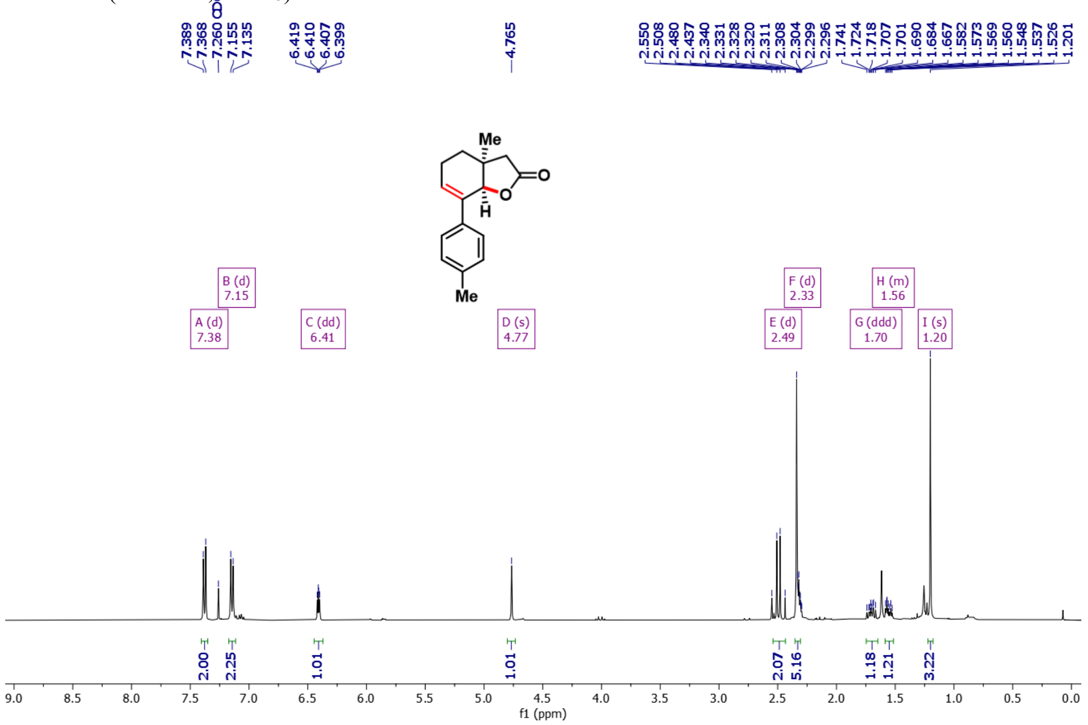

^{13}C NMR (101 MHz, $CDCl_3$)

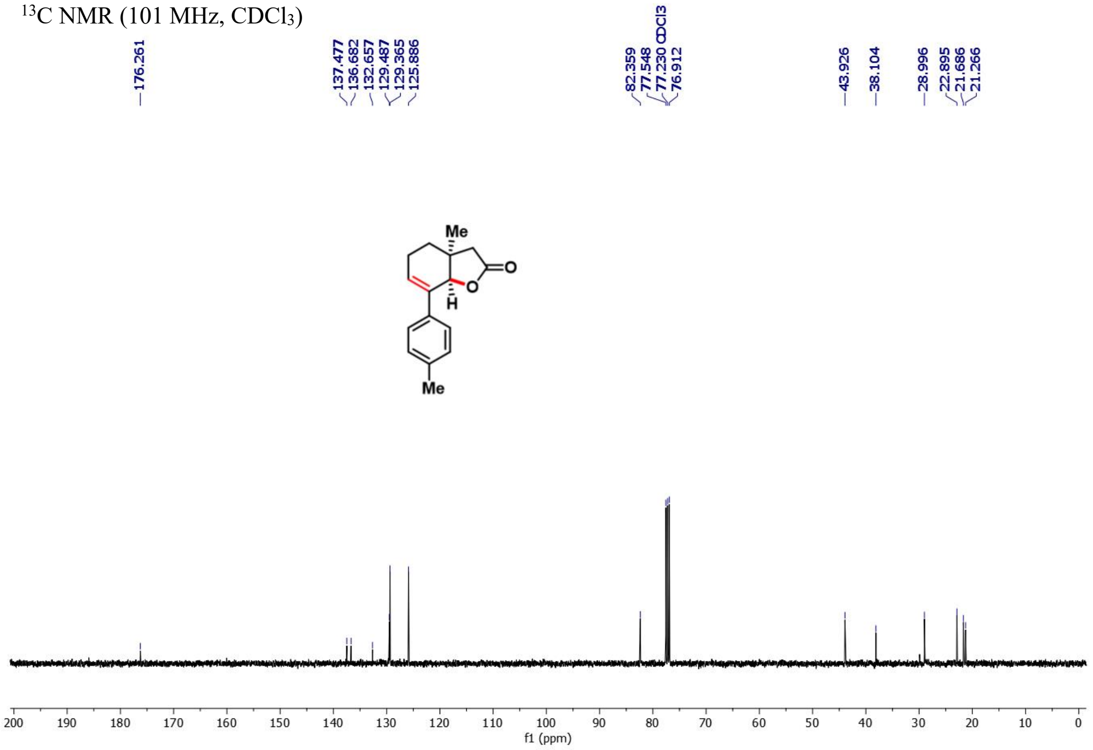

Compound 2w

(3a*S*,7a*S*)-5-(2-Fluorophenyl)-3a-methyl-3a,4,5,7a-tetrahydrobenzofuran-2(3H)-one

^{1}H NMR (400 MHz, CDCl$_3$)

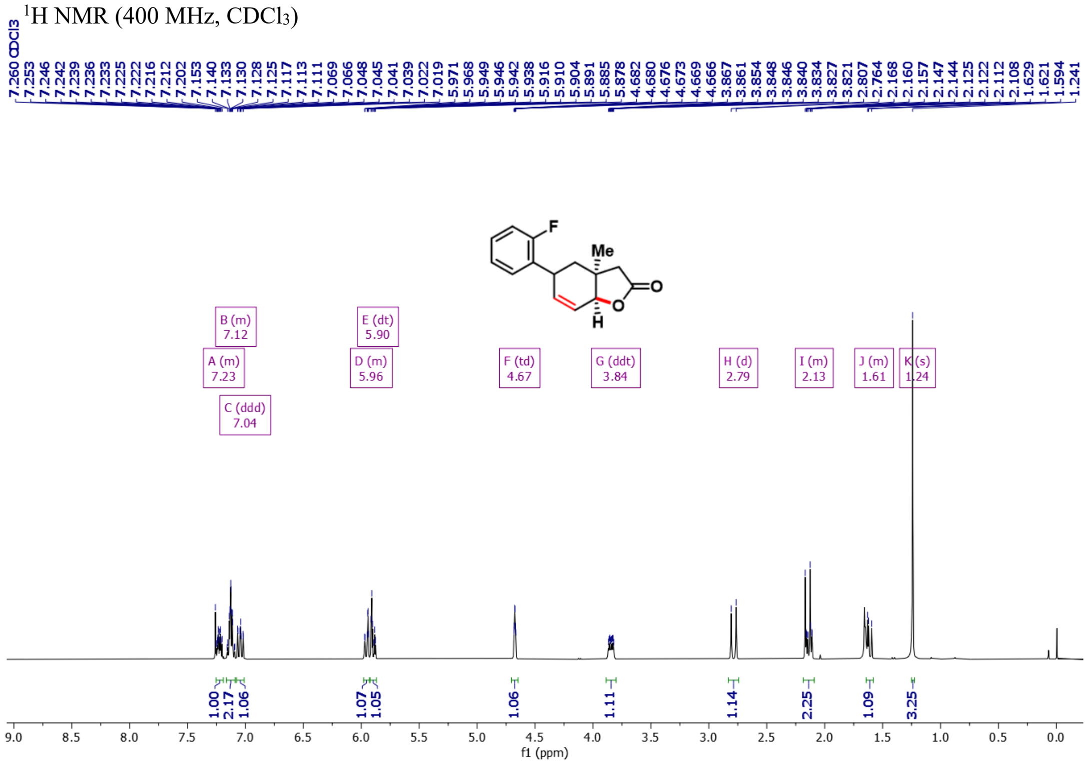

^{13}C NMR (101 MHz, CDCl$_3$)

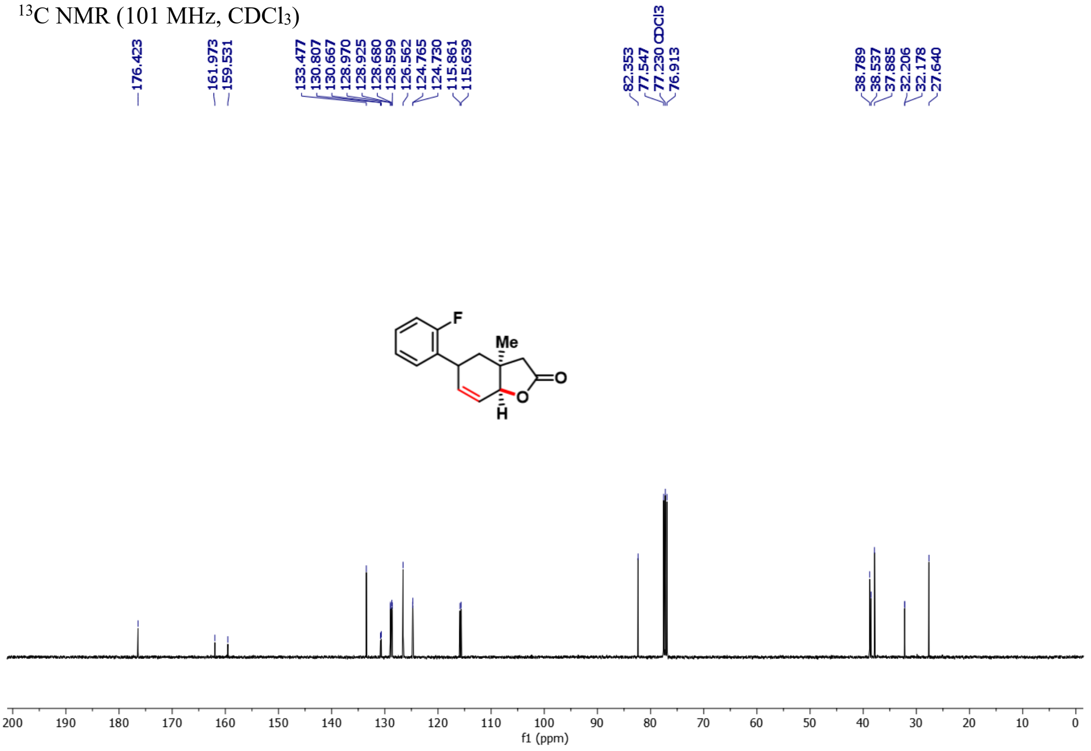

^{19}F NMR (376 MHz, $CDCl_3$)

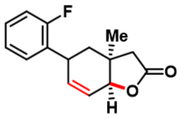

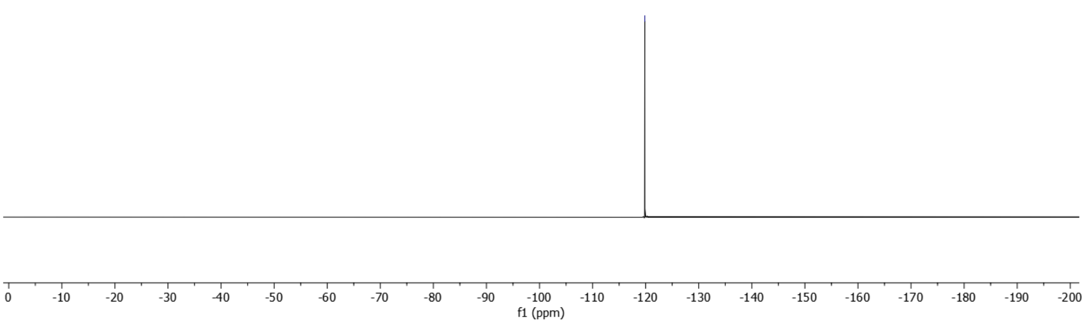

Compound 2x

(3a*S*,7a*S*)-3a-Methyl-5-(naphthalen-1-yl)-3a,4,5,7a-tetrahydrobenzofuran-2(3H)-one

^{1}H NMR (400 MHz, $CDCl_3$)

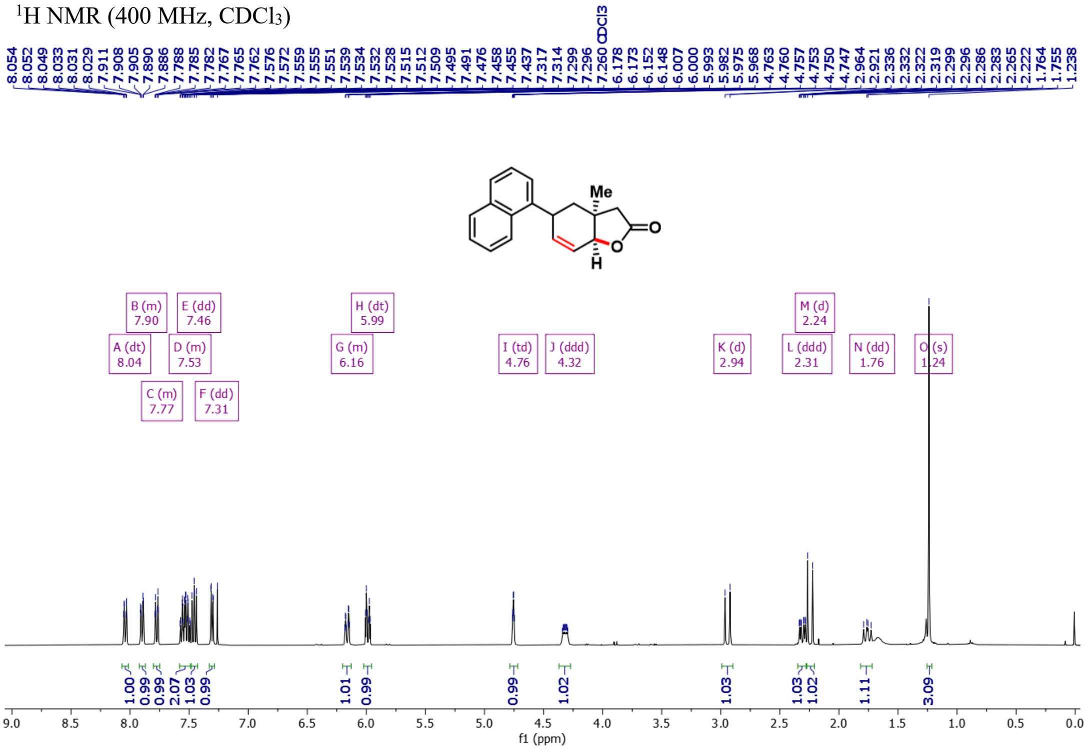

^{13}C NMR (101 MHz, $CDCl_3$)

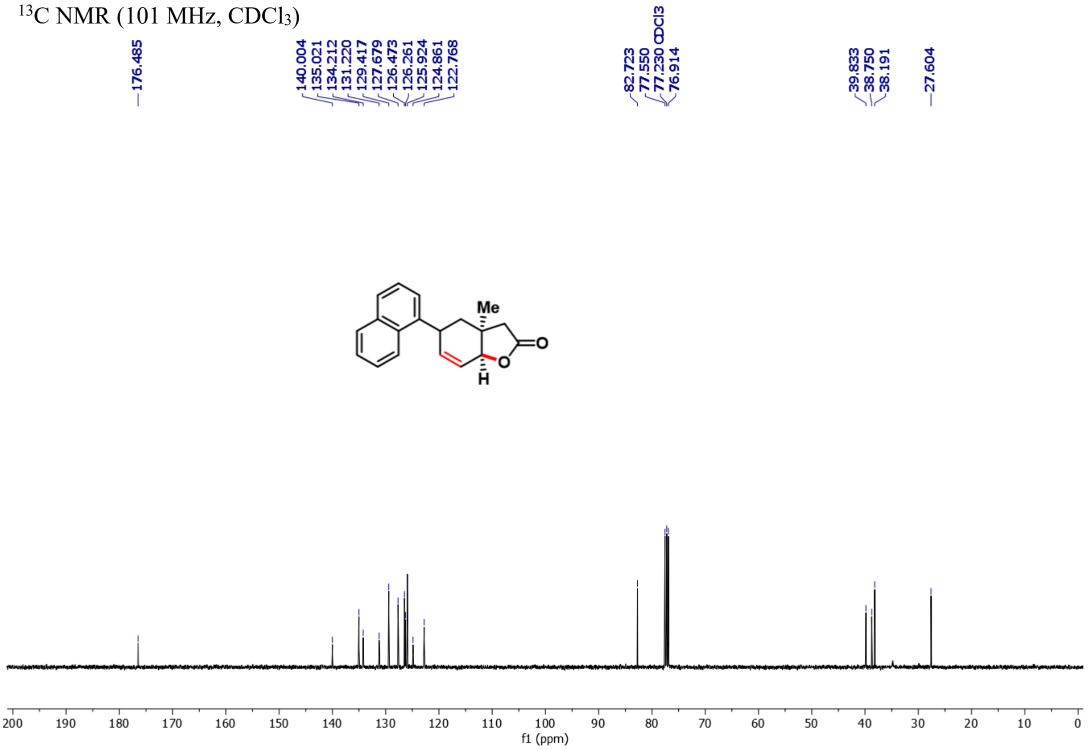

Compound 2y

(3a*S*,7a*S*)-3a,6-Dimethyl-3a,4,5,7a-tetrahydrobenzofuran-2(3H)-one

^{1}H NMR (400 MHz, $CDCl_3$)

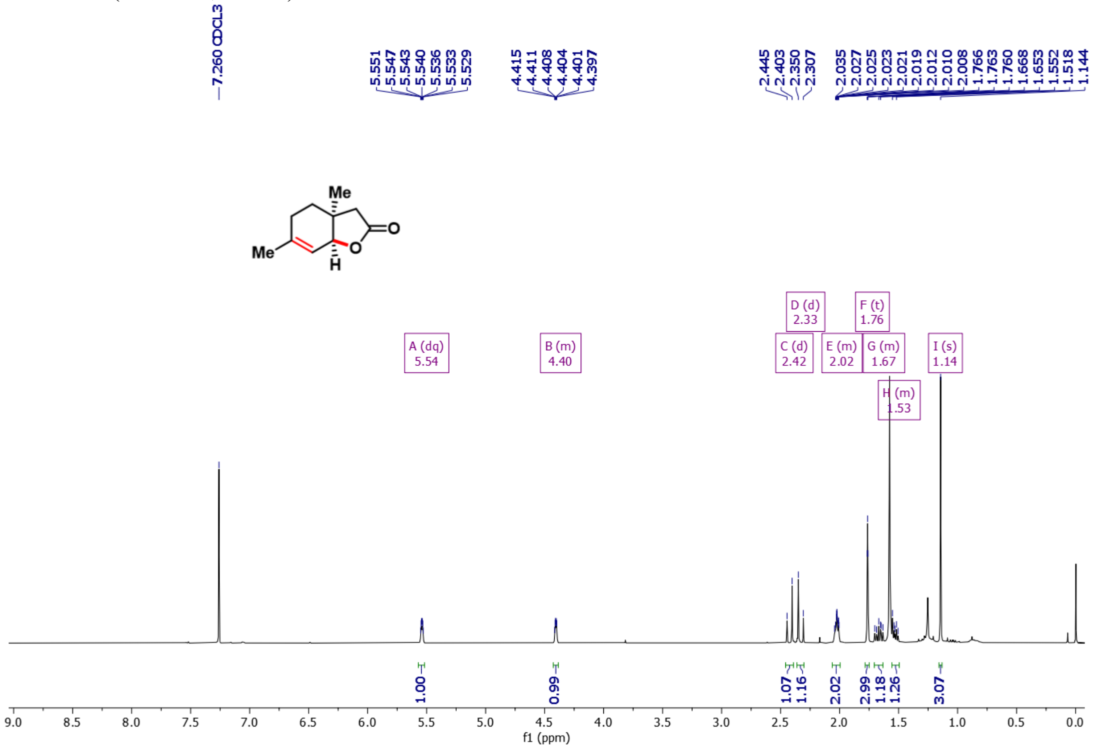

^{13}C NMR (101 MHz, $CDCl_3$)

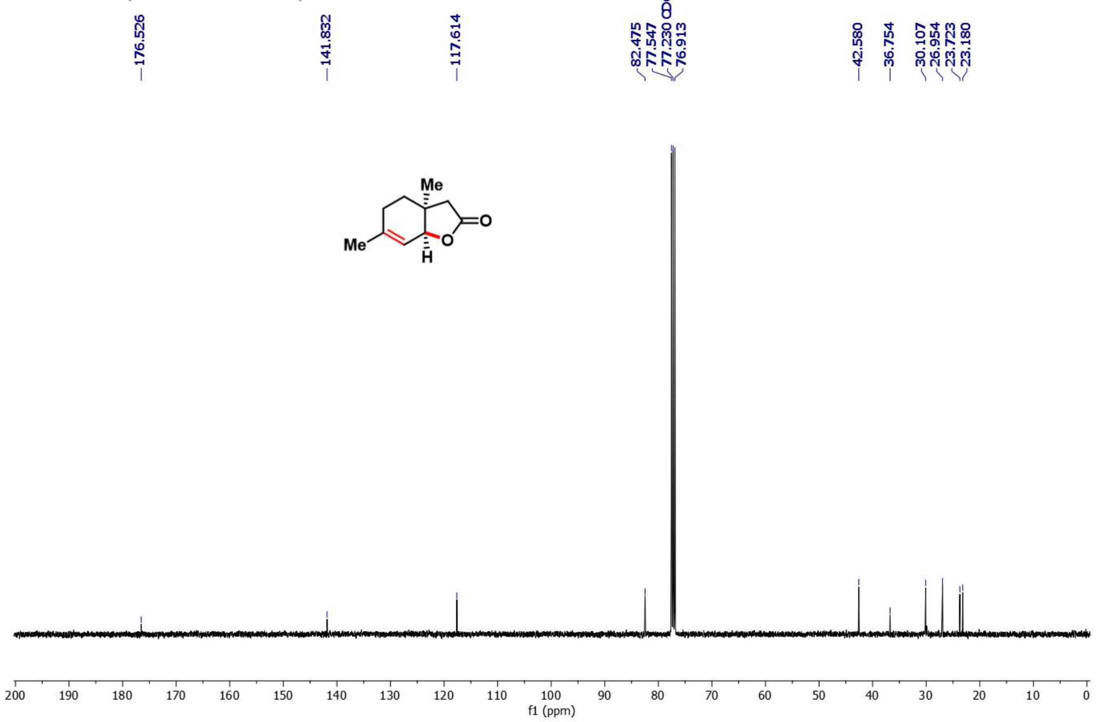

Compound 2z

(3a*S*,7a*S*)-6-Methyl-3a-propyl-3a,4,5,7a-tetrahydrobenzofuran-2(3H)-one

^{1}H NMR (400 MHz, $CDCl_3$)

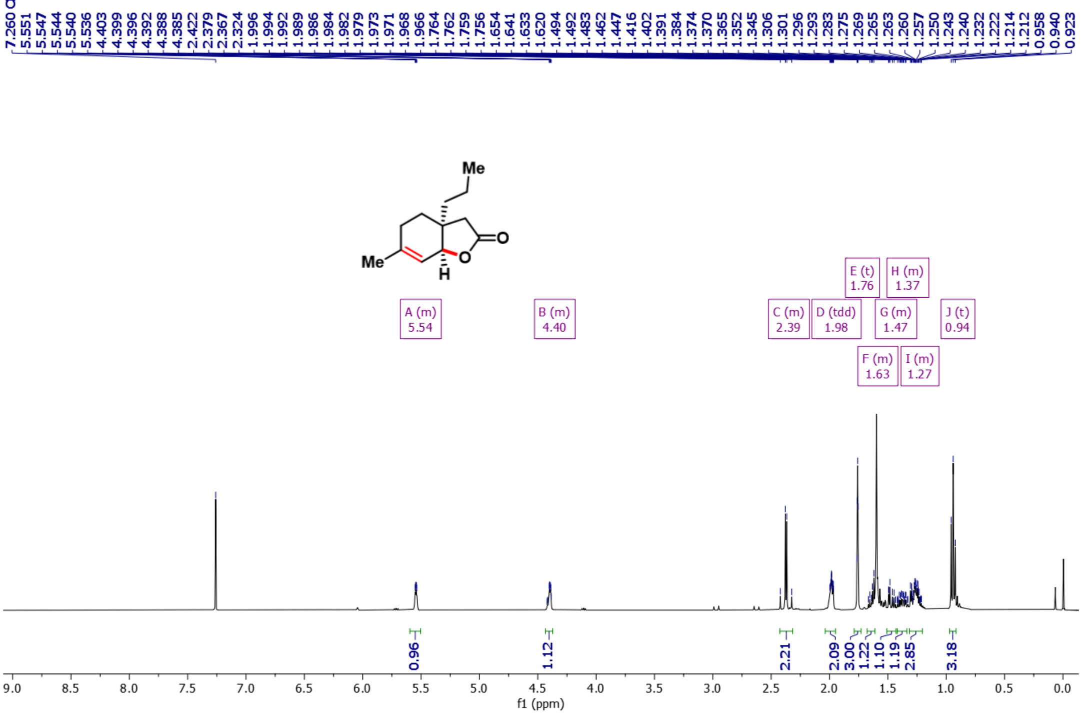

^{13}C NMR (126 MHz, $CDCl_3$)

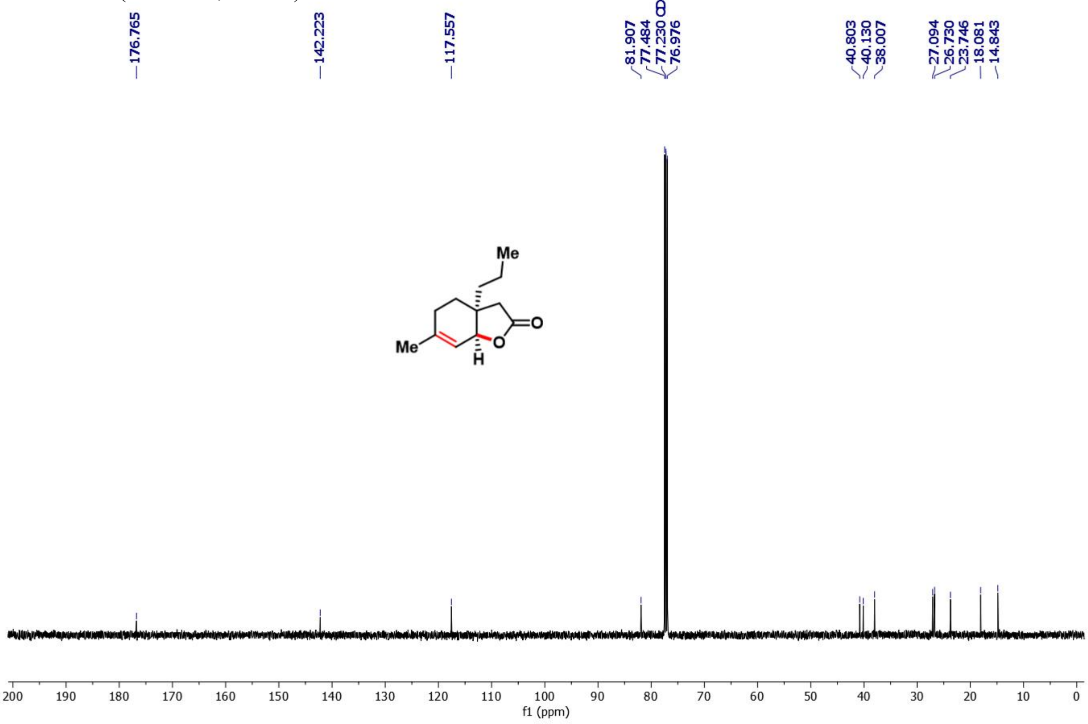

Compound 2aa

(3a*S*,7a*S*)-3a-Methyl-6-propyl-3a,4,5,7a-tetrahydrobenzofuran-2(3H)-one

^{1}H NMR (500 MHz, $CDCl_3$)

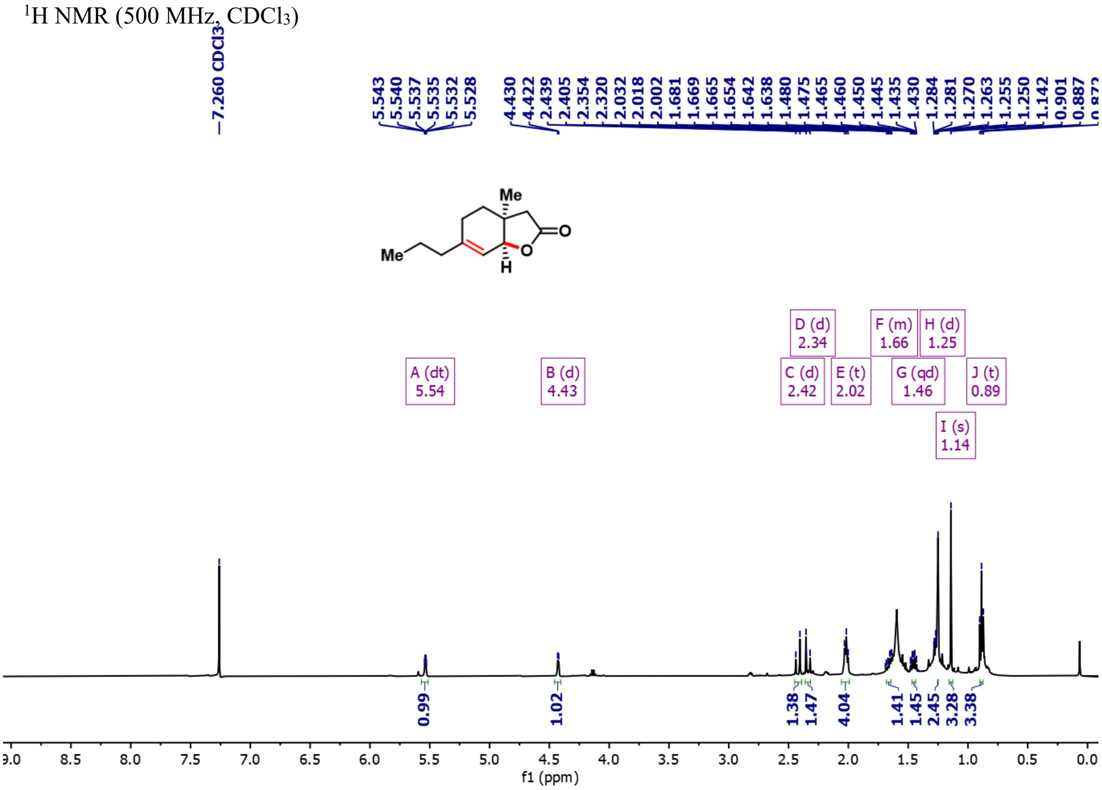

^{13}C NMR (126 MHz, $CDCl_3$)

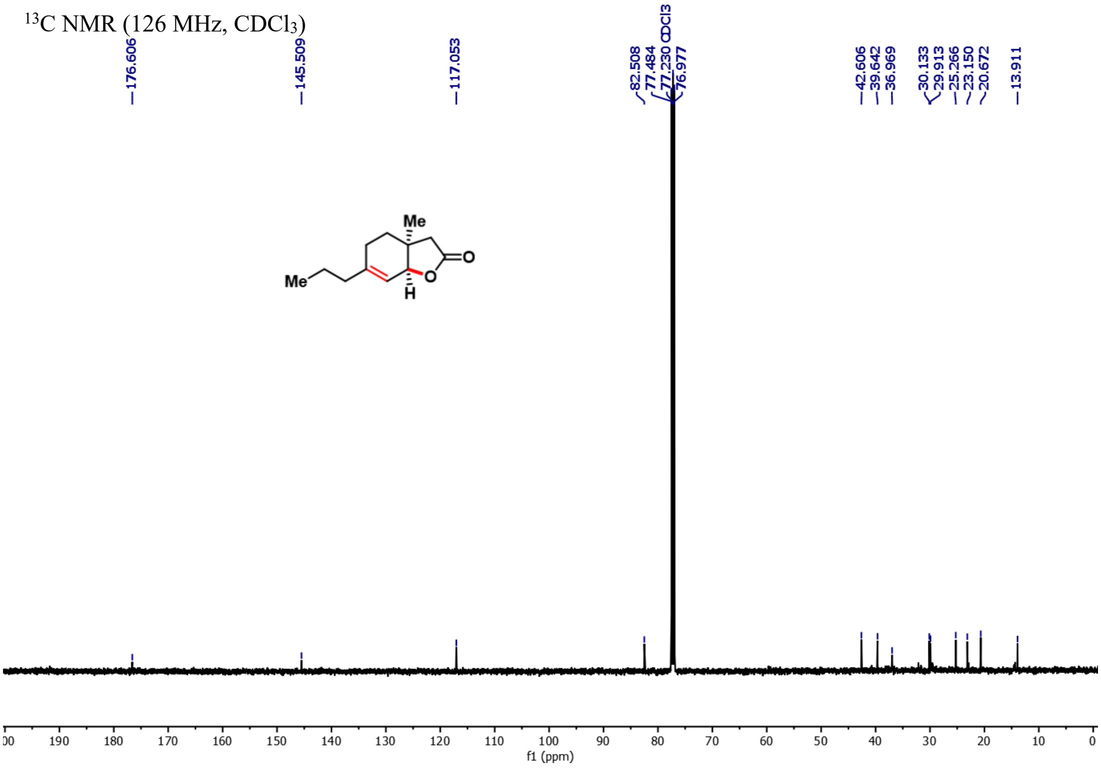

Compound 2ab

(3a*S*,7a*S*)-6-(*tert*-Butyl)-3a-methyl-3a,4,5,7a-tetrahydrobenzofuran-2(3H)-one

^{1}H NMR (400 MHz, $CDCl_3$)

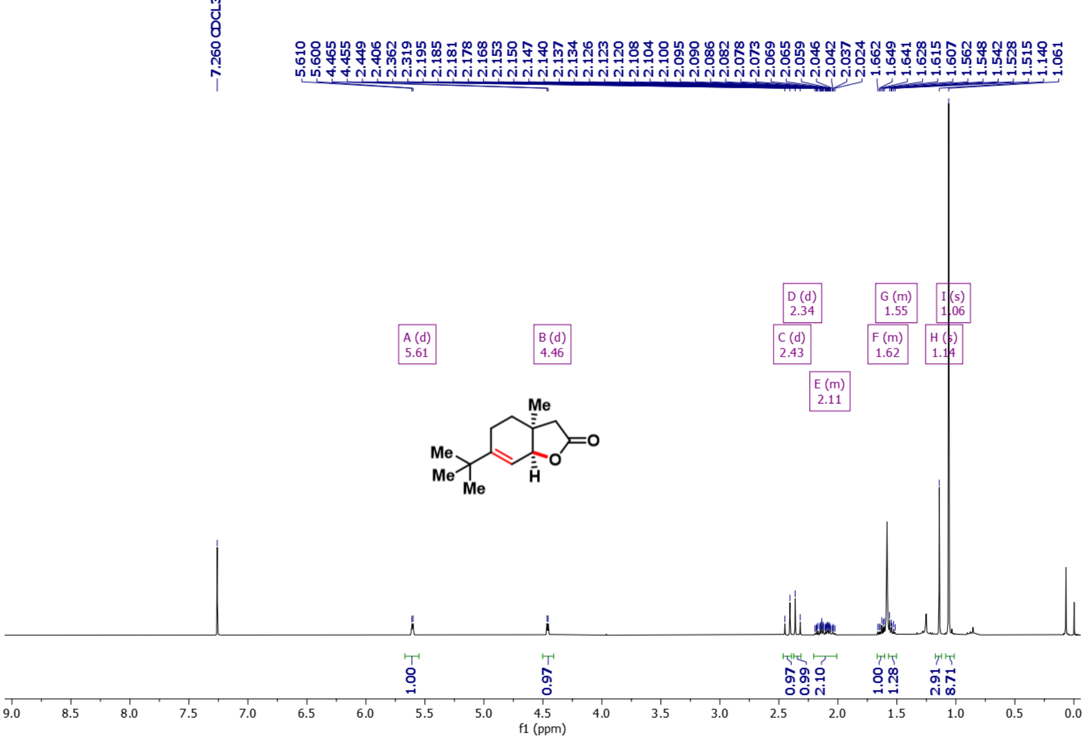

^{13}C NMR (126 MHz, $CDCl_3$)

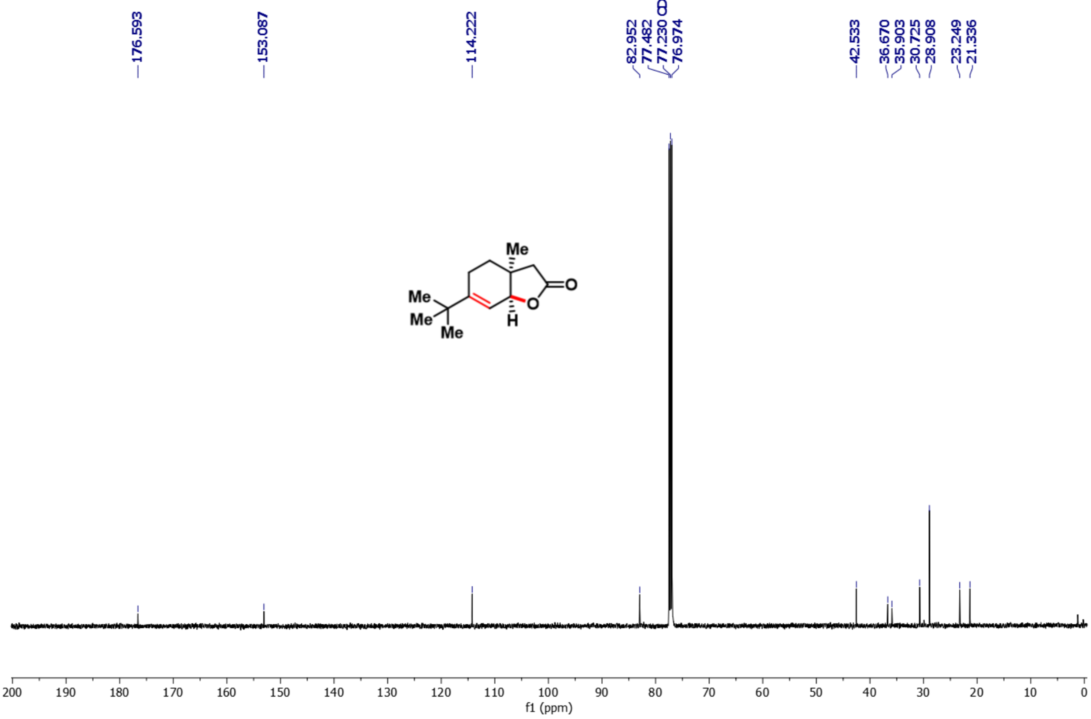

Compound 2ac

(3a*S*,7a*S*)-3a-Methyl-6-(*tert*-pentyl)-3a,4,5,7a-tetrahydrobenzofuran-2(3H)-one

^{1}H NMR (500 MHz, $CDCl_3$)

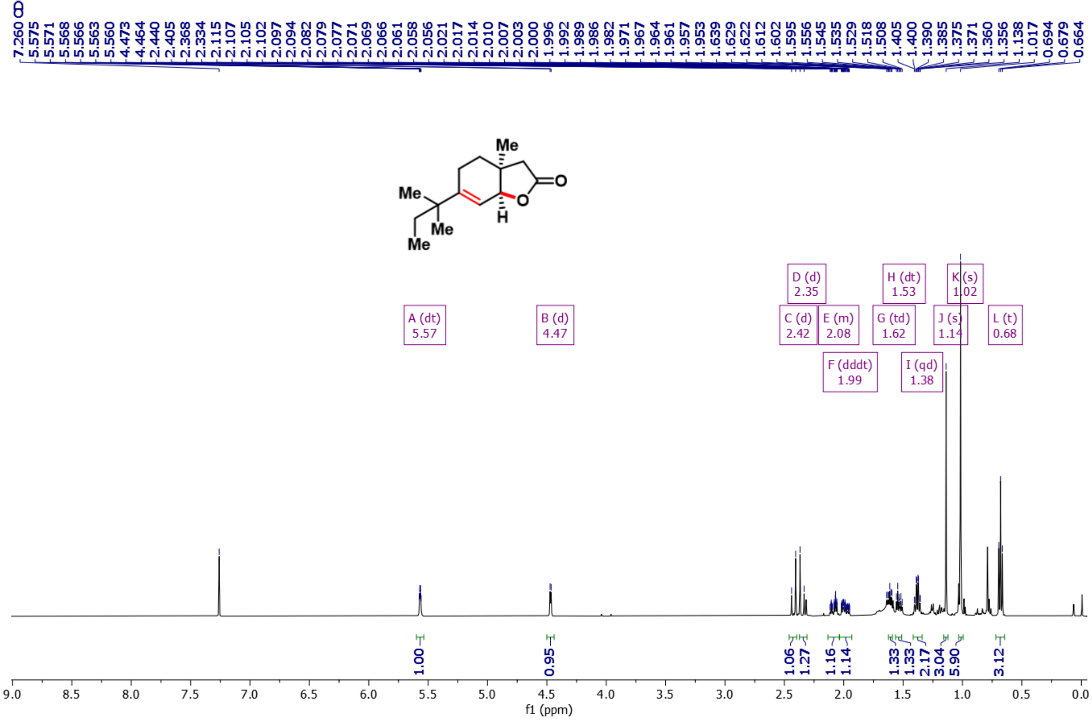

^{13}C NMR (126 MHz, $CDCl_3$)

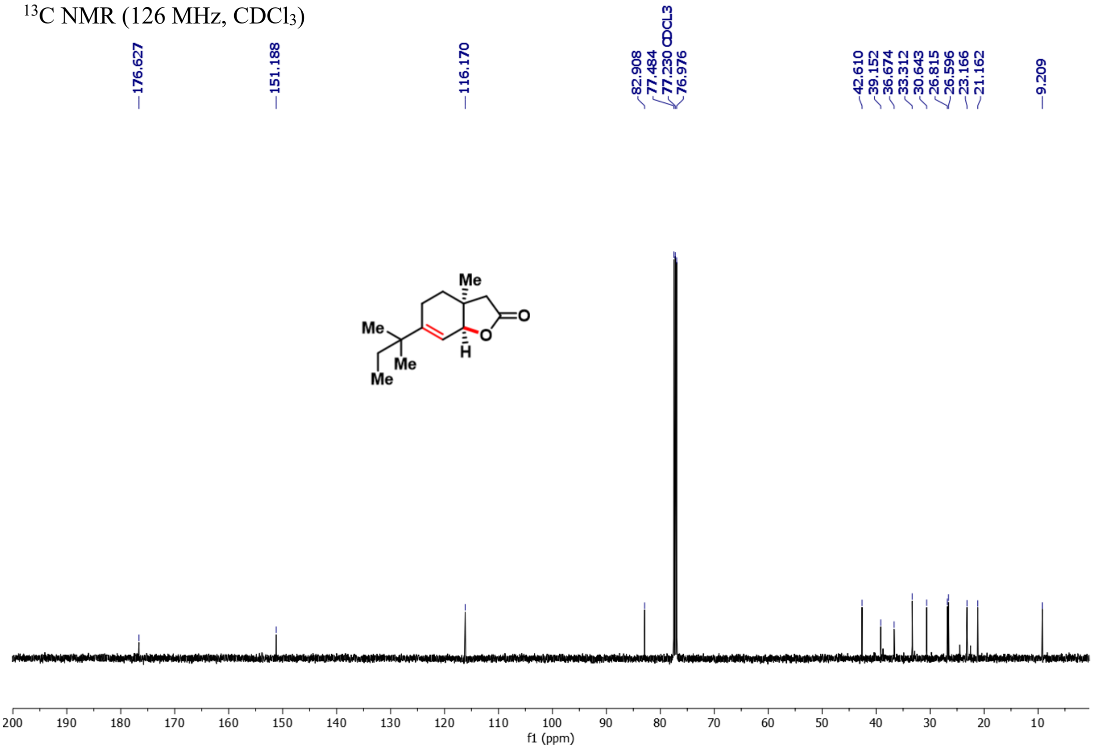

Compound 2ad

(3a*S*,7a*S*)-3a-Ethyl-6-(*tert*-pentyl)-3a,4,5,7a-tetrahydrobenzofuran-2(3H)-one

^{1}H NMR (400 MHz, $CDCl_3$)

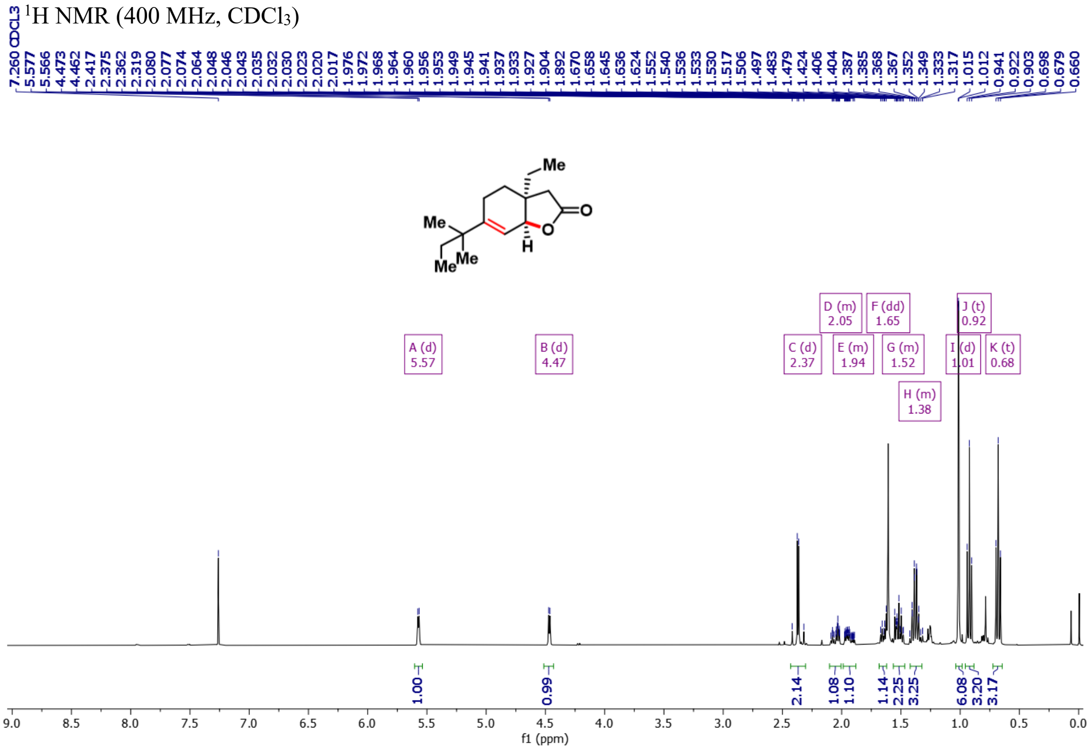

^{13}C NMR (101 MHz, $CDCl_3$)

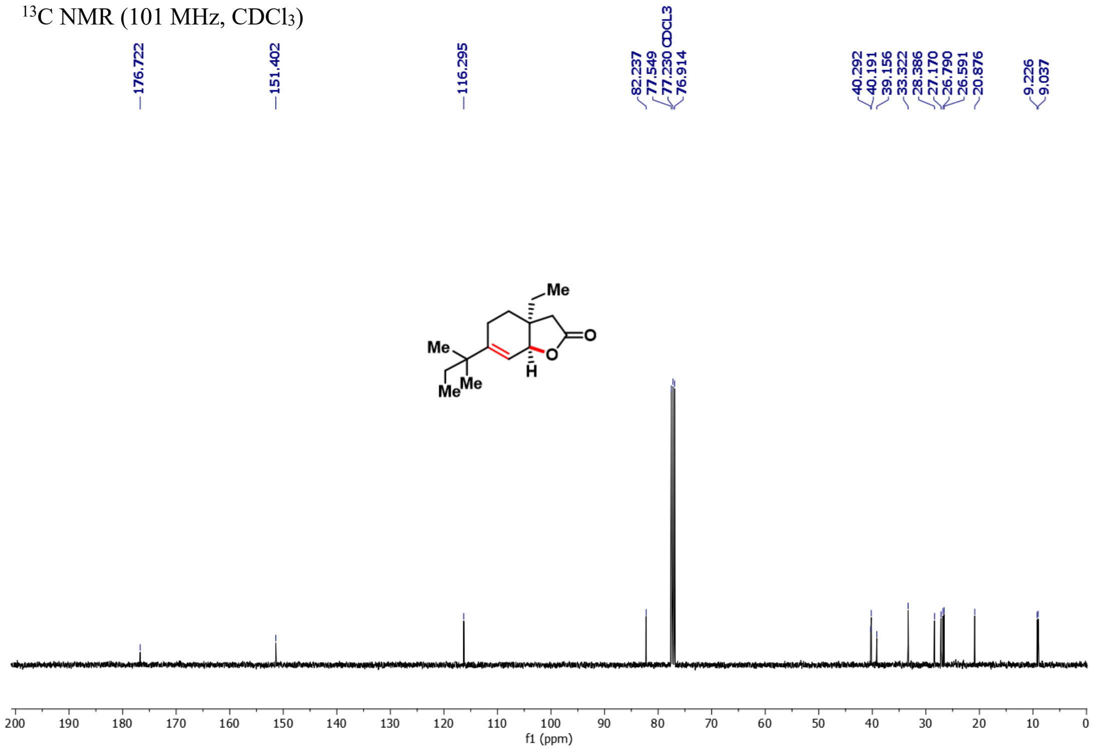

Compound 2ae

(3a*S*,7a*S*)-3a-Methyl-6-(trifluoromethyl)-3a,4,5,7a-tetrahydrobenzofuran-2(3H)-one

^{1}H NMR (400 MHz, $CDCl_3$)

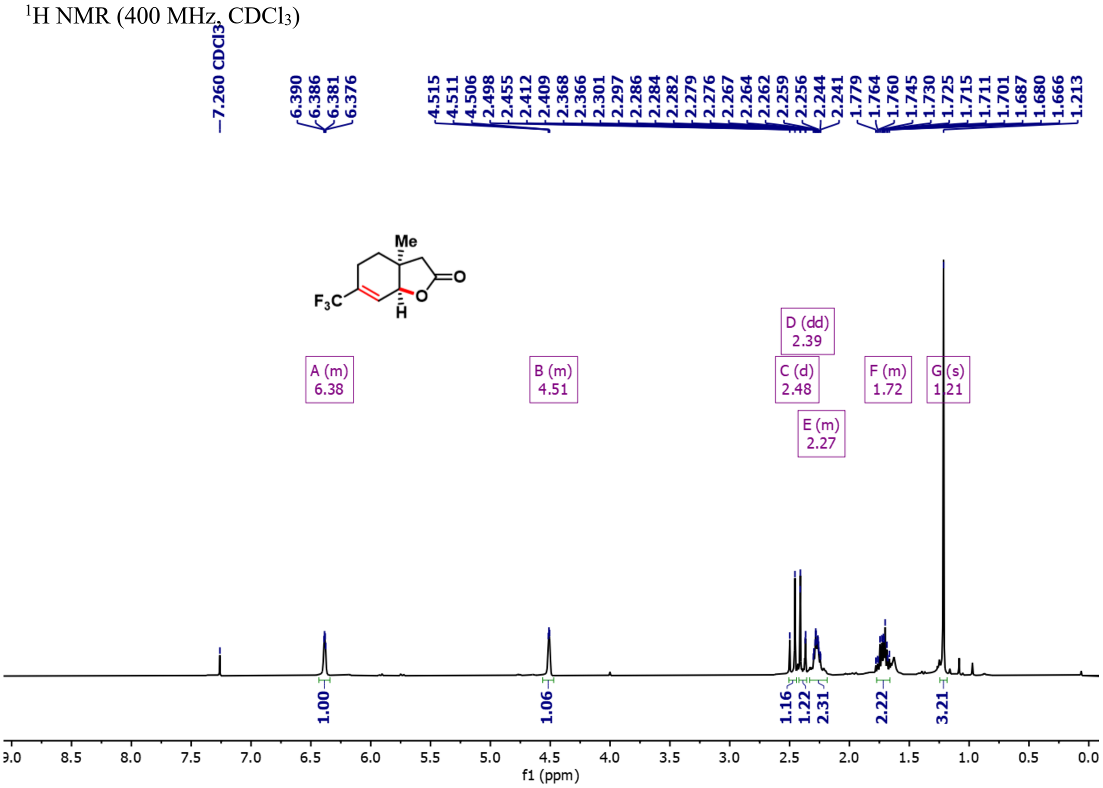

^{13}C NMR (101 MHz, $CDCl_3$)

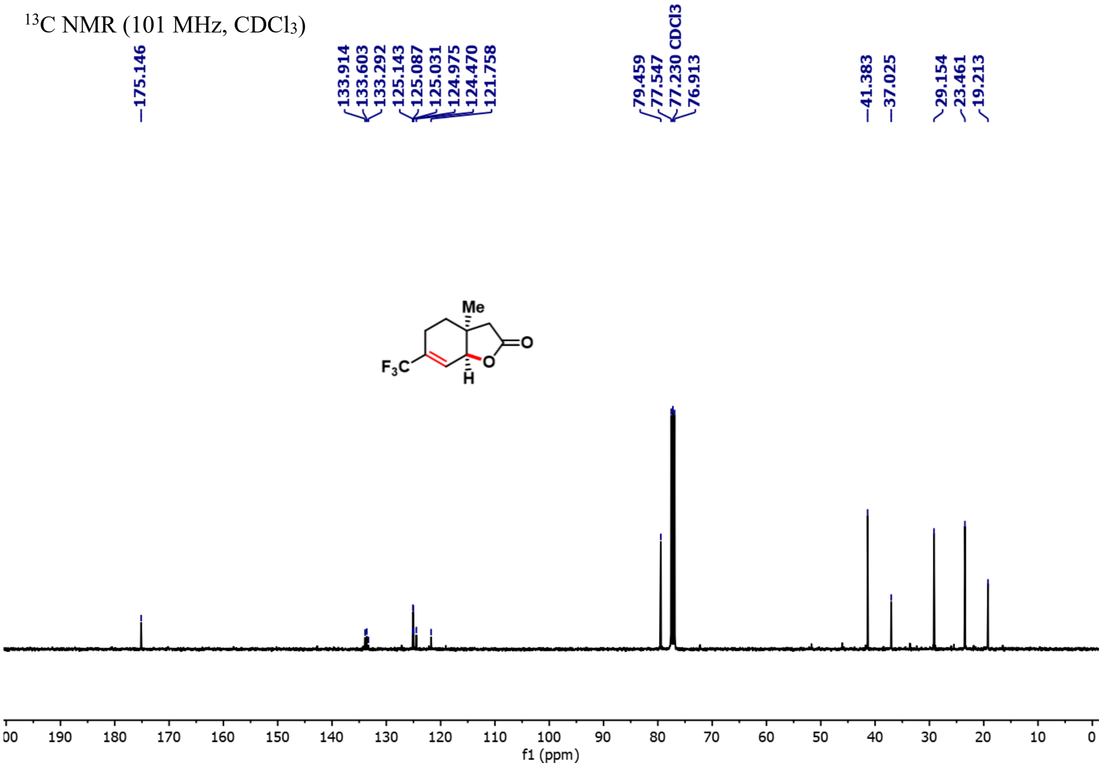

^{19}F NMR (376 MHz, $CDCl_3$)

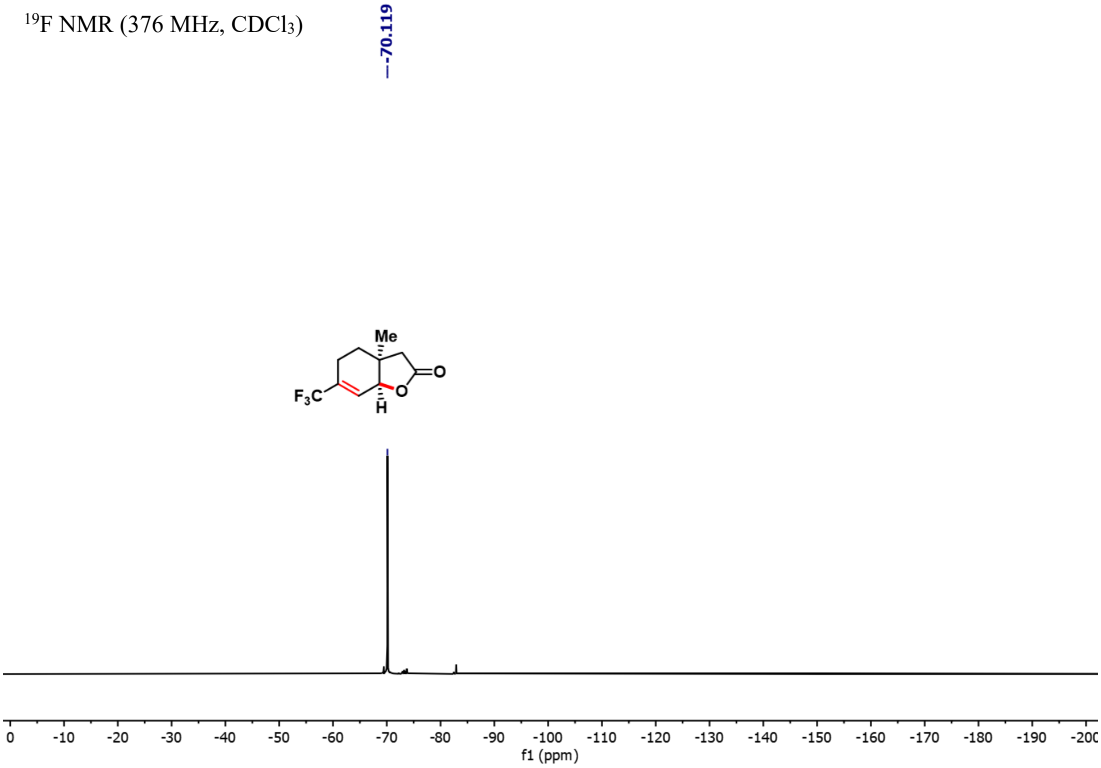

Compound 2af

(3a*S*,7a*S*)-3a-Methyl-6-phenyl-3a,4,5,7a-tetrahydrobenzofuran-2(3H)-one

^{1}H NMR (400 MHz, $CDCl_3$)

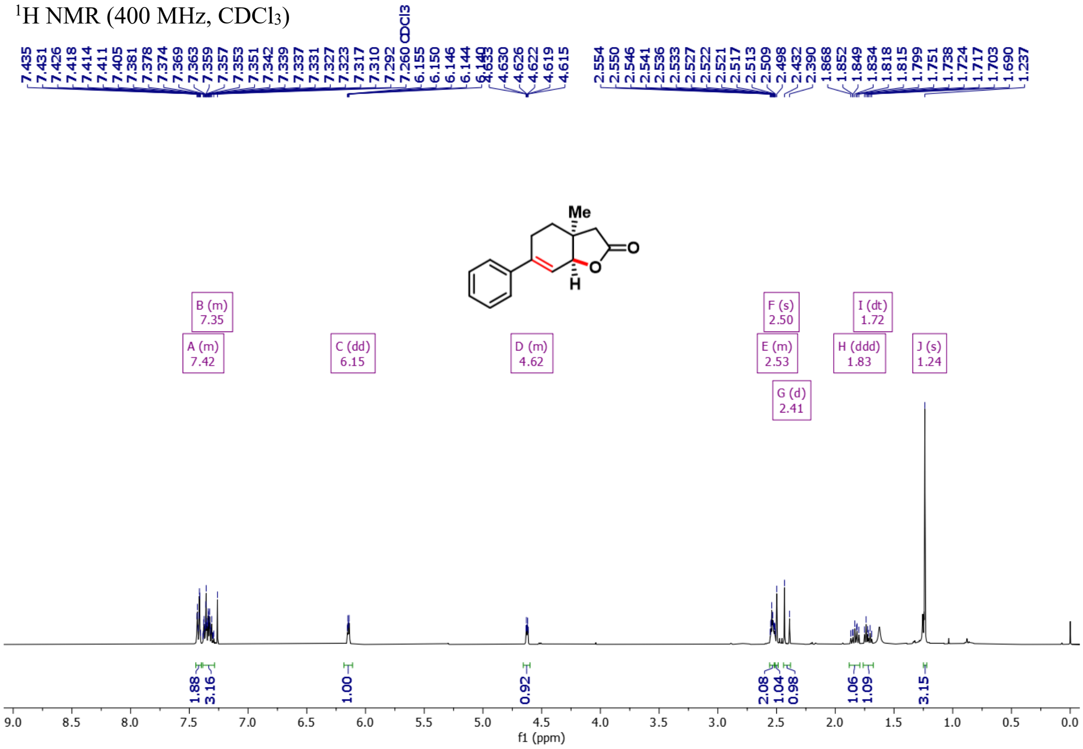

^{13}C NMR (101 MHz, $CDCl_3$)

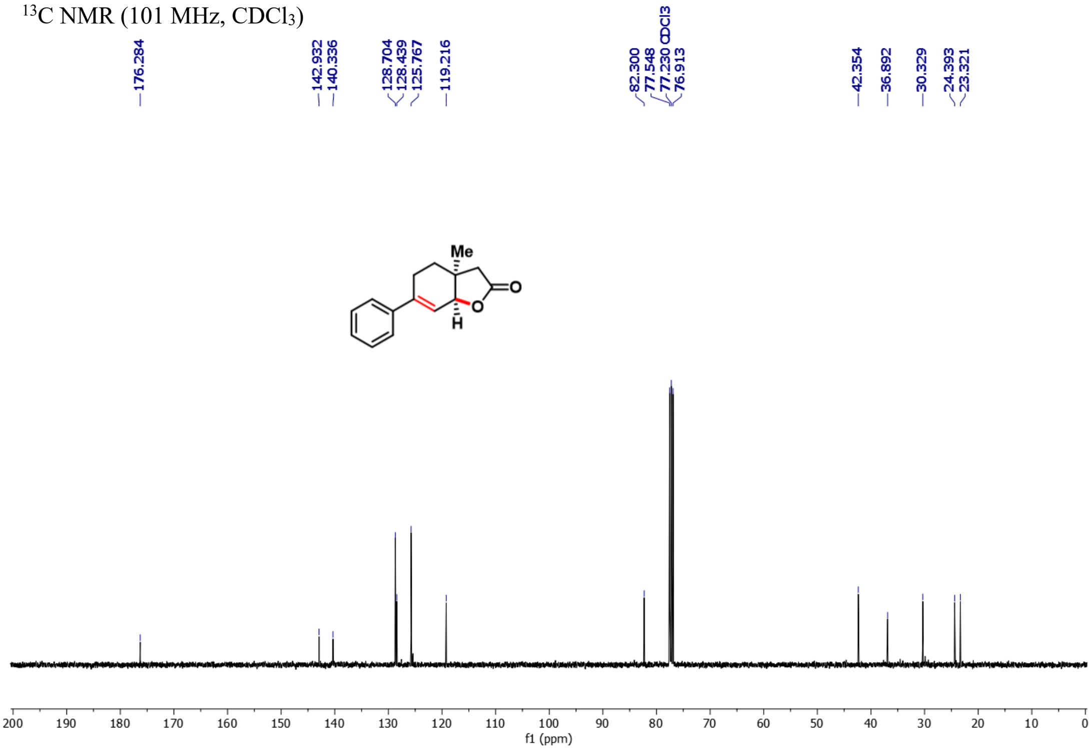

Compound 3a

(3a*S*,6a*S*)-3a-Methyl-3,3a,4,6a-tetrahydro-2H-cyclopenta[b]furan-2-one

^{1}H NMR (400 MHz, CDCl₃)

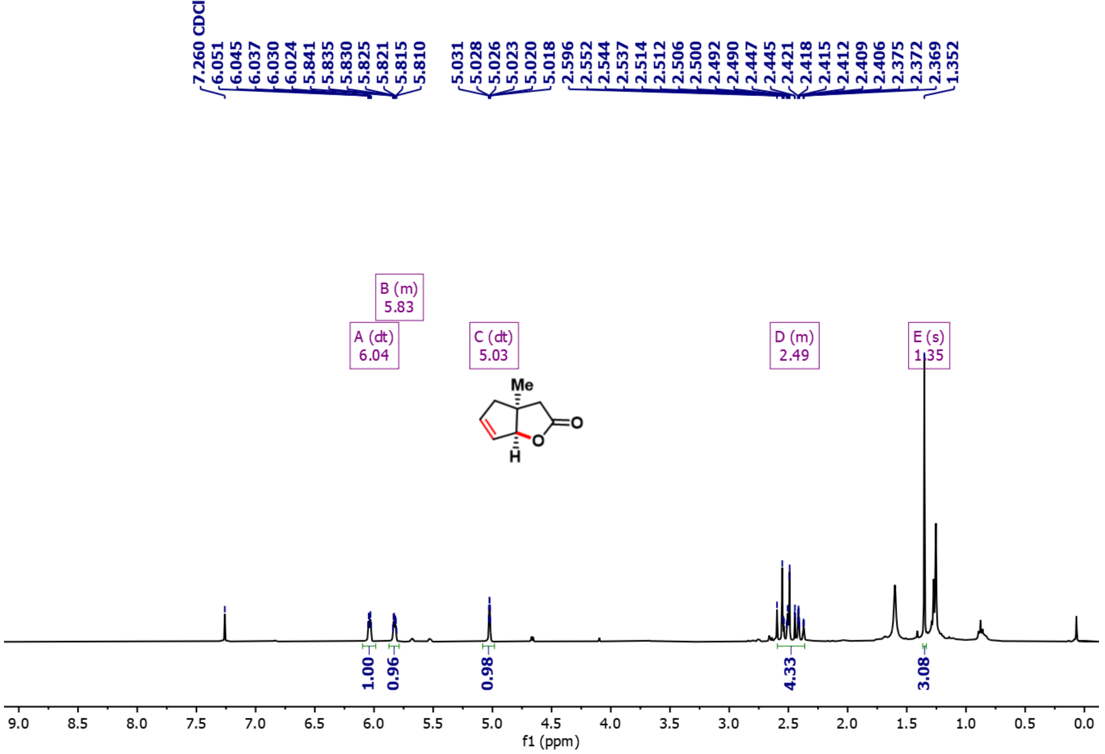

^{13}C NMR (101 MHz, CDCl₃)

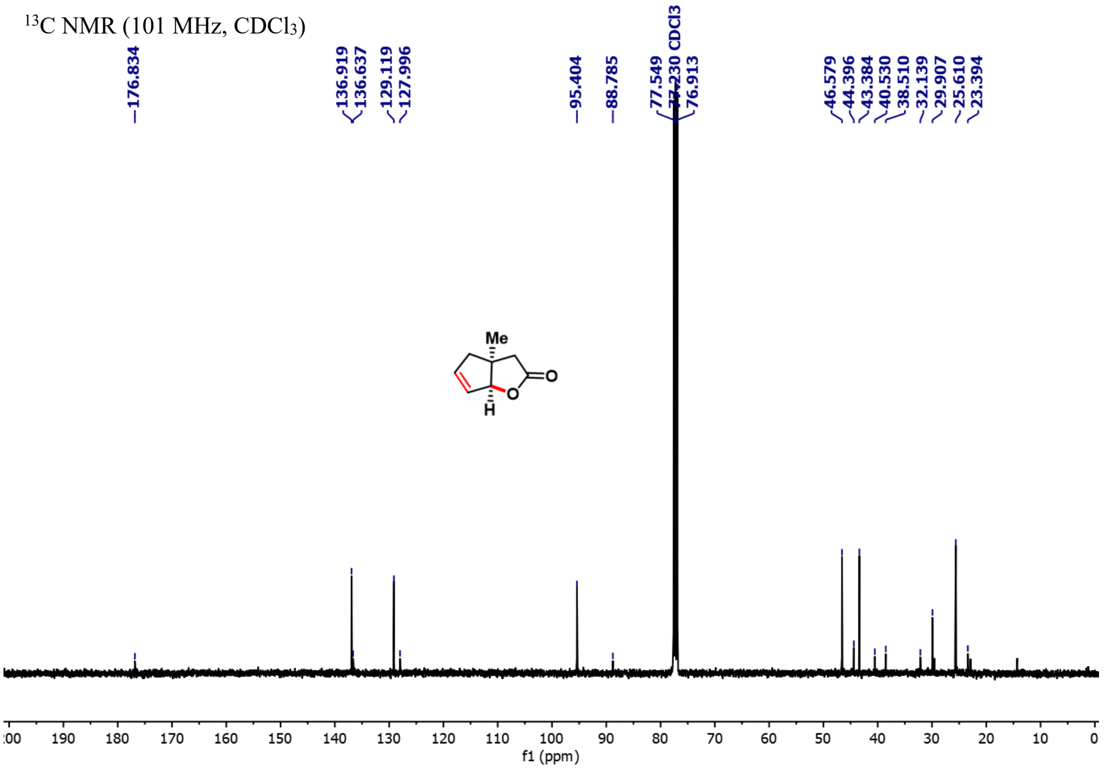

Compound 3b

(3a*S*,6a*S*)-3a-Propyl-3,3a,4,6a-tetrahydro-2H-cyclopenta[b]furan-2-one

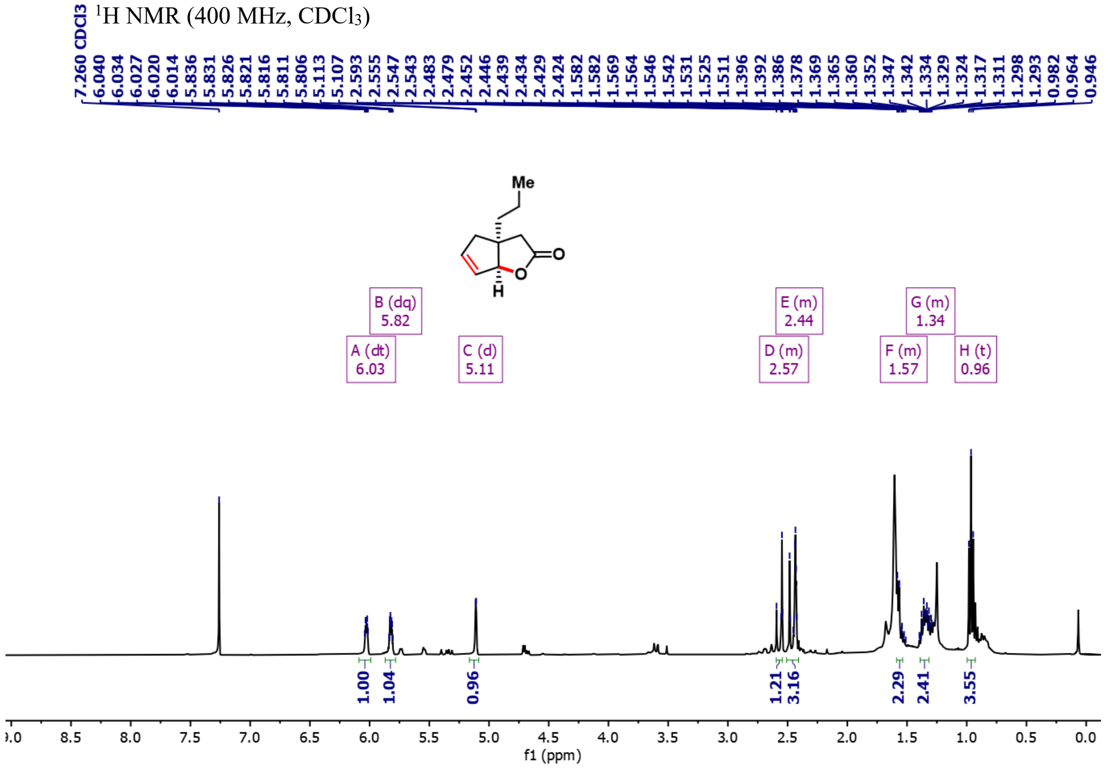

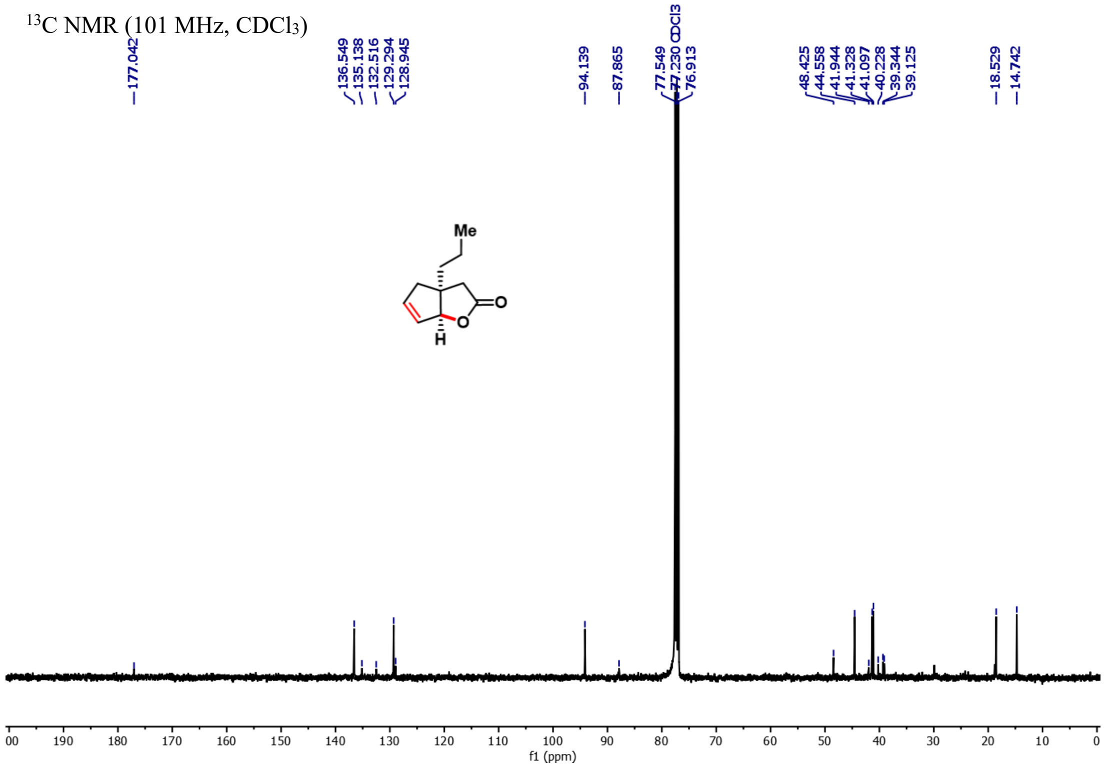

Compound 3c

(3a*S*,6a*S*)-3a-Isopropyl-3,3a,4,6a-tetrahydro-2H-cyclopenta[b]furan-2-one

^{1}H NMR (400 MHz, $CDCl_3$)

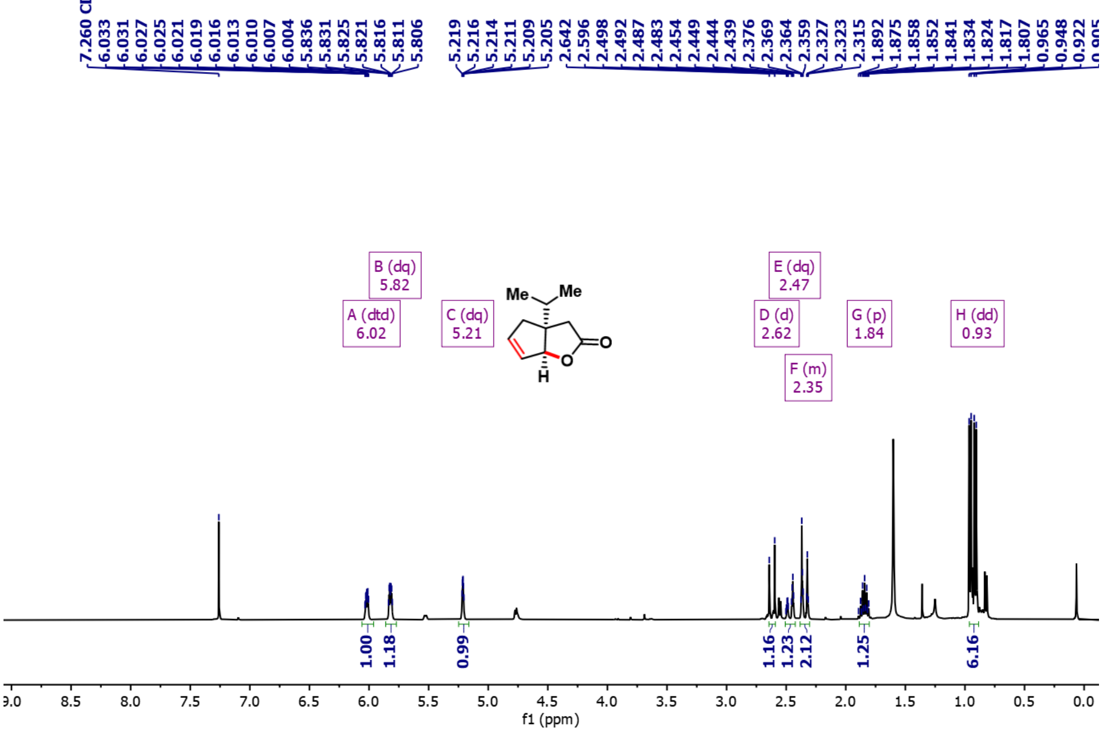

^{13}C NMR (101 MHz, $CDCl_3$)

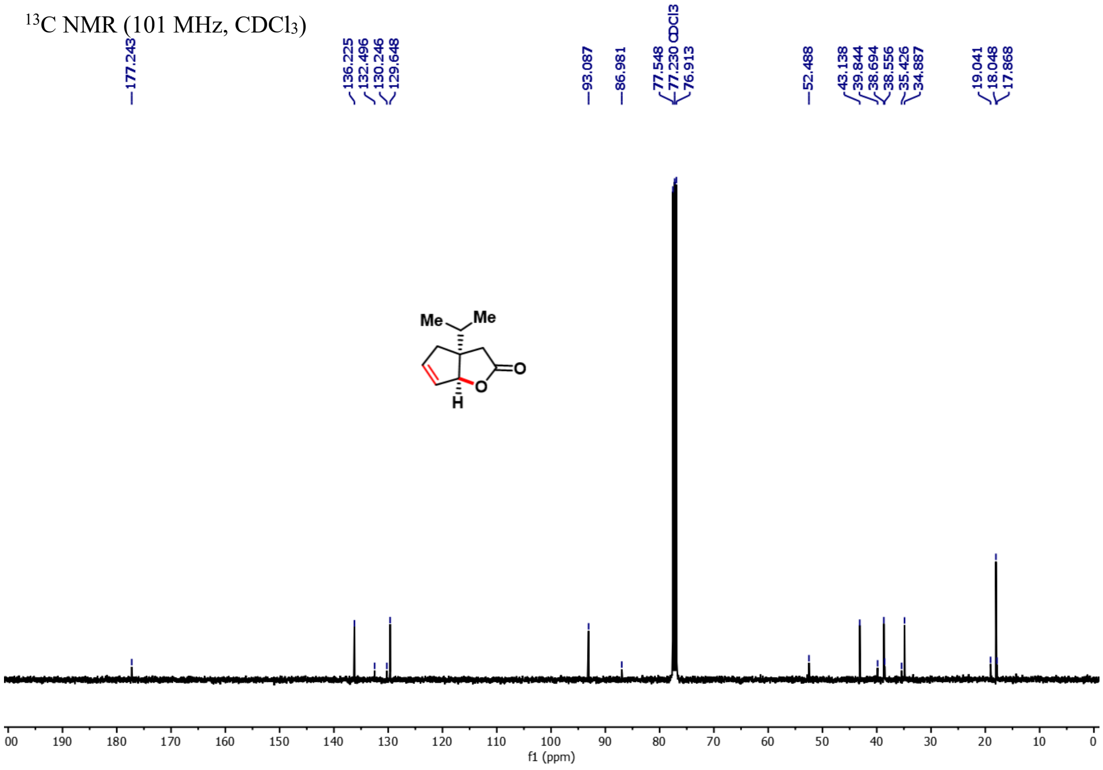

Compound 3d

(3a*S*,6a*S*)-3a-Cyclohexyl-3,3a,4,6a-tetrahydro-2H-cyclopenta[b]furan-2-one

^{1}H NMR (400 MHz, CDCl$_3$)

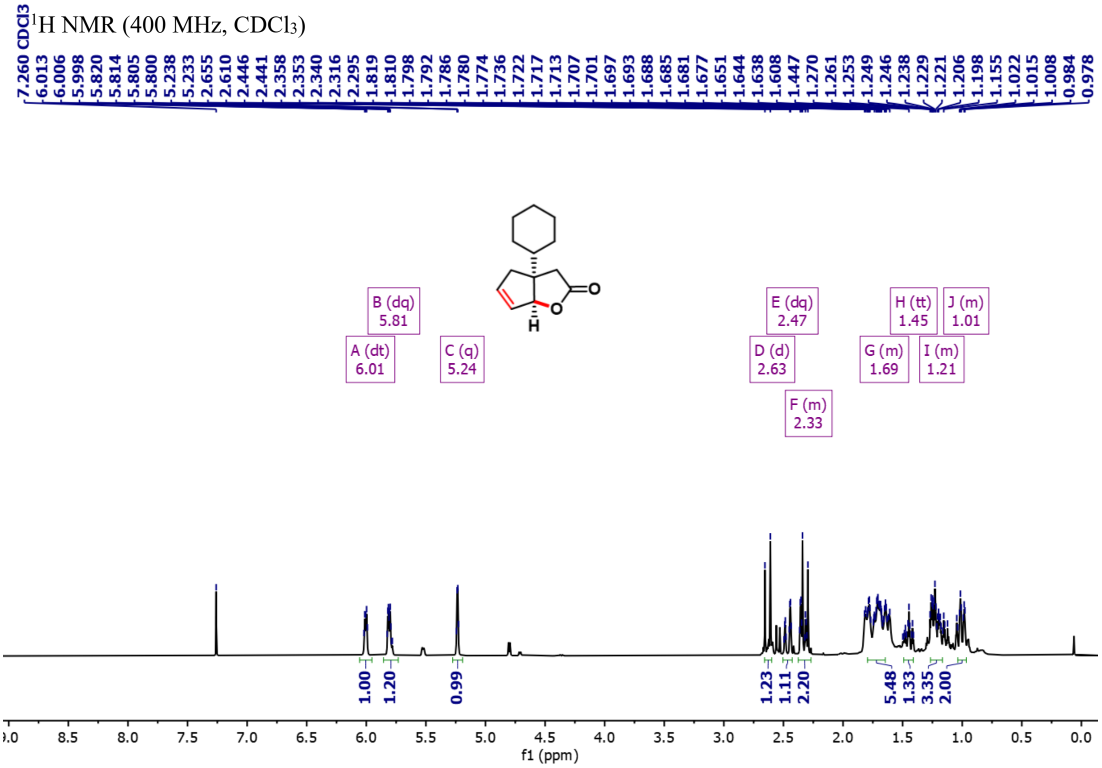

^{13}C NMR (101 MHz, CDCl$_3$)

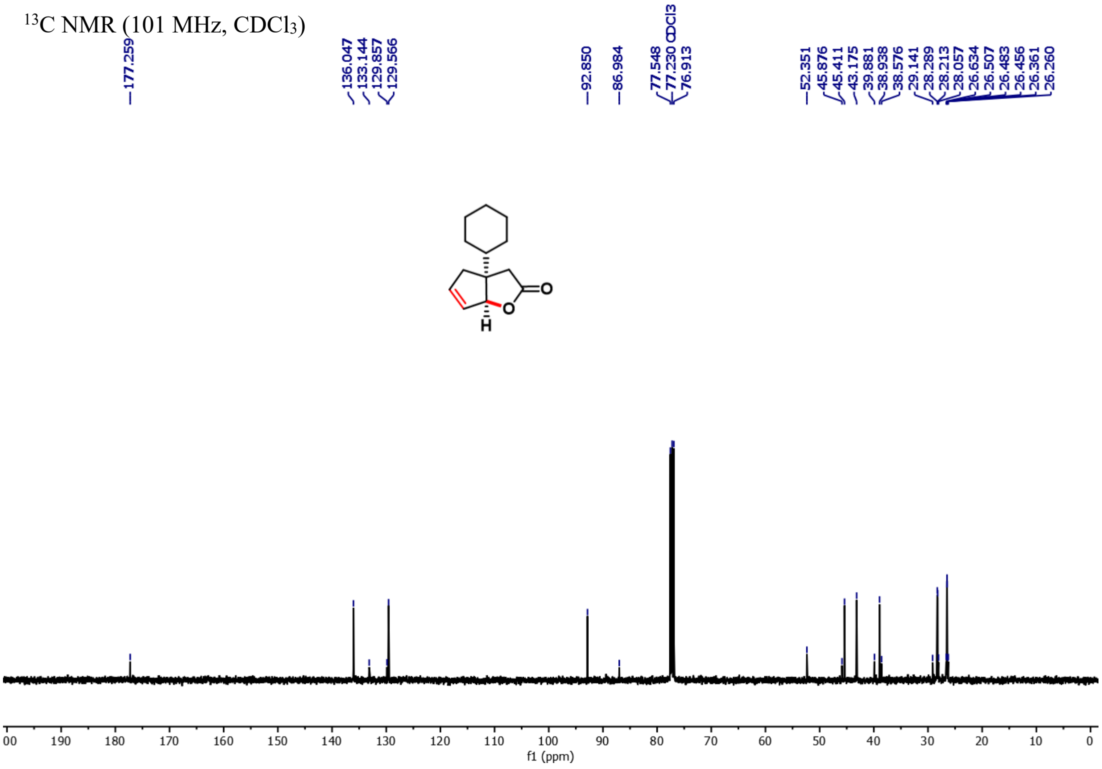

Compound 3e

(3a*S*,6a*S*)-3a,5-Dimethyl-3,3a,4,6a-tetrahydro-2H-cyclopenta[b]furan-2-one

^{1}H NMR (400 MHz, $CDCl_3$)

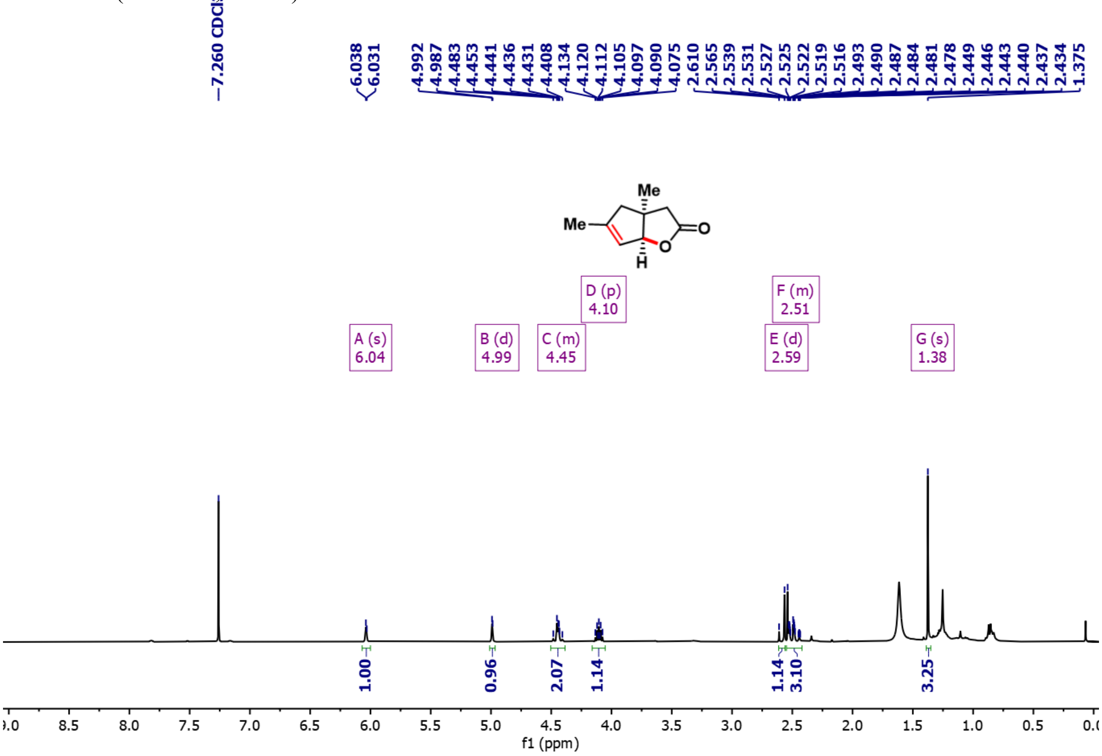

^{13}C NMR (101 MHz, $CDCl_3$)

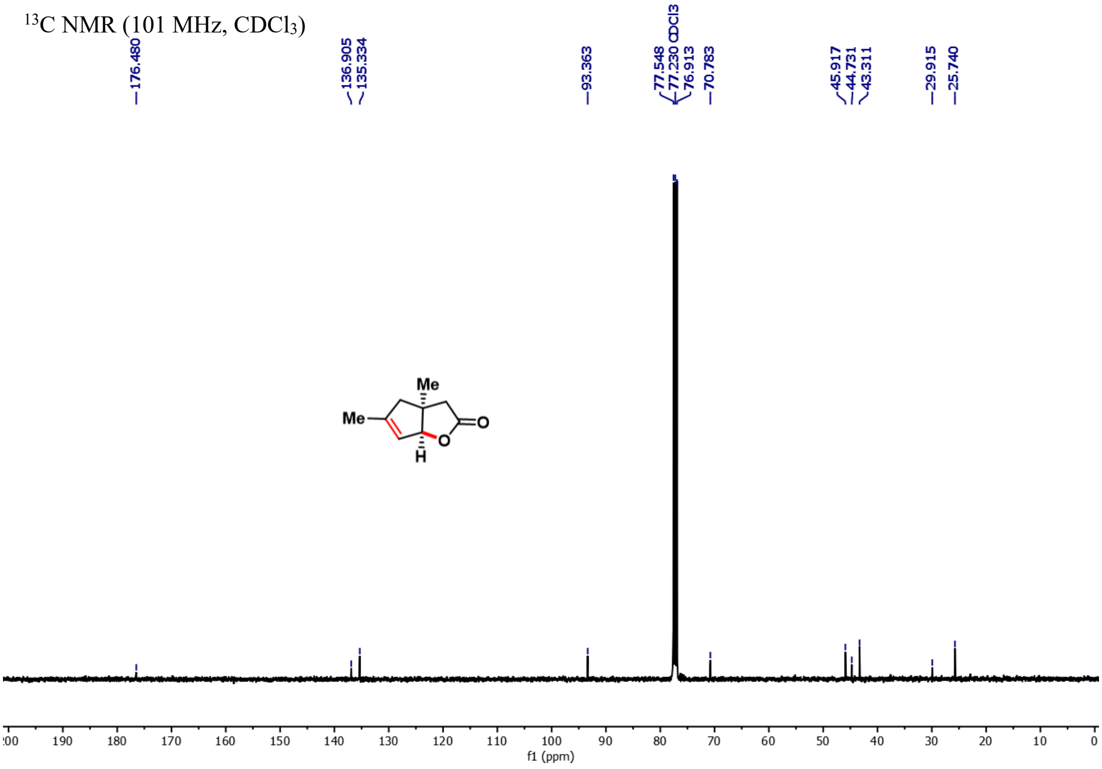

Compound 3f

(3a*S*,6a*S*)-3a-Ethyl-5-methyl-3,3a,4,6a-tetrahydro-2H-cyclopenta[b]furan-2-one

^{1}H NMR (400 MHz, $CDCl_3$)

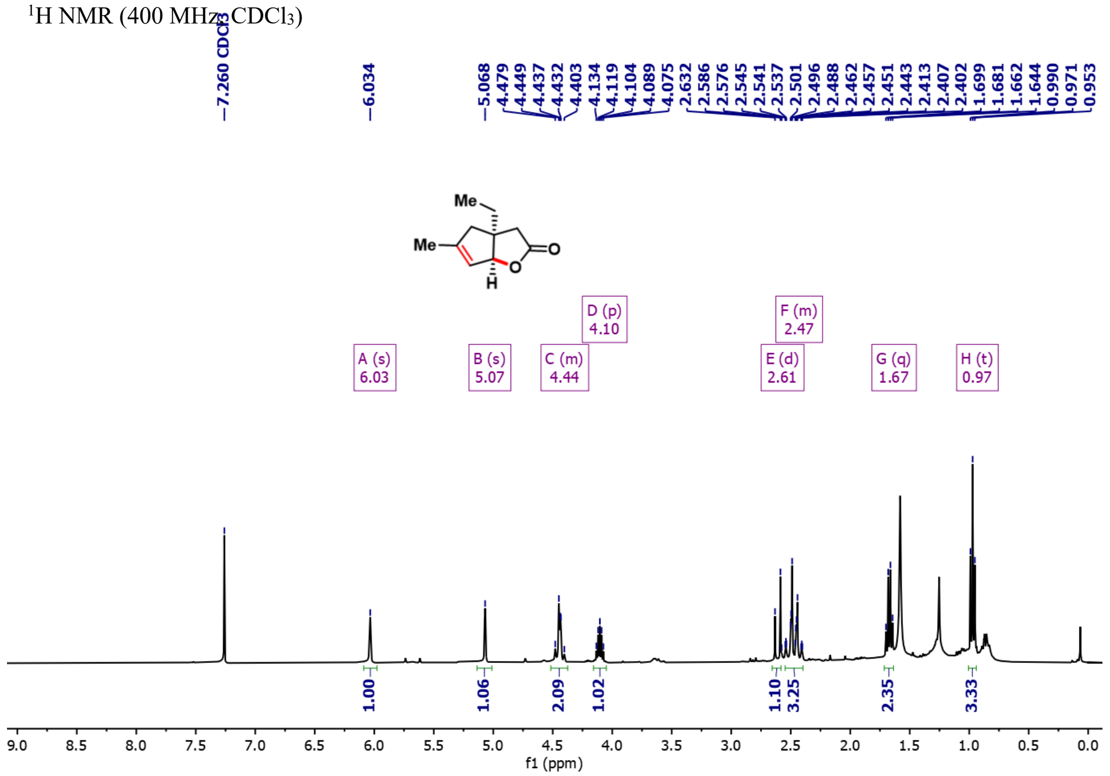

^{13}C NMR (101 MHz, $CDCl_3$)

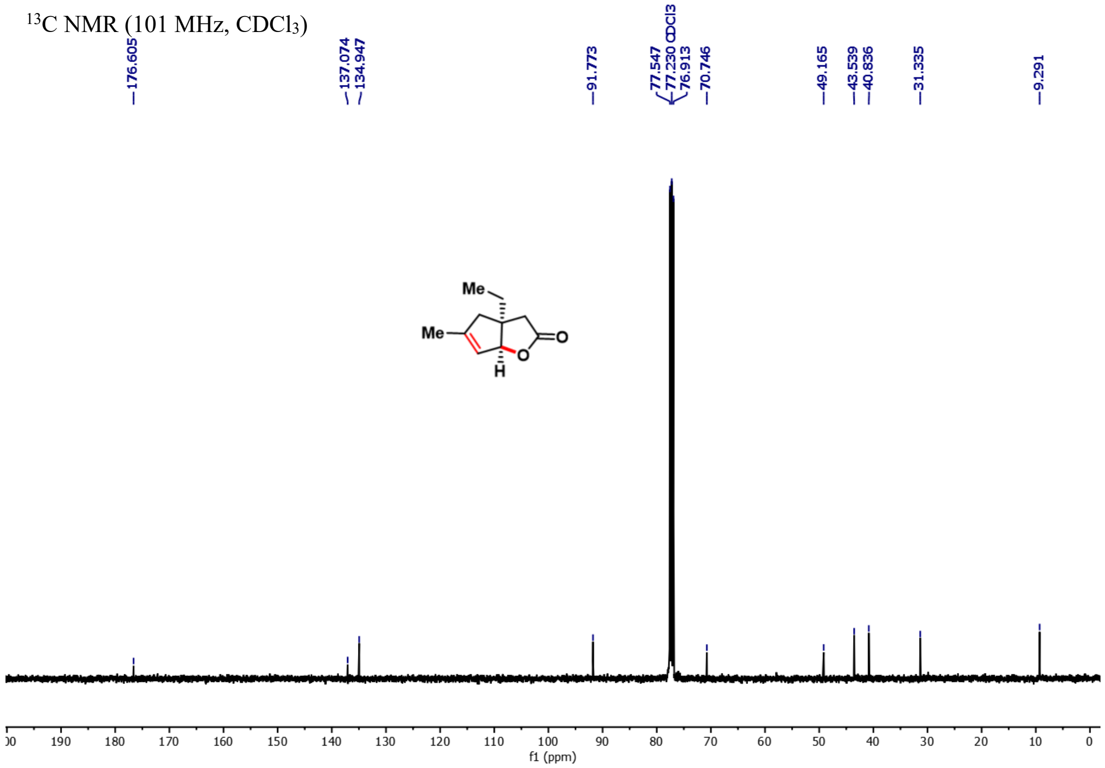

Compound 3g

(3a*S*,8a*S*)-3a-Methyl-3,3a,4,5,6,8a-hexahydro-2H-cyclohepta[b]furan-2-one

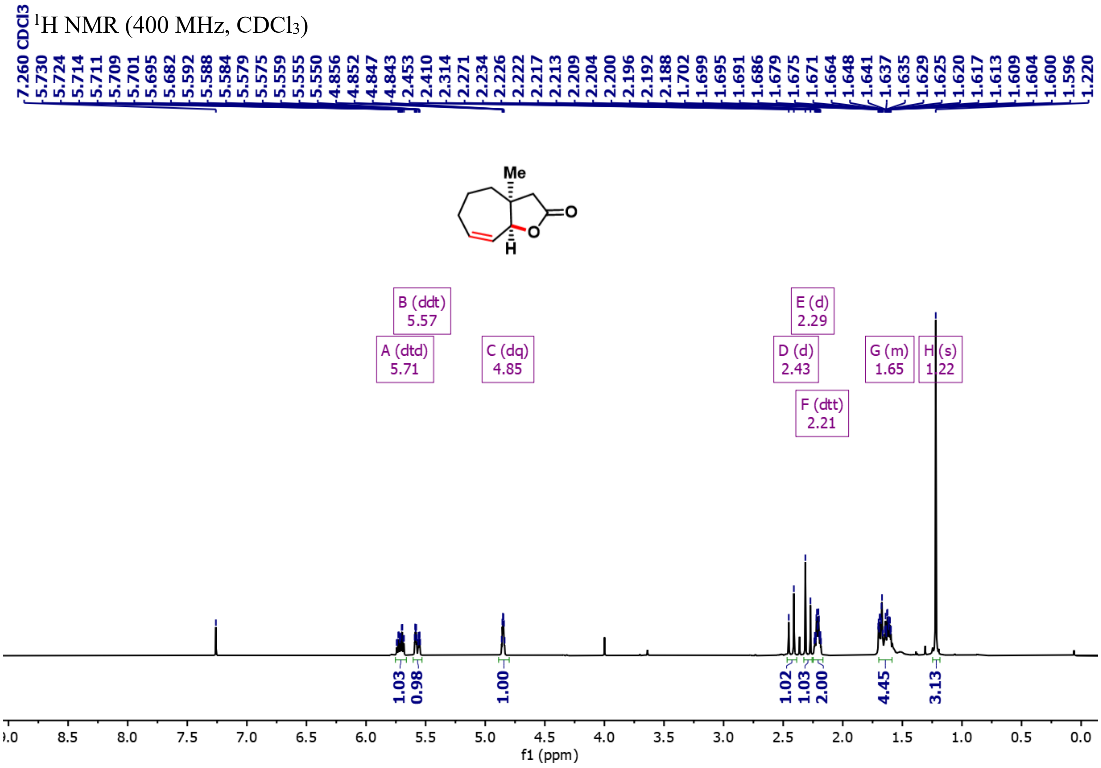

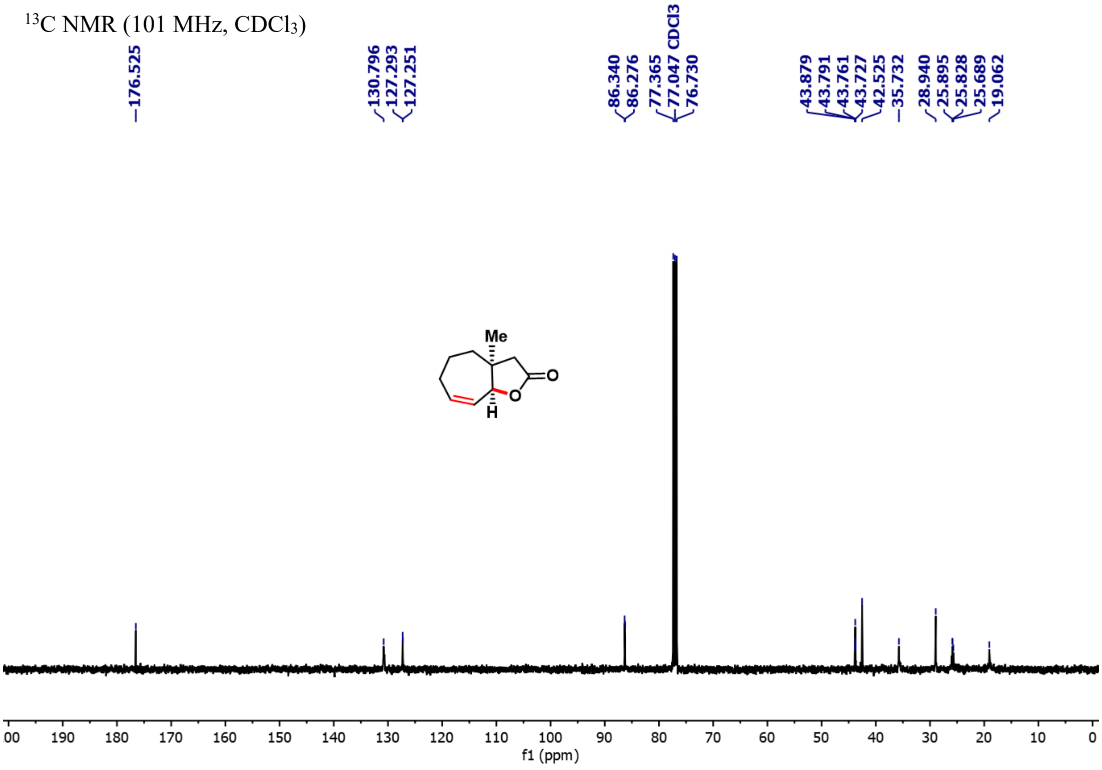

Compound 3h

(3a*S*,8a*S*)-3a-Ethyl-3,3a,4,5,6,8a-hexahydro-2H-cyclohepta[b]furan-2-one

^{1}H NMR (400 MHz, $CDCl_3$)

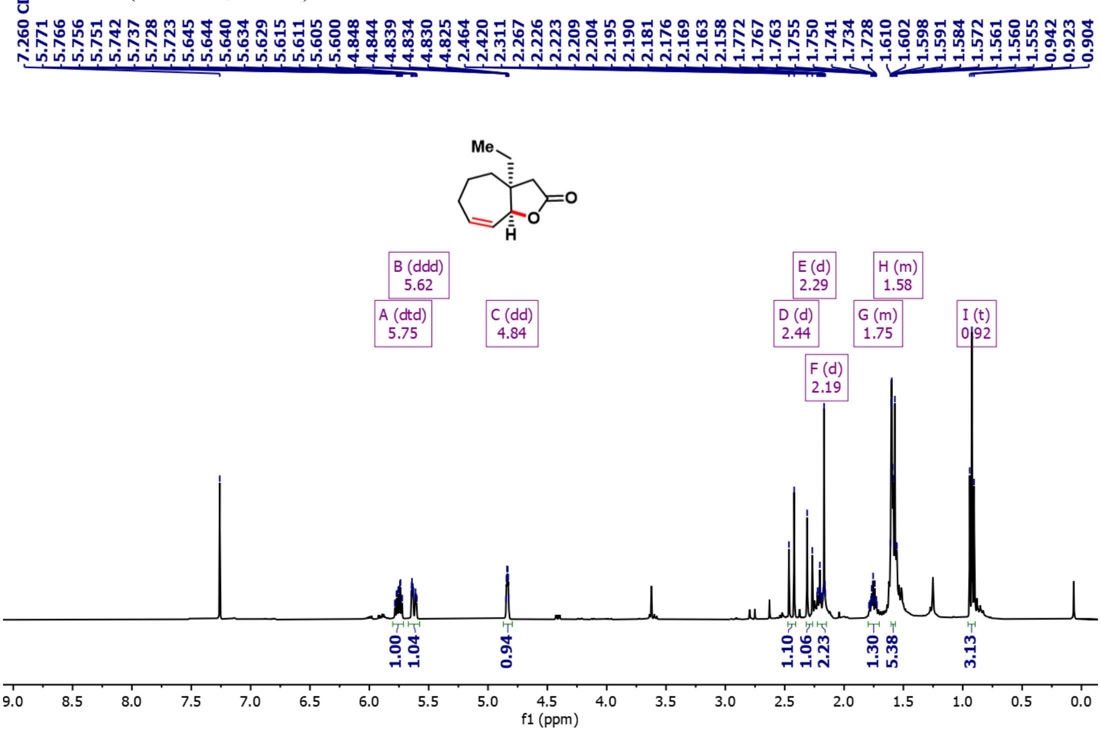

^{13}C NMR (101 MHz, $CDCl_3$)

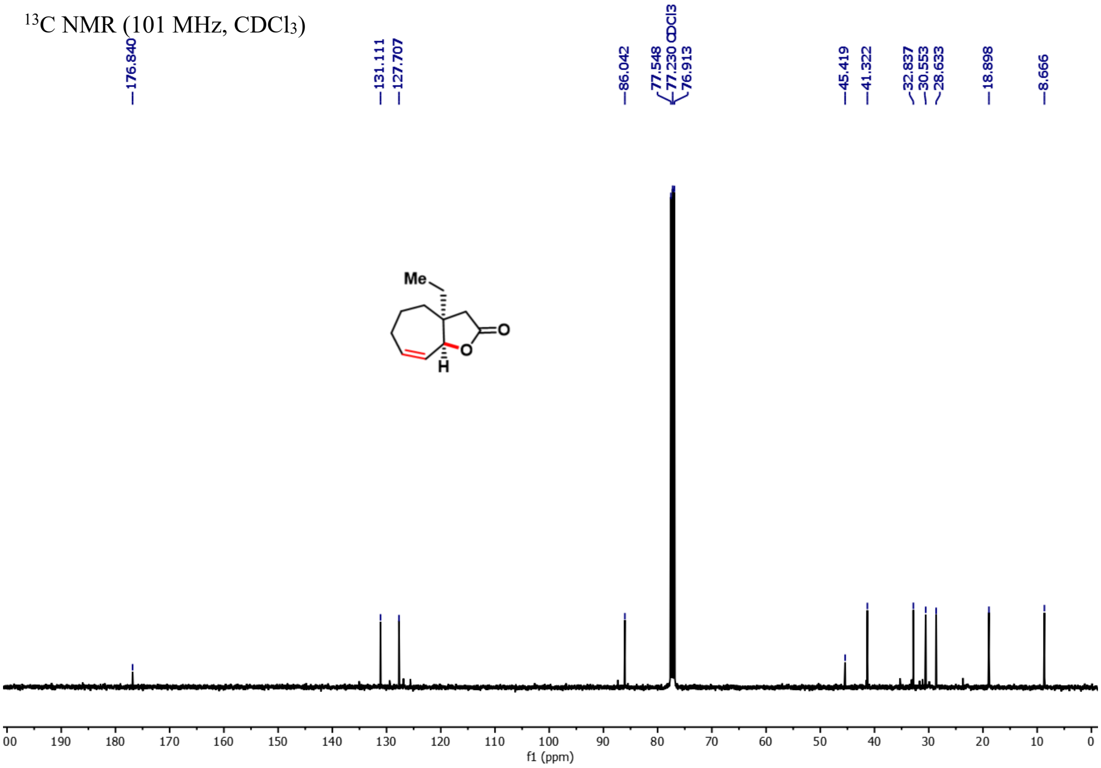

Compound 3i

(3a*S*,8a*S*)-3a-Isopropyl-3,3a,4,5,6,8a-hexahydro-2H-cyclohepta[b]furan-2-one

^{1}H NMR (400 MHz, $CDCl_3$)

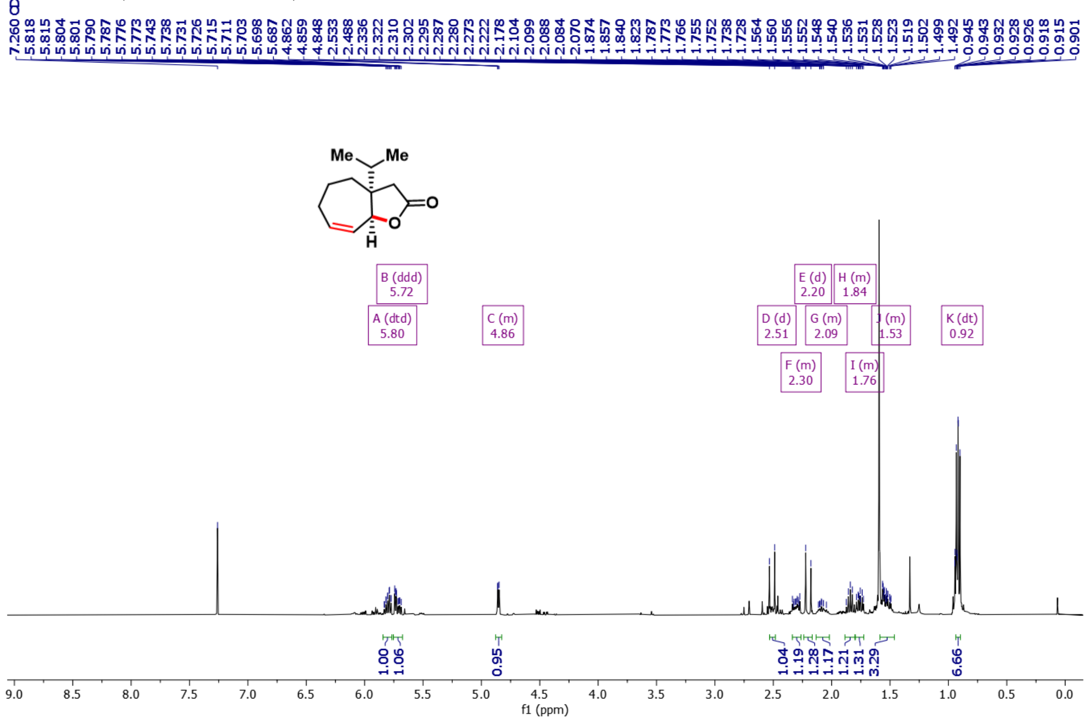

^{13}C NMR (101 MHz, $CDCl_3$)

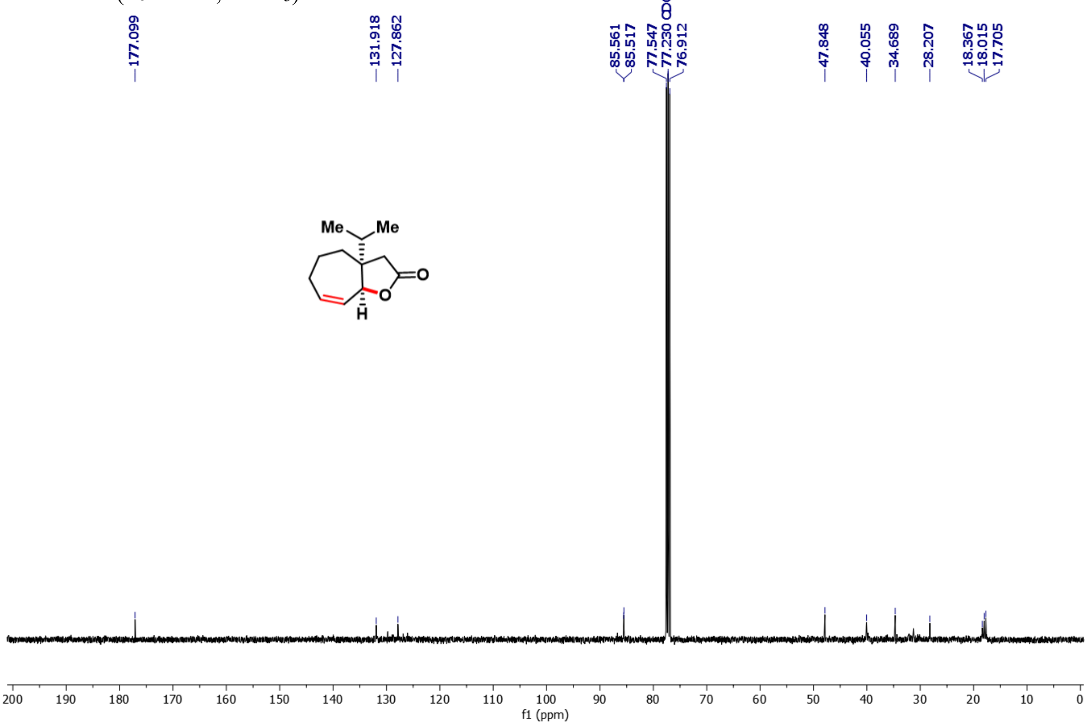

Compound 3j

(3a*R*,8a*S*)-3a-Isobutyl-3,3a,4,5,6,8a-hexahydro-2H-cyclohepta[b]furan-2-one

^{1}H NMR (400 MHz, $CDCl_3$)

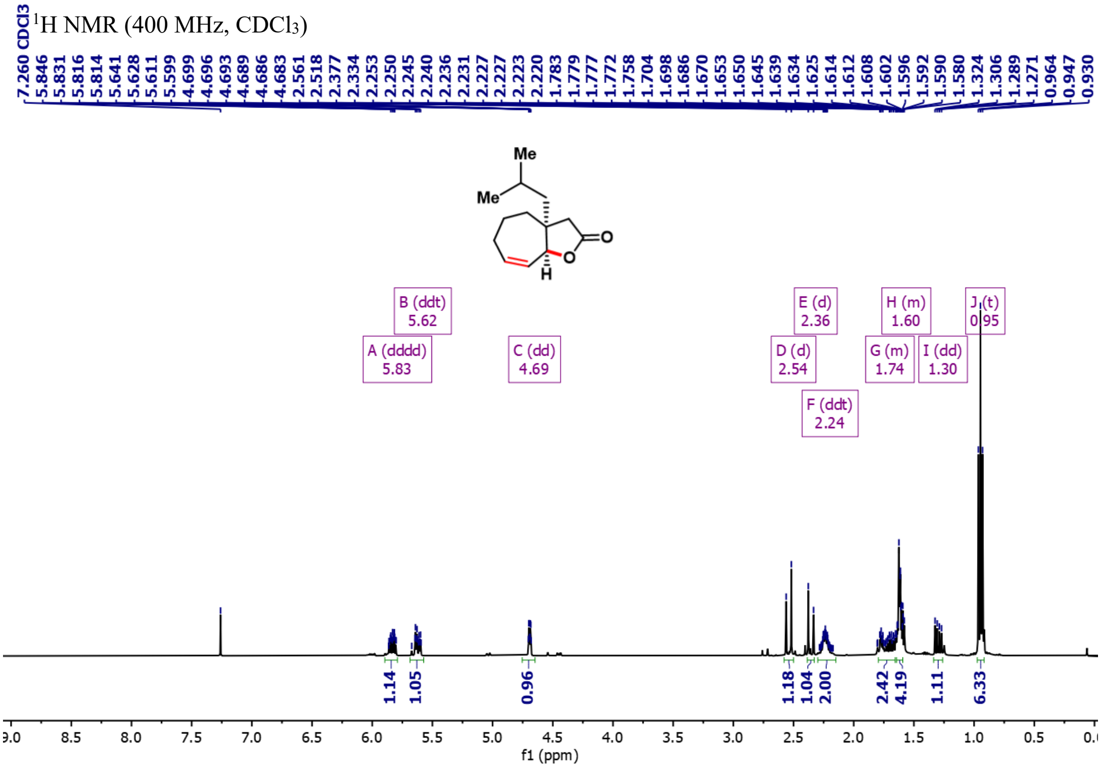

^{13}C NMR (101 MHz, $CDCl_3$)

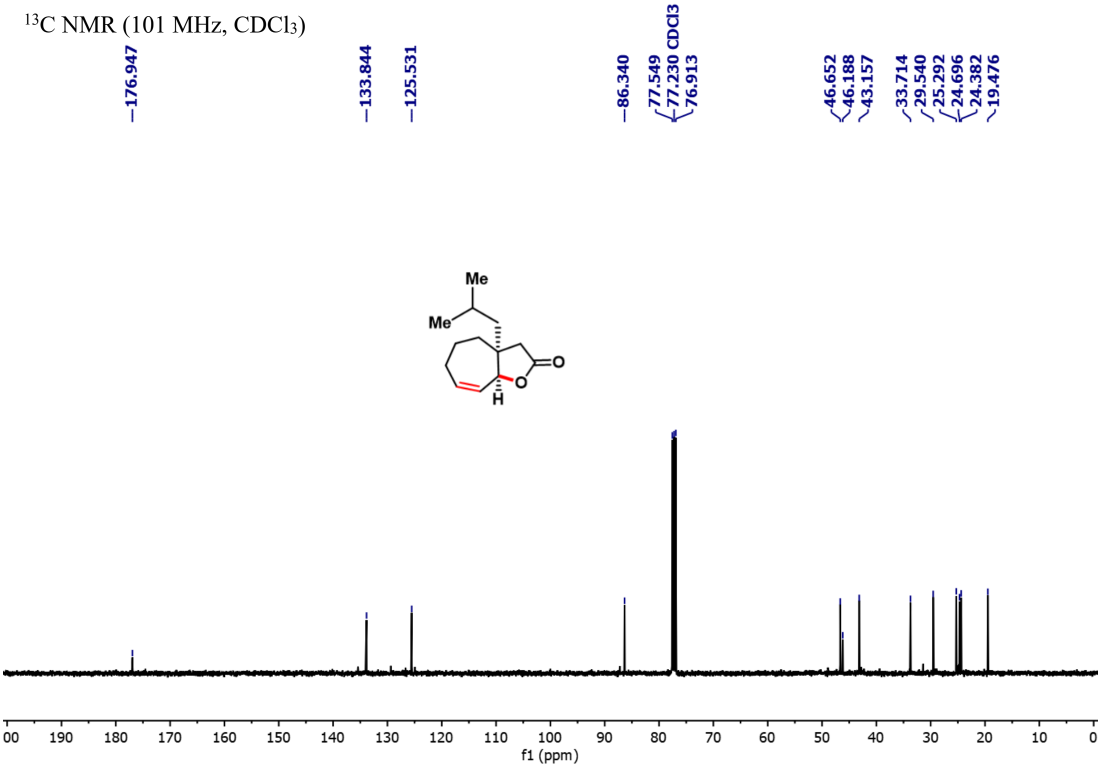

Compound 3k

(3a*S*,8a*S*)-3a-Cyclohexyl-3,3a,4,5,6,8a-hexahydro-2H-cyclohepta[b]furan-2-one

^{1}H NMR (400 MHz, $CDCl_3$)

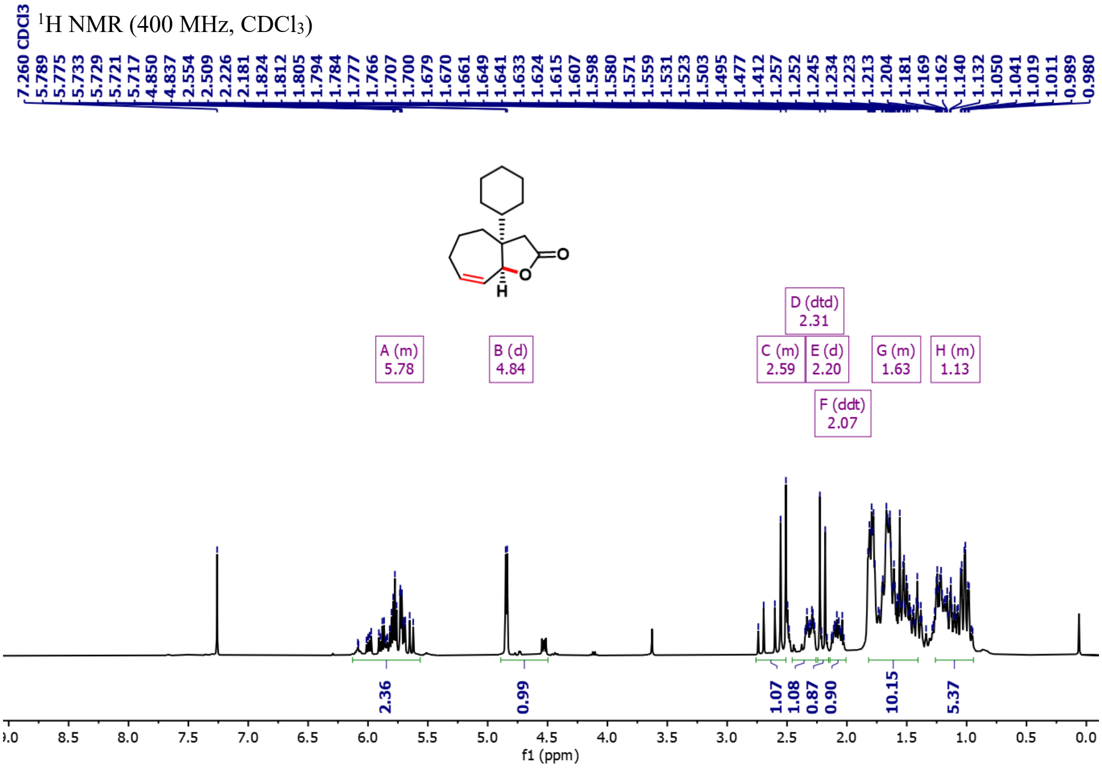

^{13}C NMR (101 MHz, $CDCl_3$)

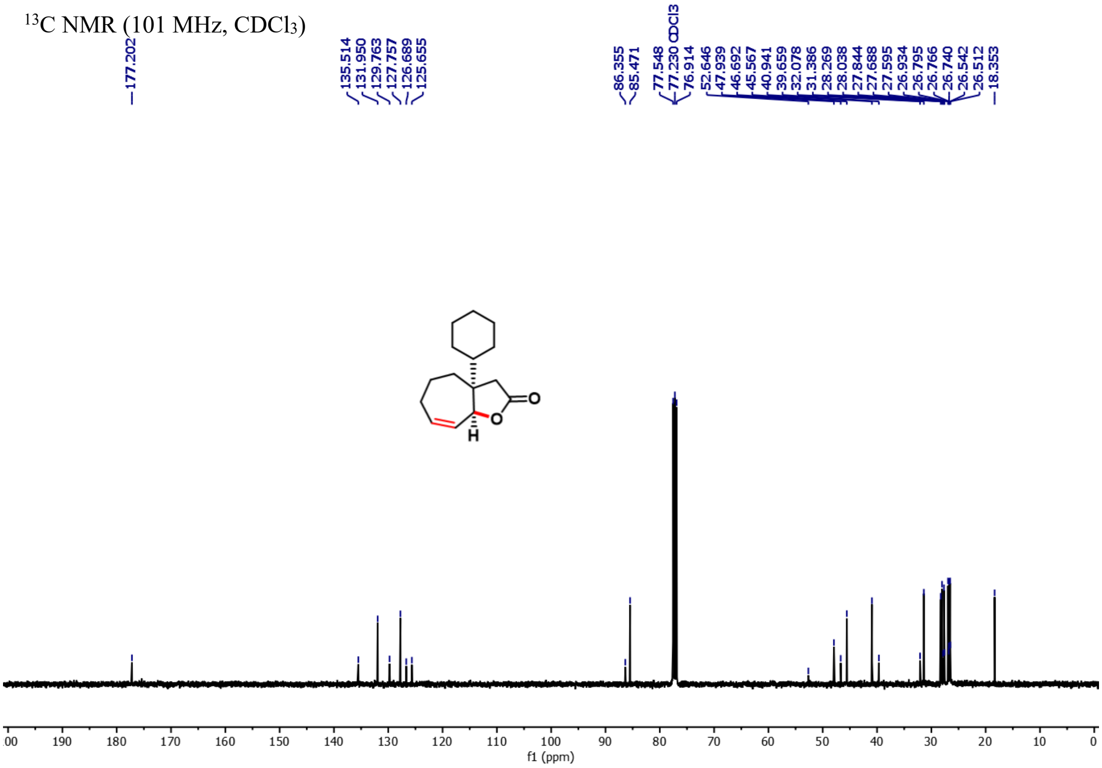

Compound 3l

(3a*S*,8a*S*)-3a-Methyl-6-(*p*-tolyl)-3,3a,4,5,6,8a-hexahydro-2H-cyclohepta[b]furan-2-one

^{1}H NMR (400 MHz, CDCl$_3$)

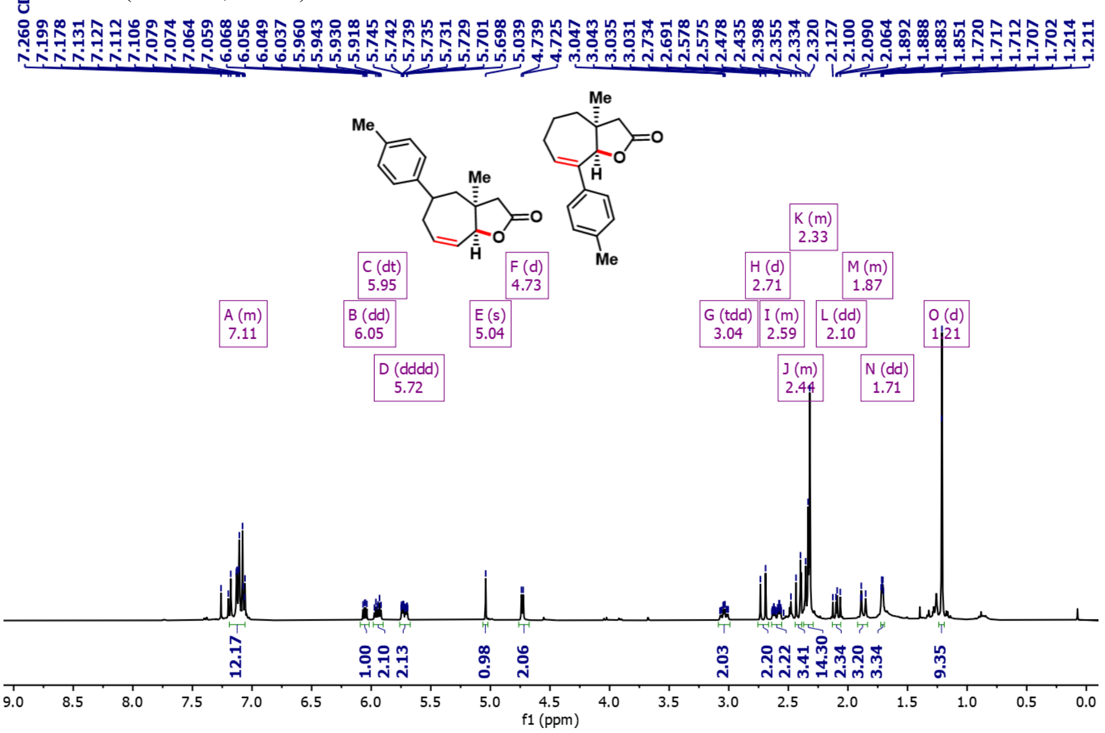

^{13}C NMR (101 MHz, CDCl$_3$)

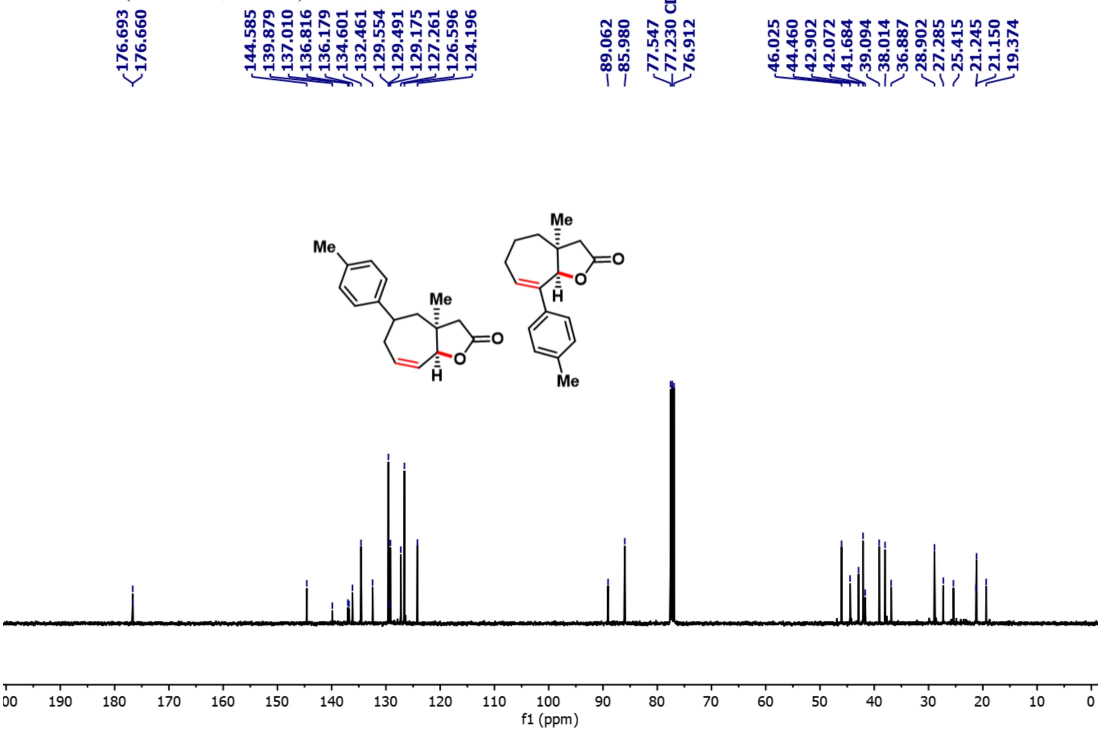

Compound 3m

(3a*S*,13a*S*,*Z*)-3a-Methyl-3a,4,5,6,7,8,9,10,11,13a-decahydrocyclododeca[b]furan-2(3H)-one

^{1}H NMR (400 MHz, $CDCl_3$)

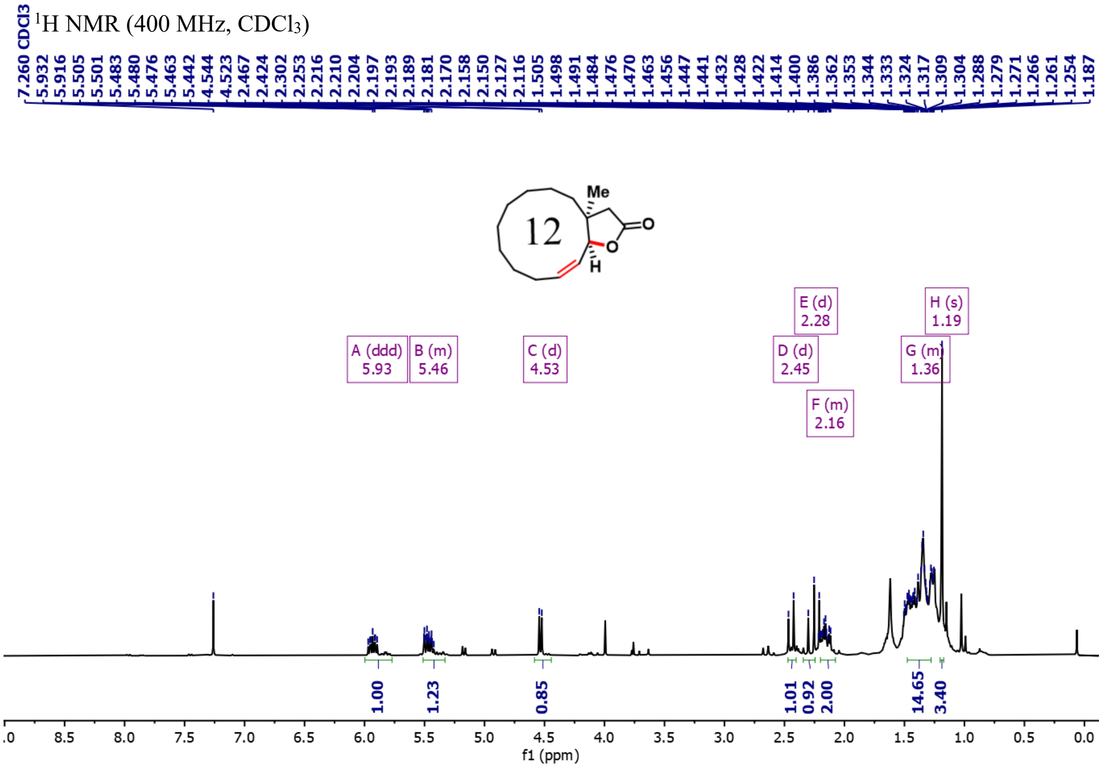

^{13}C NMR (101 MHz, $CDCl_3$)

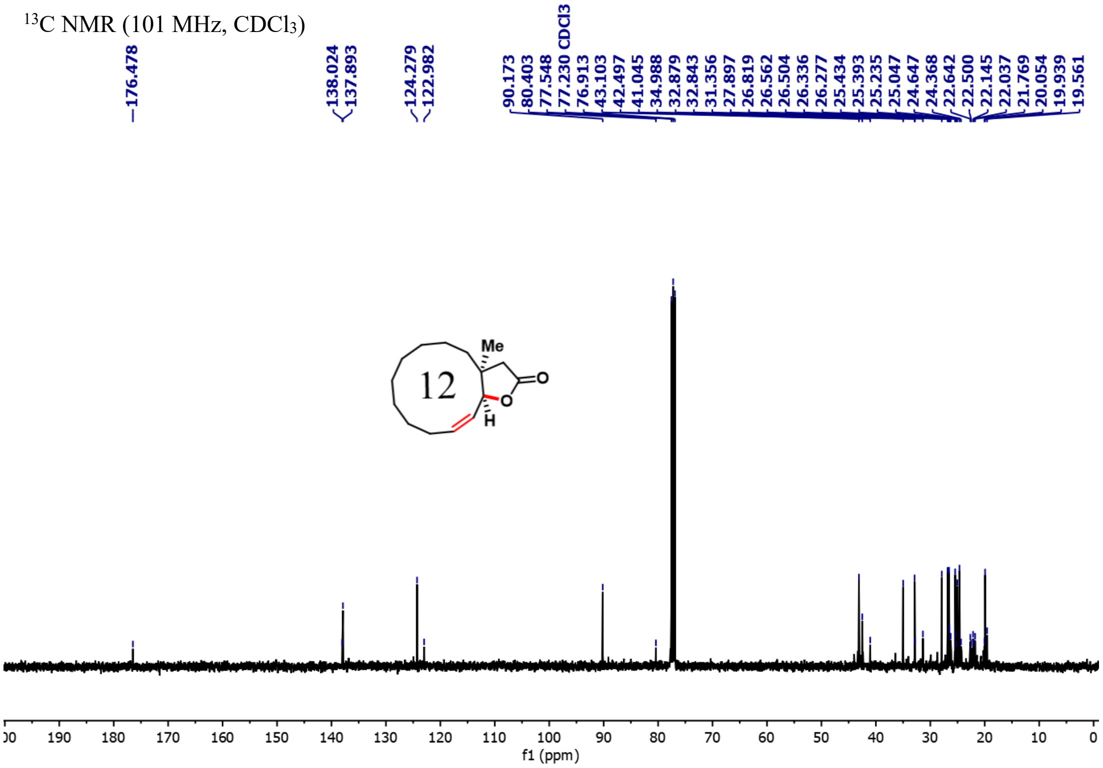

Compound 3n

(3a*S*,13a*S*,*Z*)-3a-Ethyl-3a,4,5,6,7,8,9,10,11,13a-decahydrocyclododeca[b]furan-2(3H)-one

^{1}H NMR (400 MHz, $CDCl_3$)

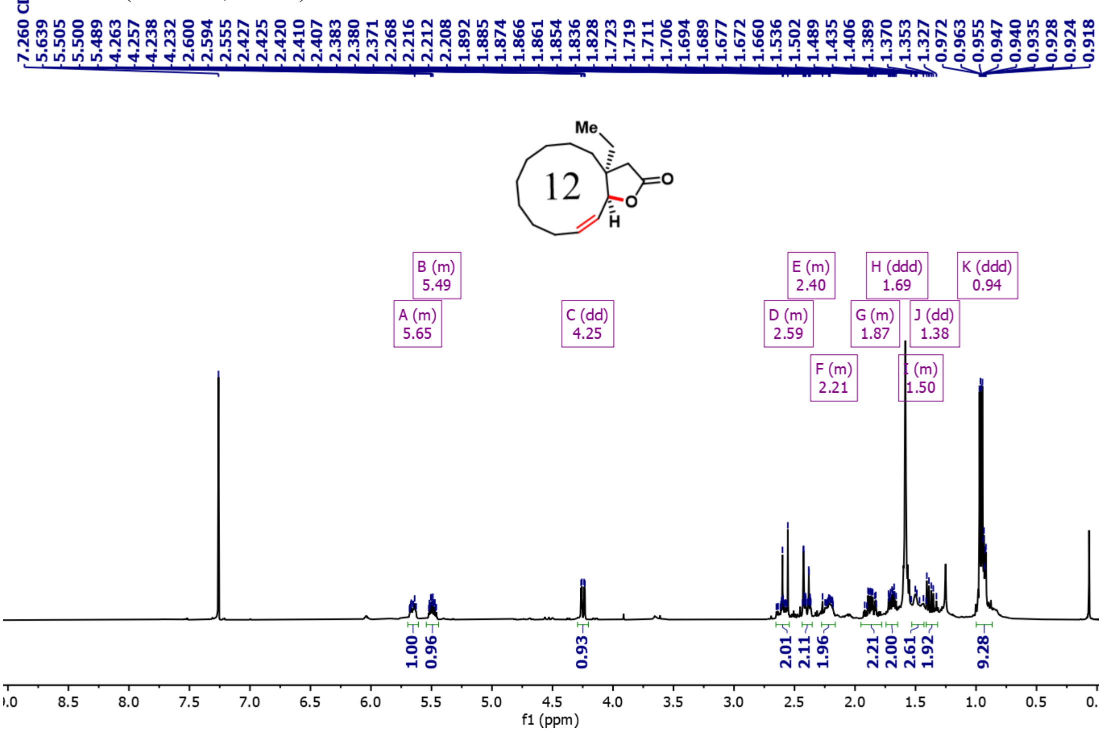

^{13}C NMR (101 MHz, $CDCl_3$)

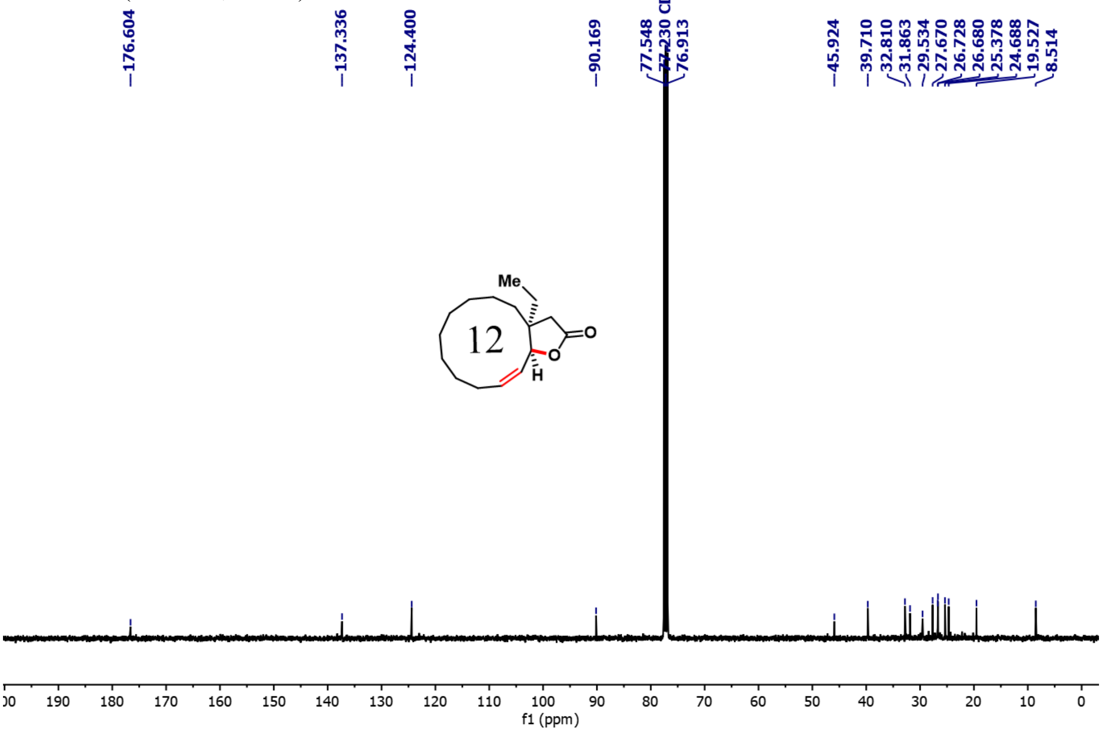

Compound 3o

(3a*R*,16a*S*,*E*)-3a-Methyl-3,3a,4,5,6,7,8,9,10,11,12,13,14,16a-tetradecahydro-2H-cyclopentadeca[b]furan-2-one

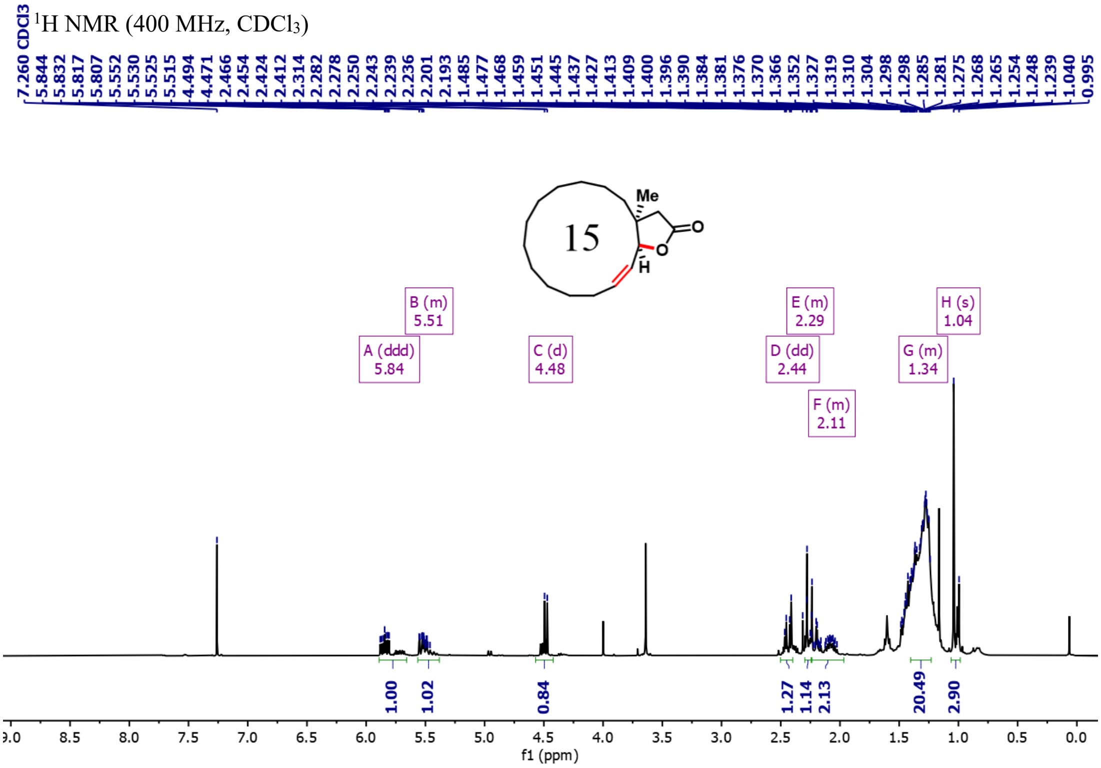

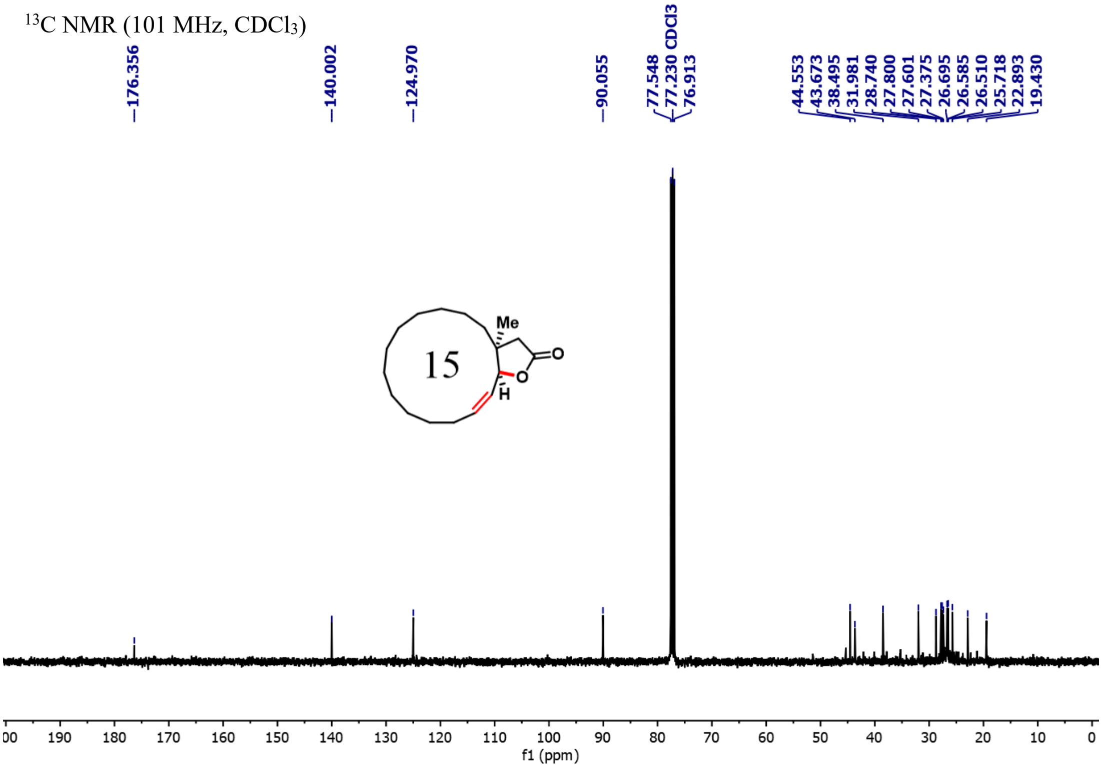

Compound 3p

(3a*S*,16a*S*,*E*)-3a-Ethyl-3,3a,4,5,6,7,8,9,10,11,12,13,14,16a-tetradecahydro-2H-cyclopentadeca[b]furan-2-one

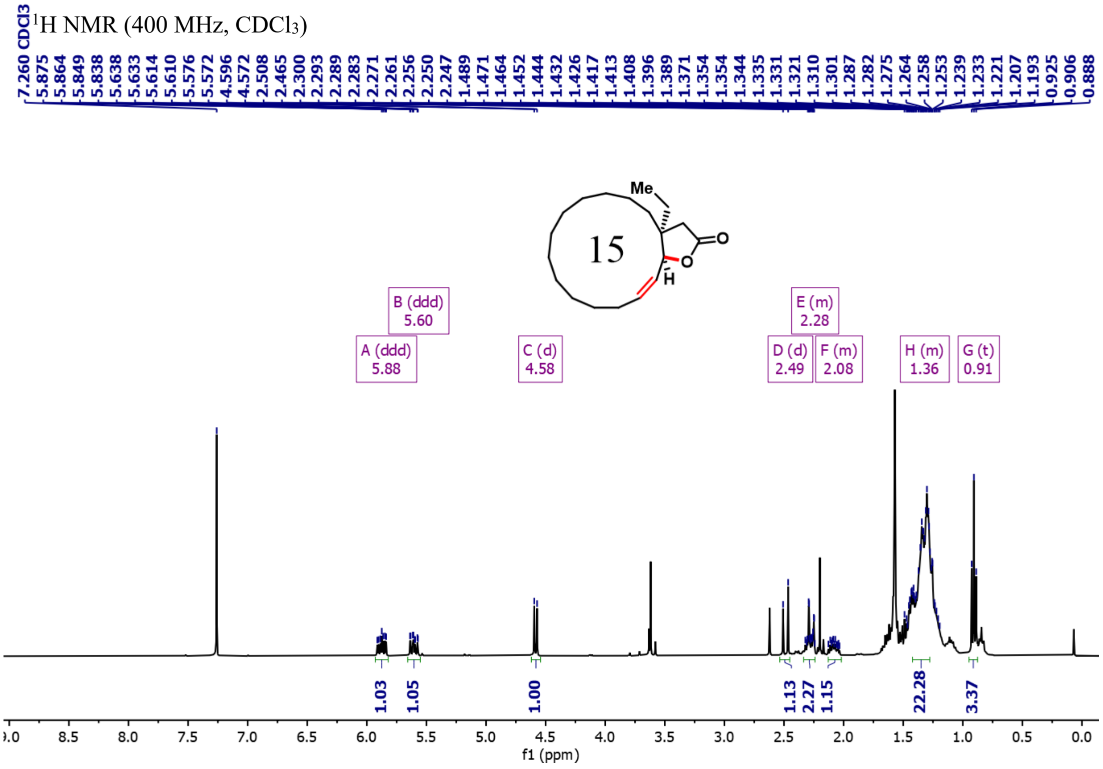

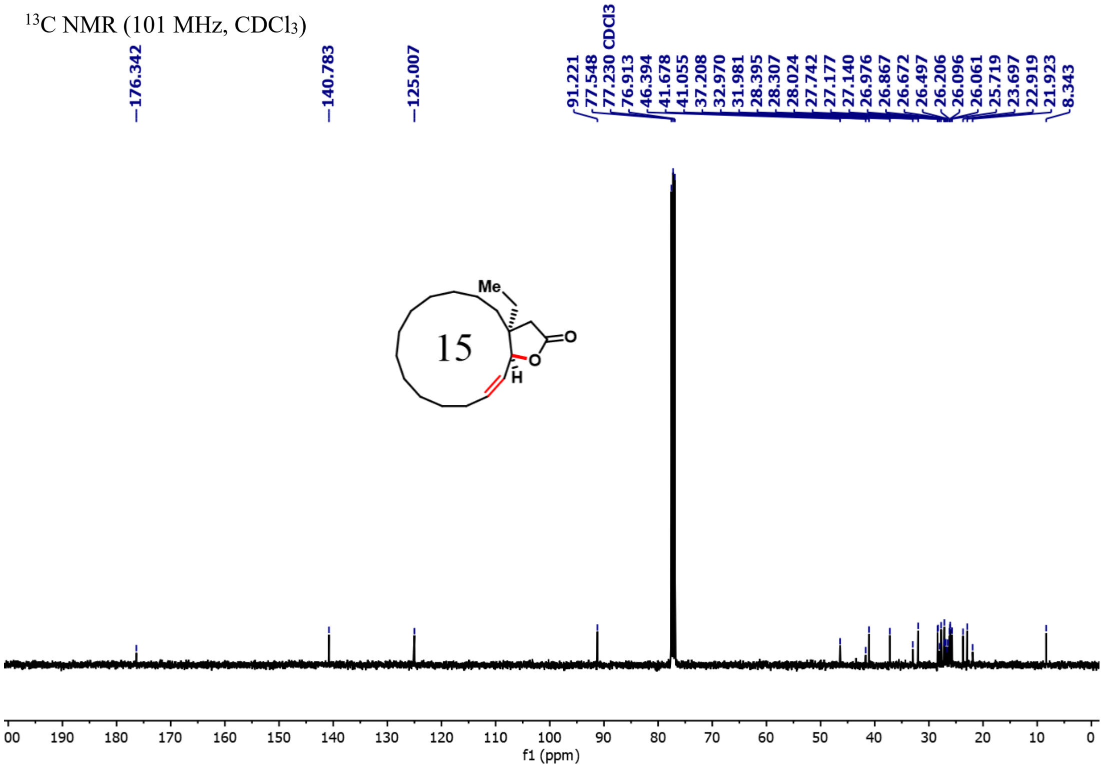

Compound 3q

(3a*S*,16a*S*,*E*)-3a,5-Dimethyl-3,3a,4,5,6,7,8,9,10,11,12,13,14,16a-tetradecahydro-2H-cyclopentadeca[b]furan-2-one

^{1}H NMR (400 MHz, $CDCl_3$)

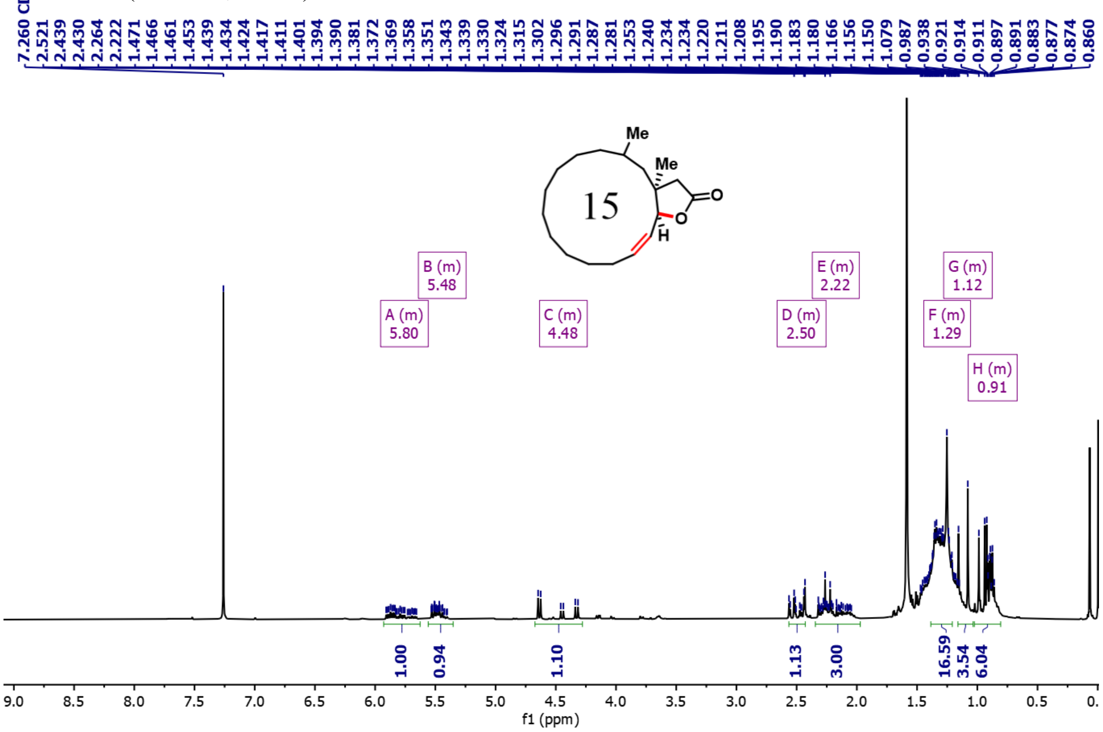

^{13}C NMR (126 MHz, $CDCl_3$)

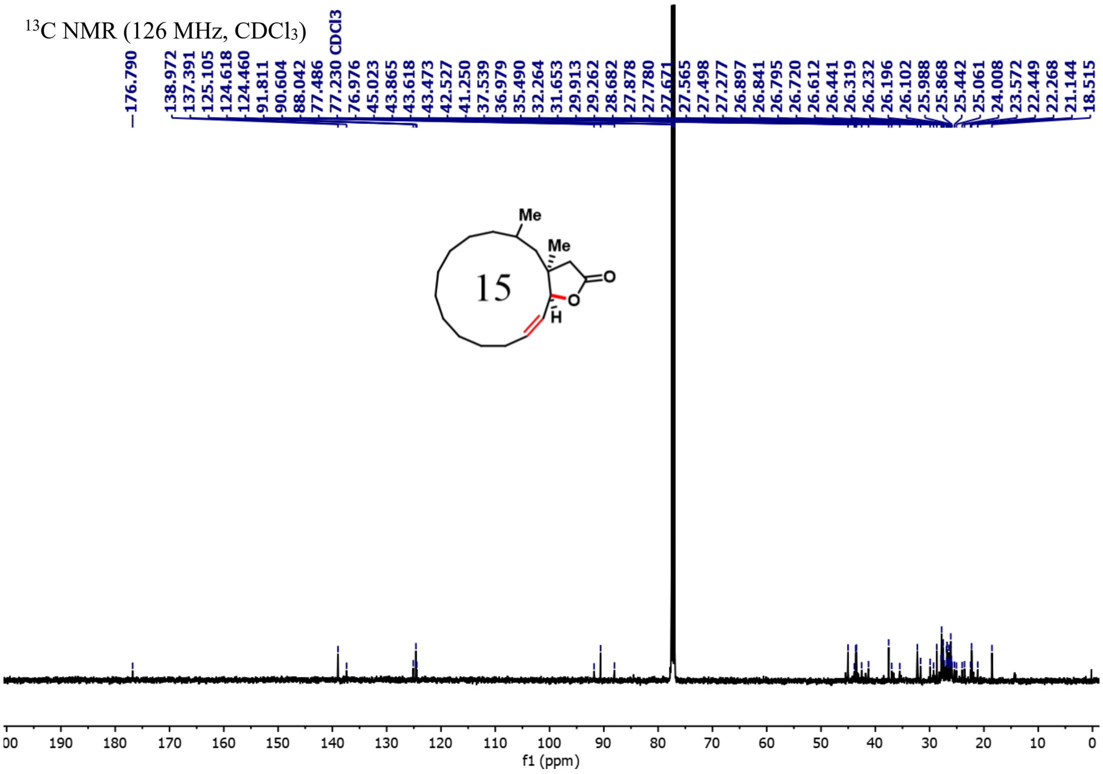

Compound 4a

Ethyl (*E*)-3-((3a*S*,7a*R*)-3a-methyl-2-oxo-3,3a,4,5-tetrahydrobenzofuran-7a(2H)-yl)acrylate

^{1}H NMR (400 MHz, $CDCl_3$)

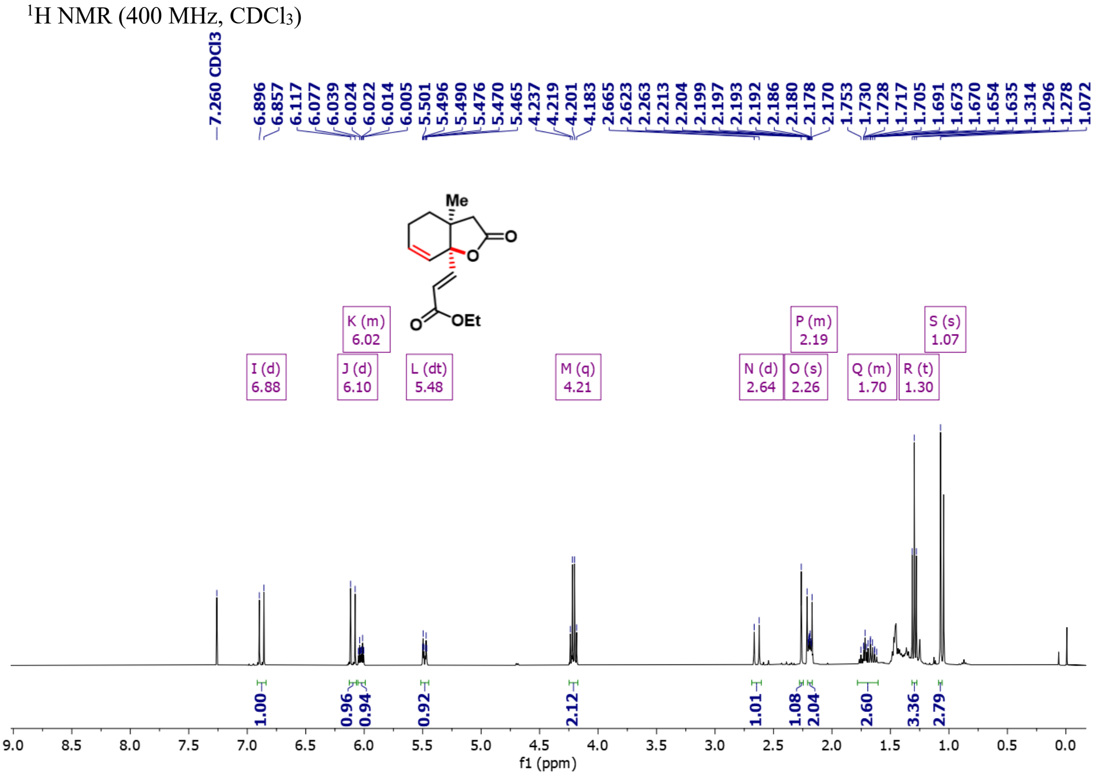

^{13}C NMR (126 MHz, $CDCl_3$)

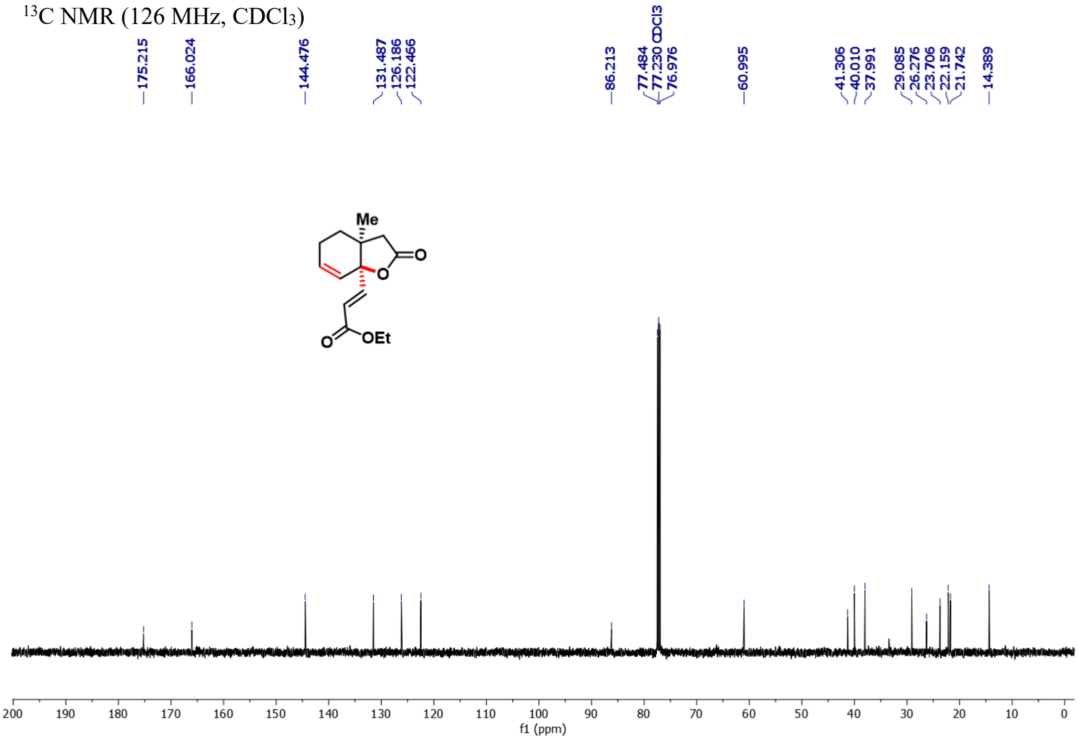

NOE experiment:

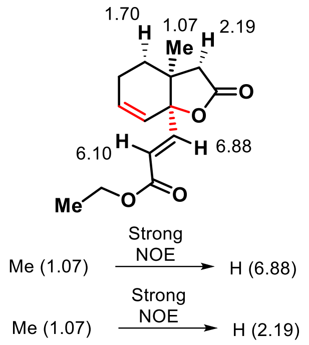

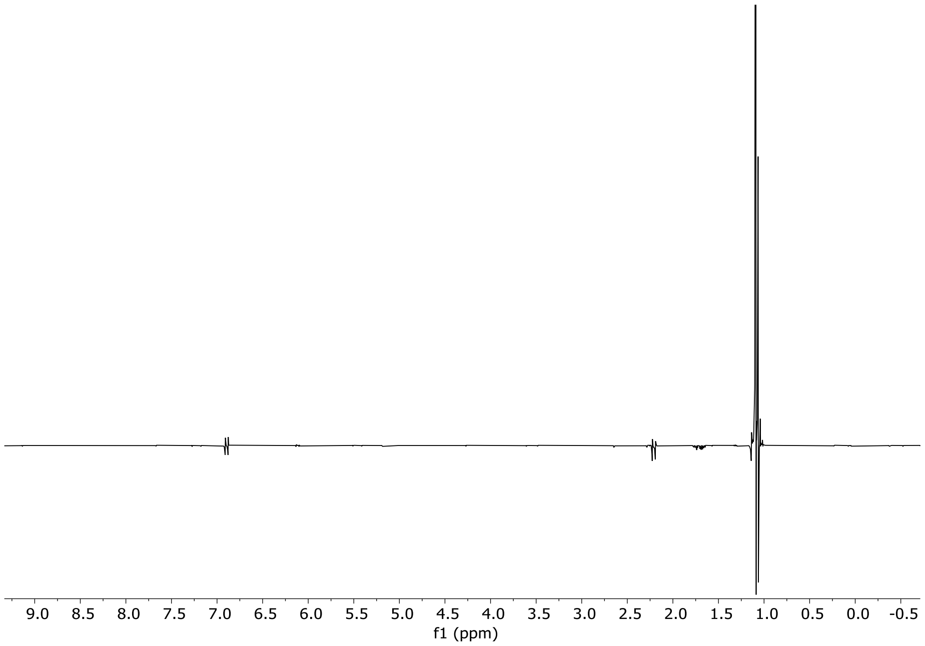

COSY Experiment:

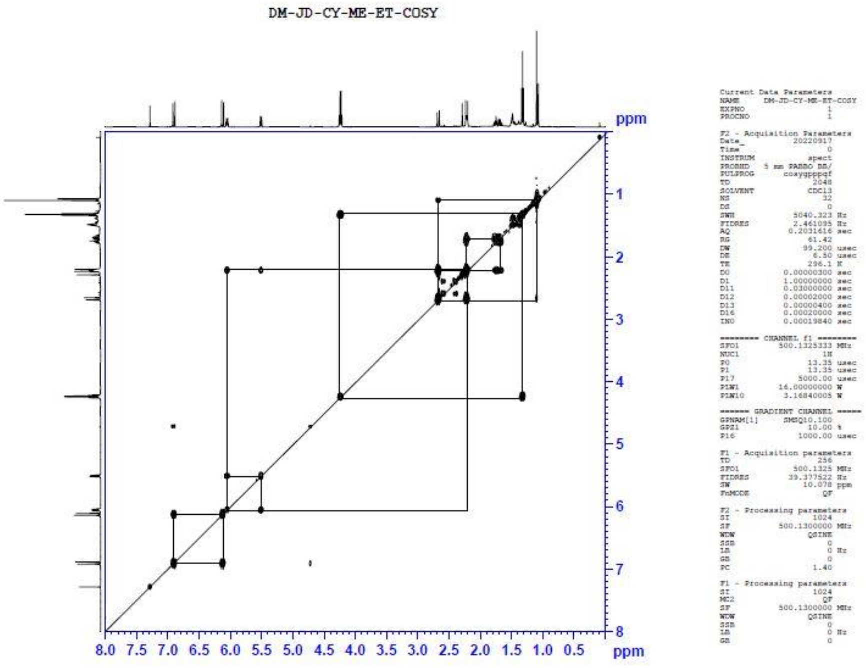

NOESY Experiment:

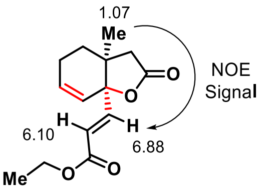

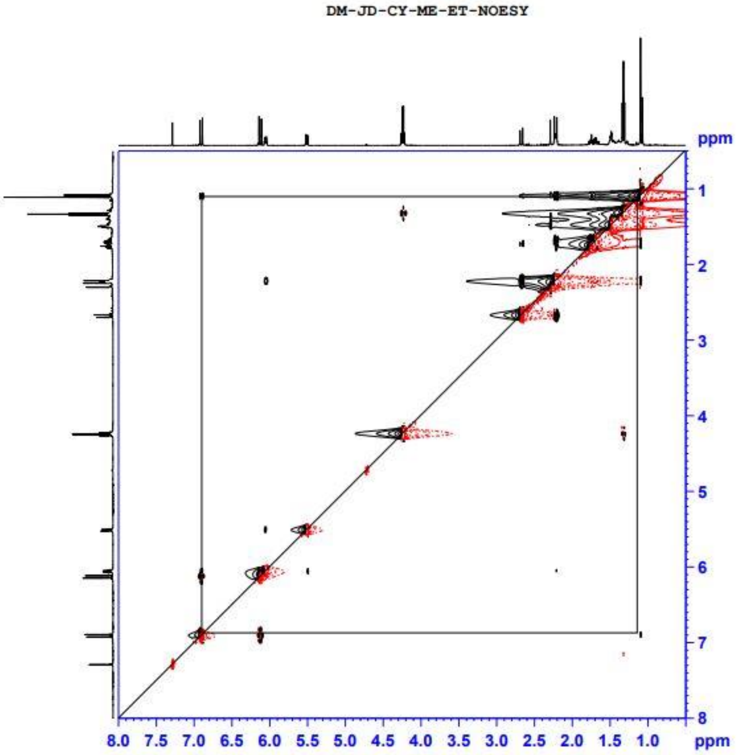

Compound 4b

Ethyl (*E*)-3-((3a*S*,7a*R*)-3a-ethyl-2-oxo-3,3a,4,5-tetrahydrobenzofuran-7a(2H)-yl)acrylate

^{1}H NMR (400 MHz, CDCl$_3$)

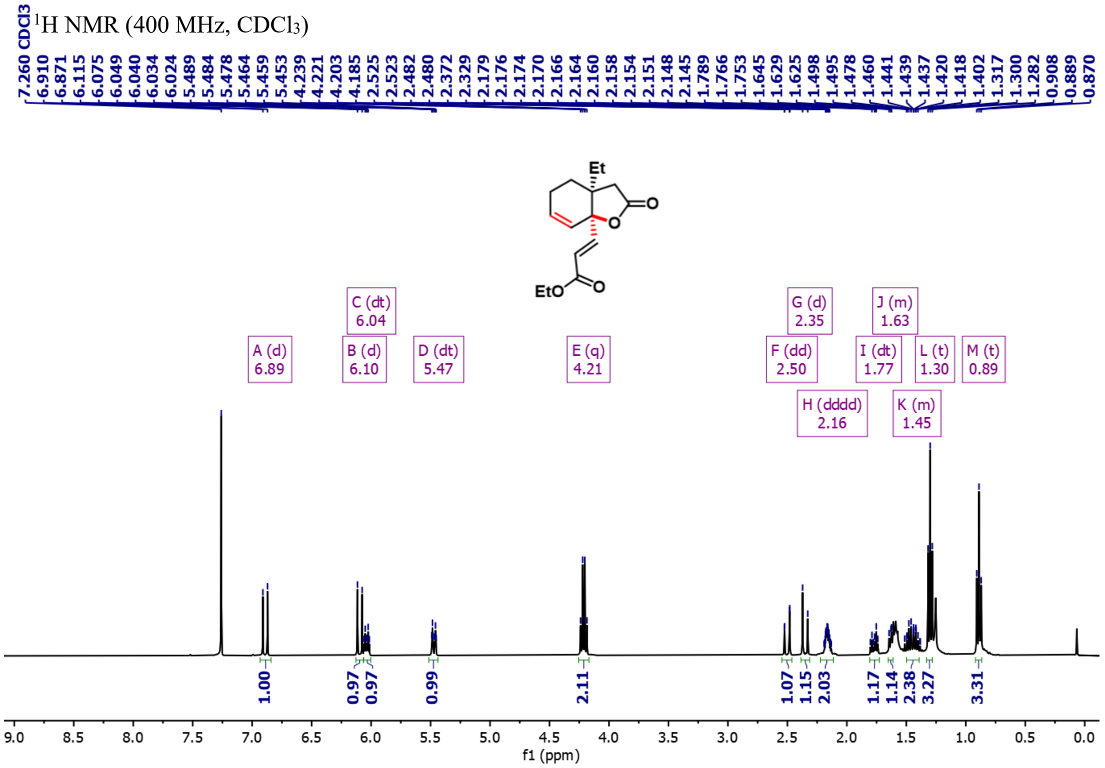

^{13}C NMR (101 MHz, CDCl$_3$)

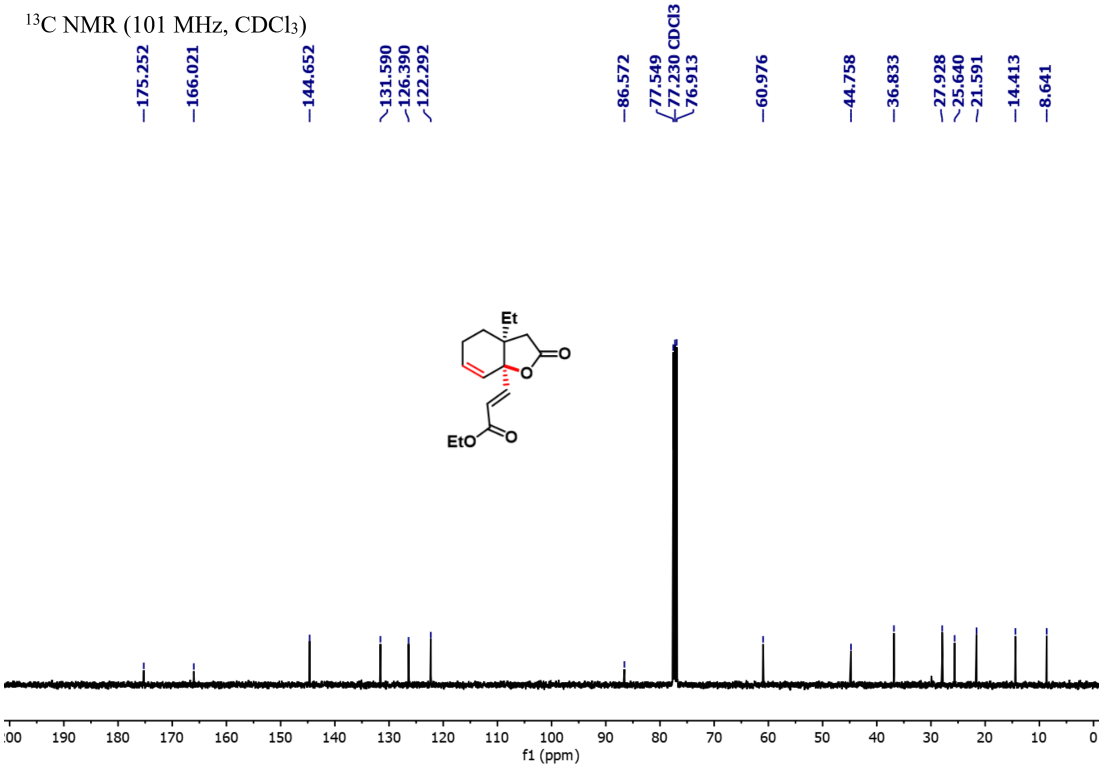

Compound 4c

Ethyl (*E*)-3-((3a*S*,7a*R*)-3a-cyclohexyl-2-oxo-3,3a,4,5-tetrahydrobenzofuran-7a(2H)-yl)acrylate

^{1}H NMR (400 MHz, $CDCl_3$)

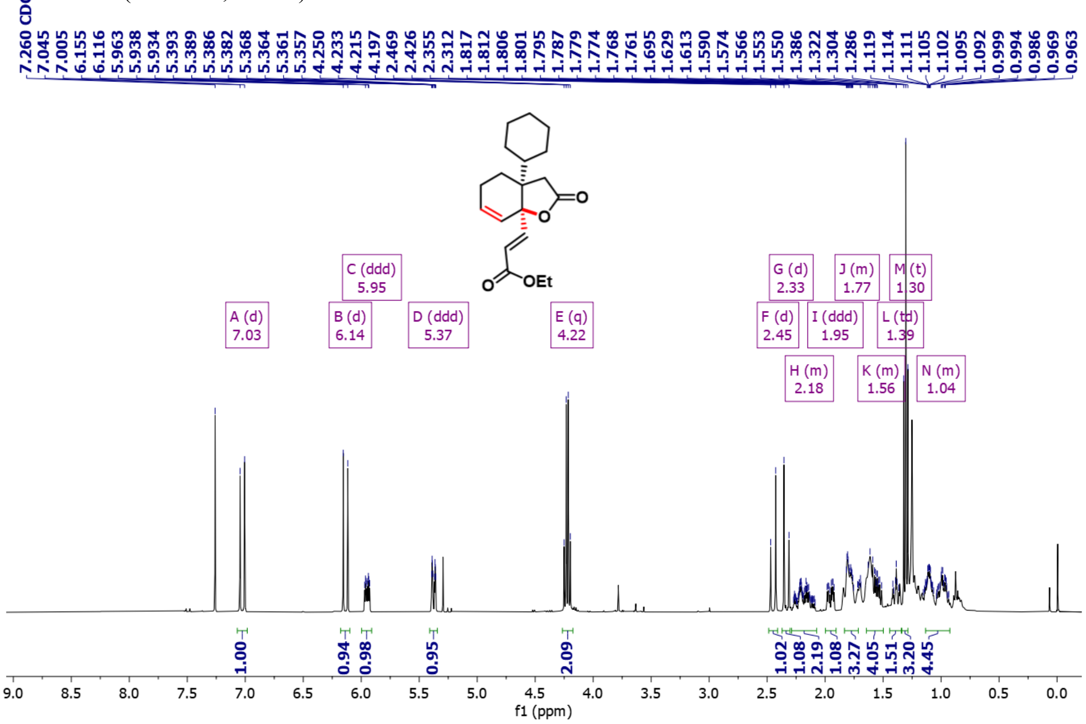

^{13}C NMR (101 MHz, $CDCl_3$)

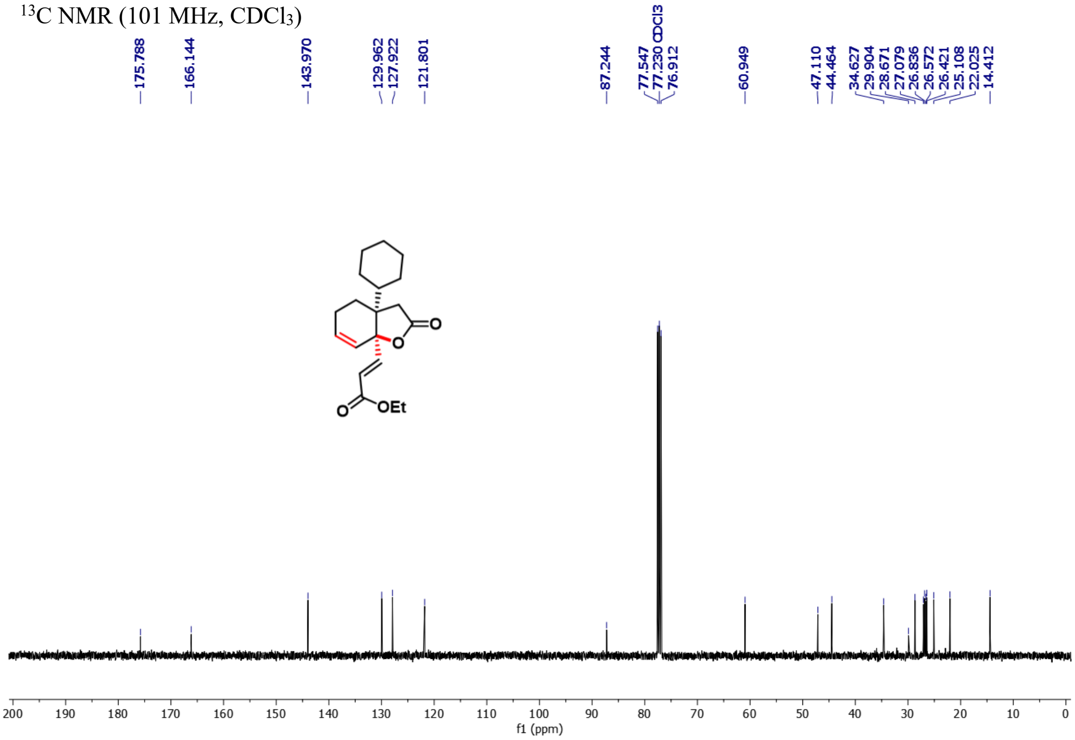

Compound 4d

Ethyl (*E*)-3-((3a*S*,7a*R*)-2-oxo-3a-propyl-3,3a,4,5-tetrahydrobenzofuran-7a(2H)-yl)acrylate

^{1}H NMR (400 MHz, $CDCl_3$)

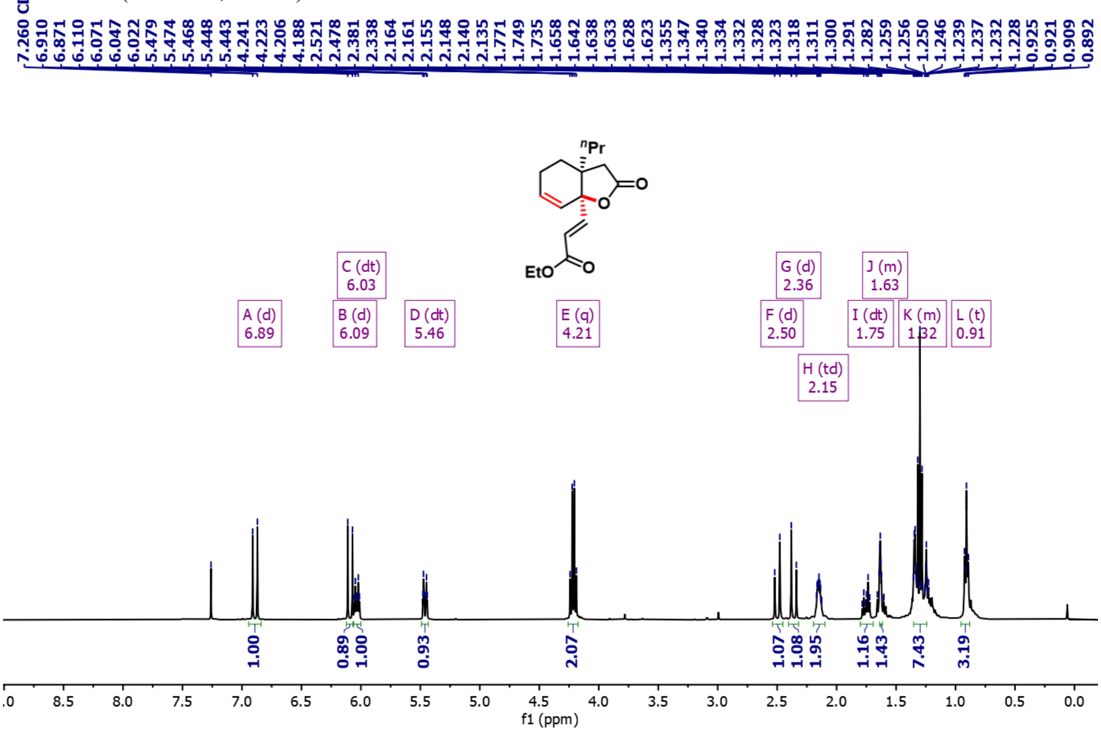

^{13}C NMR (101 MHz, $CDCl_3$)

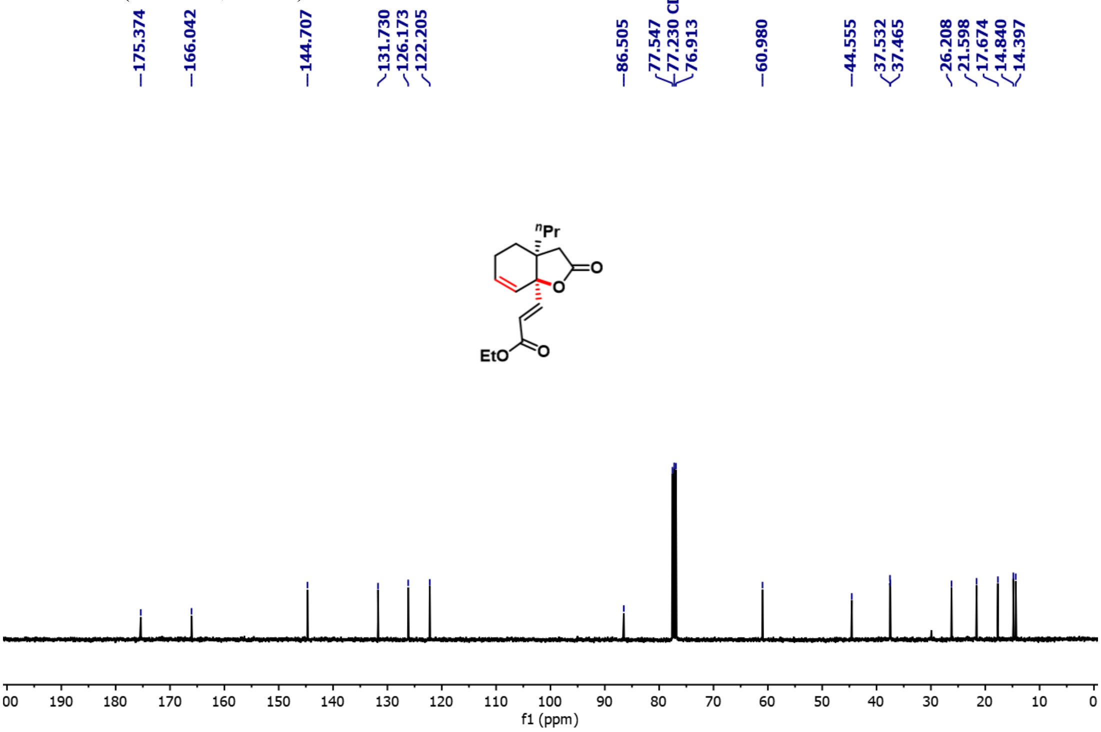

Compound 4e

Ethyl (*E*)-3-((3a*S*,7a*R*)-3a-butyl-2-oxo-3,3a,4,5-tetrahydrobenzofuran-7a(2H)-yl)acrylate

^{1}H NMR (400 MHz, $CDCl_3$)

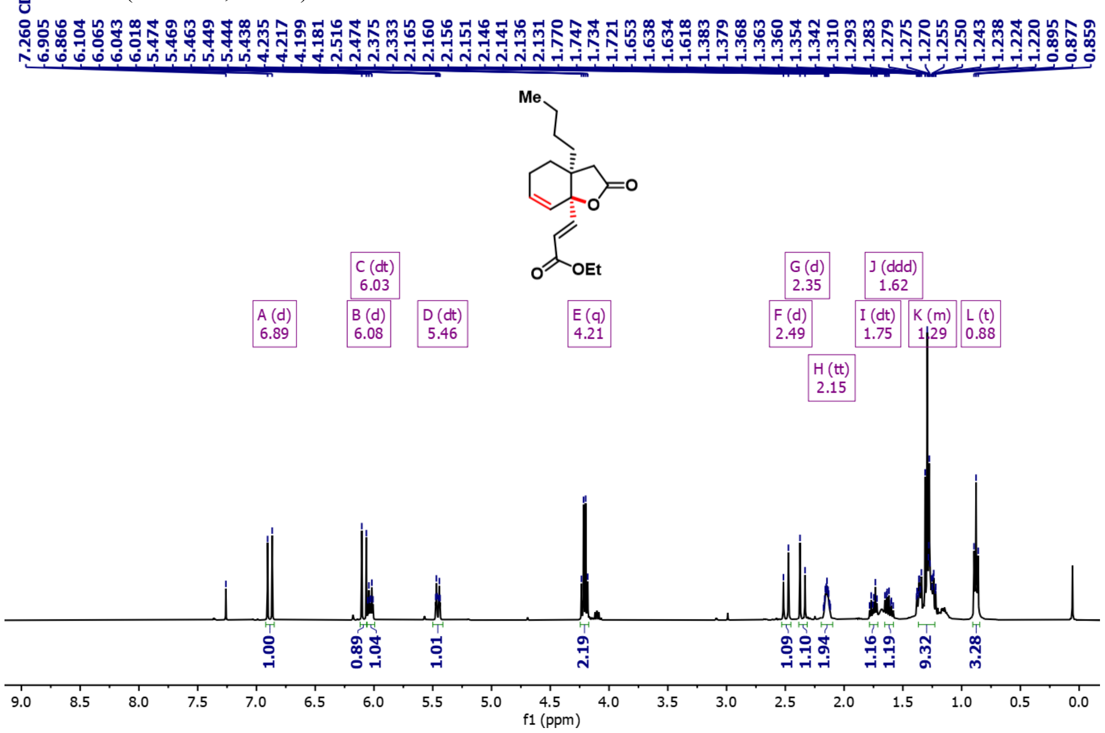

^{13}C NMR (101 MHz, $CDCl_3$)

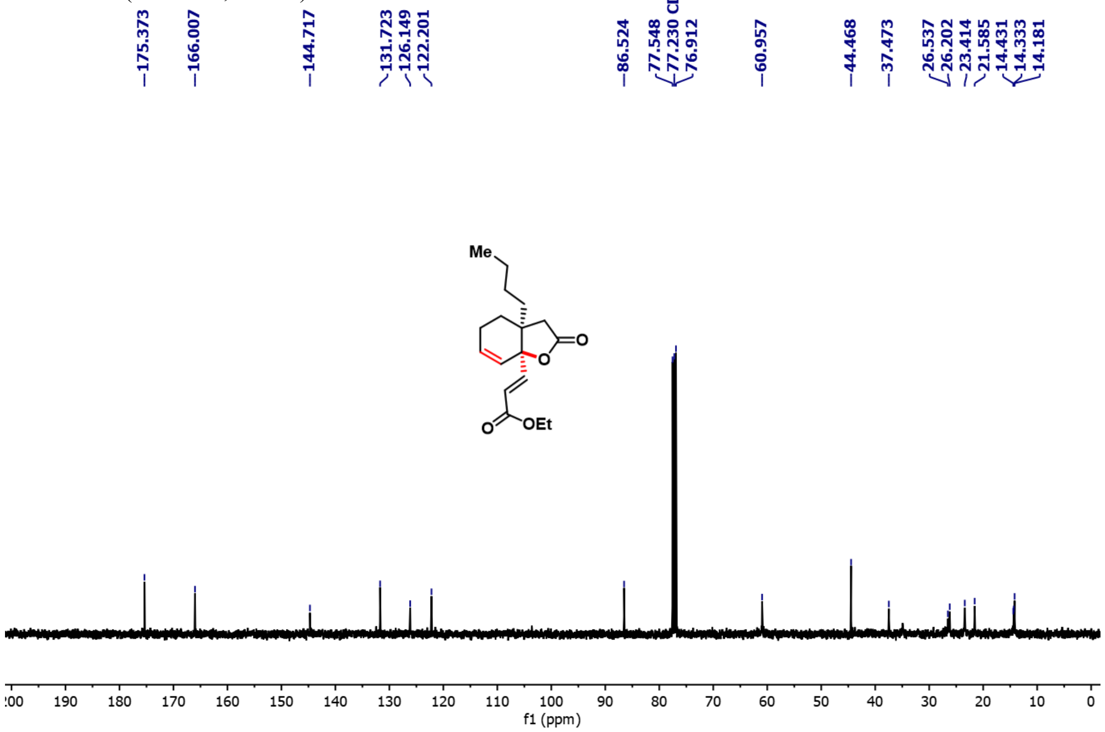

Compound 4f

Ethyl (*E*)-3-((3a*R*,7a*R*)-3a,4-dimethyl-2-oxo-3,3a,4,5-tetrahydrobenzofuran-7a(2H)-yl)acrylate

^{1}H NMR (500 MHz, $CDCl_3$)

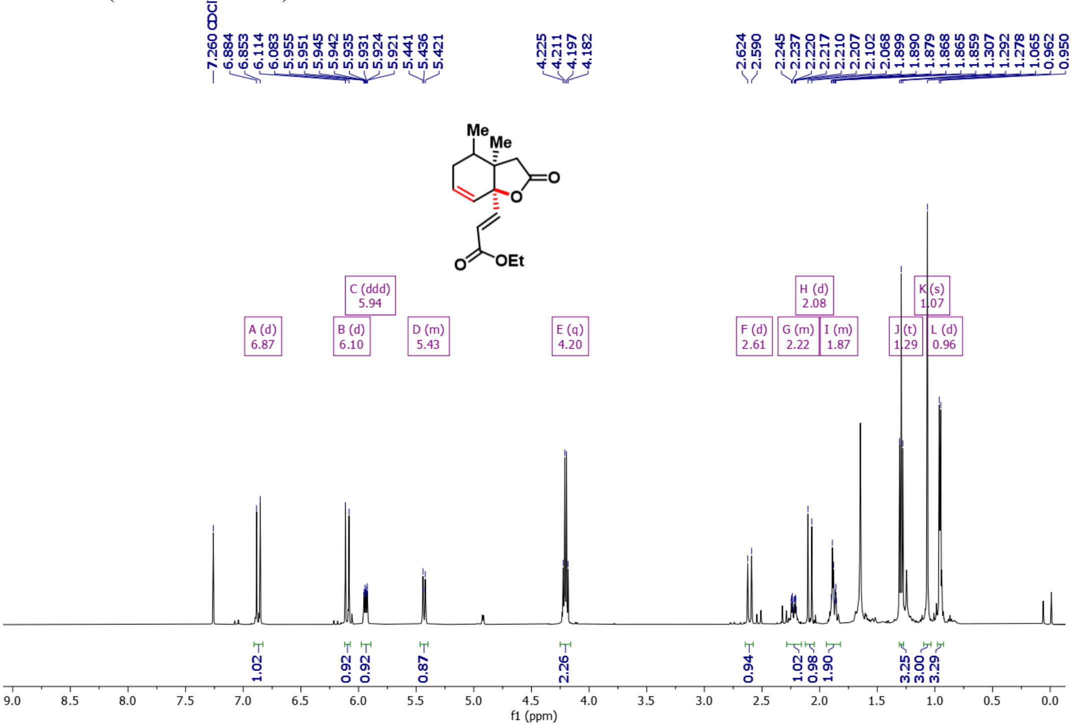

^{13}C NMR (126 MHz, $CDCl_3$)

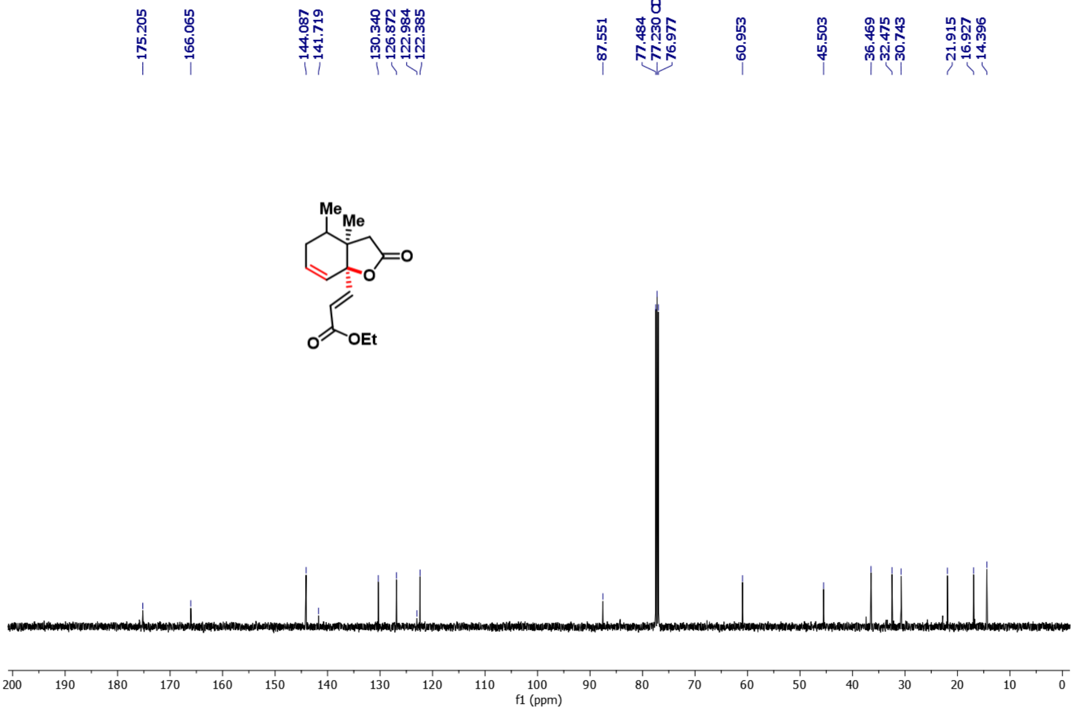

NOE Experiment:

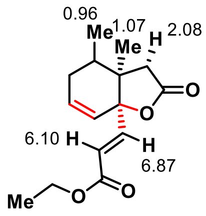

Me (1.07) $\xrightarrow{\text{Strong NOE}}$ H (6.87)

Me (1.07) $\xrightarrow{\text{Strong NOE}}$ H (2.08)

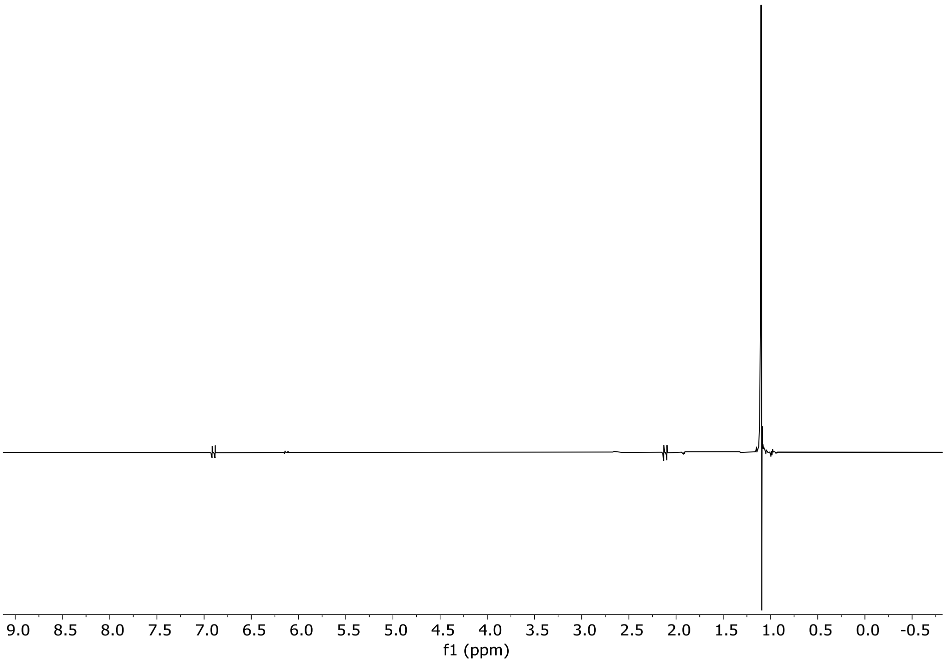

NOESY Experiment:

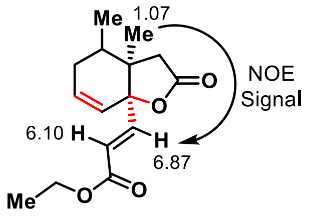

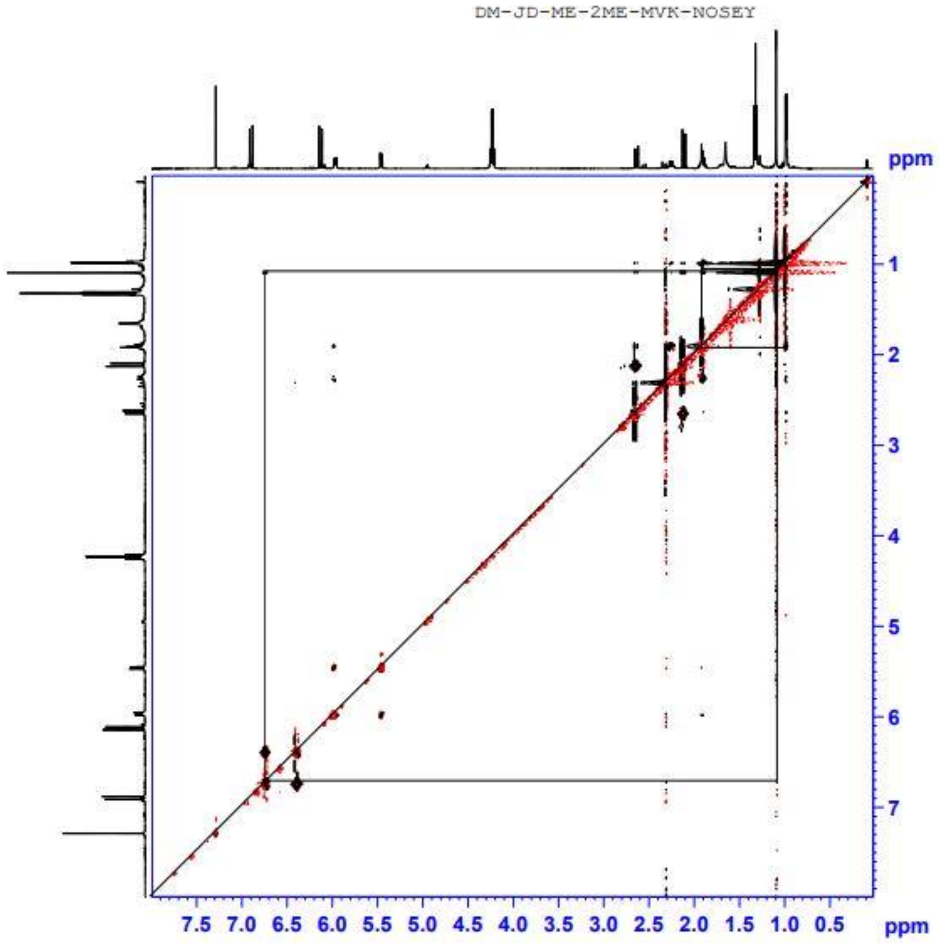

Compound 4g

Ethyl (*E*)-3-((3a*S*,7a*R*)-3a,5-dimethyl-2-oxo-3,3a,4,5-tetrahydrobenzofuran-7a(2H)-yl)acrylate

^{1}H NMR (400 MHz, $CDCl_3$)

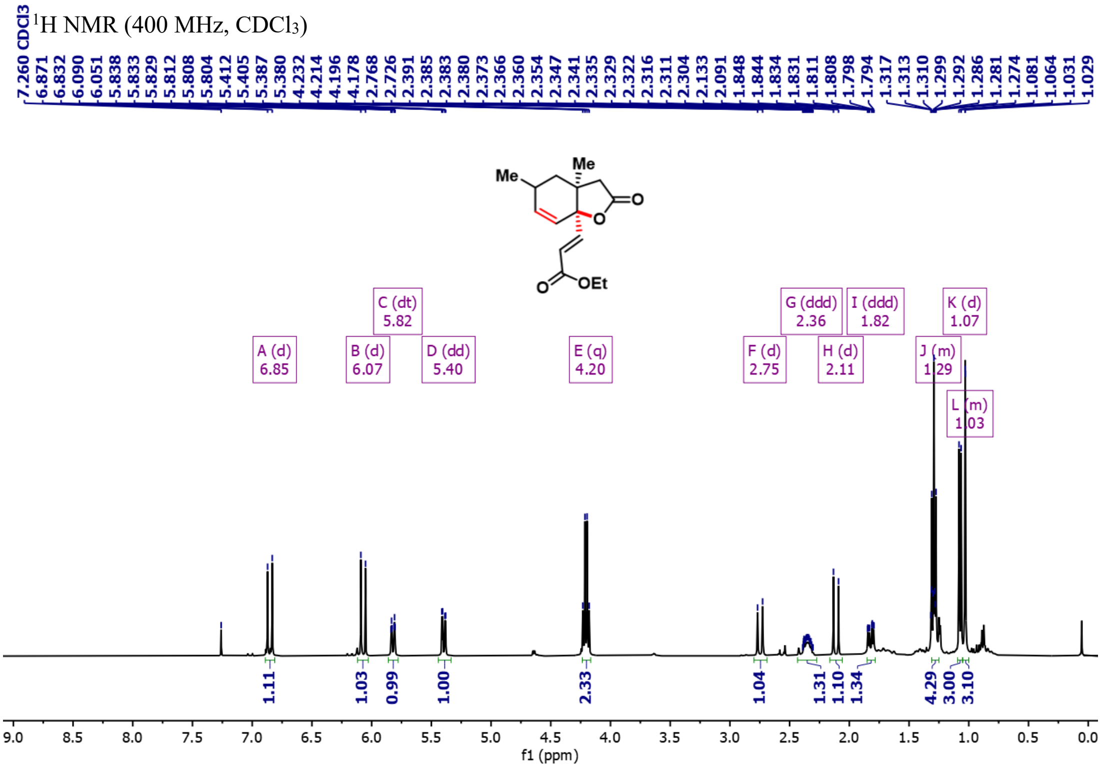

^{13}C NMR (101 MHz, $CDCl_3$)

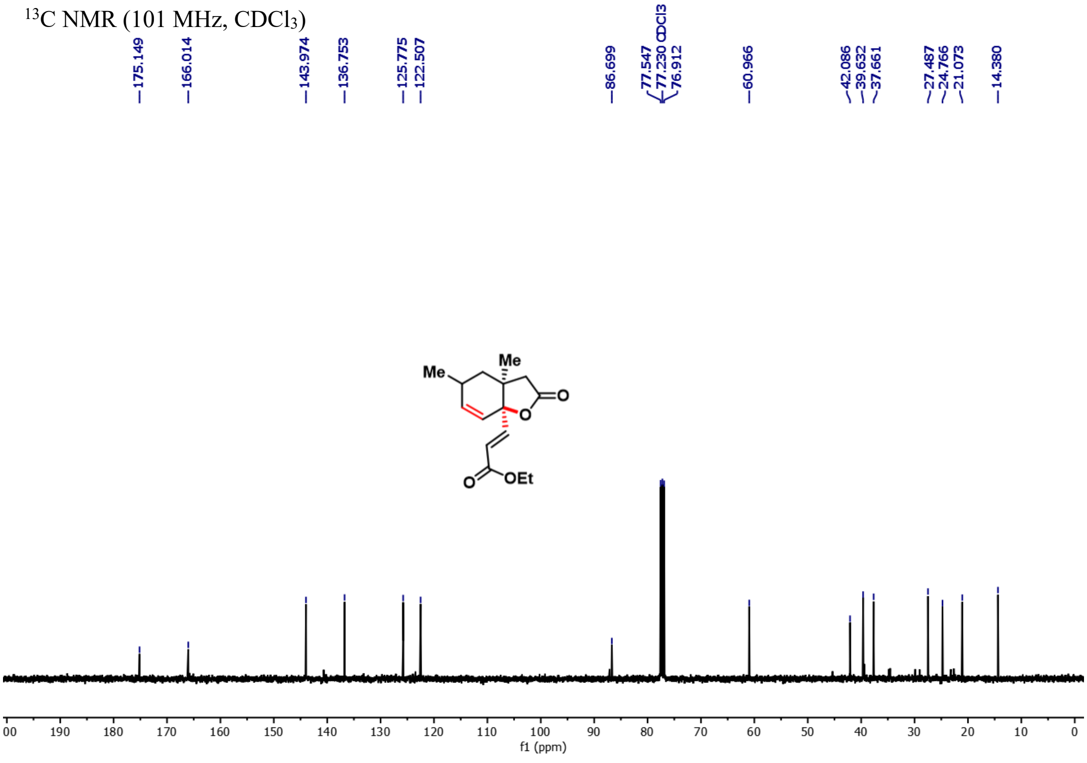

Compound 4h

Ethyl (*E*)-3-((3a*S*,7a*R*)-3a-isobutyl-5-methyl-2-oxo-3,3a,4,5-tetrahydrobenzofuran-7a(2H)-yl)acrylate

^{1}H NMR (400 MHz, $CDCl_3$)

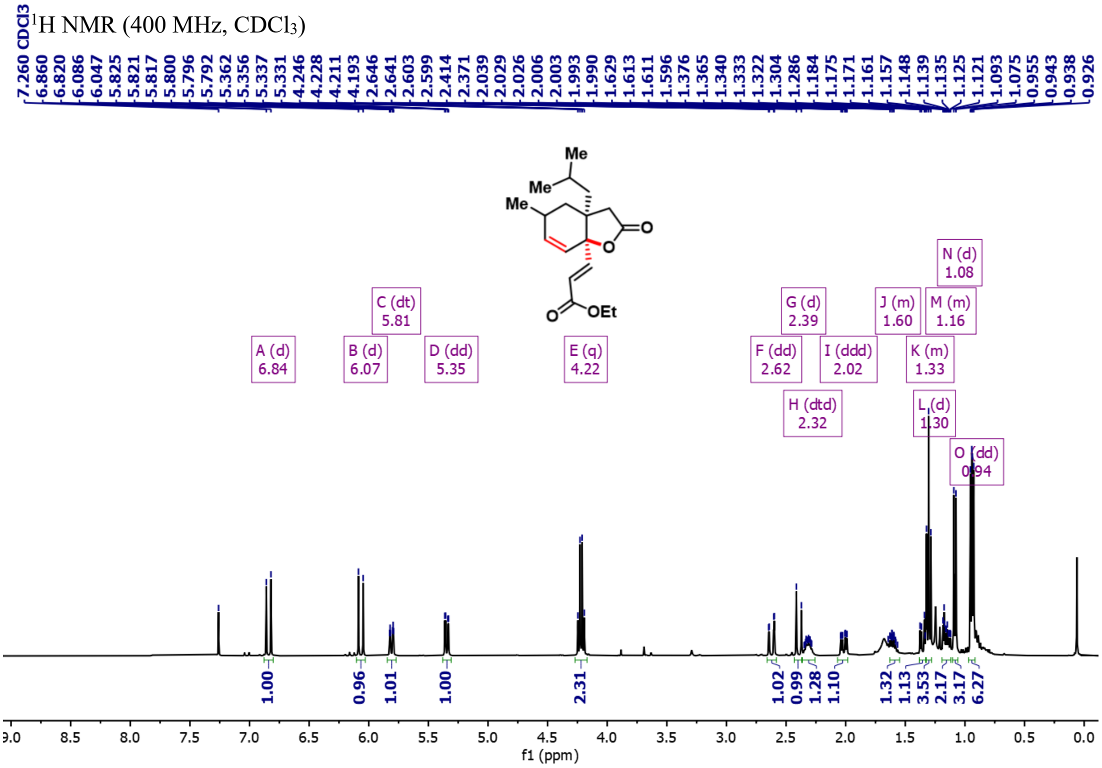

^{13}C NMR (101 MHz, $CDCl_3$)

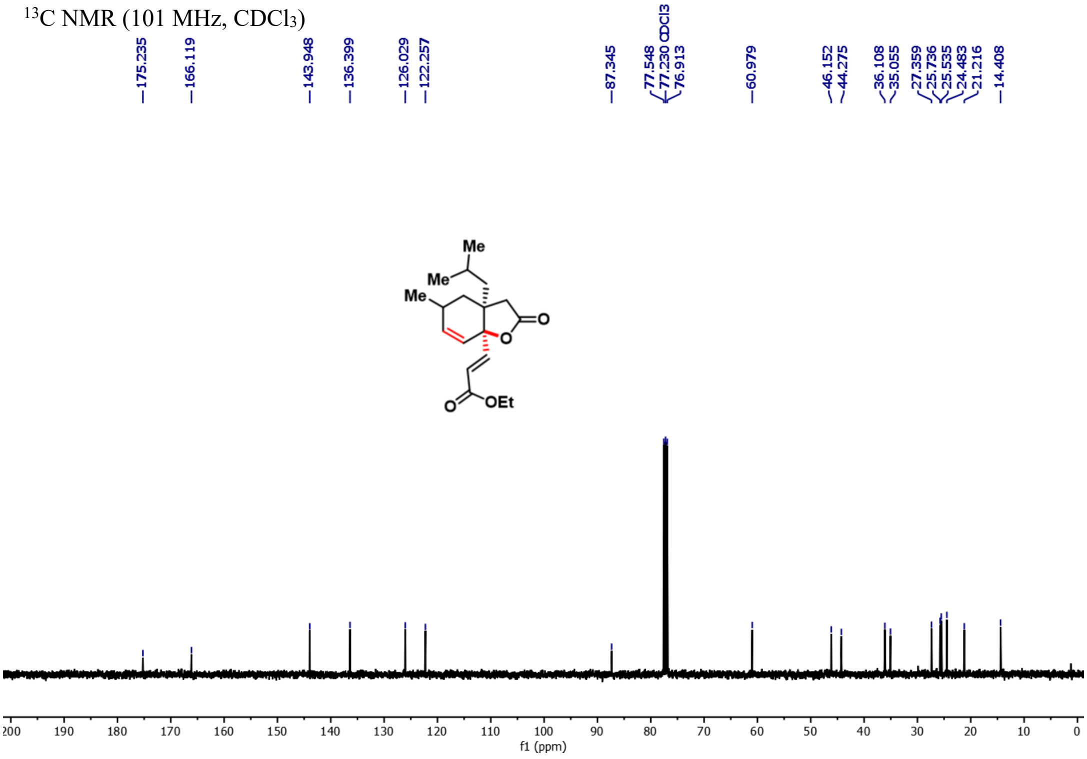

Compound 4i

Methyl (*E*)-3-((3a*S*,7a*R*)-3a-methyl-2-oxo-3,3a,4,5-tetrahydrobenzofuran-7a(2H)-yl)acrylate

^{1}H NMR (400 MHz, $CDCl_3$)

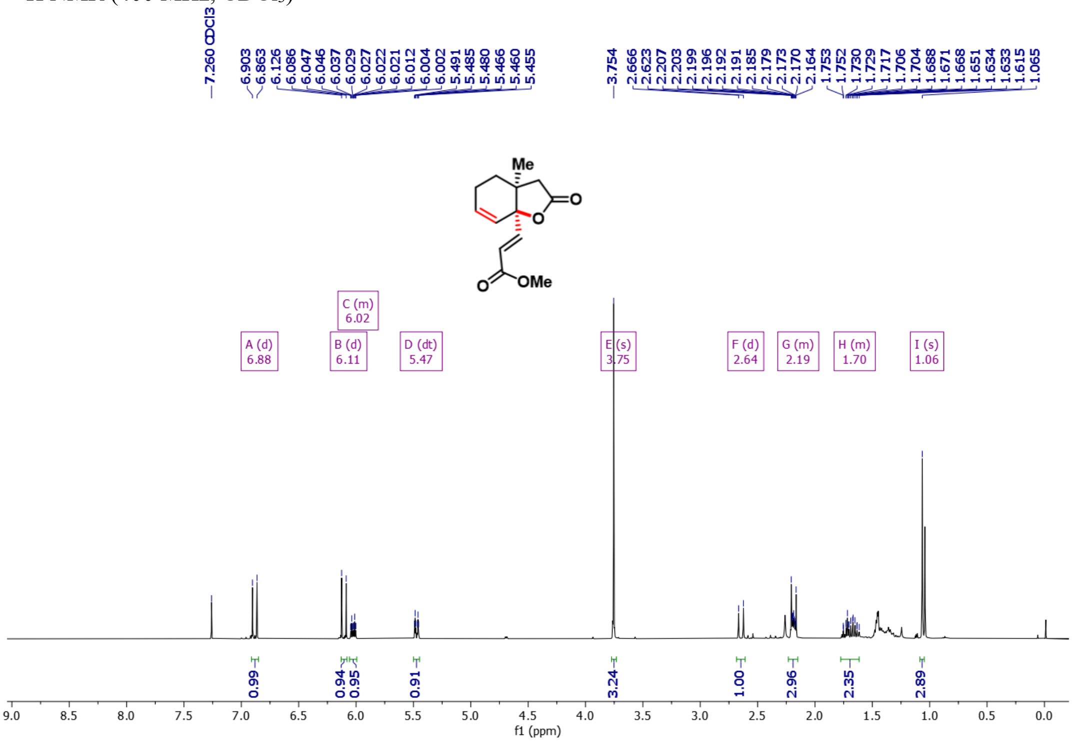

^{13}C NMR (126 MHz, $CDCl_3$)

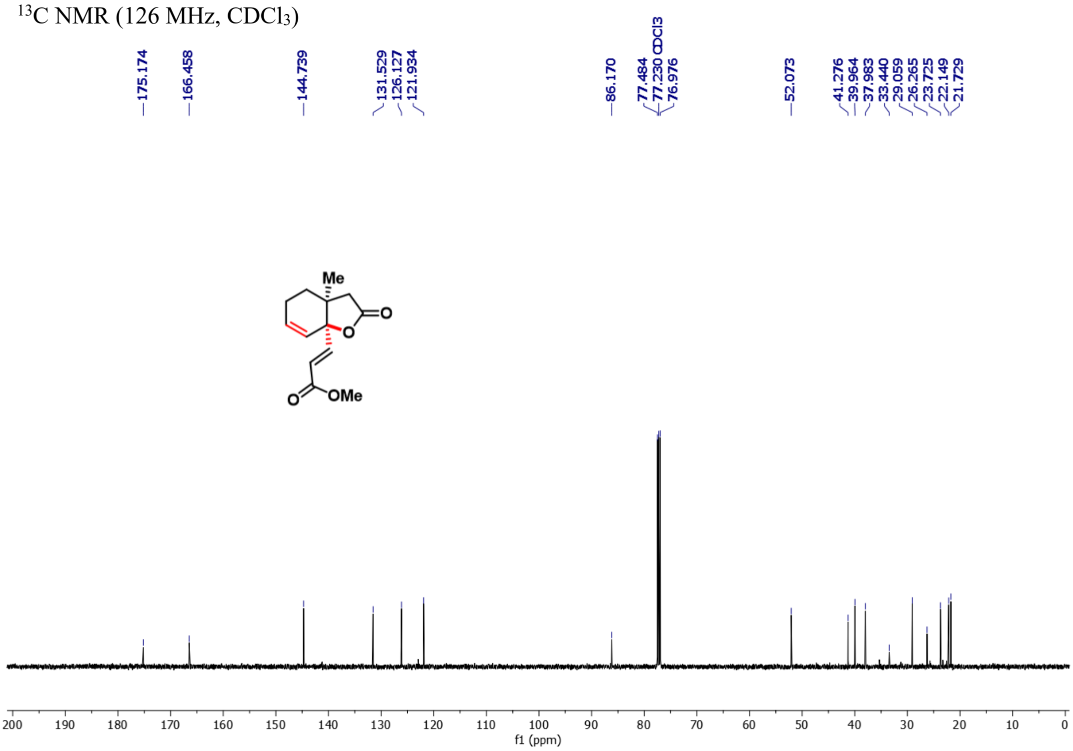

NOE Experiment:

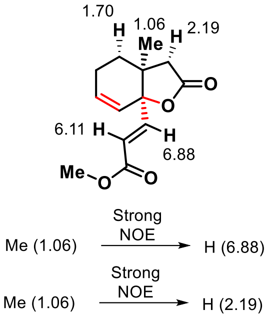

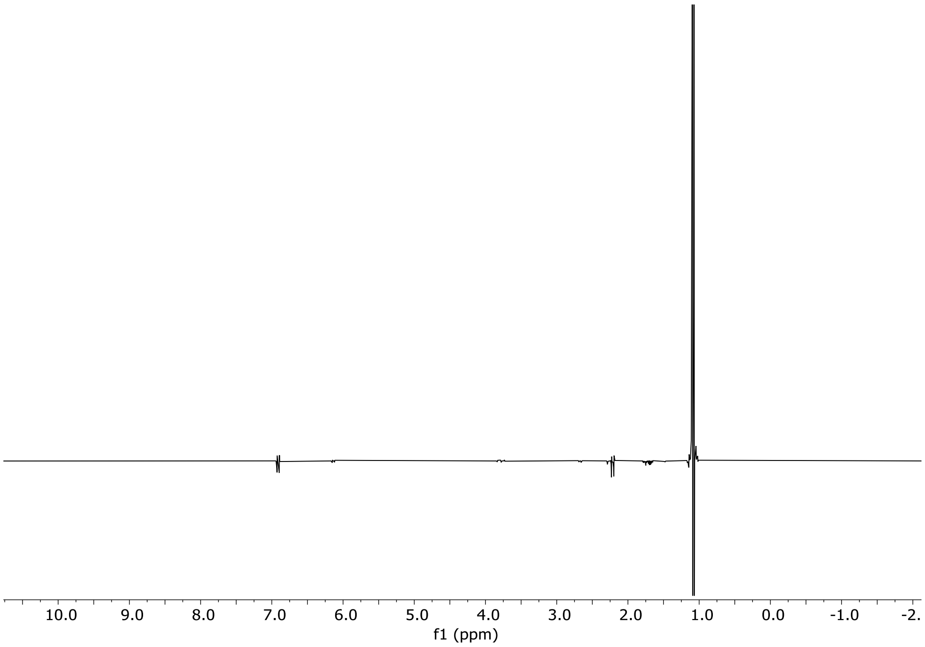

COSY Experiment:

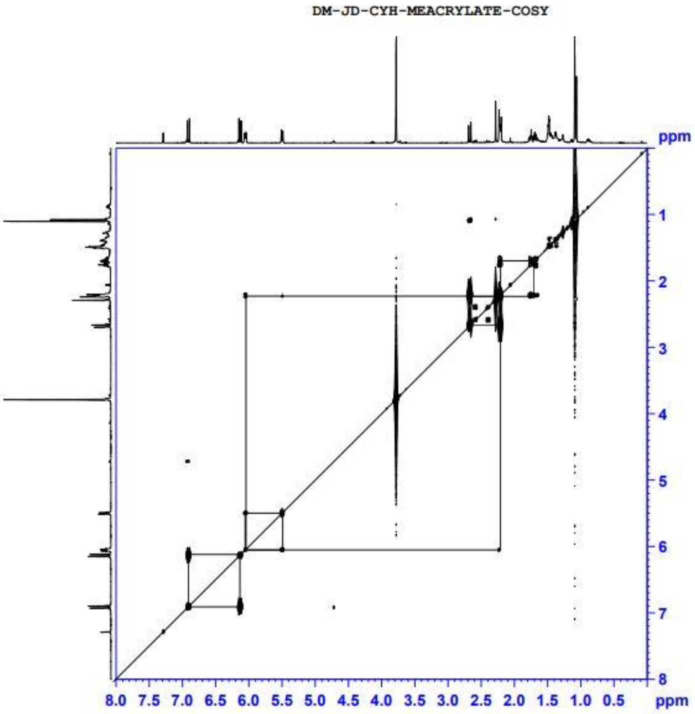

NOESY Experiment:

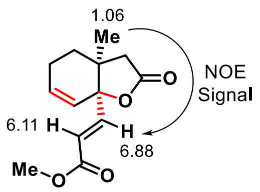

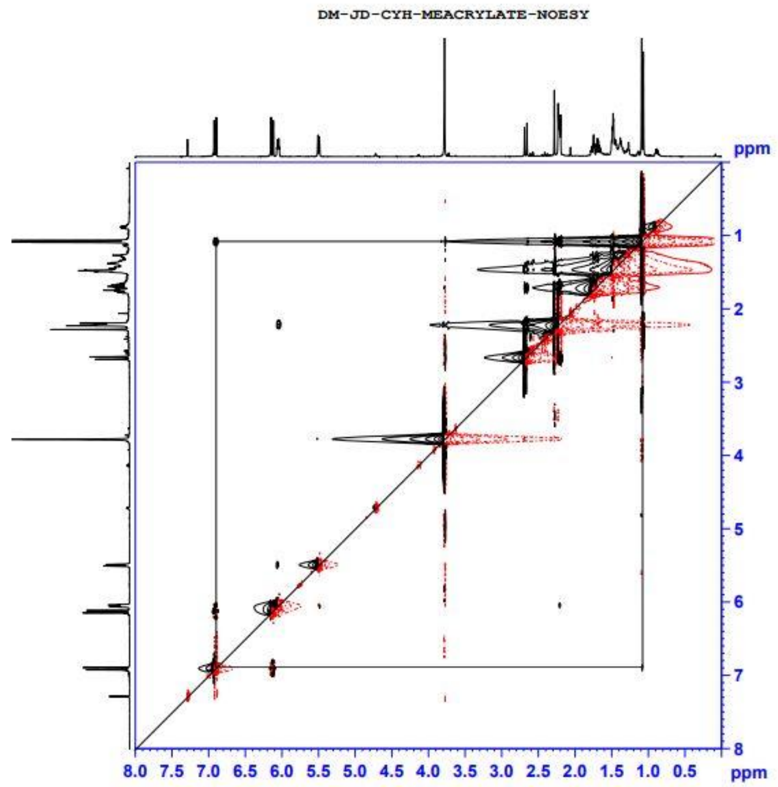
DM-JD-CYH-MEACRYLATE-NOESY
ppm
1
2
3
4
5
6
7
8
8.0 7.5 7.0 6.5 6.0 5.5 5.0 4.5 4.0 3.5 3.0 2.5 2.0 1.5 1.0 0.5
ppm

Compound 4j

Butyl (*E*)-3-((3a*S*,7a*R*)-2-oxo-3a-propyl-3,3a,4,5-tetrahydrobenzofuran-7a(2H)-yl)acrylate

^{1}H NMR (400 MHz, CDCl$_3$)

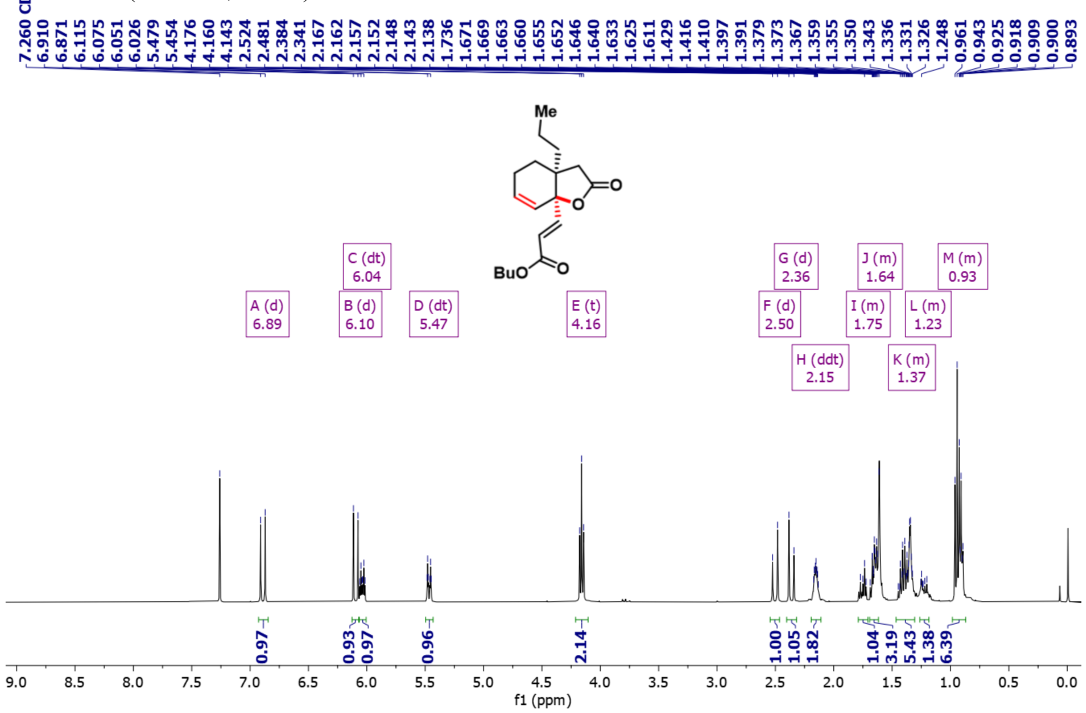

^{13}C NMR (101 MHz, CDCl$_3$)

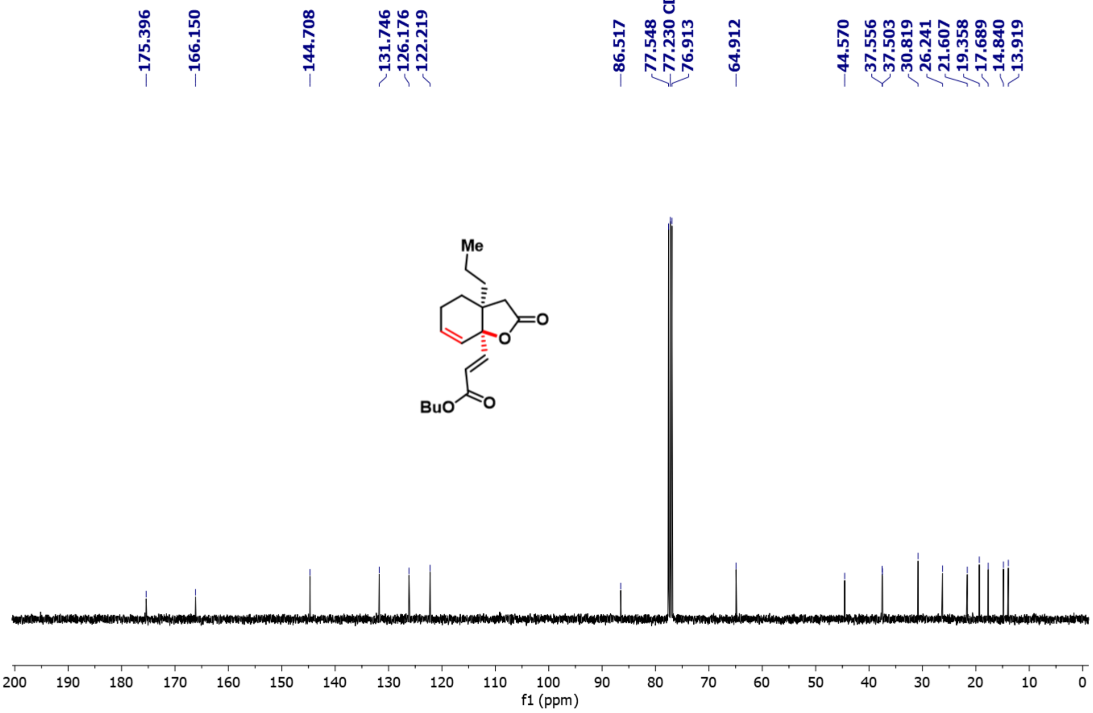

Compound 4k

7-(l2-Fluoraneyl)-7,7,7,7,7,7,7,7,7,7,7-undecafluoro-7l15-hepta-2,4,6-triyn-1-yl (*E*)-3-((3a*S*,7a*R*)-2-oxo-3a-propyl-3,3a,4,5-tetrahydrobenzofuran-7a(2H)-yl)acrylate

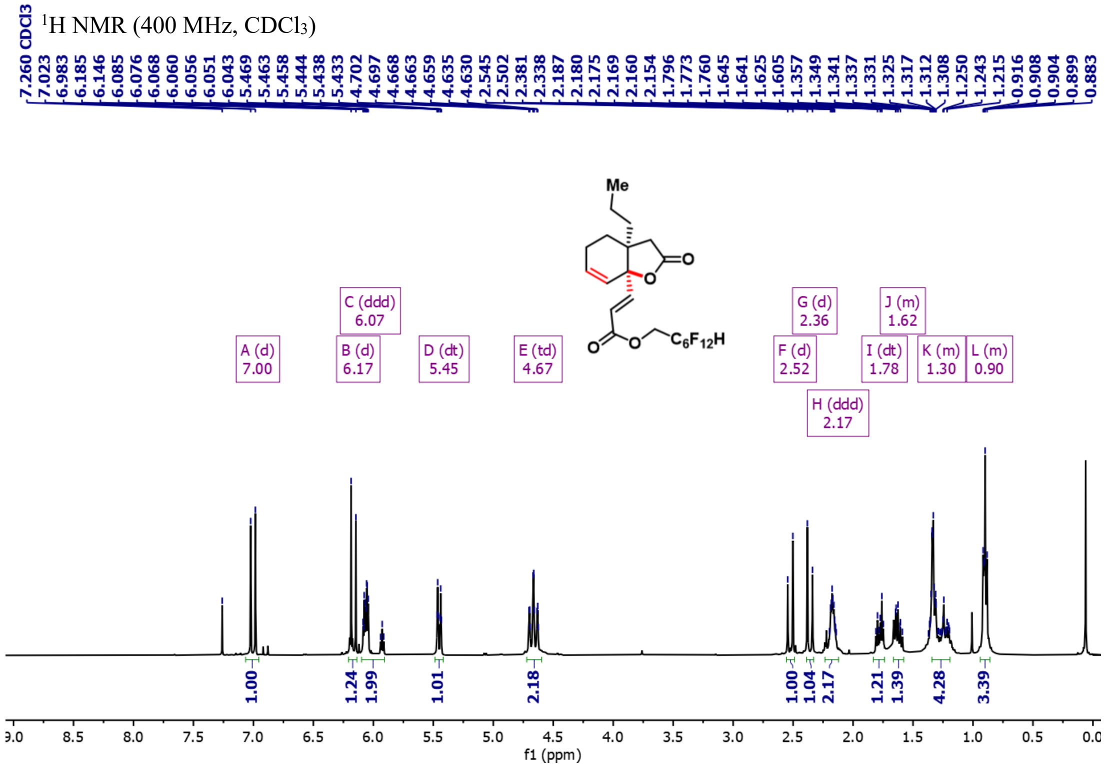

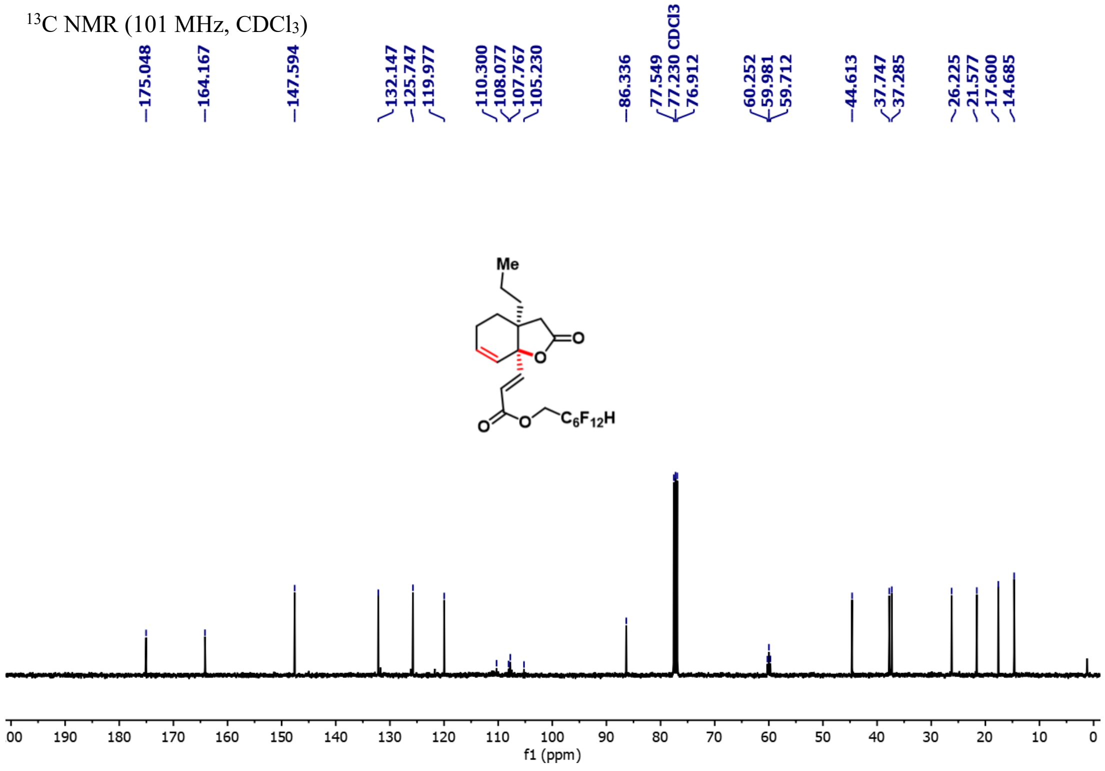

^{19}F NMR (376 MHz, $CDCl_3$)

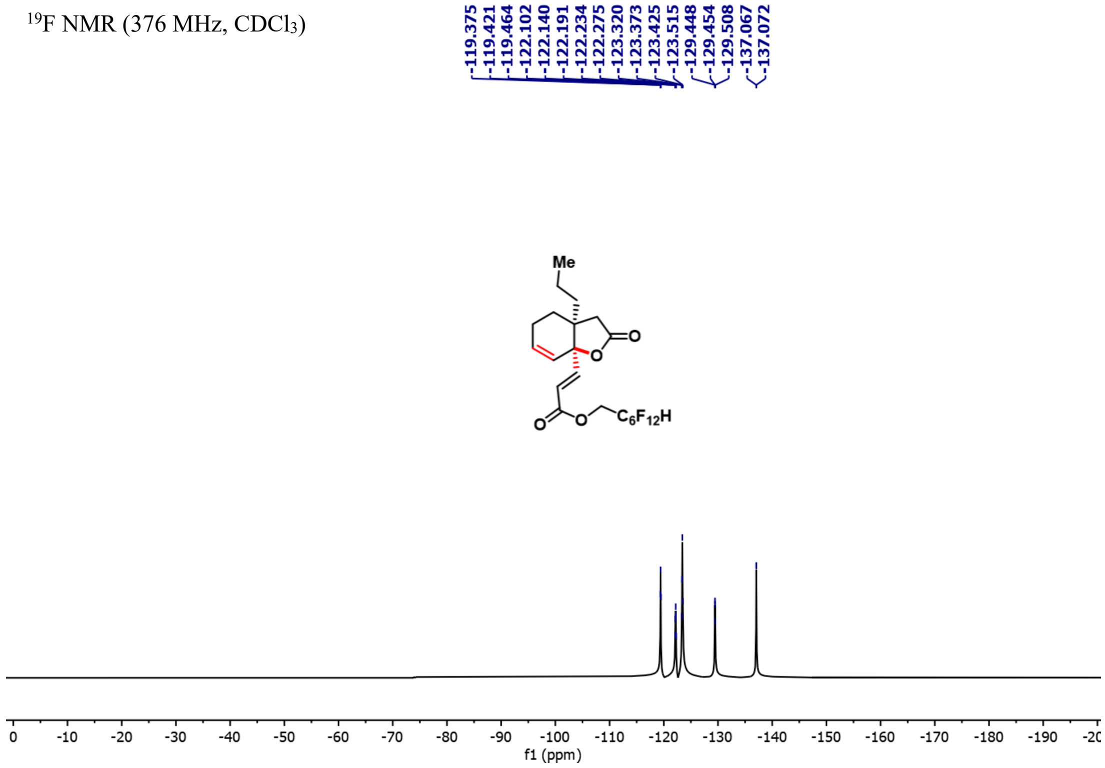

Compound 4l

Benzyl (*E*)-3-((3a*S*,7a*R*)-2-oxo-3a-propyl-3,3a,4,5-tetrahydrobenzofuran-7a(2H)-yl)acrylate

^{1}H NMR (400 MHz, $CDCl_3$)

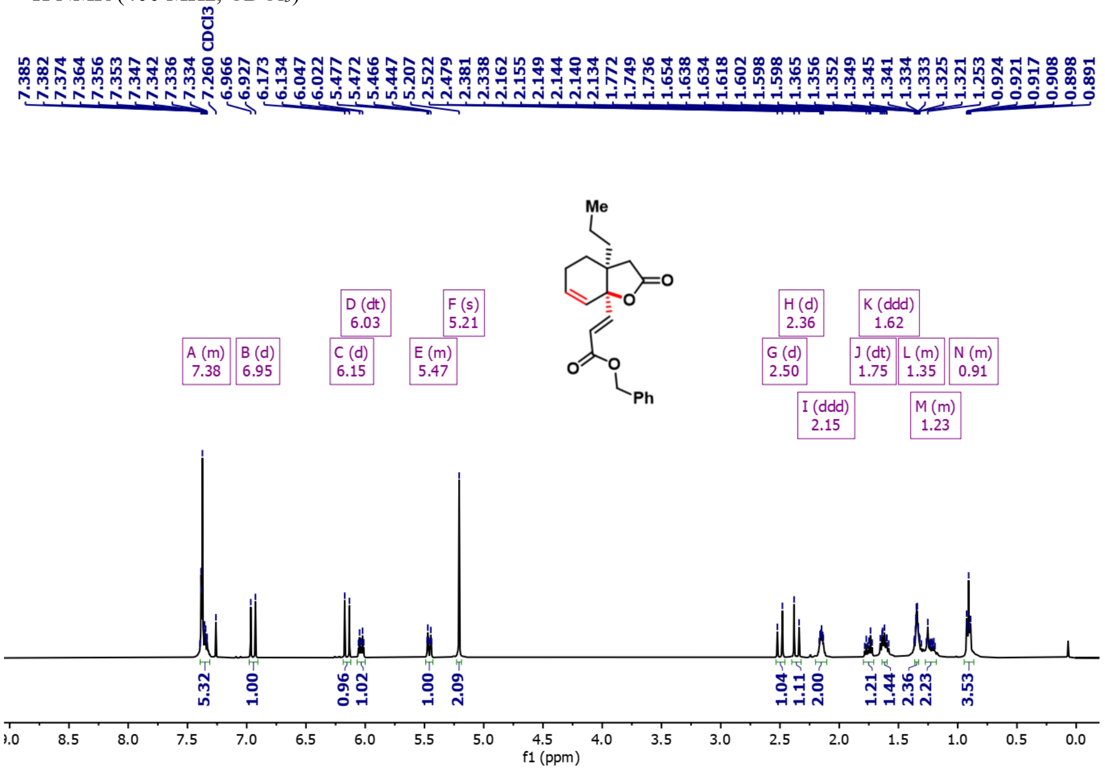

^{13}C NMR (101 MHz, $CDCl_3$)

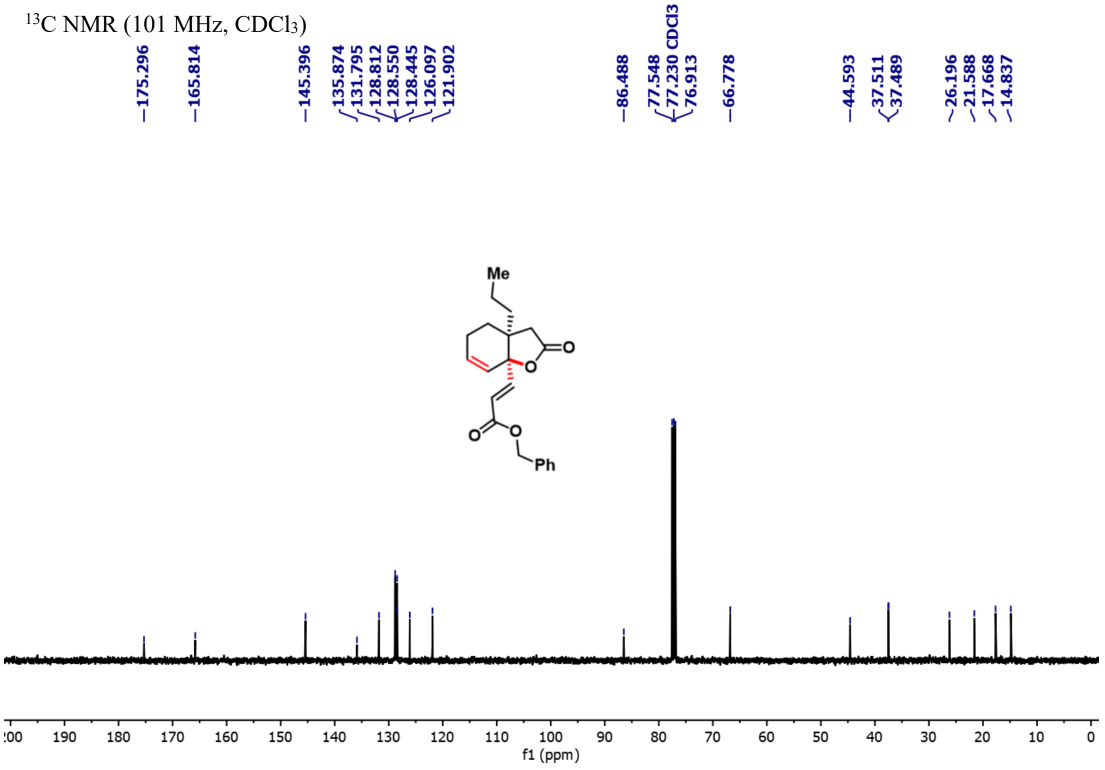

Compound 4m

2-Fluorobenzyl (*E*)-3-((3a*S*,7a*R*)-3a-ethyl-2-oxo-3,3a,4,5-tetrahydrobenzofuran-7a(2H)-yl)acrylate

^{1}H NMR (400 MHz, $CDCl_3$)

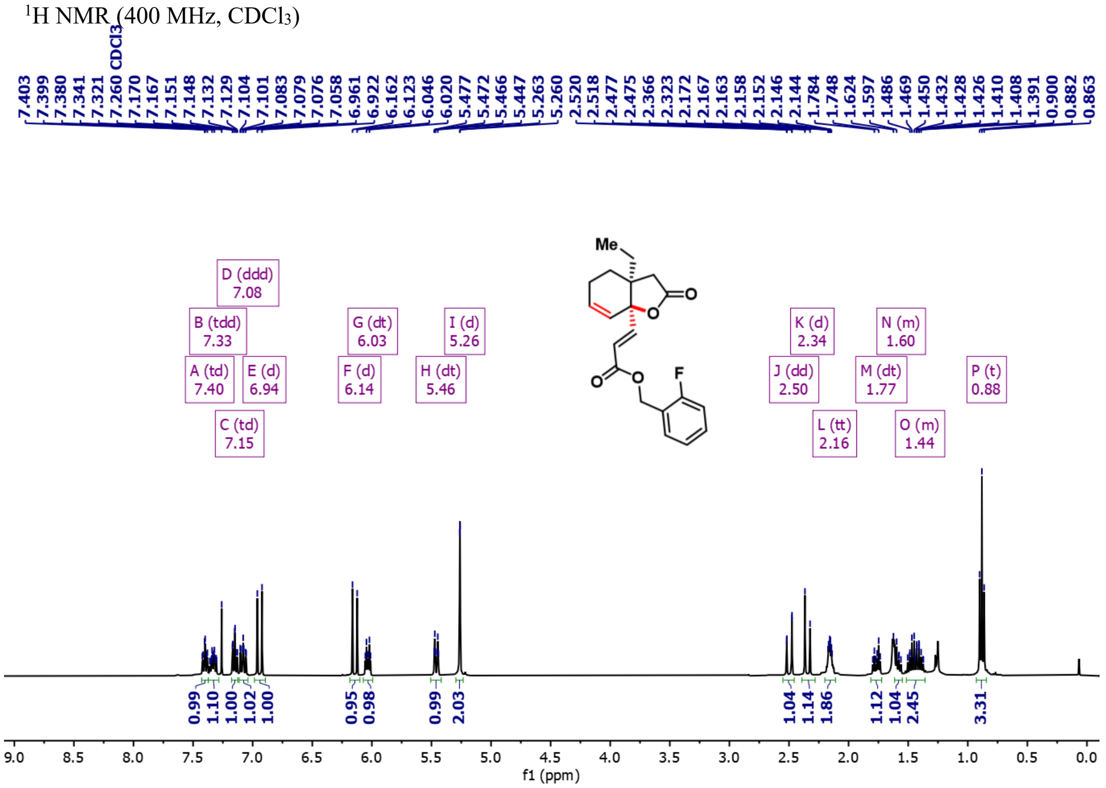

^{13}C NMR (101 MHz, $CDCl_3$)

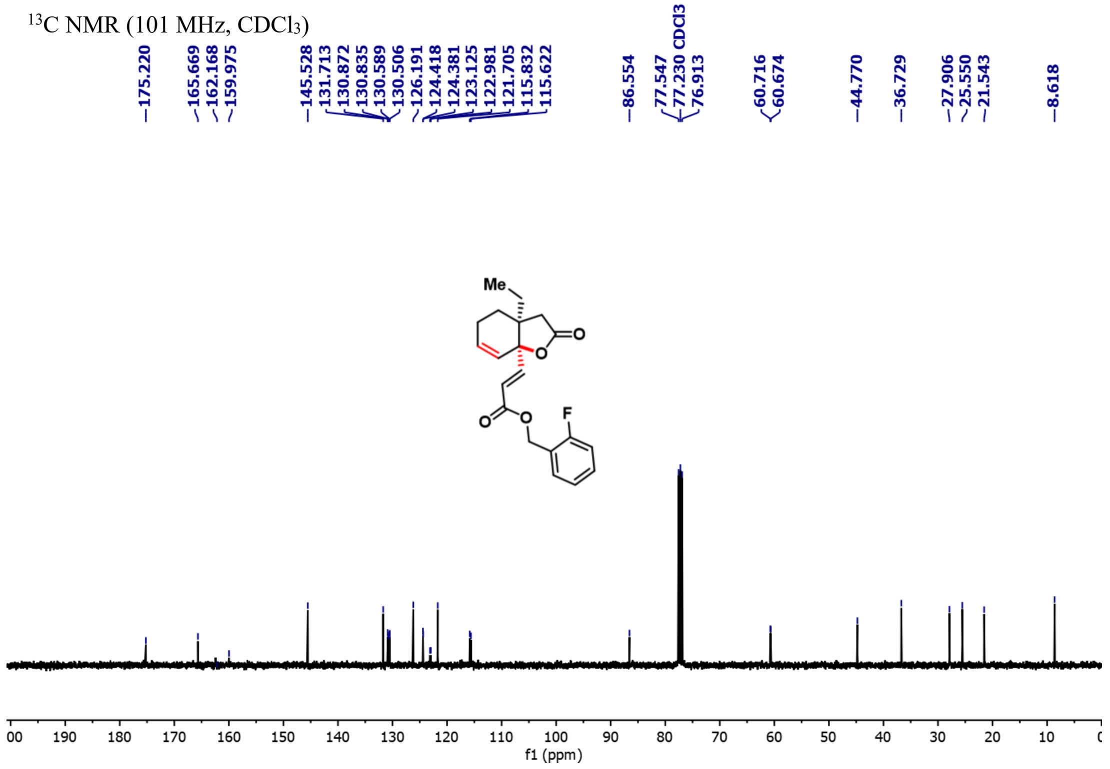

^{19}F NMR (376 MHz, $CDCl_3$)

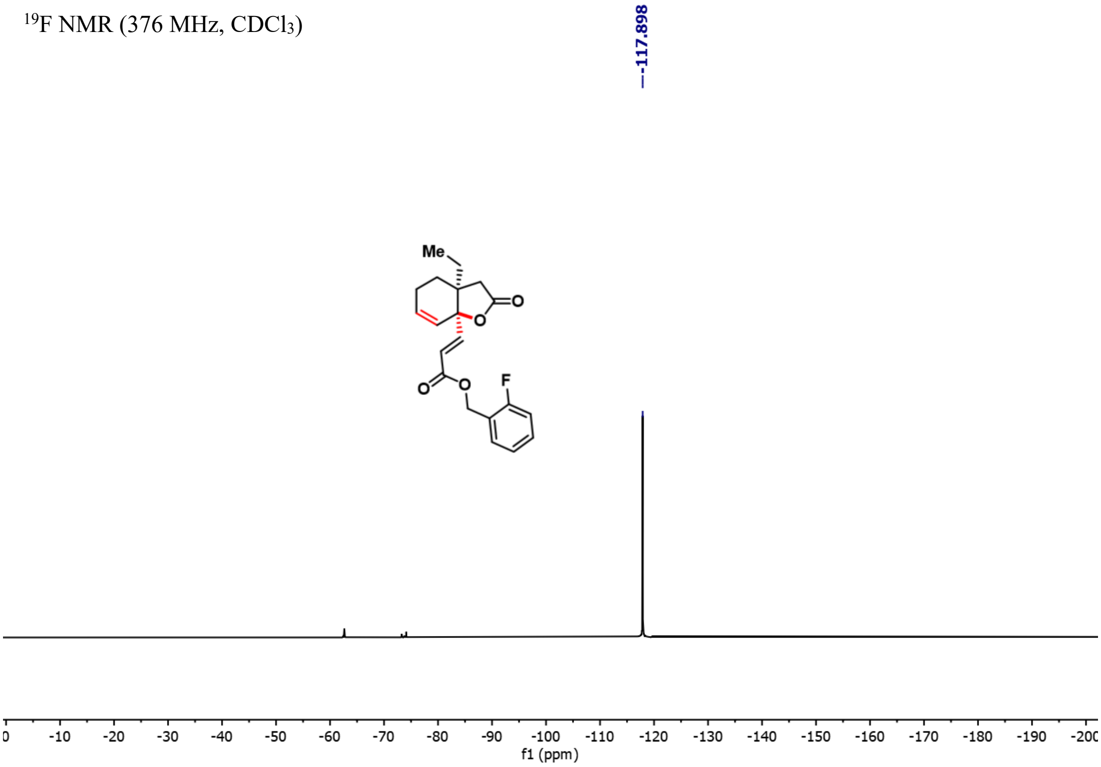

Compound 4n

4-Chlorobenzyl (*E*)-3-((3a*S*,7a*R*)-3a-ethyl-2-oxo-3,3a,4,5-tetrahydrobenzofuran-7a(2H)-yl)acrylate

^{1}H NMR (400 MHz, CDCl$_3$)

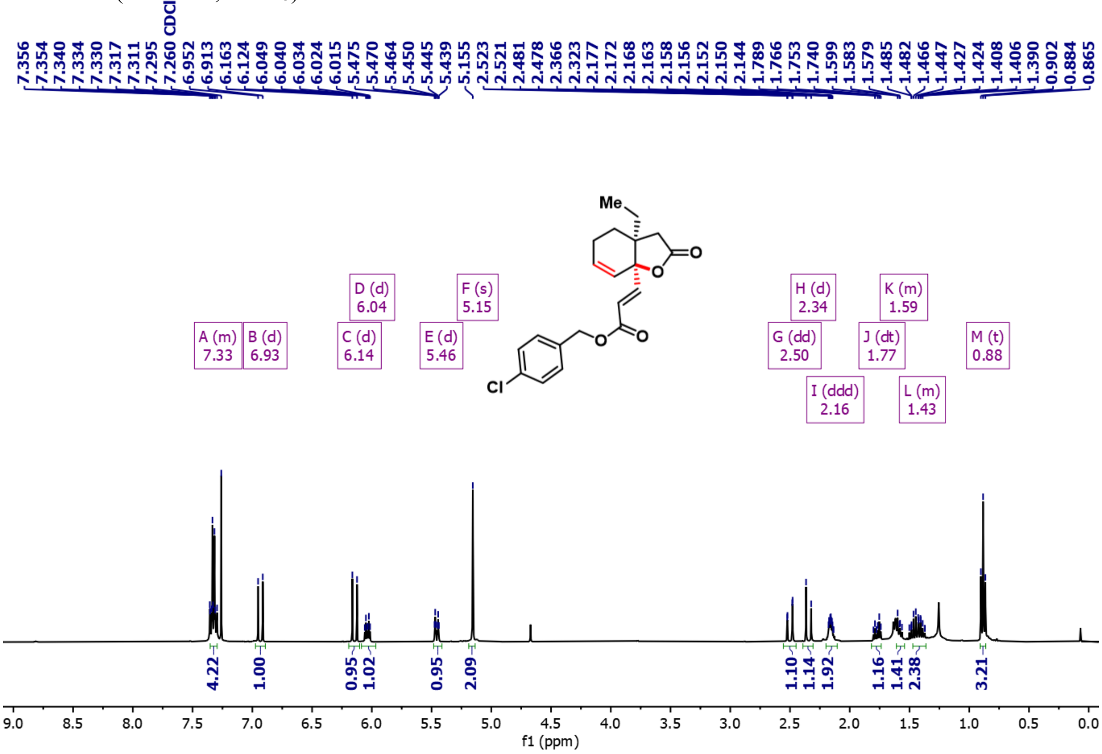

^{13}C NMR (101 MHz, CDCl$_3$)

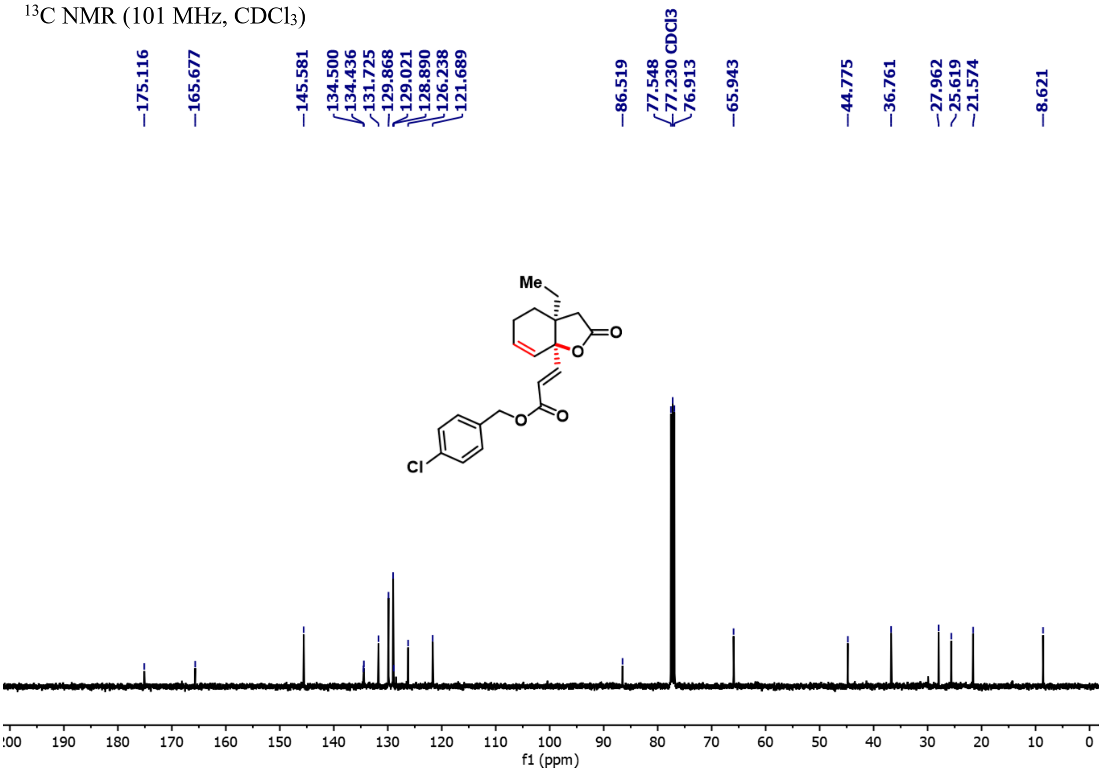

Compound 4o

3-Methylbenzyl (*E*)-3-((3a*S*,7a*R*)-3a-ethyl-2-oxo-3,3a,4,5-tetrahydrobenzofuran-7a(2H)-yl)acrylate

^{1}H NMR (500 MHz, $CDCl_3$)

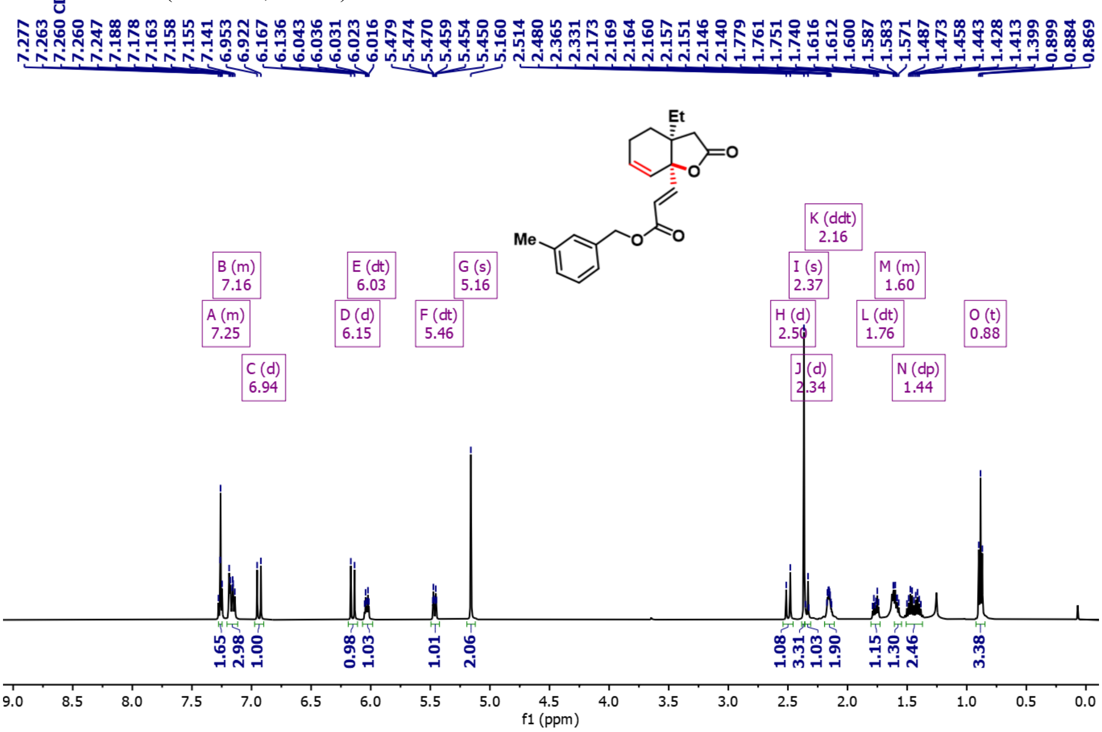

^{13}C NMR (126 MHz, $CDCl_3$)

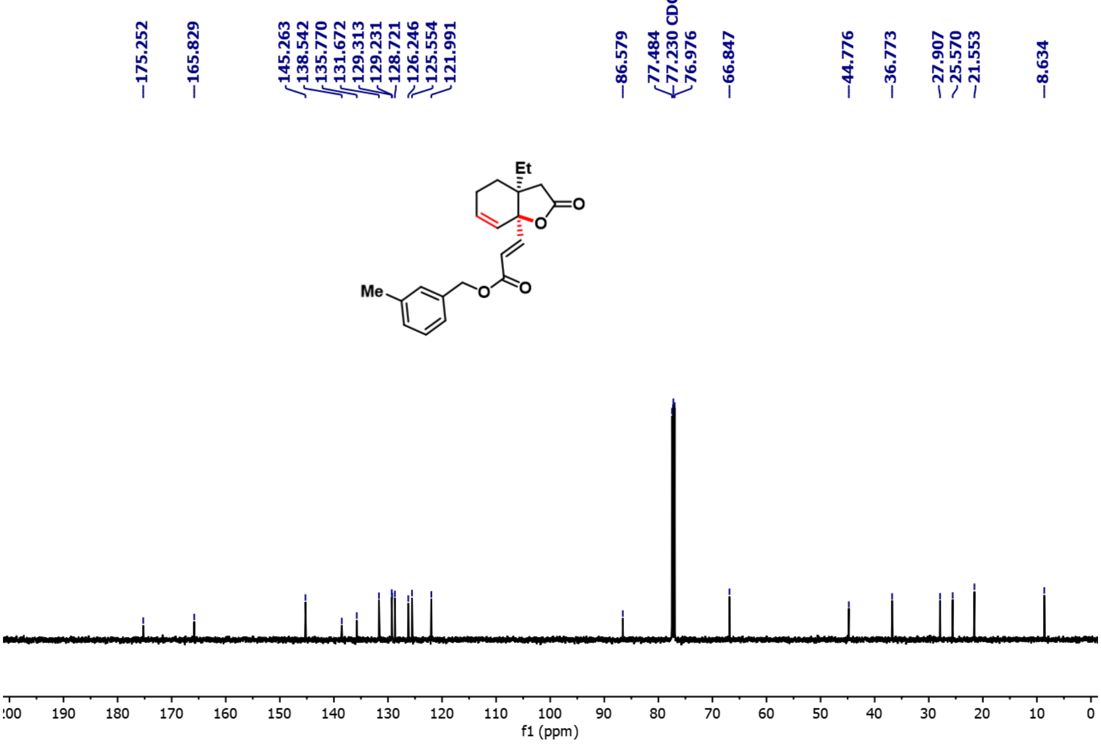

COSY Experiment:

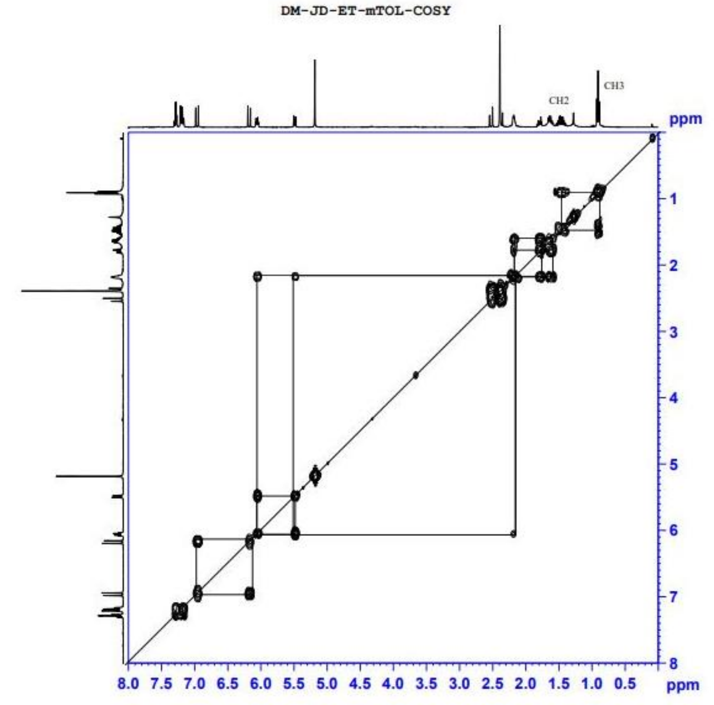

NOESY Experiment:

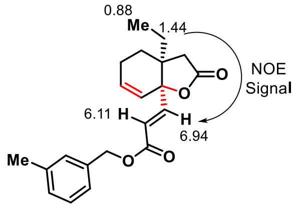

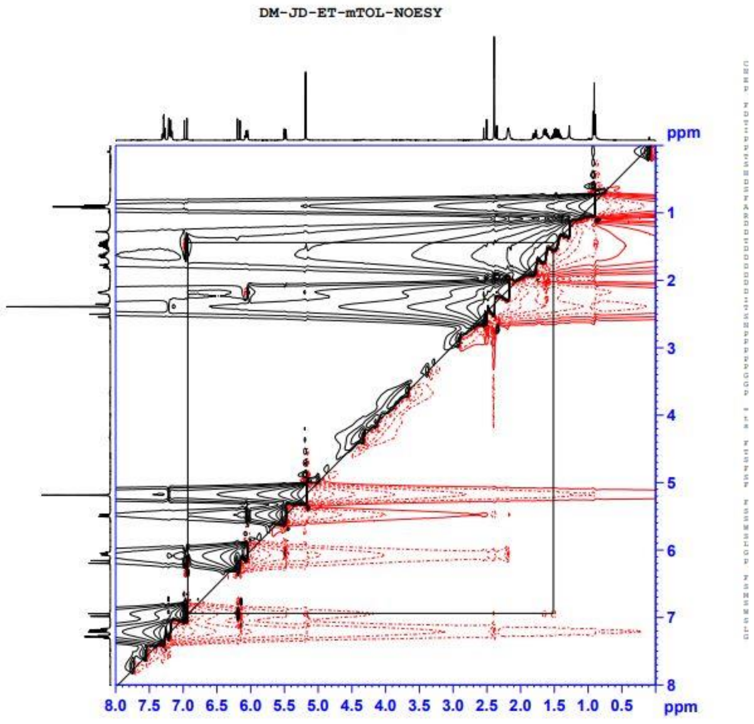

Compound 4p

(3a*S*,7a*R*)-7a-((*E*)-3-Oxobut-1-en-1-yl)-3a-propyl-3a,4,5,7a-tetrahydrobenzofuran-2(3H)-one

^{1}H NMR (400 MHz, $CDCl_3$)

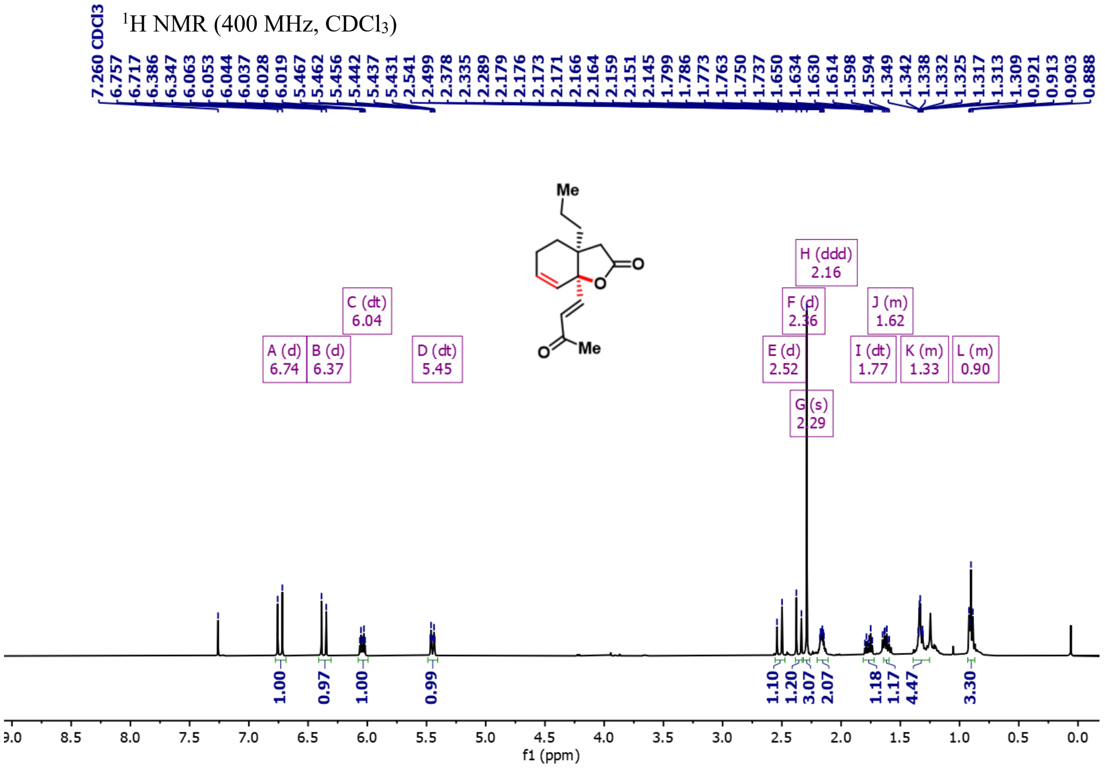

^{13}C NMR (101 MHz, $CDCl_3$)

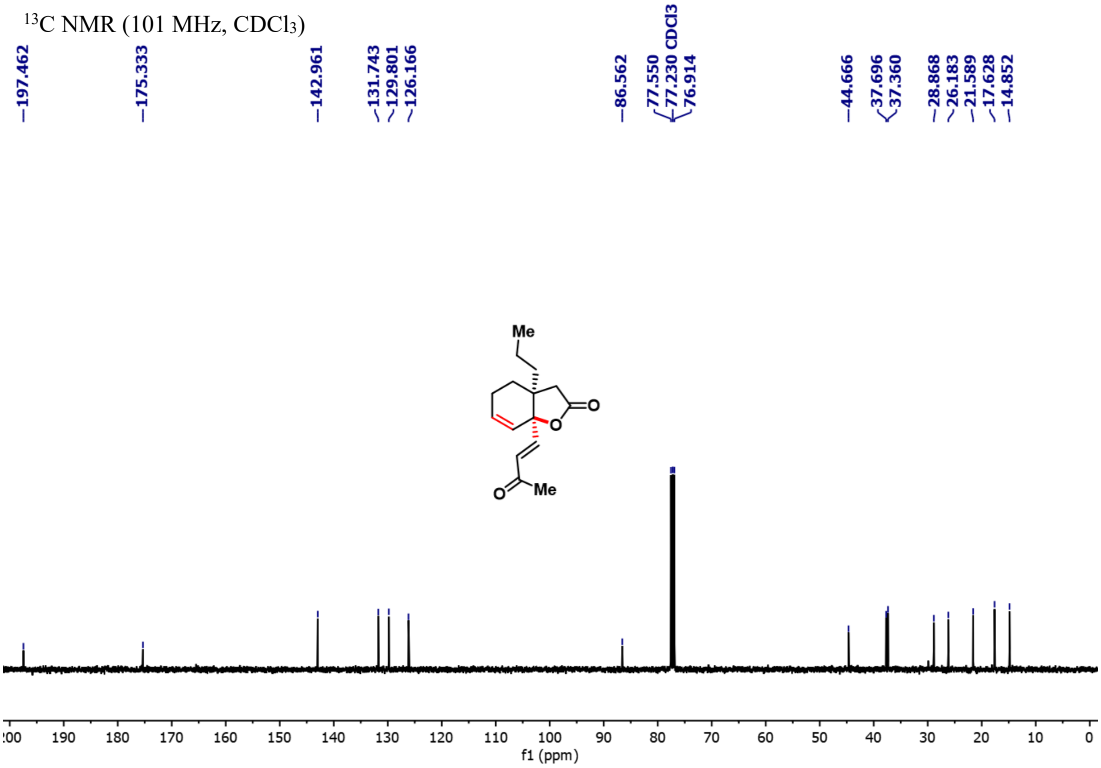

Compound 4q

(3a*S*,7a*R*)-3a-Methyl-7a-((*E*)-3-oxobut-1-en-1-yl)-3a,4,5,7a-tetrahydrobenzofuran-2(3H)-one

^{1}H NMR (400 MHz, $CDCl_3$)

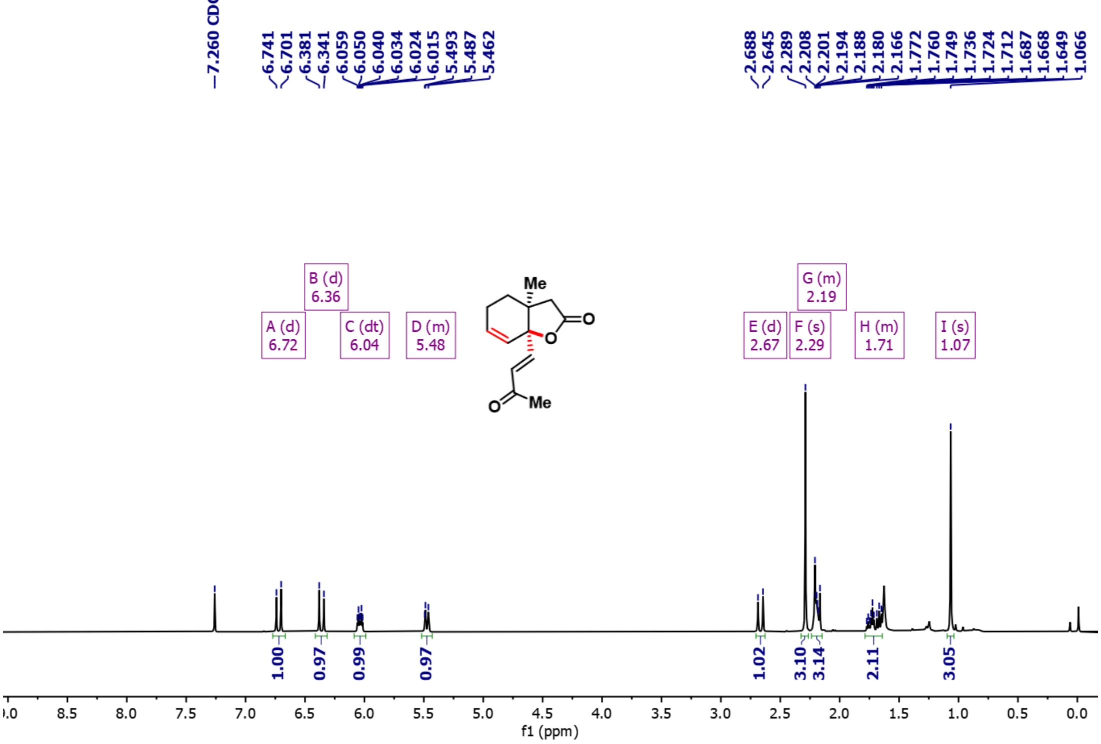

^{13}C NMR (101 MHz, $CDCl_3$)

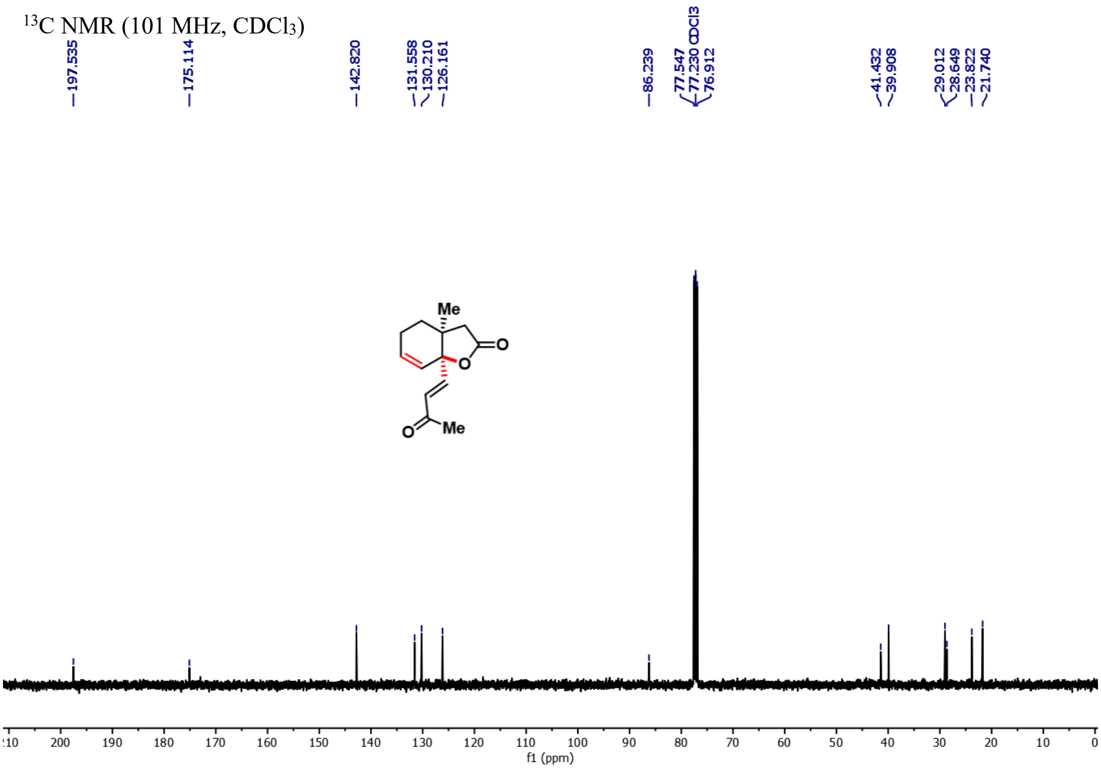

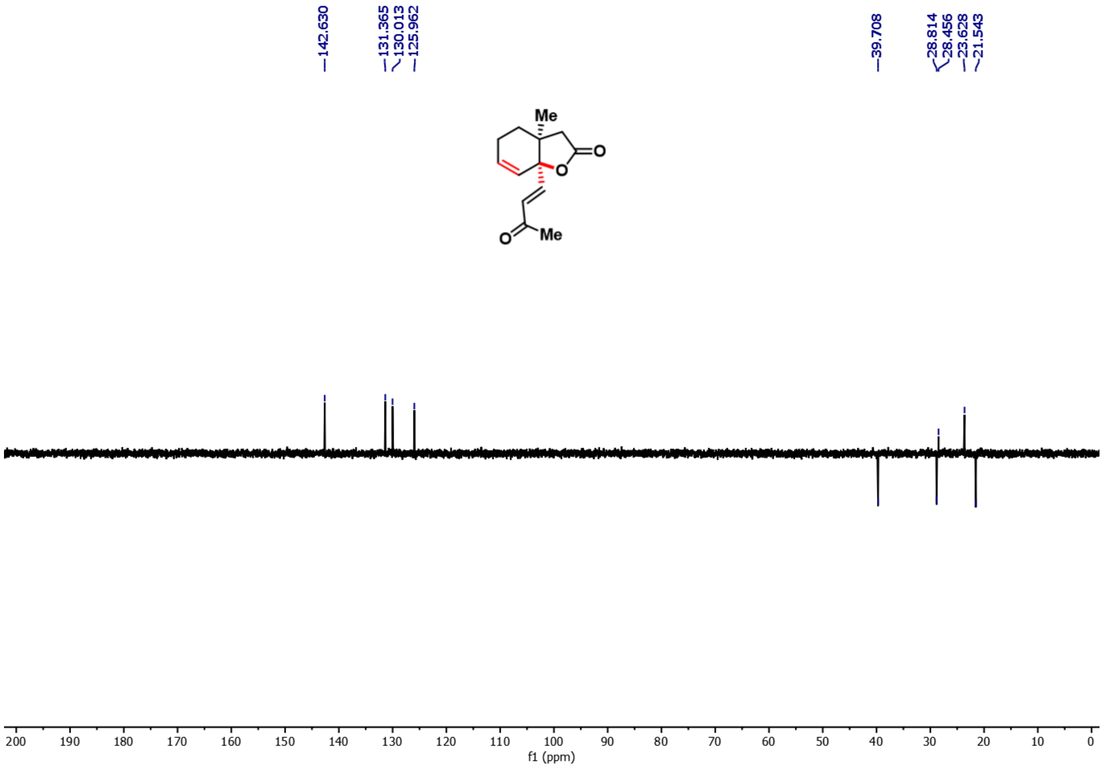

142.630
131.365
130.013
125.962
39.708
28.814
28.456
23.628
21.543
Me
O
O
O
Me
200 190 180 170 160 150 140 130 120 110 100 90 80 70 60 50 40 30 20 10 0
f1 (ppm)

Compound 4r

(3a*S*,7a*R*)-3a-Cyclohexyl-7a-((*E*)-3-oxobut-1-en-1-yl)-3a,4,5,7a-tetrahydrobenzofuran-2(3H)-one

^{1}H NMR (400 MHz, $CDCl_3$)

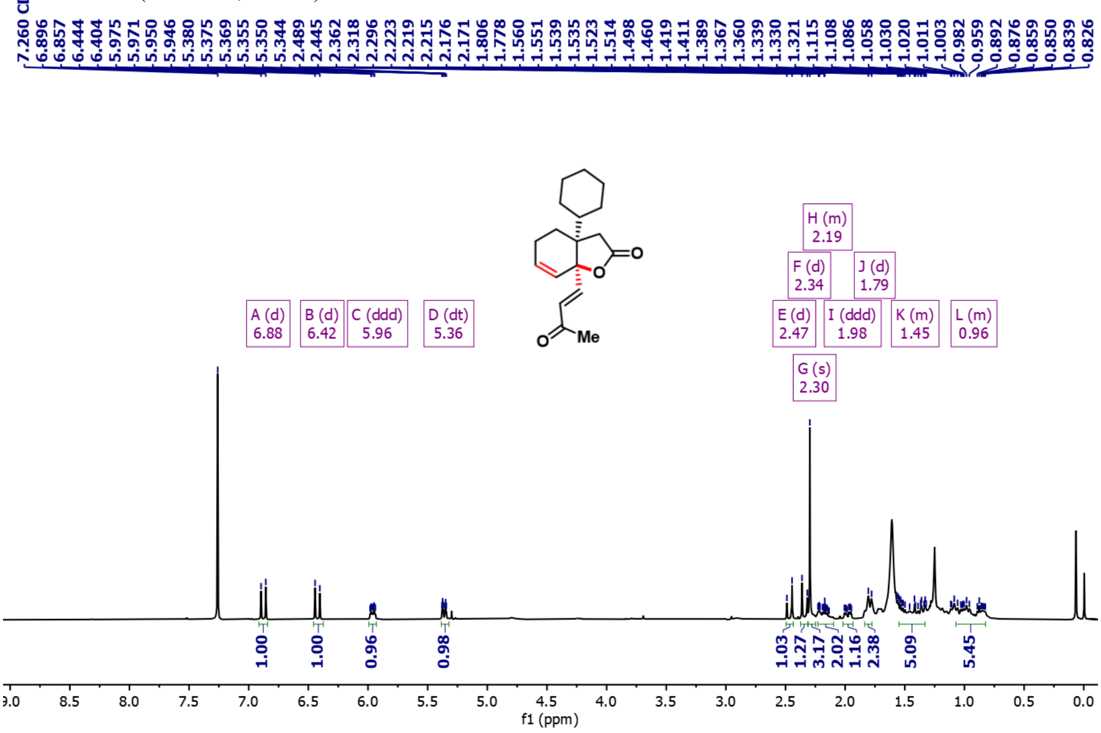

^{13}C NMR (101 MHz, $CDCl_3$)

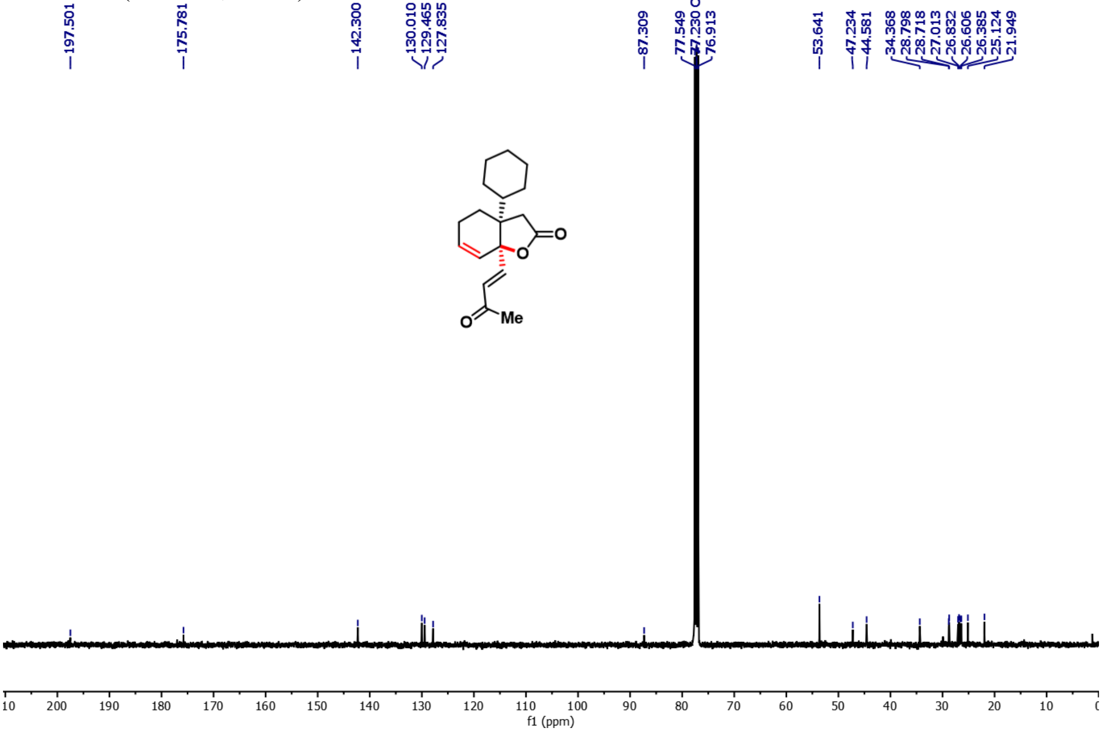

Compound 4s

(3a*S*,7a*S*)-6-(*tert*-Butyl)-3a-methyl-7a-((*E*)-3-oxobut-1-en-1-yl)-3a,4,5,7a-tetrahydrobenzofuran-2(3H)-one

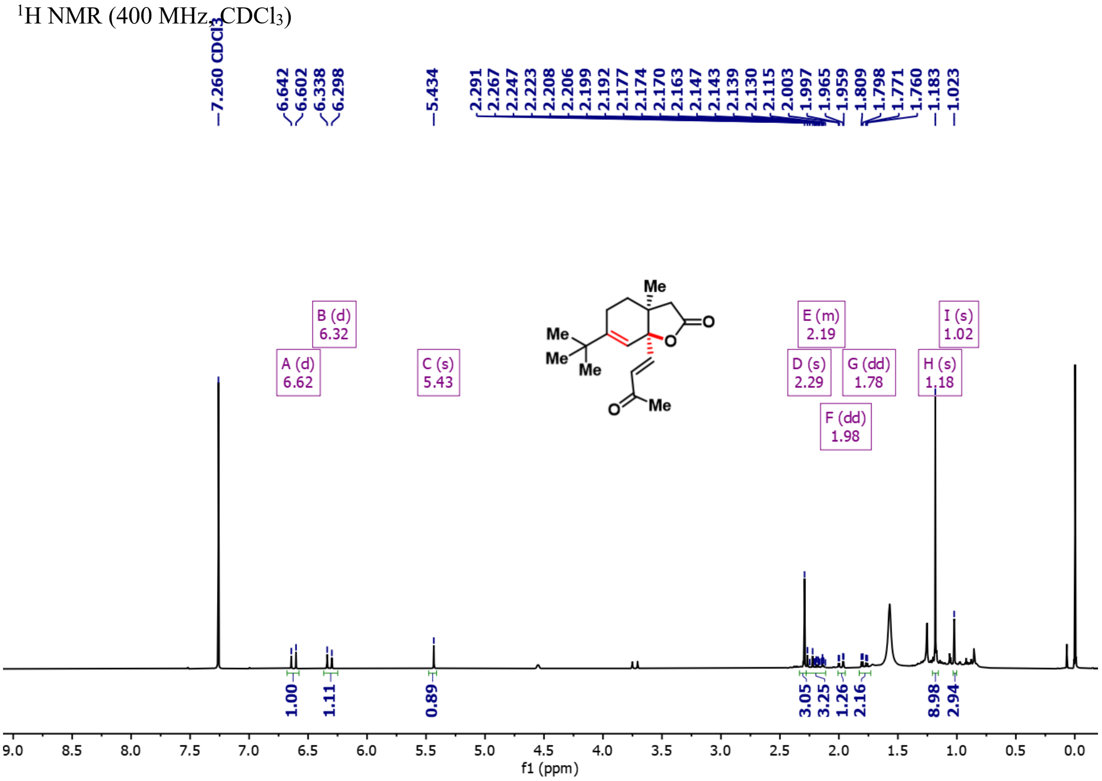

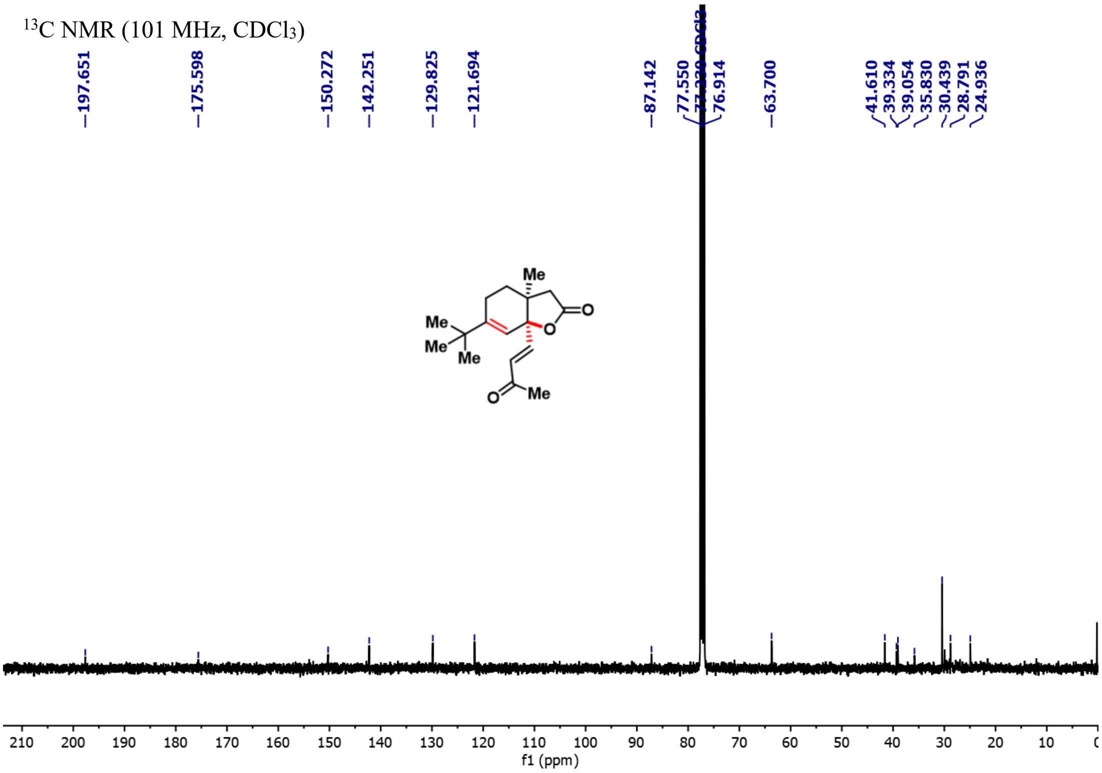

Compound 4t

(3a*R*,7a*R*)-3a,4-Dimethyl-7a-((*E*)-3-oxobut-1-en-1-yl)-3a,4,5,7a-tetrahydrobenzofuran-2(3H)-one

^{1}H NMR (500 MHz, CDCl$_3$)

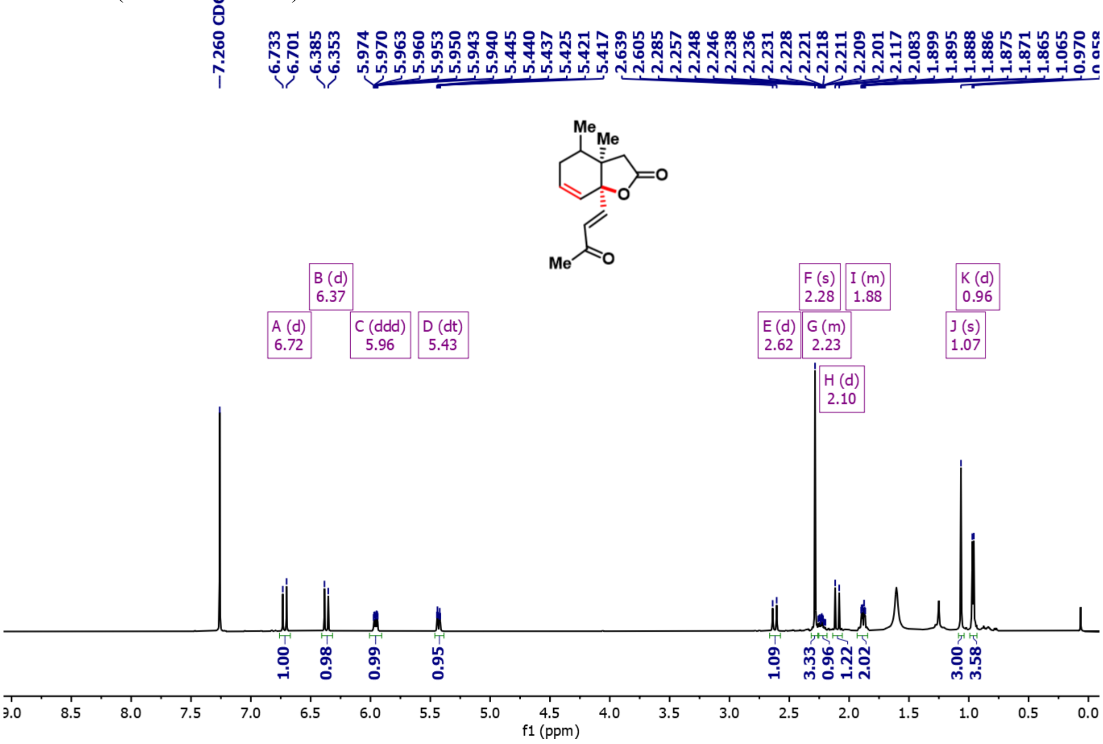

^{13}C NMR (126 MHz, CDCl$_3$)

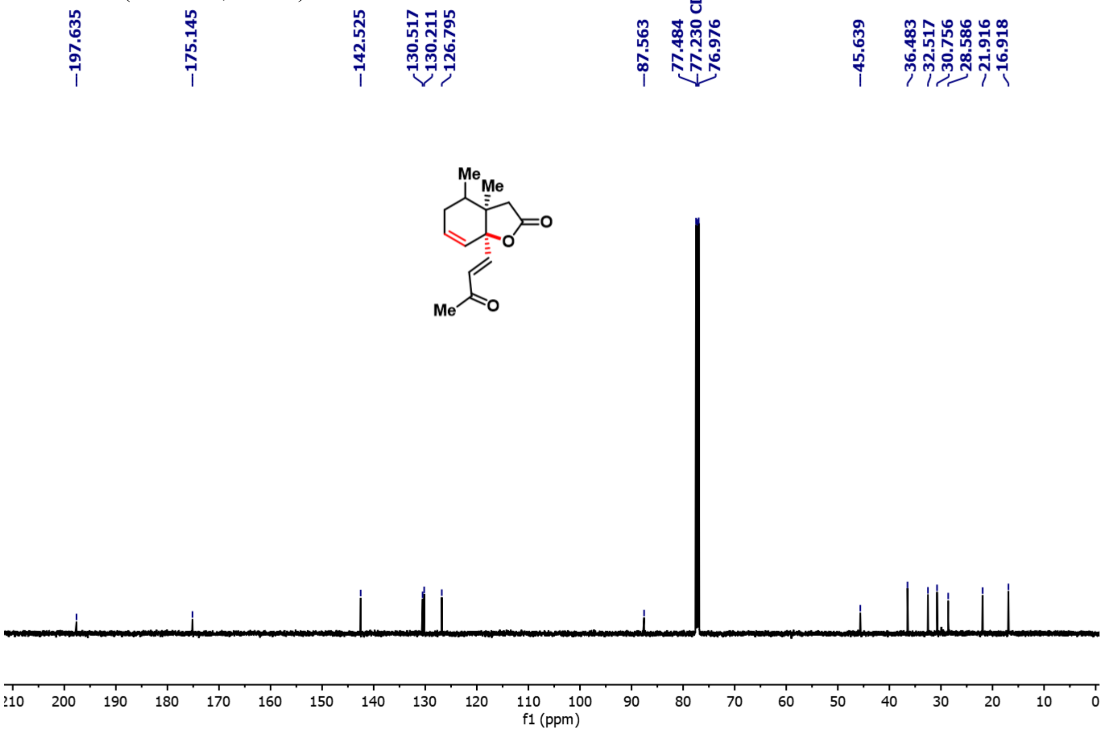

NOE Experiment:

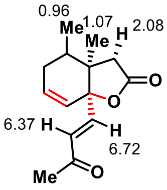

Me (1.07) $\xrightarrow{\text{Strong NOE}}$ H (6.72)

Me (1.07) $\xrightarrow{\text{Strong NOE}}$ H (2.10)

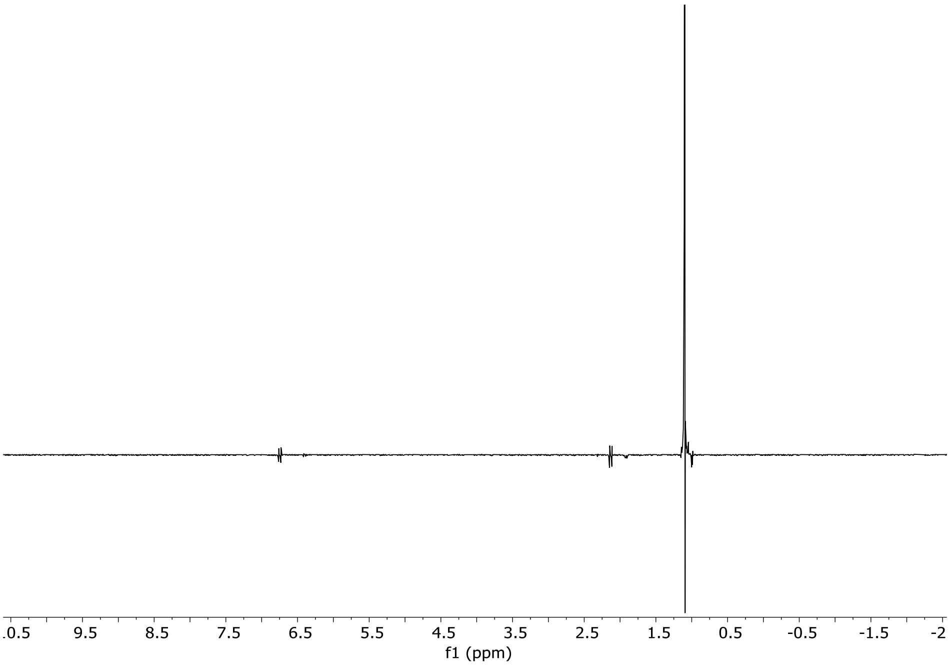

COSY Experiment:

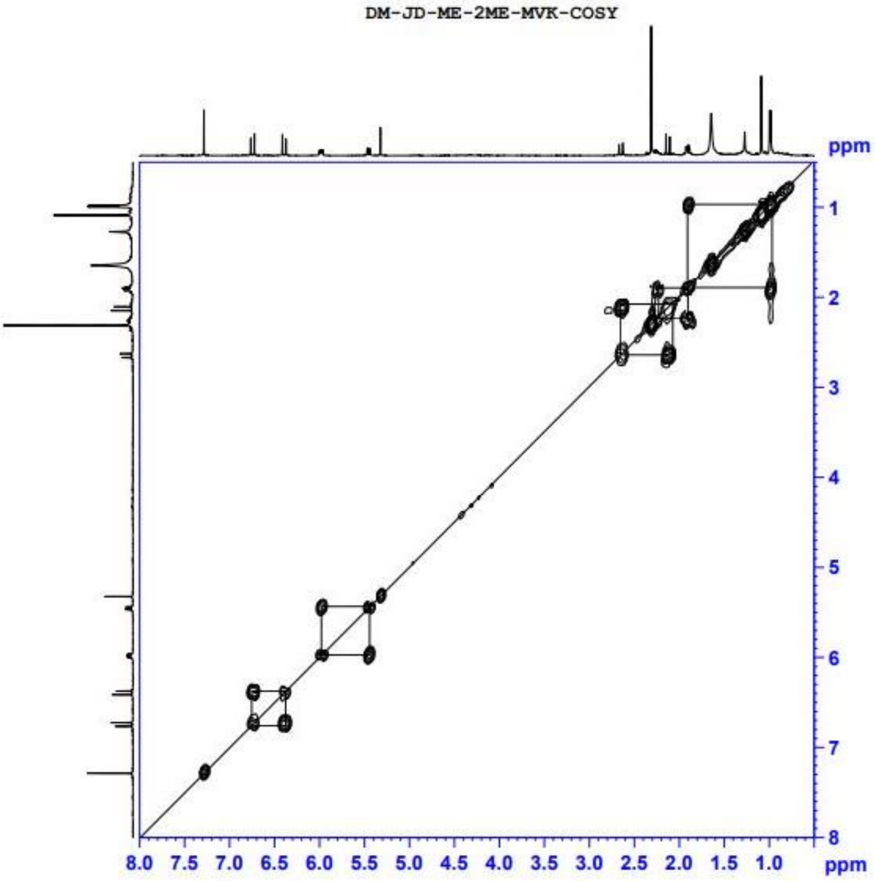

NOESY Experiment:

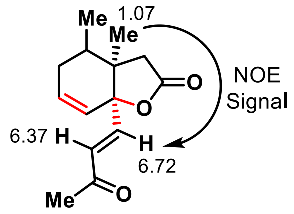

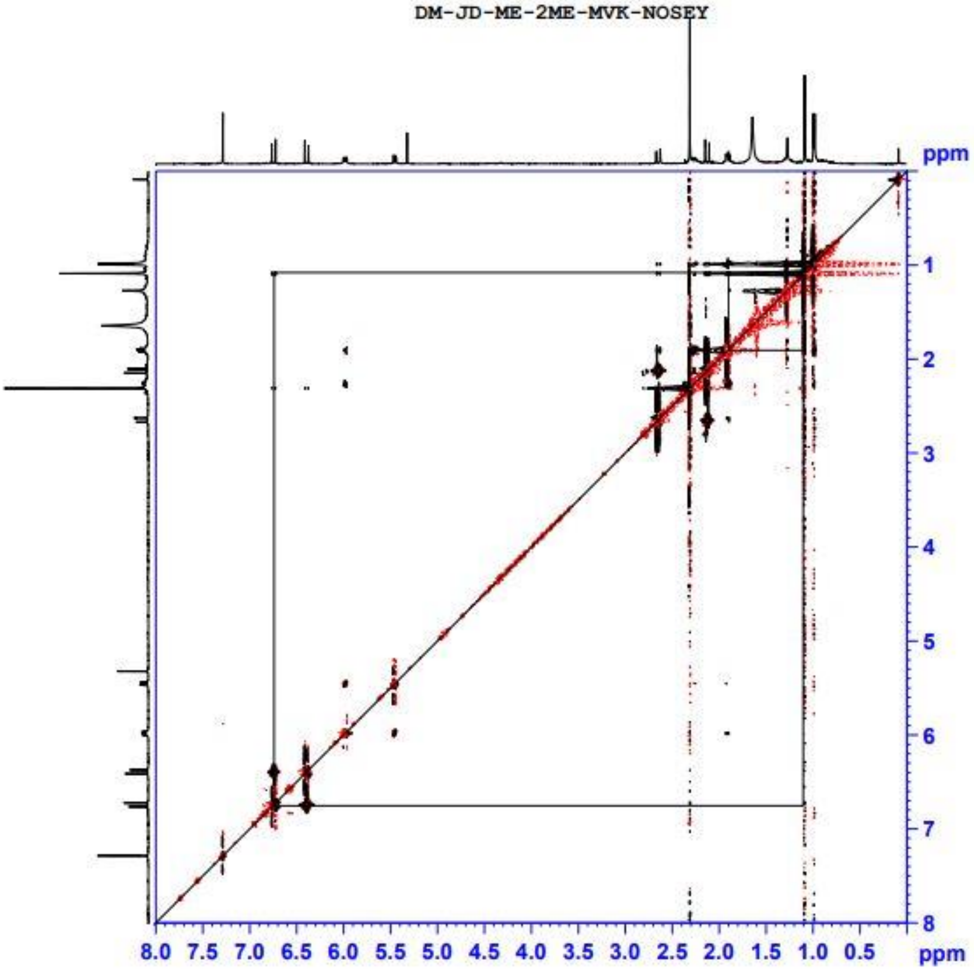

Compound 4u

(3a*S*,7a*R*)-3a,5-Dimethyl-7a-((*E*)-3-oxobut-1-en-1-yl)-3a,4,5,7a-tetrahydrobenzofuran-2(3H)-one

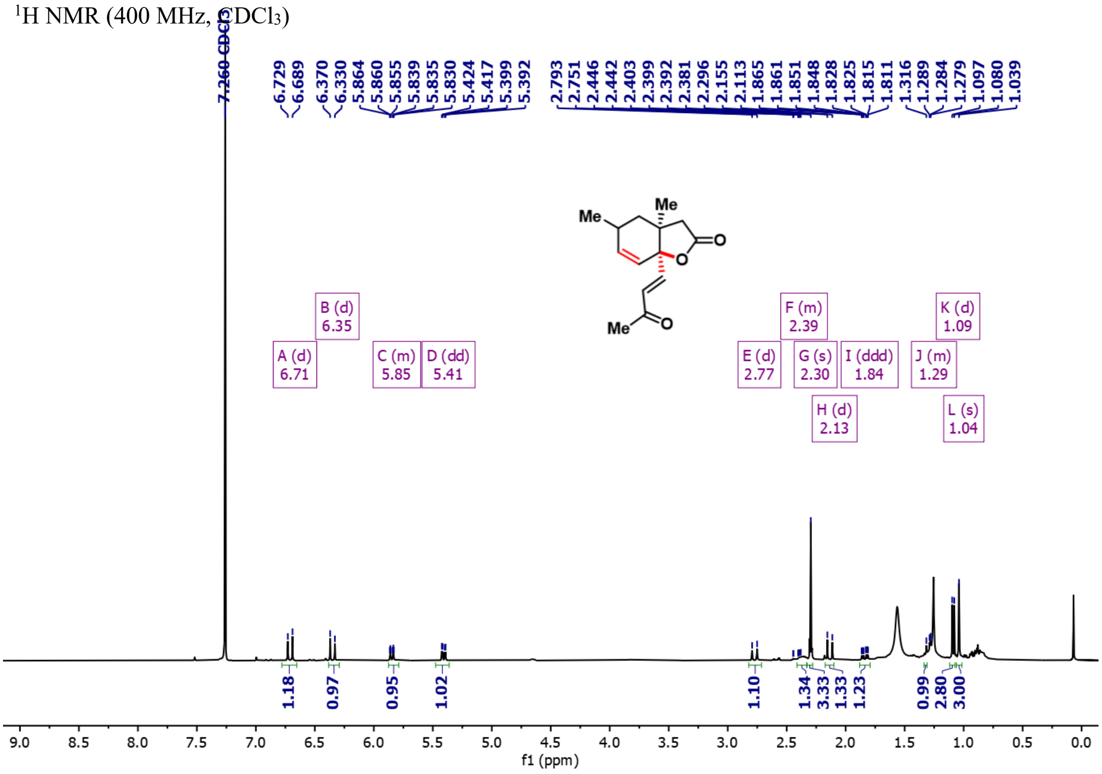

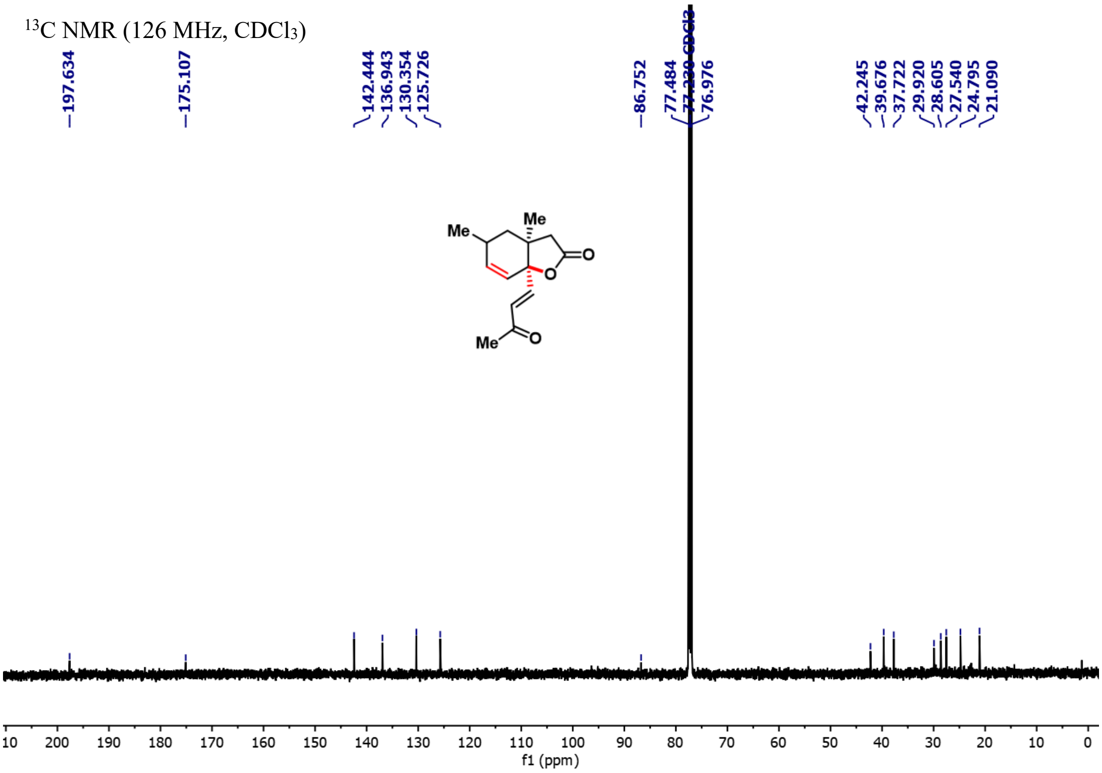

Compound 4v

(3a*S*,7a*R*)-3a-Methyl-7a-((*E*)-3-oxopent-1-en-1-yl)-3a,4,5,7a-tetrahydrobenzofuran-2(3H)-one

^{1}H NMR (500 MHz, CDCl3)

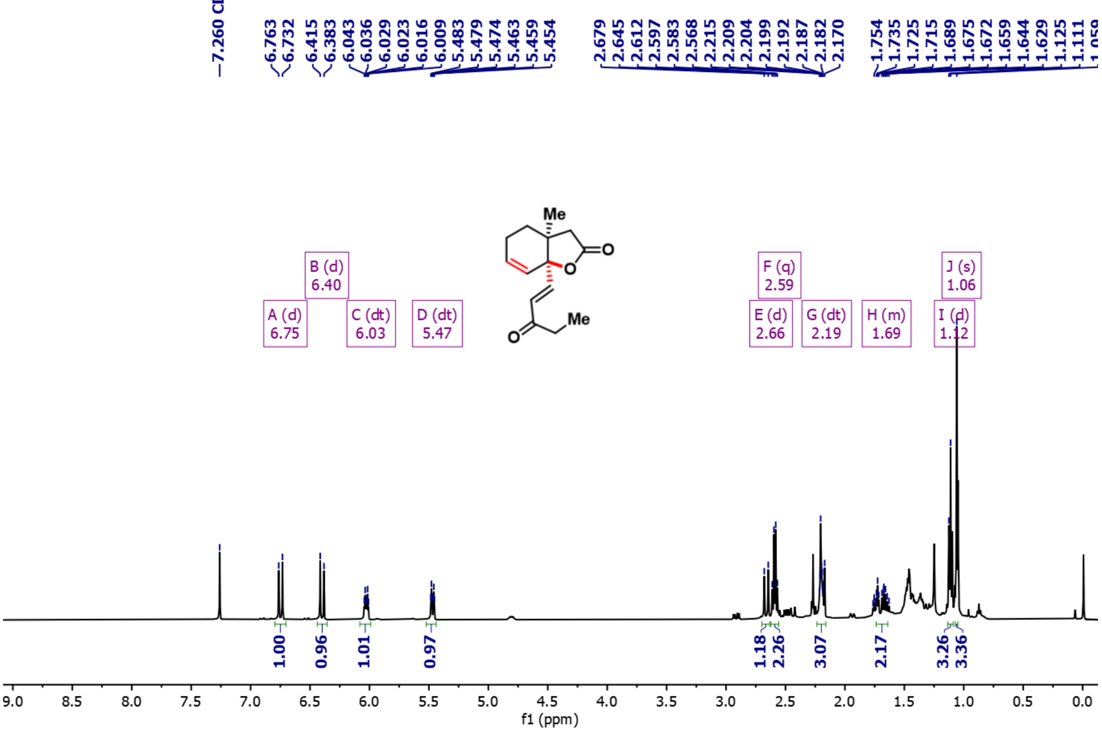

^{13}C NMR (126 MHz, CDCl3)

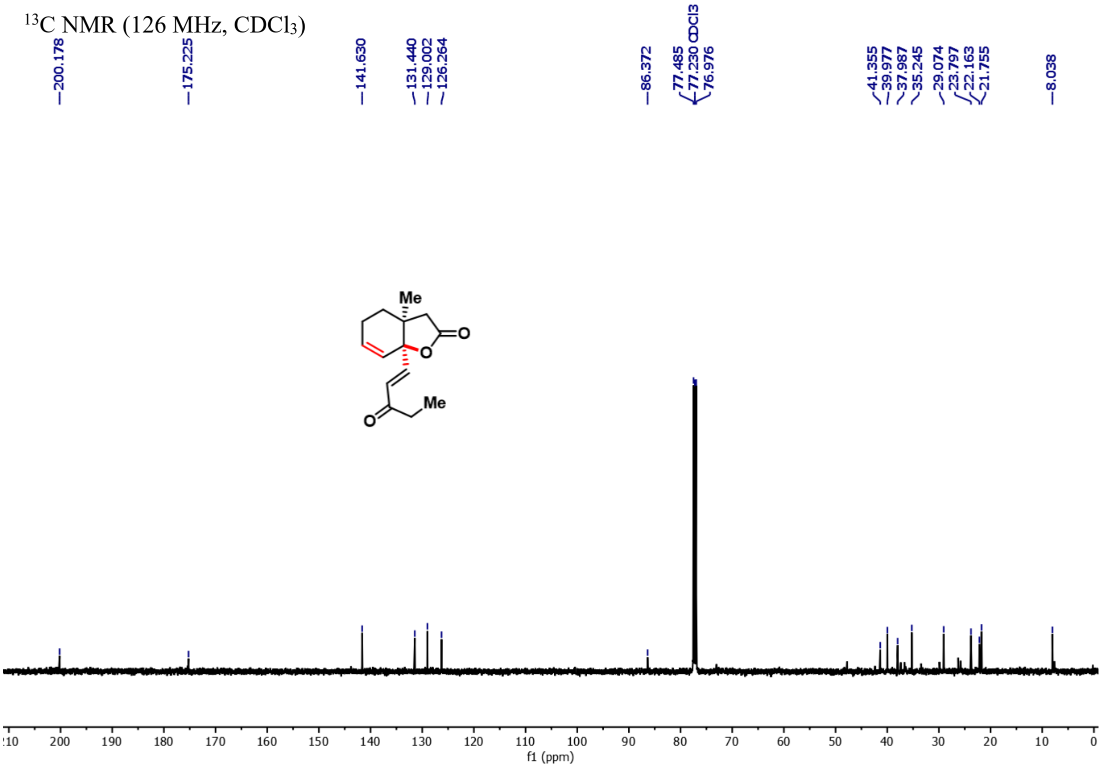

Compound 4w

(*E*)-3-((3a*S*,7a*R*)-3a-Methyl-2-oxo-3,3a,4,5-tetrahydrobenzofuran-7a(2H)-yl)acrylaldehyde

^{1}H NMR (400 MHz, $CDCl_3$)

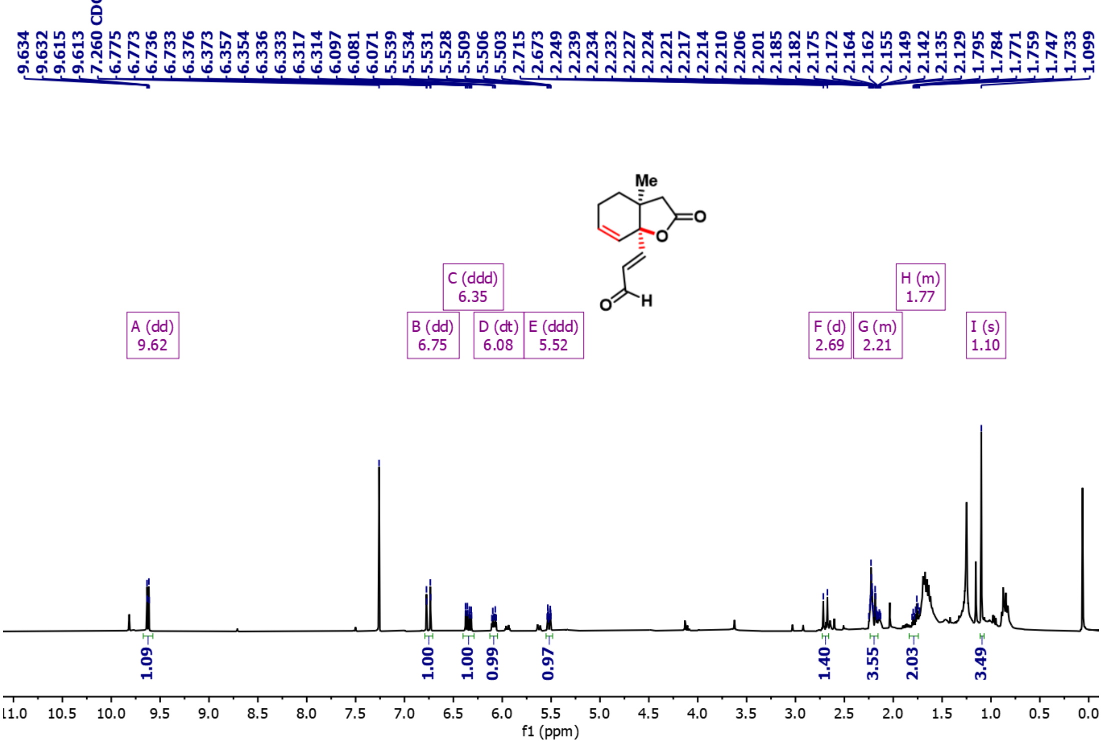

^{13}C NMR (101 MHz, $CDCl_3$)

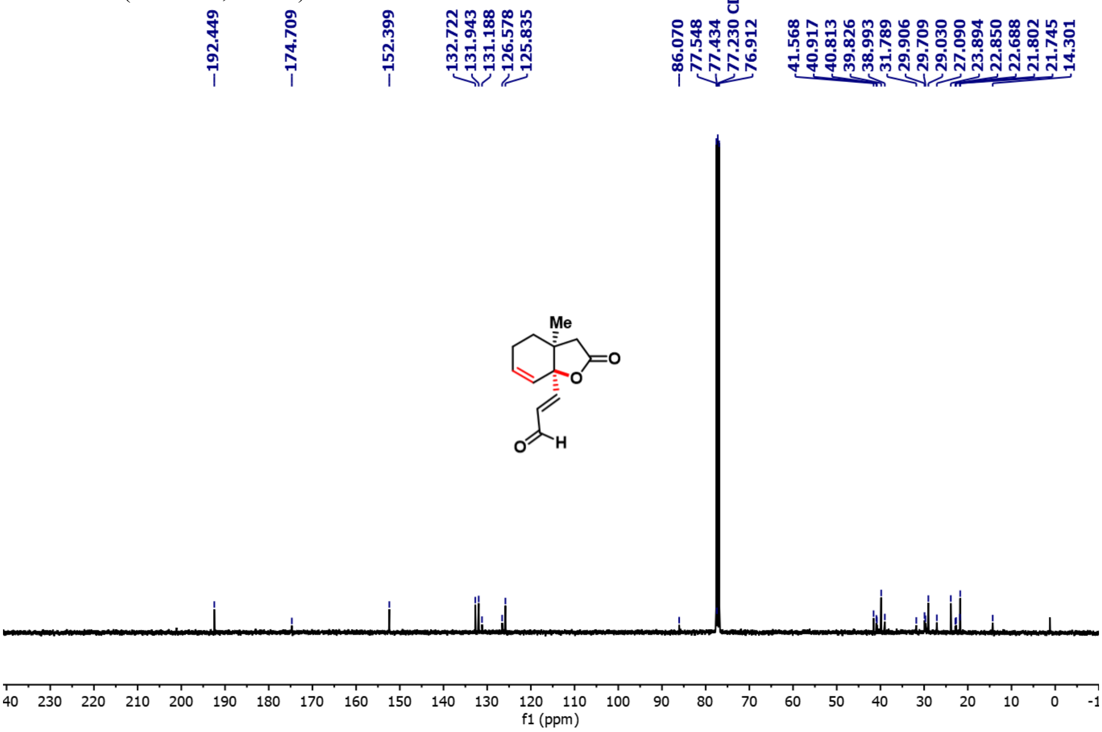

Compound 4x

(*E*)-3-((3a*S*,7a*R*)-3a-Cyclohexyl-2-oxo-3,3a,4,5-tetrahydrobenzofuran-7a(2H)-yl)acrylaldehyde

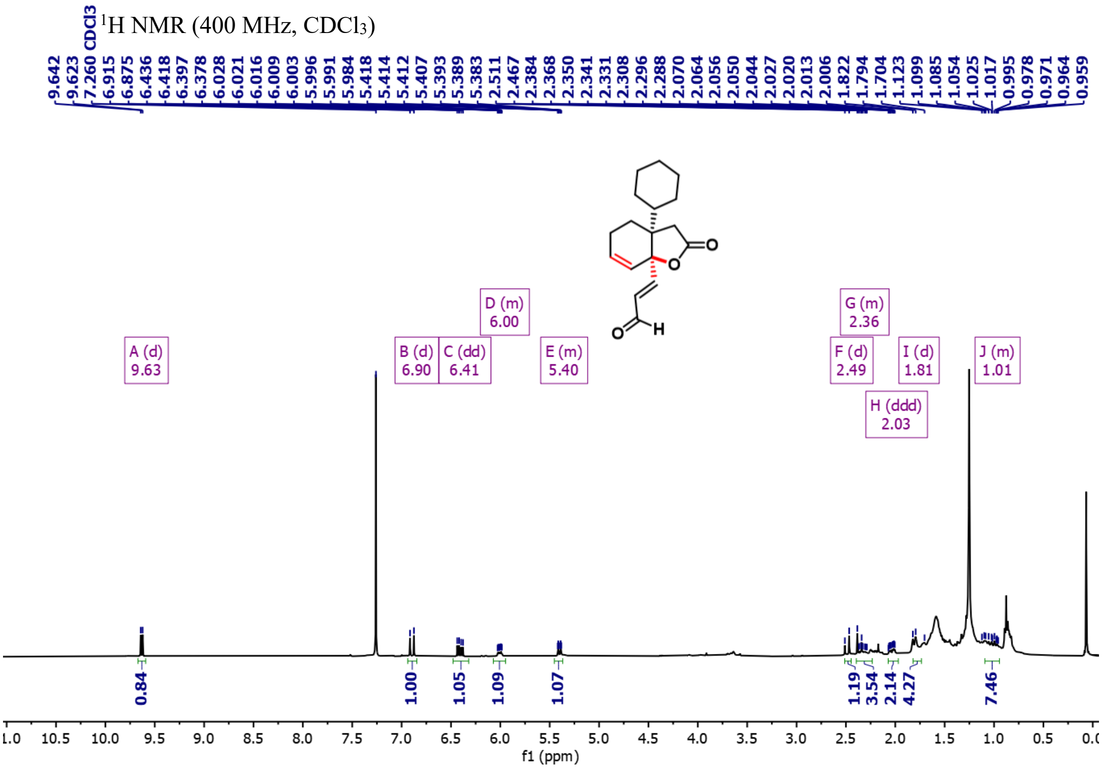

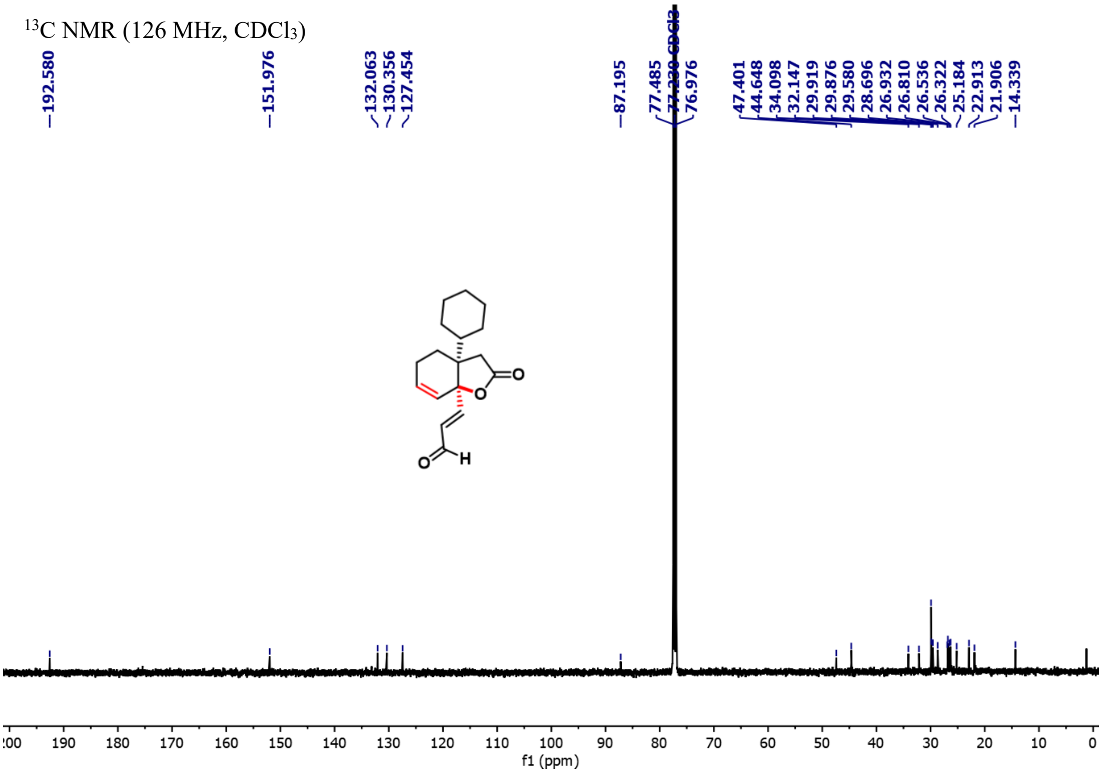

Compound 4y

(3a*S*,7a*R*)-3a-Methyl-7a-((*E*)-3-oxooct-1-en-1-yl)-3a,4,5,7a-tetrahydrobenzofuran-2(3H)-one

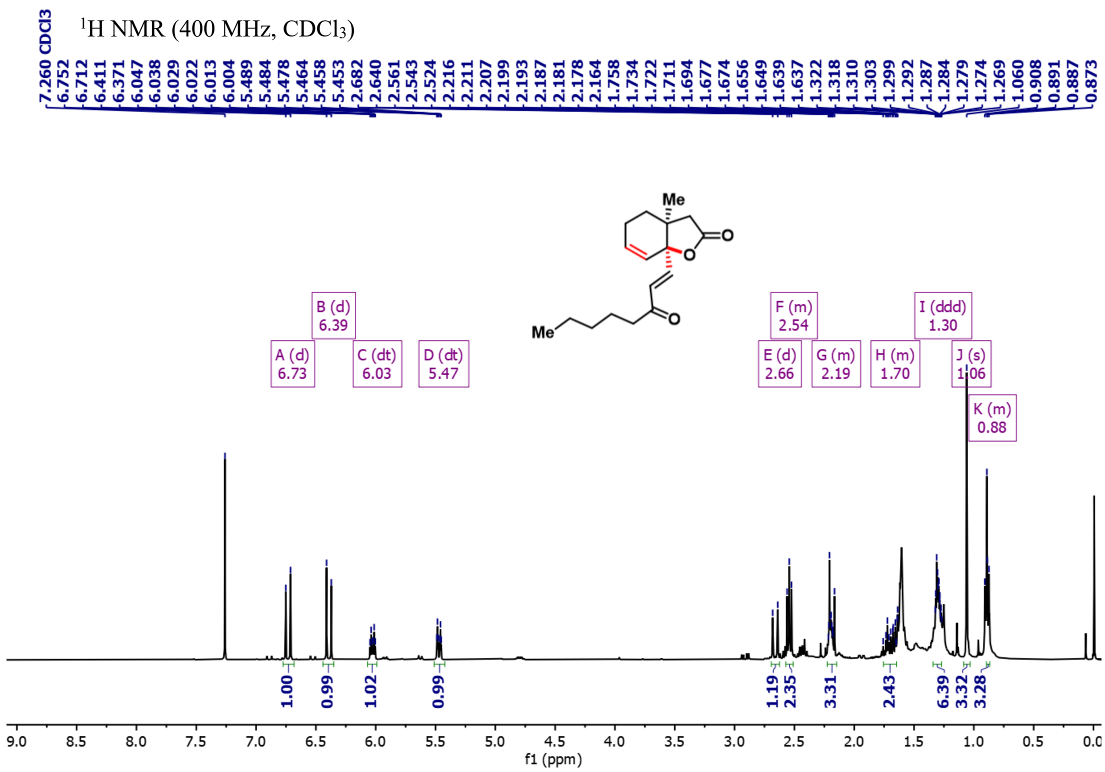

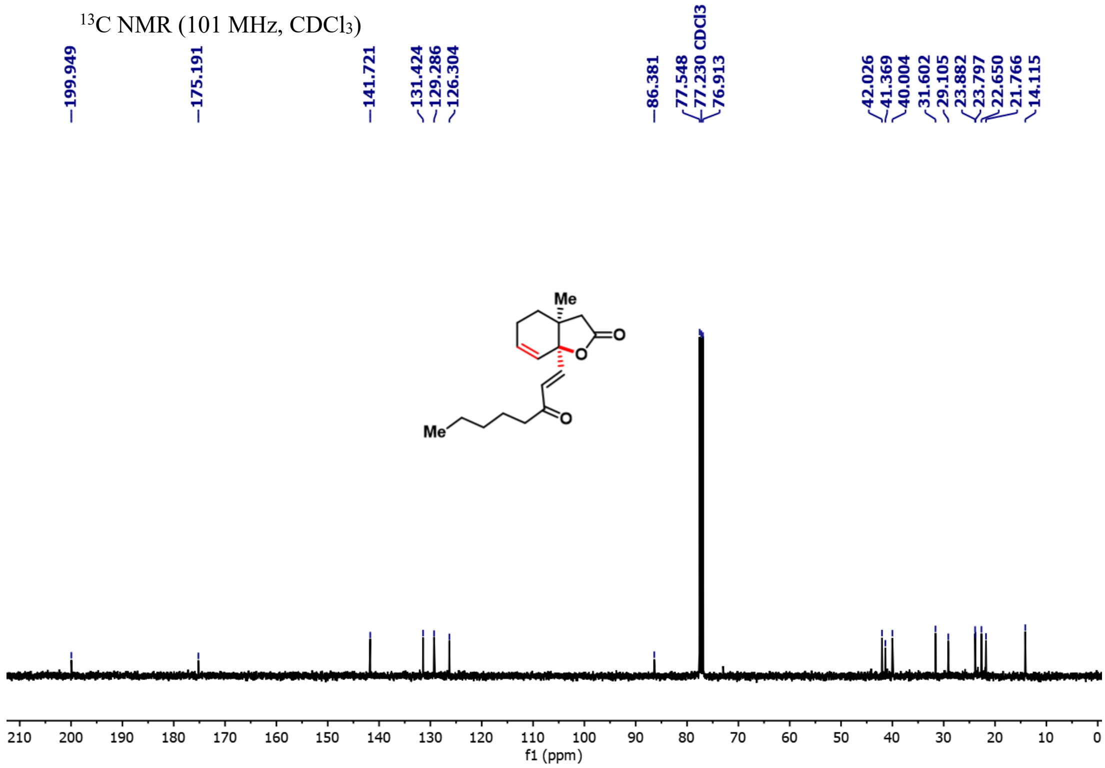

Compound 4z

(3a*S*,7a*S*)-6-(*tert*-Butyl)-7a-((*E*)-3-hydroxy-3-methylbut-1-en-1-yl)-3a-methyl-3a,4,5,7a-tetrahydrobenzofuran-2(3H)-one

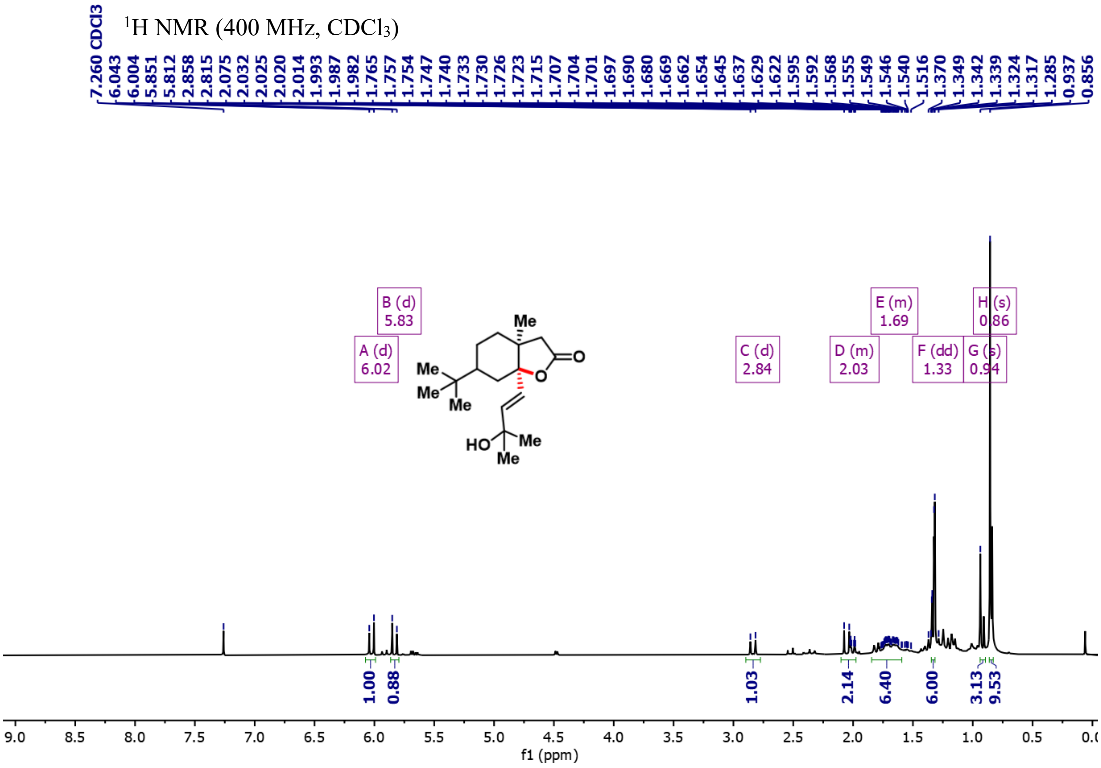

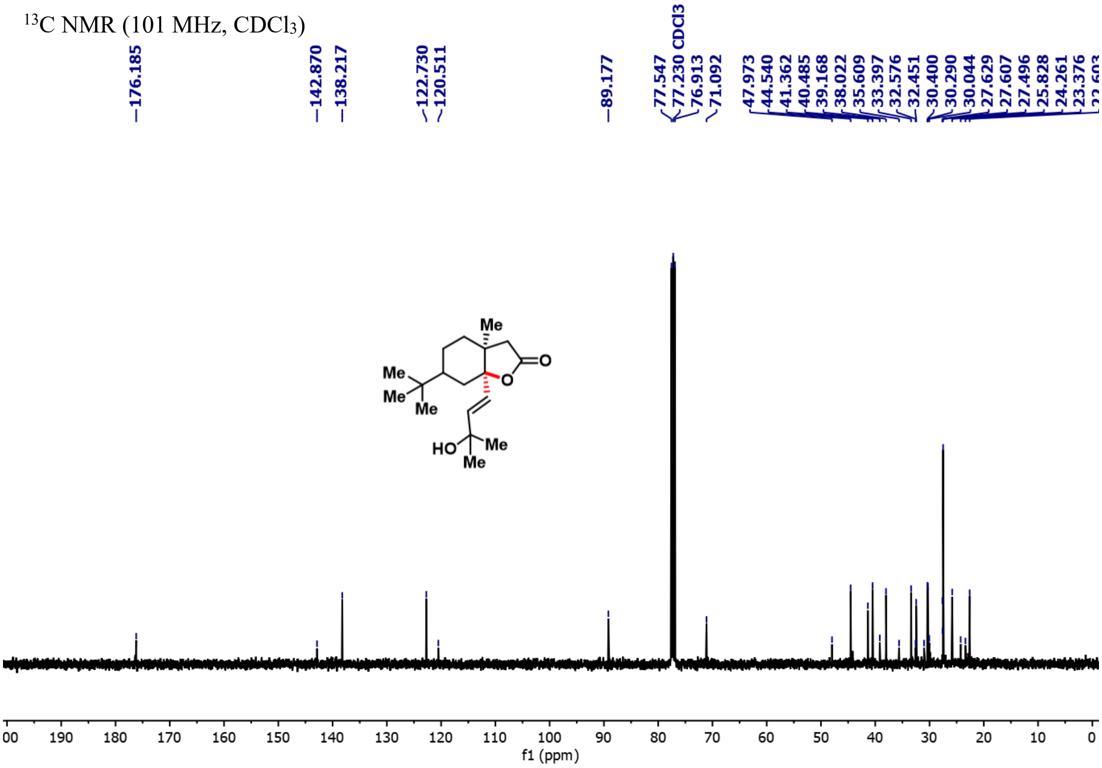

Compound 5a

(3a*S*,7a*S*)-3a-(3-Methoxyphenyl)hexahydrobenzofuran-2(3H)-one

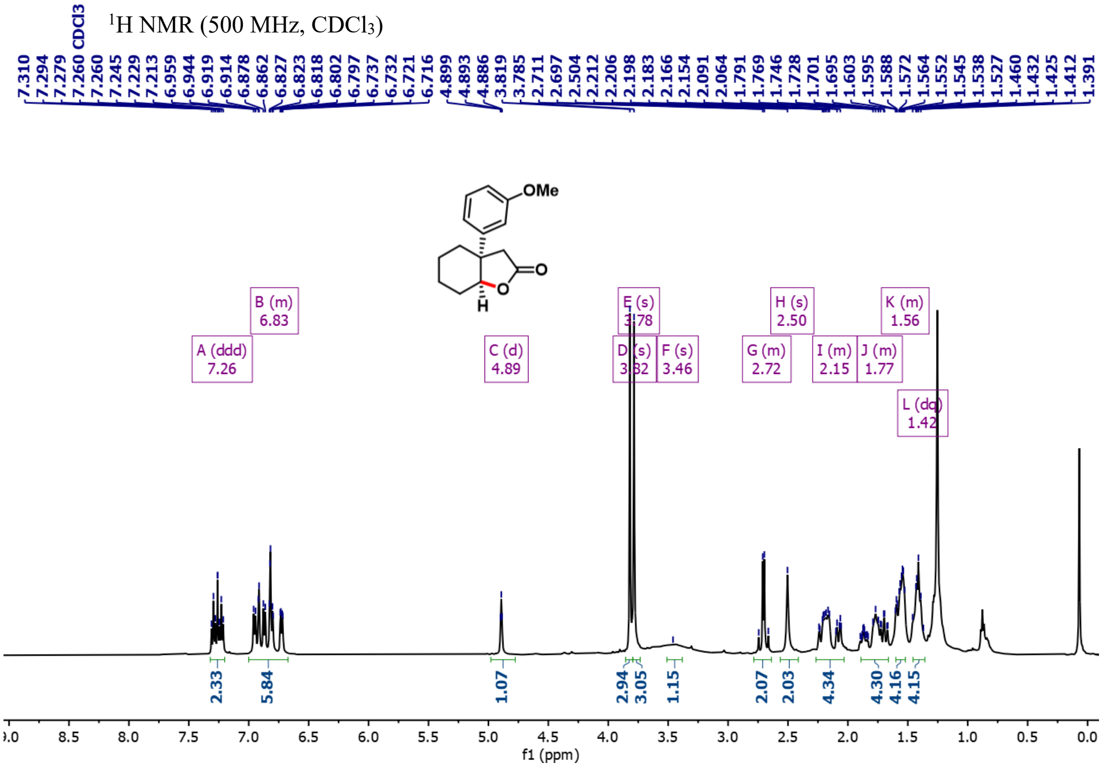

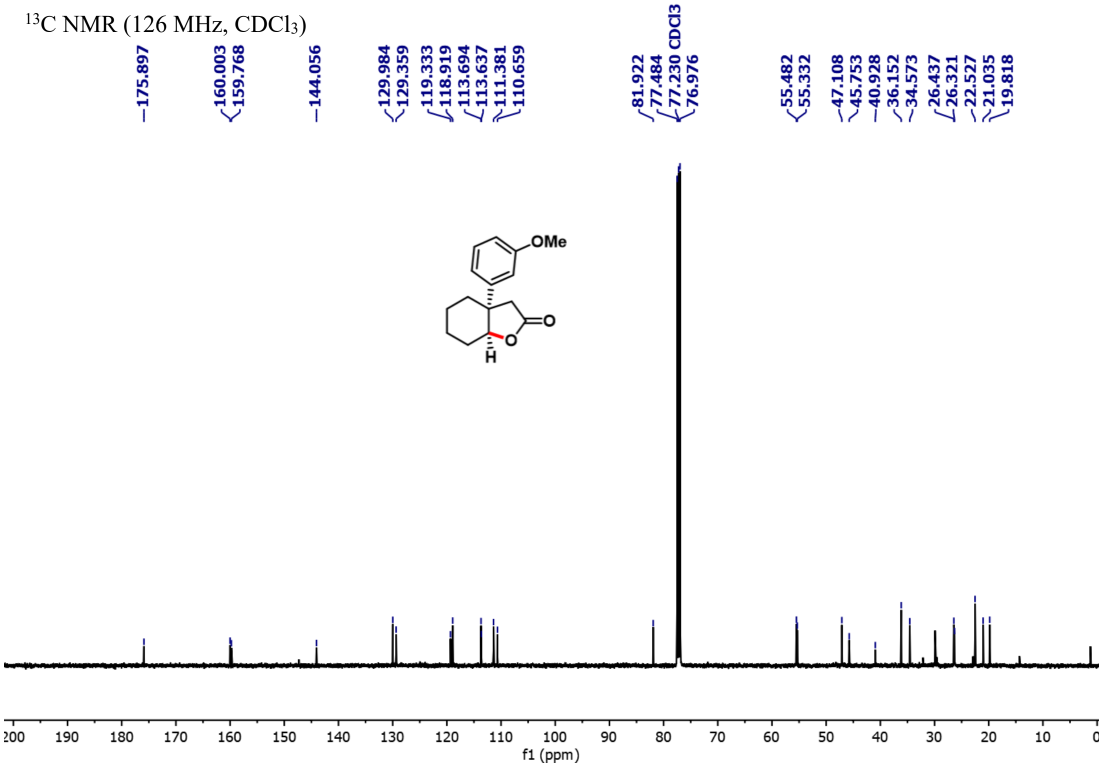

Compound 5b

(3a*S*,7a*S*)-3a-(3,4-Dimethoxyphenyl)hexahydrobenzofuran-2(3H)-one

^{1}H NMR (500 MHz, CDCl3)

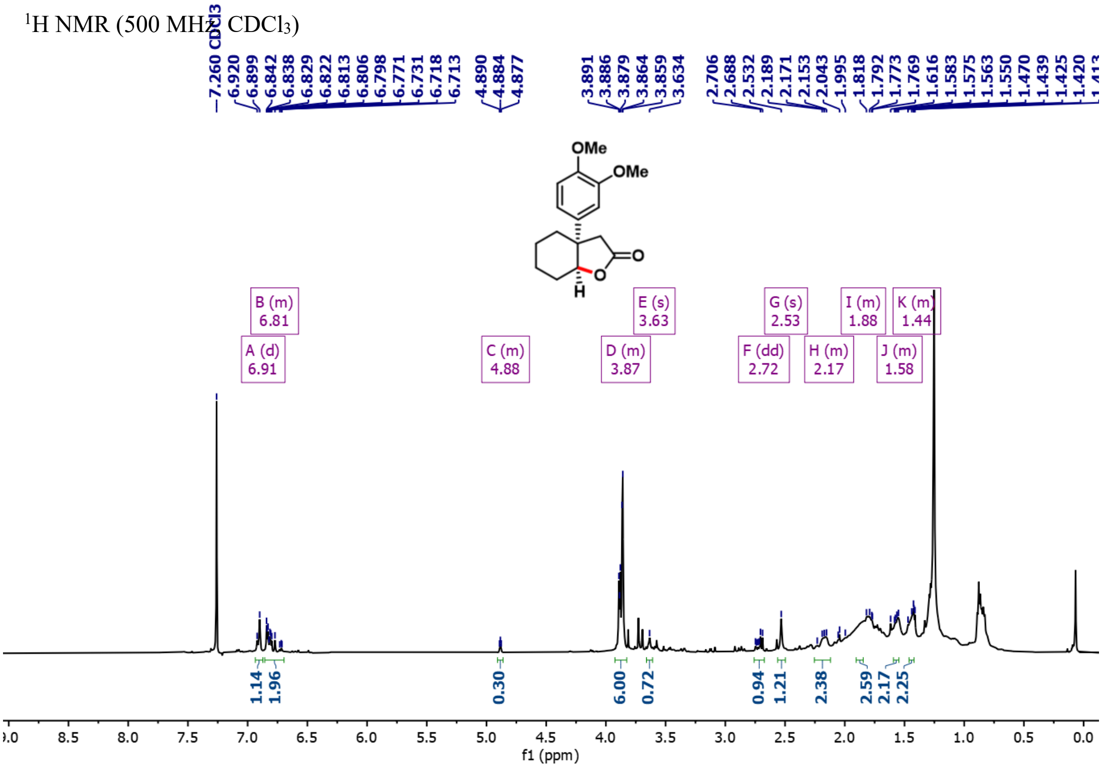

^{13}C NMR (126 MHz, CDCl3)

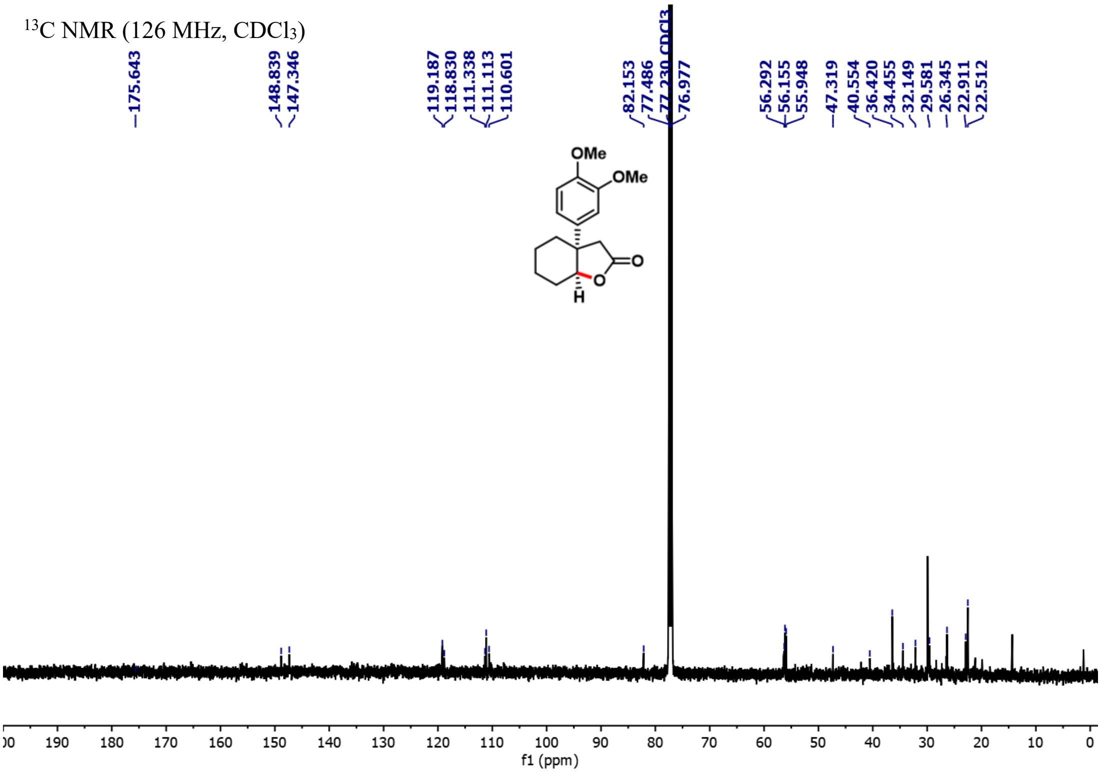

Compound 5c

(1*S*,6*S*)-6-(2-Hydroxyethyl)-6-methylcyclohex-2-en-1-ol

^{1}H NMR (400 MHz, CDCl$_3$)

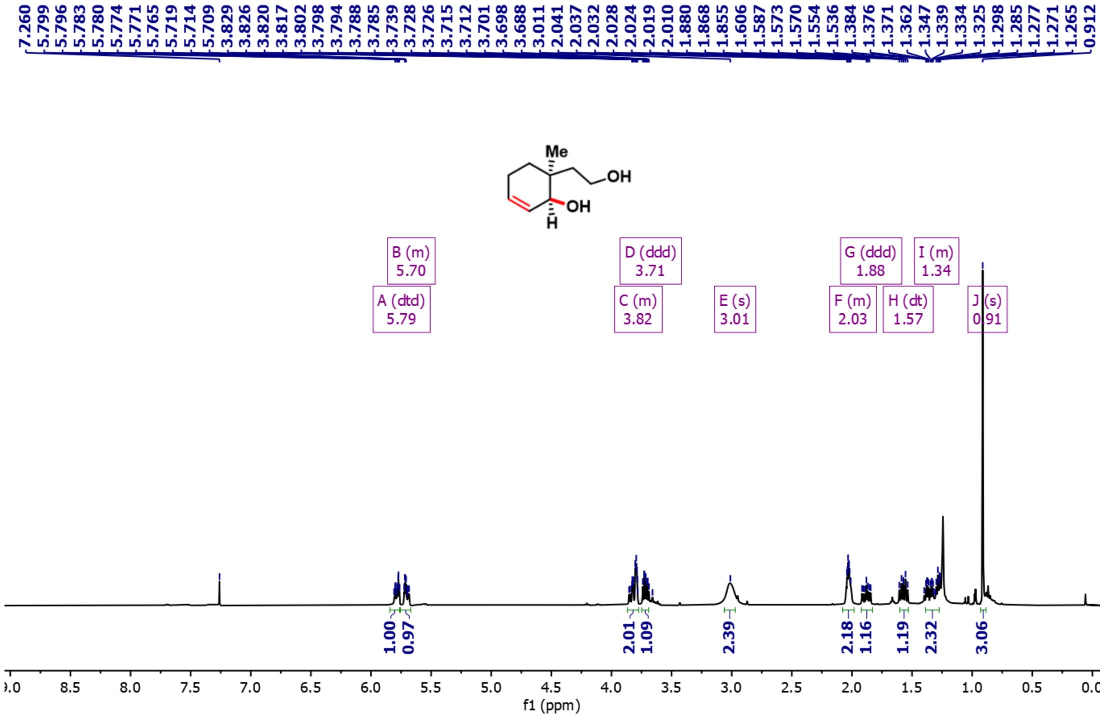

^{13}C NMR (101 MHz, CDCl$_3$)

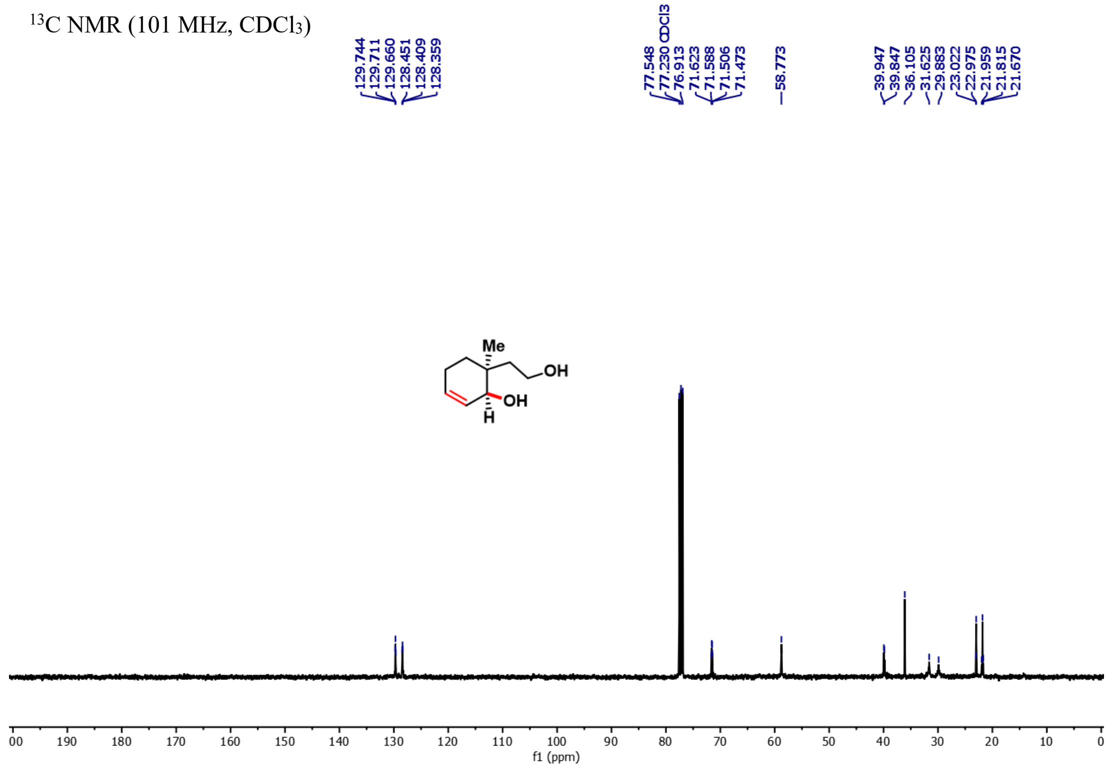

Compound 5d

(3a*S*,6*S*,7*S*,7a*R*)-6,7-Dihydroxy-3a-methylhexahydrobenzofuran-2(3H)-one

^{1}H NMR (400 MHz, $CDCl_3$)

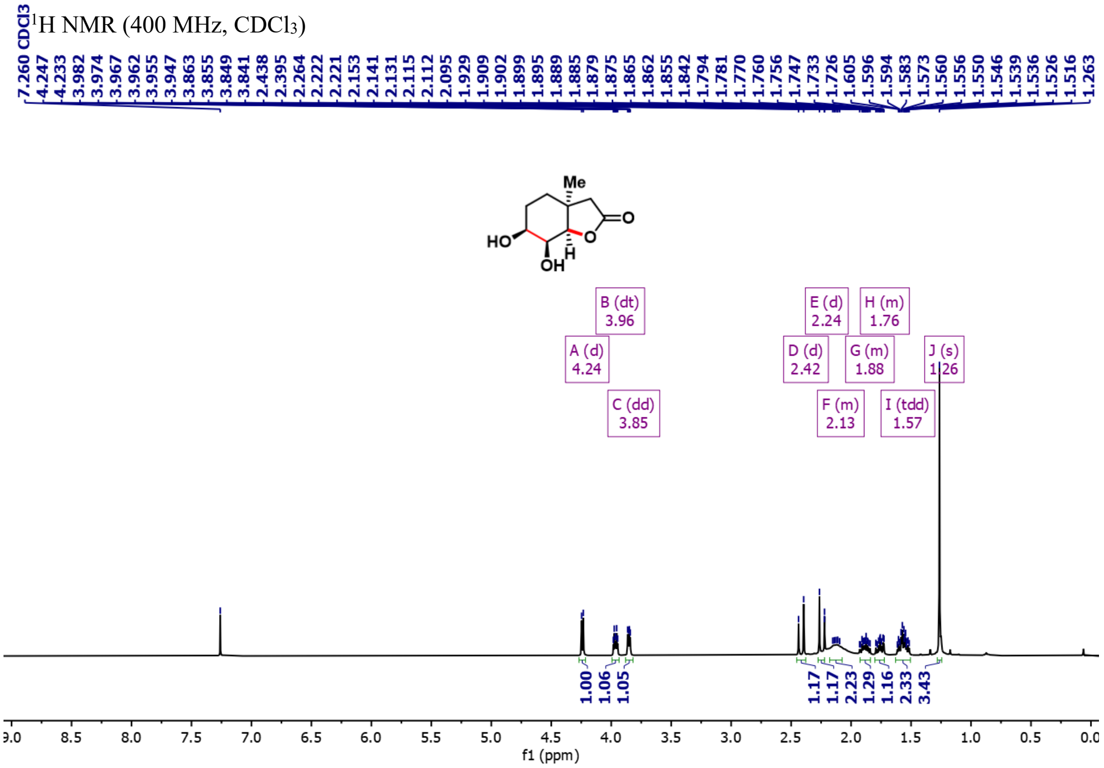

^{13}C NMR (101 MHz, $CDCl_3$)

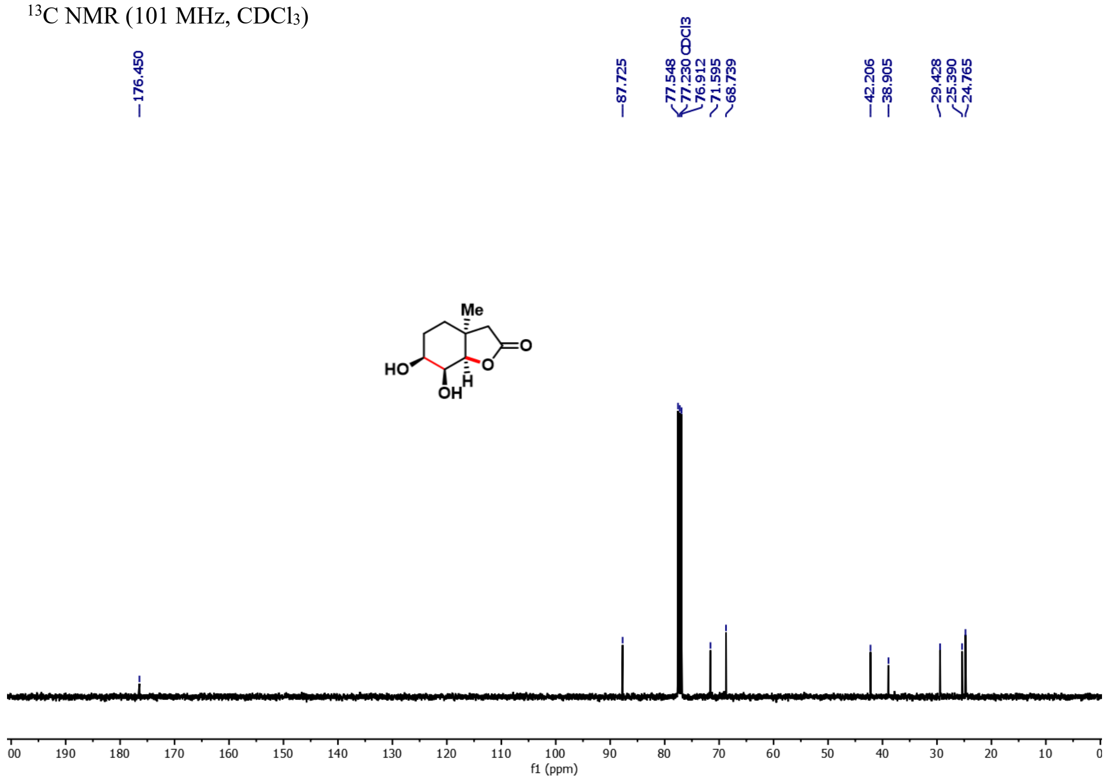

Compound 5e

(3a*S*,7a*S*)-3a-Octyl-3a,7a-dihydrobenzofuran-2,5(3H,4H)-dione

^{1}H NMR (500 MHz, $CDCl_3$)

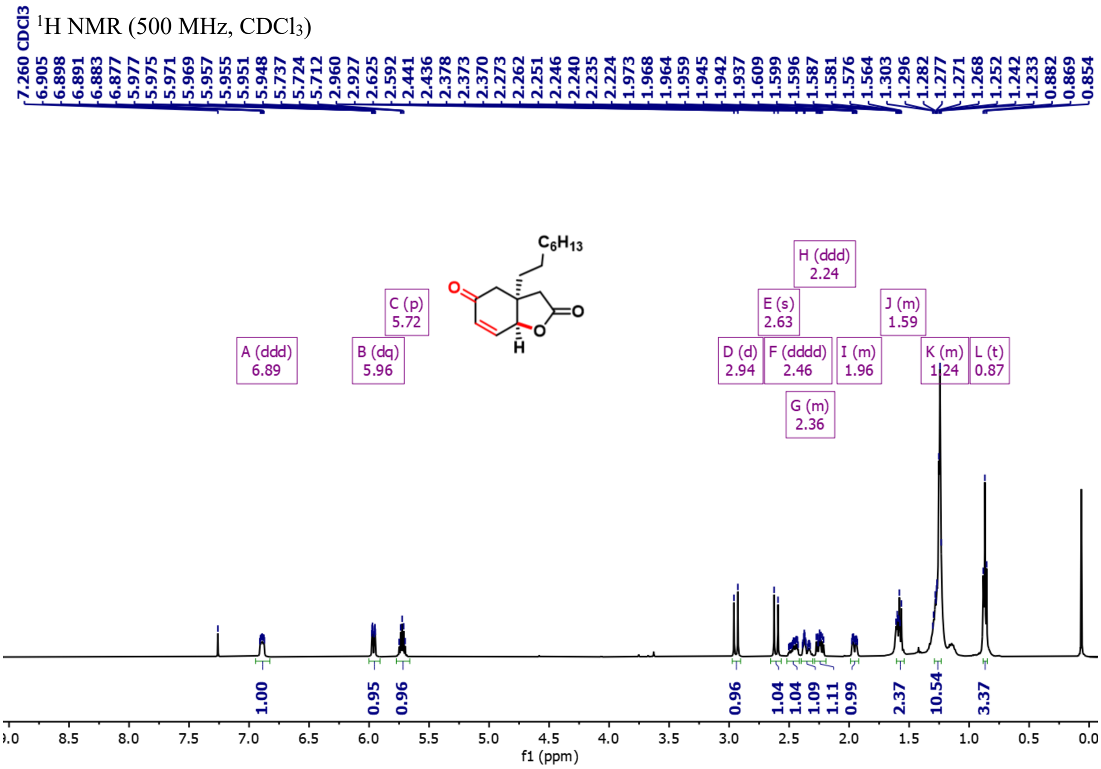

^{13}C NMR (126 MHz, $CDCl_3$)

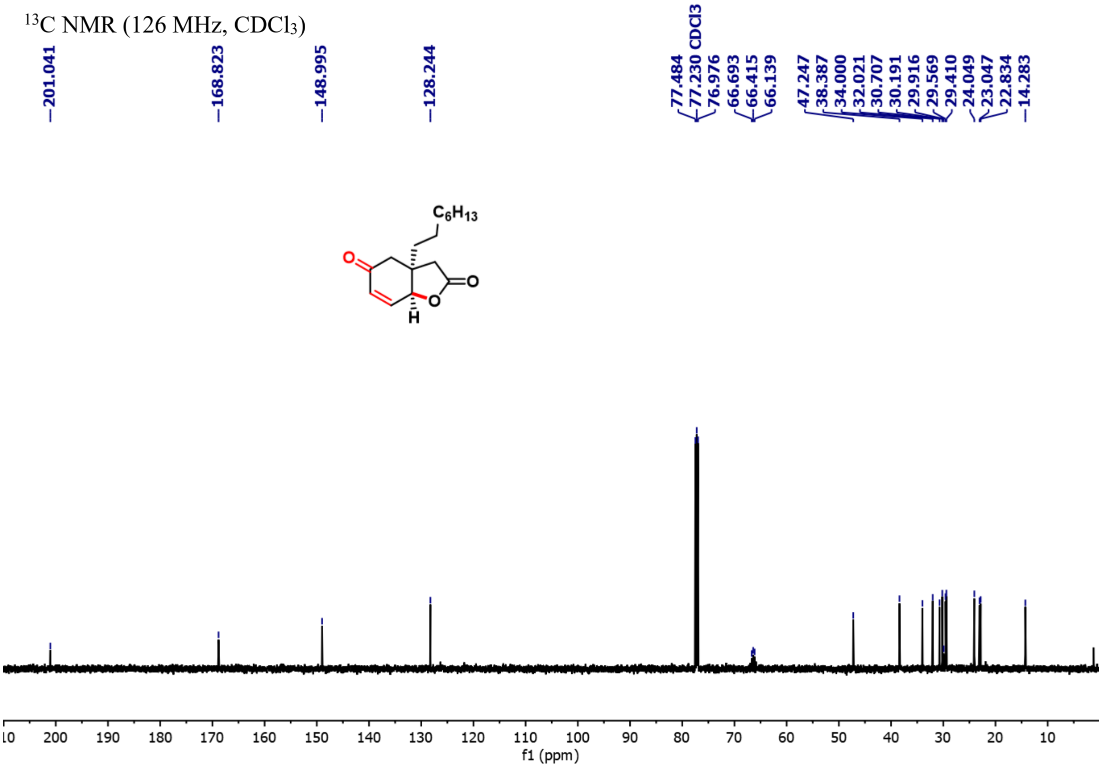

16. NMR Spectra of Alkenoic Acid

Compound 5f

2-(1-methylcyclohex-2-en-1-yl)acetic acid

^{1}H NMR (400 MHz, $CDCl_3$)

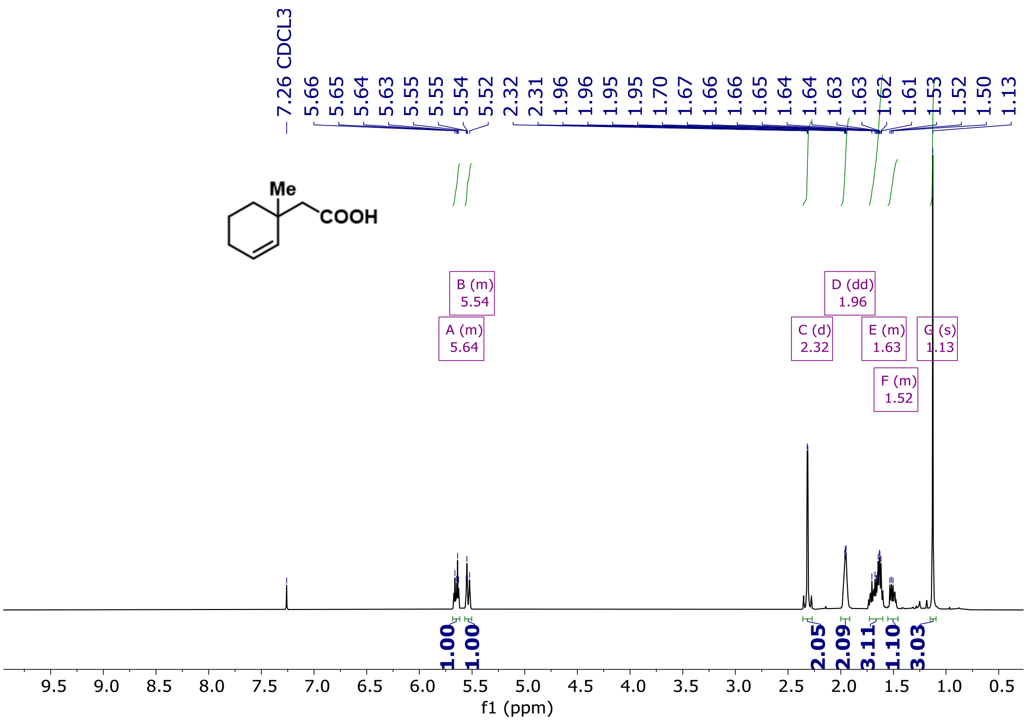

^{13}C NMR (101 MHz, $CDCl_3$)

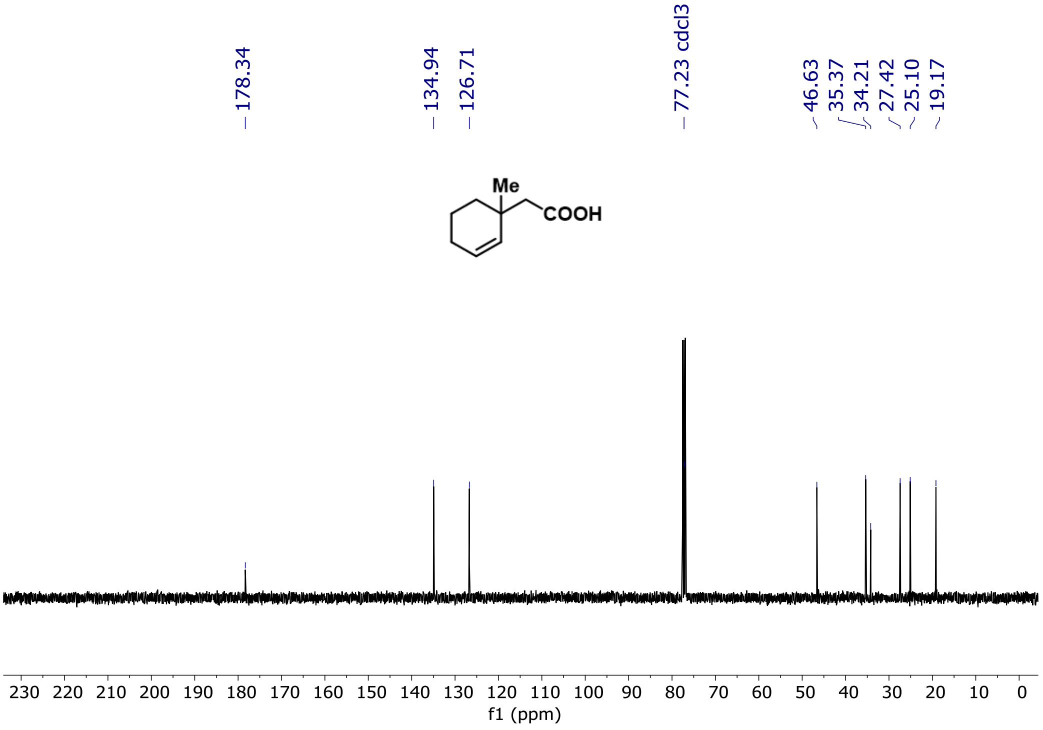

Supplement: Supplementary file 1 — Supplementary Tables 1–7, discussion and Figs. 1–15. [file 41557_2023_1295_MOESM1_ESM.pdf]
